# Prognostic value of disulfidptosis-associated genes in gastric cancer: a comprehensive analysis

**DOI:** 10.3389/fonc.2025.1512394

**Published:** 2025-03-04

**Authors:** Jin Tang, Jing Yang, Long-Kuan Yin

**Affiliations:** ^1^ Department of Gastrointestinal Surgery, Affiliated Hospital of North Sichuan Medical College, Nanchong, Sichuan, China; ^2^ Department of Rheumatology and Immunology, Nanchong Central Hospital, Beijing Anzhen Hospital affiliated to Capital Medical University, Nanchong, Sichuan, China; ^3^ Department of Gastrointestinal Surgery, People’s Hospital of Fushun County, Zigong, Sichuan, China

**Keywords:** gastric cancer, disulfidptosis, long non-coding RNA, prognostic model, immune analysis

## Abstract

**Objective:**

Disulfidptosis is a newly identified type of nonapoptotic programmed cell death related to mechanisms such as ferroptosis, cuproptosis, pyroptosis, and necrotic apoptosis. This study explores the role of disulfidptosis-related long non-coding RNAs (DRLs) in gastric cancer and their potential as prognostic biomarkers.

**Method:**

We developed a prognostic model using DRL scores to classify patients based on disulfidptosis activity. We evaluated these scores for correlations with drug sensitivity, tumor microenvironment (TME) features, tumor mutational burden (TMB), and prognosis. Potential disulfidptosis-related signaling pathways were screened, identifying FRMD6-AS as a promising therapeutic target. FRMD6-AS expression was further validated using real-time fluorescent quantitative PCR (qRT-PCR).

**Results:**

The DRL-based prognostic model, established through univariate and multivariate Cox regression and LASSO regression analyses, outperformed traditional models in predicting prognosis. We divided samples into high-risk and low-risk groups based on DRL scores, finding that the low-risk group had a significantly higher survival rate (P < 0.05). A high-precision prediction model incorporating DRL scores, age, sex, grade, and stage showed strong predictive value and consistency with actual outcomes. High DRL scores correlated with higher TME scores and lower TMB. Key signaling axes identified were AC129507.1/(FLNA, TLN1)/FOCAL ADHESION and AC107021.2/MYH10/(TIGHT JUNCTION, VIRAL MYOCARDITIS, REGULATION OF ACTIN CYTOSKELETON). Potentially effective drugs, including BMS-754807, dabrafenib, and JQ1, were identified. FRMD6-AS emerged as a potential target for gastric cancer treatment.

**Conclusions:**

This study developed a novel prognostic model for gastric cancer using DRLs, identifying two key signaling axes related to prognosis. JQ1 may be an effective treatment, and FRMD6-AS could be a promising therapeutic target.

## 1 Introduction

Gastric cancer is the fifth most common malignancy and ranks among the top three in terms of mortality; its risk factors include Helicobacter pylori infection, age, a high-salt diet and a low-vegetable and -fruit diet (1). Geographically, East Asia has the highest incidence of stomach cancer, followed by Eastern and Central Europe (2). Gastric cancer is generally diagnosed late, which may be related to the lack of clinical symptoms in the early stage of gastric cancer (3). The 5-year survival rate of patients with gastric cancer in Japan is approximately 70% (4), while it is approximately 20% lower in many other parts of the world; this may be related to overdiagnosis in Japan. The discovery of numerous new markers for gastric cancer highlights the great heterogeneity of this tumor, which may indicate the need to develop individualized treatment strategies for patients in the future (5).

The metabolic reprogramming of tumor cells leads to a high dependence on nutrients such as glucose (6). SLC7A11 (also known as xCT) is a cystine/glutamate antiporter (7) that is highly expressed in tumor cells and is an important pathway for cancer cell survival. Abnormal accumulation of disulfide bonds in cells with high SLC7A11 expression under glucose starvation conditions induces a novel form of cell death distinct from ferroptosis, cuproptosis, pyroptosis, and necrotic apoptosis, which is called disulfidptosis (8). Disulfidptosis can be triggered by the use of glucose transporter (GLUT) inhibitors and can inhibit cancer growth (9). Novel biomarkers related to disulfidptosis can be used for the clinical diagnosis, prognosis prediction and treatment of liver cancer (10). Knowledge on disulfidptosis may contribute to the development of new anticancer treatments (11).

**Figure 1:**
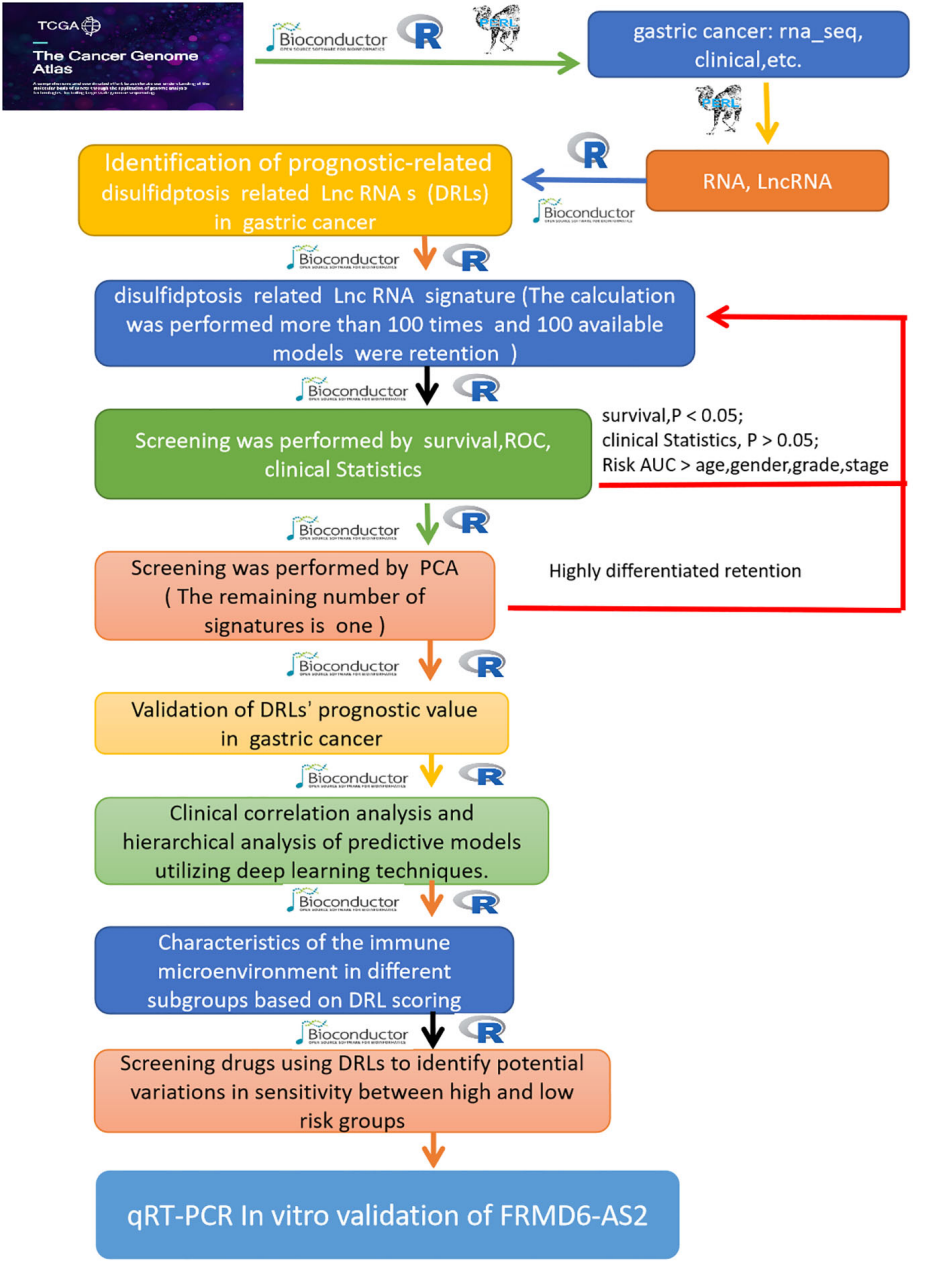
The complete pipeline of the study.

Long noncoding RNAs (lncRNAs), although largely unable to be converted into proteins, play an important role in a variety of cellular and physiological functions (12). LncRNAs regulate disulfidptosis and influence the tumor immune microenvironment and chemotherapy resistance (13). LncRNAs associated with disulfidptosis may be related to the prognosis, immune features and drug response of a variety of tumors, such as breast cancer (14), liver cancer (15), prostate cancer (16) and endometrial cancer (17). In recent years, numerous studies have shown that long noncoding RNAs (lncRNAs) play a key role in the occurrence of gastric cancer (18). Some studies have shown that LncRNA SNHG3 can promote proliferation and distant metastasis of gastric cancer cells through via the miRNA139-5p/MYB axis (19). LncRNA SNHG6 can participate in cisplatin resistance and development of gastric cancer through miR-1297/BCL-2 axis (20). To further explore the relationships between lncRNAs associated with disulfidptosis and gastric cancer, this study integrated the sequencing data of gastric cancer tissues and adjacent tissues from The Cancer Genome Atlas database (specifically, the TCGA (21) dataset). In addition, 24 disulfidptosis-related genes (DRGs)8 (GYS1, NDUFS1, OXSM, LRPPRC, NDUFA11, NUBPL, NCKAP1, RPN1, SLC3A2, SLC7A11, ACTN4, ACTB, CD2AP, CAPZB, DSTN, FLNA, FLNB, INF2, QGAP1, MYH10, MYL6, MYH9, PDLIM1, TLN1) were identified and analyzed. To explore the mechanism and potential therapeutic targets of the disulfidptosis-related lncRNAs (DRLs) involved in the occurrence and development of gastric cancer. The detailed analysis workflow is as shown in **Figure 1**.

## 2 Materials and methods

### 2.1 Data acquisition and processing

First, we obtained data from the TCGA database (https://portal.gdc.cancer.gov/) and organized the related gastric cancer (GC) gene expression data, clinical data, mutation data and copy number data. The data were processed and analyzed using the limma, dplyr, ggalluvial, and ggplot2 packages in R software (version 4.1.3) for data organization, and the survival, caret, glmnet, survminer, and timeROC packages for model screening. Additionally, Perl software (version 5.30.0) was utilized for data processing and analysis. First, the expression matrix for the gastric cancer samples was generated. Then, the lncRNA and mRNA expression matrices were generated, and the disulfidptosis mRNA expression matrix and disulfidptosis lncRNA expression matrices were compared to identify the DRLs. The correlations between disulfidptosis-related mRNAs and disulfidptosis-related lncRNAs were subsequently calculated.

### 2.2 Screening of lncRNAs associated with disulfidptosis and establishment and analysis of DRL signatures

GC-related data were extracted from the TCGA, DRGs were identified, and a comprehensive analysis was carried out. The lncRNAs associated with the DRGs were identified via R software, and the significantly related lncRNAs were subsequently screened via univariate analysis. Then, univariate and multivariate Cox regression (22) analysis and LASSO regression analysis (23–28) were performed to establish a new prediction model; our filtering conditions were more stringent in these analyses (P < 0.0005). We randomly grouped the samples and performed more than 100 iterations (in practice, a model can be applied only 3–5 times) with 100 completely different prediction models. Then, survival analysis, receiver operating characteristic (ROC) (29) curve analysis, clinical statistics, and principal component analysis (PCA) (30) were used to confirm a valid signature. The expression formula (31) is as follows, where Coefi is the coefficient and Xi is the expression quantity of the lncRNA: Riskscore $=\sum_{i=1}^{n}{\text{Coef}}_{i}*\text{X}$ . Finally, the correlations between the DRGs and lncRNAs were analyzed, and a heatmap was generated.

### 2.3 Verification of the DRL signature

R software was used to conduct subgroup analysis of all samples related to disulfidptosis genes in the TCGA database. First, the samples were randomly divided into a training set and a testing set, with 204 samples in the training set and 203 samples in the testing set. The baseline characteristics are presented in **Table 1**. Second, the two subgroups were subjected to survival analysis according to receiver operating characteristic (ROC) curves risk curves and survival status. The lncRNAs of the prediction model were subsequently analyzed via cluster analysis, and a heatmap was drawn. Finally, progression-free survival (PFS) analysis was carried out to further verify the value of the prediction model.

**Table 1:** Clinical baseline characteristics of each subgroup.

Covariates	Type	Test	Train	Pvalue
Age	≤65	99 (48.77%)	84 (41.18%)	0.1617
	>65	103 (50.74%)	118 (57.84%)	
	unknow	1 (0.49%)	2 (0.98%)	
Gender	FEMALE	76 (37.44%)	68 (33.33%)	0.4459
	MALE	127 (62.56%)	136 (66.67%)	
Grade	G1	4 (1.97%)	8 (3.92%)	0.2756
	G2	68 (33.5%)	76 (37.25%)	
	G3	128 (63.05%)	114 (55.88%)	
	unknow	3 (1.48%)	6 (2.94%)	
Stage	Stage I	25 (12.32%)	30 (14.71%)	0.4743
	Stage II	65 (32.02%)	57 (27.94%)	
	Stage III	87 (42.86%)	80 (39.22%)	
	Stage IV	16 (7.88%)	23 (11.27%)	
	unknow	10 (4.93%)	14 (6.86%)	
T	T1	7 (3.45%)	14 (6.86%)	0.2304
	T2	43 (21.18%)	43 (21.08%)	
	T3	97 (47.78%)	82 (40.2%)	
	T4	52 (25.62%)	61 (29.9%)	
	unknow	4 (1.97%)	4 (1.96%)	
M	M0	181 (89.16%)	181 (88.73%)	0.5791
	M1	11 (5.42%)	15 (7.35%)	
	unknow	11 (5.42%)	8 (3.92%)	
N	N0	60 (29.56%)	61 (29.9%)	0.7885
	N1	58 (28.57%)	50 (24.51%)	
	N2	38 (18.72%)	39 (19.12%)	
	N3	38 (18.72%)	44 (21.57%)	
	unknow	9 (4.43%)	10 (4.9%)	

### 2.4 Comparison of the accuracy of the risk score, age, sex, grade and stage in predicting the prognosis of gastric cancer and the establishment of a nomogram.

Univariate Cox analysis, multivariate Cox analysis, ROC curve analysis and Concordance index (32) (C-index) analysis were carried out for age, sex, grade, stage and risk score, respectively, and a nomogram (33) was constructed; then, the predictive accuracy of nomogram was verified via a calibration curve.

### 2.5 Stratified analysis of clinical features and PCA

Survival analysis was performed based on the risk score across various clinical characteristics, including age, G stage, M stage, sex, N stage, overall stage, and T stage, to evaluate the accuracy of the risk score in assessing clinical features of gastric cancer. Subsequently, principal component analysis (PCA) was conducted on four distinct sample sets: all gene samples, disulfidptosis gene samples, disulfidptosis LncRNA samples, and risk LncRNA samples. The results demonstrated that the risk score exhibited superior discriminative ability in distinguishing gastric cancer samples, further validating its prognostic significance.

### 2.6 Differential gene expression analysis, gene ontology and Kyoto encyclopedia of genes and genomes analysis, and gene set enrichment analysis

The samples were grouped according to the risk score of the prediction model, and differential expression analysis was conducted. Significant differentially expressed genes were screened out, and GO (34–39), KEGG (34, 35, 40) and GSEA (34, 35, 37, 41, 42) analysis were conducted on these differentially expressed genes to identify the potential related pathways and functions.

### 2.7 Analysis of the correlation between the risk score and gastric cancer immune features

To comprehensively assess the relevance of the risk score to immune features in gastric cancer, the tumor microenvironment (TME) (43), CIBERSORT (34, 35, 44), immCor, immFunction, tumor mutational burden (TMB) (34, 45, 46), TMB stratified analysis, and Tumor Immune were analyzed by predictive model Dysfunction and Exclusion (TIDE) (47) analysis; Fully evaluate the relevance of risk models to gastric cancer immunity.

### 2.8 Drug susceptibility analysis

Drugs that may have significant differences in efficacy in gastric cancer treatment were screened via a predictive model and the OncoPredict (48) package in R software.

### 2.9 Primer design

Primer design for genes FRMD6-AS2 and GAPDH (**Table 2**).

**Table 2:** The primers used were purchased from Qingke Biotechnology Co., Ltd. (China).

Genes	Primer sequence
FRMD6-AS2	forward primer 5′- ACTCAGAGGCCACACTAGAT-3’ reverse primer 5′-AGATTGGATGTTGGCACCC-3’
GAPDH	forward primer 5′-ACATCGCTCAGACACCATG-3′ reverse rimer ′-TGTAGTTGAGGTCAATGAAGGG-3′

### 2.10 Cell culture

In this study, GES1 (BNCC337969) and HGC-27 (BNCC338546) cells were purchased from Beina Biology (China). GES1 cells were cultured in complete DMEM-H supplemented with 1% penicillin−streptomycin (Sigma, USA), and HGC-27 cells were cultured in complete RPMI-1640 supplemented with 1% penicillin−streptomycin (Sigma, USA).

### 2.11 RNA extraction and reverse transcription

The cell precipitate was collected, and 1 mL of TRIzol (CWBIO, China) was added, and the sample was mixed and incubated on ice for 5 min for cell lysis. Then, 200 μL of chloroform (High Crystal Chemical Industry, China) was added to the EP tube, which was shaken vigorously, left at room temperature for 3 min, and centrifuged at 12000 rpm at 4°C for 15 min, after which the supernatant was collected. Then, an equal volume of isopropyl alcohol (McLean, China) was added, mixed upside down, left for 10 min at room temperature, and centrifuged at 12000 rpm at 4°C for 10 min, after which the supernatant was discarded. Then, 1 mL of 75% ethanol (McLean, China) was added, the mixture was centrifuged at 12000 rpm at 4°C for 3 min, the supernatant was discarded, the lid was opened to allow air-drying, 30 μL of DEPC water was added, and the sample was stored at -80°C. Then, reverse transcription was performed with a 20 μL reaction volume. The amount of each component was 3 μL of RNA, 4 μL 5× Hifair^®^ II Buffer (11119ES60 yeasen), 2 μL Hifair^®^ II Enzyme Mix, 0.5 μL + 0.5 μL random primers N6 (50 μm) + or oligo (dT)18 (50 μM), and 20 μL RNase-free H2O. The samples were added to the EP tube according to the above reaction system, mixed with a pipette and put into a PCR (BIO-RAD T100) amplification apparatus. The program was set at 25°C for 5 min according to the following conditions: 42°C for 30 min; 85°C for 5 min; hold at -20°C.

### 2.12 qRT−PCR

The volume of the fluorescence quantitative PCR reaction system was 20 μL. The amount of each component was as follows: UGreener Flex qPCR 2X mix, 10 μL; forward primer (10 µM), 0.5 μL; reverse primer (10 µM), 0.5 μL; cDNA, 1 μL; and ddH_2_O, 8 μL. The samples were added to 96-well plates for fluorescent quantitative PCR according to the above reaction system and mixed successively. The samples were put into the fluorescent quantitative PCR instrument (ABI 7500, USA), and the program was set according to the following conditions: 45 cycles were carried out in the constant temperature stage is set at 95°C for 30 seconds (denaturation), followed by the cycling stage of 95°C for 10 seconds (denaturation) and 60°C for 30 seconds (annealing) for a total of 45 cycles. The melt stage consists of 95°C for 15 seconds (denaturation), 60°C for 1 minute (annealing), 95°C for 15 seconds (denaturation), and 60°C for 15 seconds (annealing).

### 2.13 Statistical analysis

In the bioinformatics analysis, statistical analysis was performed using R software (version 4.1.3), with a P-value of less than 0.05 considered statistically significant. For the experimental part, all data were replicated three times. Differences between two groups were compared using the Student t-test, and statistical analysis and graphing were conducted using GraphPad Prism 8.0 software. A P-value of less than 0.05 was considered statistically significant.

## 3 Results

### 3.1 Construction and validation of a disulfidptosis-related lncRNA prognostic signature in gastric cancer

**Figure 2:**
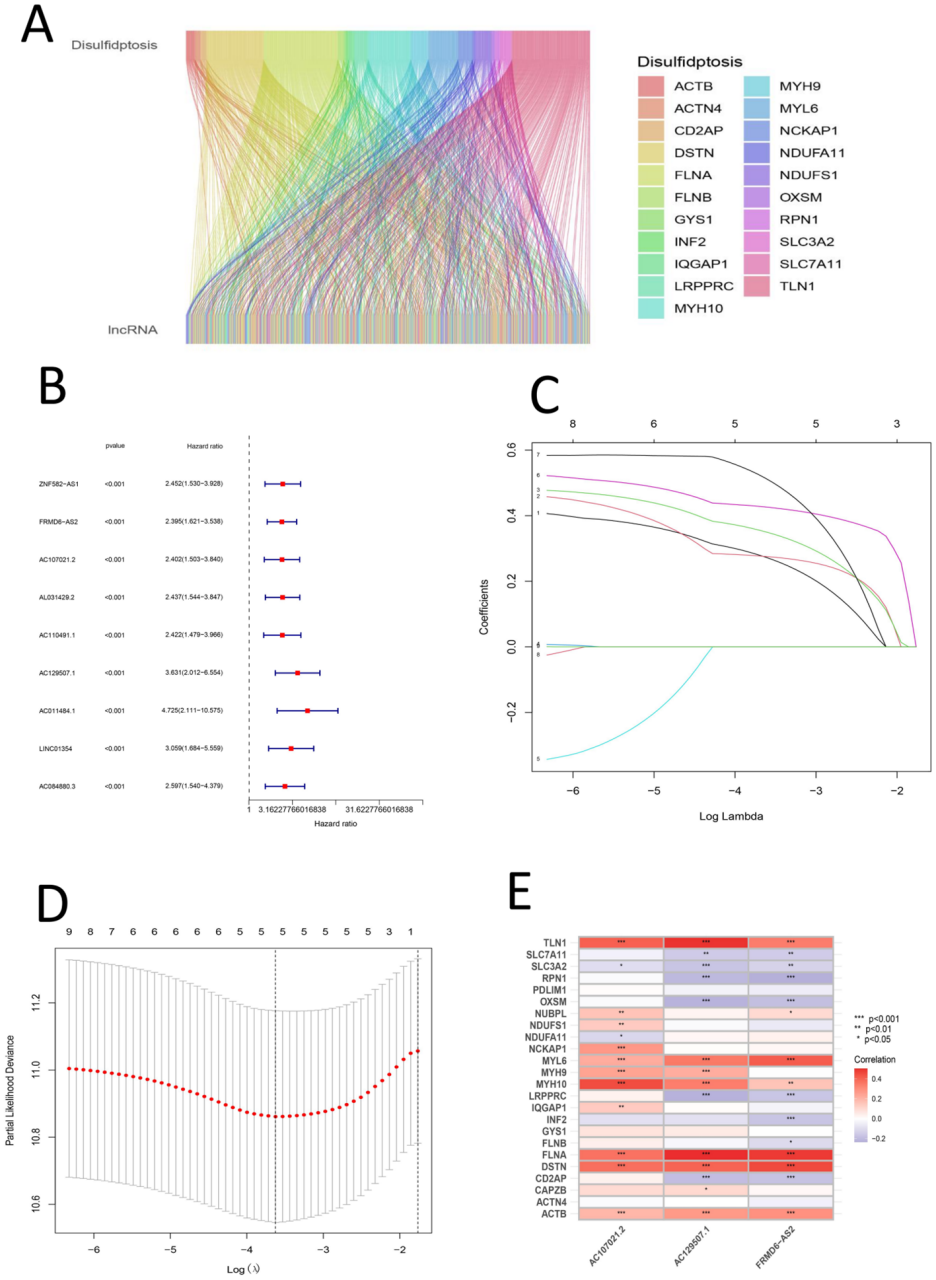
**(A)** Sankey diagram of the relationship between disulfide death gene and LncRNA **(B)** Univariate forest map of LncRNA with significant differences **(C)** Shows the lasso regression plot; c. Shows the lasso regression plot **(D)** Presents the cvfit plot of the lasso regression **(E)** Heat map of the relationship between DRGs and signature LncRNAs.

First, the expression matrix of the lncRNAs was extracted via the data matrix processed with R software (version 4.1.3) and Perl software (version 5.30.0). The correlations between lncRNAs and disulfidptosis genes were mapped via the R package ggalluvial (**Figure 2A**). The lncRNAs whose expression significantly differed between groups were screened via univariate Cox analysis and are displayed in a forest plot (**Figure 2B**). Subsequently, LASSO regression and multivariate Cox regression were used to construct a prognostic signature associated with disulfidptosis in gastric cancer patients (**Figures 2C, D**); the analysis included more than 100 iterations, and the final signature was evaluated via survival analysis, receiver operating characteristic (ROC) curve analysis, clinical statistics and PCA (**Table 3**). The formula for the signature risk score was as follows: risk score = (0.544735914395105 * AC107021.2 expression) + (0.705013376452246 * AC129507.1 expression) + (0.433534323181848 * ‘FRMD6-AS2’ expression); heatmap showing the correlation between the individual DRLs and the risk score (**Figure 2E**). Next, model lncRNAs and related DRGs were screened out (**Table 4**).

**Table 3:** Results of gastric cancer prognostic model constructed using subgroups analysis of disulfidptosis-related LncRNAs.

id	coef
AC107021.2	0.544736
AC129507.1	0.705013
`FRMD6-AS2`	0.433534

**Table 4:** List of associations between model LncRNAs and related disulfidptosis genes.

Disulfidptosis	cor	pvalue	Regulation
DSTN	0.402653447	1.72E-17	postive
FLNA	0.503576504	7.09E-28	postive
TLN1	0.495798994	6.02E-27	postive
DSTN	0.458610526	8.07E-23	postive
FLNA	0.485388579	9.69E-26	postive
MYL6	0.417863824	7.67E-19	postive
MYH10	0.452199524	3.71E-22	postive
TLN1	0.410087591	3.84E-18	postive

### 3.2 Evaluation of risk stratification and prognostic value of the DRL signature in gastric cancer

**Figure 3:**
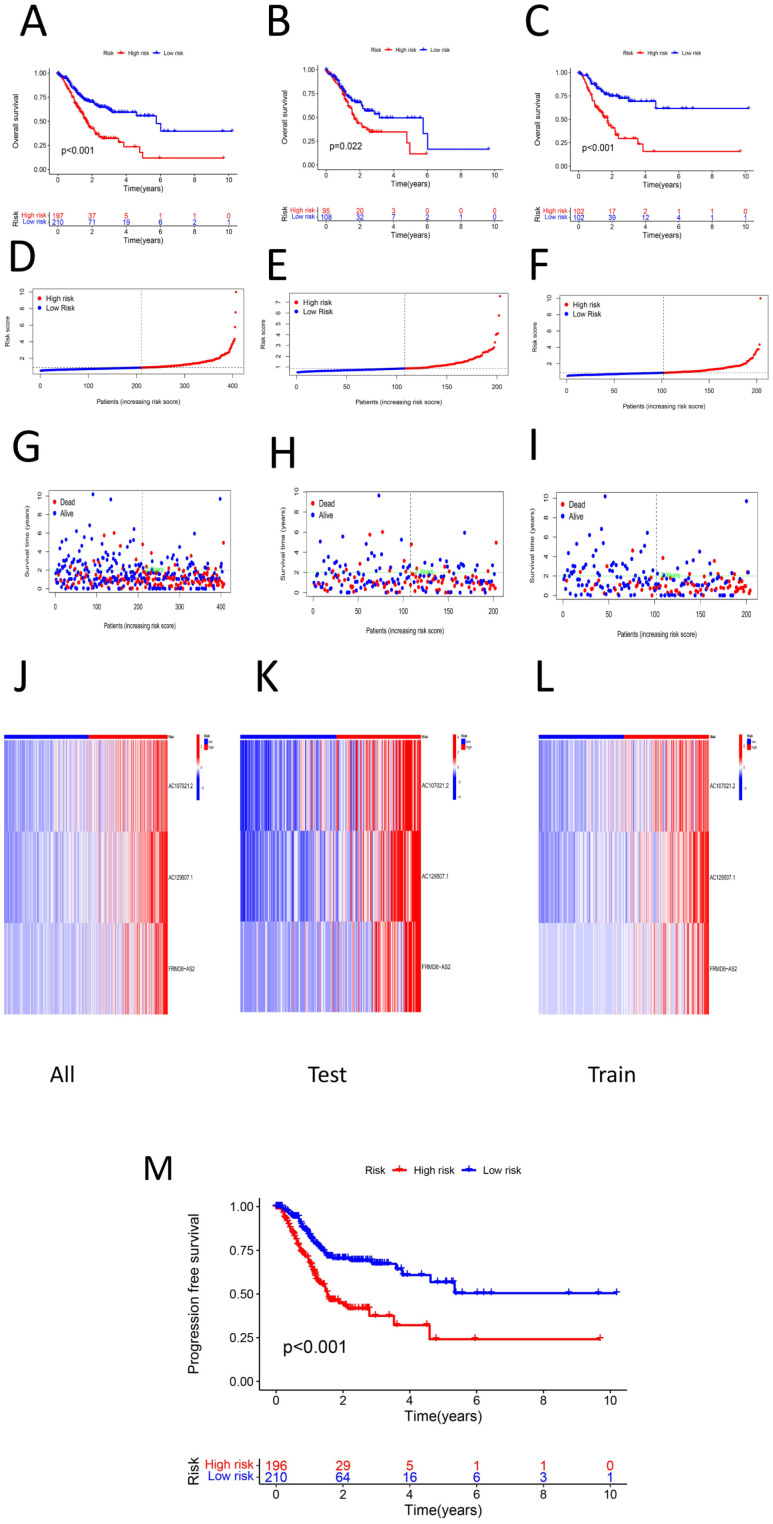
**(A)** Survival analysis of high-low risk group in total sample (P < 0.05) **(B)** survival analysis among high-low risk groups in the test group (P < 0.05) **(C)** Survival analysis among high-low risk groups in the training group (P < 0.05)**D.** risk score of the total sample **(E)** Risk score of the test group sample **(F)** Risk score of the training group sample **(G)** Survival time plot of the total sample **(H)** Survival time graph of the sample of the test group **(I)** Survival time graph of the training group samples **(J)** Prognosis of the total sample lncRNA signature heat map **(K)** Prognostic lncRNA signature heat map of the test group samples **L.** lncRNA signature heat map for the training group samples **M.** PFS between high and low risk groups (P < 0.05).

**Figure 4:**
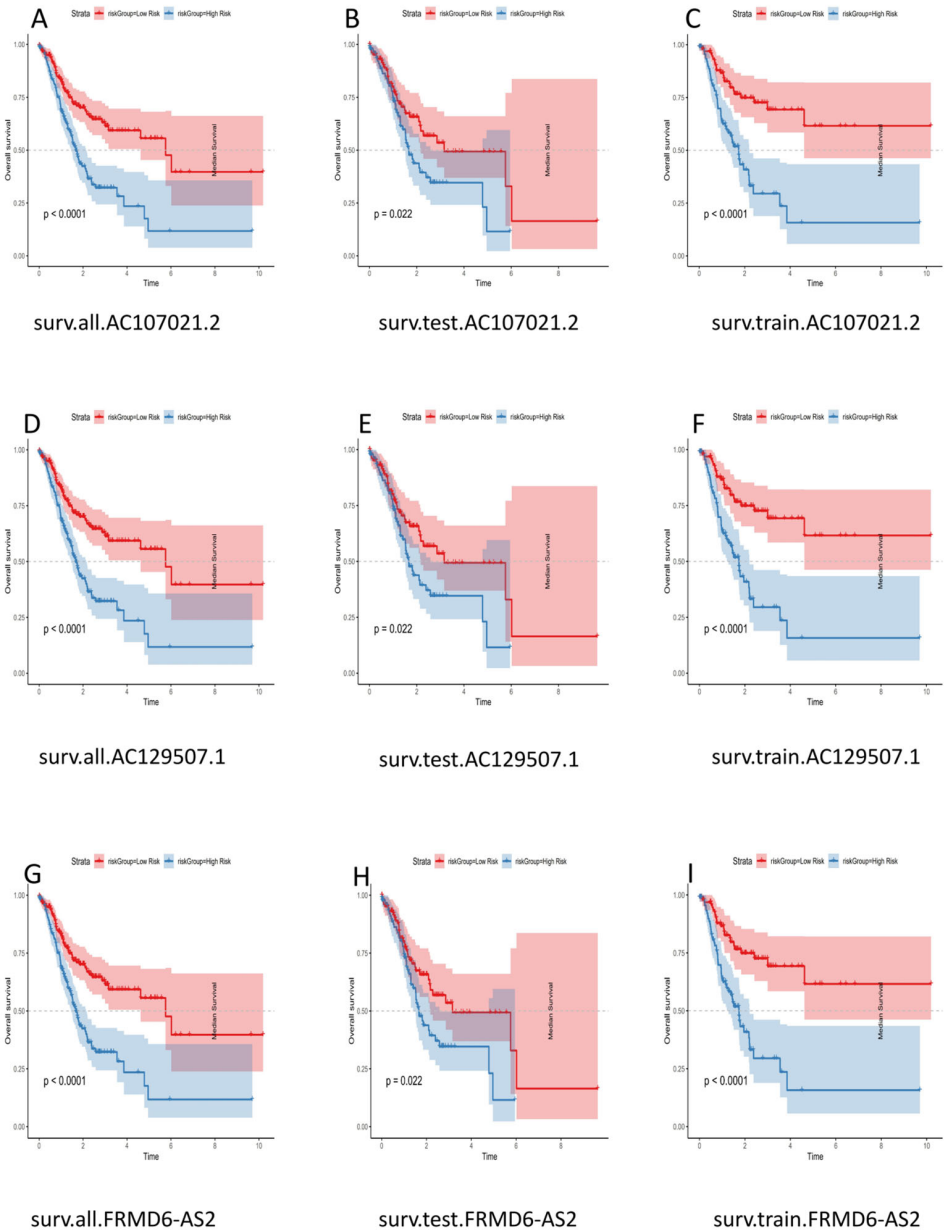
Survival Analysis of LncRNA Models. **(A)** surv.all.AC107021.2. **(B)** surv.test.AC107021.2. **(C)** surv.train.AC107021.2. **(D)** surv.all.AC129507.1. **(E)** surv.test.AC129507.1. **(F)** surv.train.AC129507.1 **(G)** surv.all.FRMD6-AS2. **(H)** surv.test.FRMD6-AS2. **(I)** surv.train.FRMD6-AS2.

The samples used to construct the DRL model were subsequently randomly divided into two groups of equal size (a test group and a training group). We used the DRL signature to score each sample for the entire cohort, the test group sample and the training group and divided the patients into high-risk and low-risk groups. Survival analysis, risk score analysis, survival time analysis, enrichment analysis, PFS (49) analysis were performed for each group. Survival analysis revealed that the high-risk patients in each group had a worse prognosis than the low-risk patients (P < 0.05) (**Figures 3A-C**). Risk score analysis revealed that the risk score trend of each group was generally consistent, the risk score of the low-risk group was less than 1, and the risk score of the high-risk group was greater than 1 (**Figures 3D-F**). Similarly, we conducted survival time analysis for each group and found that the survival time distribution of patients in each group was generally consistent: the low-risk group had a lower mortality rate than the high-risk group (**Figures 3G-I**). Through enrichment analysis and heatmapping of signature lncRNAs in each group, AC107021.2, AC129507.1 and FRMD6-AS2 were found to be high-risk lncRNAs. The PFS analysis revealed that the high-risk group had lower PFS rates at a given time point than did the low-risk group (**Figure 3M**). The median survival time (in years) is: 1.152055. Independent survival analysis for each lncrna model reveals that each lncrna can independently influence gastric cancer prognosis (**Figures 4A-I**).

### 3.3 Assessment of risk score and clinical factors in prognostic signature for gastric cancer

**Figure 5:**
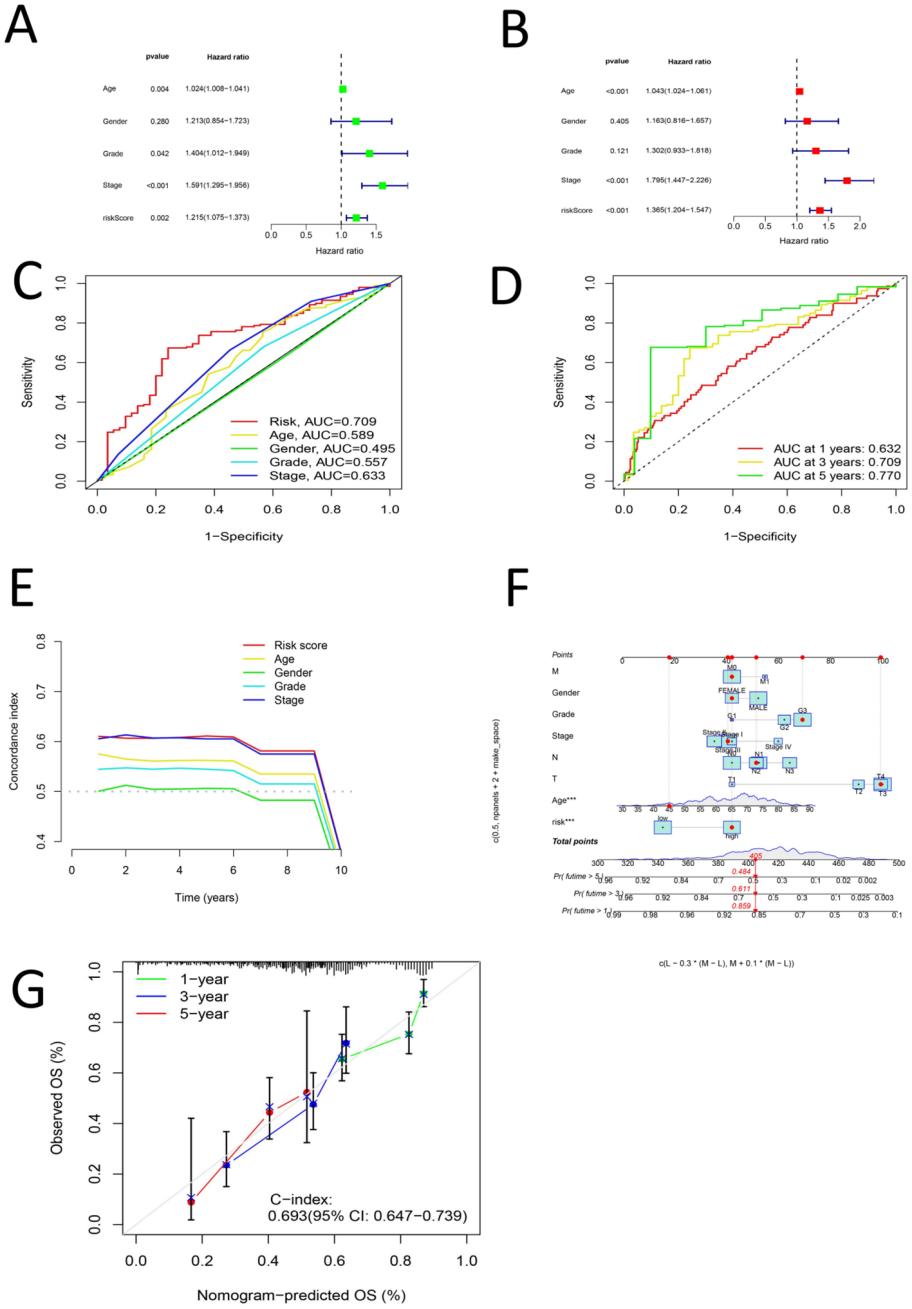
**(A)** Univariate COX analysis of Age, Grade, Stage and Risk score **(B)** Multi-factor COX analysis of Age, Grade, Stage and Risk score **(C)** ROC analysis of Age, Grade, Stage and Risk score **(D)** ROC curve for 1, 3 and 5 years of Risk Score **(E)** C curve for Age, Grade, Stage and Risk score **(F)** norm plot for T, N, M, Age, Grade, Stage, and Risk score **(G)** Shows a calibration curve.

First, univariate Cox analysis, multivariate Cox analysis, clinical characteristic ROC curve analysis, survival ROC curve analysis and concordance index analysis were performed for the risk score and clinical characteristics of age, sex, grade and stage, respectively. Univariate and multivariate Cox analyses revealed that age, grade, stage and the risk score had significant effects on the prognosis of gastric cancer (P < 0.05), whereas sex had no significant effect on the prognosis of gastric cancer (P > 0.05) (**Figures 5A, B**). Clinical characteristic ROC curve analysis revealed that the area under the curve (AUC) (50–52) value of age, grade, stage and the risk score was > 0.5, whereas the AUC value of sex was < 0.5 (**Figure 5C**). ROC curve analysis of survival years revealed that the AUC values at 1, 3 and 5 years were all greater than 0.6 (**Figure 5D**). Concordance index analysis revealed that the risk score was the main factor influencing the prognosis of gastric cancer, whereas sex had the least effect on the prognosis of gastric cancer (**Figure 5E**). The risk score, age, sex, grade, stage, M stage, N stage and T stage were used to establish a nomogram, which revealed that the risk score and age were independent influencing factors for the prognosis of gastric cancer (**Figure 5F**). Finally, calibration curves were generated to verify the accuracy of the nomogram predictions (**Figure 5G**).

### 3.4 Survival analysis by age, G stage, M stage, N stage, gender, and T stage in gastric cancer risk groups

**Figure 6:**
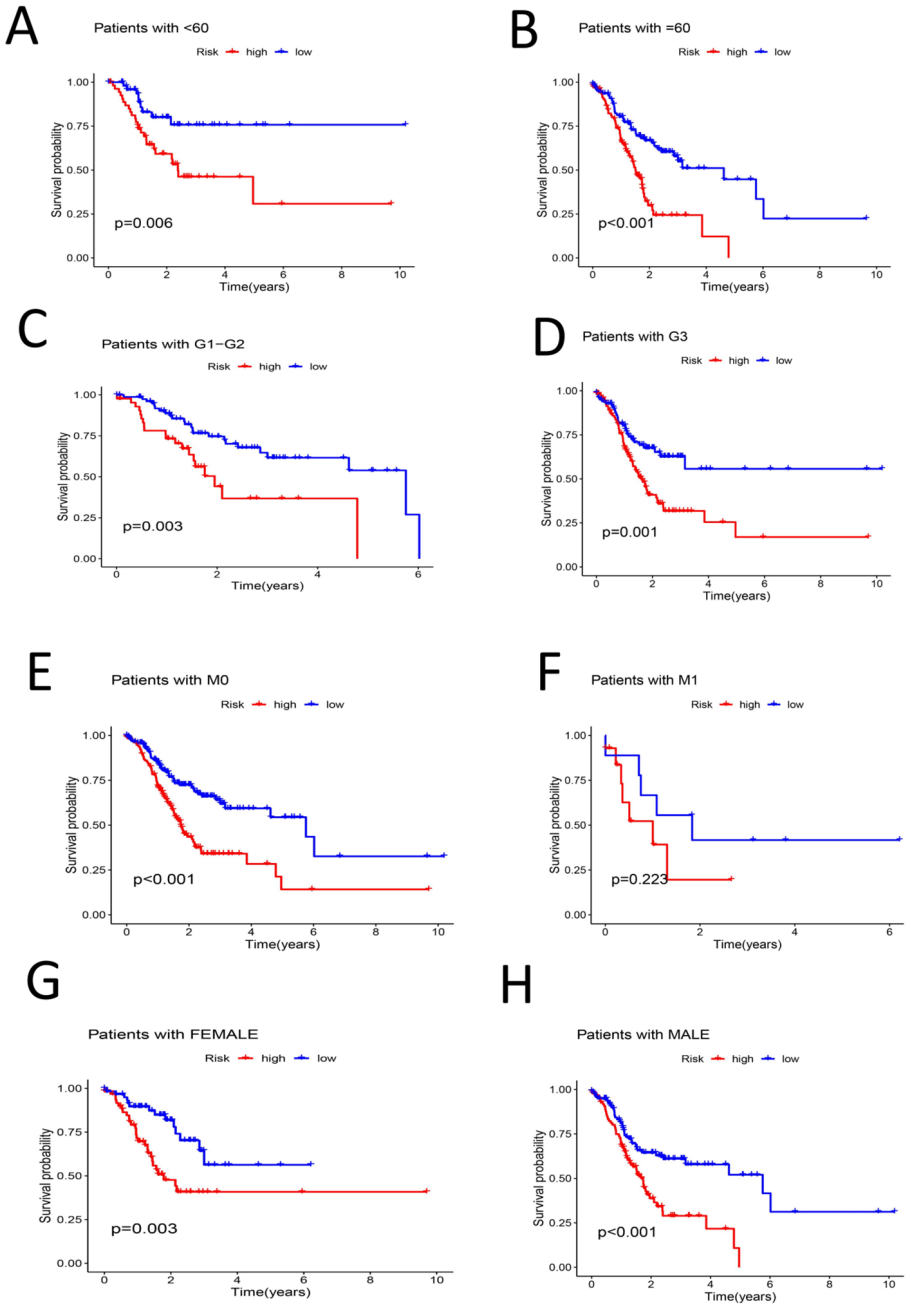
**(A)** Survival analysis among high-low risk groups in samples younger than 60 years (P < 0.05) **(B)** survival analysis among high-low risk groups in samples older than or equal to 60 years (P < 0.05) **(C)** Survival analysis of G1-G2 samples among high-low risk groups (P < 0.05) **(D)** Survival analysis of the high-low risk group of the G3 sample (P < 0.05) **(E)** Survival analysis in the high-low risk group of sample M0 (P < 0.05) **(F)** Survival analysis between high-low risk groups in sample M1 (P > 0.05) **(G)** Survival analysis among high-low risk groups of Female sample (P < 0.05) **(H)** Survival analysis among high-low risk groups in a Male sample (P < 0.05).

**Figure 7:**
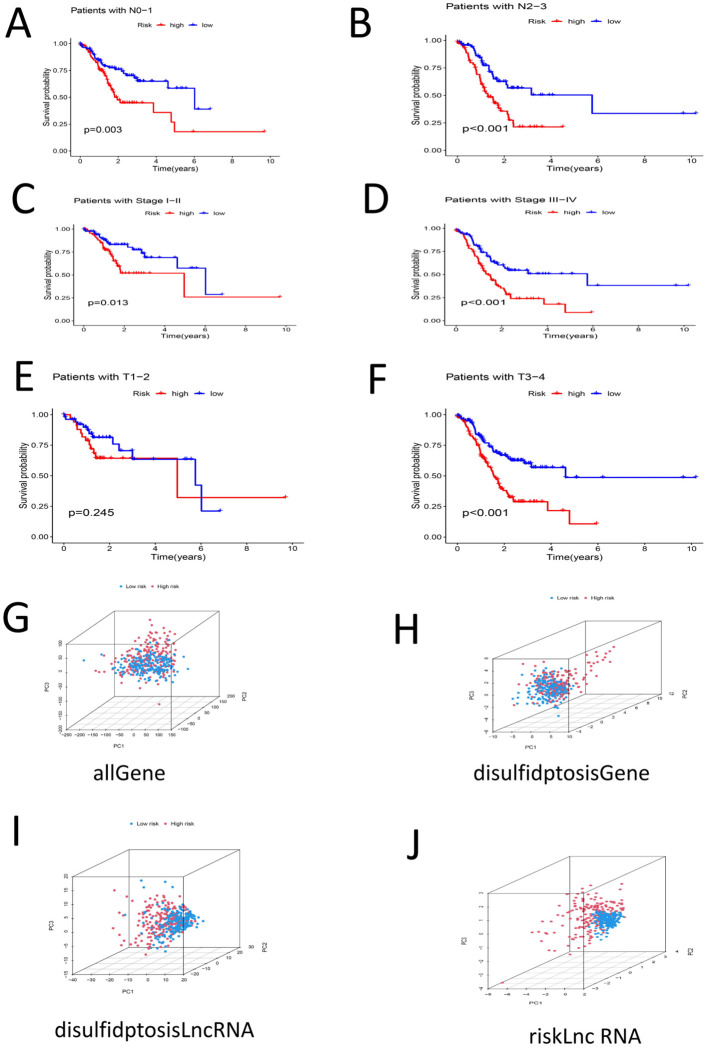
**(A)** Survival analysis among high-low risk groups in N0-N1 samples (P < 0.05) **(B)** Survival analysis among high-low risk groups in N2-N3 samples (P < 0.05) **(C)** Stage |-‖ ‖ Survival analysis of samples in high-low risk groups (P < 0.05) **(D)** Survival analysis of Stage III-IV samples of high-low risk group (P < 0.05) **(E)** Survival analysis of high-low risk group in samples T1-T2 (P > 0.05) **(F)** Survival analysis between high-low risk groups in T3-T4 samples (P < 0.05) **(G)** PCA profile of the total sample **(H)** PCA distribution of DRG samples **I** PCA distribution map of DRL sample **(J)** PCA distribution map of Risk lncRNA sample.

The patients were then grouped by age(< 60 years old and ≥ 60 years old). Survival analysis of the two groups revealed that the survival rate of the high-risk group was lower than that of the low-risk group among each of these age groups (P < 0.05) (**Figures 6A, B**). The samples were divided into G1–G2 groups and G3 groups by G stage, and survival analysis was conducted on the two groups. There was no significant difference in the survival rate between the high- and low-risk groups among the G1–G2 group (P > 0.05). However, there was a significant difference in the survival rate between the high- and low-risk groups among the G3 group (P < 0.05) (**Figures 6C, D**). We divided the samples by M stage into M0 and M1 groups; survival analysis of the M0 group revealed significant differences between the high- and low-risk groups (P < 0.05). There was no significant difference in survival between the high- and low-risk groups among the M1 group (P > 0.05) (**Figures 6E, F**). The patients were divided into a female group and a male group, and survival analysis revealed significant differences between the high- and low-risk groups for both females and males. The survival rates of the high-risk groups were lower than those of the low-risk groups (P < 0.05) (**Figures 6G, H**). By N stage, the high-risk group was divided into the N0-1 group and the N2-3 group. Survival analysis revealed that there was a significant difference between the high- and low-risk groups among patient in these subgroups and that the high-risk groups had lower survival rates (P < 0.05) (**Figures 7A, B**).The patients were divided by stage into Stage I-II and Stage III-IV groups. The survival analysis of the high- and low-risk samples in each group revealed significant differences, and the survival rates of the high-risk groups were lower (P < 0.05) (**Figures 7C, D**). In the T stage, the samples were divided into a T1-2 group and a T3-4 group. The survival analysis of the high- and low-risk samples in each group revealed that there was no significant difference in the survival rate between the high- and low-risk groups in the T1-2 subgroup (P > 0.05). The survival rate of the high- and low-risk groups in the T3-4 subgroup was significant, and the survival rate of the high-risk subgroup was lower (P < 0.05) (**Figures 7E, F**). PCA of the all-gene, DRG, DRL and risk lncRNA groups revealed that the risk-related lncRNA model presented the greatest degree of differentiation among samples (**Figures 7G-J**).

### 3.5 differential gene expression and pathway enrichment analysis in gastric cancer risk scores

**Figure 8:**
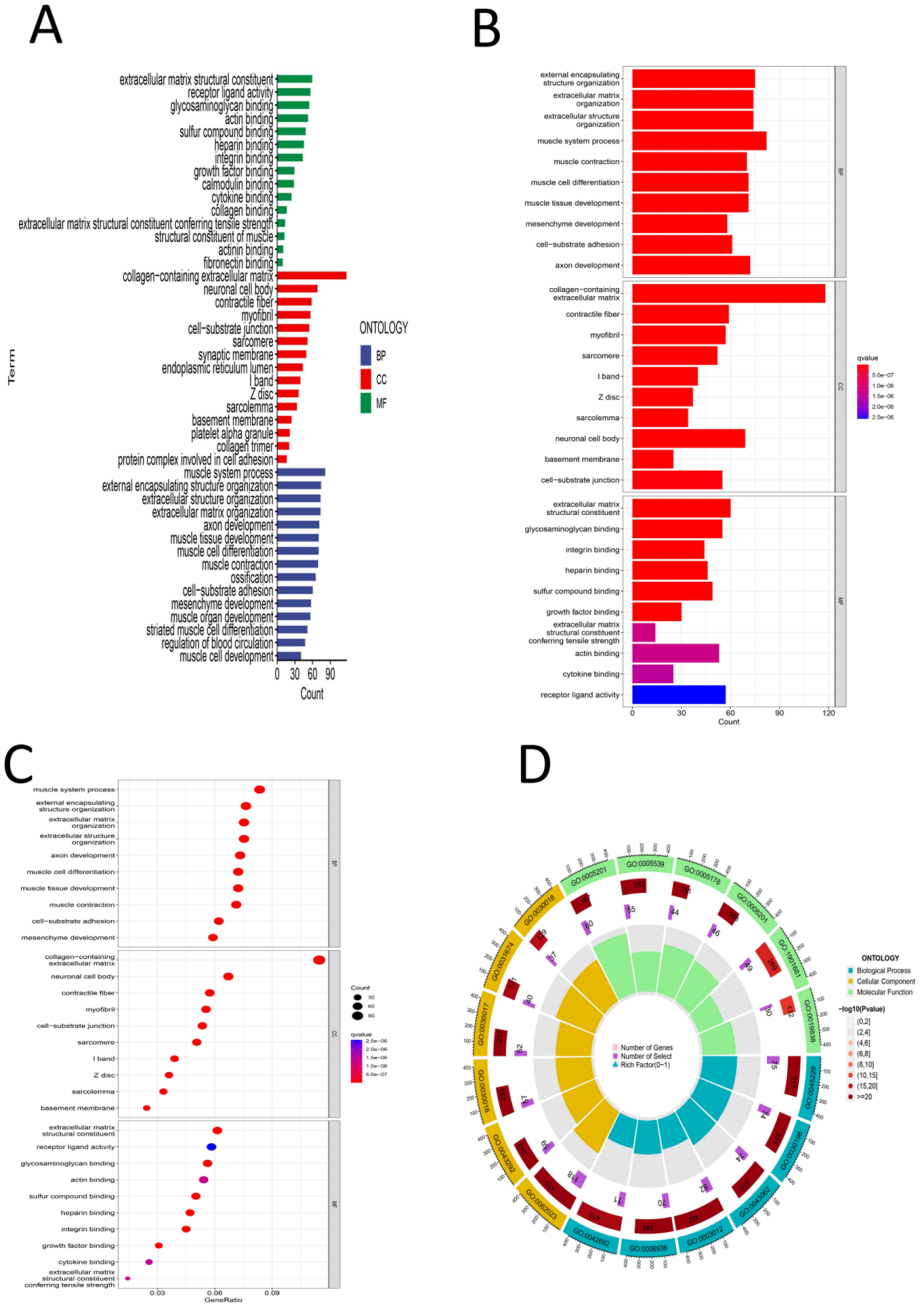
**(A)** GO color bar chart of the analysis results **(B)** GO bar chart of the analysis results **(C)** Bubble chart of GO analysis results **(D)** Loop diagram of GO analysis result.

**Figure 9:**
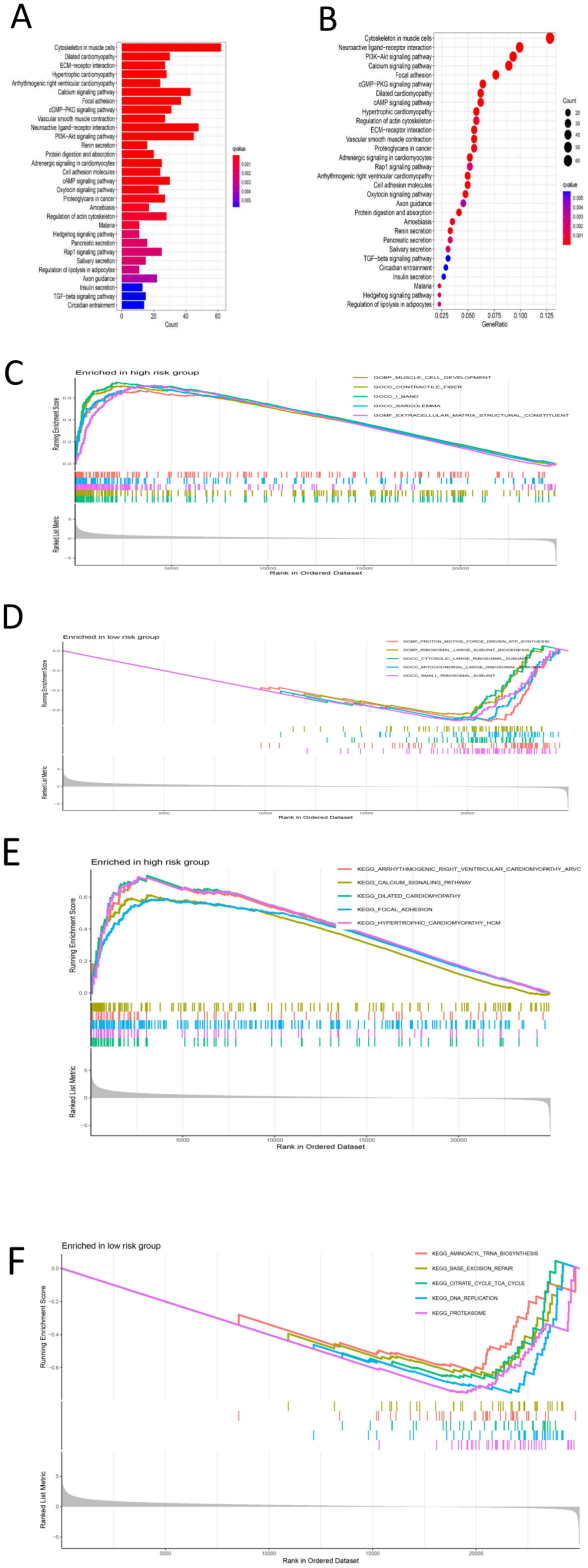
**(A)** Bar chart of KEGG’s analysis results **(B)** Bubble chart of KEGG analysis results **(C)** GSEA-GO analysis results of high-risk group results distribution map **(D)** GSEA-GO analysis results of low-risk group results distribution map **(E)** Distribution of results of high-risk group analyzed by GSEA-KEGG **(F)** Result distribution map of GSEA-KEGG analysis results of low-risk group.

First, differential expression analysis was conducted on samples grouped according to the risk score, and significantly differentially expressed genes were identified. GO analysis of the differentially expressed genes revealed that the main enriched biological process (BP) were enriched external encapsulating structure organization, extracellular matrix organization, extracellular structure organization, muscle system process, etc. The main enriched cellular component (CC terms were collagen-containing extracellular matrix, contractile fiber, myofibril, sarcomere, I band, etc. The main enriched molecular function (MF) terms were extracellular matrix structural constituent, glycosaminoglycan binding, heparin binding, integrin binding, sulfur compound binding, etc. (**Figures 8A-D**), (**Supplementary 1**). KEGG analysis revealed that the main enriched pathways were the cytoskeleton in muscle cells, dilated cardiomyopathy, ECM-receptor interaction, hypertrophic cardiomyopathy, arrhythmogenic right ventricular cardiomyopathy and other pathways (**Figures 9A, B**) (**Supplementary 2**). GSEA revealed enrichment of pathways and functions including DILATED CARDIOMYOPA, HYPERTROPHIC CARDIOMYOPATHY HCM, PROTEASOME, FOCAL ADHESION, ARRHYTHMOGENIC RIGHT VENTRICULAR CARDIOMYOPATHY ARVC, CALCIUM SIGNALING PATHWAY, ECM RECEPTOR INTERACTION, DNA REPLICATION, CITRATE CYCLE TCA CYCLE, AMINOACYL TRNA BIOSYNTHESIS (**Figures 9C-F**) (**Supplementary Materials 3, 4**). As shown in **Supplementary Material 3**, we found that FLNA is enriched in the FOCAL ADHESION pathway and that TLN1 is enriched in the FOCAL ADHESION pathway. MYH10 was enriched in the TIGHT JUNCTION, VIRAL MYOCARDITIS, and REGULATION OF ACTIN CYTOSKELETON pathways, and as shown in **Table 2**, FLNA was positively correlated with AC129507.1; TLN1 was significantly positively correlated with AC129507.1; and MYH10 was significantly positively correlated with AC107021.2. In summary, our differential gene expression analysis identified key biological processes and molecular functions linked to the risk score. GO analysis revealed enrichments in extracellular matrix and muscle system processes, while KEGG and GSEA pinpointed important pathways like cytoskeleton dynamics and cardiomyopathy. Proteins FLNA, TLN1, and MYH10 were strongly associated with specific DRLs and crucial pathways such as focal adhesion and tight junctions. These insights enhance our understanding of gastric cancer prognosis and suggest potential therapeutic targets.

### 3.6 Differences in tumor microenvironment and immune cell profiles between high- and low-risk groups

**Figure 10:**
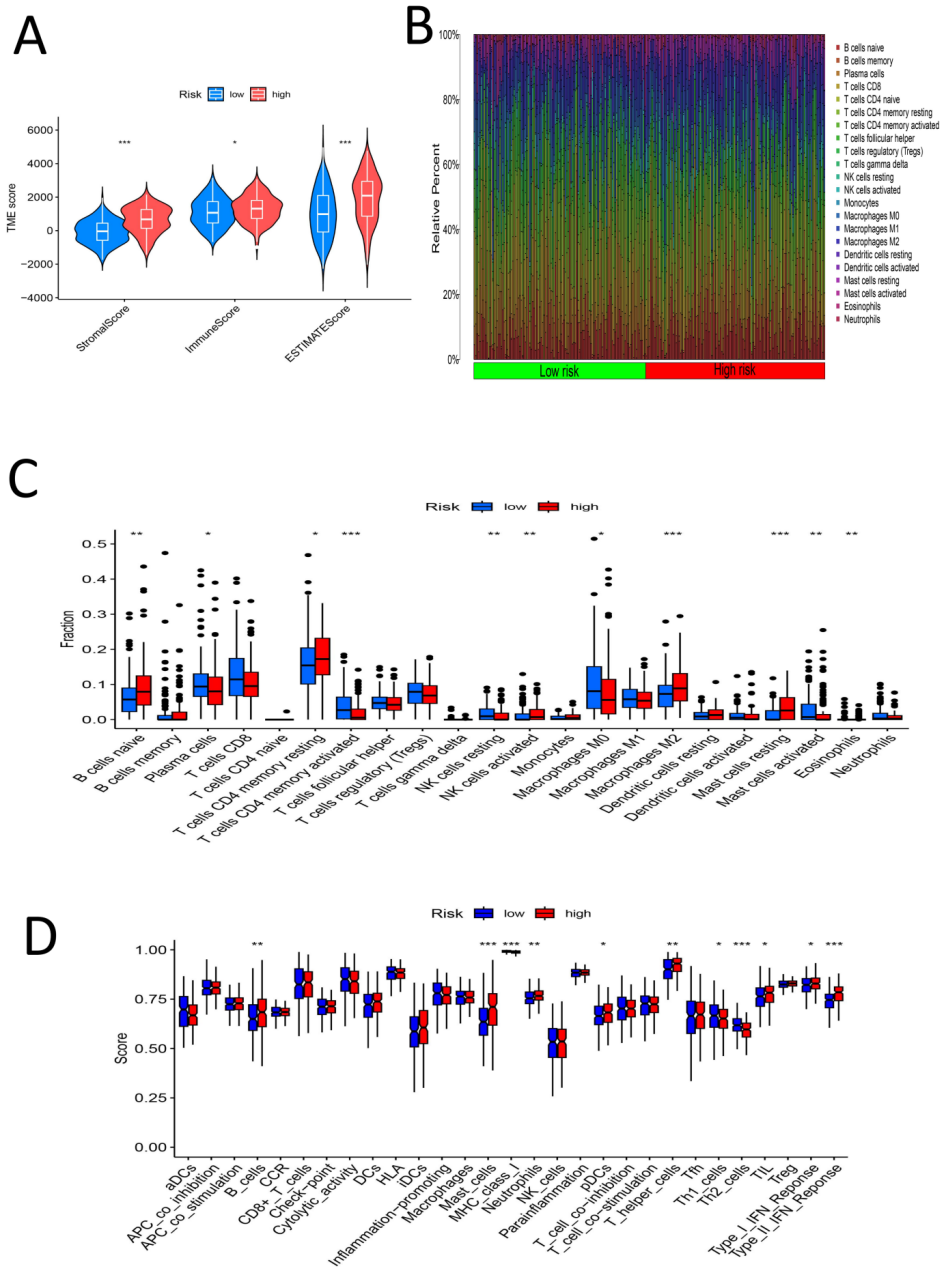
**(A)** TME-score violin chart for the high-low risk group **(B)** Histogram of percentage distribution of immune cells in the high-low risk group **(C)** Box chart of difference analysis of immune cells in high-low risk group **(D)** Box chart for analysis of differences in immune function between high and low risk groups (*, P < 0.05; **, P < 0.01; ***, P < 0.001).

**Figure 11:**
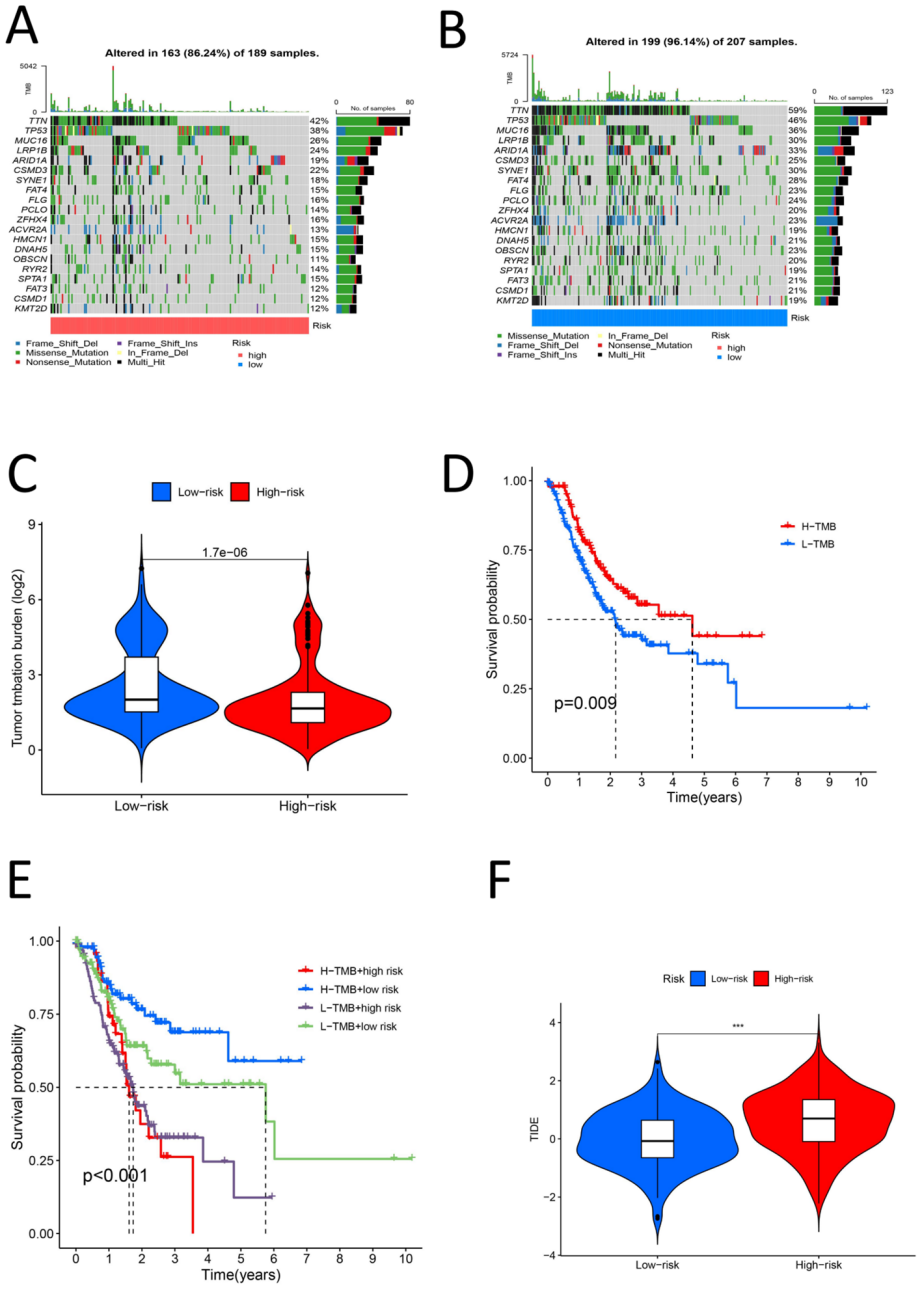
**(A)** High risk group TMB waterfall map **(B)** Low risk group TMB waterfall map **(C)** Violin chart of TMB difference analysis for the high and low risk group **(D)** Survival analysis diagram of high and low TMB samples (P < 0.05) **(E)** High-low risk stratified survival analysis diagram for high-low TMB samples (P < 0.05) **(F)** Violin chart for analysis of TIDE differences between high and low risk groups (P < 0.05).

The samples were divided into high- and low-risk groups according to the risk score. Analysis of the TME scores revealed significant differences in the stromal score, immune score and ESTIMATE score between the high- and low-risk groups (P < 0.05) (**Figure 10A**). We estimated the levels of 22 kinds of immune cells (naive B cells, memory B cells, memory B cells, memory T cells, naive CD8+ T cells, resting memory CD4+ T cells, activated memory CD4+ T cells, follicular helper T cells, regulatory T cells (Tregs), gamma delta T cells, resting NK cells, activated NK cells, monocytes, M0 macrophages, M1 macrophages, M2 macrophages, resting dendritic cells, activated dendritic cells, resting mast cells, activated mast cells, eosinophils and neutrophils) in each sample, and a histogram was drawn (**Figure 10B**). The high- and low-risk groups were compared to determine whether there were significant differences in the levels of these 22 kinds of immune cells. The levels of naive B cells, plasma cells, resting memory CD4 T cells, activated memory CD4 T cells, resting NK cells, activated NK cells (determined via calculation), M0 macrophages, M2 macrophages, resting mast cells, activated mast cells and eosinophils were significantly different between the high- and low-risk groups (P < 0.05) (**Figure 10C**). This further confirms that the immune cell infiltration patterns differ among different risk groups. We assessed 29 immune cells/functions (aDCs, APC coinhibition, APC costimulation, B cells, CCR, CD8+ T cells, checkpoint, cytolytic activity, DCs, HLA, iDCs, inflammation-promoting, macrophages, mast cells, MHC class I, neutrophils, NK cells, paraination, pDCs, T cell coinhibition, T cell costimulation, T helper cells, Tfhs, Th1 cells, Th2 cells, TIL, Treg, type I IFN response, and type II IFN response), and we found significant differences in the enrichment of related genes between the high- and low-risk groups for B cells, mast cells, MHC class I cells, neutrophils, pDCs, T helper cells, Th1 cells, Th2 cells, TILs, the type I IFN response, the type II IFN response, and 11 immune-related responses (**Figure 10D**). This indicates that high-risk groups may promote the infiltration of immunosuppressive cells and increase the expression of immune checkpoint genes (ICGs), leading to immune escape and poor prognosis. For the analysis of the relationship between the risk score and TMB of gastric cancer, the top 20 genes (TTN, TP53, MUC16, LRP1B, ARID1A, CSMD3, SYNE1, FAT4, FLG, PCLO, ZFHX4, ACVR2A, HMCN1, DNAH5, OBSCN, RYR2, SPTA1, FAT3, CSMD1, and KMT2D) had higher mutation rates in the low-risk group (**Figures 11A, B**). In the risk score-related samples, there were significant differences in TMB between the high- and low-risk groups (**Figure 11C**). There were significant differences in survival between patients with high and low TMB (H/L TMB) (P < 0.05) (**Figure 11D**). Stratified survival analysis was performed on H/L-TMB samples, and the samples were divided into four subgroups: H-TMB + low risk, H-TMB + high risk, L-TMB + high risk, and L-TMB + low risk. The survival rate of the high-risk group was lower than that of the low-risk group (P < 0.05) (**Figure 11E**). This further confirms the negative correlation between TMB levels and survival prognosis. Analysis of tumor immune dysfunction and exclusion (TIDE) revealed that the high-risk group had a higher TIDE score and was more prone to immune escape (P < 0.05) (**Figure 11F**).This suggests that tumors in the high-risk group may suppress immune responses through multiple mechanisms, leading to poor prognosis. In summary, the analysis revealed significant differences in TME scores and immune cell levels between high- and low-risk groups. Notably, various immune cells such as naive B cells and NK cells, and immune functions, showed significant variation. High-risk groups had higher TIDE scores, indicating a greater tendency for immune escape. Additionally, the low-risk group exhibited higher mutation rates in key genes and better survival outcomes compared to the high-risk group.

### 3.7 Prediction of effective drugs in the high- and low-risk groups

**Figure 12:**
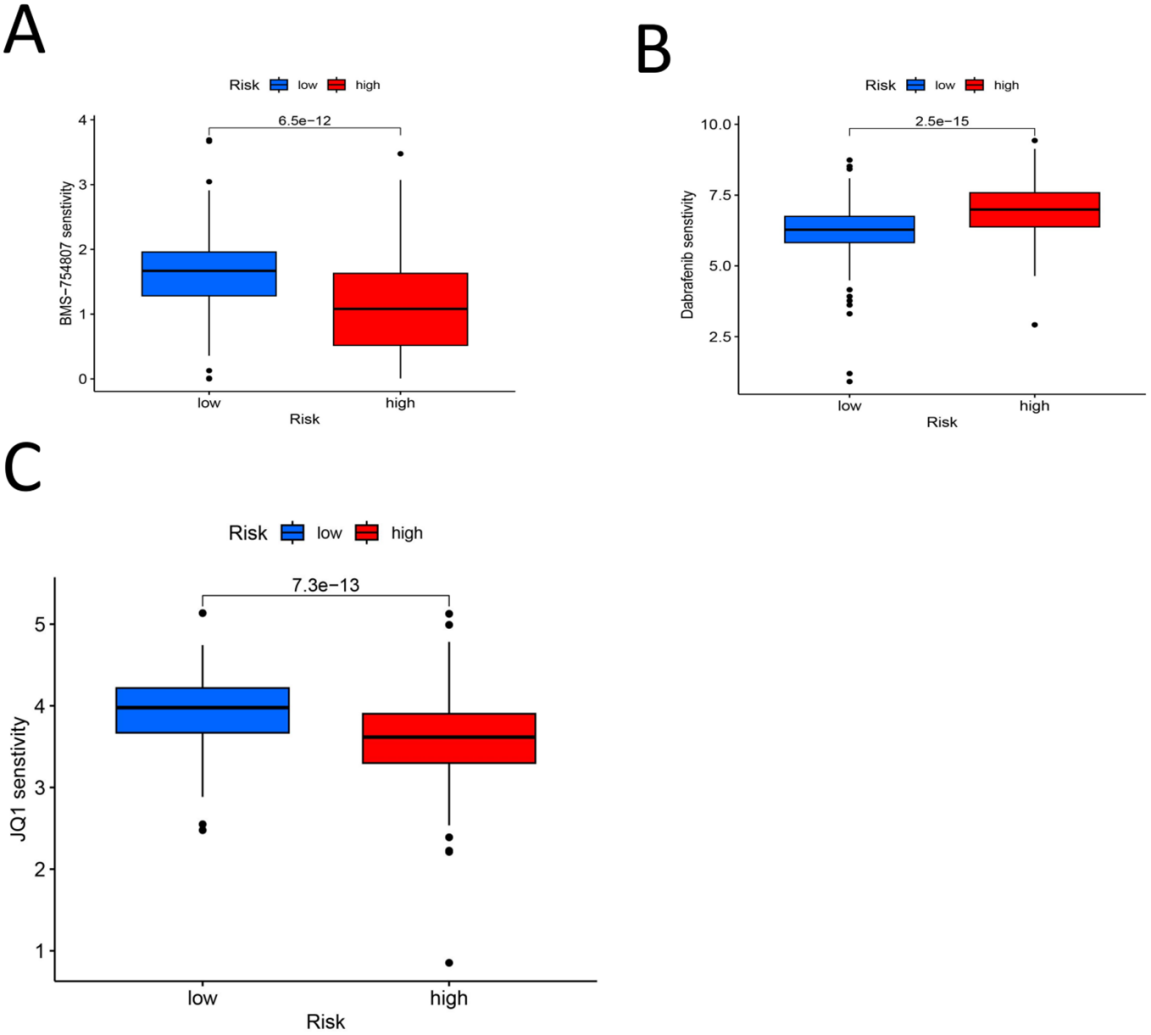
**(A)** BMS-754807 Drug PC50 analysis box diagram (P < 0.00000000001) **(B)** Drug PC50 analysis box diagram for Dabrafenib (P < 0.00000000001) **(C)** JQ1 drug PC50 analysis box diagram (P < 0.00000000001).

We set the P value of screening to pFilter = 0.00000000001 for screening effective drugs. Finally, three drugs with significantly higher sensitivity in the high-risk group were screened: BMS-754807, dabrafenib, and JQ1 (**Figures 12A-C**). As shown in **Figure 11**, BMS-754807 and JQ1 had lower scores in the high-risk group, and these two drugs were more effective in the high-risk group. However, dabrafenib scored lower in the low-risk group and was more effective in the low-risk group. These findings indicate that the model can be used at an individualized level to screen drugs likely to be effective against gastric cancer.

### 3.8 *In vitro* validation of FRMD6-AS2

**Figure 13:**
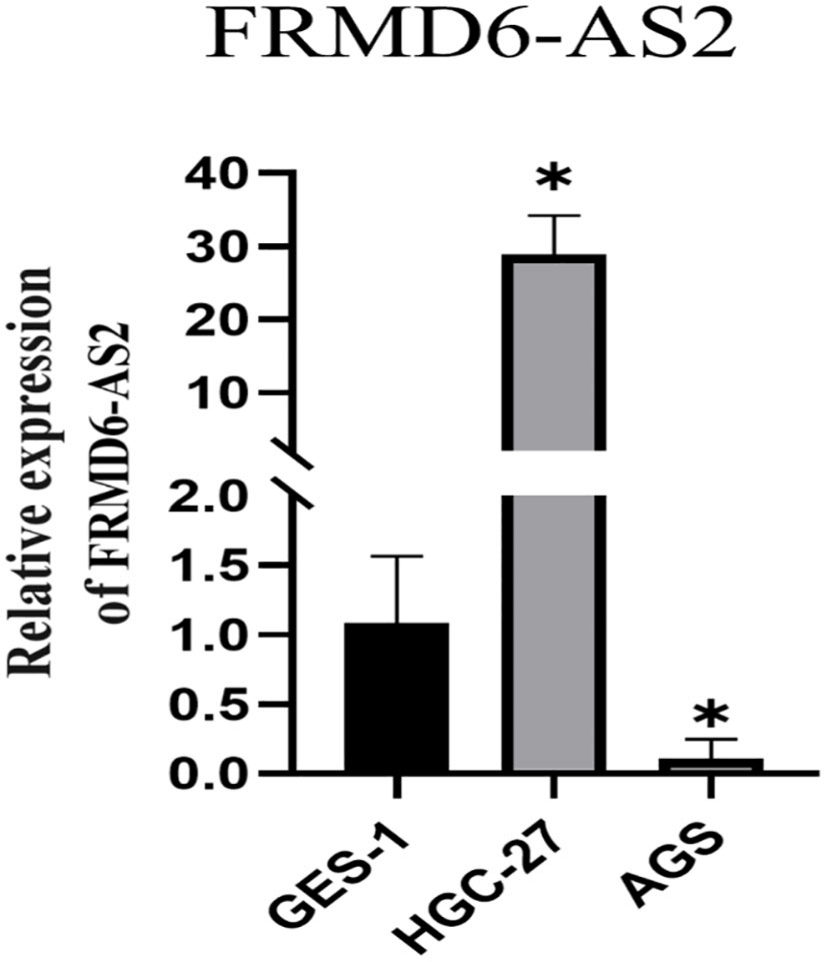
The qRT-PCR show FRMD6-AS2 was highly expressed in HGC-27 and low in AGS compared with normal GES-1 cells (*,P < 0.05).

The results revealed that FRMD6-AS2 was highly expressed in HGC-27 cells and expressed at low levels in AGS cells compared with normal GES-1 cells (**Figure 13**), but there were significant differences between the two groups (P < 0.05).

## 4 Discussion

Stomach cancer is a global health problem, with more than 1 million people newly diagnosed worldwide each year, and it remains the third leading cause of cancer-related death (53). The pathogenesis of gastric cancer is poorly understood, and there are many factors that affect gastric cancer, such as neurological (54) and genetic (55) factors. At present, gastric cancer treatment outcomes are not ideal, especially for intermediate- and advanced-stage gastric cancer. Although many therapeutic methods, such as surgery, chemotherapy, radiotherapy, and immunotherapy, are used to treat gastric cancer, the therapeutic effect on gastric cancer is still poor (56). Therefore, new prognostic signatures and new therapeutic targets are needed for gastric cancer.

The DRL gastric cancer prognostic signatures constructed in this study were composed of three lncRNAs. The screening criterion was P < 0.0005, and 100 DRL gastric cancer prognostic signatures generated by more than 100 calculations were screened. The risk score formula for the selected signature was as follows: Risk score= (0.544735914395105 * AC107021.2 expression) + (0.705013376452246 * AC129507.1 expression) + (0.433534323181848 * ‘FRMD6-AS2’ expression). Through verification, it was found that the signature performance was basically the same in the training group and the experimental group. The prognosis of the high-risk group was worse than that of the low-risk group. In addition, in the stratified analysis of 14 clinical factors, there were no significant differences in survival between the high- and low-risk groups for 3 clinical stratification factors (M1,G1-G2,T1-2), while there were significant differences in survival between high- and low-risk groups for the remaining 11 clinical stratification factors (age<60,age≥60,G3,M0,Female,Male,N0-1,N2-3,Stage I-II,Stage III-IV,T3-4). These findings further indicate that the DRL-related prognostic model of gastric cancer can provide more accurate personalized predictions for the prognosis of gastric cancer patients. These findings indicate that the prognostic signature has high accuracy.

In this study, relevant samples were screened through prognostic signatures, and differential expression analysis, GO and KEGG analysis, GSEA and other analyses were performed. It has been found that the AC129507.1/(FLNA, TLN1) signaling axis affects the prognosis of gastric cancer through the FOCAL ADHESION pathway. Meanwhile, the AC107021.2/MYH10 signaling axis influences the prognosis of gastric cancer via the TIGHT JUNCTION, VIRAL MYOCARDITIS, and REGULATION OF ACTIN CYTOSKELETON signaling pathways. Among the components of these pathways, the protein encoded by FLNA is a radioactive protein that can cross-link actin filaments and link actin filaments to membrane glycoproteins. In previous studies, AC129507.1 was found to be potentially associated with the prognosis of prostate cancer (57), but this study is the first to find a link between AC129507.1 and stomach cancer. FLNA has been found to be associated with a poorer prognosis for colon cancer (58); FLNA may be beneficial as a clinical target for gastric cancer treatment (59), and focal adhesion may be associated with the prognosis of gastric cancer. Focal adhesion kinase may be a new target for the treatment of gastric cancer (60). Focal adhesion kinase (FAK) combined with YAP/TEAD inhibition can significantly inhibit growth of gastric cancer (61). ORAI2 enhances metastasis ability of gastric cancer cells by inducing FAK-mediated MAPK/ERK activation (62). The combination of FAK inhibitors with MAPK inhibitors or CDK4/6 inhibitors may be applied in the development of gastric cancer therapies (63). We found that TLN1 may influence prognosis of gastric cancer through the PTK2-PXN-VCL-E-Cadherin-CAPN2-MAPK1 signaling pathway (64). Previous studies have shown that AC107021.2 may be associated with the prognosis of lung adenocarcinoma (65); other studies found that AC107021.2 may affect the prognosis of gastric cancer under the condition of hypoxia (66). For MYH10, It was found that combining FAK inhibitors with MAPK inhibitors or CDK4/6 inhibitors may be associated with drug resistance and prognosis in ovarian cancer (67), etc. The current study revealed for the first time that this gene may be related to the prognosis of gastric cancer. Some studies have shown that Helicobacter pylori may affect development of gastric cancer by affecting tight junction-encoding protein (68). The present study is the first to show that viral myocarditis is associated with gastric cancer, but the specific mechanism is unclear and may be related to inflammation; further research is needed. A study on the relationship between regulation of the actin cytoskeleton and gastric cancer revealed that Celastrus orbiculatus Thunb. may affect metastasis of gastric cancer by regulating the regulation of the actin cytoskeleton (69). In summary, this study revealed that two signaling axes associated with disulfidptosis may influence the prognosis of gastric cancer.

FRMD6 antisense RNA 2 (FRMD6-AS2) is a noncoding RNA, and it has been shown that it may have anticancer effects on endometrial cancer (70). Tan et al. reported that FRMD6-AS2 may participate in immune regulation in rectal cancer (71). The methylation of FRMD6-AS2 may be involved in inhibiting the growth of malignantstruma ovarii (follicular carcinoma) (72). In this study, FRMD6-AS2 was found to be highly expressed in HGC-27 cells and expressed at lower levels in AGS cells than in normal GES-1 cells. These findings indicate that FRMD6-AS2 is different in normal gastric tissues and gastric cancer tissues, but there may be differences in its expression in different gastric cancer tissues, with high expression in some gastric cancer subtypes and low expression in other gastric cancer tissues. However, further analysis of pathological tissues is needed to distinguish which subtypes of gastric cancer may have high or low expression. These findings also suggest that FRMD6-AS may be a potential target for the treatment of gastric cancer.

By analyzing the relationship between the TME and the prognosis of gastric cancer, our study revealed significant differences in TME scores between the high- and low-risk groups (P < 0.05). NK cells can kill gastric cancer cells, and this study revealed that the number of resting NK cells was greater in the low-risk gastric cancer group than in the high-risk gastric cancer group, suggesting that resting NK cells are beneficial for the prognosis of gastric cancer (73). M2 macrophages secrete chitinase 3-like protein 1 (CHI3L1), which promotes the metastasis of gastric cancer cells (74, 75). On the basis of the above analysis, we conclude that the prognostic model established in this study can accurately evaluate the efficacy of immunotherapy for gastric cancer.

Our study utilized a predictive model to identify potential drugs that may be sensitive in the treatment of gastric cancer. The drugs identified include BMS-754807, Dabrafenib, and JQ1. We found that BMS-754807 may have inhibitory effects on tumor growth (76), although its specific efficacy against gastric cancer requires further experimental validation. Dabrafenib is primarily used for the treatment of thyroid cancer and melanoma (77–79). Regarding JQ1, there is a significant body of research indicating its potential in treating gastric cancer (80, 81), as it can inhibit the growth and metastasis of gastric cancer cells. Thus, while all three drugs have demonstrated therapeutic effects against tumors, only JQ1 currently has clear evidence of efficacy in treating gastric cancer. Further research is necessary to explore the potential of BMS-754807 and Dabrafenib in the context of gastric cancer treatment.

Although this study elucidates the potential role of DRLs in gastric cancer, several limitations exist. First, the limited number of samples in the TCGA database may impact the generalizability of the model. External validation is required and should be addressed in future studies. Second, this study is primarily based on bioinformatics analysis, lacking experimental validation. Furthermore, the specific molecular mechanisms of DRLs and how they interact with disulfide death genes require further experimental research to clarify.

## 5 Conclusion

In conclusion, we established a new DRL-based prognostic signature for gastric cancer via a multiple-screen method. This signature effectively differentiates between high-risk and low-risk patients, with high-risk patients having poorer outcomes. The model’s accuracy was confirmed through various analyses, including survival and clinical factor assessments. Immune profile differences between risk groups were also noted, with the high-risk group showing greater immune escape tendencies. Two signaling axes related to disulfidptosis may be involved in the prognosis of gastric cancer, and JQ1 may be an effective drug for the treatment of gastric cancer. Moreover the noncoding RNA FRMD6-AS may be a potential target for the treatment of gastric cancer, but further experimental verification is needed. These findings offer more possibilities for personalized treatment approaches for gastric cancer in the future.

## Data Availability

The raw data supporting the conclusions of this article will be made available by the authors, without undue reservation.
