# Supplementary material for: Prognostic value of disulfidptosis-associated genes in gastric cancer: a comprehensive analysis
Source: Front Oncol. 2025 Mar 4;15:1512394. doi: 10.3389/fonc.2025.1512394 (PMC11913695; doi:10.3389/fonc.2025.1512394)
Supplement: Supplementary file 1 [file DataSheet1.pdf]

ONTOLOGY	ID	Description	pvalue	geneID
BP	GO:0003012	muscle system process	5.30E-21	CACNA1H/HTR2B/KCNE4/CHGA/SYNM/MYOCD/PLN/IGF1/KCNJ8/PTGER3/TPM1/HTR2A/CALD1/PRKG1/SCN7A/PI16/LMOD1/MYH11/CHRNA3/DES/ATP1A2/ANK2/ABCC9/PDE9A/DTNA/ACTA1/TMOD1/TACR1/CNN1/PPP1R12B/NR4A3/CASQ2/AGT/DMD/KCNA5/SMTN/FLNA/CRYAB/RGS4/SGCA/GUCY1A1/TPM2/TACR2/NKX2-5/KCNMA1/CACNB2/AKAP6/CHRM2/MYOC/SCN4B/MYL9/KCNH2/MIR145/HAND2/SGCD/GNAO1/FBXO32/MYLK/CACNA1C/SMPX
BP	GO:0006936	muscle contraction	7.95E-20	CACNA1H/HTR2B/KCNE4/CHGA/SYNM/MYOCD/PLN/KCNJ8/PTGER3/TPM1/HTR2A/CALD1/PRKG1/SCN7A/LMOD1/MYH11/CHRNA3/DES/ATP1A2/ANK2/ABCC9/DTNA/ACTA1/TMOD1/TACR1/CNN1/PPP1R12B/CASQ2/AGT/DMD/KCNA5/SMTN/FLNA/CRYAB/SGCA/GUCY1A1/TPM2/TACR2/NKX2-5/KCNMA1/CACNB2/CHRM2/SCN4B/MYL9/KCNH2/MIR145/SGCD/GNAO1/MYLK/CACNA1C/SMPX
BP	GO:0042692	muscle cell differentiation	7.94E-15	CACNA1H/CNTNAP1/PGM5/MYOCD/JAM2/IGF1/POPDC2/TPM1/ACTN2/CDON/CDH2/FLNC/BVES/SPEG/FOXF1/PI16/LMOD1/SMYD1/MYH11/RBPMS2/FGF10/ITGA8/ANK2/CFL2/ACTA1/TMOD1/DNER/PRICKLE1/NFATC4/CSRP1/ANKRD1/SYPL2/AGT/DMD/ZEB1/RGS4/RBM24/IGF2/ASB2/NKX2-5/LDB3/AKAP6/MYL9/MIR145/LAMA2/ACTC1/SGCD/GREM1
BP	GO:0030198	extracellular matrix organization	9.39E-14	COL8A1/FBLN1/ADAMTS8/DPT/CMA1/FOXF1/CCDC80/MYH11/PHLDB2/ABI3BP/HAS1/CTSG/ITGA8/ELN/SFRP2/ADAMTSL3/FKBP10/PRICKLE1/AGT/FBLN5/SMOC2/ELANE/SERPINF2/COL2A1/GPM6B/COL14A1/CYP1B1/ADAMTS16/COL4A6/TNXB/RECK/MFAP4/LAMA2/FLRT2/HMCN1/ANGPTL7/DDR2/GREM1/PTX3/VTN
BP	GO:0043062	extracellular structure organization	1.04E-13	COL8A1/FBLN1/ADAMTS8/DPT/CMA1/FOXF1/CCDC80/MYH11/PHLDB2/ABI3BP/HAS1/CTSG/ITGA8/ELN/SFRP2/ADAMTSL3/FKBP10/PRICKLE1/AGT/FBLN5/SMOC2/ELANE/SERPINF2/COL2A1/GPM6B/COL14A1/CYP1B1/ADAMTS16/COL4A6/TNXB/RECK/MFAP4/LAMA2/FLRT2/HMCN1/ANGPTL7/DDR2/GREM1/PTX3/VTN

BP	GO:0045229	external encapsulating structure organization	1.29E-13	COL8A1/FBLN1/ADAMTS8/DPT/CMA1/FOXF1/CCDC80/MYH11/PHLDB2/ABI3BP/HAS1/CTSG/ITGA8/ELN/SFRP2/ADAMTSL3/FKBP10/PRICKLE1/AGT/FBLN5/SMOC2/ELANE/SERPINF2/COL2A1/GPM6B/COL14A1/CYP1B1/ADAMTS16/COL4A6/TNXB/RECK/MFAP4/LAMA2/FLRT2/HMCN1/ANGPTL7/DDR2/GREM1/PTX3/VTN
BP	GO:0060537	muscle tissue development	5.66E-13	CNTNAP1/PGM5/MYOCD/PLN/SVIL/FGF2/ITGA7/IGF1/POPDC2/TPM1/ACTN2/CDON/BVES/ZFPM2/ALDH1A2/PI16/HLF/SMYD1/MYH11/HOXD10/ITGA8/ELN/CFL2/ACTA1/DNER/PRICKLE1/CSRP1/ANKRD1/AGT/NOG/DMD/RGS4/RBM24/IGF2/KCNK2/ASB2/JPH2/MEOX2/NKX2-5/AKAP6/MIR145/ACTC1/SGCD/TNN/MYLK/GREM1
BP	GO:0055001	muscle cell development	6.28E-13	CNTNAP1/PGM5/IGF1/TPM1/ACTN2/FLNC/BVES/PI16/LMOD1/MYH11/ANK2/CFL2/ACTA1/TMOD1/DNER/PRICKLE1/NFATC4/CSRP1/ANKRD1/SYPL2/AGT/DMD/RGS4/ASB2/NKX2-5/LDB3/AKAP6/MYL9/ACTC1/SGCD
BP	GO:0007517	muscle organ development	8.39E-13	CACNA1H/CNTNAP1/MKX/MYOCD/SVIL/ITGA7/IGF1/POPDC2/TPM1/CDON/BVES/ZFPM2/SPEG/FHL1/HLF/SMYD1/ITGB1BP2/DES/HOXD10/ELN/TAGLN/CFL2/ACTA1/DNER/ANKRD1/NOG/DMD/SMTN/CRYAB/SGCA/RBM24/IGF2/ASB2/JPH2/MEOX2/NKX2-5/MIR145/LAMA2/ACTC1/SGCD/MYLK
BP	GO:0031589	cell-substrate adhesion	1.76E-12	FREM1/FERMT2/JAM3/NPY/COL8A1/ITGA7/LIMS2/FBLN1/ACTN2/BVES/FGA/APOA1/SFRP1/FOXF1/ATP1B2/CCDC80/PHLDB2/COL26A1/ABI3BP/ITGBL1/ITGA8/SEMA3E/CCL21/FLNA/FBLN5/FGL1/FGG/GPM6B/APOD/MYOC/TNXB/STON1/TNN/EPHA3/LYVE1/MSLNL/CD36/GREM1/ITGA9/VTN/SORBS1
BP	GO:1903522	regulation of blood circulation	3.73E-12	CACNA1H/ADRA1D/KCNE4/CHGA/PLN/SLC8A2/POPDC2/TPM1/HTR2A/BVES/FGA/HSPB7/ATP1B2/DES/ATP1A2/ANK2/ABCC9/KCNH6/TACR1/CASQ2/AGT/DMD/KCNA5/FLNA/RGS4/FGG/NKX2-5/CACNB2/CHRM2/SCN4B/KCNH2/AGTR1/TRPC1/CACNA1C

BP	GO:0051146	striated muscle cell differentiation	7.31E-12	CACNA1H/CNTNAP1/PGM5/MYOCD/JAM2/IGF1/POPDC2/TPM1/ACTN2/CDON/CDH2/FLNC/BVES/PI16/LMOD1/SMYD1/MYH11/CFL2/ACTA1/TMOD1/DNER/PRICKLE1/CSRP1/ANKRD1/AGT/RGS4/RBM24/IGF2/ASB2/NKX2-5/LDB3/AKAP6/MYL9/ACTC1/SGCD/GREM1
BP	GO:0061564	axon development	3.41E-11	CNTNAP1/RELN/ARHGEF25/GLI3/ADCY1/PTN/NTN1/CDH2/NGFR/CNTN4/PRKG1/TNC/SLIT3/PLPPR4/NEXN/CNR1/GAP43/NDN/PRICKLE1/MAP1A/SEMA3E/NOG/NFASC/FLNA/DIXDC1/RND2/MAP6/GFRA3/CCKAR/NPTX1/MAP1B/PLXNA4/GPM6B/CHL1/NGF/CNTN1/APOD/BOC/FLRT2/SLIT2/CRTAC1/TNN/NRXN3/CXCL12/EPHA3/EPHA7
BP	GO:0060485	mesenchyme development	6.34E-11	DLL3/FERMT2/HTR2B/TGFB1I1/IGF1/BNC2/CDH2/ZFPM2/ACTG2/SFRP1/ALDH1A2/AMH/FOXF1/TMEM100/ACTA2/PHLDB2/FGF10/SFRP2/ACTA1/PRICKLE1/SEMA3E/FRZB/NOG/FLNA/RBM24/SIX2/NKX2-5/DACT3/SOX10/TNXB/MIR145/ACTC1/HAND2/EPHA3/TGFB3/GREM1
BP	GO:0010927	cellular component assembly involved in	8.44E-11	CNTNAP1/PGM5/PLN/TPM1/ACTN2/PLA2G3/FLNC/LMOD1/MYH11/PHLDB2/ANK2/CFL2/CCDC136/ACTA1/TMOD1/CSRP1/ANKRD1/NKX2-5/LDB3/CNTN1/MYL9/ACTC1
BP	GO:0060047	heart contraction	8.85E-11	CACNA1H/KCNE4/CHGA/PLN/SLC8A2/KCNJ8/POPDC2/TPM1/BVES/HSPB7/ATP1B2/DES/ATP1A2/ANK2/ABCC9/KCNH6/CASQ2/AGT/DMD/KCNA5/FLNA/RGS4/NKX2-5/CACNB2/CHRM2/SCN4B/KCNH2/TRPC1/ACTC1/SGCD/CACNA1C
BP	GO:0003018	vascular process in circulatory system	1.21E-10	ADRA1D/FERMT2/HTR2B/SLC8A2/KCNJ8/PDE3A/HTR2A/VSTM4/CPS1/NPR3/FGA/PRKG1/ATP1B2/SLC2A4/AKAP12/ATP1A2/LEPR/SLC38A3/ABCC9/TACR1/AGT/KCNA5/KCNMB1/GUCY1A1/FGG/SERPINF2/AZU1/TACR2/SLC7A2/AGTR1/SLIT2/CD36
BP	GO:0008016	regulation of heart contraction	2.47E-10	CACNA1H/KCNE4/CHGA/PLN/SLC8A2/POPDC2/TPM1/BVES/HSPB7/ATP1B2/DES/ATP1A2/ANK2/ABCC9/KCNH6/CASQ2/AGT/DMD/KCNA5/FLNA/RGS4/NKX2-5/CACNB2/CHRM2/SCN4B/KCNH2/TRPC1/CACNA1C
BP	GO:0003015	heart process	2.95E-10	CACNA1H/KCNE4/CHGA/PLN/SLC8A2/KCNJ8/POPDC2/TPM1/BVES/HSPB7/ATP1B2/DES/ATP1A2/ANK2/ABCC9/KCNH6/CASQ2/AGT/DMD/KCNA5/FLNA/RGS4/NKX2-5/CACNB2/CHRM2/SCN4B/KCNH2/TRPC1/ACTC1/SGCD/CACNA1C

BP	GO:0090257	regulation of muscle system process	7.92E-10	CHGA/MYOCD/PLN/IGF1/TPM1/PRKG1/PI16/CHRNA3/ATP1A2/ANK2/PDE9A/TACR1/CNN1/PPP1R12B/NR4A3/CASQ2/AGT/DMD/RGS4/SGCA/GUCY1A1/TACR2/NKX2-5/AKAP6/CHRM2/MYL9/MIR145/HAND2/FBXO32/CACNA1C
BP	GO:0007160	cell-matrix adhesion	8.06E-10	FREM1/FERMT2/JAM3/ITGA7/ACTN2/FGA/SFRP1/PHLDB2/ITGBL1/ITGA8/SEMA3E/CCL21/FBLN5/FGL1/FGG/GPM6B/APOD/MYOC/TNXB/STON1/TNN/EPHA3/LYVE1/MSLNL/CD36/GREM1/ITGA9/VTN/SORBS1
BP	GO:0019932	second-messenger-mediated signaling	1.44E-09	HTR2B/PLN/SLC8A2/IGF1/ADCY1/PDE3A/IRAG1/PRKG1/TMEM100/PDE7B/ATP1A2/RGN/ANK2/RCAN2/PDE9A/GIP/NFATC4/CASQ2/AGT/DMD/GUCY1A1/PTGFR/AZU1/SCT/AKAP6/AGTR1/SGCD/ADCYAP1/CACNA1C/CD36/CAP2/NEUROD1
BP	GO:0031032	actomyosin structure organization	1.72E-09	FERMT2/PGM5/TPM1/ACTN2/FLNC/APOA1/SFRP1/LMOD1/MYH11/PHLDB2/ELN/CFL2/ACTA1/TMOD1/TACR1/CNN1/CSRP1/ANKRD1/SERPINF2/NKX2-5/LDB3/MYOC/MYL9/ACTC1/TGFB3/SORBS1
BP	GO:0050804	modulation of chemical synaptic transmission	2.07E-09	ROR2/RELN/NPY/CACNG4/SLC8A2/ADCY1/PTN/GRIK5/HTR2A/PTPRD/CDH2/NGFR/CNTN4/ADIPOQ/SYT4/CHRNA3/NRXN2/AKAP12/ATP1A2/PLPPR4/NLGN4X/CNR1/BCHE/TACR1/GIP/MAP1A/INA/NFATC4/AGT/NOG/SCGN/NPTX2/RGS4/NPTXR/NPTX1/MAP1B/NLGN4Y/SCT/TACR2/CHRM2/LAMA2/EPHA7/CHRDL1
BP	GO:0042391	regulation of membrane potential	2.11E-09	CACNA1H/CNTNAP1/SEZ6/KCNE4/RELN/PLN/SLC8A2/KCNK3/KCNJ8/POPDC2/GRIK5/ACTN2/BVES/ATP1B2/FHL1/CHRNA3/ATP1A2/ANK2/UCN3/NLGN4X/CNR1/KCNH6/TACR1/GLRB/CASQ2/DMD/KCNA5/FLNA/RGS4/KCNK2/DCN/KCNMA1/CACNB2/AKAP6/MYOC/SCN4B/KCNH2/RGS7BP/CACNA1C/CD36
BP	GO:0099177	regulation of trans-synaptic signaling	2.21E-09	ROR2/RELN/NPY/CACNG4/SLC8A2/ADCY1/PTN/GRIK5/HTR2A/PTPRD/CDH2/NGFR/CNTN4/ADIPOQ/SYT4/CHRNA3/NRXN2/AKAP12/ATP1A2/PLPPR4/NLGN4X/CNR1/BCHE/TACR1/GIP/MAP1A/INA/NFATC4/AGT/NOG/SCGN/NPTX2/RGS4/NPTXR/NPTX1/MAP1B/NLGN4Y/SCT/TACR2/CHRM2/LAMA2/EPHA7/CHRDL1

BP	GO:0061337	cardiac conduction	3.13E-09	KCNE4/PLN/SLC8A2/ATP1B2/ATP1A2/ANK2/ABCC9/KCNH6/CASQ2/AGT/KCNA5/FLNA/NKX2-5/CACNB2/SCN4B/KCNH2/TRPC1/CACNA1C
BP	GO:0030239	myofibril assembly	4.63E-09	PGM5/TPM1/ACTN2/FLNC/LMOD1/MYH11/CFL2/ACTA1/TMOD1/CSRP1/ANKRD1/NKX2-5/LDB3/MYL9/ACTC1
BP	GO:0007409	axonogenesis	4.65E-09	CNTNAP1/RELN/ARHGEF25/GLI3/ADCY1/NTN1/CDH2/NGFR/CNTN4/PRKG1/SLIT3/PLPPR4/NEXN/GAP43/NDN/PRICKLE1/MAP1A/SEMA3E/NOG/NFASC/DIXDC1/RND2/MAP6/GFRA3/CCKAR/NPTX1/MAP1B/PLXNA4/CHL1/NGF/CNTN1/BOC/FLRT2/SLIT2/TNN/NRXN3/CXCL12/EPHA3/EPHA7
BP	GO:0055002	striated muscle cell development	5.70E-09	PGM5/TPM1/ACTN2/FLNC/LMOD1/MYH11/CFL2/ACTA1/TMOD1/CSRP1/ANKRD1/NKX2-5/LDB3/MYL9/ACTC1
BP	GO:0010959	regulation of metal ion transport	1.16E-08	KCNE4/PLN/KCNK3/HTR2A/ACTN2/ATP1B2/FHL1/FXYD6/ATP1A2/RGN/ANK2/PRKD1/ABCC9/VIP/CASQ2/AGT/DMD/GRP/KCNA5/FLNA/KCNMB1/RGS4/TF/JPH2/NPSR1/NKX2-5/CACNB2/F2/AKAP6/CNTN1/GCG/SCN4B/KCNH2/TRPC1/MYLK/CXCL12/HSPA2/CACNA1C
BP	GO:0001503	ossification	1.69E-08	ROR2/FERMT2/PTH1R/MN1/GLI3/FGF2/IGF1/PTN/ASPN/SFRP1/TNC/GPC3/PRKD1/CHRDL2/AHSG/SFRP2/PRICKLE1/TACR1/NOG/ZBTB16/IGF2/TWIST2/COL2A1/SIX2/GPM6B/MYOC/RSPO2/BMP3/HAND2/CYP24A1/TNN/MGP/CHRD/GDF10/PENK/DDR2/GREM1/CHRDL1
BP	GO:0006939	smooth muscle contraction	1.98E-08	HTR2B/MYOCD/PTGER3/HTR2A/PRKG1/MYH11/CHRNA3/ATP1A2/TACR1/CNN1/AGT/SMTN/GUCY1A1/TACR2/KCNMA1/CHRM2/MIR145/MYLK
BP	GO:0007611	learning or memory	2.21E-08	RELN/GRPR/SLC8A2/ADCY1/PTN/HTR2A/SLC2A4/SYT4/NRXN2/ITGA8/ATP1A2/LRRN4/NLGN4X/CNR1/BCHE/TACR1/GIP/MAP1A/NFATC4/VIP/AGT/NOG/NPTX2/KCNK2/NLGN4Y/TACR2/NGF/NRXN3/TUBA1A
BP	GO:0034764	positive regulation of transmembrane transport	4.16E-08	RELN/CACNG4/KCNK3/IGF1/ACTN2/ADIPOQ/CLIP3/ATP1B2/RGN/ANK2/GPC3/GIP/NR4A3/AGT/DMD/FLNA/KCNMB1/JPH2/NPSR1/CACNB2/F2/AKAP6/KCNH2/TRPC1/HSPA2/SORBS1
BP	GO:0055123	digestive system development	4.54E-08	BARX1/MYOCD/NPY/GLI3/CPS1/SFRP1/ALDH1A2/FOXF1/RBPMS2/FGF10/NKX3-2/CLMP/SFRP2/GIP/IGF2/SCT/SIX2/SOX10/ASCL1/TGFB3

BP	GO:0042063	gliogenesis	4.80E-08	ROR2/CNTNAP1/RELN/GLI3/FGF2/TRPC4/PTN/NTN1/CDH2/ATP1B2/FGF10/LEPR/GAP43/NDN/DNER/LGI4/NOG/AZU1/WASF3/EMX1/F2/GPM6B/CNTN1/SOX10/MYOC/DAAM2/ASCL1/TUBA1A/PENK/IL33/VTN
BP	GO:0006874	cellular calcium ion homeostasis	4.87E-08	HTR2B/PTH1R/NPY/PLN/SLC8A2/FGF2/TRPC4/PLCH2/HTR2A/SV2A/ATP1A2/RGN/ANK2/PRKD1/CNR1/CCL21/CASQ2/SYPL2/DMD/SCGN/FLNA/JPH2/ELANE/NPSR1/CACNB2/F2/AKAP6/TRPC1/CXCL12/CACNA1C
BP	GO:0048660	regulation of smooth muscle cell proliferation	5.25E-08	OGN/MYOCD/FGF2/IGF1/TPM1/NPR3/ADIPOQ/PRKG1/RBPMS2/ELN/TACR1/CNN1/NR4A3/VIP/AGT/ELANE/SERPINF2/APOD/MIR145/VIPR2/TGFB3/DDR2
BP	GO:0085029	extracellular matrix assembly	5.84E-08	MYH11/PHLDB2/HAS1/ELN/FKBP10/PRICKLE1/AGT/FBLN5/GPM6B/TNXB/MFAP4
BP	GO:0043266	regulation of potassium ion transport	5.87E-08	KCNE4/KCNK3/HTR2A/ACTN2/ATP1B2/FHL1/ANK2/ABCC9/VIP/CASQ2/GRP/KCNA5/FLNA/KCNMB1/RGS4/AKAP6/KCNH2
BP	GO:0086009	membrane repolarization	6.78E-08	KCNE4/KCNJ8/ATP1B2/ATP1A2/ANK2/KCNH6/CASQ2/KCNA5/FLNA/AKAP6/SCN4B/KCNH2
BP	GO:0055074	calcium ion homeostasis	7.87E-08	HTR2B/PTH1R/NPY/PLN/SLC8A2/FGF2/TRPC4/PLCH2/HTR2A/SV2A/ATP1A2/RGN/ANK2/PRKD1/CNR1/CCL21/CASQ2/SYPL2/DMD/SCGN/FLNA/JPH2/ELANE/NPSR1/CACNB2/F2/AKAP6/TRPC1/SGCD/CXCL12/CACNA1C
BP	GO:0048659	smooth muscle cell proliferation	7.88E-08	OGN/MYOCD/FGF2/IGF1/TPM1/NPR3/ADIPOQ/PRKG1/RBPMS2/ELN/TACR1/CNN1/NR4A3/VIP/AGT/ELANE/SERPINF2/APOD/MIR145/VIPR2/TGFB3/DDR2
BP	GO:0016055	Wnt signaling pathway	1.12E-07	ROR2/FERMT2/BARX1/CPE/TGFB1I1/GLI3/IGFBP1/FGF2/SHISA3/BICC1/CDH2/SFRP1/FGF10/RSPO3/GPC3/PYGO1/SFRP2/PRICKLE1/NFATC4/FRZB/NOG/DIXDC1/NKX2-5/SFRP4/WNT9A/DACT3/SOX10/MYOC/RSPO2/MIR145/RECK/RBMS3/RSPO1/DAAM2/TNN/PRKAA2/PRICKLE2/GREM1
BP	GO:0046883	regulation of hormone secretion	1.14E-07	CHGA/SSTR5/NNAT/PLA2G3/ADIPOQ/FGA/SFRP1/CARTPT/SCG5/UCN3/CNR1/TACR1/GIP/AGT/GRP/KCNA5/CCKAR/FGG/ADCY5/SCT/TACR2/F2/GCG/AGTR1/ADCYAP1/NEUROD1
BP	GO:0007200	phospholipase C-activating G protein-coupled	1.16E-07	ADRA1D/HTR2B/CHGA/PTH1R/GRPR/PTGER3/HTR2A/ACTN2/NPR3/TACR1/VIP/AGT/GRP/CCKAR/F2/CHRM2/AGTR1

BP	GO:0043270	positive regulation of ion transport	1.17E-07	RELN/CACNG4/KCNK3/ACTN2/ATP1B2/FHL1/RGN/ANK2/DMD/FLNA/KCNMB1/JPH2/NPSR1/NKX2-5/CACNB2/F2/AKAP6/CNTN1/GCG/SCN4B/KCNH2/TRPC1/MYLK/CXCL12/HSPA2
BP	GO:0032970	regulation of actin filament-based process	1.18E-07	FERMT2/JAM3/PLN/SVIL/SYNPO2/KANK4/TPM1/ACTN2/APOA1/SFRP1/ACTA2/LMOD1/PHLDB2/ATP1A2/ANK2/ELN/CFL2/TMOD1/TACR1/SEMA3E/CCL21/FLNA/RGS4/DIXDC1/SERPINF2/WASF3/GPM6B/MYOC/SLIT2/DAAM2/CXCL12/EPHA3/CACNA1C/TGFB3
BP	GO:0198738	cell-cell signaling by wnt	1.25E-07	ROR2/FERMT2/BARX1/CPE/TGFB1I1/GLI3/IGFBP1/FGF2/SHISA3/BICC1/CDH2/SFRP1/FGF10/RSPO3/GPC3/PYGO1/SFRP2/PRICKLE1/NFATC4/FRZB/NOG/DIXDC1/NKX2-5/SFRP4/WNT9A/DACT3/SOX10/MYOC/RSPO2/MIR145/RECK/RBMS3/RSPO1/DAAM2/TNN/PRKAA2/PRICKLE2/GREM1
BP	GO:0050890	cognition	1.33E-07	RELN/GRPR/SLC8A2/ADCY1/PTN/HTR2A/SLC2A4/SYT4/NRXN2/ITGA8/ATP1A2/LRRN4/NLGN4X/CNR1/BCHE/TACR1/GIP/MAP1A/NFATC4/VIP/AGT/NOG/NPTX2/KCNK2/NLGN4Y/TACR2/CHL1/NGF/NRXN3/TUBA1A
BP	GO:0033002	muscle cell proliferation	1.34E-07	OGN/MYOCD/FGF2/IGF1/TPM1/NPR3/ZFPM2/ADIPOQ/PRKG1/RBPMS2/ELN/TACR1/CNN1/NR4A3/VIP/AGT/NOG/KCNK2/ELANE/SERPINF2/NKX2-5/APOD/MIR145/VIPR2/TGFB3/DDR2
BP	GO:0030534	adult behavior	1.61E-07	SEZ6/NPY/HTR2A/CHRNA3/CARTPT/NRXN2/HOXD10/ATP1A2/RNF180/NLGN4X/TMOD1/GIP/GLRB/LGI4/CHL1/DMBX1/NRXN3/CXCL12/TUBA1A
BP	GO:0010001	glial cell differentiation	1.63E-07	ROR2/CNTNAP1/RELN/GLI3/FGF2/TRPC4/PTN/CDH2/FGF10/GAP43/DNER/LGI4/NOG/WASF3/EMX1/F2/GPM6B/CNTN1/SOX10/MYOC/DAAM2/ASCL1/TUBA1A/IL33/VTN
BP	GO:0001763	morphogenesis of a branching structure	1.63E-07	TMEM59L/GLI3/FGF2/SFRP1/FOXF1/FGF10/RSPO3/FGF7/GPC3/SFRP2/AR/SEMA3E/NFATC4/AGT/NOG/SIX2/GREB1L/SOX10/ADAMTS16/RSPO2/SLIT2/EPHA7/GREM1
BP	GO:0061138	morphogenesis of a branching epithelium	1.71E-07	TMEM59L/GLI3/FGF2/SFRP1/FOXF1/FGF10/RSPO3/FGF7/GPC3/SFRP2/AR/SEMA3E/NFATC4/AGT/NOG/SIX2/GREB1L/SOX10/ADAMTS16/RSPO2/SLIT2/GREM1

BP	GO:0051209	release of sequestered calcium ion into	1.81E-07	HTR2B/PLN/FGF2/PLCH2/HTR2A/ATP1A2/ANK2/PRKD1/CCL21/CASQ2/DMD/FLNA/JPH2/NPSR1/F2/AKAP6/TRPC1/CACNA1C
BP	GO:0046879	hormone secretion	1.94E-07	CHGA/SSTR5/CPE/NNAT/PLA2G3/ADIPOQ/FGA/SFRP1/CARTPT/SCG5/UCN3/CNR1/TACR1/GIP/VIP/AGT/GRP/KCNA5/CCKAR/FGG/ADCY5/SCT/TACR2/F2/GCG/AGTR1/ADCYAP1/PTPRN/NEUROD1
BP	GO:0010975	regulation of neuron projection development	1.96E-07	ROR2/SEZ6/RELN/ALKAL1/PTN/RGMA/PTPRD/NTN1/PLA2G3/CDH2/NEGR1/DPYSL3/SFRP1/ATP1B2/CHRNA3/PRKD1/CNR1/SFRP2/SEMA3E/NFATC4/AGT/FLNA/DIXDC1/RND2/MAP6/DGKG/MAP1B/STMN2/PLXNA4/NGF/CNTN1/SLIT2/TNN/CXCL12/EPHA3/DDR2/EPHA7
BP	GO:0070252	actin-mediated cell contraction	1.99E-07	KCNE4/PLN/KCNJ8/TPM1/ACTA2/ATP1A2/ANK2/ABCC9/KCNA5/FLNA/CACNB2/SCN4B/KCNH2/ACTC1/SGCD/CACNA1C
BP	GO:0070371	ERK1 and ERK2 cascade	2.02E-07	FERMT2/HTR2B/ALKAL1/NPY/FGF2/IGF1/FBLN1/TNFAIP8L3/HTR2A/LMO3/ADIPOQ/FGA/ACTA2/DUSP26/FGF10/AKAP12/PLA2G5/CCL21/AGT/TF/FGG/NPSR1/SERPINF2/GCG/THPO/MIR145/HAND2/ADCYAP1/CD36/DDR2/EPHA7
BP	GO:0051283	negative regulation of sequestering of	2.04E-07	HTR2B/PLN/FGF2/PLCH2/HTR2A/ATP1A2/ANK2/PRKD1/CCL21/CASQ2/DMD/FLNA/JPH2/NPSR1/F2/AKAP6/TRPC1/CACNA1C
BP	GO:0010810	regulation of cell-substrate adhesion	2.13E-07	FERMT2/NPY/COL8A1/LIMS2/FBLN1/FGA/APOA1/SFRP1/FOXF1/CCDC80/PHLDB2/COL26A1/ABI3BP/SEMA3E/CCL21/FLNA/FGG/GPM6B/APOD/MYOC/EPHA3/CD36/GREM1/VTN
BP	GO:0051282	regulation of sequestering of calcium ion	2.57E-07	HTR2B/PLN/FGF2/PLCH2/HTR2A/ATP1A2/ANK2/PRKD1/CCL21/CASQ2/DMD/FLNA/JPH2/NPSR1/F2/AKAP6/TRPC1/CACNA1C
BP	GO:0002027	regulation of heart rate	2.61E-07	KCNE4/PLN/POPDC2/TPM1/BVES/ANK2/KCNH6/CASQ2/AGT/DMD/KCNA5/RGS4/CACNB2/SCN4B/KCNH2/CACNA1C
BP	GO:0034368	protein-lipid complex	2.65E-07	PLA2G3/APOA1/APOC3/APOA2/PLA2G5/APOB/AGT/MPO/AGTR1
BP	GO:0034369	plasma lipoprotein	2.65E-07	PLA2G3/APOA1/APOC3/APOA2/PLA2G5/APOB/AGT/MPO/AGTR1
BP	GO:0060538	skeletal muscle organ development	2.84E-07	CNTNAP1/MYOCD/SVIL/ITGA7/POPDC2/CDON/BVES/HLF/SMYD1/DES/HOXD10/ELN/CFL2/ACTA1/DNER/ANKRD1/DMD/RBM24/IGF2/ASB2/MEOX2
BP	GO:0034374	low-density lipoprotein	3.00E-07	PLA2G3/APOA2/PLA2G5/APOB/AGT/MPO/AGTR1

BP	GO:0060828	regulation of canonical Wnt signaling pathway	3.14E-07	GLI3/IGFBP1/FGF2/SHISA3/BICC1/CDH2/SFRP1/FGF10/RSPO3/GPC3/SFRP2/PRICKLE1/FRZB/NOG/NKX2-5/SFRP4/DACT3/SOX10/RSPO2/MIR145/RECK/RBMS3/RSPO1/DAAM2/TNN/GREM1
BP	GO:0060070	canonical Wnt signaling pathway	3.36E-07	GLI3/IGFBP1/FGF2/SHISA3/BICC1/CDH2/SFRP1/FGF10/RSPO3/GPC3/PYGO1/SFRP2/PRICKLE1/FRZB/NOG/DIXDC1/NKX2-5/SFRP4/WNT9A/DACT3/SOX10/RSPO2/MIR145/RECK/RBMS3/RSPO1/DAAM2/TNN/GREM1
BP	GO:0007519	skeletal muscle tissue development	3.50E-07	CNTNAP1/MYOCD/SVIL/ITGA7/POPDC2/CDON/BVES/HLF/SMYD1/HOXD10/ELN/CFL2/ACTA1/DNER/ANKRD1/DMD/RBM24/IGF2/ASB2/MEOX2
BP	GO:0034329	cell junction assembly	3.63E-07	CNTNAP1/FERMT2/CDH19/JAM3/PCDHB4/PTPRD/ACTN2/PCDHB5/NTN1/CDH2/NEGR1/SFRP1/PHLDB2/LGI2/MPDZ/NRXN2/ANK2/NLGN4X/GAP43/DNER/CLDN6/AGT/NPTX1/MAP1B/NLGN4Y/GPM6B/APOD/MYOC/STON1/FLRT2/CLDN11/EPHA3/SDK2/EPHA7/GREM1/SORBS1
BP	GO:0009914	hormone transport	3.84E-07	CHGA/SSTR5/CPE/NNAT/PLA2G3/ADIPOQ/FGA/SFRP1/CARTPT/SCG5/UCN3/CNR1/TACR1/GIP/VIP/AGT/GRP/KCNA5/CCKAR/FGG/ADCY5/SCT/TACR2/F2/GCG/AGTR1/ADCYAP1/PTPRN/NEUROD1
BP	GO:0070372	regulation of ERK1 and ERK2 cascade	3.84E-07	FERMT2/HTR2B/ALKAL1/NPY/FGF2/IGF1/FBLN1/TNFAIP8L3/HTR2A/LMO3/ADIPOQ/FGA/ACTA2/DUSP26/FGF10/AKAP12/PLA2G5/CCL21/FGG/NPSR1/SERPINF2/GCG/THPO/MIR145/HAND2/ADCYAP1/CD36/DDR2/EPHA7
BP	GO:0051208	sequestering of calcium ion	4.04E-07	HTR2B/PLN/FGF2/PLCH2/HTR2A/ATP1A2/ANK2/PRKD1/CCL21/CASQ2/DMD/FLNA/JPH2/NPSR1/F2/AKAP6/TRPC1/CACNA1C
BP	GO:0006941	striated muscle contraction	4.13E-07	KCNE4/CHGA/SYNM/PLN/KCNJ8/TPM1/ATP1A2/ANK2/ABCC9/DTNA/CASQ2/DMD/KCNA5/FLNA/NKX2-5/CACNB2/SCN4B/KCNH2/SGCD/CACNA1C/SMPX
BP	GO:0060048	cardiac muscle contraction	4.50E-07	KCNE4/CHGA/PLN/KCNJ8/TPM1/ATP1A2/ANK2/ABCC9/CASQ2/DMD/KCNA5/FLNA/NKX2-5/CACNB2/SCN4B/KCNH2/SGCD/CACNA1C
BP	GO:0007613	memory	4.58E-07	RELN/SLC8A2/ADCY1/PTN/HTR2A/SLC2A4/SYT4/ITGA8/LRRN4/CNR1/TACR1/GIP/MAP1A/NFATC4/KCNK2/NGF/TUBA1A

BP	GO:0019935	cyclic-nucleotide-mediated	4.88E-07	HTR2B/ADCY1/PDE3A/IRAG1/PRKG1/PDE7B/PDE9A/GIP/GUCY1A1/SCT/AKAP6/ADCYAP1/CD36/CAP2
BP	GO:0034367	protein-containing	4.90E-07	PLA2G3/APOA1/APOC3/APOA2/PLA2G5/APOB/AGT/MPO/AGTR1
BP	GO:0090090	negative regulation of canonical Wnt signaling pathway	5.02E-07	GLI3/IGFBP1/SHISA3/BICC1/CDH2/SFRP1/GPC3/SFRP2/PRICKLE1/FRZB/NOG/NKX2-5/SFRP4/DACT3/SOX10/RBMS3/TNN/GREM1
BP	GO:0010811	positive regulation of cell-substrate	5.14E-07	FERMT2/NPY/COL8A1/LIMS2/FGA/APOA1/SFRP1/FOXF1/CCDC80/COL26A1/ABI3BP/CCL21/FLNA/FGG/MYOC/CD36/VTN
BP	GO:0097006	regulation of plasma lipoprotein	6.55E-07	CES3/PLA2G3/ADIPOQ/APOA1/GPIHBP1/APOC3/APOA2/PLA2G5/APOB/AGT/MPO/SOAT2/AGTR1/CD36
BP	GO:0016049	cell growth	6.73E-07	PAPPA2/MYOCD/PTCH2/IGFBP1/IGF1/NTN1/PRKG1/SFRP1/PI16/FHL1/SYT4/RERG/TNC/SLIT3/SFRP2/NDN/PRICKLE1/SEMA3E/FRZB/AGT/CRYAB/RGS4/RND2/FBLN5/MAP1B/EMX1/PLXNA4/F2/MEG3/AKAP6/NGF/DACT3/AGTR1/SLIT2/TNN/CXCL12/EPHA7/GREM1
BP	GO:0001508	action potential	6.92E-07	CNTNAP1/KCNE4/SLC8A2/KCNJ8/ATP1A2/ANK2/CNR1/KCNH6/TACR1/DMD/KCNA5/FLNA/CACNB2/AKAP6/SCN4B/KCNH2/CACNA1C/CD36
BP	GO:0006937	regulation of muscle contraction	7.54E-07	CHGA/MYOCD/PLN/TPM1/PRKG1/CHRNA3/ATP1A2/ANK2/TACR1/CNN1/PPP1R12B/CASQ2/DMD/GUCY1A1/TACR2/NKX2-5/CHRM2/MYL9/MIR145/CACNA1C
BP	GO:0086003	cardiac muscle cell contraction	7.98E-07	KCNE4/PLN/KCNJ8/ATP1A2/ANK2/ABCC9/KCNA5/FLNA/CACNB2/SCN4B/KCNH2/SGCD/CACNA1C
BP	GO:2000027	regulation of animal organ morphogenesis	8.09E-07	ROR2/FGF2/NGFR/SFRP1/FGF10/RSPO3/FGF7/GPC3/SFRP2/AR/PRICKLE1/AGT/NOG/SIX2/RSPO2/PRICKLE2/GREM1
BP	GO:0070374	positive regulation of ERK1 and ERK2 cascade	8.11E-07	FERMT2/HTR2B/ALKAL1/NPY/FGF2/IGF1/TNFAIP8L3/HTR2A/FGA/ACTA2/FGF10/AKAP12/PLA2G5/CCL21/FGG/NPSR1/SERPINF2/GCG/THPO/HAND2/ADCYAP1/CD36/DDR2
BP	GO:0030178	negative regulation of Wnt signaling pathway	8.27E-07	BARX1/GLI3/IGFBP1/SHISA3/BICC1/CDH2/SFRP1/GPC3/SFRP2/PRICKLE1/NFATC4/FRZB/NOG/NKX2-5/SFRP4/DACT3/SOX10/RBMS3/TNN/GREM1

BP	GO:0023061	signal release	9.95E-07	CHGA/NPY/SSTR5/CPE/NNAT/GRIK5/HTR2A/PLA2G3/ADIPOQ/SV2A/FGA/SFRP1/SYT4/CHRNA3/CARTPT/NRXN2/SCG5/UCN3/CNR1/TACR1/GIP/VIP/AGT/GRP/KCNA5/CCKAR/FGG/ADCY5/SCT/TACR2/F2/GCG/AGTR1/ADCYAP1/CXCL12/PTPRN/NEUROD1
BP	GO:0014706	striated muscle tissue development	1.02E-06	PGM5/MYOCD/PLN/FGF2/IGF1/TPM1/ACTN2/BVES/ZFPM2/ALDH1A2/PI16/MYH11/PRICKLE1/ANKRD1/AGT/NOG/RGS4/KCNK2/ASB2/JPH2/NKX2-5/AKAP6/ACTC1/SGCD/GREM1
BP	GO:0007411	axon guidance	1.03E-06	RELN/ARHGEF25/GLI3/NTN1/NGFR/CNTN4/SLIT3/NEXN/GAP43/SEMA3E/NOG/NFASC/GFRA3/PLXNA4/CHL1/CNTN1/BOC/FLRT2/SLIT2/NRXN3/CXCL12/EPHA3/EPHA7
BP	GO:0097485	neuron projection guidance	1.03E-06	RELN/ARHGEF25/GLI3/NTN1/NGFR/CNTN4/SLIT3/NEXN/GAP43/SEMA3E/NOG/NFASC/GFRA3/PLXNA4/CHL1/CNTN1/BOC/FLRT2/SLIT2/NRXN3/CXCL12/EPHA3/EPHA7
BP	GO:0048738	cardiac muscle tissue development	1.12E-06	MYOCD/PLN/FGF2/IGF1/TPM1/ACTN2/BVES/ZFPM2/ALDH1A2/PI16/MYH11/PRICKLE1/ANKRD1/AGT/NOG/RGS4/KCNK2/ASB2/JPH2/NKX2-5/AKAP6/ACTC1/SGCD/GREM1
BP	GO:0050808	synapse organization	1.15E-06	ROR2/CNTNAP1/SEZ6/RELN/PCDHB4/SLC8A2/PTPRD/PCDHB5/NTN1/CDH2/NEGR1/LGI2/NRXN2/TNC/NLGN4X/GAP43/DNER/SEMA3E/INA/NFATC4/GLRB/FLNA/NPTX1/MAP1B/NLGN4Y/WASF3/CACNB2/PLXNA4/SPARCL1/FLRT2/PDZRN3/SDK2/TUBA1A/CAP2/EPHA7/CHRDL1
BP	GO:0031346	positive regulation of cell projection organization	1.31E-06	ROR2/RELN/ALKAL1/PTN/PTPRD/NTN1/PLA2G3/NEGR1/DPYSL3/ATP1B2/DZIP1/PRKD1/CNR1/CCL21/AGT/FLNA/DIXDC1/RND2/MAP6/MAP1B/STMN2/PLXNA4/NGF/CNTN1/SLIT2/TNN/CXCL12/EPHA3/TGFB3/DDR2
BP	GO:0048565	digestive tract development	1.39E-06	MYOCD/NPY/GLI3/CPS1/SFRP1/ALDH1A2/FOXF1/RBPMS2/FGF10/NKX3-2/CLMP/SFRP2/SCT/SIX2/SOX10/ASCL1/TGFB3
BP	GO:0035265	organ growth	1.42E-06	FGF2/IGF1/BNC2/ZFPM2/PI16/FGF10/LEPR/FGF7/NLGN4X/AR/AGT/NOG/RGS4/MAEL/IGF2/KCNK2/NKX2-5/AKAP6/RSPO2/DDR2
BP	GO:0071827	plasma lipoprotein	1.54E-06	PLA2G3/APOA1/APOC3/APOA2/PLA2G5/APOB/AGT/MPO/SOAT2/AGTR1

BP	GO:0090066	regulation of anatomical structure size	1.54E-06	ADRA1D/HTR2B/SVIL/KANK4/HTR2A/ACTN2/NTN1/VSTM4/CPS1/NPR3/FGA/PRKG1/LMOD1/ATP1A2/ELN/CFL2/TMOD1/TACR1/SEMA3E/CCL21/AGT/KCNA5/KCNMB1/RND2/GUCY1A1/FGG/SERPINF2/MAP1B/SCT/KCNMA1/PLXNA4/NGF/AGTR1/SLIT2/DAAM2/CXCL12/EPHA7
BP	GO:0015837	amine transport	1.62E-06	CHGA/HTR2A/SV2A/SYT4/CHRNA3/CARTPT/ATP1A2/SLC38A3/CNR1/VIP/AGT/RGS4/TACR2/AQP8/CXCL12
BP	GO:0030111	regulation of Wnt signaling pathway	1.63E-06	BARX1/GLI3/IGFBP1/FGF2/SHISA3/BICC1/CDH2/SFRP1/FGF10/RSPO3/GPC3/SFRP2/PRICKLE1/NFATC4/FRZB/NOG/DIXDC1/NKX2-5/SFRP4/DACT3/SOX10/RSPO2/MIR145/RECK/RBMS3/RSPO1/DAAM2/TNN/GREM1
BP	GO:1902903	regulation of supramolecular fiber organization	1.67E-06	FERMT2/TUBB4A/SVIL/SYNPO2/KANK4/TPM1/ACTN2/APOA1/SFRP1/CLIP3/LMOD1/PHLDB2/ELN/CFL2/TMOD1/TACR1/MAP1A/CCL21/FLNA/CRYAB/RGS4/SERPINF2/MAP1B/WASF3/STMN2/MYOC/TNXB/SLIT2/DAAM2/CXCL12/TGFB3
BP	GO:0072001	renal system development	1.68E-06	TMEM59L/MYOCD/GLI3/FGF2/KCNJ8/BICC1/GFRA1/ADIPOQ/SFRP1/ALDH1A2/FOXF1/ACTA2/FGF10/ITGA8/RGN/GPC3/PYGO1/PRICKLE1/AGT/NOG/ZBTB16/SIX2/GREB1L/ADAMTS16/AGTR1/SLIT2/EPHA7/GREM1
BP	GO:1901379	regulation of potassium ion transmembrane	1.72E-06	KCNE4/KCNK3/ACTN2/ATP1B2/FHL1/ANK2/ABCC9/CASQ2/GRP/FLNA/KCNMB1/RGS4/AKAP6/KCNH2
BP	GO:0007218	neuropeptide signaling pathway	2.06E-06	NPY/SSTR5/CPE/GRPR/TAC1/CARTPT/SCG5/NXPH4/GLRB/TAC3/GRP/NPSR1/ADCYAP1/PENK/NXPH3
BP	GO:0034765	regulation of ion transmembrane transport	2.07E-06	CACNA1H/KCNE4/RELN/PLN/CACNG4/KCNK3/KCNJ8/ACTN2/SCN7A/ATP1B2/FHL1/FXYD6/ATP1A2/RGN/ANK2/PRKD1/ABCC9/KCNH6/CASQ2/DMD/GRP/KCNA5/FLNA/KCNMB1/RGS4/JPH2/NPSR1/KCNMA1/CACNB2/F2/AKAP6/SCN4B/KCNH2/TRPC1/HSPA2/CACNA1C
BP	GO:0097553	calcium ion transmembrane import into cytosol	2.12E-06	CACNA1H/HTR2B/PLN/SLC8A2/FGF2/PLCH2/HTR2A/SCN7A/ATP1A2/ANK2/PRKD1/CCL21/CASQ2/DMD/FLNA/JPH2/NPSR1/F2/AKAP6/TRPC1/CACNA1C
BP	GO:0001558	regulation of cell growth	2.23E-06	PAPPA2/MYOCD/PTCH2/IGFBP1/IGF1/NTN1/SFRP1/PI16/FHL1/SYT4/RERG/TNC/SLIT3/SFRP2/SEMA3E/FRZB/AGT/CRYAB/RGS4/RND2/FBLN5/MAP1B/PLXNA4/F2/MEG3/AKAP6/NGF/DACT3/AGTR1/SLIT2/CXCL12/EPHA7/GREM1

BP	GO:0060306	regulation of membrane	2.40E-06	KCNE4/ANK2/KCNH6/CASQ2/KCNA5/FLNA/AKAP6/SCN4B/KCNH2
BP	GO:1902074	response to salt	2.45E-06	CACNA1H/CACNG4/GHR/ADCY1/HTR2A/ADIPOQ/FGA/ASPN/FABP4/SYT4/CHRNA3/CNR1/ABCC9/ACTA1/TACR1/NFATC4/CASQ2/F7/KCNMB1/RGS4/NPTX1/FGG/KCNMA1/CHRM2/TRPC1/PCSK1/PRKAA2/ASCL1/TUBA1A/PENK/CD36
BP	GO:0051216	cartilage development	2.49E-06	OGN/PTH1R/GLI3/FGF2/GHR/NKX3-2/CHRDL2/SFRP2/FRZB/NOG/EFEMP1/ZEB1/ZBTB16/COL2A1/SIX2/WNT9A/RSPO2/BMP3/HAND2/MGP/GREM1
BP	GO:0008217	regulation of blood pressure	2.57E-06	CHGA/NPY/TPM1/CMA1/NPR3/ADIPOQ/ACTA2/CTSG/CARTPT/ATP1A2/CNR1/ABCC9/AR/TACR1/TAC3/AGT/GUCY1A1/SERPINF2/ADAMTS16/AGTR1
BP	GO:0032956	regulation of actin cytoskeleton organization	2.60E-06	FERMT2/JAM3/SVIL/SYNPO2/KANK4/TPM1/ACTN2/APOA1/SFRP1/LMOD1/PHLDB2/ELN/CFL2/TMOD1/TACR1/SEMA3E/CCL21/FLNA/RGS4/DIXDC1/SERPINF2/WASF3/GPM6B/MYOC/SLIT2/DAAM2/CXCL12/EPHA3/TGFB3
BP	GO:0048762	mesenchymal cell differentiation	2.62E-06	FERMT2/HTR2B/TGFB1I1/IGF1/CDH2/SFRP1/ALDH1A2/TMEM100/PHLDB2/FGF10/SFRP2/SEMA3E/FRZB/NOG/FLNA/SIX2/DACT3/SOX10/TNXB/MIR145/HAND2/EPHA3/TGFB3/GREM1
BP	GO:0071825	protein-lipid complex subunit	2.84E-06	PLA2G3/APOA1/APOC3/APOA2/PLA2G5/APOB/AGT/MPO/SOAT2/AGTR1
BP	GO:0051952	regulation of amine transport	2.86E-06	CHGA/HTR2A/SV2A/SYT4/CHRNA3/CARTPT/ATP1A2/SLC38A3/CNR1/VIP/AGT/RGS4/TACR2/CXCL12
BP	GO:0055013	cardiac muscle cell development	2.96E-06	IGF1/ACTN2/BVES/PI16/MYH11/PRICKLE1/AGT/RGS4/ASB2/NKX2-5/AKAP6/ACTC1/SGCD
BP	GO:0048754	branching morphogenesis of an epithelial tube	3.02E-06	TMEM59L/GLI3/FGF2/FOXF1/FGF10/GPC3/SFRP2/AR/SEMA3E/NFATC4/AGT/NOG/SIX2/GREB1L/ADAMTS16/RSPO2/SLIT2/GREM1
BP	GO:0035296	regulation of tube diameter	3.13E-06	ADRA1D/HTR2B/HTR2A/VSTM4/CPS1/NPR3/FGA/PRKG1/ATP1A2/TACR1/AGT/KCNA5/KCNMB1/GUCY1A1/FGG/SERPINF2/AGTR1
BP	GO:0097746	blood vessel diameter maintenance	3.13E-06	ADRA1D/HTR2B/HTR2A/VSTM4/CPS1/NPR3/FGA/PRKG1/ATP1A2/TACR1/AGT/KCNA5/KCNMB1/GUCY1A1/FGG/SERPINF2/AGTR1
BP	GO:0035150	regulation of tube size	3.45E-06	ADRA1D/HTR2B/HTR2A/VSTM4/CPS1/NPR3/FGA/PRKG1/ATP1A2/TACR1/AGT/KCNA5/KCNMB1/GUCY1A1/FGG/SERPINF2/AGTR1

BP	GO:0048662	negative regulation of	4.38E-06	OGN/MYOCD/TPM1/NPR3/ADIPOQ/PRKG1/CNN1/VIP/APOD/MIR145/VIPR2/TGFB3
BP	GO:0001823	mesonephros development	4.63E-06	TMEM59L/GLI3/FGF2/SFRP1/FGF10/GPC3/AGT/NOG/ZBTB16/SIX2/GREB1L/ADAMTS16/SLIT2/GREM1
BP	GO:0030048	actin filament-based movement	4.67E-06	KCNE4/PLN/KCNJ8/TPM1/ACTA2/ATP1A2/ANK2/ABCC9/KCNA5/FLNA/CACNB2/SCN4B/KCNH2/ACTC1/SGCD/CACNA1C
BP	GO:0042060	wound healing	5.74E-06	TFPI2/FERMT2/FGF2/IGF1/FBLN1/TPM1/MMRN1/FGA/PRKG1/F13A1/ACTA2/PHLDB2/FGF10/CTSG/FKBP10/CSRP1/NOG/F7/FLNA/SMOC2/DGKG/FGL1/FGG/SERPINF2/SERPIND1/F2/MYL9/MYLK/TGFB3/CD36/IL33/DDR2/VTN
BP	GO:0001649	osteoblast differentiation	6.18E-06	FERMT2/PTH1R/GLI3/FGF2/IGF1/SFRP1/TNC/PRKD1/SFRP2/NOG/IGF2/TWIST2/MYOC/RSPO2/BMP3/HAND2/CYP24A1/TNN/CHRD/GDF10/PENK/DDR2/GREM1
BP	GO:0110053	regulation of actin filament organization	6.20E-06	FERMT2/SVIL/SYNPO2/KANK4/TPM1/ACTN2/APOA1/SFRP1/LMOD1/PHLDB2/ELN/CFL2/TMOD1/TACR1/CCL21/FLNA/RGS4/SERPINF2/WASF3/MYOC/SLIT2/DAAM2/CXCL12/TGFB3
BP	GO:0045785	positive regulation of cell adhesion	6.43E-06	FERMT2/CD274/NPY/GLI3/COL8A1/IGF1/LIMS2/TPM1/MMRN1/FGA/APOA1/SFRP1/FOXF1/CCDC80/COL26A1/ABI3BP/DUSP26/CTSG/TNFSF9/SFRP2/CCL21/NR4A3/FLNA/ZBTB16/IGF2/ELANE/FGG/SERPINF2/AZU1/MYOC/LAMA2/CXCL12/CHRD/CD36/VTN
BP	GO:0031644	regulation of nervous system process	6.62E-06	RELN/CACNG4/SLC8A2/JAM2/TMEM100/CARTPT/NRXN2/NLGN4X/VIP/AGT/NPTX2/RGS4/NPTXR/NPTX1/WASF3/SOX10/IL33
BP	GO:0007015	actin filament organization	6.62E-06	FERMT2/SVIL/SYNPO2/KANK4/TPM1/ACTN2/CALD1/APOA1/DPYSL3/SFRP1/LMOD1/PHLDB2/ELN/CFL2/ACTA1/TMOD1/TACR1/CCL21/FLNA/RGS4/TF/CORO6/TPM2/SERPINF2/WASF3/MYOC/ACTC1/SLIT2/DAAM2/CXCL12/HMCN1/TGFB3/SORBS1
BP	GO:0003002	regionalization	6.64E-06	DLL3/RELN/BARX1/GLI3/FGF2/BICC1/CDON/SFRP1/ALDH1A2/FOXF1/FGF10/NKX3-2/HOXD10/GPC3/SFRP2/AR/PRICKLE1/NOG/ZBTB16/ASB2/MEOX2/SIX2/NKX2-5/EMX1/GREM2/HAND2/DAAM2/HOXA4/CHRD/ASCL1/GREM1/NEUROD1
BP	GO:0007188	adenylate cyclase-modulating G protein-coupled	6.93E-06	ADRA1D/CHGA/PTH1R/PLN/ADCY1/PTGER3/GLP2R/NPR3/AKAP12/UCN3/CNR1/GIP/VIP/FLNA/ADGRD1/PTGFR/ADCY5/CHRM2/GCG/VIPR2/GNAO1/ADCYAP1

BP	GO:0090287	regulation of cellular response to growth factor stimulus	7.12E-06	MYOCD/TGFB1I1/FGF2/RGMA/NGFR/CILP/ASPN/SFRP1/RBPMS2/FGF10/ITGA8/GPC3/CHRDL2/SFRP2/AGT/NOG/ZEB1/SMOC2/DCN/SFRP4/GREM2/TNXB/SLIT2/CHRD/TGFB3/GREM1/CHRDL1/VTN
BP	GO:0055006	cardiac cell development	7.29E-06	IGF1/ACTN2/BVES/PI16/MYH11/PRICKLE1/AGT/RGS4/ASB2/NKX2-5/AKAP6/ACTC1/SGCD
BP	GO:1904062	regulation of cation transmembrane transport	7.52E-06	KCNE4/PLN/KCNK3/ACTN2/ATP1B2/FHL1/FXYD6/ATP1A2/RGN/ANK2/PRKD1/ABCC9/CASQ2/DMD/GRP/FLNA/KCNMB1/RGS4/JPH2/NPSR1/CACNB2/F2/AKAP6/SCN4B/KCNH2/TRPC1/HSPA2/CACNA1C
BP	GO:0007612	learning	7.91E-06	RELN/SLC8A2/PTN/NRXN2/ATP1A2/LRRN4/NLGN4X/BCHE/TACR1/MAP1A/AGT/NOG/NPTX2/NLGN4Y/TACR2/NRXN3/TUBA1A
BP	GO:0007389	pattern specification process	8.23E-06	DLL3/RELN/BARX1/GLI3/FGF2/BICC1/CDON/SFRP1/ALDH1A2/FOXF1/FGF10/NKX3-2/HOXD10/GPC3/SFRP2/AR/PRICKLE1/NOG/ZEB1/ZBTB16/ASB2/MEOX2/SIX2/NKX2-5/EMX1/GREM2/HAND2/DAAM2/HOXA4/CHRD/ASCL1/GREM1/CHRDL1/NEUROD1
BP	GO:0061041	regulation of wound healing	8.37E-06	FERMT2/FGF2/MMRN1/FGA/PRKG1/ACTA2/PHLDB2/F7/SMOC2/FGG/SERPINF2/F2/MYLK/CD36/DDR2/VTN
BP	GO:0030072	peptide hormone secretion	9.08E-06	CHGA/SSTR5/CPE/NNAT/FGA/SFRP1/CARTPT/UCN3/CNR1/GIP/VIP/GRP/KCNA5/FGG/ADCY5/SCT/TACR2/F2/GCG/ADCYAP1/PTPRN/NEUROD1
BP	GO:1903034	regulation of response to wounding	9.31E-06	FERMT2/FGF2/PTN/MMRN1/FGA/PRKG1/ACTA2/PHLDB2/F7/FLNA/SMOC2/FGG/SERPINF2/F2/MYLK/CD36/DDR2/VTN
BP	GO:0008037	cell recognition	1.03E-05	COLEC12/CNTN4/CLGN/TUB/NEXN/CNR1/PLA2G5/GAP43/NDN/CCL21/SPESP1/COLEC11/CRTAC1/TNN/EPHA3/CD36/PTX3
BP	GO:0006813	potassium ion transport	1.11E-05	KCNE4/KCNK3/KCNJ8/HTR2A/ACTN2/ATP1B2/FHL1/ATP1A2/ANK2/ABCC9/KCNH6/VIP/CASQ2/GRP/KCNA5/FLNA/KCNMB1/RGS4/KCNK2/KCNMA1/AKAP6/KCNH2
BP	GO:0051279	regulation of release of sequestered	1.13E-05	PLN/ATP1A2/ANK2/PRKD1/CASQ2/DMD/JPH2/NPSR1/F2/AKAP6/TRPC1/CACNA1C
BP	GO:0010976	positive regulation of neuron	1.22E-05	ROR2/RELN/ALKAL1/PTN/PLA2G3/NEGR1/DPYSL3/ATP1B2/PRKD1/CNR1/AGT/FLNA/STMN2/CNTN1/TNN/EPHA3/DDR2
BP	GO:0002790	peptide secretion	1.26E-05	CHGA/SSTR5/CPE/NNAT/FGA/SFRP1/CARTPT/UCN3/CNR1/GIP/VIP/GRP/KCNA5/FGG/ADCY5/SCT/TACR2/F2/GCG/ADCYAP1/PTPRN/NEUROD1

BP	GO:0034394	protein localization to	1.31E-05	JAM3/ACTN2/GPIHBP1/FGF10/ANK2/FGF7/TMEM35A/MAP1A/FLNA/FBLN5/GPM6B
BP	GO:0055007	cardiac muscle cell differentiation	1.39E-05	MYOCD/IGF1/ACTN2/BVES/PI16/MYH11/PRICKLE1/AGT/RGS4/ASB2/NKX2-5/AKAP6/ACTC1/SGCD/GREM1
BP	GO:0034767	positive regulation of ion transmembrane	1.39E-05	RELN/CACNG4/KCNK3/ACTN2/ATP1B2/RGN/ANK2/DMD/FLNA/KCNMB1/JPH2/NPSR1/CACNB2/F2/AKAP6/KCNH2/TRPC1/HSPA2
BP	GO:0035051	cardiocyte differentiation	1.70E-05	MYOCD/IGF1/ACTN2/BVES/PI16/MYH11/PRICKLE1/AGT/RGS4/ASB2/NKX2-5/AKAP6/MIR145/ACTC1/HAND2/SGCD/GREM1
BP	GO:0043268	positive regulation of	1.85E-05	KCNK3/ACTN2/ATP1B2/FHL1/ANK2/FLNA/KCNMB1/AKAP6/KCNH2
BP	GO:0048588	developmental cell growth	1.86E-05	IGF1/NTN1/PRKG1/PI16/SYT4/SLIT3/NDN/PRICKLE1/SEMA3E/AGT/RGS4/RND2/MAP1B/EMX1/PLXNA4/AKAP6/NGF/SLIT2/TNN/CXCL12/EPHA7
BP	GO:0001658	branching involved in	1.90E-05	TMEM59L/GLI3/FGF2/GPC3/AGT/NOG/SIX2/GREB1L/ADAMTS16/GREM1
BP	GO:0035567	non-canonical Wnt signaling pathway	1.97E-05	ROR2/SFRP1/RSPO3/GPC3/SFRP2/PRICKLE1/FRZB/SFRP4/MYOC/DAAM2/PRICKLE2
BP	GO:0010744	positive regulation of macrophage	2.02E-05	PLA2G3/PLA2G5/APOB/AGT/AGTR1/CD36
BP	GO:0040013	negative regulation of locomotion	2.17E-05	MYOCD/FGF2/FBLN1/PODN/TPM1/NGFR/ADIPOQ/PRKG1/DPYSL3/SFRP1/ATP1B2/PHLDB2/HAS1/RGN/SFRP2/SEMA3E/CCL21/NOG/ELANE/MEOX2/DCN/CYP1B1/APOD/MIR145/RECK/SLIT2/TNN/CXCL12/CHRD/IL33/GREM1
BP	GO:0010742	macrophage derived foam	2.23E-05	PLA2G3/ADIPOQ/PLA2G5/APOB/AGT/SOAT2/AGTR1/CD36
BP	GO:0071805	potassium ion transmembrane transport	2.40E-05	KCNE4/KCNK3/KCNJ8/ACTN2/ATP1B2/FHL1/ATP1A2/ANK2/ABCC9/KCNH6/CASQ2/GRP/KCNA5/FLNA/KCNMB1/RGS4/KCNK2/KCNMA1/AKAP6/KCNH2
BP	GO:0060348	bone development	2.41E-05	FREM1/OGN/PAPPA2/GLI3/GHR/IGF1/BNC2/RGN/LEPR/RAB23/LRRC17/FLNA/ZBTB16/COL2A1/SFRP4/MYOC/THPO/TNN/TGFB3/DDR2/GREM1
BP	GO:0001822	kidney development	2.43E-05	TMEM59L/GLI3/FGF2/KCNJ8/BICC1/GFRA1/ADIPOQ/SFRP1/ALDH1A2/ACTA2/FGF10/ITGA8/RGN/GPC3/PYGO1/AGT/NOG/ZBTB16/SIX2/GREB1L/ADAMTS16/AGTR1/SLIT2/EPHA7/GREM1

BP	GO:2000146	negative regulation of cell motility	2.57E-05	MYOCD/FGF2/FBLN1/PODN/TPM1/NGFR/ADIPOQ/PRKG1/DPYSL3/SFRP1/ATP1B2/PHLDB2/HAS1/RGN/SFRP2/CCL21/NOG/MEOX2/DCN/CYP1B1/APOD/MIR145/RECK/SLIT2/TNN/CXCL12/CHRD/IL33/GREM1
BP	GO:0090077	foam cell differentiation	2.73E-05	PLA2G3/ADIPOQ/PLA2G5/APOB/AGT/SOAT2/AGTR1/CD36
BP	GO:0099623	regulation of cardiac muscle	2.84E-05	KCNE4/ANK2/KCNH6/KCNA5/FLNA/SCN4B/KCNH2
BP	GO:0045834	positive regulation of lipid metabolic	2.87E-05	HTR2B/FGF2/TNFAIP8L3/HTR2A/PLA2G3/ADIPOQ/APOA1/RGN/PRKD1/APOA2/CES1/CCL21/NR4A3/SCT/F2/SORBS1
BP	GO:0061448	connective tissue development	2.89E-05	OGN/PTH1R/GLI3/FGF2/GHR/EBF2/ACTA2/NKX3-2/CHRDL2/SFRP2/FRZB/NOG/EFEMP1/ZEB1/ZBTB16/COL2A1/SIX2/WNT9A/RSPO2/BMP3/HAND2/MGP/GREM1
BP	GO:1903036	positive regulation of	2.91E-05	FERMT2/PTN/F7/FLNA/SMOC2/SERPINF2/F2/MYLK/CD36/DDR2/VTN
BP	GO:0014910	regulation of smooth muscle	2.97E-05	MYOCD/IGF1/TPM1/ADIPOQ/PRKG1/TACR1/NR4A3/AGT/CYP1B1/SLIT2/DDR2/VTN
BP	GO:0060562	epithelial tube morphogenesis	3.09E-05	TMEM59L/GLI3/FGF2/RGMA/NTN1/SFRP1/FOXF1/FGF10/GPC3/SFRP2/AR/PRICKLE1/SEMA3E/NFATC4/AGT/NOG/ASB2/SIX2/NKX2-5/GREB1L/ADAMTS16/RSPO2/HAND2/SLIT2/EPHA7/GREM1
BP	GO:1904064	positive regulation of cation	3.11E-05	KCNK3/ACTN2/ATP1B2/RGN/ANK2/DMD/FLNA/KCNMB1/JPH2/NPSR1/CACNB2/F2/AKAP6/KCNH2/TRPC1/HSPA2
BP	GO:0043267	negative regulation of	3.32E-05	KCNE4/HTR2A/ACTN2/VIP/CASQ2/GRP/RGS4/KCNH2
BP	GO:0099622	cardiac muscle cell membrane	3.32E-05	KCNE4/KCNJ8/ANK2/KCNH6/KCNA5/FLNA/SCN4B/KCNH2
BP	GO:0015833	peptide transport	3.39E-05	CHGA/SSTR5/CPE/NNAT/FGA/SFRP1/CARTPT/UCN3/CNR1/GIP/VIP/GRP/KCNA5/FGG/ADCY5/SCT/TACR2/F2/GCG/ADCYAP1/PTPRN/NEUROD1
BP	GO:0006940	regulation of smooth muscle	3.40E-05	MYOCD/PRKG1/CHRNA3/ATP1A2/TACR1/CNN1/GUCY1A1/TACR2/CHRM2/MIR145
BP	GO:0030199	collagen fibril organization	3.40E-05	DPT/SFRP2/FKBP10/SERPINF2/COL2A1/COL14A1/CYP1B1/TNXB/DDR2/GREM1
BP	GO:0019722	calcium-mediated signaling	3.41E-05	HTR2B/PLN/SLC8A2/IGF1/TMEM100/ATP1A2/RGN/ANK2/RCAN2/NFATC4/CASQ2/DMD/PTGFR/AZU1/AKAP6/AGTR1/SGCD/CACNA1C
BP	GO:0045666	positive regulation of neuron	3.71E-05	BEND6/FGF2/CDON/ZEB1/MAP1B/NKX2-5/PCP4/NGF/NAP1L2/CXCL12/ASCL1/NEUROD1

BP	GO:1902905	positive regulation of supramolecular fiber	3.72E-05	FERMT2/SYNPO2/TPM1/ACTN2/APOA1/SFRP1/LMOD1/CFL2/TACR1/CCL21/SERPINF2/MAP1B/WASF3/STMN2/MYOC/TNXB/TGFB3
BP	GO:0009410	response to xenobiotic stimulus	3.85E-05	SULT1C4/HTR2B/SLCO1B3/KCNK3/ADCY1/PDE3A/CES3/HTR2A/CPS1/ADIPOQ/SFRP1/AMH/AIM2/APOA2/SFRP2/BCHE/CA9/PRICKLE1/GIP/ANKRD1/MAP1B/EMX1/AOX1/CYP1B1/SOX10/SULT2A1/FMO1/KCNH2/PCSK1/PRKAA2/NEUROD1
BP	GO:1901381	positive regulation of	4.01E-05	KCNK3/ACTN2/ATP1B2/ANK2/FLNA/KCNMB1/AKAP6/KCNH2
BP	GO:0051051	negative regulation of transport	4.01E-05	KCNE4/CHGA/PLN/PTGER3/HTR2A/PRTN3/ACTN2/ADIPOQ/SFRP1/FOXF1/SYT4/CARTPT/APOC3/ATP1A2/PRKD1/CNR1/APOA2/RAB23/VIP/CASQ2/GRP/CRYAB/RGS4/NPSR1/MAP1B/SCT/TACR2/GPM6B/SFRP4/APOD/KCNH2/EPHA3/CD36
BP	GO:0007586	digestion	4.09E-05	PTGER3/PNLIP/NPR3/APOA1/FGF10/CTRB1/UCN3/APOA2/TACR1/PNLIPRP2/NPSR1/SCT/SOAT2/CD36/NEUROD1
BP	GO:0086001	cardiac muscle cell action	4.21E-05	KCNE4/KCNJ8/ATP1A2/ANK2/DMD/KCNA5/FLNA/CACNB2/SCN4B/KCNH2/CACNA1C
BP	GO:0060675	ureteric bud morphogenesis	4.47E-05	TMEM59L/GLI3/FGF2/GPC3/AGT/NOG/SIX2/GREB1L/ADAMTS16/GREM1
BP	GO:0010880	regulation of release of sequestered calcium ion into	4.53E-05	PLN/ATP1A2/ANK2/CASQ2/DMD/AKAP6/CACNA1C
BP	GO:0086091	regulation of heart rate by	4.81E-05	KCNE4/ANK2/KCNH6/KCNA5/CACNB2/SCN4B/KCNH2/CACNA1C
BP	GO:0090276	regulation of peptide hormone secretion	4.84E-05	CHGA/SSTR5/NNAT/FGA/SFRP1/CARTPT/UCN3/CNR1/GIP/GRP/KCNA5/FGG/ADCY5/SCT/F2/GCG/ADCYAP1/NEUROD1
BP	GO:0086002	cardiac muscle cell action potential	4.95E-05	KCNE4/KCNJ8/ANK2/KCNA5/FLNA/CACNB2/SCN4B/KCNH2/CACNA1C
BP	GO:0072171	mesonephric tubule	5.10E-05	TMEM59L/GLI3/FGF2/GPC3/AGT/NOG/SIX2/GREB1L/ADAMTS16/GREM1
BP	GO:0010881	regulation of cardiac muscle contraction by regulation of	5.11E-05	PLN/ATP1A2/ANK2/CASQ2/DMD/CACNA1C
BP	GO:0044241	lipid digestion	5.11E-05	PNLIP/APOA1/APOA2/PNLIPRP2/SOAT2/CD36
BP	GO:0014812	muscle cell migration	5.12E-05	THBS4/MYOCD/IGF1/TPM1/ADIPOQ/PRKG1/TACR1/NR4A3/AGT/CYP1B1/SLIT2/DDR2/VTN
BP	GO:0060019	radial glial cell	5.25E-05	GLI3/CDH2/FGF10/GAP43/EMX1

BP	GO:0008344	adult locomotory behavior	5.33E-05	SEZ6/HOXD10/ATP1A2/TMOD1/GIP/GLRB/LGI4/CHL1/DMBX1/CXCL12/TUBA1A
BP	GO:0045664	regulation of neuron differentiation	5.55E-05	RELN/BEND6/GLI3/FGF2/CDON/CNTN4/SFRP1/SFRP2/ZEB1/DIXDC1/MAP1B/NKX2-5/PCP4/NGF/NAP1L2/CXCL12/ASCL1/NEUROD1
BP	GO:0010743	regulation of macrophage	5.64E-05	PLA2G3/ADIPOQ/PLA2G5/APOB/AGT/AGTR1/CD36
BP	GO:0061037	negative regulation of	5.64E-05	GLI3/NKX3-2/FRZB/NOG/EFEMP1/WNT9A/GREM1
BP	GO:0044058	regulation of digestive system	5.75E-05	PTGER3/APOA1/FGF10/APOA2/TACR1/NPSR1/SCT/NEUROD1
BP	GO:0034381	plasma lipoprotein	5.76E-05	CES3/ADIPOQ/APOA1/GPIHBP1/APOC3/APOA2/APOB/SOAT2/CD36
BP	GO:0090288	negative regulation of cellular	5.79E-05	FGF2/NGFR/SFRP1/RBPMS2/CHRDL2/SFRP2/AGT/NOG/DCN/GREM2/SLIT2/CHRD/GREM1/CHRDL1
BP	GO:0002791	regulation of peptide secretion	5.93E-05	CHGA/SSTR5/NNAT/FGA/SFRP1/CARTPT/UCN3/CNR1/GIP/GRP/KCNA5/FGG/ADCY5/SCT/F2/GCG/ADCYAP1/NEUROD1
BP	GO:1903532	positive regulation of secretion by cell	6.25E-05	NNAT/IGF1/PLA2G3/FGA/MYOM1/SYT4/CARTPT/UCN3/TACR1/GIP/ANKRD1/VIP/AGT/GRP/FGG/SCT/TACR2/F2/GCG/PCSK1/ADCYAP1/CXCL12/TGFB3
BP	GO:0001657	ureteric bud development	6.30E-05	TMEM59L/GLI3/FGF2/SFRP1/GPC3/AGT/NOG/SIX2/GREB1L/ADAMTS16/SLIT2/GREM1
BP	GO:0014909	smooth muscle cell migration	6.30E-05	MYOCD/IGF1/TPM1/ADIPOQ/PRKG1/TACR1/NR4A3/AGT/CYP1B1/SLIT2/DDR2/VTN
BP	GO:0042886	amide transport	6.46E-05	CHGA/SSTR5/CPE/NNAT/FGA/SFRP1/SYT4/CARTPT/ATP1A2/SLC38A3/UCN3/CNR1/GIP/VIP/GRP/KCNA5/FGG/ADCY5/SCT/TACR2/F2/GCG/AQP8/ADCYAP1/PTPRN/NEUROD1
BP	GO:0043410	positive regulation of MAPK cascade	6.47E-05	ROR2/FERMT2/HTR2B/ALKAL1/NPY/FGF2/GHR/IGF1/TNFAIP8L3/HTR2A/CDON/CDH2/FGA/ACTA2/FGF10/CARTPT/AKAP12/AR/PLA2G5/CCL21/DIXDC1/IGF2/ELANE/FGG/NPSR1/SERPINF2/GCG/THPO/HAND2/ADCYAP1/TGFB3/CD36/DDR2
BP	GO:0051651	maintenance of location in cell	6.59E-05	HTR2B/PLN/FGF2/PLCH2/GRIK5/HTR2A/ATP1A2/ANK2/DZIP1/PRKD1/CCL21/CASQ2/DMD/FLNA/JPH2/NPSR1/F2/AKAP6/TRPC1/CACNA1C
BP	GO:1901016	regulation of potassium ion transmembrane	6.61E-05	KCNE4/KCNK3/ACTN2/ATP1B2/FHL1/ANK2/ABCC9/CASQ2/GRP/AKAP6

BP	GO:0009612	response to mechanical stimulus	6.75E-05	FGF2/PTN/HTR2A/CDH2/NRXN2/ATP1A2/ACTA1/TACR1/ANKRD1/AGT/DMD/KCNA5/KCNK2/MPO/MAP1B/CXCL12/TUBA1A/IL33/DDR2
BP	GO:0090087	regulation of peptide transport	6.78E-05	CHGA/SSTR5/NNAT/FGA/SFRP1/CARTPT/UCN3/CNR1/GIP/GRP/KCNA5/FGG/ADCY5/SCT/F2/GCG/ADCYAP1/NEUROD1
BP	GO:0048566	embryonic digestive tract	6.96E-05	GLI3/ALDH1A2/FOXF1/RBPMS2/FGF10/SCT/SIX2
BP	GO:0072163	mesonephric epithelium development	6.97E-05	TMEM59L/GLI3/FGF2/SFRP1/GPC3/AGT/NOG/SIX2/GREB1L/ADAMTS16/SLIT2/GREM1
BP	GO:0072164	mesonephric tubule development	6.97E-05	TMEM59L/GLI3/FGF2/SFRP1/GPC3/AGT/NOG/SIX2/GREB1L/ADAMTS16/SLIT2/GREM1
BP	GO:0006816	calcium ion transport	7.20E-05	CACNA1H/HTR2B/PLN/CACNG4/SLC8A2/FGF2/TRPC4/PLCH2/HTR2A/SCN7A/ATP1A2/RGN/ANK2/PRKD1/CCL21/CASQ2/AGT/DMD/FLNA/RGS4/JPH2/NPSR1/CACNB2/F2/AKAP6/GCG/TRPC1/MYLK/CXCL12/HSPA2/CACNA1C
BP	GO:0035107	appendage morphogenesis	7.23E-05	GLI3/ALDH1A2/FGF10/HOXD10/GPC3/SFRP2/PRICKLE1/NOG/ZBTB16/COL2A1/RSPO2/RECK/HAND2/CACNA1C/GREM1
BP	GO:0035108	limb morphogenesis	7.23E-05	GLI3/ALDH1A2/FGF10/HOXD10/GPC3/SFRP2/PRICKLE1/NOG/ZBTB16/COL2A1/RSPO2/RECK/HAND2/CACNA1C/GREM1
BP	GO:0072378	blood coagulation,	7.67E-05	FBLN1/FGA/F13A1/FLNA/FGG
BP	GO:0045667	regulation of osteoblast differentiation	7.81E-05	FERMT2/GLI3/FGF2/IGF1/SFRP1/PRKD1/SFRP2/NOG/TWIST2/HAND2/TNN/CHRD/GDF10/DDR2/GREM1
BP	GO:0048736	appendage development	8.16E-05	FERMT2/GLI3/ALDH1A2/FGF10/HOXD10/GPC3/SFRP2/PRICKLE1/NOG/ZBTB16/MEOX2/COL2A1/RSPO2/RECK/HAND2/CACNA1C/GREM1
BP	GO:0060173	limb development	8.16E-05	FERMT2/GLI3/ALDH1A2/FGF10/HOXD10/GPC3/SFRP2/PRICKLE1/NOG/ZBTB16/MEOX2/COL2A1/RSPO2/RECK/HAND2/CACNA1C/GREM1
BP	GO:0030336	negative regulation of cell migration	8.38E-05	MYOCD/FGF2/PODN/TPM1/NGFR/ADIPOQ/PRKG1/DPYSL3/SFRP1/ATP1B2/PHLDB2/HAS1/SFRP2/CCL21/NOG/MEOX2/DCN/CYP1B1/APOD/MIR145/RECK/SLIT2/TNN/CXCL12/CHRD/IL33/GREM1
BP	GO:0007626	locomotory behavior	8.79E-05	SEZ6/RELN/NEGR1/IDO1/CHRNA3/HOXD10/ATP1A2/TMOD1/GIP/GLRB/LGI4/ADCY5/CHL1/CNTN1/DMBX1/CXCL12/TUBA1A/PENK

BP	GO:0030900	forebrain development	8.88E-05	RELN/NPY/GLI3/FGF2/FOXP2/CDON/CDH2/PRKG1/ALDH1A2/ATP1B2/FGF10/ATP1A2/SEMA3E/IGF2BP1/NOG/ZEB1/DIXDC1/CCKAR/SCT/EMX1/PLXNA4/DCLK2/PCSK1/SLIT2/CXCL12/ASCL1/TUBA1A/NEUROD1
BP	GO:0007178	transmembrane receptor protein serine/threonine kinase signaling pathway	9.27E-05	FERMT2/MYOCD/TGFB1I1/RGMA/CILP/ASPN/SFRP1/AMH/TMEM100/RBPMS2/FGF10/ITGA8/GPC3/CHRDL2/SFRP2/NOG/ZEB1/TF/SFRP4/GREM2/BMP3/AFP/TNXB/CHRD/GDF10/TGFB3/GREM1/CHRDL1
BP	GO:0048041	focal adhesion assembly	9.31E-05	FERMT2/ACTN2/SFRP1/PHLDB2/GPM6B/APOD/MYOC/STON1/EPHA3/GREM1/SORBS1
BP	GO:0048709	oligodendrocyte differentiation	9.38E-05	CNTNAP1/GLI3/TRPC4/PTN/WASF3/GPM6B/CNTN1/SOX10/DAAM2/ASCL1/IL33/VTN
BP	GO:0008038	neuron recognition	9.50E-05	CNTN4/NEXN/CNR1/GAP43/NDN/CRTAC1/TNN/EPHA3
BP	GO:0048678	response to axon injury	0.000104	FGF2/PTN/DPYSL3/TNC/GAP43/GIP/FLNA/KCNK2/MAP1B/APOD/PCSK1
BP	GO:0014808	release of sequestered calcium ion into	0.000104	PLN/ATP1A2/ANK2/CASQ2/DMD/AKAP6/CACNA1C
BP	GO:0070588	calcium ion transmembrane transport	0.000107	CACNA1H/HTR2B/PLN/CACNG4/SLC8A2/FGF2/TRPC4/PLCH2/HTR2A/SCN7A/ATP1A2/RGN/ANK2/PRKD1/CCL21/CASQ2/DMD/FLNA/JPH2/NPSR1/CACNB2/F2/AKAP6/TRPC1/HSPA2/CACNA1C
BP	GO:0050795	regulation of behavior	0.000108	HTR2B/RELN/NPY/GRPR/LEPR/CNR1/NR4A3/GRP/NPSR1/PENK
BP	GO:0007158	neuron cell-cell	0.000109	CNTN4/NRXN2/NLGN4X/NLGN4Y/NRXN3
BP	GO:0050919	negative chemotaxis	0.000111	NTN1/APOA1/SLIT3/SEMA3E/PLXNA4/FLRT2/SLIT2/EPHA7
BP	GO:0003081	regulation of systemic arterial blood	0.000112	CMA1/CTSG/TACR1/AGT/SERPINF2/AGTR1
BP	GO:0086010	membrane depolarization	0.000112	ATP1A2/ANK2/CACNB2/SCN4B/KCNH2/CACNA1C
BP	GO:0050878	regulation of body fluid levels	0.000112	TFPI2/CDO1/FBLN1/MMRN1/NPR3/FGA/PRKG1/F13A1/FGF10/CTSG/PRICKLE1/TACR1/CSRP1/VIP/F7/FLNA/DGKG/FGL1/FGG/SERPINF2/SERPIND1/SCT/F2/MYL9/CD36/VTN
BP	GO:0150115	cell-substrate junction organization	0.000114	FERMT2/ACTN2/SFRP1/PHLDB2/PRICKLE1/GPM6B/APOD/MYOC/STON1/EPHA3/GREM1/SORBS1
BP	GO:0071772	response to BMP	0.000114	RGMA/SFRP1/TMEM100/RBPMS2/GPC3/CHRDL2/SFRP2/NOG/COL2A1/SFRP4/GREM2/BMP3/CHRD/GDF10/TGFB3/GREM1/CHRDL1

BP	GO:0071773	cellular response to BMP stimulus	0.000114	RGMA/SFRP1/TMEM100/RBPMS2/GPC3/CHRDL2/SFRP2/NOG/COL2A1/SFRP4/GREM2/BMP3/CHRD/GDF10/TGFB3/GREM1/CHRDL1
BP	GO:0090092	regulation of transmembrane receptor protein serine/threonine	0.000116	MYOCD/TGFB1I1/RGMA/CILP/ASPN/SFRP1/RBPMS2/FGF10/ITGA8/GPC3/CHRDL2/SFRP2/NOG/ZEB1/SFRP4/GREM2/BMP3/TNXB/CHRD/GDF10/TGFB3/GREM1/CHRDL1
BP	GO:0007596	blood coagulation	0.000117	TFPI2/FBLN1/MMRN1/FGA/PRKG1/F13A1/CTSG/CSRP1/F7/FLNA/DGKG/FGL1/FGG/SERPINF2/SERPIND1/F2/MYL9/CD36/VTN
BP	GO:0003007	heart morphogenesis	0.000117	HTR2B/CPE/TPM1/ZFPM2/ALDH1A2/FOXF1/TMEM100/SLIT3/ELN/SFRP2/PRICKLE1/ANKRD1/NOG/FLNA/ASB2/COL2A1/NKX2-5/ACTC1/FLRT2/HAND2/SLIT2
BP	GO:0050679	positive regulation of epithelial cell proliferation	0.000124	THBS4/HTR2B/FGF2/IGF1/PTN/SFRP1/FGF10/FGF7/PRKD1/AR/TACR1/NR4A3/VIP/NOG/IGF2/NKX2-5/AGTR1/CXCL12/PTPRN
BP	GO:0019934	cGMP-mediated signaling	0.000125	HTR2B/PDE3A/IRAG1/PRKG1/PDE9A/GUCY1A1/CD36
BP	GO:1903514	release of sequestered calcium ion into	0.000125	PLN/ATP1A2/ANK2/CASQ2/DMD/AKAP6/CACNA1C
BP	GO:0030326	embryonic limb morphogenesis	0.000126	GLI3/ALDH1A2/HOXD10/GPC3/SFRP2/PRICKLE1/NOG/ZBTB16/RSPO2/RECK/HAND2/CACNA1C/GREM1
BP	GO:0035113	embryonic appendage morphogenesis	0.000126	GLI3/ALDH1A2/HOXD10/GPC3/SFRP2/PRICKLE1/NOG/ZBTB16/RSPO2/RECK/HAND2/CACNA1C/GREM1
BP	GO:0019216	regulation of lipid metabolic process	0.000131	HTR2B/FGF2/TNFAIP8L3/HTR2A/PLA2G3/ADIPOQ/APOA1/C1QTNF2/APOC3/RGN/PDK4/PRKD1/CNR1/APOA2/CES1/GIP/APOB/CCL21/NR4A3/SCT/F2/APOD/AQP8/FMO1/SORBS1
BP	GO:0019229	regulation of vasoconstriction	0.000132	ADRA1D/HTR2A/FGA/ATP1A2/TACR1/AGT/KCNA5/FGG/AGTR1
BP	GO:0051480	regulation of cytosolic	0.000132	NPY/PLN/SLC8A2/TRPC4/CNR1/SCGN/CACNB2/F2/TRPC1
BP	GO:0086065	cell communication	0.000132	ATP1B2/ATP1A2/ANK2/CASQ2/KCNA5/FLNA/CACNB2/SCN4B/CACNA1C
BP	GO:0042698	ovulation cycle	0.000136	PTN/AMH/SLIT3/CYP1B1/AFP/SLIT2/GDF10/TGFB3/PTPRN/PTX3
BP	GO:0072078	nephron tubule morphogenesis	0.000136	TMEM59L/GLI3/FGF2/GPC3/AGT/NOG/SIX2/GREB1L/ADAMTS16/GREM1
BP	GO:1901654	response to ketone	0.000136	ADCY1/CPS1/SFRP1/SLIT3/UCN3/AR/CA9/TACR1/CCL21/F7/PTGFR/ADCY5/CYP1B1/SOX10/SLIT2/FBXO32/PRKAA2/TGFB3

BP	GO:0030282	bone mineralization	0.000137	ROR2/PTH1R/IGF1/PTN/ASPN/GPC3/AHSG/PRICKLE1/GPM6B/RSPO2/MGP/DDR2/GREM1
BP	GO:0043500	muscle adaptation	0.000137	IGF1/PI16/PDE9A/ACTA1/NR4A3/AGT/RGS4/SGCA/AKAP6/MYOC/MIR145/HAND2/FBXO32
BP	GO:0051495	positive regulation of cytoskeleton organization	0.000138	FERMT2/SYNPO2/TPM1/ACTN2/APOA1/SFRP1/LMOD1/CFL2/TACR1/CCL21/SERPINF2/MAP1B/WASF3/STMN2/MYOC/DAAM2/TGFB3
BP	GO:0045444	fat cell differentiation	0.000139	FERMT2/TGFB1I1/FNDC5/EBF2/HTR2A/ZFPM2/LMO3/ADIPOQ/SFRP1/CLIP3/FABP4/SLC2A4/FGF10/SFRP2/FRZB/NR4A3/ZBTB16/MEDAG/GDF10/RUNX1T1
BP	GO:0010882	regulation of cardiac muscle contraction by	0.000141	PLN/ATP1A2/ANK2/CASQ2/DMD/CACNA1C
BP	GO:2000050	regulation of non-canonical	0.000141	SFRP1/RSPO3/GPC3/SFRP2/SFRP4/DAAM2
BP	GO:0030308	negative regulation of cell growth	0.000148	NTN1/SFRP1/PI16/FHL1/RERG/SLIT3/SFRP2/SEMA3E/FRZB/AGT/CRYAB/RGS4/MEG3/DACT3/SLIT2/EPHA7/GREM1
BP	GO:0048557	embryonic digestive tract	0.00015	GLI3/FOXF1/RBPMS2/FGF10/SIX2
BP	GO:0001941	postsynaptic membrane	0.00015	RELN/CDH2/NRXN2/NLGN4X/GLRB/NLGN4Y/CHRDL1
BP	GO:0086019	cell-cell signaling	0.00015	ANK2/CASQ2/KCNA5/FLNA/CACNB2/SCN4B/CACNA1C
BP	GO:0032233	positive regulation of	0.00015	FERMT2/SYNPO2/TPM1/APOA1/SFRP1/TACR1/SERPINF2/MYOC/TGFB3
BP	GO:0090303	positive regulation of	0.00015	FERMT2/F7/SMOC2/SERPINF2/F2/MYLK/CD36/DDR2/VTN
BP	GO:0051235	maintenance of location	0.00015	HTR2B/PLN/FGF2/PLCH2/GRIK5/HTR2A/CPS1/APOA1/ATP1A2/ANK2/DZIP1/PRKD1/CES1/APOB/CCL21/CASQ2/DMD/FLNA/JPH2/NPSR1/F2/AKAP6/TRPC1/CACNA1C/CD36
BP	GO:0010171	body morphogenesis	0.000151	CDON/PHLDB2/GPC3/PRICKLE1/NOG/GREM2/CRISPLD1/TGFB3
BP	GO:0007422	peripheral nervous system	0.000152	CNTNAP1/HOXD10/LGI4/NFASC/GFRA3/PLXNA4/SOX10/MYOC/HAND2/ASCL1
BP	GO:0050817	coagulation	0.000156	TFPI2/FBLN1/MMRN1/FGA/PRKG1/F13A1/CTSG/CSRP1/F7/FLNA/DGKG/FGL1/FGG/SERPINF2/SERPIND1/F2/MYL9/CD36/VTN
BP	GO:0022600	digestive system process	0.000164	PTGER3/PNLIP/NPR3/APOA1/FGF10/APOA2/TACR1/NPSR1/SCT/SOAT2/CD36/NEUROD1

BP	GO:0007599	hemostasis	0.000165	TFPI2/FBLN1/MMRN1/FGA/PRKG1/F13A1/CTSG/CSRP1/F7/FLNA/DGKG/FGL1/FGG/SERPINF2/SERPIND1/F2/MYL9/CD36/VTN
BP	GO:0007189	adenylate cyclase-activating G	0.000166	ADRA1D/CHGA/PTH1R/PLN/ADCY1/PTGER3/UCN3/GIP/VIP/ADGRD1/PTGFR/ADCY5/GCG/ADCYAP1
BP	GO:0072088	nephron epithelium	0.000169	TMEM59L/GLI3/FGF2/GPC3/AGT/NOG/SIX2/GREB1L/ADAMTS16/GREM1
BP	GO:0048546	digestive tract morphogenesis	0.000175	GLI3/SFRP1/FOXF1/RBPMS2/FGF10/SFRP2/SIX2/SOX10
BP	GO:0032411	positive regulation of transporter	0.000176	RELN/CACNG4/KCNK3/ACTN2/ADIPOQ/ATP1B2/RGN/ANK2/DMD/JPH2/CACNB2/AKAP6/HSPA2
BP	GO:0030509	BMP signaling pathway	0.00018	RGMA/SFRP1/TMEM100/RBPMS2/GPC3/CHRDL2/SFRP2/NOG/SFRP4/GREM2/BMP3/CHRD/GDF10/TGFB3/GREM1/CHRDL1
BP	GO:0048017	inositol lipid-mediated signaling	0.00018	ROR2/HTR2B/RELN/FGF2/IGF1/PLCH2/TNFAIP8L3/HTR2A/MUC5AC/NPR3/SEMA3E/AGT/DCN/F2/MYOC/DDR2
BP	GO:0048251	elastic fiber	0.000189	MYH11/FBLN5/TNXB/MFAP4
BP	GO:0097104	postsynaptic	0.000189	CDH2/NRXN2/NLGN4X/NLGN4Y
BP	GO:1905918	regulation of CoA-transferase	0.000189	APOA1/APOA2/AGT/AGTR1
BP	GO:0009306	protein secretion	0.000189	CHGA/SSTR5/NNAT/IGF1/ARL4D/FGA/SFRP1/MYOM1/SYT4/CARTPT/UCN3/CNR1/GIP/ANKRD1/VIP/KCNA5/FGG/ADCY5/TACR2/F2/GCG/PCSK1/ADCYAP1/TGFB3/PTPRN/NEUROD1
BP	GO:0045926	negative regulation of growth	0.000192	NTN1/SFRP1/PI16/FHL1/RERG/SLIT3/GPC3/SFRP2/SEMA3E/FRZB/AGT/NOG/CRYAB/RGS4/KCNK2/MEG3/DACT3/SLIT2/EPHA7/GREM1
BP	GO:0097305	response to alcohol	0.000192	CDO1/ADCY1/ADIPOQ/SFRP1/SLC2A4/SLIT3/UCN3/CNR1/CES1/TACR1/CCL21/F7/KCNMB1/RGS4/PTGFR/ADCY5/CYP1B1/SLIT2/PRKAA2/PENK
BP	GO:0007631	feeding behavior	0.000195	NPY/NEGR1/CARTPT/LEPR/CNR1/TACR1/NR4A3/AGT/NPSR1/GCG/DMBX1/HAND2
BP	GO:0035592	establishment of protein localization to extracellular region	0.000197	CHGA/SSTR5/NNAT/IGF1/ARL4D/FGA/SFRP1/MYOM1/SYT4/CARTPT/UCN3/CNR1/GIP/ANKRD1/VIP/KCNA5/FGG/ADCY5/TACR2/F2/GCG/PCSK1/ADCYAP1/TGFB3/PTPRN/NEUROD1
BP	GO:0043113	receptor clustering	0.000201	RELN/PTN/GRIK5/CDH2/NRXN2/GLRB/FLNA/CHRDL1
BP	GO:0060688	regulation of morphogenesis of	0.000201	SFRP1/FGF10/FGF7/AR/AGT/NOG/SIX2/GREM1
BP	GO:0030299	intestinal cholesterol	0.000202	PNLIP/APOA1/APOA2/SOAT2/CD36
BP	GO:0072376	protein	0.000202	FBLN1/FGA/F13A1/FLNA/FGG

BP	GO:0060560	developmental growth involved in morphogenesis	0.000206	NTN1/PRKG1/SFRP1/SYT4/FGF10/SLIT3/SFRP2/NDN/PRICKLE1/SEMA3E/RND2/MAP1B/EMX1/PLXNA4/NGF/SLIT2/TNN/CXCL12/EPHA7
BP	GO:0003231	cardiac ventricle development	0.000207	MYOCD/CPE/TPM1/ZFPM2/FOXF1/SLIT3/SFRP2/NOG/KCNK2/NKX2-5/GREB1L/HAND2/SLIT2
BP	GO:0051960	regulation of nervous system development	0.00021	DLL3/RELN/GLI3/FGF2/JAM2/PTN/PTPRD/CDON/NTN1/SYT4/SEMA3E/NFATC4/LGI4/NOG/DIXDC1/RND2/MAP6/MAP1B/WASF3/PLXNA4/F2/NGF/SOX10/FLRT2/SLIT2/DAAM2/CXCL12/ASCL1/IL33/EPHA7
BP	GO:0042310	vasoconstriction	0.00021	ADRA1D/HTR2B/HTR2A/FGA/ATP1A2/TACR1/AGT/KCNA5/FGG/AGTR1
BP	GO:0061333	renal tubule morphogenesis	0.00021	TMEM59L/GLI3/FGF2/GPC3/AGT/NOG/SIX2/GREB1L/ADAMTS16/GREM1
BP	GO:0010717	regulation of epithelial to mesenchymal	0.000213	FERMT2/TGFB1I1/SFRP1/PHLDB2/SFRP2/NOG/DACT3/TNXB/MIR145/EPHA3/TGFB3/GREM1
BP	GO:0090263	positive regulation of canonical Wnt	0.000213	FGF2/SFRP1/FGF10/RSPO3/GPC3/SFRP2/SFRP4/RSPO2/MIR145/RECK/RSPO1/DAAM2
BP	GO:0007416	synapse assembly	0.000214	PCDHB4/PTPRD/PCDHB5/NTN1/CDH2/NEGR1/LGI2/NRXN2/NLGN4X/GAP43/DNER/NPTX1/MAP1B/NLGN4Y/FLRT2/SDK2/EPHA7
BP	GO:0051017	actin filament bundle assembly	0.000217	FERMT2/SYNPO2/TPM1/CALD1/APOA1/DPYSL3/SFRP1/PHLDB2/ELN/TACR1/FLNA/SERPINF2/MYOC/TGFB3/SORBS1
BP	GO:1905330	regulation of morphogenesis of	0.000218	FGF2/SFRP1/FGF10/FGF7/AR/AGT/NOG/SIX2/GREM1
BP	GO:0014072	response to isoquinoline	0.000219	GHR/CNR1/TACR1/RGS4/PCSK1/PENK
BP	GO:0043278	response to	0.000219	GHR/CNR1/TACR1/RGS4/PCSK1/PENK
BP	GO:0050708	regulation of protein secretion	0.000228	CHGA/SSTR5/NNAT/IGF1/FGA/SFRP1/MYOM1/SYT4/CARTPT/UCN3/CNR1/GIP/ANKRD1/KCNA5/FGG/ADCY5/F2/GCG/PCSK1/TGFB3/NEUROD1
BP	GO:1904705	regulation of vascular associated	0.000229	MYOCD/FGF2/IGF1/TPM1/ADIPOQ/PRKG1/CNN1/NR4A3/AGT/TGFB3/DDR2
BP	GO:0019933	cAMP-mediated signaling	0.000231	ADCY1/PDE3A/PDE7B/GIP/SCT/AKAP6/ADCYAP1/CAP2
BP	GO:0051496	positive regulation of	0.000231	FERMT2/TPM1/APOA1/SFRP1/TACR1/SERPINF2/MYOC/TGFB3
BP	GO:0072028	nephron morphogenesis	0.000233	TMEM59L/GLI3/FGF2/GPC3/AGT/NOG/SIX2/GREB1L/ADAMTS16/GREM1
BP	GO:0051047	positive regulation of secretion	0.000247	NNAT/IGF1/PLA2G3/FGA/MYOM1/SYT4/CARTPT/UCN3/TACR1/GIP/ANKRD1/VIP/AGT/GRP/FGG/SCT/TACR2/F2/GCG/PCSK1/ADCYAP1/CXCL12/TGFB3

BP	GO:0048771	tissue remodeling	0.000248	THBS4/PTH1R/PTN/NPR3/SFRP1/FGF10/CARTPT/RSPO3/PDK4/LEPR/AGT/FLNA/TF/HAND2/DDR2/GREM1
BP	GO:0099068	postsynapse assembly	0.00025	PTPRD/CDH2/NRXN2/NLGN4X/GAP43/NPTX1/NLGN4Y
BP	GO:0007044	cell-substrate junction	0.000252	FERMT2/ACTN2/SFRP1/PHLDB2/GPM6B/APOD/MYOC/STON1/EPHA3/GREM1/SORBS1
BP	GO:0043502	regulation of muscle	0.000252	IGF1/PI16/PDE9A/NR4A3/AGT/RGS4/SGCA/AKAP6/MIR145/HAND2/FBXO32
BP	GO:0050673	epithelial cell proliferation	0.000252	THBS4/HTR2B/COL8A1/FGF2/IGF1/LIMS2/PTN/RETNLB/VSTM4/NGFR/APOA1/SFRP1/ALDH1A2/FGF10/RGN/FGF7/GPC3/PRKD1/SFRP2/AR/TACR1/NR4A3/VIP/NOG/ZEB1/IGF2/FGL1/NKX2-5/AGTR1/CXCL12/PTPRN
BP	GO:0007272	ensheathment of neurons	0.000259	MPZ/CNTNAP1/JAM3/JAM2/PTN/ANK2/LGI4/NFASC/WASF3/GPM6B/CNTN1/SOX10/MYOC/CLDN11
BP	GO:0008366	axon ensheathment	0.000259	MPZ/CNTNAP1/JAM3/JAM2/PTN/ANK2/LGI4/NFASC/WASF3/GPM6B/CNTN1/SOX10/MYOC/CLDN11
BP	GO:0062013	positive regulation of small molecule	0.000259	GPT/PTH1R/IGF1/HTR2A/PLA2G3/ADIPOQ/APOA1/RGN/CES1/NR4A3/IGF2/GCG/PRKAA2/SORBS1
BP	GO:0072073	kidney epithelium development	0.000259	TMEM59L/GLI3/FGF2/ADIPOQ/SFRP1/GPC3/AGT/NOG/SIX2/GREB1L/ADAMTS16/SLIT2/EPHA7/GREM1
BP	GO:0050767	regulation of neurogenesis	0.000264	DLL3/RELN/GLI3/FGF2/PTN/PTPRD/CDON/NTN1/SYT4/SEMA3E/NFATC4/NOG/DIXDC1/RND2/MAP6/MAP1B/PLXNA4/F2/NGF/SOX10/SLIT2/DAAM2/CXCL12/ASCL1/IL33/EPHA7
BP	GO:0032412	regulation of ion transmembrane transporter	0.000264	KCNE4/RELN/PLN/CACNG4/KCNK3/ACTN2/ATP1B2/FHL1/FXYD6/ATP1A2/RGN/ANK2/ABCC9/CASQ2/DMD/GRP/JPH2/CACNB2/AKAP6/SCN4B/HSPA2
BP	GO:0071625	vocalization	0.000267	GLI3/NRXN2/NLGN4X/NLGN4Y/NRXN3
BP	GO:0086014	atrial cardiac muscle cell	0.000267	ANK2/KCNA5/FLNA/CACNB2/CACNA1C
BP	GO:0086026	atrial cardiac muscle cell to	0.000267	ANK2/KCNA5/FLNA/CACNB2/CACNA1C
BP	GO:0086066	atrial cardiac muscle cell to	0.000267	ANK2/KCNA5/FLNA/CACNB2/CACNA1C
BP	GO:0006910	phagocytosis, recognition	0.000269	COLEC12/TUB/PLA2G5/COLEC11/CD36/PTX3
BP	GO:0099625	ventricular cardiac muscle	0.000269	KCNE4/KCNJ8/ANK2/KCNH6/SCN4B/KCNH2
BP	GO:0032414	positive regulation of ion	0.000275	RELN/CACNG4/KCNK3/ACTN2/ATP1B2/RGN/ANK2/DMD/JPH2/CACNB2/AKAP6/HSPA2

BP	GO:0071692	protein localization to extracellular region	0.000275	CHGA/SSTR5/NNAT/IGF1/ARL4D/FGA/SFRP1/MYOM1/SYT4/CARTPT/UCN3/CNR1/GIP/ANKRD1/VIP/KCNA5/FGG/ADCY5/TACR2/F2/GCG/PCSK1/ADCYAP1/TGFB3/PTPRN/NEUROD1
BP	GO:0060993	kidney morphogenesis	0.000276	TMEM59L/GLI3/FGF2/FGF10/GPC3/AGT/NOG/SIX2/GREB1L/ADAMTS16/GREM1
BP	GO:1990874	vascular associated	0.000276	MYOCD/FGF2/IGF1/TPM1/ADIPOQ/PRKG1/CNN1/NR4A3/AGT/TGFB3/DDR2
BP	GO:0061572	actin filament bundle organization	0.000283	FERMT2/SYNPO2/TPM1/CALD1/APOA1/DPYSL3/SFRP1/PHLDB2/ELN/TACR1/FLNA/SERPINF2/MYOC/TGFB3/SORBS1
BP	GO:0070944	neutrophil-mediated killing	0.000289	CTSG/ELANE/AZU1/F2
BP	GO:0055021	regulation of cardiac muscle	0.000308	FGF2/IGF1/ZFPM2/PI16/NOG/RGS4/KCNK2/NKX2-5/AKAP6
BP	GO:0014068	positive regulation of phosphatidylinos	0.000317	ROR2/RELN/FGF2/IGF1/SEMA3E/AGT/DCN/F2/MYOC/DDR2
BP	GO:0032409	regulation of transporter activity	0.000337	KCNE4/RELN/PLN/CACNG4/KCNK3/ACTN2/ADIPOQ/ATP1B2/FHL1/FXYD6/ATP1A2/RGN/ANK2/ABCC9/APOA2/CASQ2/DMD/GRP/JPH2/CACNB2/AKAP6/SCN4B/HSPA2
BP	GO:0070296	sarcoplasmic reticulum	0.000342	PLN/ATP1A2/ANK2/CASQ2/DMD/AKAP6/CACNA1C
BP	GO:0010611	regulation of cardiac muscle	0.000345	IGF1/PI16/PDE9A/NR4A3/AGT/RGS4/AKAP6/MIR145/HAND2
BP	GO:0061326	renal tubule development	0.00036	TMEM59L/GLI3/FGF2/GPC3/PRICKLE1/AGT/NOG/SIX2/GREB1L/ADAMTS16/GREM1
BP	GO:0007568	aging	0.000367	NPY/IGFBP1/FGF2/GFRA1/HTR2A/AMH/RGN/CNR1/AGT/CRYAB/KCNMB1/MPO/APOD/TGFB3/PENK
BP	GO:0030514	negative regulation of	0.000384	SFRP1/RBPMS2/CHRDL2/SFRP2/NOG/GREM2/CHRD/GREM1/CHRDL1
BP	GO:0051924	regulation of calcium ion transport	0.00039	PLN/ATP1A2/RGN/ANK2/PRKD1/CASQ2/AGT/DMD/RGS4/JPH2/NPSR1/CACNB2/F2/AKAP6/GCG/TRPC1/MYLK/CXCL12/HSPA2/CACNA1C
BP	GO:0007187	G protein-coupled receptor signaling pathway, coupled	0.00039	HTR2B/PTH1R/NPY/SSTR5/HTR2A/CNR1/AGT/CHRM2
BP	GO:0050764	regulation of phagocytosis	0.000393	PRTN3/ADIPOQ/APOA1/TUB/AHSG/APOA2/PLA2G5/AZU1/COLEC11/CD36/PTX3
BP	GO:0086011	membrane repolarization	0.000394	KCNE4/KCNJ8/KCNH6/KCNA5/FLNA/KCNH2
BP	GO:0010463	mesenchymal cell proliferation	0.000398	FOXF1/FGF7/GPC3/ZEB1/SIX2/HAND2/CHRD
BP	GO:0051954	positive regulation of	0.000398	SYT4/CARTPT/SLC38A3/VIP/AGT/TACR2/CXCL12

BP	GO:0030510	regulation of BMP signaling pathway	0.00041	RGMA/SFRP1/RBPMS2/GPC3/CHRDL2/SFRP2/NOG/SFRP4/GREM2/CHRD/GREM1/CHRDL1
BP	GO:0070943	neutrophil-mediated killing	0.000422	CTSG/ELANE/AZU1/F2
BP	GO:0034103	regulation of tissue	0.000423	THBS4/SFRP1/CARTPT/PDK4/LEPR/AGT/TF/HAND2/DDR2/GREM1
BP	GO:0031099	regeneration	0.000427	IGFBP1/IGF1/PTN/FGF10/TNC/RGN/APOA2/GAP43/NR4A3/F7/FLNA/SGCA/MAP1B/APOD/CXCL12/VTN
BP	GO:0014897	striated muscle hypertrophy	0.000428	HTR2B/IGF1/PI16/PDE9A/NR4A3/AGT/RGS4/AKAP6/MYOC/MIR145/HAND2
BP	GO:0046620	regulation of organ growth	0.000428	FGF2/IGF1/ZFPM2/PI16/NOG/RGS4/MAEL/IGF2/KCNK2/NKX2-5/AKAP6
BP	GO:0009952	anterior/posterior pattern specification	0.000428	DLL3/BARX1/GLI3/CDON/SFRP1/ALDH1A2/FOXF1/HOXD10/GPC3/SFRP2/PRICKLE1/NOG/ZBTB16/MEOX2/SIX2/HOXA4/NEUROD1
BP	GO:0050433	regulation of catecholamine	0.000442	CHGA/HTR2A/SYT4/CARTPT/CNR1/VIP/AGT/CXCL12
BP	GO:0001837	epithelial to mesenchymal transition	0.000442	FERMT2/TGFB1I1/IGF1/SFRP1/TMEM100/PHLDB2/SFRP2/NOG/FLNA/DACT3/TNXB/MIR145/EPHA3/TGFB3/GREM1
BP	GO:0048015	phosphatidylinositol-mediated signaling	0.000442	ROR2/HTR2B/RELN/FGF2/IGF1/PLCH2/HTR2A/MUC5AC/NPR3/SEMA3E/AGT/DCN/F2/MYOC/DDR2
BP	GO:0007413	axonal	0.000443	CNTN4/CNR1/NDN/CRTAC1/EPHA3
BP	GO:0098962	regulation of postsynaptic neurotransmitter	0.000443	CACNG4/NRXN2/NPTX2/NPTXR/NPTX1
BP	GO:0106030	neuron	0.000443	CNTN4/CNR1/NDN/CRTAC1/EPHA3
BP	GO:0022898	regulation of transmembrane transporter activity	0.000444	KCNE4/RELN/PLN/CACNG4/KCNK3/ACTN2/ATP1B2/FHL1/FXYD6/ATP1A2/RGN/ANK2/ABCC9/CASQ2/DMD/GRP/JPH2/CACNB2/AKAP6/SCN4B/HSPA2
BP	GO:0010828	positive regulation of	0.00046	IGF1/ADIPOQ/CLIP3/GPC3/GIP/NR4A3/SORBS1
BP	GO:0045214	sarcomere organization	0.00046	TPM1/ACTN2/FLNC/CFL2/CSRP1/ANKRD1/LDB3
BP	GO:0099084	postsynaptic specialization	0.00046	CNTNAP1/RELN/PTPRD/CDH2/NRXN2/GAP43/NPTX1
BP	GO:1900026	positive regulation of substrate	0.00046	FERMT2/LIMS2/FGA/APOA1/FLNA/FGG/MYOC
BP	GO:0010232	vascular transport	0.000464	SLC8A2/KCNJ8/ATP1B2/SLC2A4/ATP1A2/LEPR/SLC38A3/ABCC9/SLC7A2/CD36
BP	GO:0033627	cell adhesion mediated by	0.000464	JAM3/ITGA7/MMRN1/ITGBL1/ITGA8/SFRP2/CCL21/CYP1B1/ITGA9/VTN
BP	GO:0150104	transport across blood-brain	0.000464	SLC8A2/KCNJ8/ATP1B2/SLC2A4/ATP1A2/LEPR/SLC38A3/ABCC9/SLC7A2/CD36

BP	GO:0019233	sensory perception of	0.000465	HTR2A/TAC1/TMEM100/CNR1/NDN/TACR1/GIP/VIP/CXCL12/PENK/IL33
BP	GO:0014743	regulation of muscle	0.000475	IGF1/PI16/PDE9A/NR4A3/AGT/RGS4/AKAP6/MIR145/HAND2
BP	GO:0032355	response to estradiol	0.000478	SFRP1/ALDH1A2/FGF10/TACR1/APOB/AGT/F7/CRYAB/PTGFR/MAP1B/CYP1B1/PENK
BP	GO:0048732	gland development	0.000499	CDO1/GLI3/FGF2/LIMS2/PTN/NTN1/CPS1/APOA1/SFRP1/ALDH1A2/FOXF1/FGF10/TNC/RGN/FGF7/AR/FRZB/NOG/IGF2/FGL1/NKX2-5/CYP1B1/SOX10/HAND2/PCSK1/ASCL1/TGFB3/VTN
BP	GO:0014896	muscle hypertrophy	0.000506	HTR2B/IGF1/PI16/PDE9A/NR4A3/AGT/RGS4/AKAP6/MYOC/MIR145/HAND2
BP	GO:0050769	positive regulation of neurogenesis	0.000518	RELN/GLI3/FGF2/PTN/PTPRD/CDON/NTN1/DIXDC1/RND2/MAP6/MAP1B/PLXNA4/NGF/SOX10/SLIT2/CXCL12/ASCL1/IL33
BP	GO:0050766	positive regulation of	0.000527	APOA1/TUB/AHSG/APOA2/PLA2G5/AZU1/COLEC11/CD36/PTX3
BP	GO:0061035	regulation of cartilage development	0.000527	GLI3/NKX3-2/FRZB/NOG/EFEMP1/ZBTB16/SIX2/WNT9A/GREM1
BP	GO:0070527	platelet aggregation	0.000527	MMRN1/FGA/PRKG1/CTSG/CSRP1/FLNA/FGL1/FGG/MYL9
BP	GO:0048562	embryonic organ morphogenesis	0.000556	GLI3/NTN1/FOXF1/RBPMS2/FGF10/NKX3-2/HOXD10/ITGA8/FRZB/NOG/EFEMP1/ZEB1/ASB2/MFAP5/COL2A1/SIX2/NKX2-5/HAND2/HOXA4/TGFB3/NEUROD1
BP	GO:0060191	regulation of lipase activity	0.000557	HTR2B/FGF2/HTR2A/PNLIP/GPIHBP1/APOC3/APOA2/PLA2G5/AGT/AGTR1
BP	GO:0086012	membrane depolarization during cardiac	0.000558	ATP1A2/ANK2/CACNB2/SCN4B/CACNA1C
BP	GO:0098856	intestinal lipid	0.000558	PNLIP/APOA1/APOA2/SOAT2/CD36
BP	GO:0050432	catecholamine secretion	0.00056	CHGA/HTR2A/SYT4/CARTPT/CNR1/VIP/AGT/CXCL12
BP	GO:0071711	basement membrane	0.000561	CMA1/PHLDB2/PRICKLE1/LAMA2/FLRT2/HMCN1
BP	GO:1901380	negative regulation of	0.000561	KCNE4/ACTN2/CASQ2/GRP/RGS4/KCNH2
BP	GO:0001764	neuron migration	0.000564	RELN/NTN1/PRKG1/NDN/DNER/SEMA3E/GFRA3/CCKAR/MAP1B/CHL1/FLRT2/TNN/CXCL12/ASCL1/TUBA1A
BP	GO:0060420	regulation of heart growth	0.000583	FGF2/IGF1/ZFPM2/PI16/NOG/RGS4/KCNK2/NKX2-5/AKAP6
BP	GO:0022612	gland morphogenesis	0.000598	GLI3/LIMS2/PTN/NTN1/SFRP1/FGF10/TNC/FGF7/AR/NOG/FGL1/TGFB3

BP	GO:0007369	gastrulation	0.000601	COL8A1/ITGA7/ADIPOQ/APOA1/SFRP1/FOXF1/PHLDB2/ITGA8/GPC3/SFRP2/PRICKLE1/NR4A3/NOG/SIX2/MIR145/VTN
BP	GO:0015850	organic hydroxy compound transport	0.000607	CHGA/SLCO1B3/HTR2A/PNLIP/ADIPOQ/APOA1/ABCA8/SYT4/CARTPT/APOC3/CNR1/APOA2/CES1/APOB/VIP/AGT/GPM6B/SOAT2/AGTR1/CXCL12/CD36
BP	GO:0050829	defense response to Gram-negative	0.00061	CHGA/SSC5D/DEFA6/CTSG/DMBT1/PRKD1/ELANE/AZU1/F2/LYPD8
BP	GO:0051899	membrane depolarization	0.000644	FHL1/ATP1A2/ANK2/DCN/CACNB2/MYOC/SCN4B/KCNH2/CACNA1C
BP	GO:0051384	response to glucocorticoid	0.000662	CDO1/SSTR5/GHR/CPS1/ADIPOQ/SLIT3/UCN3/APOA2/BCHE/CYP1B1/PCSK1/SLIT2/FBXO32
BP	GO:0034383	low-density lipoprotein	0.000664	CES3/ADIPOQ/APOC3/APOB/SOAT2/CD36
BP	GO:0021700	developmental maturation	0.000665	CNTNAP1/SEZ6/PTH1R/RELN/IGF1/PDE3A/PLA2G3/ALDH1A2/NFATC4/CCL21/LGI4/ZBTB16/FGG/MAP1B/SOX10/MYOC/RECK/FEV/ASCL1/PTPRN/GREM1/CHRDL1
BP	GO:0032231	regulation of actin filament bundle assembly	0.000697	FERMT2/SYNPO2/TPM1/APOA1/SFRP1/PHLDB2/TACR1/FLNA/SERPINF2/MYOC/TGFB3
BP	GO:0045668	negative regulation of	0.000704	SFRP1/NOG/TWIST2/HAND2/TNN/CHRD/GDF10/GREM1
BP	GO:1900024	regulation of substrate adhesion-	0.000704	FERMT2/LIMS2/FBLN1/FGA/APOA1/FLNA/FGG/MYOC
BP	GO:0050772	positive regulation of	0.000711	NTN1/DIXDC1/RND2/MAP6/MAP1B/PLXNA4/NGF/SLIT2/CXCL12
BP	GO:0055017	cardiac muscle tissue growth	0.000726	FGF2/IGF1/ZFPM2/PI16/AGT/NOG/RGS4/KCNK2/NKX2-5/AKAP6
BP	GO:0030038	contractile actin filament bundle assembly	0.000754	FERMT2/TPM1/APOA1/SFRP1/PHLDB2/ELN/TACR1/SERPINF2/MYOC/TGFB3/SORBS1
BP	GO:0043149	stress fiber assembly	0.000754	FERMT2/TPM1/APOA1/SFRP1/PHLDB2/ELN/TACR1/SERPINF2/MYOC/TGFB3/SORBS1
BP	GO:0060840	artery development	0.000754	MYOCD/GLI3/NGFR/FOXF1/FKBP10/PRICKLE1/APOB/NOG/MIR145/HAND2/MYLK
BP	GO:0050731	positive regulation of peptidyl-	0.000754	THBS4/RELN/GHR/IGF1/GFRA1/HTR2A/ADIPOQ/FGF10/FGF7/AGT/IGF2/CNTN1/CD36/GREM1/VTN
BP	GO:0042552	myelination	0.000755	MPZ/CNTNAP1/JAM3/JAM2/PTN/ANK2/LGI4/NFASC/WASF3/GPM6B/CNTN1/SOX10/MYOC

BP	GO:0048568	embryonic organ development	0.000757	GLI3/NTN1/ZFPM2/ALDH1A2/FOXF1/RBPMS2/FGF10/NKX3-2/HOXD10/ITGA8/RSPO3/PRICKLE1/FRZB/NOG/EFEMP1/ZEB1/IGF2/ASB2/MFAP5/SCT/COL2A1/SIX2/NKX2-5/PLXNA4/HAND2/HOXA4/TGFB3/NEUROD1
BP	GO:0045777	positive regulation of	0.000779	TPM1/CARTPT/CNR1/TACR1/TAC3/AGT
BP	GO:0070528	protein kinase C signaling	0.000779	SEZ6/HTR2B/AKAP12/ANKRD1/DGKG/AZU1
BP	GO:0045165	cell fate commitment	0.00078	PTCH2/TGFB1I1/GLI3/FGF2/EBF2/CDON/SFRP1/GAS1/FGF10/HOXD10/SFRP2/AR/GAP43/SIX2/NKX2-5/WNT9A/TNXB/FEV/ASCL1/NEUROD1
BP	GO:0003044	regulation of systemic arterial blood	0.000792	TPM1/CMA1/CTSG/TACR1/AGT/SERPINF2/AGTR1
BP	GO:0042311	vasodilation	0.000792	VSTM4/CPS1/NPR3/PRKG1/AGT/KCNMB1/GUCY1A1
BP	GO:0060976	coronary vasculature	0.000792	MYOCD/FGF2/GPC3/PRICKLE1/MIR145/HAND2/SGCD
BP	GO:0070942	neutrophil	0.000811	CTSG/ELANE/AZU1/F2
BP	GO:0060307	regulation of ventricular cardiac muscle	0.000855	KCNE4/ANK2/KCNH6/SCN4B/KCNH2
BP	GO:0030177	positive regulation of Wnt signaling	0.000859	FGF2/SFRP1/FGF10/RSPO3/GPC3/SFRP2/DIXDC1/SFRP4/RSPO2/MIR145/RECK/RSPO1/DAAM2
BP	GO:0045598	regulation of fat cell differentiation	0.000859	FERMT2/TGFB1I1/FNDC5/HTR2A/ZFPM2/LMO3/ADIPOQ/SFRP1/SFRP2/FRZB/ZBTB16/MEDAG/RUNX1T1
BP	GO:0061045	negative regulation of	0.00086	FGF2/MMRN1/FGA/PRKG1/PHLDB2/FGG/SERPINF2/F2/VTN
BP	GO:0021543	pallium development	0.000893	RELN/NPY/GLI3/FOXP2/CDON/CDH2/IGF2BP1/DIXDC1/SCT/EMX1/DCLK2/SLIT2/ASCL1/TUBA1A/NEUROD1
BP	GO:0043114	regulation of vascular	0.000899	FERMT2/PDE3A/AKAP12/TACR1/AZU1/TACR2/SLIT2
BP	GO:1904752	regulation of vascular associated	0.000899	MYOCD/TPM1/ADIPOQ/PRKG1/NR4A3/AGT/DDR2
BP	GO:0010613	positive regulation of	0.00091	IGF1/PDE9A/NR4A3/AGT/AKAP6/HAND2
BP	GO:0051928	positive regulation of	0.000915	RGN/ANK2/JPH2/NPSR1/CACNB2/F2/AKAP6/GCG/TRPC1/MYLK/CXCL12/HSPA2
BP	GO:0003205	cardiac chamber development	0.000943	MYOCD/CPE/TPM1/ZFPM2/FOXF1/ANK2/SLIT3/SFRP2/NOG/KCNK2/NKX2-5/GREB1L/HAND2/SLIT2
BP	GO:0045913	positive regulation of	0.000943	GPT/PTH1R/IGF1/HTR2A/RGN/IGF2/GCG/PRKAA2/SORBS1

BP	GO:0007229	integrin-mediated	0.000946	FERMT2/ITGA7/LIMS2/PTN/APOA1/ITGBL1/ITGA8/PRKD1/FLNA/LAMA2/ITGA9
BP	GO:0048638	regulation of developmental growth	0.000958	FGF2/GHR/IGF1/NTN1/ZFPM2/PI16/SYT4/AR/SEMA3E/NOG/RGS4/MAEL/RND2/IGF2/KCNK2/MAP1B/NKX2-5/PLXNA4/AKAP6/NGF/CXCL12/EPHA7
BP	GO:0051893	regulation of focal adhesion	0.000972	FERMT2/SFRP1/PHLDB2/GPM6B/APOD/MYOC/EPHA3/GREM1
BP	GO:0090109	regulation of cell-substrate	0.000972	FERMT2/SFRP1/PHLDB2/GPM6B/APOD/MYOC/EPHA3/GREM1
BP	GO:1903169	regulation of calcium ion transmembrane	0.000997	PLN/ATP1A2/RGN/ANK2/PRKD1/CASQ2/DMD/JPH2/NPSR1/CACNB2/F2/AKAP6/TRPC1/HSPA2/CACNA1C
BP	GO:0072080	nephron tubule development	0.001013	TMEM59L/GLI3/FGF2/GPC3/AGT/NOG/SIX2/GREB1L/ADAMTS16/GREM1
BP	GO:0010226	response to	0.001041	ADCY1/FABP4/ACTA1/NFATC4/ASCL1
BP	GO:0032331	negative regulation of	0.001041	GLI3/NKX3-2/EFEMP1/WNT9A/GREM1
BP	GO:0007616	long-term memory	0.001057	RELN/ADCY1/SLC2A4/LRRN4/TACR1/NFATC4
BP	GO:0014742	positive regulation of	0.001057	IGF1/PDE9A/NR4A3/AGT/AKAP6/HAND2
BP	GO:0016486	peptide hormone	0.001057	CPE/CMA1/CTSG/SCG5/BCHE/PCSK1
BP	GO:0046461	neutral lipid catabolic	0.001057	CPS1/GPIHBP1/APOC3/APOA2/APOB/PNLIPRP2
BP	GO:0046464	acylglycerol catabolic	0.001057	CPS1/GPIHBP1/APOC3/APOA2/APOB/PNLIPRP2
BP	GO:0048863	stem cell differentiation	0.001072	HTR2B/MYOCD/FGF2/PTN/CDH2/SFRP1/ALDH1A2/SFRP2/PRICKLE1/SEMA3E/FRZB/NOG/RBM24/NKX2-5/TRIM6/SOX10/HAND2/GREM1
BP	GO:0062012	regulation of small molecule metabolic process	0.001078	GPT/PTH1R/IGF1/HTR2A/PLA2G3/ADIPOQ/APOA1/APOC3/RGN/PDK4/LEPR/CNR1/CES1/GIP/APOB/NR4A3/IGF2/GCG/AQP8/FMO1/PRKAA2/SORBS1
BP	GO:0033689	negative regulation of	0.001078	MN1/SFRP1/TNN/GREM1
BP	GO:0050678	regulation of epithelial cell proliferation	0.00109	THBS4/HTR2B/FGF2/IGF1/LIMS2/PTN/NGFR/SFRP1/ALDH1A2/FGF10/RGN/FGF7/GPC3/PRKD1/SFRP2/AR/TACR1/NR4A3/VIP/NOG/ZEB1/IGF2/NKX2-5/AGTR1/CXCL12/PTPRN
BP	GO:0031016	pancreas development	0.00113	CDH2/ALDH1A2/FOXF1/FGF10/NKX3-2/GIP/IGF2/PCSK1/NEUROD1
BP	GO:1904738	vascular associated	0.001146	MYOCD/TPM1/ADIPOQ/PRKG1/NR4A3/AGT/DDR2
BP	GO:0043279	response to alkaloid	0.001188	CACNG4/GHR/HTR2A/CNR1/BCHE/TACR1/CASQ2/RGS4/PCSK1/PENK

BP	GO:0021537	telencephalon development	0.001193	RELN/NPY/GLI3/FOXP2/CDON/CDH2/ATP1B2/ATP1A2/IGF2BP1/DIXDC1/SCT/EMX1/PLXNA4/DCLK2/SLIT2/CXCL12/ASCL1/TUBA1A/NEUROD1
BP	GO:0048705	skeletal system morphogenesis	0.001194	FREM1/PAPPA2/GLI3/GHR/SFRP1/NKX3-2/HOXD10/RAB23/NOG/ZEB1/COL2A1/SIX2/SFRP4/HOXA4/MGP/TGFB3/GREM1
BP	GO:0097106	postsynaptic density	0.001222	CNTNAP1/RELN/PTPRD/CDH2/NRXN2/NPTX1
BP	GO:0034377	plasma lipoprotein	0.001255	APOA1/APOC3/APOA2/APOB/SOAT2
BP	GO:0060314	regulation of ryanodine-sensitive	0.001255	PLN/CASQ2/DMD/JPH2/AKAP6
BP	GO:0060740	prostate gland epithelium	0.001255	SFRP1/FGF10/TNC/AR/NOG
BP	GO:0086013	membrane repolarization during cardiac	0.001255	KCNE4/KCNJ8/KCNA5/FLNA/KCNH2
BP	GO:0072009	nephron epithelium	0.001263	TMEM59L/GLI3/FGF2/ADIPOQ/GPC3/AGT/NOG/SIX2/GREB1L/ADAMTS16/GREM1
BP	GO:0003300	cardiac muscle hypertrophy	0.001285	HTR2B/IGF1/PI16/PDE9A/NR4A3/AGT/RGS4/AKAP6/MIR145/HAND2
BP	GO:0022602	ovulation cycle process	0.001288	AMH/SLIT3/AFP/SLIT2/TGFB3/PTPRN/PTX3
BP	GO:0001666	response to hypoxia	0.001299	MYOCD/KCNK3/ADIPOQ/SFRP1/SLC2A4/HIF3A/UCN3/CA9/KCNA5/F7/CRYAB/KCNMB1/KCNK2/KCNMA1/MIR145/PTGIS/CXCL12/TGFB3/PENK/DDR2
BP	GO:0060193	positive regulation of	0.001318	HTR2B/FGF2/HTR2A/PNLIP/GPIHBP1/PLA2G5/AGT/AGTR1
BP	GO:0034446	substrate adhesion-	0.001387	FERMT2/LIMS2/FBLN1/BVES/FGA/APOA1/ITGA8/FLNA/FGG/MYOC
BP	GO:0060419	heart growth	0.001387	FGF2/IGF1/ZFPM2/PI16/AGT/NOG/RGS4/KCNK2/NKX2-5/AKAP6
BP	GO:1901019	regulation of calcium ion transmembrane	0.001387	PLN/ATP1A2/RGN/ANK2/CASQ2/DMD/JPH2/CACNB2/AKAP6/HSPA2
BP	GO:0034375	high-density lipoprotein	0.001402	PLA2G3/APOA1/APOC3/APOA2
BP	GO:2000095	regulation of Wnt signaling pathway, planar	0.001402	SFRP1/RSPO3/GPC3/SFRP2
BP	GO:0001990	regulation of systemic	0.001406	CMA1/CTSG/TACR1/AGT/SERPINF2/AGTR1
BP	GO:0007588	excretion	0.001406	CHRNA3/TACR1/AGT/NPSR1/KCNMA1/AGTR1
BP	GO:0060071	Wnt signaling pathway, planar	0.001444	ROR2/SFRP1/RSPO3/GPC3/SFRP2/PRICKLE1/PRICKLE2

BP	GO:0048545	response to steroid hormone	0.001463	CDO1/SSTR5/GHR/CPS1/LMO3/ADIPOQ/SFRP1/ATP1A2/SLIT3/UCN3/APOA2/AR/BCHE/ACTA1/TACR1/NR4A3/CYP1B1/SOX10/PCSK1/SLIT2/FBXO32/TGFB3
BP	GO:0019433	triglyceride	0.0015	CPS1/GPIHBP1/APOC3/APOB/PNLIPRP2
BP	GO:0042730	fibrinolysis	0.0015	FGA/FGG/SERPINF2/F2/VTN
BP	GO:0048520	positive	0.0015	NPY/GRPR/NR4A3/GRP/PENK
BP	GO:0060259	regulation of	0.0015	NPY/LEPR/CNR1/NR4A3/NPSR1
BP	GO:0045216	cell-cell junction organization	0.001528	CNTNAP1/FERMT2/CDH19/JAM3/LIMS2/PRTN3/CDH2/MPDZ/ANK2/NLGN4X/CLDN6/AGT/FLNA/SVEP1/CLDN11/TGFB3
BP	GO:0050730	regulation of peptidyl-tyrosine	0.001535	THBS4/RELN/GHR/IGF1/GFRA1/HTR2A/ADIPOQ/SFRP1/FGF10/FGF7/SFRP2/AGT/IGF2/CNTN1/CD36/EPHA7/GREM1/VTN
BP	GO:0051258	protein polymerization	0.00154	TUBB4A/SVIL/KANK4/FGA/CLIP3/LMOD1/ELN/TMOD1/CCL21/CASQ2/FGG/MAP1B/WASF3/STMN2/TRIM6/SLIT2/DAAM2/TUBA1A/VTN
BP	GO:0035270	endocrine system development	0.001545	FGF2/CDH2/APOA1/ALDH1A2/FGF10/GIP/NOG/NKX2-5/CYP1B1/PCSK1/ASCL1/NEUROD1
BP	GO:0030278	regulation of ossification	0.001554	PTN/SFRP1/AHSG/TACR1/ZBTB16/SIX2/GPM6B/MGP/GDF10/DDR2/GREM1
BP	GO:0007584	response to nutrient	0.001578	MN1/ADIPOQ/SFRP1/ALDH1A2/CNR1/SFRP2/BCHE/F7/MAP1B/CYP1B1/CYP24A1/ASCL1/PENK
BP	GO:0150116	regulation of cell-substrate	0.001598	FERMT2/SFRP1/PHLDB2/GPM6B/APOD/MYOC/EPHA3/GREM1
BP	GO:0010762	regulation of fibroblast	0.00161	FGF2/ACTA2/HAS1/AKAP12/MIR145/DDR2
BP	GO:0035136	forelimb morphogenesis	0.00161	ALDH1A2/HOXD10/ZBTB16/RSPO2/RECK/CACNA1C
BP	GO:0140448	signaling receptor ligand	0.00161	CPE/CMA1/CTSG/SCG5/BCHE/PCSK1
BP	GO:0032330	regulation of chondrocyte	0.001614	GLI3/NKX3-2/EFEMP1/ZBTB16/SIX2/WNT9A/GREM1
BP	GO:0031667	response to nutrient levels	0.00164	GPT/MN1/NPY/GHR/CPS1/ADIPOQ/SFRP1/ALDH1A2/CARTPT/PDK4/PRKD1/SLC38A3/UCN3/CNR1/SFRP2/BCHE/GIP/ZEB1/F7/MPO/MAP1B/SCT/CYP1B1/PCSK1/CYP24A1/PRKAA2/ASCL1/PENK
BP	GO:0046887	positive regulation of	0.001644	NNAT/PLA2G3/FGA/UCN3/TACR1/GIP/GRP/FGG/SCT/F2/GCG/ADCYAP1
BP	GO:0008306	associative learning	0.00173	RELN/ATP1A2/LRRN4/TACR1/MAP1A/AGT/NOG/NPTX2/TUBA1A
BP	GO:0015844	monoamine transport	0.00173	CHGA/HTR2A/SYT4/CARTPT/CNR1/VIP/AGT/GPM6B/CXCL12
BP	GO:0030193	regulation of blood	0.001754	FGA/PRKG1/F7/FGG/SERPINF2/F2/CD36/VTN

BP	GO:0030073	insulin secretion	0.001771	CHGA/SSTR5/NNAT/SFRP1/CARTPT/UCN3/CNR1/GIP/KCNA5/ADCY5/F2/GCG/ADCYAP1/PTPRN/NEUROD1
BP	GO:0030194	positive regulation of	0.001778	F7/SERPINF2/F2/CD36/VTN
BP	GO:0060512	prostate gland	0.001778	SFRP1/FGF10/TNC/AR/NOG
BP	GO:1900048	positive regulation of	0.001778	F7/SERPINF2/F2/CD36/VTN
BP	GO:1903779	regulation of	0.001778	SLC8A2/ANK2/AGT/NKX2-5/TRPC1
BP	GO:0010763	positive regulation of	0.001787	ACTA2/AKAP12/MIR145/DDR2
BP	GO:0099509	regulation of presynaptic cytosolic	0.001787	NPY/CNR1/SCGN/CACNB2
BP	GO:0010761	fibroblast migration	0.001799	FGF2/ACTA2/HAS1/AKAP12/TNS1/MIR145/DDR2
BP	GO:0051962	positive regulation of nervous system development	0.001816	RELN/GLI3/FGF2/PTN/PTPRD/CDON/NTN1/DIXDC1/RND2/MAP6/MAP1B/PLXNA4/NGF/SOX10/FLRT2/SLIT2/CXCL12/ASCL1/IL33
BP	GO:0010038	response to metal ion	0.001818	CACNA1H/KCNK3/ADCY1/CPS1/FGA/CYBRD1/FABP4/SYT4/ACTA1/NFATC4/CASQ2/KCNMB1/TF/NPTX1/FGG/KCNMA1/TRPC1/PCSK1/PRKAA2/ASCL1/TUBA1A/PENK/CPA1
BP	GO:0051346	negative regulation of hydrolase activity	0.001825	TFPI2/SLCO1B3/ITIH5/APOA1/PI16/APOC3/RGN/LEPR/GPC3/AHSG/APOA2/SFRP2/SERPINA6/ITIH2/CRYAB/SERPINF2/SERPIND1/NGF/RECK/PI15/PTX3/VTN
BP	GO:0003208	cardiac ventricle	0.001923	CPE/TPM1/ZFPM2/FOXF1/SFRP2/NOG/NKX2-5/HAND2
BP	GO:0033344	cholesterol efflux	0.001923	ADIPOQ/APOA1/ABCA8/APOC3/APOA2/CES1/APOB/SOAT2
BP	GO:0045600	positive regulation of	0.001923	FNDC5/HTR2A/LMO3/SFRP1/SFRP2/FRZB/ZBTB16/MEDAG
BP	GO:0033273	response to vitamin	0.002031	MN1/SFRP1/ALDH1A2/BCHE/F7/MAP1B/CYP24A1/ASCL1/PENK
BP	GO:0051147	regulation of muscle cell differentiation	0.002092	MYOCD/IGF1/PI16/SMYD1/RBPMS2/ZEB1/RGS4/RBM24/IGF2/NKX2-5/AKAP6/MIR145/LAMA2
BP	GO:0035640	exploration	0.002093	ATP1A2/GIP/NOG/CHL1/PENK
BP	GO:0065005	protein-lipid	0.002093	APOA1/APOC3/APOA2/APOB/SOAT2
BP	GO:1901018	positive regulation of potassium ion	0.002093	KCNK3/ACTN2/ATP1B2/ANK2/AKAP6
BP	GO:1904385	cellular	0.002093	ACTN2/AGT/AGTR1/MIR145/DDR2
BP	GO:1900046	regulation of hemostasis	0.002104	FGA/PRKG1/F7/FGG/SERPINF2/F2/CD36/VTN
BP	GO:0043583	ear development	0.002123	GLI3/FGF2/KCNK3/NTN1/FGF10/NKX3-2/ITGA8/PLPPR4/FRZB/NOG/ZEB1/KCNK2/COL2A1/SIX2/TGFB3/NEUROD1

BP	GO:0036293	response to decreased oxygen levels	0.00217	MYOCD/KCNK3/ADIPOQ/SFRP1/SLC2A4/HIF3A/UCN3/CA9/KCNA5/F7/CRYAB/KCNMB1/KCNK2/KCNMA1/MIR145/PTGIS/CXCL12/TGFB3/PENK/DDR2
BP	GO:2000177	regulation of neural precursor	0.002197	GLI3/FGF2/LIMS2/PTN/CDON/CDH2/EMX1/SOX10/ASCL1
BP	GO:0010676	positive regulation of	0.002219	GPT/PTH1R/IGF1/RGN/IGF2/GCG/SORBS1
BP	GO:0072132	mesenchyme morphogenesis	0.002219	ACTG2/FOXF1/TMEM100/ACTA2/ACTA1/NOG/ACTC1
BP	GO:0090175	regulation of establishment of	0.002219	ROR2/SFRP1/RSPO3/GPC3/SFRP2/PRICKLE1/PRICKLE2
BP	GO:0043116	negative regulation of	0.002239	FERMT2/PDE3A/AKAP12/SLIT2
BP	GO:0097094	craniofacial suture	0.002239	FREM1/GLI3/RAB23/TGFB3
BP	GO:0051937	catecholamine transport	0.002298	CHGA/HTR2A/SYT4/CARTPT/CNR1/VIP/AGT/CXCL12
BP	GO:0060415	muscle tissue morphogenesis	0.002298	TPM1/ZFPM2/ANKRD1/NOG/NKX2-5/MIR145/ACTC1/MYLK
BP	GO:0001952	regulation of cell-matrix	0.002303	FERMT2/SFRP1/PHLDB2/SEMA3E/CCL21/GPM6B/APOD/MYOC/EPHA3/CD36/GREM1
BP	GO:0071867	response to monoamine	0.002308	HTR2B/HTR2A/ADIPOQ/NR4A3/FLNA/RGS4/ADCY5/CHRM2/GNAO1/PENK
BP	GO:0071869	response to catecholamine	0.002308	HTR2B/HTR2A/ADIPOQ/NR4A3/FLNA/RGS4/ADCY5/CHRM2/GNAO1/PENK
BP	GO:0090277	positive regulation of	0.002308	NNAT/FGA/UCN3/GIP/GRP/FGG/SCT/F2/GCG/ADCYAP1
BP	GO:0050796	regulation of insulin secretion	0.002333	CHGA/SSTR5/NNAT/SFRP1/CARTPT/UCN3/CNR1/GIP/KCNA5/ADCY5/F2/GCG/NEUROD1
BP	GO:0098657	import into cell	0.002342	CACNA1H/SLC8A2/KCNJ8/SCN7A/CYBRD1/ATP1B2/ATP1A2/PLPPR4/SLC38A3/ABCC9/AGT/RGS4/GPM6B/SLC7A2/AQP8/KCNH2/CACNA1C/CD36
BP	GO:0014065	phosphatidylinositol 3-kinase	0.002354	ROR2/HTR2B/RELN/FGF2/IGF1/HTR2A/SEMA3E/AGT/DCN/F2/MYOC/DDR2
BP	GO:0010907	positive regulation of	0.002357	GPT/IGF1/RGN/IGF2/GCG/SORBS1
BP	GO:0033555	multicellular organismal	0.002374	THBS4/RELN/GRPR/TAC1/IDO1/ATP1A2/TACR1/GRP/PENK
BP	GO:0045907	positive regulation of	0.002446	ADRA1D/HTR2A/FGA/TACR1/FGG
BP	GO:0050820	positive regulation of	0.002446	F7/SERPINF2/F2/CD36/VTN
BP	GO:0021987	cerebral cortex development	0.002452	RELN/NPY/GLI3/FOXP2/CDON/CDH2/DIXDC1/EMX1/SLIT2/ASCL1/TUBA1A
BP	GO:0048008	platelet-derived growth factor	0.002455	MYOCD/ADIPOQ/NR4A3/F7/PDGFRL/APOD/STON1
BP	GO:0045123	cellular extravasation	0.002505	JAM3/JAM2/PRTN3/CCL21/ELANE/AZU1/CXCL12/LYVE1

BP	GO:0051145	smooth muscle cell	0.002505	MYOCD/FOXF1/RBPMS2/FGF10/ITGA8/NFATC4/ZEB1/MIR145
BP	GO:0014902	myotube differentiation	0.002609	CACNA1H/CNTNAP1/MYOCD/JAM2/IGF1/CDON/SMYD1/ACTA1/DNER/RBM24/NKX2-5
BP	GO:0070482	response to oxygen levels	0.002616	MYOCD/KCNK3/ADIPOQ/SFRP1/SLC2A4/HIF3A/UCN3/CA9/KCNA5/F7/CRYAB/KCNMB1/GUCY1A1/KCNK2/KCNMA1/MIR145/PTGIS/CXCL12/TGFB3/PENK/DDR2
BP	GO:0099173	postsynapse organization	0.002628	ROR2/CNTNAP1/RELN/PTPRD/CDH2/NRXN2/NLGN4X/GAP43/INA/GLRB/NPTX1/NLGN4Y/EPHA7/CHRDL1
BP	GO:0050714	positive regulation of	0.00264	NNAT/IGF1/FGA/MYOM1/UCN3/GIP/ANKRD1/FGG/F2/GCG/PCSK1/TGFB3
BP	GO:0001708	cell fate specification	0.002646	PTCH2/GLI3/FGF2/CDON/HOXD10/SFRP2/AR/SIX2/FEV/ASCL1
BP	GO:0002793	positive regulation of	0.002646	NNAT/FGA/UCN3/GIP/GRP/FGG/SCT/F2/GCG/ADCYAP1
BP	GO:0051955	regulation of amino acid	0.002657	SV2A/SYT4/ATP1A2/SLC38A3/AGT/RGS4
BP	GO:0007162	negative regulation of cell adhesion	0.002715	JAM3/CD274/GLI3/FBLN1/ADIPOQ/PRKG1/APOA1/IDO1/PHLDB2/CTSG/PLA2G5/SEMA3E/CCL21/FGL1/FGG/PLXNA4/CYP1B1/APOD/MYOC/CXCL12
BP	GO:0045669	positive regulation of	0.002727	FERMT2/GLI3/FGF2/IGF1/PRKD1/SFRP2/GDF10/DDR2
BP	GO:0050818	regulation of coagulation	0.002727	FGA/PRKG1/F7/FGG/SERPINF2/F2/CD36/VTN
BP	GO:0031960	response to corticosteroid	0.002736	CDO1/SSTR5/GHR/CPS1/ADIPOQ/SLIT3/UCN3/APOA2/BCHE/CYP1B1/PCSK1/SLIT2/FBXO32
BP	GO:0010002	cardioblast	0.002765	MYOCD/PRICKLE1/NKX2-5/GREM1
BP	GO:0061744	motor behavior	0.002765	GRPR/ATP1B2/GRP/TUBA1A
BP	GO:0003298	physiological	0.002841	IGF1/PI16/AGT/RGS4/AKAP6
BP	GO:0003301	physiological cardiac muscle	0.002841	IGF1/PI16/AGT/RGS4/AKAP6
BP	GO:0010667	negative regulation of	0.002841	MYOCD/SFRP2/NKX2-5/MIR145/HAND2
BP	GO:0033688	regulation of osteoblast	0.002841	MN1/NPR3/SFRP1/TNN/GREM1
BP	GO:0061049	cell growth involved in	0.002841	IGF1/PI16/AGT/RGS4/AKAP6
BP	GO:0044703	multi-organism reproductive process	0.002858	PTN/FBLN1/TAC1/IDO1/SLC38A3/CNR1/AR/TACR1/GIP/TAC3/AGT/PTGFR/RECK/ADCYAP1/TGFB3
BP	GO:0001667	ameboidal-type cell migration	0.002868	HTR2B/FGF2/PTN/CDH2/VSTM4/APOA1/ACTA2/HAS1/FGF10/AKAP12/FGF7/GPC3/TNS1/PRKD1/TACR1/SEMA3E/AGT/SMOC2/MEOX2/DCN/CYP1B1/SOX10/MIR145/HAND2/SLIT2/DAAM2/DDR2/GREM1

BP	GO:0043434	response to peptide hormone	0.002942	GPT/CDO1/IGFBP1/GHR/GLP2R/ACTN2/CPS1/ADIPOQ/SLC2A4/PDK4/AHSG/GIP/NR4A3/AGT/F7/IGF2/TWIST2/MAP1B/CYP1B1/AGTR1/MIR145/PCSK1/CXCL12/DDR2/SORBS1
BP	GO:0048706	embryonic skeletal system development	0.002947	GLI3/NKX3-2/HOXD10/NOG/ZEB1/COL2A1/SIX2/WNT9A/HAND2/HOXA4/TGFB3
BP	GO:0051781	positive regulation of	0.002971	THBS4/HTR2B/SSTR5/SVIL/FGF2/PTN/FGF7/IGF2/TGFB3
BP	GO:1903035	negative regulation of	0.002971	FGF2/MMRN1/FGA/PRKG1/PHLDB2/FGG/SERPINF2/F2/VTN
BP	GO:1904427	positive regulation of	0.002971	RGN/ANK2/JPH2/NPSR1/CACNB2/F2/AKAP6/TRPC1/HSPA2
BP	GO:0014003	oligodendrocyte development	0.002984	CNTNAP1/WASF3/GPM6B/CNTN1/SOX10/ASCL1
BP	GO:1901021	positive regulation of calcium ion	0.002984	RGN/ANK2/JPH2/CACNB2/AKAP6/HSPA2
BP	GO:1904706	negative regulation of vascular	0.002984	MYOCD/TPM1/ADIPOQ/PRKG1/CNN1/TGFB3
BP	GO:0051592	response to calcium ion	0.003122	ADCY1/FGA/SYT4/CASQ2/KCNMB1/FGG/KCNMA1/TRPC1/PCSK1/PRKAA2/TUBA1A/PENK
BP	GO:0048167	regulation of synaptic plasticity	0.003131	RELN/SLC8A2/ADCY1/PTN/CNTN4/SYT4/GIP/MAP1A/NFATC4/AGT/NOG/SCGN/MAP1B/SCT/CHRDL1
BP	GO:0050920	regulation of chemotaxis	0.003162	THBS4/JAM3/FGF2/PTN/FGF10/PRKD1/SEMA3E/CCL21/F7/SMOC2/ELANE/AZU1/PLXNA4/SLIT2/CXCL12/GREM1
BP	GO:0071466	cellular response to xenobiotic	0.003192	SULT1C4/SLCO1B3/CES3/ADIPOQ/AIM2/BCHE/ANKRD1/AOX1/CYP1B1/SOX10/SULT2A1/FMO1/KCNH2/PRKAA2
BP	GO:0003073	regulation of systemic	0.003195	TPM1/CMA1/CTSG/AR/TACR1/AGT/SERPINF2/ADAMTS16/AGTR1
BP	GO:0051492	regulation of stress fiber	0.003195	FERMT2/TPM1/APOA1/SFRP1/PHLDB2/TACR1/SERPINF2/MYOC/TGFB3
BP	GO:0090101	negative regulation of transmembrane receptor protein	0.003196	TGFB1I1/CILP/ASPN/SFRP1/RBPMS2/CHRDL2/SFRP2/NOG/GREM2/CHRD/TGFB3/GREM1/CHRDL1
BP	GO:0014066	regulation of phosphatidylinositol 3-kinase	0.003226	ROR2/RELN/FGF2/IGF1/SEMA3E/AGT/DCN/F2/MYOC/DDR2
BP	GO:0010765	positive regulation of	0.003279	ATP1B2/DMD/NKX2-5/CNTN1/SCN4B
BP	GO:0034694	response to	0.003279	SFRP1/APOB/CCL21/PTGFR/PRKAA2
BP	GO:0098900	regulation of action potential	0.00328	SLC8A2/ANK2/CNR1/TACR1/FLNA/CACNA1C/CD36

BP	GO:0061351	neural precursor cell	0.003297	GLI3/FGF2/LIMS2/PTN/CDON/CDH2/IGF2BP1/EML1/DIXDC1/EMX1/SOX10/ASCL1
BP	GO:0001654	eye development	0.003318	GLI3/COL8A1/FGF2/CDON/VSTM4/ALDH1A2/TUB/FGF10/SALL2/TMOD1/PRICKLE1/EFEMP1/ZEB1/CRYAB/MFAP5/WNT9A/CYP1B1/PRPH2/SDK2/ANGPTL7/CACNA1C/CHRDL1/NEUROD1
BP	GO:0008343	adult feeding	0.003328	NPY/CARTPT/DMBX1
BP	GO:0014012	peripheral nervous system	0.003328	TNC/MAP1B/APOD
BP	GO:0014848	urinary tract smooth muscle	0.003328	HTR2A/TACR1/KCNMA1
BP	GO:0014866	skeletal	0.003328	MYH11/ACTA1/ACTC1
BP	GO:0097090	presynaptic membrane	0.003328	PTPRD/NLGN4X/NLGN4Y
BP	GO:0098917	retrograde trans-synaptic	0.003328	SYT4/CNR1/GUCY1A1
BP	GO:1903365	regulation of	0.003328	GRPR/GRP/PENK
BP	GO:0010712	regulation of collagen	0.00334	SERPINF2/F2/MIR145/MFAP4/TGFB3/DDR2
BP	GO:0014911	positive regulation of	0.00334	IGF1/NR4A3/AGT/CYP1B1/DDR2/VTN
BP	GO:0050806	positive regulation of synaptic	0.003362	ROR2/RELN/CACNG4/SLC8A2/ADCY1/PTN/TACR1/GIP/NFATC4/NOG/SCGN/TACR2/LAMA2
BP	GO:0021511	spinal cord	0.00337	RELN/GLI3/CHRD/ASCL1
BP	GO:0043691	reverse	0.00337	APOA1/APOC3/APOA2/CES1
BP	GO:0071379	cellular response to	0.00337	SFRP1/APOB/PTGFR/PRKAA2
BP	GO:0010720	positive regulation of cell development	0.003422	ROR2/RELN/GLI3/FGF2/PTN/PDE3A/PTPRD/CDON/NTN1/PLA2G3/ADIPOQ/TNFSF9/DIXDC1/ZBTB16/RND2/MAP6/MAP1B/PLXNA4/NGF/SOX10/MIR145/SLIT2/CXCL12/ASCL1/IL33
BP	GO:0019217	regulation of fatty acid	0.003431	PLA2G3/ADIPOQ/APOC3/RGN/PDK4/CNR1/GIP/NR4A3/FMO1
BP	GO:0002062	chondrocyte differentiation	0.003441	PTH1R/GLI3/NKX3-2/SFRP2/EFEMP1/ZBTB16/COL2A1/SIX2/WNT9A/GREM1
BP	GO:0055117	regulation of cardiac muscle	0.003487	CHGA/PLN/ATP1A2/ANK2/CASQ2/DMD/NKX2-5/CACNA1C
BP	GO:0098739	import across plasma membrane	0.00358	CACNA1H/SLC8A2/KCNJ8/SCN7A/ATP1B2/ATP1A2/SLC38A3/ABCC9/AGT/RGS4/SLC7A2/AQP8/KCNH2/CACNA1C/CD36
BP	GO:0032387	negative regulation of	0.003598	PLN/ADIPOQ/RAB23/CRYAB/MAP1B/APOD/CD36
BP	GO:0002526	acute inflammatory	0.003666	NPY/PTGER3/HP/AHSG/CNR1/APOA2/TACR1/ELANE/SERPINF2/F2
BP	GO:0072006	nephron development	0.003672	TMEM59L/GLI3/FGF2/ADIPOQ/ACTA2/GPC3/AGT/NOG/SIX2/GREB1L/ADAMTS16/GREM1

BP	GO:0030168	platelet activation	0.00373	MMRN1/FGA/PRKG1/CTSG/CSRP1/FLNA/DGKG/FGL1/FGG/F2/MYL9
BP	GO:0009743	response to carbohydrate	0.003742	COLEC12/GRIK5/CMA1/ADIPOQ/UCN3/APOA2/GIP/APOB/NPTX1/MAP1B/ADCY5/GCG/PCSK1/PRKAA2/PTPRN/NEUROD1
BP	GO:0010664	negative regulation of	0.003764	MYOCD/SFRP2/NKX2-5/MIR145/HAND2
BP	GO:0090183	regulation of	0.003764	ADIPOQ/AGT/NOG/SIX2/GREM1
BP	GO:1990776	response to	0.003764	ACTN2/AGT/AGTR1/MIR145/DDR2
BP	GO:0150063	visual system development	0.003765	GLI3/COL8A1/FGF2/CDON/VSTM4/ALDH1A2/TUB/FGF10/SALL2/TMOD1/PRICKLE1/EFEMP1/ZEB1/CRYAB/MFAP5/WNT9A/CYP1B1/PRPH2/SDK2/ANGPTL7/CACNA1C/CHRDL1/NEUROD1
BP	GO:0031214	biomineral tissue development	0.003903	ROR2/PTH1R/IGF1/PTN/ASPN/GPC3/AHSG/PRICKLE1/GPM6B/RSPO2/MGP/DDR2/GREM1
BP	GO:0001938	positive regulation of	0.003903	THBS4/HTR2B/FGF2/FGF10/FGF7/PRKD1/VIP/IGF2/AGTR1/CXCL12
BP	GO:0030100	regulation of endocytosis	0.003908	ADIPOQ/CLIP3/SYT4/APOC3/GPC3/PRKD1/CCL21/TF/SFRP4/STON1/RSPO1/EPHA3/CD36/GREM1/VTN
BP	GO:0034109	homotypic cell-cell adhesion	0.003946	MMRN1/FGA/PRKG1/CTSG/CSRP1/FLNA/FGL1/FGG/MYL9
BP	GO:0061050	regulation of cell growth involved in	0.004059	IGF1/PI16/RGS4/AKAP6
BP	GO:0090189	regulation of branching	0.004059	AGT/NOG/SIX2/GREM1
BP	GO:2000738	positive regulation of	0.004059	FGF2/PTN/RBM24/NKX2-5
BP	GO:0010466	negative regulation of peptidase	0.004064	TFPI2/SLCO1B3/ITIH5/PI16/GPC3/AHSG/SFRP2/SERPINA6/ITIH2/CRYAB/SERPINF2/SERPIND1/NGF/RECK/PI15/VTN
BP	GO:0032272	negative regulation of	0.004079	TUBB4A/SVIL/KANK4/CLIP3/LMOD1/TMOD1/STMN2/SLIT2
BP	GO:0032890	regulation of organic acid	0.004079	PLA2G3/SV2A/SYT4/ATP1A2/SLC38A3/CES1/AGT/RGS4
BP	GO:0048644	muscle organ morphogenesis	0.004079	TPM1/ZFPM2/ANKRD1/NOG/NKX2-5/MIR145/ACTC1/MYLK
BP	GO:0048844	artery morphogenesis	0.004079	MYOCD/FOXF1/FKBP10/APOB/NOG/MIR145/HAND2/MYLK
BP	GO:0051966	regulation of synaptic	0.004079	ROR2/RELN/CACNG4/HTR2A/CDH2/ATP1A2/PLPPR4/CNR1
BP	GO:2001257	regulation of cation channel	0.004081	KCNE4/PLN/KCNK3/ACTN2/ANK2/ABCC9/CASQ2/DMD/GRP/JPH2/CACNB2/AKAP6
BP	GO:0006953	acute-phase	0.004147	PTGER3/HP/AHSG/CNR1/SERPINF2/F2
BP	GO:0070509	calcium ion import	0.004147	CACNA1H/PLN/TRPC4/CACNB2/GCG/CXCL12

BP	GO:0018108	peptidyl-tyrosine phosphorylation	0.004256	ROR2/THBS4/RELN/GHR/IGF1/GFRA1/HTR2A/ADIPOQ/SFRP1/FGF10/FGF7/SFRP2/AGT/EFEMP1/IGF2/CNTN1/EPHA3/CD36/DDR2/EPHA7/GREM1/VTN
BP	GO:0044706	multi-multicellular organism process	0.004259	PTN/FBLN1/TAC1/IDO1/SLC38A3/CNR1/AR/TACR1/GIP/TAC3/AGT/PTGFR/RECK/ADCYAP1/TGFB3
BP	GO:0050900	leukocyte migration	0.004262	ROR2/THBS4/CHGA/JAM3/ITGA7/JAM2/PTN/PRTN3/CTSG/CH25H/TACR1/CCL21/F7/ASB2/ELANE/AZU1/APOD/SLIT2/CXCL12/LYVE1/IL33/GREM1/ITGA9
BP	GO:0016125	sterol metabolic process	0.004298	APOA1/LEPR/APOA2/CH25H/CES1/APOB/SOAT2/CYP1B1/AQP8/SULT2A1/PRKAA2/FAXDC2
BP	GO:0050770	regulation of axonogenesis	0.004298	NTN1/CDH2/SEMA3E/DIXDC1/RND2/MAP6/MAP1B/PLXNA4/NGF/SLIT2/CXCL12/EPHA7
BP	GO:0009954	proximal/distal pattern	0.004299	GLI3/ALDH1A2/FGF10/HOXD10/GREM1
BP	GO:0055075	potassium ion	0.004299	ATP1B2/ATP1A2/KCNA5/KCNMA1/KCNH2
BP	GO:0060074	synapse	0.004299	CNTNAP1/SEZ6/RELN/NFATC4/CHRDL1
BP	GO:0060563	neuroepithelial cell	0.004299	FGF2/CDH2/MAP1B/EMX1/ASCL1
BP	GO:0090075	relaxation of	0.004299	CHGA/PLN/PRKG1/ATP1A2/GUCY1A1
BP	GO:0099171	presynaptic modulation of	0.004299	ADCY1/HTR2A/NGFR/NOG/CHRM2
BP	GO:0046503	glycerolipid catabolic	0.004303	CPS1/GPIHBP1/APOC3/APOA2/PLA2G5/APOB/PNLIPRP2
BP	GO:0048511	rhythmic process	0.004344	ADCY1/PTN/NGFR/ADIPOQ/AMH/HLF/CARTPT/SLIT3/C3orf70/F7/CYP1B1/AFP/SLIT2/ENOX1/PRKAA2/GDF10/TGFB3/PTPRN/PTX3
BP	GO:0007494	midgut	0.004468	CPS1/ALDH1A2/FOXF1
BP	GO:0010172	embryonic body	0.004468	CDON/PHLDB2/GREM2
BP	GO:0014820	tonic smooth	0.004468	HTR2A/AGT/MYLK
BP	GO:0034379	very-low-density lipoprotein	0.004468	APOC3/APOB/SOAT2
BP	GO:0060601	lateral sprouting from	0.004468	FGF10/AR/NOG
BP	GO:0070471	uterine smooth muscle	0.004468	TACR1/AGT/TACR2
BP	GO:0086016	AV node cell	0.004468	CACNB2/SCN4B/CACNA1C
BP	GO:0086027	AV node cell to bundle of His	0.004468	CACNB2/SCN4B/CACNA1C
BP	GO:0048880	sensory system development	0.00453	GLI3/COL8A1/FGF2/CDON/VSTM4/ALDH1A2/TUB/FGF10/SALL2/TMOD1/PRICKLE1/EFEMP1/ZEB1/CRYAB/MFAP5/WNT9A/CYP1B1/PRPH2/SDK2/ANGPTL7/CACNA1C/CHRDL1/NEUROD1

BP	GO:0018212	peptidyl-tyrosine modification	0.004533	ROR2/THBS4/RELN/GHR/IGF1/GFRA1/HTR2A/ADIPOQ/SFRP1/FGF10/FGF7/SFRP2/AGT/EFEMP1/IGF2/CNTN1/EPHA3/CD36/DDR2/EPHA7/GREM1/VTN
BP	GO:0010665	regulation of cardiac muscle	0.0046	MYOCD/SFRP2/AGT/NKX2-5/MIR145/HAND2
BP	GO:0046850	regulation of	0.0046	SFRP1/CARTPT/PDK4/LEPR/TF/GREM1
BP	GO:0030902	hindbrain development	0.004761	SEZ6/FGF2/ALDH1A2/ATP1B2/NLGN4X/NOG/DIXDC1/CNTN1/GDF10/ASCL1/TUBA1A/NEUROD1
BP	GO:0007620	copulation	0.004837	TAC1/CNR1/TACR1/VIP
BP	GO:0034104	negative regulation of	0.004837	SFRP1/CARTPT/AGT/GREM1
BP	GO:0048485	sympathetic nervous system	0.004837	GFRA3/PLXNA4/HAND2/ASCL1
BP	GO:2001044	regulation of integrin-	0.004837	LIMS2/PRKD1/FLNA/LAMA2
BP	GO:0030212	hyaluronan	0.004886	FGF2/ITIH5/HAS1/ITIH2/LYVE1
BP	GO:0048333	mesodermal cell	0.004886	FOXF1/ITGA8/SFRP2/SIX2/MIR145
BP	GO:1901653	cellular response to peptide	0.004976	GPT/IGFBP1/GHR/IGF1/GLP2R/ACTN2/NGFR/CPS1/ADIPOQ/SLC2A4/PDK4/AHSG/NR4A3/AGT/IGF2/MAP1B/CYP1B1/AGTR1/MIR145/CD36/DDR2/SORBS1
BP	GO:0030195	negative regulation of	0.005089	FGA/PRKG1/FGG/SERPINF2/F2/VTN
BP	GO:0048009	insulin-like growth factor	0.005089	IGFBP1/GHR/IGF1/CILP/AR/IGF2
BP	GO:0046209	nitric oxide metabolic	0.005107	NPY/CPS1/RGN/AGT/CYP1B1/PTGIS/CD36/PTX3
BP	GO:0043255	regulation of carbohydrate	0.005154	GPT/PTH1R/IGF1/PPP1R3C/ADIPOQ/LEPR/IGF2/GCG/SORBS1
BP	GO:0071375	cellular response to peptide hormone stimulus	0.005168	GPT/IGFBP1/GHR/GLP2R/ACTN2/CPS1/ADIPOQ/SLC2A4/PDK4/AHSG/NR4A3/AGT/IGF2/MAP1B/CYP1B1/AGTR1/MIR145/DDR2/SORBS1
BP	GO:0007204	positive regulation of cytosolic	0.005197	ADRA1D/PTGER3/HTR2A/TAC1/TACR1/AGT/PTGFR/JPH2/ADCY5/AGTR1/ADCYAP1/CACNA1C/CD36
BP	GO:0009855	determination of bilateral symmetry	0.005205	BICC1/ALDH1A2/FOXF1/FGF10/NKX3-2/ASB2/NKX2-5/GREM2/HAND2/DAAM2/GREM1
BP	GO:0010721	negative regulation of cell development	0.005218	DLL3/GLI3/IGF1/PTN/NTN1/ADIPOQ/SFRP1/SYT4/CARTPT/SEMA3E/NFATC4/LRRC17/FRZB/NOG/F2/SOX10/DAAM2/EPHA7
BP	GO:2001057	reactive nitrogen species	0.00549	NPY/CPS1/RGN/AGT/CYP1B1/PTGIS/CD36/PTX3
BP	GO:0009799	specification of symmetry	0.005491	BICC1/ALDH1A2/FOXF1/FGF10/NKX3-2/ASB2/NKX2-5/GREM2/HAND2/DAAM2/GREM1
BP	GO:0043271	negative regulation of	0.005491	KCNE4/PLN/HTR2A/ACTN2/ATP1A2/CNR1/VIP/CASQ2/GRP/RGS4/KCNH2

BP	GO:0001935	endothelial cell proliferation	0.005504	THBS4/HTR2B/FGF2/VSTM4/NGFR/APOA1/ALDH1A2/FGF10/FGF7/PRKD1/VIP/IGF2/AGTR1/CXCL12
BP	GO:0010737	protein kinase A	0.005527	ADIPOQ/MYOM1/AKAP12/AKAP6/GCG
BP	GO:0033687	osteoblast	0.005527	MN1/NPR3/SFRP1/TNN/GREM1
BP	GO:0086005	ventricular cardiac muscle	0.005527	KCNE4/KCNJ8/ANK2/KCNH2/CACNA1C
BP	GO:0090218	positive regulation of	0.005527	FGF2/TNFAIP8L3/PRKD1/CCL21/F2
BP	GO:1905332	positive regulation of	0.005527	FGF2/AR/AGT/NOG/GREM1
BP	GO:0009620	response to fungus	0.005549	CHGA/DEFA6/CTSG/PLA2G5/MPO/ELANE/PTX3
BP	GO:0060389	pathway-restricted SMAD	0.005549	AMH/NOG/BMP3/TNXB/GDF10/TGFB3/GREM1
BP	GO:0010662	regulation of striated muscle	0.005615	MYOCD/SFRP2/AGT/NKX2-5/MIR145/HAND2
BP	GO:1900047	negative regulation of	0.005615	FGA/PRKG1/FGG/SERPINF2/F2/VTN
BP	GO:0044062	regulation of	0.005708	TACR1/AGT/NPSR1/AGTR1
BP	GO:0071305	cellular	0.005708	MN1/SFRP1/CYP24A1/PENK
BP	GO:1900120	regulation of	0.005708	RGMA/ADIPOQ/NOG/GREM2
BP	GO:0014831	gastro-intestinal	0.005816	HTR2B/PTGER3/TACR2
BP	GO:0034370	triglyceride-rich lipoprotein	0.005816	APOA1/APOC3/APOA2
BP	GO:0034372	very-low-density lipoprotein	0.005816	APOA1/APOC3/APOA2
BP	GO:0060379	cardiac muscle cell myoblast	0.005816	MYOCD/PRICKLE1/GREM1
BP	GO:0061303	cornea development in	0.005816	PRICKLE1/WNT9A/ANGPTL7
BP	GO:0086067	AV node cell to bundle of His	0.005816	CACNB2/SCN4B/CACNA1C
BP	GO:0099550	trans-synaptic signaling,	0.005816	SYT4/CNR1/GUCY1A1
BP	GO:2000035	regulation of	0.005816	SFRP2/NAP1L2/MIR145
BP	GO:0010906	regulation of glucose	0.005857	GPT/IGF1/ADIPOQ/RGN/PDK4/LEPR/IGF2/GCG/SORBS1
BP	GO:0035914	skeletal muscle cell	0.006019	MYOCD/CDON/HLF/SMYD1/ANKRD1/RBM24/ASB2
BP	GO:0048645	animal organ formation	0.006019	GLI3/FGF2/FGF10/NKX3-2/AR/NOG/HAND2
BP	GO:0010543	regulation of platelet	0.006179	MMRN1/PRKG1/CTSG/FLNA/FGG/F2
BP	GO:0010656	negative regulation of	0.006179	MYOCD/IGF1/SFRP2/NKX2-5/MIR145/HAND2
BP	GO:0031103	axon	0.006179	PTN/TNC/GAP43/FLNA/MAP1B/APOD
BP	GO:0032094	response to food	0.006225	NPY/GHR/CPS1/CARTPT/MPO
BP	GO:0071868	cellular response to	0.006235	HTR2B/HTR2A/ADIPOQ/NR4A3/FLNA/RGS4/ADCY5/CHRM2/GNAO1

BP	GO:0071870	cellular response to	0.006235	HTR2B/HTR2A/ADIPOQ/NR4A3/FLNA/RGS4/ADCY5/CHRM2/GNAO1
BP	GO:0110020	regulation of actomyosin	0.006235	FERMT2/TPM1/APOA1/SFRP1/PHLDB2/TACR1/SERPINF2/MYOC/TGFB3
BP	GO:0006109	regulation of carbohydrate metabolic	0.006237	GPT/PTH1R/IGF1/HTR2A/PPP1R3C/ADIPOQ/RGN/PDK4/LEPR/IGF2/GCG/PRKAA2/SORBS1
BP	GO:0090596	sensory organ morphogenesis	0.006271	GLI3/COL8A1/FGF2/CDON/NTN1/FGF10/NKX3-2/ITGA8/FRZB/NOG/EFEMP1/ZEB1/MFAP5/COL2A1/SIX2/WNT9A/SDK2
BP	GO:0061053	somite development	0.006322	DLL3/SFRP1/ALDH1A2/FOXF1/SFRP2/FRZB/NOG/MEOX2
BP	GO:1901879	regulation of protein	0.006322	SVIL/ACTN2/LMOD1/CFL2/TMOD1/MAP1A/MAP1B/STMN2
BP	GO:0050921	positive regulation of	0.006422	THBS4/FGF2/PTN/FGF10/PRKD1/CCL21/F7/SMOC2/AZU1/SLIT2/CXCL12
BP	GO:0042593	glucose homeostasis	0.006502	SSTR5/GRIK5/CMA1/NGFR/ADIPOQ/SLC2A4/CARTPT/PDK4/LEPR/CNR1/NPTX1/ADCY5/GCG/PRKAA2/PTPRN/NEUROD1
BP	GO:0043010	camera-type eye development	0.006576	GLI3/COL8A1/FGF2/CDON/VSTM4/ALDH1A2/TUB/FGF10/TMOD1/PRICKLE1/EFEMP1/ZEB1/CRYAB/WNT9A/CYP1B1/PRPH2/SDK2/ANGPTL7/CACNA1C/NEUROD1
BP	GO:0030301	cholesterol transport	0.006627	PNLIP/ADIPOQ/APOA1/ABCA8/APOC3/APOA2/CES1/APOB/SOAT2/CD36
BP	GO:0010640	regulation of platelet-derived growth factor	0.006679	MYOCD/ADIPOQ/F7/APOD
BP	GO:0030539	male genitalia	0.006679	ROR2/FGF10/AR/GREB1L
BP	GO:0030307	positive regulation of	0.006703	IGFBP1/IGF1/NTN1/SFRP1/SYT4/SFRP2/RND2/MAP1B/F2/AKAP6/NGF/CXCL12
BP	GO:0033500	carbohydrate homeostasis	0.00675	SSTR5/GRIK5/CMA1/NGFR/ADIPOQ/SLC2A4/CARTPT/PDK4/LEPR/CNR1/NPTX1/ADCY5/GCG/PRKAA2/PTPRN/NEUROD1
BP	GO:0010659	cardiac muscle cell apoptotic	0.006783	MYOCD/SFRP2/AGT/NKX2-5/MIR145/HAND2
BP	GO:0035176	social behavior	0.006783	GRPR/NRXN2/NLGN4X/GRP/NLGN4Y/NRXN3
BP	GO:0048846	axon extension involved in axon	0.006983	SLIT3/SEMA3E/PLXNA4/SLIT2/CXCL12
BP	GO:1902284	neuron projection extension	0.006983	SLIT3/SEMA3E/PLXNA4/SLIT2/CXCL12
BP	GO:0048639	positive regulation of	0.007025	FGF2/GHR/IGF1/NTN1/ZFPM2/SYT4/RND2/IGF2/MAP1B/AKAP6/NGF/CXCL12
BP	GO:0046849	bone remodeling	0.007247	PTH1R/PTN/SFRP1/CARTPT/PDK4/LEPR/TF/GREM1
BP	GO:0010642	negative regulation of platelet-derived	0.007383	MYOCD/ADIPOQ/APOD

BP	GO:0014874	response to stimulus	0.007383	AGT/SGCA/FBXO32
BP	GO:0033604	negative regulation of	0.007383	CHGA/SYT4/CNR1
BP	GO:0033605	positive regulation of	0.007383	CARTPT/VIP/CXCL12
BP	GO:0051481	negative regulation of	0.007383	KCNK3/ATP1A2/KCNA5
BP	GO:0051918	negative regulation of	0.007383	SERPINF2/F2/VTN
BP	GO:0072537	fibroblast	0.007383	ACTA2/AKAP12/DDR2
BP	GO:0097475	motor neuron	0.007383	RELN/NTN1/ASCL1
BP	GO:1900121	negative regulation of	0.007383	RGMA/ADIPOQ/NOG
BP	GO:1901894	regulation of ATPase-coupled calcium	0.007383	PLN/RGN/HSPA2
BP	GO:2001046	positive regulation of	0.007383	LIMS2/FLNA/LAMA2
BP	GO:0003206	cardiac chamber morphogenesis	0.007395	CPE/TPM1/ZFPM2/FOXF1/SLIT3/SFRP2/NOG/NKX2-5/HAND2/SLIT2
BP	GO:0019730	antimicrobial humoral response	0.007395	PRTN3/FGA/DEFA6/CTSG/DMBT1/TF/ELANE/AZU1/F2/COLEC11
BP	GO:0014009	glial cell	0.00743	NTN1/LEPR/LGI4/SOX10/PENK/IL33
BP	GO:0050819	negative regulation of	0.00743	FGA/PRKG1/FGG/SERPINF2/F2/VTN
BP	GO:0071320	cellular response to cAMP	0.00743	CPS1/ADIPOQ/AKAP6/CYP1B1/AQP8/PENK
BP	GO:0007224	smoothened signaling	0.007472	PTCH2/GLI3/CDON/SFRP1/FOXF1/GAS1/FGF10/DZIP1/GPC3/NOG/TUBA1A
BP	GO:1901655	cellular response to	0.007482	ADCY1/SFRP1/AR/PTGFR/ADCY5/CYP1B1/SOX10/FBXO32/PRKAA2
BP	GO:0002237	response to molecule of bacterial origin	0.007634	CD274/SSC5D/KCNJ8/CPS1/IDO1/DEFA6/FGF10/CTSG/AKAP12/CNR1/APOB/ANKRD1/MPO/PTGFR/ELANE/TRIM6/THPO/FMO1/PCSK1/PENK/CD36
BP	GO:1902904	negative regulation of supramolecular	0.007706	TUBB4A/SVIL/KANK4/CLIP3/LMOD1/PHLDB2/TMOD1/CRYAB/MAP1B/STMN2/MYOC/SLIT2
BP	GO:0006112	energy reserve metabolic	0.007745	PYGM/IGF1/PPP1R3C/LEPR/PPP1R1A/IGF2/SORBS1/GFPT2
BP	GO:0034114	regulation of heterotypic	0.007753	ADIPOQ/FGA/APOA1/FGG
BP	GO:0043567	regulation of insulin-like growth factor	0.007753	IGFBP1/IGF1/CILP/AR
BP	GO:0050927	positive regulation of	0.007753	FGF10/F7/AZU1/CXCL12
BP	GO:2000679	positive regulation of transcription	0.007753	IGF1/TRIM6/HAND2/NEUROD1

BP	GO:0045823	positive regulation of	0.007804	CHGA/TPM1/ATP1A2/RGS4/NKX2-5
BP	GO:0052652	cyclic purine nucleotide	0.007804	ADCY1/PDE7B/PDE9A/GUCY1A1/ADCY5
BP	GO:0002686	negative regulation of	0.008119	CCL21/APOD/SLIT2/CXCL12/IL33/GREM1
BP	GO:0010518	positive regulation of	0.008119	HTR2B/FGF2/HTR2A/PLA2G5/AGT/AGTR1
BP	GO:0010658	striated muscle cell apoptotic	0.008119	MYOCD/SFRP2/AGT/NKX2-5/MIR145/HAND2
BP	GO:0051703	biological process involved in intraspecies	0.008119	GRPR/NRXN2/NLGN4X/GRP/NLGN4Y/NRXN3
BP	GO:0032496	response to lipopolysaccharide	0.008164	CD274/KCNJ8/CPS1/IDO1/DEFA6/FGF10/CTSG/AKAP12/CNR1/APOB/ANKRD1/MPO/PTGFR/ELANE/TRIM6/THPO/FMO1/PCSK1/PENK/CD36
BP	GO:0098742	cell-cell adhesion via plasma-membrane adhesion	0.008586	MPZ/CDH19/CDHR1/CADM3/PCDHB4/PTPRD/PCDHB5/CDH2/CNTN4/ADIPOQ/APOA1/NEXN/CLDN6/SPARCL1/CLDN11/SDK2/HMCN1
BP	GO:0008064	regulation of actin polymerization	0.008652	SVIL/KANK4/ACTN2/LMOD1/ELN/CFL2/TMOD1/CCL21/SLIT2/DAAM2/CXCL12
BP	GO:0048608	reproductive structure development	0.008685	ROR2/MYOCD/GFRA1/ZFPM2/SFRP1/AMH/FGF10/TNC/SLIT3/SFRP2/AR/NOG/GREB1L/CYP1B1/AFP/SLIT2/PTPRN/PTX3
BP	GO:0009187	cyclic nucleotide	0.008688	ADCY1/PDE7B/PDE9A/GUCY1A1/ADCY5
BP	GO:1903115	regulation of actin filament-	0.008688	PLN/ACTA2/ATP1A2/ANK2/CACNA1C
BP	GO:1903524	positive regulation of	0.008688	CHGA/TPM1/ATP1A2/RGS4/NKX2-5
BP	GO:0007405	neuroblast proliferation	0.008827	GLI3/FGF2/PTN/CDON/EML1/SOX10/ASCL1
BP	GO:0042246	tissue regeneration	0.008827	IGFBP1/IGF1/PTN/FGF10/GAP43/SGCA/APOD
BP	GO:0043462	regulation of ATP-dependent	0.008827	PLN/TPM1/ATP1B2/FGF10/RGN/TPM2/HSPA2
BP	GO:0050832	defense response	0.008853	CHGA/DEFA6/CTSG/PLA2G5/MPO/ELANE
BP	GO:0055078	sodium ion homeostasis	0.008853	SCN7A/ATP1B2/ATP1A2/TACR1/AGT/AGTR1
BP	GO:0002444	myeloid leukocyte	0.008911	CHGA/PLA2G3/FOXF1/CTSG/NR4A3/GRP/ELANE/AZU1/F2
BP	GO:0022010	central nervous system	0.008935	CNTNAP1/GPM6B/CNTN1/SOX10
BP	GO:0032291	axon ensheathment in	0.008935	CNTNAP1/GPM6B/CNTN1/SOX10
BP	GO:0035994	response to	0.008935	CDH2/ANKRD1/DMD/DDR2
BP	GO:0048745	smooth muscle	0.008935	ITGA8/MIR145/TNN/MYLK

BP	GO:0050926	regulation of positive	0.008935	FGF10/F7/AZU1/CXCL12
BP	GO:2000647	negative regulation of	0.008935	GLI3/FGF2/FBLN1/FGF10
BP	GO:0050768	negative regulation of	0.009076	DLL3/PTN/NTN1/SYT4/SEMA3E/NFATC4/NOG/F2/SOX10/DAAM2/EPHA7
BP	GO:0031115	negative regulation of	0.009175	TUBB4A/CLIP3/STMN2
BP	GO:0033700	phospholipid	0.009175	APOA1/APOC3/APOA2
BP	GO:0050930	induction of	0.009175	FGF10/AZU1/CXCL12
BP	GO:0070593	dendrite self-	0.009175	CNTN4/NEXN/TNN
BP	GO:0045927	positive regulation of growth	0.009335	IGFBP1/FGF2/GHR/IGF1/NTN1/ZFPM2/SFRP1/SYT4/SFRP2/RND2/IGF2/MAP1B/F2/AKAP6/NGF/CXCL12
BP	GO:1901888	regulation of cell junction assembly	0.009362	FERMT2/PTPRD/NTN1/NEGR1/SFRP1/PHLDB2/AGT/GPM6B/APOD/MYOC/FLRT2/EPHA3/EPHA7/GREM1
BP	GO:0001656	metanephros development	0.009397	GLI3/ADIPOQ/FGF10/ITGA8/GPC3/SIX2/GREB1L/GREM1
BP	GO:0002028	regulation of sodium ion	0.009397	ATP1B2/FXYD6/ATP1A2/DMD/GRP/NKX2-5/CNTN1/SCN4B
BP	GO:0006909	phagocytosis	0.009403	COLEC12/PRTN3/ADIPOQ/APOA1/SRPX/TUB/LEPR/AHSG/APOA2/PLA2G5/ELANE/AZU1/COLEC11/CD36/PTX3
BP	GO:0001736	establishment of planar polarity	0.009487	ROR2/SFRP1/RSPO3/GPC3/SFRP2/PRICKLE1/PRICKLE2
BP	GO:0007164	establishment of tissue polarity	0.009487	ROR2/SFRP1/RSPO3/GPC3/SFRP2/PRICKLE1/PRICKLE2
BP	GO:0048839	inner ear development	0.009566	GLI3/FGF2/KCNK3/NTN1/FGF10/ITGA8/PLPPR4/FRZB/ZEB1/KCNK2/COL2A1/TGFB3/NEUROD1
BP	GO:0043388	positive regulation of	0.009634	MYOCD/IGF1/NGF/TRIM6/HAND2/NEUROD1
BP	GO:1990138	neuron projection extension	0.009635	NTN1/SYT4/SLIT3/NDN/PRICKLE1/SEMA3E/MAP1B/EMX1/PLXNA4/SLIT2/TNN/CXCL12
BP	GO:0002691	regulation of cellular	0.00964	JAM3/CCL21/ELANE/CXCL12/LYVE1
BP	GO:0032941	secretion by	0.00964	NPR3/FGF10/PRICKLE1/TACR1/SCT
BP	GO:0032965	regulation of collagen	0.00964	SERPINF2/F2/MIR145/TGFB3/DDR2
BP	GO:0050892	intestinal	0.00964	PNLIP/APOA1/APOA2/SOAT2/CD36
BP	GO:0051281	positive regulation of release of	0.00964	JPH2/NPSR1/F2/AKAP6/TRPC1
BP	GO:1902742	apoptotic process involved	0.00964	SLIT3/CRYAB/NKX2-5/HAND2/SLIT2
BP	GO:0061458	reproductive system development	0.009869	ROR2/MYOCD/GFRA1/ZFPM2/SFRP1/AMH/FGF10/TNC/SLIT3/SFRP2/AR/NOG/GREB1L/CYP1B1/AFP/SLIT2/PTPRN/PTX3

BP	GO:0009749	response to glucose	0.009964	GRIK5/CMA1/ADIPOQ/UCN3/APOA2/GIP/NPTX1/ADCY5/GCG/PCSK1/PRKAA2/PTPRN/NEUROD1
BP	GO:0010675	regulation of cellular	0.009971	GPT/PTH1R/IGF1/PPP1R3C/ADIPOQ/RGN/PDK4/LEPR/IGF2/GCG/SORBS1
BP	GO:0030832	regulation of actin filament	0.009971	SVIL/KANK4/ACTN2/LMOD1/ELN/CFL2/TMOD1/CCL21/SLIT2/DAAM2/CXCL12
BP	GO:0035249	synaptic transmission,	0.009973	ROR2/RELN/CACNG4/GRIK5/HTR2A/CDH2/ATP1A2/PLPPR4/CNR1
BP	GO:0042180	cellular ketone metabolic process	0.010109	CACNA1H/SULT1C4/PLA2G3/ADIPOQ/IDO1/APOC3/RGN/PDK4/CNR1/CES1/GIP/NR4A3/FMO1/AFP
BP	GO:0005977	glycogen metabolic	0.010183	PYGM/IGF1/PPP1R3C/LEPR/PPP1R1A/IGF2/SORBS1
BP	GO:0007205	protein kinase C-activating G protein-coupled	0.010228	HTR2B/GAP43/DGKG/AZU1
BP	GO:0010875	positive regulation of	0.010228	ADIPOQ/APOA1/ABCA8/CES1
BP	GO:0032967	positive regulation of	0.010228	SERPINF2/F2/TGFB3/DDR2
BP	GO:0048668	collateral	0.010228	PRKG1/RND2/NGF/EPHA7
BP	GO:0051953	negative regulation of	0.010228	CHGA/SYT4/CNR1/RGS4
BP	GO:1904753	negative regulation of vascular	0.010228	MYOCD/TPM1/ADIPOQ/PRKG1
BP	GO:0016042	lipid catabolic process	0.010458	PLCH2/PNLIP/CPS1/ADIPOQ/GPIHBP1/APOC3/CNR1/APOA2/PLA2G5/CES1/APOB/PLIN1/PNLIPRP2/SCT/CYP1B1/SULT2A1/PNLIPRP1/PLCXD3/CYP24A1
BP	GO:0045861	negative regulation of proteolysis	0.010458	TFPI2/SLCO1B3/ITIH5/PI16/GAS1/GPC3/AHSG/SFRP2/SERPINA6/ITIH2/MAP1A/CRYAB/SERPINF2/SERPIND1/F2/NGF/RECK/PI15/VTN
BP	GO:0042306	regulation of protein import	0.010462	GLI3/PRKD1/RAB23/FLNA/APOD/CD36
BP	GO:0042733	embryonic digit morphogenesis	0.010462	GLI3/SFRP2/PRICKLE1/NOG/ZBTB16/HAND2
BP	GO:0044344	cellular response to	0.010538	FGF2/NGFR/CPS1/SFRP1/FGF10/FGF7/NOG/SMOC2/FLRT2
BP	GO:0001738	morphogenesis of a polarized	0.010635	ROR2/SFRP1/FOXF1/RSPO3/GPC3/SFRP2/PRICKLE1/PRICKLE2
BP	GO:0032535	regulation of cellular component size	0.010649	SVIL/KANK4/ACTN2/NTN1/LMOD1/ELN/CFL2/TMOD1/SEMA3E/CCL21/RND2/MAP1B/SCT/KCNMA1/PLXNA4/NGF/SLIT2/DAAM2/CXCL12/EPHA7
BP	GO:0014912	negative regulation of	0.010661	MYOCD/TPM1/ADIPOQ/PRKG1/SLIT2
BP	GO:0021983	pituitary gland	0.010661	FGF2/ALDH1A2/FGF10/NOG/PCSK1

BP	GO:0006073	cellular glucan metabolic	0.010916	PYGM/IGF1/PPP1R3C/LEPR/PPP1R1A/IGF2/SORBS1
BP	GO:0044042	glucan metabolic process	0.010916	PYGM/IGF1/PPP1R3C/LEPR/PPP1R1A/IGF2/SORBS1
BP	GO:1901342	regulation of vasculature development	0.010958	THBS4/FGF2/CMA1/SFRP1/HSPB6/PRKD1/SFRP2/SEMA3E/AGT/SMOC2/DCN/ISM1/CYP1B1/MIR145/RECK/WARS1/PTGIS/TNN/ANGPTL7/GREM1
BP	GO:0090130	tissue migration	0.011068	FGF2/PTN/VSTM4/ACTG2/APOA1/FOXF1/ACTA2/FGF10/FGF7/PRKD1/ACTA1/TACR1/AGT/SMOC2/MEOX2/DCN/CYP1B1/ACTC1/SLIT2/DAAM2/GREM1
BP	GO:0001894	tissue homeostasis	0.01108	PTH1R/JAM3/CDHR1/JAM2/VSTM4/ATP1B2/TUB/CARTPT/PDK4/PRICKLE1/DMD/TF/COL2A1/LAMA2/TUBA1A/NEUROD1
BP	GO:0060249	anatomical structure homeostasis	0.01108	PTH1R/JAM3/CDHR1/JAM2/VSTM4/ATP1B2/TUB/CARTPT/PDK4/PRICKLE1/DMD/TF/COL2A1/LAMA2/TUBA1A/NEUROD1
BP	GO:0051604	protein maturation	0.0111	CPE/GLI3/NNAT/FBLN1/CMA1/FGA/F13A1/GAS1/CTSG/SCG5/BCHE/SPON1/CPXM2/F7/FLNA/FGG/SERPINF2/PCSK1/PRPH2
BP	GO:1900180	regulation of protein	0.011176	FERMT2/GLI3/NGFR/PRKD1/RAB23/FLNA/F2/APOD/DCLK2/CD36
BP	GO:0003214	cardiac left ventricle	0.011199	CPE/FOXF1/SFRP2
BP	GO:0030007	cellular potassium ion	0.011199	ATP1B2/ATP1A2/KCNMA1
BP	GO:0038166	angiotensin-activated	0.011199	ACTN2/AGTR1/MIR145
BP	GO:0042308	negative regulation of	0.011199	RAB23/APOD/CD36
BP	GO:0045725	positive regulation of	0.011199	IGF1/IGF2/SORBS1
BP	GO:0048934	peripheral nervous system	0.011199	HOXD10/HAND2/ASCL1
BP	GO:0048935	peripheral nervous system	0.011199	HOXD10/HAND2/ASCL1
BP	GO:0060026	convergent	0.011199	SFRP1/SFRP2/FRZB
BP	GO:0060572	morphogenesis of an epithelial	0.011199	FGF10/AR/NOG
BP	GO:0072498	embryonic skeletal joint	0.011199	NOG/COL2A1/WNT9A
BP	GO:2000052	positive regulation of	0.011199	SFRP1/RSPO3/GPC3
BP	GO:2001028	positive regulation of	0.011199	FGF2/PRKD1/SMOC2
BP	GO:0014033	neural crest cell	0.011297	HTR2B/CDH2/SFRP1/ALDH1A2/SEMA3E/FRZB/SOX10/HAND2
BP	GO:0048704	embryonic skeletal system	0.011297	GLI3/HOXD10/NOG/ZEB1/COL2A1/SIX2/HOXA4/TGFB3
BP	GO:0008347	glial cell	0.01134	RELN/GLI3/NTN1/ATP1B2/NDN/AZU1

BP	GO:0031102	neuron	0.01134	PTN/TNC/GAP43/FLNA/MAP1B/APOD
BP	GO:1903053	regulation of extracellular	0.01134	PHLDB2/AGT/TNXB/LAMA2/ANGPTL7/DDR2
BP	GO:0070507	regulation of microtubule cytoskeleton	0.01144	TUBB4A/CLIP3/PHLDB2/MPDZ/MAP1A/DIXDC1/MAP6/MAP1B/STMN2/EPHA3/PRKAA2
BP	GO:0003401	axis elongation	0.011636	SFRP1/FGF10/SFRP2/PRICKLE1
BP	GO:0010714	positive regulation of	0.011636	SERPINF2/F2/TGFB3/DDR2
BP	GO:0051873	killing by host of symbiont	0.011636	CTSG/ELANE/AZU1/F2
BP	GO:0098698	postsynaptic specialization	0.011636	PTPRD/NRXN2/GAP43/NPTX1
BP	GO:1903055	positive regulation of extracellular	0.011636	PHLDB2/AGT/TNXB/DDR2
BP	GO:0010827	regulation of glucose	0.011685	IGF1/ADIPOQ/CLIP3/GPC3/GIP/NR4A3/SORBS1
BP	GO:0010565	regulation of cellular ketone	0.011736	PLA2G3/ADIPOQ/APOC3/RGN/PDK4/CNR1/CES1/GIP/NR4A3/FMO1
BP	GO:0010719	negative regulation of epithelial to	0.011754	SFRP1/SFRP2/NOG/DACT3/MIR145
BP	GO:0060412	ventricular	0.011754	ZFPM2/SLIT3/NOG/NKX2-5/SLIT2
BP	GO:0051961	negative regulation of	0.011964	DLL3/PTN/NTN1/SYT4/SEMA3E/NFATC4/NOG/F2/SOX10/DAAM2/EPHA7
BP	GO:0044264	cellular polysaccharide	0.011989	PYGM/IGF1/PPP1R3C/HAS1/LEPR/PPP1R1A/IGF2/SORBS1
BP	GO:0045807	positive regulation of	0.011989	CLIP3/GPC3/CCL21/TF/SFRP4/CD36/GREM1/VTN
BP	GO:0033674	positive regulation of kinase activity	0.011993	ROR2/FERMT2/HTR2B/RELN/SLC8A2/FGF2/GHR/IGF1/TNFAIP8L3/HTR2A/ADIPOQ/CHRNA3/CARTPT/PRKD1/CCL21/AGT/ELANE/AZU1/F2/GCG/ADCYAP1/DDR2/GREM1
BP	GO:0009746	response to hexose	0.012148	GRIK5/CMA1/ADIPOQ/UCN3/APOA2/GIP/NPTX1/ADCY5/GCG/PCSK1/PRKAA2/PTPRN/NEUROD1
BP	GO:0055008	cardiac muscle tissue	0.012268	TPM1/ZFPM2/ANKRD1/NOG/NKX2-5/ACTC1
BP	GO:0098659	inorganic cation import across	0.012317	CACNA1H/SLC8A2/KCNJ8/SCN7A/ATP1B2/ATP1A2/ABCC9/AQP8/KCNH2/CACNA1C
BP	GO:0099587	inorganic ion import across	0.012317	CACNA1H/SLC8A2/KCNJ8/SCN7A/ATP1B2/ATP1A2/ABCC9/AQP8/KCNH2/CACNA1C

CC	GO:0062023	collagen-containing extracellular matrix	1.04E-32	THBS4/FREM1/OGN/SSC5D/TGFB1I1/COL8A1/ANGPTL1/FBLN1/ADAMTS8/DPT/PODN/PRTN3/CDON/CMA1/MMRN1/CDH2/ITIH5/CILP/ADIPOQ/FGA/APOA1/ASPN/SFRP1/F13A1/ACTA2/SRPX/CCDC80/CLC/COL26A1/ABI3BP/FGF10/CTSG/TNC/APOC3/ELN/GPC3/PRELP/AHSG/SFRP2/ITIH2/SPON1/AGT/EFEMP1/F7/FBLN5/SMOC2/ELANE/FGL1/MFAP5/FGG/SERPINF2/DCN/COL2A1/F2/SPARCL1/COL14A1/COL21A1/MYOC/COL4A6/TNXB/MFAP4/LAMA2/TNN/MGP/CXCL12/PRG4/GDF10/HMCN1/ANGPTL7/TGFB3/GREM1/VTN
CC	GO:0043292	contractile fiber	4.48E-21	FERMT2/PGM5/SYNM/ARHGEF25/SVIL/KCNJ8/SYNPO2/TPM1/ACTN2/FLNC/CALD1/IDO1/SYNC/MYOM1/ACTA2/LMOD1/ITGB1BP2/MYH11/DES/ANK2/NEXN/ABCC9/CFL2/ACTA1/TMOD1/CSRP1/PPP1R12B/ANKRD1/CASQ2/DMD/KCNA5/FLNA/CRYAB/PDLIM3/TPM2/ASB2/JPH2/LDB3/MYL9/FBXL22/ACTC1/FBXO32/CACNA1C/SMPX
CC	GO:0030016	myofibril	3.05E-19	FERMT2/PGM5/SYNM/ARHGEF25/SVIL/KCNJ8/SYNPO2/TPM1/ACTN2/FLNC/CALD1/SYNC/MYOM1/LMOD1/ITGB1BP2/DES/ANK2/NEXN/ABCC9/CFL2/ACTA1/TMOD1/CSRP1/PPP1R12B/ANKRD1/CASQ2/DMD/KCNA5/FLNA/CRYAB/PDLIM3/TPM2/ASB2/JPH2/LDB3/MYL9/FBXL22/ACTC1/FBXO32/CACNA1C/SMPX
CC	GO:0030017	sarcomere	2.73E-17	FERMT2/PGM5/ARHGEF25/SYNPO2/TPM1/ACTN2/FLNC/SYNC/MYOM1/LMOD1/ITGB1BP2/DES/ANK2/NEXN/ABCC9/CFL2/ACTA1/TMOD1/CSRP1/PPP1R12B/ANKRD1/CASQ2/DMD/KCNA5/FLNA/CRYAB/PDLIM3/TPM2/ASB2/JPH2/LDB3/MYL9/FBXL22/ACTC1/FBXO32/CACNA1C/SMPX
CC	GO:0031674	I band	6.25E-15	FERMT2/PGM5/SYNPO2/ACTN2/FLNC/SYNC/ITGB1BP2/DES/ANK2/NEXN/CFL2/CSRP1/PPP1R12B/ANKRD1/CASQ2/DMD/KCNA5/FLNA/CRYAB/PDLIM3/ASB2/JPH2/LDB3/MYL9/FBXL22/ACTC1/FBXO32/CACNA1C
CC	GO:0030018	Z disc	3.21E-13	PGM5/SYNPO2/ACTN2/FLNC/SYNC/ITGB1BP2/DES/ANK2/NEXN/CFL2/CSRP1/PPP1R12B/CASQ2/DMD/KCNA5/FLNA/CRYAB/PDLIM3/ASB2/JPH2/LDB3/MYL9/FBXL22/FBXO32/CACNA1C

CC	GO:0043025	neuronal cell body	2.83E-12	ROR2/SEZ6/TUBB4A/NPY/SLC8A2/GHR/GRIK5/GFRA1/HTR2A/NEGR1/NGFR/TAC1/SV2A/TMEM100/SYT4/CHRNA3/ITGA8/AKAP12/ATP1A2/GAP43/NDN/PDE9A/DNER/GIP/MAP1A/APOB/PDE1A/IGF2BP1/VIP/FLNA/CRYAB/DIXDC1/KCNK2/MAP1B/STMN2/CHRM2/APOD/RGS7BP/PCSK1/ADCYAP1/TNN/TTLL7/PRKAA2/ASCL1/CACNA1C/TGFB3/PENK/PTPRN/EPHA7
CC	GO:0005788	endoplasmic reticulum lumen	5.85E-12	SCG3/ERP27/IGFBP1/COL8A1/CES3/CDH2/FGA/APOA1/COL26A1/TNC/GPC3/AHSG/APOA2/BCHE/FKBP10/CES1/ITIH2/GIP/APOB/SPON1/CASQ2/F7/TF/FGG/SERPIND1/COL2A1/F2/SPARCL1/COL14A1/GCG/COL21A1/COL4A6/FMO1/AFP/PENK/CHRDL1/VTN
CC	GO:0042383	sarcolemma	5.33E-11	PGM5/SYNM/CACNG4/SLC8A2/KCNJ8/POPDC2/CDH2/FLNC/BVES/SYNC/SLC2A4/DES/ATP1A2/ANK2/PDE9A/DMD/SGCA/CACNB2/AKAP6/LAMA2/SGCD/CACNA1C/TGFB3
CC	GO:0043204	perikaryon	1.23E-07	NPY/SLC8A2/GRIK5/NGFR/TMEM100/ITGA8/GAP43/NDN/PDE9A/VIP/FLNA/CRYAB/MAP1B/RGS7BP/PCSK1/ADCYAP1/TTLL7/CACNA1C/PENK/PTPRN
CC	GO:0098636	protein complex involved in cell	1.85E-07	JAM3/ITGA7/JAM2/MMRN1/ITGBL1/ITGA8/TNC/TNXB/LAMA2/TNN/ITGA9/VTN
CC	GO:0015629	actin cytoskeleton	4.17E-07	SMTNL2/FERMT2/PGM5/JAM3/CD274/SVIL/SYNPO2/TPM1/ACTN2/NTN1/CDH2/AMPH/CALD1/DPYSL3/HSPB7/ACTA2/LMOD1/MYH11/FILIP1/NEXN/CDC42EP3/CNR1/CFL2/ACTA1/TMOD1/PKNOX2/SMTN/FLNA/CRYAB/PDLIM3/TPM2/LDB3/MYL9/ACTC1/MYLK/EPHA3/DDR2/SORBS1
CC	GO:0043034	costamere	7.48E-07	PGM5/SYNM/SVIL/FLNC/ANK2/DMD/SMPX
CC	GO:0072562	blood microparticle	7.65E-07	HP/ACTG2/FGA/APOA1/F13A1/AHSG/APOA2/BCHE/ACTA1/ITIH2/AGT/TF/FGG/SERPINF2/F2/ACTC1/HSPA2/VTN
CC	GO:0032432	actin filament bundle	9.97E-07	FERMT2/PGM5/SYNPO2/TPM1/ACTA2/ACTA1/FLNA/CRYAB/PDLIM3/LDB3/MYL9/MYLK/SORBS1
CC	GO:0031983	vesicle lumen	2.22E-06	SCG3/RNASE2/IGF1/HP/PRTN3/ACTN2/MMRN1/FGA/APOA1/F13A1/CTSG/AHSG/GIP/APOB/TTR/GRP/TF/IGF2/MPO/ELANE/FGG/SERPINF2/AZU1/GCG/PCSK1/TGFB3/PENK/PTX3
CC	GO:0005604	basement membrane	2.38E-06	THBS4/FREM1/COL8A1/FBLN1/ACTA2/CCDC80/TNC/SMOC2/COL2A1/COL4A6/LAMA2/HMCN1/VTN

CC	GO:0034774	secretory granule lumen	4.98E-06	SCG3/RNASE2/IGF1/HP/PRTN3/ACTN2/MMRN1/FGA/APOA1/F13A1/CTSG/AHSG/GIP/TTR/GRP/TF/IGF2/MPO/ELANE/FGG/SERPINF2/AZU1/GCG/PCSK1/TGFB3/PENK/PTX3
CC	GO:0060205	cytoplasmic vesicle lumen	5.91E-06	SCG3/RNASE2/IGF1/HP/PRTN3/ACTN2/MMRN1/FGA/APOA1/F13A1/CTSG/AHSG/GIP/TTR/GRP/TF/IGF2/MPO/ELANE/FGG/SERPINF2/AZU1/GCG/PCSK1/TGFB3/PENK/PTX3
CC	GO:0098637	protein complex involved in	9.29E-06	MMRN1/TNC/TNXB/LAMA2/TNN/VTN
CC	GO:0001725	stress fiber	1.06E-05	FERMT2/PGM5/SYNPO2/TPM1/ACTA2/ACTA1/PDLIM3/LDB3/MYL9/MYLK/SORBS1
CC	GO:0097517	contractile actin filament	1.06E-05	FERMT2/PGM5/SYNPO2/TPM1/ACTA2/ACTA1/PDLIM3/LDB3/MYL9/MYLK/SORBS1
CC	GO:0034703	cation channel complex	1.27E-05	CACNA1H/CACNG4/TRPC4/KCNJ8/GRIK5/SCN7A/ABCC9/CASQ2/KCNA5/KCNMB1/KCNK2/KCNMA1/CACNB2/AKAP6/SCN4B/KCNH2/TRPC1/HSPA2/CACNA1C
CC	GO:0005581	collagen trimer	1.53E-05	COLEC12/COL8A1/ADIPOQ/C1QTNF2/COL26A1/C1QTNF7/COL2A1/SCARA3/COL14A1/COL21A1/COL4A6/COLEC11
CC	GO:0044853	plasma membrane raft	1.57E-05	TRPC4/HTR2A/PRTN3/CDH2/BVES/CHRNA3/ATP1A2/GASK1A/KCNA5/KCNMA1/AKAP6/PTGIS/CD36/SORBS1
CC	GO:0030055	cell-substrate junction	2.36E-05	FERMT2/PGM5/SVIL/TGFB1I1/LIMS2/LAYN/SYNPO2/TNS4/ACTN2/CDH2/FLNC/FHL1/PHLDB2/ITGBL1/ITGA8/TNC/AKAP12/NEXN/TNS1/CNN1/CSRP1/DMD/NFASC/FLNA/DIXDC1/ACTC1/FLRT2/HMCN1/SMPX/DDR2/SORBS1
CC	GO:0042641	actomyosin	2.71E-05	FERMT2/PGM5/SYNPO2/TPM1/ACTA2/ACTA1/PDLIM3/LDB3/MYL9/MYLK/SORBS1
CC	GO:0005884	actin filament	2.88E-05	SMTNL2/JAM3/TPM1/ACTN2/DPYSL3/LMOD1/ACTA1/TMOD1/SMTN/PDLIM3/TPM2/LDB3/ACTC1
CC	GO:0031093	platelet alpha granule lumen	4.78E-05	IGF1/ACTN2/MMRN1/FGA/F13A1/AHSG/IGF2/FGG/SERPINF2/TGFB3
CC	GO:0098978	glutamatergic synapse	5.57E-05	ROR2/CNTNAP1/CACNG4/ADCY1/KCNJ8/GRIK5/HTR2A/PTPRD/SV2A/SYT4/FXYD6/NRXN2/ITGA8/PLPPR4/NLGN4X/CNR1/NPTX2/GUCY1A1/NPTXR/NPTX1/ADAM23/NLGN4Y/WASF3/PLXNA4/SPARCL1/CHRM2/RGS7BP/ACTC1/EPHA7
CC	GO:0005901	caveola	6.25E-05	TRPC4/HTR2A/BVES/ATP1A2/GASK1A/KCNA5/KCNMA1/AKAP6/PTGIS/CD36/SORBS1
CC	GO:0044306	neuron projection terminus	6.34E-05	NPY/SLC8A2/GRIK5/UCN3/DMD/SCGN/DIXDC1/KCNK2/CHRM2/PCSK1/TNN/PENK/SCRG1/PTPRN

CC	GO:0044291	cell-cell contact zone	8.97E-05	PGM5/JAM3/JAM2/CDH2/DES/ATP1A2/ANK2/KCNA5/AKAP6/SCN4B
CC	GO:0036379	myofilament	0.000135	TPM1/MYOM1/LMOD1/ACTA1/TMOD1/TPM2
CC	GO:0031091	platelet alpha granule	0.000146	IGF1/ACTN2/MMRN1/FGA/F13A1/AHSG/IGF2/FGG/SERPINF2/TGFB3/CD36
CC	GO:0014704	intercalated disc	0.000166	PGM5/CDH2/DES/ATP1A2/ANK2/KCNA5/AKAP6/SCN4B
CC	GO:0031941	filamentous actin	0.000169	SMTNL2/JAM3/DPYSL3/SMTN/PDLIM3/LDB3
CC	GO:0090533	cation-transporting	0.000195	PLN/KCNJ8/ATP1B2/ATP1A2/ABCC9
CC	GO:0005925	focal adhesion	0.000218	FERMT2/PGM5/SVIL/TGFB1I1/LIMS2/LAYN/SYNPO2/TNS4/ACTN2/CDH2/FLNC/FHL1/PHLDB2/ITGBL1/ITGA8/TNC/AKAP12/NEXN/TNS1/CNN1/CSRP1/NFASC/FLNA/DIXDC1/ACTC1/FLRT2/DDR2/SORBS1
CC	GO:0030315	T-tubule	0.00022	CACNG4/SLC2A4/ATP1A2/ANK2/CACNB2/AKAP6/CACNA1C/TGFB3
CC	GO:0150034	distal axon	0.000227	NPY/SLC8A2/GRIK5/NGFR/DPYSL3/UCN3/CNR1/GAP43/IGF2BP1/SCGN/FLNA/DIXDC1/KCNK2/MAP1B/STMN2/CHRM2/BOC/PCSK1/TNN/PENK/PTPRN
CC	GO:0043679	axon terminus	0.000278	NPY/SLC8A2/GRIK5/UCN3/SCGN/DIXDC1/KCNK2/CHRM2/PCSK1/TNN/PENK/PTPRN
CC	GO:0032280	symmetric	0.00028	NLGN4X/NLGN4Y/CHRM2/PENK
CC	GO:0045121	membrane raft	0.00029	ADCY1/TRPC4/HTR2A/PRTN3/CDH2/BVES/CLIP3/SLC2A4/CHRNA3/ATP1A2/ANK2/CNR1/GASK1A/DMD/KCNA5/SGCA/KCNMA1/GPM6B/AKAP6/CNTN1/PTGIS/CD36/SORBS1
CC	GO:0098857	membrane microdomain	0.000303	ADCY1/TRPC4/HTR2A/PRTN3/CDH2/BVES/CLIP3/SLC2A4/CHRNA3/ATP1A2/ANK2/CNR1/GASK1A/DMD/KCNA5/SGCA/KCNMA1/GPM6B/AKAP6/CNTN1/PTGIS/CD36/SORBS1
CC	GO:0097060	synaptic membrane	0.000337	CADM3/CACNG4/ADCY1/KCNJ8/GRIK5/HTR2A/PTPRD/CDH2/FXYD6/CHRNA3/ITGA8/ANK2/PLPPR4/NLGN4X/CNR1/GLRB/DMD/CRYAB/ADAM23/NLGN4Y/KCNMA1/CHRM2/CNTN1/RGS7BP/CACNA1C/EPHA7
CC	GO:0005865	striated muscle	0.000539	TPM1/LMOD1/ACTA1/TMOD1/TPM2
CC	GO:0005796	Golgi lumen	0.000558	OGN/MUC5AC/DEFA6/GPC3/PRELP/F7/DCN/OMD/F2/NGF/VTN
CC	GO:0098984	neuron to neuron synapse	0.000571	CACNG4/SLC8A2/ADCY1/GRIK5/PTPRD/CDH2/NGFR/ATP1B2/CHRNA3/MPDZ/ITGA8/PLPPR4/NLGN4X/GAP43/CRYAB/MAP1B/NLGN4Y/PLXNA4/CHRM2/RGS7BP/PSD/CACNA1C/PENK/CAP2/EPHA7

CC	GO:0045211	postsynaptic membrane	0.000593	CACNG4/ADCY1/GRIK5/HTR2A/CDH2/FXYD6/CHRNA3/ITGA8/ANK2/PLPPR4/NLGN4X/GLRB/DMD/NLGN4Y/KCNMA1/CHRM2/CNTN1/RGS7BP/CACNA1C/EPHA7
CC	GO:0034702	ion channel complex	0.000628	CACNA1H/CACNG4/TRPC4/KCNJ8/GRIK5/SCN7A/CHRNA3/ABCC9/GLRB/CASQ2/KCNA5/KCNMB1/KCNK2/KCNMA1/CACNB2/AKAP6/SCN4B/KCNH2/TRPC1/HSPA2/CACNA1C
CC	GO:0090665	glycoprotein	0.000671	PGM5/DMD/FLNA/SGCA/SGCD
CC	GO:0098533	ATPase dependent transmembrane	0.000825	PLN/KCNJ8/ATP1B2/ATP1A2/ABCC9
CC	GO:0034704	calcium channel complex	0.000893	CACNA1H/CACNG4/TRPC4/SCN7A/CASQ2/CACNB2/AKAP6/HSPA2/CACNA1C
CC	GO:0097386	glial cell projection	0.001174	SCN7A/ATP1B2/SYT4/NFASC/KCNK2/WASF3
CC	GO:0098839	postsynaptic density membrane	0.001523	CACNG4/ADCY1/GRIK5/ITGA8/PLPPR4/NLGN4X/NLGN4Y/RGS7BP/CACNA1C/EPHA7
CC	GO:0098992	neuronal dense	0.001718	CHGA/NPY/SYT4/GRP/PENK
CC	GO:1902495	transmembrane transporter complex	0.001803	CACNA1H/PLN/CACNG4/TRPC4/KCNJ8/GRIK5/SCN7A/ATP1B2/CHRNA3/ATP1A2/ABCC9/GLRB/CASQ2/KCNA5/KCNMB1/KCNK2/KCNMA1/CACNB2/AKAP6/SCN4B/KCNH2/TRPC1/HSPA2/CACNA1C
CC	GO:0016010	dystrophin-associated	0.002176	PGM5/DMD/SGCA/SGCD
CC	GO:0016529	sarcoplasmic reticulum	0.002189	THBS4/PLN/IRAG1/SLC2A4/CASQ2/JPH2/AKAP6/SGCD
CC	GO:0031594	neuromuscular junction	0.002189	NGFR/SV2A/SYNC/DES/LAMA2/PDZRN3/TUBA1A/EPHA7
CC	GO:0032279	asymmetric synapse	0.002207	CACNG4/SLC8A2/ADCY1/GRIK5/CDH2/NGFR/CHRNA3/MPDZ/ITGA8/PLPPR4/NLGN4X/GAP43/CRYAB/MAP1B/NLGN4Y/PLXNA4/CHRM2/RGS7BP/PSD/CACNA1C/CAP2/EPHA7
CC	GO:0099634	postsynaptic specialization membrane	0.002777	CACNG4/ADCY1/GRIK5/CDH2/ITGA8/PLPPR4/NLGN4X/NLGN4Y/RGS7BP/CACNA1C/EPHA7
CC	GO:0031430	M band	0.003275	MYOM1/ANK2/CRYAB/SMPX
CC	GO:0034361	very-low-density lipoprotein	0.003275	APOA1/APOC3/APOA2/APOB
CC	GO:0034385	triglyceride-rich plasma	0.003275	APOA1/APOC3/APOA2/APOB
CC	GO:0031672	A band	0.00364	MYOM1/ANK2/PPP1R12B/CRYAB/SMPX
CC	GO:1990351	transporter complex	0.004099	CACNA1H/PLN/CACNG4/TRPC4/KCNJ8/GRIK5/SCN7A/ATP1B2/CHRNA3/ATP1A2/ABCC9/GLRB/CASQ2/KCNA5/KCNMB1/KCNK2/KCNMA1/CACNB2/AKAP6/SCN4B/KCNH2/TRPC1/HSPA2/CACNA1C
CC	GO:0044304	main axon	0.004123	CNTNAP1/TUBB4A/UCN3/MAP1A/NFASC/MAP1B/MYOC

CC	GO:0005775	vacuolar lumen	0.004232	OGN/RNASE2/PRTN3/CTSG/GPC3/PRELP/APOB/TTR/MPO/ELANE/AZU1/DCN/OMD
CC	GO:0005614	interstitial	0.004369	CCDC80/TNC/COL14A1
CC	GO:0098982	GABA-ergic synapse	0.005242	NPY/SV2A/NLGN4X/CNR1/GAP43/GLRB/GUCY1A1/NLGN4Y
CC	GO:0071682	endocytic	0.005551	HP/APOA1/APOB/MPO
CC	GO:0042627	chylomicron	0.005689	APOC3/APOA2/APOB
CC	GO:1990454	L-type voltage-gated calcium	0.005689	CACNG4/CACNB2/CACNA1C
CC	GO:0014069	postsynaptic density	0.006255	CACNG4/SLC8A2/ADCY1/GRIK5/CDH2/NGFR/CHRNA3/MPDZ/ITGA8/PLPPR4/NLGN4X/GAP43/CRYAB/MAP1B/NLGN4Y/RGS7BP/PSD/CACNA1C/CAP2/EPHA7
CC	GO:0042734	presynaptic membrane	0.006316	CADM3/KCNJ8/GRIK5/HTR2A/PTPRD/CDH2/FXYD6/CNR1/ADAM23/CHRM2/CNTN1/RGS7BP
CC	GO:0099572	postsynaptic specialization	0.006617	CACNG4/SLC8A2/ADCY1/GRIK5/CDH2/NGFR/CHRNA3/MPDZ/ITGA8/PLPPR4/NLGN4X/GAP43/GLRB/CRYAB/MAP1B/NLGN4Y/RGS7BP/PSD/CACNA1C/CAP2/EPHA7
CC	GO:0034705	potassium channel complex	0.007403	KCNJ8/GRIK5/ABCC9/KCNA5/KCNMB1/KCNK2/KCNMA1/KCNH2
CC	GO:0005891	voltage-gated calcium channel	0.007825	CACNA1H/CACNG4/SCN7A/CACNB2/HSPA2/CACNA1C
CC	GO:0016528	sarcoplasm	0.007906	THBS4/PLN/IRAG1/SLC2A4/CASQ2/JPH2/AKAP6/SGCD
CC	GO:0031045	dense core	0.009336	CHGA/NPY/SYT4/GRP/PENK
CC	GO:0034364	high-density lipoprotein	0.009954	APOA1/APOC3/APOA2/APOB
CC	GO:0045178	basal part of cell	0.010082	MPZ/PTH1R/SLCO1B3/SLC8A2/TRPC4/CDH2/GPIHBP1/ABCA8/PHLDB2/ANK2/LEPR/SLC38A3/CA9/TF/AQP8/CLDN11/ITGA9
CC	GO:0098985	asymmetric, glutamatergic,	0.013175	NLGN4X/NLGN4Y/PLXNA4
MF	GO:0005201	extracellular matrix structural constituent	4.46E-18	TFPI2/OGN/COL8A1/FBLN1/DPT/PODN/MMRN1/MUC5AC/CILP/ADIPOQ/FGA/ASPN/SRPX/ABI3BP/TNC/ELN/PRELP/SPON1/EFEMP1/FBLN5/MFAP5/FGG/DCN/COL2A1/COL14A1/COL21A1/COL4A6/TNXB/MFAP4/LAMA2/MGP/PRG4/HMCN1/VTN
MF	GO:0005539	glycosaminoglycan binding	3.72E-12	THBS4/FGF2/PTN/LAYN/ADAMTS8/DPYSL3/SFRP1/PCOLCE2/CCDC80/FGF10/CTSG/RSPO3/SLIT3/FGF7/PRELP/ITIH2/APOB/SUSD5/MPO/SMOC2/ELANE/SERPIND1/AZU1/DCN/F2/GREM2/RSPO2/TNXB/SLIT2/RSPO1/LYVE1/VTN
MF	GO:0008201	heparin binding	2.94E-11	THBS4/FGF2/PTN/ADAMTS8/SFRP1/PCOLCE2/CCDC80/FGF10/CTSG/RSPO3/SLIT3/FGF7/PRELP/APOB/MPO/SMOC2/ELANE/SERPIND1/AZU1/F2/GREM2/RSPO2/TNXB/SLIT2/RSPO1/VTN

MF	GO:0005178	integrin binding	9.64E-11	THBS4/FERMT2/JAM3/FGF2/ITGA7/JAM2/IGF1/PTN/FBLN1/ADAMTS8/GFRA1/ACTN2/ITGB1BP2/ITGBL1/ITGA8/SFRP2/IGF2/FBLN5/ADAM23/TNXB/TNN/CXCL12/ITGA9/VTN
MF	GO:0048018	receptor ligand activity	1.29E-10	THBS4/OGN/C1QTNF4/ALKAL1/NPY/FNDC5/FGF2/IGF1/PTN/RETNLB/ADIPOQ/APOA1/AMH/FGF10/CTSG/CARTPT/TNFSF9/FGF7/UCN3/SFRP2/GIP/SEMA3E/CCL21/TTR/VIP/AGT/GRP/EFEMP1/IGF2/SCT/F2/NGF/WNT9A/GCG/GREM2/THPO/BMP3/FLRT2/ADCYAP1/CXCL12/GDF10/TGFB3/PENK/IL33/EPHA7/GREM1
MF	GO:1901681	sulfur compound binding	3.35E-09	THBS4/FGF2/PTN/ADAMTS8/DPYSL3/SFRP1/PCOLCE2/CCDC80/FGF10/CTSG/RSPO3/SLIT3/FGF7/PRELP/APOB/TF/MPO/SMOC2/ELANE/SERPIND1/AZU1/F2/SOAT2/GREM2/SULT2A1/RSPO2/TNXB/SLIT2/RSPO1/VTN
MF	GO:0005179	hormone activity	1.23E-08	NPY/FNDC5/IGF1/RETNLB/ADIPOQ/AMH/CARTPT/UCN3/GIP/TTR/VIP/AGT/GRP/IGF2/SCT/GCG/THPO/ADCYAP1/PENK
MF	GO:0008307	structural constituent of	6.03E-08	SYNM/TPM1/ACTN2/MYOM1/MYH11/NEXN/CSRP1/DMD/SMTN/TPM2/MYL9
MF	GO:0003779	actin binding	1.78E-06	FERMT2/SVIL/SYNPO2/TPM1/TNS4/ACTN2/FLNC/CALD1/LMOD1/MYH11/NEXN/TNS1/TAGLN/CFL2/TMOD1/CNN1/MAP1A/PKNOX2/DMD/SMTN/FLNA/PDLIM3/DIXDC1/CORO6/TPM2/MAP1B/KCNMA1/WASF3/CACNB2/LDB3/DAAM2/MYLK/CAP2/SORBS1
MF	GO:0008083	growth factor activity	4.13E-06	THBS4/OGN/FGF2/IGF1/PTN/AMH/FGF10/FGF7/AGT/EFEMP1/IGF2/F2/NGF/THPO/BMP3/CXCL12/GDF10/TGFB3
MF	GO:0030021	extracellular matrix structural	5.19E-05	OGN/PODN/ASPN/PRELP/DCN/PRG4
MF	GO:0042562	hormone binding	7.70E-05	PTH1R/GHR/PDE3A/GLP2R/NPR3/ATP1A2/AR/TTR/CCKAR/VIPR2/MLNR
MF	GO:0022843	voltage-gated cation channel activity	0.000172	CACNA1H/KCNE4/CACNG4/KCNK3/KCNJ8/SCN7A/ABCC9/KCNH6/KCNA5/KCNK2/KCNMA1/CACNB2/KCNH2/CACNA1C
MF	GO:0019955	cytokine binding	0.00029	FGF2/GHR/LEPR/CHRDL2/NOG/ELANE/ACKR1/GREM2/CHRD/TGFB3/CD36/GREM1/CHRDL1
MF	GO:0005184	neuropeptide	0.000332	NPY/CARTPT/VIP/GRP/ADCYAP1/PENK
MF	GO:0050840	extracellular matrix binding	0.000351	SSC5D/ITGA7/ELN/SMOC2/DCN/SPARCL1/SLIT2/VTN
MF	GO:0036122	BMP binding	0.000352	CHRDL2/GREM2/CHRD/GREM1/CHRDL1
MF	GO:0008179	adenylate	0.000427	AKAP12/ADCY5/AKAP6/CAP2

MF	GO:0001664	G protein-coupled receptor binding	0.000462	NPY/PTCH2/REEP1/SFRP1/TUB/RSPO3/REEP2/UCN3/GIP/CCL21/AGT/FLNA/SCT/WNT9A/GCG/MYOC/AGTR1/RSPO1/GNAO1/CXCL12/PENK
MF	GO:0017147	Wnt-protein	0.00048	ROR2/SFRP1/SFRP2/FRZB/SFRP4/RECK
MF	GO:0005044	scavenger receptor	0.000806	SSC5D/COLEC12/DMBT1/SCARA3/PRG4/CD36/VTN
MF	GO:0042805	actinin binding	0.000925	SYNPO2/CSRP1/KCNA5/PDLIM3/LDB3/CACNA1C
MF	GO:0005244	voltage-gated ion channel activity	0.001209	CACNA1H/KCNE4/CACNG4/KCNK3/KCNJ8/SCN7A/ABCC9/KCNH6/KCNA5/KCNK2/KCNMA1/CACNB2/SCN4B/KCNH2/CACNA1C
MF	GO:0022832	voltage-gated channel activity	0.001209	CACNA1H/KCNE4/CACNG4/KCNK3/KCNJ8/SCN7A/ABCC9/KCNH6/KCNA5/KCNK2/KCNMA1/CACNB2/SCN4B/KCNH2/CACNA1C
MF	GO:0038024	cargo receptor activity	0.001374	SSC5D/COLEC12/DMBT1/SCARA3/PRG4/LYVE1/CD36/VTN/ASGR2
MF	GO:0086008	voltage-gated potassium channel activity involved in	0.001418	KCNE4/KCNJ8/KCNA5/KCNH2
MF	GO:0004857	enzyme inhibitor activity	0.001421	TFPI2/PLN/SLCO1B3/PTN/ITIH5/PI16/APOC3/SCG5/GPC3/AHSG/APOA2/PPP1R14A/PPP1R1A/SERPINA6/ITIH2/PPP1R12B/SERPINF2/SERPIND1/NGF/RECK/SLIT2/PI15/WARS1
MF	GO:0051393	alpha-actinin	0.001803	SYNPO2/KCNA5/PDLIM3/LDB3/CACNA1C
MF	GO:0005267	potassium channel activity	0.001821	KCNE4/KCNK3/KCNJ8/GRIK5/ABCC9/KCNH6/KCNA5/KCNMB1/KCNK2/KCNMA1/KCNH2
MF	GO:0005261	cation channel activity	0.001955	CACNA1H/KCNE4/CACNG4/KCNK3/TRPC4/KCNJ8/GRIK5/SCN7A/CHRNA3/TMEM63C/ABCC9/KCNH6/KCNA5/KCNMB1/KCNK2/KCNMA1/CACNB2/SCN4B/KCNH2/TRPC1/CACNA1C
MF	GO:0005249	voltage-gated potassium	0.002074	KCNE4/KCNK3/KCNJ8/ABCC9/KCNH6/KCNA5/KCNK2/KCNMA1/KCNH2
MF	GO:0005516	calmodulin binding	0.002114	PNCK/SLC8A2/ADCY1/NGFR/CALD1/MYH11/AKAP12/GAP43/CNN1/PDE1A/RGS4/MAP6/PCP4/MYLK/CACNA1C
MF	GO:0070325	lipoprotein particle	0.002121	RELN/APOA1/APOC3/APOA2/APOB
MF	GO:0008528	G protein-coupled peptide receptor	0.002148	PTH1R/SSTR5/GRPR/GLP2R/NPR3/TACR1/CCKAR/NPSR1/TACR2/AGTR1/VIPR2/MLNR
MF	GO:0022836	gated channel activity	0.002424	CACNA1H/KCNE4/CACNG4/KCNK3/KCNJ8/GRIK5/SCN7A/CHRNA3/TMEM63C/ABCC9/KCNH6/GLRB/KCNA5/KCNMB1/KCNK2/KCNMA1/CACNB2/SCN4B/KCNH2/CACNA1C

MF	GO:0022839	ion gated channel activity	0.002424	CACNA1H/KCNE4/CACNG4/KCNK3/KCNJ8/GRIK5/SCN7A/CHRNA3/TMEM63C/ABCC9/KCNH6/GLRB/KCNA5/KCNMB1/KCNK2/KCNMA1/CACNB2/SCN4B/KCNH2/CACNA1C
MF	GO:0071813	lipoprotein	0.002479	COLEC12/APOA1/GPIHBP1/APOA2/CD36
MF	GO:0071814	protein-lipid	0.002479	COLEC12/APOA1/GPIHBP1/APOA2/CD36
MF	GO:0051428	peptide hormone	0.002797	UCN3/VIP/GNAO1/ADCYAP1
MF	GO:0001968	fibronectin	0.002879	SSC5D/FBLN1/CCDC80/SFRP2/MYOC
MF	GO:0051427	hormone receptor	0.002879	FABP4/UCN3/VIP/GNAO1/ADCYAP1
MF	GO:0001653	peptide receptor activity	0.003028	PTH1R/SSTR5/GRPR/GLP2R/NPR3/TACR1/CCKAR/NPSR1/TACR2/AGTR1/VIPR2/MLNR
MF	GO:0061134	peptidase regulator activity	0.003257	TFPI2/SLCO1B3/FBLN1/ITIH5/PCOLCE2/PI16/GPC3/AHSG/SFRP2/SERPINA6/ITIH2/SERPINF2/SERPIND1/NGF/RECK/PI15
MF	GO:0030023	extracellular matrix	0.003358	MMRN1/ELN/FBLN5
