# Supplementary material for: Prognostic value of disulfidptosis-associated genes in gastric cancer: a comprehensive analysis
Source: Front Oncol. 2025 Mar 4;15:1512394. doi: 10.3389/fonc.2025.1512394 (PMC11913695; doi:10.3389/fonc.2025.1512394)
Supplement: Supplementary file 2 [file DataSheet2.pdf]

ID	Description	pvalue	geneID	Count
hsa04820	Cytoskeleton in muscle cells	3.07E-13	THBS4/ITGA7/SYNPO2/TPM1/ACTN2/FLNC/ACTG2/ATP1B2/FHL1/MYOM1/LMOD1/MYH11/DES/ITGA8/ATP1A2/ANK2/ELN/DTNA/ACTA1/TMOD1/CSRP1/ANKRD1/DMD/PDLIM3/SGCA/TPM2/DCN/LDB3/COL4A6/MYL9	35
hsa05414	Dilated cardiomyopathy	1.69E-11	PLN/CACNG4/SLC8A2/ITGA7/IGF1/ADCY1/TPM1/DES/ITGA8/DTNA/AGT/DMD/SGCA/TPM2/ADCY5/CACNB2/LAMA2/ACTC	22
hsa05410	Hypertrophic cardiomyopathy	2.90E-10	CACNG4/SLC8A2/ITGA7/IGF1/TPM1/DES/ITGA8/DTNA/AGT/DMD/SGCA/TPM2/CACNB2/LAMA2/ACTC1/SGCD/PRKAA2/CA	20
hsa04080	Neuroactive ligand-receptor interaction	1.29E-08	ADRA1D/HTR2B/PTH1R/NPY/SSTR5/GRPR/GHR/PTGER3/GRIK5/HTR2A/GLP2R/TAC1/CHRNA3/CTSG/LEPR/UCN3/CNR1/TACR1/GIP/GLRB/TAC3/VIP/AGT/GRP/CCKAR/PTGFR/NPSR1/SCT/TACR2/F2/CHRM2/GCG/	37
hsa04270	Vascular smooth muscle contraction	1.31E-08	ADRA1D/ADCY1/PLA2G3/IRAG1/ACTG2/CALD1/PRKG1/ACTA2/MYH11/PPP1R14A/PLA2G5/PPP1R12B/AGT/KCNMB1/GUCY1A1/ADCY5/KCNMA1/MYL9/AG	21
hsa04261	Adrenergic signaling in cardiomyocytes	1.57E-07	ADRA1D/PLN/CACNG4/SLC8A2/ADCY1/POPDC2/TPM1/BVES/SCN7A/ATP1B2/ATP1A2/PPP1R1A/AGT/KCNK2/TPM2/ADCY5/CACNB2/SCN4B/AGTR1/ACTC	21
hsa05412	Arrhythmogenic right ventricular cardiomyopathy	3.93E-07	CACNG4/SLC8A2/ITGA7/ACTN2/CDH2/DES/ITGA8/DTNA/DMD/SGCA/CACNB2/LAMA2/SGCD	15
hsa04512	ECM-receptor interaction	6.24E-07	THBS4/FREM1/RELN/ITGA7/SV2A/ITGA8/TNC/COL2A1/COL4A6/TNXB/LAMA2/TNN/CD36/	15
hsa04972	Pancreatic secretion	7.22E-07	ADCY1/PNLIP/PLA2G3/ATP1B2/ATP1A2/CTRB1/PLA2G5/CPA2/PNLIPRP2/CCKAR/ADCY5/SCT/KCNMA1/PNLIPRP1/TRPC	16
hsa04514	Cell adhesion molecules	3.88E-06	MPZ/CNTNAP1/JAM3/CD274/CADM3/JAM2/PTPRD/CDH2/NEGR1/NRXN2/ITGA8/NLGN4X/CLDN6/NFASC/NLGN4Y/CNTN1/C	19
hsa04022	cGMP-PKG signaling pathway	8.85E-06	ADRA1D/PLN/SLC8A2/ADCY1/KCNJ8/PDE3A/IRAG1/PRKG1/ATP1B2/ATP1A2/NFATC4/KCNMB1/GUCY1A1/ADCY5/KCNMA1	19

hsa04020	Calcium signaling pathway	1.57E-05	CACNA1H/ADRA1D/HTR2B/GRPR/PLN/SLC8A2/FGF2/ADCY1/PTGER3/HTR2A/FGF10/FGF7/TACR1/PDE1A/NFATC4/CASQ2/CCKAR/PTGFR/TACR2/NGF/C	24
hsa04024	cAMP signaling pathway	2.23E-05	NPY/SSTR5/PLN/GLI3/ADCY1/POPDC2/PTGER3/PDE3A/BVES/AMH/ATP1B2/ATP1A2/GIP/VIP/KCNK2/ADCY5/CHRM2/GC	22
hsa04974	Protein digestion and absorption	8.61E-05	SLC8A2/COL8A1/ATP1B2/COL26A1/ATP1A2/CTRB1/ELN/CPA2/COL2A1/COL14A1/COL21A	13
hsa04975	Fat digestion and absorption	0.000134	PNLIP/PLA2G3/APOA1/PLA2G5/APOB/PNLIPRP2/PNLIPRP1	8
hsa04911	Insulin secretion	0.000267	ADCY1/ATP1B2/ATP1A2/GIP/KCNMB1/CCKAR/ADCY5/KCNMA1/GCG/ADCYAP1/CACNA1C	11
hsa04260	Cardiac muscle contraction	0.000296	CACNG4/SLC8A2/TPM1/ATP1B2/ATP1A2/CASQ2/TPM2/CACNB2/ACTC1/COX7A1/CACNA1C	11
hsa04924	Renin secretion	0.000829	PDE3A/PDE1A/AGT/GUCY1A1/ADCY5/KCNMA1/AGTR1/ADCYA	9
hsa04510	Focal adhesion	0.001093	THBS4/RELN/ITGA7/IGF1/ITGA8/TNC/PPP1R12B/FLNA/COL2A1/COL4A6/MYL9/TNXB/LAMA2/TNN/MYLK/ITGA9/VTN	17
hsa03320	PPAR signaling pathway	0.001517	ADIPOQ/APOA1/FABP4/APOC3/PLIN4/APOA2/PLIN1/CD36/	9
hsa04970	Salivary secretion	0.001965	ADRA1D/ADCY1/MUC5AC/PRKG1/ATP1B2/DMBT1/ATP1A2/GUCY1A1/ADCY5/KCNMA1	10
hsa04927	Cortisol synthesis and secretion	0.002336	CACNA1H/KCNK3/ADCY1/AGT/KCNK2/ADCY5/AGTR1/CACNA1	8
hsa04360	Axon guidance	0.002593	TRPC4/RGMA/NTN1/SLIT3/CFL2/SEMA3E/NFATC4/PLXNA4/BOC/MYL9/TRPC1/SLIT2/CXC	15
hsa04925	Aldosterone synthesis and secretion	0.002904	CACNA1H/KCNK3/ADCY1/ATP1B2/ATP1A2/PRKD1/AGT/ADCY5/AGTR1/CACNA1C	10
hsa04610	Complement and coagulation cascades	0.003921	FGA/F13A1/C7/F7/FGG/SERPINF2/SERPIND1/F2/VTN	9
hsa04540	Gap junction	0.004573	HTR2B/TUBB4A/TUBA3C/ADCY1/HTR2A/PRKG1/GUCY1A1/AD	9
hsa04923	Regulation of lipolysis in	0.004839	NPY/ADCY1/PTGER3/PRKG1/FABP4/PLIN1/ADCY5	7
