# Supplementary material for: Prognostic value of disulfidptosis-associated genes in gastric cancer: a comprehensive analysis
Source: Front Oncol. 2025 Mar 4;15:1512394. doi: 10.3389/fonc.2025.1512394 (PMC11913695; doi:10.3389/fonc.2025.1512394)
Supplement: Supplementary file 3 [file DataSheet3.pdf]

| ID | Description | pvalue | core_enrichment |
|---|---|---|---|
| KEGG_NEUROACTIVE_LIGAND_RECEPTOR_INTERACTION | KEGG_NEUROACTIVE_LIGAND_RECEPTOR_INTERACTION | 2.08E-09 | CHRM2/AGTR1/TACR2/GRIK3/CHRNA3/PRSS1/MLNR/TACR1/ADCYAP1R1/GRIK1/CTSG/NPFFR2/GLP2R/GRPR/GRIN2A/F2/HTR2A/GHR/GRIK5/HTR2B/GLRB/PTGFR/CCKAR/ADRA1D/P2RX2/PTGER3/GABBR2/CNR1/PTH1R/VIPR2/PRSS2/LEPR/LPAR4/ADRA1B/CHRNB4/P2RX1/LPAR3/EDNRA/CNR2/CHRM4/LPAR1/S1PR3/NMUR1/BDKRB1/P2RY14/AVPR1A/CYSLTR1/SCTR/CGA/ADRB2/CCKBR/GLP1R/OXTR/THRB/SSTR2/PTGIR/S1PR1/AVPR2/PTGER1/FPR1/GABBR1/NR3C1/GABRR1/ADORA3/CHRNB2/P2RY1/GABRE/PTGDR/S1PR5/UTS2R/EDNRB/TAAR1/APLNR/PRLR/FPR2/GRID1 |
| KEGG_CALCIUM_SIGNALING_PATHWAY | KEGG_CALCIUM_SIGNALING_PATHWAY | 9.97E-09 | CHRM2/AGTR1/TACR2/PLN/MYLK/TACR1/PLCD4/GRPR/GRIN2A/SLC8A2/HTR2A/RYR3/HTR2B/ADCY2/PTGFR/CCKAR/CACNA1C/ADRA1D/P2RX2/PTGER3/TRPC1/PDE1A/CACNA1H/ADCY1/CALML3/ADRA1B/P2RX1/ATP2B4/EDNRA/CALML5/PDE1C/PDE1B/SLC8A1/BDKRB1/GNAL/PRKCB/CACNA1A/AVPR1A/CYSLTR1/PDGFRA/ADRB2/CCKBR/OXTR/PHKG1/PTGER1/BST1/ITPR1/RYR2 |
| KEGG_DILATED_CARDIOMYOPATHY | KEGG_DILATED_CARDIOMYOPATHY | 5.77E-08 | DES/PLN/SGCA/ADCY5/TPM2/IGF1/DMD/MYL3/ADCY2/ITGA7/CACNA1C/SGCD/SGCG/CACNG4/LAMA2/ADCY1/CACNB2/TPM1/ACTC1/ITGA8/ITGA9/TGFB3/TGFB2/ITGA1/SLC8A1/ITGA5/CACNA2D3/CACNA2D1 |
| KEGG_VASCULAR_SMOOTH_MUSCLE_CONTRACTION | KEGG_VASCULAR_SMOOTH_MUSCLE_CONTRACTION | 6.70E-08 | PLA2G1B/ACTG2/MYH11/AGTR1/MYLK/KCNMA1/KCNMB1/MYL9/ADCY5/PPP1R12B/ACTA2/PPP1R14A/CALD1/PLA2G5/ADCY2/IRAG1/CACNA1C/ADRA1D/PLA2G2C/ADCY1/GUCY1A1/CALML3/PRKG1/ADRA1B/PLA2G12B/GUCY1B1/EDNRA/CALML5/NPR1/PRKCB/AVPR1A |

| | | | |
|---|---|---|---|
| KEGG_SPLICEOSOME | KEGG_SPLICEOSOME | 4.39E-07 | PRPF18/SNW1/MAGOHB/RBM17/SNRPD3/ISY1/LSM5/DHX8/CDC5L/DDX46/LSM6/USP39/RBM22/BCAS2/SNRPG/HNRNPA1L2/DDX42/PRPF38B/CRNKL1/SRSF2/SRSF10/HNRNPA3/SRSF4/SNRPC/PRPF6/SMNDC1/MAGOH/TRA2B/SF3B1/TRA2A/ACIN1/CCDC12/SRSF7/PRPF38A/DHX16/SF3A1/SRSF8/PRPF31/PPIL1/LSM3/RBMX/SNRPF/TCERG1/WBP11/LSM2/SNRPA1/PPIH/HSPA8/SRSF3/PHF5A/CDC40/SART1/SF3A3/SNRNP70/LSM7/DHX38/SNRPB/XAB2/EFTUD2/PRPF19/NCBP1/PRPF8/SNRNP40/PRPF40A/AQR/EIF4A3/HNRNPK/SNRNP200/SRSF1/SF3B2/HNRNPU/HNRNPC/PRPF4/DDX23/SNRPD1/TXNL4A/SF3A2/PUF60/HNRNPM/SF3B5/SF3B3/U2AF2/SRSF9/SNRPA/DHX15/PCBP1/HNRNPA1/LSM4/THOC3/CHERP/ALYREF/PLRG1 |
| KEGG_HYPERTROPHIC_CARDIOMYOPATHY_HCM | KEGG_HYPERTROPHIC_CARDIOMYOPATHY_HCM | 7.02E-07 | DES/SGCA/TPM2/IGF1/DMD/MYL3/ITGA7/CACNA1C/SGCD/PRKAA2/SGCG/CACNG4/LAMA2/CACNB2/TPM1/ACTC1/ITGA8/ITGA9/TGFB3/TGFB2/ITGA1/SLC8A1/ITGA5/CACNA2D3/CACNA2D1/IL6 |
| KEGG_RIBOSOME | KEGG_RIBOSOME | 9.63E-07 | RPS26/RPS25/RPL27A/RPLP1/RPL13/RPS15A/RPL11/RPS13/RPL34/RPL38/RPS24/RPS21/RPL24/RPLP2/RPS11/UBA52/RPS8/RPS16/RPL22/RPS4X/RPS12/RPL23A/RPL15/RPL10A/RPS10/FAU/RPL32/RPS27L/RPL26/RPL6/RPS9/RPL37A/RPL13A/RPL14/RPS18/RPS3/RPS5/RPL4/RPS23/RPL30/MRPL13/RPS3A/RPL12/RPL26L1/RPL18/RSL24D1/RPS7/RPL7A/RPS28/RPL9/RPL35/RPS19/RPL7/RPL3/RPS6/RPLP0/RPS17/RPL29/RPL19/RPL36/RPS15/RPL41/RPSA/RPS2/RPL18A/RPL8/RPL17/RPL22L1 |
| KEGG_PYRIMIDINE_METABOLISM | KEGG_PYRIMIDINE_METABOLISM | 9.39E-07 | POLR3D/NME1/NME3/NT5M/DHODH/POLR2C/DPYD/CMPK2/UMPS/ENTPD3/ITPA/POLR2H/POLR1C/POLR2L/POLR1E/NME6/POLR2D/POLA1/PRIM2/TXNRD1/POLR3H/POLE2/POLR2E/ENTPD4/POLR1A/POLR3B/POLR3A/POLR2B/UCK2/TK2/DCTD/NT5C/POLE3/POLR1B/NUDT2/PNP/NT5C3A/POLR2A/RRM2B/POLR3K/CAD/CTPS1/POLA2/DTYMK/CDA/DCK/POLD3/DUT/PRIM1/CMPK1/POLE/ENTPD6/POLD2/CANT1/RRM1/TYMP/POLD1/POLR3G/TYMS/TK1/RRM2/ENTPD8 |

| | | | |
|---|---|---|---|
| KEGG_CELL_CYCLE | KEGG_CELL_CYCLE | 1.64E-06 | ANAPC2/E2F5/YWHAZ/CDKN2C/YWHAE/E2F4/SKP2/RAD21/DBF4/TTK/PRKDC/SMC1A/FZR1/BUB1/BUB3/ORC6/HDAC1/CDC14B/MCM7/TFDP1/CDC25C/MCM6/PTTG1/CDK1/CDK6/CCNB2/CHEK1/PCNA/BUB1B/E2F1/CDC20/TP53/MCM3/CDC45/CCNB1/ESPL1/MAD2L1/SFN/MCM5/ORC5/CDC7/MDM2/PLK1/CCNA2/CCND1/MCM4/CDC25A/CCNE2/PKMYT1/MCM2/ORC1/E2F2 |
| KEGG_DNA_REPLICATION | KEGG_DNA_REPLICATION | 8.61E-06 | RFC3/RFC1/POLA1/PRIM2/RPA1/POLE2/RFC4/POLE3/RFC5/POLA2/RFC2/POLD3/PRIM1/MCM7/POLE/MCM6/POLD2/PCNA/MCM3/LIG1/FEN1/DNA2/MCM5/POLD1/MCM4/MCM2 |
| KEGG_AMINOACYL_TRNA_BIOSYNTHESIS | KEGG_AMINOACYL_TRNA_BIOSYNTHESIS | 1.18E-05 | AARS1/MTFMT/WARS2/PARS2/TARS1/CARS2/GARS1/CARS1/HARS1/SARS2/FARSB/RARS1/NARS1/MARS1/HARS2/EPRS1/KARS1/EARS2/QARS1/IARS1/SARS1/TARS2/IARS2/VARS1/SEPSECS/YARS1/NARS2/LARS2/MARS2/FARSA/WARS1 |
| KEGG_ECM_RECEPTOR_INTERACTION | KEGG_ECM_RECEPTOR_INTERACTION | 2.04E-05 | RELN/COL2A1/VTN/THBS4/COL4A6/TNXB/ITGA7/COL11A2/TNC/TNN/SV2B/LAMA2/CD36/ITGA8/ITGA9/SV2A/THBS1/LAMA1/ITGA1/ITGA5/COMP/COL3A1/FN1/LAMA4/SDC2/COL6A2/COL6A3/COL1A2/COL4A4/LAMC1/THBS2/ITGB3/ITGA11/COL1A1/COL5A1 |
| KEGG_ALLOGRAFT_REJECTION | KEGG_ALLOGRAFT_REJECTION | 2.70E-05 | HLA-DPA1/FAS/HLA-DMB/HLA-E/HLA-DOB/HLA-DRA/HLA-B/HLA-A/HLA-DQA2/HLA-DQA1/HLA-DRB5/HLA-F/HLA-C/HLA-DRB1/HLA-DOA/HLA-DQB1/HLA-DMA/PRF1/FASLG/IFNG/GZMB/IL10 |
| KEGG_FOCAL_ADHESION | KEGG_FOCAL_ADHESION | 3.32E-05 | RELN/COL2A1/ACTN2/FLNC/VTN/MYLK/THBS4/MYL9/IGF1/COL4A6/TNXB/FLNA/PAK3/ITGA7/COL11A2/TNC/MAPK10/TNN/LAMA2/ITGA8/ITGA9/AKT3/THBS1/LAMA1/CAV1/HGF/ITGA1/ITGA5/SHC4/COMP/PRKCB/COL3A1/PDGFRA/FN1/LAMA4/PARVA/PDGFD/COL6A2/ILK/COL6A3/ACTN1/COL1A2/COL4A4/LAMC1/PDGFC/THBS2/ITGB3/VEGFC/ITGA11/COL1A1/PDGFRB/COL5A1 |

| | | | |
|---|---|---|---|
| KEGG_ANTIGEN_PROCESSING_AND_PRESENTATION | KEGG_ANTIGEN_PROCESSING_AND_PRESENTATION | 3.87E-05 | HLA-DPB1/PDIA3/HSPA8/HSP90AA1/KLRC1/KLRD1/HSPA4/CTSS/TAPBP/CALR/HSPA5/HLA-DPA1/CANX/HLA-DMB/HLA-E/HLA-DOB/HLA-DRA/RFXANK/HLA-B/KLRC2/HLA-A/HLA-DQA2/HLA-DQA1/CD8A/RFX5/PSME1/HLA-DRB5/CD74/HLA-F/HLA-C/TAP2/HLA-DRB1/HLA-DOA/PSME2/HLA-DQB1/KIR2DL4/HLA-DMA/CIITA/TAP1 |
| KEGG_RNA_DEGRADATION | KEGG_RNA_DEGRADATION | 5.28E-05 | DCP2/SKIV2L/LSM5/XRN2/PNPT1/LSM6/PAPOLA/EDC3/LSM1/ZCCHC7/CNOT8/EXOSC8/CNOT3/ENO2/LSM3/PARN/CNOT6L/LSM2/DIS3/TENT4A/MTREX/XRN1/LSM7/CNOT10/MPHOSPH6/CNOT7/EXOSC10/EXOSC2/EXOSC7/EXOSC5/CNOT6/PATL1/EXOSC9/CNOT9/EXOSC4/HSPA9/EDC4/CNOT1/DCPS/HSPD1/LSM4/ENO1 |
| KEGG_ARRHYTHMOGENIC_RIGHT_VENTRICULAR_CARDIOMYOPATHY_ARVC | KEGG_ARRHYTHMOGENIC_RIGHT_VENTRICULAR_CARDIOMYOPATHY_ARVC | 7.81E-05 | DES/ACTN2/CTNNA3/SGCA/DMD/CDH2/ITGA7/CACNA1C/SGCD/SGCG/CACNG4/LAMA2/CACNB2/ITGA8/ITGA9/TCF7L1/ITGA1/SLC8A1/ITGA5/CACNA2D3/CACNA2D1/GJA1/RYR2/ACTN1/ITGB3/ITGA11/CACNG6/LEF1 |
| KEGG_NUCLEOTIDE_EXCISION_REPAIR | KEGG_NUCLEOTIDE_EXCISION_REPAIR | 0.000117 | ERCC2/RPA3/GTF2H2/RPA2/DDB1/CDK7/RAD23B/RFC3/RFC1/XPC/CUL4A/RPA1/POLE2/RFC4/POLE3/ERCC5/GTF2H3/RFC5/RAD23A/RFC2/POLD3/POLE/POLD2/DDB2/PCNA/LIG1/POLD1 |
| KEGG_PROTEASOME | KEGG_PROTEASOME | 0.0001296 | PSMD1/PSMC6/PSMD4/PSMA1/PSMA7/PSME3/PSMB5/PSMD6/PSMB4/PSMC5/PSMF1/PSMB1/PSMA3/PSMD13/PSMB6/PSMD7/PSMC3/PSMC2/PSMC4/PSMD2/PSME4/PSMB3/PSMD8/PSMC1/PSMB7/PSMA6/PSMD12/PSMB2/PSMD14/PSMA4/PSMA5/PSME1/PSMB9/PSME2/PSMB8/PSMD3/IFNG/PSMB10 |
| KEGG_PPAR_SIGNALING_PATHWAY | KEGG_PPAR_SIGNALING_PATHWAY | 0.0002388 | APOA2/APOA1/APOC3/ADIPOQ/FABP4/PLIN1/SORBS1/CD36/RXRG/SLC27A6/FABP3/ACOX2/CPT1C/ILK/LPL/CYP27A1 |
| KEGG_COMPLEMENT_AND_COAGULATION_CASCADES | KEGG_COMPLEMENT_AND_COAGULATION_CASCADES | 0.0003979 | C7/FGA/SERPIND1/F2/FGG/F7/MASP1/F13A1/SERPINF2/C4A/C4B/BDKRB1/SERPINA5/SERPINE1/PROS1/F8/A2M/C1S/C1R/THBD/CFH/FGB/SERPING1/TFPI/F10/CR1/C3/MASP2/PLAT/CFD/F2R/C5/C3AR1/PROC/C5AR1 |

| | | | |
|---|---|---|---|
| KEGG_BASE_EXCISION_REPAIR | KEGG_BASE_EXCISION_REPAIR | 0.0004395 | PARP4/POLL/XRCC1/POLE2/POLB/APEX2/POLE3/PARP1/MPG/NTHL1/UNG/POLD3/NEIL3/POLE/POLD2/PCNA/PARP3/LIG1/FEN1/POLD1 |
| KEGG_GRAFT_VERSUS_HOST_DISEASE | KEGG_GRAFT_VERSUS_HOST_DISEASE | 0.0005474 | HLA-DPA1/IL1B/FAS/HLA-DMB/HLA-E/HLA-DOB/HLA-DRA/HLA-B/HLA-A/HLA-DQA2/HLA-DQA1/HLA-DRB5/HLA-F/HLA-C/HLA-DRB1/HLA-DOA/HLA-DQB1/HLA-DMA/PRF1/FASLG/IFNG/GZMB |
| KEGG_HUNTINGTONS_DISEASE | KEGG_HUNTINGTONS_DISEASE | 0.0007166 | NDUFB5/NDUFC1/NDUFA1/REST/DCTN1/COX7B/CLTC/AP2A1/NDUFB4/UQCRB/CASP9/COX6B1/NDUFS8/UQCR11/NDUFB2/COX7C/NDUFA8/POLR2C/AP2A2/COX7A2L/ATP5F1C/NDUFA6/NDUFB10/ATP5PD/NDUFS5/NDUFB3/DNAL4/NDUFA5/POLR2H/UQCRQ/NDUFA9/NDUFB6/POLR2L/NDUFS6/UQCRFS1/NDUFA10/NDUFS2/CREB3/HDAC2/CLTA/NDUFA2/POLR2D/NDUFS1/COX7A2/NDUFS3/TFAM/SDHD/CASP8/HAP1/TAF4B/NDUFB7/COX5B/ATP5PO/NDUFAB1/UQCR10/DNAH1/POLR2E/BAX/COX4I1/SLC25A5/ATP5MC1P5/SOD1/POLR2B/ATP5MC1/ATP5PB/SP1/COX6A1/PPID/CYCS/AP2B1/SDHB/APAF1/POLR2A/COX5A/VDAC1/UQCRC2/ATP5MC3/CLTB/ATP5F1A/UQCRH/CASP3/COX8A/SDHA/HDAC1/ATP5F1B/HTT/VDAC2P5/VDAC3/NDUFV1/RCOR1/CREB3L1/NDUFS7/VDAC2/PPARG/ATP5F1D/UQCRC1/UQCRHL/TP53/CYC1/PLCB3/BBC3/GRIN1/COX6B2 |
| KEGG_HOMOLOGOUS_RECOMBINATION | KEGG_HOMOLOGOUS_RECOMBINATION | 0.0008164 | RAD50/RAD51D/SSBP1/RPA3/RPA2/XRCC2/TOP3A/RPA1/RAD54B/EME1/MUS81/BRCA2/XRCC3/NBN/POLD3/POLD2/BLM/RAD51/RAD54L/POLD1 |
| KEGG_AUTOIMMUNE_THYROID_DISEASE | KEGG_AUTOIMMUNE_THYROID_DISEASE | 0.0010827 | HLA-DPA1/FAS/HLA-DMB/HLA-E/HLA-DOB/HLA-DRA/HLA-B/HLA-A/HLA-DQA2/HLA-DQA1/HLA-DRB5/HLA-F/HLA-C/HLA-DRB1/HLA-DOA/HLA-DQB1/HLA-DMA/CTLA4/PRF1/FASLG/GZMB/IL10 |
| KEGG_PEROXISOME | KEGG_PEROXISOME | 0.0010443 | ABCD4/EHHADH/NUDT19/PEX13/PEX11G/PEX5/ABCD1/SLC25A17/SCP2/ACOX1/PRDX1/PEX11B/PEX2/HSD17B4/HACL1/FAR1/HMGCL/PEX1/SOD1/PRDX5/ACOX3/ABCD3/BAAT/PEX14/PEX7/ACAA1/XDH/GSTK1/NUDT12/PEX10/MVK/DHRS4/EPHX2/IDH2/IDH1/ACSL5/PXMP2/SLC27A2/NOS2 |

| | | | |
|---|---|---|---|
| KEGG_MISMATCH_REPAIR | KEGG_MISMATCH_REPAIR | 0.0011633 | SSBP1/MSH3/RPA3/RPA2/PMS2/RFC3/RFC1/MSH6/RPA1/RFC4/RFC5/RFC2/POLD3/MSH2/POLD2/PCNA/EXO1/LIG1/POLD1 |
| KEGG_FRUCTOSE_AND_MANNOSE_METABOLISM | KEGG_FRUCTOSE_AND_MANNOSE_METABOLISM | 0.0012579 | HK3/FCSK/GMPPA/PMM2/PFKL/TPI1/FBP1/GMPPB/SORD/KHK/PFKP/PFKFB2/GFUS/HK2/ALDOA/GMDS/AKR1B10 |
| KEGG_UBIQUITIN_MEDIATED_PROTEOLYSIS | KEGG_UBIQUITIN_MEDIATED_PROTEOLYSIS | 0.0019679 | CUL1/FBXW8/KLHL9/CBLB/CDC16/ERCC8/BTRC/RHOBTB2/TRIP12/WWP1/COP1/HERC4/UBE2W/UBE2A/UBE2E3/CBL/XIAP/ANAPC11/ANAPC7/BIRC2/UBE2G1/UBE3A/UBE2L3/CUL5/UBE4B/UBE2Q1/CUL2/UBE3B/ELOB/CUL7/SAE1/CDC27/UBE2D2/UBE2E1/CDC26/ELOC/UBR5/TRIM37/UBE2I/HUWE1/CUL3/ANAPC4/RCHY1/UBE2N/ANAPC1/UBE2D3/MAP3K1/UBE2K/HERC2/UBE2R2/UBE4A/SMURF1/PPIL2/NEDD4L/DDB1/FBXO4/UBE2J2/CDC23/VHL/UBE2J1/PIAS4/BIRC6/UBE2Z/ANAPC5/STUB1/MGRN1/CUL4A/PRPF19/CDC34/BRCA1/SYVN1/UBE2M/PML/ANAPC2/UBA1/FANCL/UBE3C/SKP2/WWP2/UBE2O/UBA7/FZR1/UBA6/SOCS1/UBE2S/KEAP1/UBE2L6/DDB2/CDC20/BIRC3/CBLC/MDM2 |
| KEGG_AMINO_SUGAR_AND_NUCLEOTIDE_SUGAR_METABOLISM | KEGG_AMINO_SUGAR_AND_NUCLEOTIDE_SUGAR_METABOLISM | 0.0020673 | GALK2/CMAS/PGM3/HK3/FCSK/GMPPA/PMM2/PGM2/CHIT1/GFPT1/GMPPB/GPI/GALE/GNPNAT1/NANS/GFUS/HK2/GMDS/CHIA |
| KEGG_ADIPOCYTOKINE_SIGNALING_PATHWAY | KEGG_ADIPOCYTOKINE_SIGNALING | 0.0024041 | IRS4/ADIPOQ/SLC2A4/NPY/PRKAA2/MAPK10/LEPR/CD36/RXRG/AKT3 |
| KEGG_HEDGEHOG_SIGNALING_PATHWAY | KEGG_HEDGEHOG_SIGNALING_PATHWAY | 0.0025291 | PTCH2/GAS1/GLI3/WNT9A/RAB23/GLI1/HHIP/SHH/SMO/DHH/WNT2B/WNT10B/PTCH1/PRKACB/GLI2/BMP5/BMP6/WNT6/WNT16/WNT5B |
| KEGG_CITRATE_CYCLE_TCA_CYCLE | KEGG_CITRATE_CYCLE_TCA_CYCLE | 0.0029567 | PC/OGDH/IDH3G/SUCLA2/DLD/SUCLG2/ACO1/MDH1/SDHD/DLST/IDH3B/SUCLG2P2/PDHA1/PCK2/SDHB/CS/MDH2/ACO2/SDHA/IDH3A/FH/IDH2/IDH1/ACLY/DLAT/PCK1 |
| KEGG_PRIMARY_IMMUNODEFICIENCY | KEGG_PRIMARY_IMMUNODEFICIENC | 0.003374 | BLNK/RFXANK/UNG/CD8A/RFX5/TAP2/ADA/LCK/CIITA/TAP1/IGLL1 |
| KEGG_GAP_JUNCTION | KEGG_GAP_JUNCTION | 0.0036397 | TUBA3C/ADCY5/HTR2A/TUBB4A/HTR2B/ADCY2/ADCY1/GUCY1A1/PRKG1/TUBA1A/GUCY1B1/LPAR1/TUBB6/PRKCB/PDGFRA/GJA1/PDGFD/GUCY1A2/ITPR1/GNAI1/PDGFC/PDGFRB/PRKACB |

| | | | |
|---|---|---|---|
| KEGG_MAPK_SIGNALING_PATHWAY | KEGG_MAPK_SIGNALING_PATHWAY | 0.0044616 | PLA2G1B/FLNC/FGF10/FGF7/PLA2G5/RPS6KA6/FLNA/NGF/CACNA1C/FGF2/MAPK10/PLA2G2C/CACNG4/CACNA1H/PTPN5/HSPA2/CACNB2/TGFB3/MAPT/NFATC4/AKT3/PLA2G12B/IL1R1/FGF13/RASGRP2/FGFR1/MAP3K20/TGFB2/CACNA2D3/CACNA2D1/PRKCB/CACNA1A/PDGFRA/NTF3/FGF1/DUSP9/DUSP1/HSPB1/MRAS/MEF2C/HSPA1A/GADD45G/FGF12/RASGRF2/RASGRP4/GADD45B/CACNG6/PDGFRB/PRKACB |
| KEGG_CARDIAC_MUSCLE_CONTRACTION | KEGG_CARDIAC_MUSCLE_CONTRACTION | 0.0048546 | COX8C/ATP1A2/TPM2/MYL3/ATP1B2/CACNA1C/CACNG4/CACNB2/TPM1/ACTC1/COX7A1/COX7B2/SLC8A1/CACNA2D3/CACNA2D1 |
| KEGG_GLYCEROLIPID_METABOLISM | KEGG_GLYCEROLIPID_METABOLISM | 0.0061065 | PNLIP/PNLIPRP1/CEL/PNLIPRP2 |
| KEGG_ALANINE_ASPARTATE_AND_GLUTAMATE_METABOLISM | KEGG_ALANINE_ASPARTATE_AND_GLUTAMATE_METAB | 0.0062684 | ADSS1/ASS1/IL4I1/CAD/GOT2/GFPT1/PPAT/ALDH4A1/GOT1/ACY3/GPT2/GAD1/GPT/CPS1 |
| KEGG_BUTANOATE_METABOLISM | KEGG_BUTANOATE_METABOLISM | 0.0065208 | ALDH7A1/HMGCS1/EHHADH/ALDH2/ALDH3A2/HADHA/ALDH9A1/AACS/HMGCL/PDHA1/ACSM1/HADH/L2HGDH/BDH1/ACAT2/ECHS1/ACSM3/ACADS/AKR1B10/GAD1 |
| KEGG_TYPE_I_DIABETES_MELLITUS | KEGG_TYPE_I_DIABETES_MELLITUS | 0.0097826 | HLA-DPA1/IL1B/FAS/HLA-DMB/HLA-E/HLA-DOB/HLA-DRA/HLA-B/HLA-A/HLA-DQA2/HLA-DQA1/HSPD1/HLA-DRB5/HLA-F/HLA-C/HLA-DRB1/HLA-DOA/HLA-DQB1/HLA-DMA/PRF1/FASLG/IFNG/GZMB/GAD1 |
| KEGG_NATURAL_KILLER_CELL_MEDIATED_CYTOTOXICITY | KEGG_NATURAL_KILLER_CELL_MEDIATED_CYTOTOXICITY | 0.0098157 | KLRC1/KLRD1/PIK3R3/GRB2/PTPN6/PTK2B/CD244/NRAS/KRAS/PIK3CB/CHP2/MAP2K1/ULBP2/PLCG2/MAPK3/RAET1L/FAS/SYK/HLA-E/MAP2K2/SH3BP2/HLA-B/KLRC2/HLA-A/TNFSF10/VAV2/HRAS/CASP3/SH2D1B/TNFRSF10D/MICB/HLA-C/CHP1/ICAM1/TNFRSF10B/ULBP3/KIR2DL4/PRKCG/TNFRSF10A/LCK/PRF1/FASLG/IFNG/GZMB |
| KEGG_APOPTOSIS | KEGG_APOPTOSIS | 0.0100704 | IKBKB/BID/BAD/BCL2L1/PIK3R3/NFKB1/TRADD/CASP8/PIK3CB/CHP2/BAX/IL1B/CAPN2/TRAF2/MYD88/AKT1/CASP6/FAS/CYCS/APAF1/AIFM1/CHUK/TNFSF10/CASP3/IRAK1/PRKX/PRKAR1B/TNFRSF10D/CAPN1/CHP1/CASP10/FADD/TNFRSF10B/TP53/BIRC3/DFFB/ENDOG/TNFRSF10A/CASP7/FASLG |

| | | | |
|---|---|---|---|
| KEGG_TYPE_II_DIABETES_MELLITUS | KEGG_TYPE_II_DIABETES_MELLIT | 0.0109315 | IRS4/ADIPOQ/SLC2A4/SLC2A2/CACNA1C/MAPK10/CACNA1A |
| KEGG_ONE_CARBON_POOL_BY_FOLATE | KEGG_ONE_CARBON_POOL_BY_FOLATE | 0.0118434 | MTHFD2L/ALDH1L1/GART/ATIC/MTHFR/SHMT1/MTHFD2/MTHFD1L/DHFR/MTHFD1/SHMT2/TYMS |
| KEGG_BASAL_CELL_CARCINOMA | KEGG_BASAL_CELL_CARCINOMA | 0.0119509 | PTCH2/GLI3/WNT9A/GLI1/HHIP/TCF7L1/FZD10/SHH/SMO/FZD7/WNT2B/WNT10B/PTCH1/LEF1/GLI2/FZD4/FZD1/FZD8/WNT6/WNT16/WNT5B |
| KEGG_CELL_ADHESION_MOLECULES_CAMS | KEGG_CELL_ADHESION_MOLECULES_CAMS | 0.0144702 | MPZ/NRXN3/NRXN2/NEGR1/CADM3/NFASC/CDH2/CNTN1/CLDN6/JAM2/CLDN11/JAM3/NLGN4X/NLGN1/NCAM2/CNTNAP1/ITGA8/ITGA9/NCAM1/SELP/L1CAM/SDC2/NLGN3/CLDN9/CLDN19/SELL/VCAN/CLDN5/NRCAM/NLGN2/PTPRM/SELE |
| KEGG_PENTOSE_PHOSPHATE_PATHWAY | KEGG_PENTOSE_PHOSPHATE_PATHW | 0.0169313 | RPE/PGLS/TALDO1/PGM2/PFKL/FBP1/TKT/PRPS2/PGD/GPI/PFKP/ALDOA |
| KEGG_RNA_POLYMERASE | KEGG_RNA_POLYMERASE | 0.0201228 | POLR3D/POLR2C/POLR2H/POLR1C/POLR2L/POLR1E/POLR2D/POLR3H/POLR2E/POLR1A/POLR3B/POLR3A/POLR2B/POLR1B/POLR2A/POLR3K/POLR3G |
| KEGG_CYTOSOLIC_DNA_SENSING_PATHWAY | KEGG_CYTOSOLIC_DNA_SENSING_PATHWAY | 0.0288762 | POLR3D/NFKBIB/RIPK1/RELA/IRF7/ADAR/IKBKB/POLR1C/NFKB1/POLR3H/POLR3B/IL1B/IRF3/POLR3A/PYCARD/CHUK/POLR3K/IKBKE/CCL5/IL18/CASP1/POLR3G/CXCL10/AIM2 |
| KEGG_INTESTINAL_IMMUNE_NETWORK_FOR_IGA_PRODUCTION | KEGG_INTESTINAL_IMMUNE_NETWORK_FOR_IGA_PRODUCTION | 0.0318707 | HLA-DPA1/IL15RA/HLA-DMB/HLA-DOB/HLA-DRA/HLA-DQA2/HLA-DQA1/ITGB7/HLA-DRB5/HLA-DRB1/HLA-DOA/HLA-DQB1/HLA-DMA/PIGR/CCL28/TNFSF13/IL10 |
| KEGG_LONG_TERM_DEPRESSION | KEGG_LONG_TERM_DEPRESSION | 0.0360465 | PLA2G1B/GNAO1/IGF1/PLA2G5/PLA2G2C/GUCY1A1/PRKG1/PLA2G12B/GUCY1B1/PRKCB/CACNA1A/GUCY1A2/ITPR1/GNAI1/GNAZ |

| | | | |
|---|---|---|---|
| KEGG_OXIDATIVE_PHOSPHORYLATION | KEGG_OXIDATIVE_PHOSPHORYLATION | 0.037261 | NDUFB5/NDUFC1/NDUFA1/COX7B/NDUFB4/UQCRB/ATP5MG/COX6B1/NDUFS8/UQCR11/NDUFB2/ATP5MF/COX7C/NDUFA8/ATP6V1A/COX7A2L/ATP5F1C/NDUFA6/NDUFB10/ATP5PD/ATP6V0A2/NDUFS5/NDUFB3/NDUFA5/ATP6V1B2/UQCRQ/NDUFA9/NDUFB6/NDUFA11/NDUFS6/UQCRFS1/NDUFA10/NDUFS2/NDUFA2/NDUFS1/COX7A2/NDUFS3/SDHD/NDUFB7/COX5B/ATP5PO/NDUFAB1/UQCR10/COX4I1/ATP6V0B/ATP5MC1P5/ATP5MC1/ATP5PB/COX6A1/SDHB/COX15/COX5A/COX10/UQCRC2/ATP5MC3/ATP5F1A/UQCRH/COX8A/PPA2/PPA1/SDHA/ATP5F1B/ATP5ME/NDUFV1/TCIRG1/NDUFS7/ATP5F1D/UQCRC1/UQCRHL/CYC1/ATP4B/ATP12A/COX6B2/ATP4A |
| KEGG_TGF_BETA_SIGNALING_PATHWAY | KEGG_TGF_BETA_SIGNALING_PATHWAY | 0.0399088 | THBS4/AMHR2/GDF6/NOG/DCN/CHRD/TGFB3/LEFTY2/THBS1/LTBP1/NODAL/TGFB2/SMAD9/COMP/GDF7/ID4/BMPR1B/INHBA/THBS2/ACVR1/BMP5/BMP6/PITX2 |
| KEGG_TYROSINE_METABOLISM | KEGG_TYROSINE_METABOLISM | 0.0425791 | AOX1/ADH1B/AOC3/HPD/ADH1A/ADH7/TPO/ALDH1A3/ADH4/TYRP1 |
| KEGG_P53_SIGNALING_PATHWAY | KEGG_P53_SIGNALING_PATHWAY | 0.0420615 | SESN2/PERP/FAS/PPM1D/EI24/CYCS/APAF1/CCNG1/RRM2B/PIDD1/CASP3/CD82/GTSE1/CDK1/CDK6/CCNB2/CHEK1/DDB2/PMAIP1/TNFRSF10B/TP53/CCNB1/SFN/MDM2/STEAP3/CCND1/BBC3/CCNE2/RRM2/TP73/SERPINB5 |
| KEGG_GNRH_SIGNALING_PATHWAY | KEGG_GNRH_SIGNALING_PATHWAY | 0.0453001 | PLA2G1B/ADCY5/PLA2G5/ADCY2/CACNA1C/MAPK10/PLA2G2C/ADCY1/CALML3/PLA2G12B/CALML5/MMP2/PRKCB/CGA |
| KEGG_TIGHT_JUNCTION | KEGG_TIGHT_JUNCTION | 0.0476718 | MYH11/ACTN2/MYL9/CTNNA3/CLDN6/MPDZ/JAM2/CLDN11/JAM3/AKT3/MAP3K20/AMOTL1/PRKCB/MYH10/CLDN9/EPB41L3/RAB3B/CLDN19/MRAS/CLDN5/GNAI1/ACTN1 |
| KEGG_REGULATION_OF_ACTIN_CYTOSKELETON | KEGG_REGULATION_OF_ACTIN_CYTOSKELETON | 0.048218 | CHRM2/ACTN2/MYLK/FGF10/MYL9/F2/FGF7/CFL2/PAK3/ITGA7/FGF2/ITGA8/ITGA9/CHRM4/FGF13/FGFR1/ITGA1/ITGA5/BDKRB1/MYH10/PDGFRA/FN1/PDGFD/FGF1/FGD1/MRAS/ACTN1/WASF1/PDGFC/FGF12/ITGB3/ENAH/ITGA11/PDGFRB/GSN/RDX/PFN2/RRAS |
