# Supplementary material for: Prognostic value of disulfidptosis-associated genes in gastric cancer: a comprehensive analysis
Source: Front Oncol. 2025 Mar 4;15:1512394. doi: 10.3389/fonc.2025.1512394 (PMC11913695; doi:10.3389/fonc.2025.1512394)
Supplement: Supplementary file 4 [file DataSheet4.pdf]

ID	Description	pvalue	core_enrichment
GOBP_DNA_TEMPLATED_DNA_REPLICATION	GOBP_DNA_TEMPLATED_DNA_REPLICATION	1.00E-10	ZPR1/GMNN/POLA1/ATAD5/FAM111A/PRIM2/RPA1/CDC34/BRCA1/MCMBP/POLE2/POLB/INO80/TOPBP1/NOC3L/FANCM/DDX21/RFC4/LRWD1/DDX23/POLE3/BARD1/UPF1/EME1/MUS81/PARP1/MCM9/DSCC1/DONSON/WRNIP1/WRN/BRCA2/POLG/HELB/DBF4/RRM2B/RFC5/GINS1/TRAIP/DDX11/FBXO5/SIRT7/POLA2/MMS22L/DNAJA3/MCM10/CARM1/ZGRF1/ORC6/RFWD3/RBBP8/RFC2/NBN/POLD3/WDR18/GINS2/TIPIN/STOML2/PRIM1/MCM7/GEN1/POLE/MCM6/WDHD1/TWNK/EME2/POLD2/ALYREF/CENPX/RRM1/TIMELESS/PCNA/NUGGC/CHTF18/GINS3/HMGA1/BLM/POLQ/RAD51/TICRR/MCM3/LIG1/FEN1/DNA2/CDC45/ENDOG/MCM5/ORC5/CDC7/MCIDAS/RECQL4/TONSL/GINS4/POLD1/MCM4/CCNE2/E2F8/MCM2/E2F7/CDT1/ORC1

GOBP_TELOMERE_ORGANIZATION	GOBP_TELOMERE_ORGANIZATION	1.00E-10	H4C13/CCT6A/SHLD2/SHLD3/XRCC6/MAD2L2/RPA3/H3C12/PNKP/RIF1/RPA2/SMC5/MAP3K4/H3-3B/FBXO4/PPP1R10/ATR/NABP2/PARN/TERF2/H3C10/TFIP11/SMG1/HAT1/ACTL6A/HSP90AA1/H3-3A/RTEL1/MAP2K7/SMG5/XRCC5/NAF1/RFC1/UCHL5/XRN1/ACD/INO80D/H4C9/LTO1/H3C1/RPA1/H4C4/STN1/RUVBL1/XRCC1/INO80E/USP7/INO80/MYC/SRC/H4C3/NAT10/DKC1/HNRNPU/SLF1/HNRNPC/SLX4/MAPK3/PML/RUVBL2/TCP1/EXOSC10/PTGES3/NEK2/HNRNPD/PIF1/CCT8/MCRS1/CCT2/UPF1/HNRNPA2B1/H4C2/PARP1/CCT3/H4C1/WRN/GNL3/BRCA2/NFRKB/PINX1/ZSCAN4/CCT4/PRKDC/CCT7/CCT5/XRCC3/AURKB/H3C2/SIRT6/NBN/H3C7/GAR1/TELO2/DCLRE1B/HNRNPA1/WRAP53/NHP2/PRKCQ/EZH2/TNKS/PCNA[illegible]

GOBP_MITOCHONDRIAL_GENE_EXPRESSION	GOBP_MITOCHONDRIAL_GENE_EXPRESSION	1.00E-10	UQCC2/MRPS23/DAP3/MRPL24/MRPS15/MRPL57/MRPL48/TRMT5/MRPL9/MRPL40/MTRF1L/PRORP/AARS2/NSUN3/MRPL27/PNPT1/RARS2/GATC/MRPS10/TRNT1/MRPL45/DARS2/MRPS7/MRPL36/YARS2/FASTKD2/TRIT1/GFM1/MRPL32/MRPL20/MRPS33/RMND1/MRPL17/MRPS30/MTO1/MRPL14/TFB2M/FASTKD5/THAP11/TRMT61B/MTG2/MRPL30/MRPL3/RCC1L/HSD17B10/MRPL18/RPUSD3/MRPL22/MRPL21/MRPL52/MRPL13/WARS2/MRPS11/MRPL19/MRPL35/MRPS18B/MRPS9/MRPL42/FASTK/TRUB2/GARS1/MRPL43/MRPL34/TFAM/HARS1/MRPL28/MRPL47/FASTKD1/SARS2/MRPS18A/MRPS22/MRPL39/PTCD3/MRPL2/TACO1/MRPL58/MRPL49/MRPS26/MRPS5/CHCHD1/MTG1/MRPL1/MRPL15/MRPL50/PUS1/GATB/MRPL46/GADD45GIP1[illegible]

GOBP_TRNA_METABOLIC_PROCESS	GOBP_TRNA_METABOLIC_PROCESS	1.00E-10	AARS2/NSUN3/CLP1/FAM98A/LSM6/RARS2/ANG/GATC/TRMT112/TRNT1/TARBP1/LAGE3/POP5/TRMT6/ALKBH8/TYW3/DARS2/LARS1/YARS2/TRIT1/PSTK/ZCCHC7/ELP1/FARS2/MTO1/SLFN13/THUMPD3/GTF3C1/THUMPD2/EXOSC8/ELP5/TRMO/ADAT1/GTF3C3/TRMT61B/HSD17B10/AARS1/FTSJ1/TRPT1/GTF3C4/MTFMT/THUMPD1/POLR2L/URM1/WARS2/PARS2/TARS1/CARS2/TRUB2/GARS1/TSEN34/GTF3C2/CARS1/DALRD3/HARS1/TSEN2/POP4/SARS2/TRMT11/QTRT2/METTL2A/FARSB/METTL2B/RTCB/TRMT10A/TRMT2A/GTF3C6/NSUN2/ELP6/LRRC47/NAT10/TRUB1/CTU1/EXOSC10/PUS1/GATB/EXOSC2/EXOSC7/TRMU/RARS1/NARS1/POP7/WDR6/WDR4/GON7/MARS1/HARS2/BRF1/DTWD2/EPRS1/KARS1/DTD2/ELP3/EARS2/PUS[illegible]

GOBP_MITOCHONDRIAL_TRANSLATION	GOBP_MITOCHONDRIAL_TRANSLATION	1.00E-10	UQCC2/MRPS23/DAP3/MRPL24/MRPS15/MRPL57/MRPL48/MRPL9/MRPL40/MTRF1L/AARS2/NSUN3/MRPL27/RARS2/GATC/MRPS10/MRPL45/DARS2/MRPS7/MRPL36/YARS2/FASTKD2/GFM1/MRPL32/MRPL20/MRPS33/RMND1/MRPL17/MRPS30/MRPL14/MTG2/MRPL30/MRPL3/RCC1L/MRPL18/RPUSD3/MRPL22/MRPL21/MRPL52/MRPL13/WARS2/MRPS11/MRPL19/MRPL35/MRPS18B/MRPS9/MRPL42/TRUB2/GARS1/MRPL43/MRPL34/HARS1/MRPL28/MRPL47/SARS2/MRPS18A/MRPS22/MRPL39/PTCD3/MRPL2/TACO1/MRPL58/MRPL49/MRPS26/MRPS5/CHCHD1/MTG1/MRPL1/MRPL15/MRPL50/GATB/MRPL46/GADD45GIP1/MRPL54/MRPL11/EARS2/MRPL16/MPV17L2/GFM2/MRPS18C/LRPPRC/MRPS12/MRPS25/MRPL41/AURKAIP1/RPUSD4/MRPS[illegible]

GOCC_DNA_PACKAGING_COMPLEX	GOCC_DNA_PACKAGING_COMPLEX	1.00E-10	IRF4/H4C13/XRCC6/H3C12/EP400/SMC3/H3-3B/BRD8/DSN1/H2BC7/MRGBP/H1-10/TERF2/H3C10/ZNHIT1/MACROH2A1/ACTL6A/KAT5/H3-3A/XRCC5/H2AW/STAG1/ACD/SPHK2/SGO2/ACTR6/H4C9/H3C1/H2BC11/H4C4/STN1/RUVBL1/H4C3/SLF1/RUVBL2/H2BC9/NUF2/H1-2/H4C2/H2BC18/SRCAP/H2AC18/H4C1/NCAPH2/KDM1B/RAD21/SMC4/NCAPD2/SMC1A/H2BC10/H1-3/NDC80/H3C13/H3C2/H3C7/H2AC20/CENPA/H2BC12/SPC25/SMC2/H2AZ1/H2BC15/H2AC13/NCAPG2/SPC24/H2BC14/H3C6/NCAPH/TERT/NCAPD3/H2AC21/H2AX/H3C8/H2BC13/H2BC17/KNL1/H3C11/NCAPG/ZWINT/H1-4/H3C3/H2AC12/H2AC16/REC8/H1-5

GOBP_CELL_CYCLE_CHECKPOINT_SIGNALING	GOBP_CELL_CYCLE_CHECKPOINT_SIGNALING	1.00E-10	MAD2L1BP/CDK2/UIMC1/RPS27L/RPL26/TP53BP1/TPR/MAD2L2/HINFP/RPA2/CRADD/FBXO4/PPP1R10/ATR/NABP2/RAD9A/SPDL1/CDK5RAP3/NAE1/RAD9B/ANAPC15/NEK11/USP28/XPC/CUL4A/TRIP13/CHEK2/PRPF19/BRCC3/BRCA1/INTS7/NSUN2/TOPBP1/PML/NUF2/BARD1/EME1/MUS81/DONSON/ATRIP/ZFYVE19/ZW10/BRCA2/TRIAP1/CCNG1/TAOK2/RINT1/TTK/PRKDC/KNTC1/PIDD1/XRCC3/FZR1/CASP2/ZWILCH/AURKB/NDC80/BUB1/CCAR2/BUB3/RFWD3/RBBP8/CDC14B/NBN/TELO2/MSH2/TIPIN/GEN1/GTSE1/SPC25/CDK1/PLK2/EME2/TTI2/FBXO6/CENPF/INCENP/TIMELESS/CHEK1/SPC24/BUB1B/E2F1/DOT1L/BLM/CDC20/TP53/RAD51/TICRR/CLSPN/CHMP4C/FANCD2/DNA2/CDC45/CCNB1/HASPIN/MAD2L1/DTL/H[illegible]

GOBP_TELOMERE_MAINTENANCE	GOBP_TELOMERE_MAINTENANCE	1.00E-10	CCT6A/SHLD2/SHLD3/XRCC6/MAD2L2/RPA3/PNKP/RIF1/RPA2/SMC5/MAP3K4/FBXO4/PPP1R10/ATR/NABP2/PARN/TERF2/TFIP11/SMG1/ACTL6A/HSP90AA1/RTEL1/MAP2K7/SMG5/XRCC5/NAF1/RFC1/UCHL5/XRN1/ACD/INO80D/LTO1/RPA1/STN1/RUVBL1/XRCC1/INO80E/USP7/INO80/MYC/SRC/NAT10/DKC1/HNRNPU/SLF1/HNRNPC/SLX4/MAPK3/PML/RUVBL2/TCP1/EXOSC10/PTGES3/NEK2/HNRNPD/PIF1/CCT8/MCRS1/CCT2/UPF1/HNRNPA2B1/PARP1/CCT3/WRN/GNL3/BRCA2/NFRKB/PINX1/ZSCAN4/CCT4/PRKDC/CCT7/CCT5/XRCC3/AURKB/SIRT6/NBN/GAR1/TELO2/DCLRE1B/HNRNPA1/WRAP53/NHP2/PRKCQ/TNKS/PCNA/PARP3/BLM/DCLRE1A/RAD51/EXO1/FEN1/DNA2/TERT/MAPK15/RECQL4/CCNE2

GOCC_CONDENSED_CHROMOSOME_CENTROMERIC_REGION	GOCC_CONDENSED_CHROMOSOME_CENTROMERIC_REGION	1.00E-10	TP53BP1/CBX3/TPR/CHAMP1/SMARCD1/NUDCD2/ORC2/BRD7/H3-3B/SUGT1/CENPS/DSN1/SPDL1/CHMP6/AHCTF1/SMARCC1/XPO1/NUP160/ACTL6A/PPP1CC/SEH1L/KAT5/H3-3A/SKA2/SMARCC2/CHMP2B/SGO2/SMARCB1/CHMP7/ITGB3BP/CHMP1A/NUP85/NUP43/HSF1/NDE1/HNRNPU/CENPW/LRWD1/NUF2/CHMP5/NEK2/CENPI/CKAP5/MTBP/SKA3/ZW10/PINX1/KNSTRN/TTK/HJURP/NCAPD2/NUP37/KNTC1/SMC1A/SGO1/RANGAP1/KMT5C/SEC13/CENPO/ZWILCH/AURKB/NDC80/BUB1/SMARCD2/BUB3/SPAG5/CENPA/SMARCE1/ERCC6L/SPC25/SMARCA4/CENPX/CENPK/CENPF/INCENP/KIF18A/CENPV/CENPU/SPC24/BUB1B/CENPN/KIF22/CHMP4C/CCNB1/KIF2C/SKA1/NCAPD3/MAD2L1/CENPE/PLK1/BIRC5[illegible]

GOCC_RIBOSOMAL_SUBUNIT	GOCC_RIBOSOMAL_SUBUNIT	1.00E-10	RPS25/RPL27A/RPLP1/RPL13/MRPS23/DAP3/RPS15A/RPL11/MRPL24/MRPS15/MRPL57/RPS13/RPL34/RPL38/MRPL48/RPS24/MRPL9/RPS21/MRPL40/RPL24/NSUN3/MRPL27/RPLP2/MRPS10/MRPL45/RPS11/UBA52/RPS8/RPS16/RPL22/RPS4X/RPS12/RPL23A/MRPS7/MRPL36/RPL15/MRPL32/RPL10A/MRPL20/MRPS33/RPS10/FAU/RPL32/MRPL17/RPS27L/MRPS30/RPL26/RPS14/RPL6/RPS9/MRPL14/RPL37A/RPL13A/RPL14/MRPL30/MRPL3/RPS18/MRPL18/RPS3/RPS5/RPL4/MRPL22/MRPL21/RPS23/RPL30/MRPL52/MRPL13/MRPS11/MRPL19/RPS3A/MRPL35/RPL12/RPL26L1/MRPS18B/MRPS9/MRPL42/RPL18/RPS7/MRPL43/MRPL34/NSUN4/FXR2/RPL7A/MRPL28/MRPL47/RPS28/MRPS18A/RPL9/RACK1/MRPS22/MRPL39/R[illegible]

GOBP_SISTER_CHROMATID_SEGREGATION	GOBP_SISTER_CHROMATID_SEGREGATION	1.00E-10	TOP2B/ANAPC1/DIS3L2/NEK6/TPR/MAD2L2/CHAMP1/SMARCD1/CTCF/SMC5/BRD7/SMC3/WAPL/DSN1/SPDL1/CHMP6/EML3/PPP2R1A/CDC23/SMARCC1/MACROH2A1/TUBG1/ACTL6A/RB1/SEH1L/KPNB1/TENT4A/KAT5/ANAPC15/ESCO1/ANAPC5/SMARCC2/STAG1/CHMP2B/NUP62/SGO2/TRIP13/SMARCB1/PDCD6IP/SFPQ/RAB11A/CHMP7/MCMBP/CHMP1A/NAA50/KMT5A/VPS4B/INO80/AKAP8/HNRNPU/SLF1/NAA10/ANAPC2/NUF2/CHMP5/NEK2/RAN/DSCC1/NCAPH2/ZW10/PINX1/KATNB1/RAD21/BCL7A/SMC4/KNSTRN/TTK/NCAPD2/PDS5A/KNTC1/SMC1A/PSRC1/SGO1/PRC1/DDX11/XRCC3/FBXO5/ZWILCH/AURKB/NDC80/BUB1/SMARCD2/BUB3/SPAG5/KIF23/DDX12P/WRAP73/SMARCE1/KIF14/GEN1/PHB2/RRS1/TNKS/SPC25/NUDC/S[illegible]

GOMF_STRUCTURAL_CONSTITUENT_OF_RIBOSOME	GOMF_STRUCTURAL_CONSTITUENT_OF_RIBOSOME	1.00E-10	RPS25/RPL27A/RPLP1/RPL13/MRPS23/DAP3/RPS15A/RPL11/MRPL24/MRPS15/MRPL57/RPS13/RPL34/RPL38/RPS24/MRPL9/RPS21/RPL24/MRPL27/RPLP2/RPS11/UBA52/RPS8/RPS16/RPL22/RPS4X/RPS12/RPL23A/MRPS7/MRPL36/RPL15/MRPL32/RPL10A/MRPL20/MRPS33/RPS10/FAU/RPL32/MRPL17/RPS27L/MRPS30/RPL26/RPS14/RPL6/RPS9/MRPL14/RPL37A/RPL13A/RPL14/SRBD1/MRPL30/MRPL3/RPS18/MRPL18/RPS3/RPS5/RPL4/MRPL22/MRPL21/RPS23/RPL30/MRPL52/MRPL13/MRPS11/MRPL19/RPS3A/MRPL35/RPL12/RPL26L1/MRPS18B/MRPS9/MRPL42/RPL18/RSL24D1/RPS7/MRPL43/MRPL34/RPL7A/MRPL28/MRPL47/RPS28/MRPS18A/RPL9/MRPS22/RPL35/RPS19/RPL7/RPL3/MRPL2/RPS6/MRPL49/RPL7L[illegible]

GOBP_RECOMBINATIONAL_REPAIR	GOBP_RECOMBINATIONAL_REPAIR	1.00E-10	RECQL5/UBE2N/MCM8/SFR1/SHLD2/SHLD3/XRCC6/TP53BP1/WAS/MAD2L2/RPA3/EP400/SETD2/RIF1/RPA2/SMC5/BRD8/NABP2/MRGBP/PIAS4/RNF138/ACTL6A/KAT5/SPIDR/RTEL1/XRCC5/XRCC2/FIGNL1/SMCHD1/HDGFL2/SWSAP1/MMS19/TOP3A/HROB/FAN1/FUS/POLL/SFPQ/RPA1/BRCA1/RUVBL1/XRCC1/PSMD14/INO80/SLF1/FANCM/SLX4/RUVBL2/RAD54B/KDM1A/MUS81/FANCB/PARP1/UBQLN4/ERCC5/PALB2/MCM9/PPP4C/WRN/BRCA2/RAD21/HELB/XRCC3/MMS22L/RFWD3/SIRT6/RBBP8/NBN/RMI1/GINS2/WRAP53/RAD51AP1/MCM7/GEN1/DMC1/MCM6/PARPBP/CGAS/TIMELESS/CHEK1/BLM/POLQ/RAD51/AUNIP/RMI2/MCM3/FEN1/CDC45/H2AX/MCM5/CDC7/RECQL4/TONSL/GINS4/RAD54L/MCM4/MCM2/REC8

GOBP_DOUBLE_STRAND_BREAK_REPAIR	GOBP_DOUBLE_STRAND_BREAK_REPAIR	1.00E-10	RNF168/UIMC1/RNF169/MCM8/SFR1/SHLD2/SHLD3/XRCC6/TP53BP1/WAS/MAD2L2/RPA3/SMARCD1/EP400/PNKP/SETD2/RIF1/RPA2/SMC5/BRD7/BRD8/ATR/NABP2/MRGBP/CYREN/MTA1/ACTR5/TFIP11/DEK/SMARCC1/PIAS4/RNF138/ACTL6A/PARP9/ZBTB7A/NHEJ1/TENT4A/KAT5/NUDT16L1/SPIDR/RTEL1/PPP5C/XRCC5/XRCC2/SMARCC2/FIGNL1/OTUB1/SMARCAD1/SMCHD1/HDGFL2/SWSAP1/MMS19/POLA1/TOP3A/TRIP13/FAN1/SMARCB1/FUS/CHEK2/PRPF19/POLL/SFPQ/RPA1/BRCC3/BRCA1/POLM/RUVBL1/XRCC1/DTX3L/PSMD14/POLB/INO80/HSF1/SLF1/FANCM/SLX4/PML/RUVBL2/PAXX/RAD54B/KDM1A/APTX/EME1/MUS81/FANCB/PARP1/UBQLN4/ERCC5/PALB2/MCM9/TDP1/SPIRE2/ATRIP/PPP4C/WRN/BRCA2/RAD21[illegible]

GOBP_RRNA_METABOLIC_PROCESS	GOBP_RRNA_METABOLIC_PROCESS	1.00E-10	RIOK3/NOP10/KRR1/RPL10A/UTP25/ZCCHC7/ZNHIT3/MAK16/UTP14A/BRIX1/ERCC2/TENT4B/UTP11/RPL26/DDX52/RPS14/SLFN13/BUD23/RPF2/SHQ1/GTF3C1/EXOSC8/NOP56/NSA2/DCAF13/WDR46/KRI1/TFB2M/GTF3C3/HEATR1/NOL10/TRMT61B/TSR3/RPL14/UTP3/RBFA/LYAR/RPUSD3/DDX51/WBP11/MACROH2A1/PELP1/DIS3L/GTF3C4/LAS1L/WDR12/DIS3/FTSJ3/MRPS11/TCOF1/MTREX/YTHDF2/UTP4/WDR75/NAF1/POLR1E/SART1/RSL1D1/XRN1/METTL15/RPS7/GTF3C2/IMP3/NSUN4/RPL7A/WDR43/POP4/SMARCB1/RPS28/PIH1D1/ABT1/WDR36/ZCCHC4/FDXACB1/NOP58/RPL35/RPS19/DEDD2/RPL7/RRP7A/MDN1/BMS1/RPS6/DDX56/UTP20/GTF3C6/EIF4A3/NAT10/MPHOSPH6/NCL/DKC1/RPL7L1/NSUN5/RCL1/MTO[illegible]

GOCC_CHROMOSOME_CENTROMERIC_REGION	GOCC_CHROMOSOME_CENTROMERIC_REGION	1.00E-10	PPP2R5A/CSNK1A1/PPP2CA/DAXX/STAG2/CHRAC1/ZNF618/NUP98/MIS18BP1/ZNF330/SUV39H2/PSEN1/PPP2CB/PHF6/H4C13/TP53BP1/CBX3/TPR/CHAMP1/SMARCD1/NUDCD2/CTCF/SMC5/ORC2/BRD7/SMC3/WAPL/H3-3B/SUGT1/CENPS/CENPL/DSN1/SPDL1/CHMP6/AHCTF1/PPP2R1A/SMARCC1/MACROH2A1/XPO1/NUP160/ACTL6A/PPP1CC/SEH1L/KAT5/H3-3A/SKA2/SMARCC2/STAG1/CHMP2B/SGO2/H4C9/SMARCB1/H2BC11/CHMP7/H4C4/ITGB3BP/CHMP1A/NUP85/NUP43/HSF1/MIS18A/H4C3/NDE1/HNRNPU/CENPW/LRWD1/NUF2/CHMP5/NEK2/H4C2/CENPI/DSCC1/CKAP5/H4C1/MTBP/SKA3/ZW10/KDM4C/PINX1/RAD21/SMC4/KNSTRN/TTK/HJURP/NCAPD2/PDS5A/NUP37/KNTC1/SMC1A/SGO1/SUV39H1/RANG[illegible]

GOBP_REGULATION_OF_CHROMOSOME_ORGANIZATION	GOBP_REGULATION_OF_CHROMOSOME_ORGANIZATION	1.00E-10	CDC26/MAPKAPK5/SMARCA5/CDK5RAP2/TRAPPC12/ACTR8/SMG6/SSBP1/LCMT1/CTNNB1/CUL3/ANAPC4/MAD2L1BP/CDK2/TENT4B/ANAPC1/NEK6/CCT6A/TPR/MAD2L2/SMARCD1/PNKP/GTF2H2/CTCF/SMC5/BRD7/MAP3K4/WAPL/SETDB1/H3-3B/FBXO4/PPP1R10/ATR/NABP2/PARN/SPDL1/TERF2/ACTR5/SMG1/CDC23/SMARCC1/MACROH2A1/ACTL6A/RB1/KAT5/ANAPC15/H3-3A/ANAPC5/RTEL1/MAP2K7/SMG5/XRCC5/SMARCC2/NAF1/UCHL5/XRN1/ACD/INO80D/TRIP13/SMARCB1/SFPQ/STN1/RUVBL1/XRCC1/INO80E/USP7/RNF4/INO80/MYC/SRC/NAT10/DKC1/HNRNPU/SLF1/HNRNPC/SLX4/MAPK3/PML/NAA10/RUVBL2/ANAPC2/TCP1/NUF2/EXOSC10/NEK2/HNRNPD/PIF1/CCT8/MCRS1/CCT2/UPF1/H[illegible]

GOCC_COLLAGEN_CONTAINING_EXTRACELLULAR_MATRIX	GOCC_COLLAGEN_CONTAINING_EXTRACELLULAR_MATRIX	1.00E-10	ELANE/PRTN3/FGL1/AHSG/APOA1/OGN/COL2A1/APOC3/PRG4/SFRP1/APOH/ANGPTL1/ADIPOQ/MYOC/PRSS1/VTN/FGA/ITIH2/THBS4/ANGPTL7/CTSG/FGF10/CMA1/OMD/MGP/F2/PRELP/SPARCL1/CCDC80/ACTA2/COL4A6/COL14A1/FGG/ABI3BP/ADAMTS8/SRPX/CILP/COL21A1/SFRP2/EFEMP1/TNXB/CLC/GPC3/CDH2/SSC5D/FBLN1/SPON1/MFAP4/COL11A2/SMOC2/TNC/EMILIN3/TNN/PODN/HMCN1/GREM1/DPT/VIT/ASPN/DCN/MMP8/COL26A1/CDON/FREM1/LAMA2/CXCL12/TGFB1I1/F7/GDF10/ELN/MFAP5/FBLN5/NTN1/F13A1/COL8A1/SERPINF2/AMBP/AGT/MMRN1/ITIH5/TGFB3/SCARA3/LEFTY2/ADAMTS1/THBS1/LAMA1/HMCN2/COL4A5/SERPINF1/MATN3/LUM/S100A8/CSPG4/CPA3/LTBP1/ECM2/NDP/NCAM1/SERPINA3/C[illegible]

GOBP_DNA_RECOMBINATION	GOBP_DNA_RECOMBINATION	1.00E-10	MSH3/RECQL5/UBE2N/CCNB1IP1/ERCC2/RNF168/TOP2B/MCM8/SFR1/SHLD2/SHLD3/XRCC6/TP53BP1/WAS/MAD2L2/RPA3/EP400/SETD2/RIF1/RPA2/SMC5/CENPS/BATF/BRD8/NABP2/TSN/MRGBP/CYREN/H1-10/TERF2/ACTR5/CNTD1/PIAS4/RNF138/ACTL6A/NHEJ1/KAT5/SPIDR/RTEL1/XRCC5/XRCC2/NONO/FIGNL1/SMARCAD1/SMCHD1/UCHL5/HDAC10/HDGFL2/SWSAP1/INO80D/MMS19/ATAD5/TOP3A/HROB/TRIP13/FAN1/TBX21/FUS/POLL/SFPQ/MSH6/RPA1/HMGB3/BRCA1/PSMC3IP/POLM/RUVBL1/XRCC1/PSMD14/INO80E/POLB/INO80/TOPBP1/APEX2/BCL11B/SLF1/FANCM/SLX4/RUVBL2/PIF1/TFRC/RAD54B/H1-2/KDM1A/MCRS1/BARD1/EME1/MUS81/FANCB/PARP1/UBQLN4/ERCC5/PAXI[illegible]

GOCC_I_BAND	GOCC_I_BAND	1.00E-10	CASQ2/SYNPO2/DES/LDB3/ACTN2/PGM5/FLNC/CASQ1/JPH2/MYL9/ANKRD1/ANK2/PPP1R12B/CRYAB/KCNA5/PDLIM3/SYNC/DMD/NEXN/ASB2/CFL2/MYL3/FBXL22/RYR3/FERMT2/FLNA/CACNA1C/CSRP1/KY/SMTNL2/ITGB1BP2/HRC/ACTC1/FBXO32/ATP2B4/PALLD/PDLIM4/FHL5/SLC8A1/FHOD3/CSRP2/PARVA/SORBS2/SYNPO/PDLIM7/HSPB1/RYR2/ACTN1/DST

GOMF_EXTRACELLULAR_MATRIX_STRUCTURAL_CONSTITUENT	GOMF_EXTRACELLULAR_MATRIX_STRUCTURAL_CONSTITUENT	1.00E-10	OGN/COL2A1/PRG4/ADIPOQ/VTN/FGA/MGP/PRELP/COL4A6/COL14A1/FGG/ABI3BP/SRPX/CILP/COL21A1/EFEMP1/TNXB/FBLN1/SPON1/MFAP4/COL11A2/TNC/EMILIN3/PODN/HMCN1/DPT/TFPI2/ASPN/DCN/LAMA2/ELN/MFAP5/FBLN5/COL8A1/MMRN1/THBS1/LAMA1/GP2/HMCN2/COL4A5/MATN3/LUM/LTBP1/CCN1/FGL2/FBN1/COL24A1/COMP/SBSPON/COL3A1/EMILIN1/FN1/IGFBP7/COL8A2/EFEMP2/LAMA4/FBLN2/COL23A1/COL16A1/COL6A2/COL6A3/TGFBI/SRPX2/VCAN/AEBP1/FMOD/FGB/NID1/PCOLCE/COLQ/COL1A2/COL4A4/SPARC/LAMC1/ANOS1/COL15A1/MFAP2/BGN/COL10A1/LTBP2/THBS2/MMRN2/CTHRC1/COL1A1/OIT3/COL5A1/THSD4/COL7A1/COL9A1/COL12A1/COL5A2/EDIL3/COL4A2/POSTN

GOCC_NUCLEAR_CHROMOSOME	GOCC_NUCLEAR_CHROMOSOME	1.00E-10	RPA3/H3C12/EP400/RPA2/ORC2/SMC3/WAPL/H3-3B/TOP1/BRD8/RAD9A/ACTR5/H3C10/ZNHIT1/MACROH2A1/TUBG1/ACTL6A/RAD9B/KAT5/H3-3A/SPIDR/FIGNL1/HDAC2/SMARCAD1/SMCHD1/UCHL5/ZMIZ2/INO80D/POLA1/ACTR6/H4C9/H3C1/SMARCB1/ING2/PRIM2/RPA1/H4C4/SUDS3/DHX9/BRCA1/PSMC3IP/RUVBL1/POLE2/CHMP1A/INO80E/INO80/TOPBP1/H4C3/HNRNPU/SLX4/RUVBL2/LRWD1/NEK2/POLE3/MCRS1/EME1/H4C2/MUS81/PARP1/SRCAP/ANP32E/RBBP4/H4C1/NCAPH2/BRCA2/NFRKB/PINX1/SINHCAF/RAD21/EXOSC9/SMC4/LRPPRC/INO80C/BRMS1/NCAPD2/GINS1/SMC1A/SUV39H1/SIRT7/POLA2/MMS22L/H3C2/MCM10/CARM1/ORC6/HDAC1/H3C7/POLD3/GINS2/SMARCE1/TIPIN/PRIM1/MCM7/RRS1/DM[illegible]

GOCC_EXTERNAL_ENCAPSULATING_STRUCTURE	GOCC_EXTERNAL_ENCAPSULATING_STRUCTURE	1.00E-10	ELANE/PRTN3/FGL1/AHSG/APOA1/RELN/OGN/COL2A1/APOC3/PRG4/SFRP1/APOH/ANGPTL1/ADIPOQ/KERA/MYOC/PRSS1/VTN/FGA/ITIH2/THBS4/ANGPTL7/CTSG/FGF10/CMA1/OMD/HPSE2/MGP/F2/PRELP/SPARCL1/FLRT2/CCDC80/ACTA2/COL4A6/COL14A1/FGG/ABI3BP/ADAMTS8/SRPX/CILP/COL21A1/SFRP2/ADAMTS18/EFEMP1/TNXB/ADAMTS16/CLC/GPC3/CDH2/SSC5D/FBLN1/ADAMTSL3/BMPER/SPON1/MFAP4/COL11A2/SMOC2/TNC/EMILIN3/TNN/COLEC12/PODN/HMCN1/GREM1/DPT/VIT/TFPI2/ASPN/DCN/MMP8/COL26A1/CDON/FREM1/LRRC17/LAMA2/CXCL12/TGFB1I1/F7/GDF10/ELN/MFAP5/FBLN5/PTX3/NTN1/F13A1/COL8A1/SERPINF2/PRSS2/AMBP/AGT/MMRN1/ITIH5/TGFB3/SCARA3/LEFTY2/ADAMTS1/THBS1/[illegible]

GOMF_HEPARIN_BINDING	GOMF_HEPARIN_BINDING	1.00E-10	ELANE/MPO/AZU1/CEL/SFRP1/APOH/VTN/THBS4/CTSG/FGF10/SERPIND1/F2/PRELP/SLIT2/APOB/CCDC80/RSPO1/FGF7/ADAMTS8/SERPINA10/TNXB/GREM2/SLIT3/RSPO3/PTN/SMOC2/FGF2/CXCL6/PCOLCE2/RSPO2/ADAMTS1/THBS1/ECM2/SELP/FSTL1/FGFR1/CCN1/ADAMTS3/SERPINE2/TENM1/FBN1/CRISPLD2/NRP2/COMP/SERPINA5/SOD3/FN1/CLEC3B/CCN5/EFEMP2/NELL1/CCN3/COL23A1/CCN4/CCN2/FGF1/SELL/LPL/CFH/GPNMB/PCOLCE/TGFBR3/COLQ/ANOS1/PTCH1/LTBP2/THBS2/MPIG6B/RSPO4

GOMF_CATALYTIC_ACTIVITY_ACTING_ON_DNA	GOMF_CATALYTIC_ACTIVITY_ACTING_ON_DNA	1.00E-10	MSH3/RECQL5/NEIL2/BTAF1/ERCC2/TENT4B/RAD54L2/TOP2B/MCM8/TDG/XRCC6/EP400/DNASE1L1/MGME1/SMC3/TOP1/CDK7/RAD9A/PMS2/MYO18A/PMS1/TERF2/RPS3/RFC3/ALKBH4/RTEL1/XRCC5/XRCC2/RFC1/SMARCAD1/POLA1/ATAD5/TOP3A/FAN1/POLL/MSH6/DNASE2/POLH/DHX9/POLM/RUVBL1/XRCC1/POLE2/POLB/INO80/APEX2/DKC1/FANCM/RUVBL2/RFC4/PTGES3/PIF1/RAD54B/APTX/G3BP1/POLE3/UPF1/MUS81/SRCAP/ERCC5/SUPV3L1/MCM9/TDP1/RBBP4/DSCC1/MPG/WRNIP1/NTHL1/WRN/POLG/ASCC3/HELB/HMCES/RFC5/UNG/DDX11/MAP1S/XRCC3/DDX1/TOP1MT/ZGRF1/RBBP8/RFC2/DDX12P/POLD3/MSH2/DCLRE1B/ERCC6L/MCM7/GEN1/DMC1/NEIL3/POLE/MCM6/SMARCA4/ALKBH2/TWNK/ISG20/PCNA/DNA[illegible]

GOCC_RIBOSOME	GOCC_RIBOSOME	1.00E-10	DHX29/MRPS17/LINC01004/RPS26/MRPL38/RPS25/RPL27A/RPLP1/RPL13/MRPS23/DAP3/RPS15A/RPL11/MRPL24/MRPS15/MRPL57/RPS13/RPL34/RPL38/MRPL48/RPS24/MRPL9/RPS21/MRPL40/NUFIP2/RPL24/NSUN3/MRPL27/PNPT1/RPLP2/MRPS10/MRPL45/RPS11/UBA52/RPS8/RPS16/RPL22/RPS4X/RPS12/RPL23A/MRPS7/RPS6KL1/MRPL36/RRBP1/RPL15/MRPL32/RPL10A/MRPL20/MRPS33/RPS10/FAU/RPL32/MRPL17/RPS27L/MRPS30/RPL26/RPS14/RPL6/RPS9/MRPL14/RPL37A/RPL13A/SERP1/MTG2/RPL14/HSPA14/MRPL30/MRPL3/RPS18/MRPL18/RPS3/RPS5/RPL4/EIF3H/MRPL22/MRPL21/RPS23/RPL30/MRPL52/MRPL13/MRPS11/SF1/MRPL19/RPS3A/MRPL35/RPL12/RPL26L1/MRPS18B/MRPS9/MRPL42/RPL18[illegible]

GOCC_CONTRACTILE_FIBER	GOCC_CONTRACTILE_FIBER	1.00E-10	CASQ2/SYNM/MYH11/SYNPO2/DES/LDB3/ACTN2/PGM5/FLNC/LMOD1/CASQ1/JPH2/MYL9/ANKRD1/ANK2/PPP1R12B/CRYAB/TPM2/KCNA5/PDLIM3/ABCC9/ACTA2/TMOD1/SYNC/KLHL41/DMD/NEXN/ASB2/CFL2/MYL3/SMPX/FBXL22/CALD1/RYR3/FERMT2/ARHGEF25/FLNA/SVIL/CACNA1C/CSRP1/MYOM1/KY/CMYA5/SMTNL2/ACTA1/KCNJ8/ITGB1BP2/HRC/TPM1/ACTC1/FBXO32/ATP2B4/PALLD/LMOD3/PDLIM4/FHL5/SLC8A1/MMP2/SYNE1/TIMP4/FHOD3/CSRP2/PARVA/SORBS2/SYNPO/PDLIM7/ILK/HSPB1/RYR2/ACTN1/DST

GOMF_GLYCOSAMINOGLYCAN_BINDING	GOMF_GLYCOSAMINOGLYCAN_BINDING	1.00E-10	ELANE/MPO/REG3G/AZU1/CEL/SFRP1/APOH/VTN/REG1B/ITIH2/THBS4/CTSG/FGF10/SERPIND1/F2/PRELP/SLIT2/APOB/SUSD5/CCDC80/RSPO1/FGF7/ADAMTS8/SERPINA10/CHODL/DPYSL3/TNXB/GREM2/SLIT3/RSPO3/PTN/SMOC2/FGF2/VIT/CXCL6/DCN/LYVE1/LAYN/PCOLCE2/RSPO2/ADAMTS1/THBS1/ECM2/SELP/FSTL1/FGFR1/CCN1/ADAMTS3/SERPINE2/TENM1/FBN1/CRISPLD2/NRP2/COMP/SERPINA5/SHH/SOD3/FN1/CLEC3B/PGLYRP1/CCN5/EFEMP2/NELL1/CCN3/COL23A1/EPYC/CCN4/CCN2/FGF1/SELL/PRNP/LPL/VCAN/CFH/GPNMB/PCOLCE/TGFBR3/COLQ/ANOS1/PTCH1/ANXA6/BGN/LTBP2/THBS2/MPIG6B/RSPO4

GOCC_MITOCHONDRIAL_PROTEIN_CONTAINING_COMPLEX	GOCC_MITOCHONDRIAL_PROTEIN_CONTAINING_COMPLEX	1.00E-10	MRPL24/MRPS15/MRPL57/SUCLG1/NDUFV2/TIMM8A/MRPL48/MRPL9/MRPL40/PRORP/TOMM40L/DBT/NSUN3/BCKDHB/ATP5MC2/PAM16/MRPL27/PNPT1/NDUFB8/TIMM17B/NDUFC2/MRPS10/MRPL45/NDUFB5/DNAJC15/IDH3G/NDUFC1/MICU2/NDUFA1/MFN1/COX7B/MRPS7/IMMP1L/TIMM23/MRPL36/NDUFB4/UQCRB/ATP5MG/TIMM23B/COX6B1/NDUFS8/PDHX/MRPL32/UQCR11/NDUFB2/ATP5MF/COX7C/MRPL20/MRPS33/NDUFA8/TIMM17A/COX7A2L/MRPL17/MRPS30/ATP5F1C/NDUFA6/NDUFB10/ATP5PD/DLD/SUCLG2/NDUFS5/MRPL14/COX7A2P2/NDUFB3/HADHA/TIMM50/NDUFA12/NDUFA5/TOMM70/MRPL30/TIMM22/UQCRQ/MRPL3/MRPS36/HSD17B10/MRPL18/NDUFA9/TIMM8B/SAMM50/NDUFB6/MCCC1/MRPL22/MRPL21/CHCHD6/NDU[illegible]

GOCC_CONDENSED_CHROMOSOME	GOCC_CONDENSED_CHROMOSOME	1.00E-10	TP53BP1/CBX3/TPR/CHAMP1/SMARCD1/RIF1/NUDCD2/CTCF/SMC5/ORC2/BRD7/SMC3/WAPL/H3-3B/SUGT1/CENPS/DSN1/RAD9A/SPDL1/CHMP6/AHCTF1/SMARCC1/MACROH2A1/TUBG1/XPO1/NUP160/ACTL6A/PPP1CC/SEH1L/RAD9B/KAT5/H3-3A/SKA2/SMARCC2/CHMP2B/SGO2/SMARCB1/CHMP7/BRCA1/PSMC3IP/ITGB3BP/CHMP1A/NUP85/NUP43/TOPBP1/HSF1/AKAP8/NDE1/HNRNPU/CENPW/SLF1/LRWD1/NUF2/CHMP5/NEK2/CENPI/CKAP5/MTBP/SKA3/NCAPH2/ZW10/BRCA2/PINX1/RAD21/SMC4/LRPPRC/KNSTRN/TTK/HJURP/NCAPD2/NUP37/KNTC1/SMC1A/HMGB2/SGO1/SUV39H1/RANGAP1/KMT5C/SEC13/CENPO/ZWILCH/AURKB/NDC80/BUB1/SMARCD2/BUB3/SPAG5/CENPA/SMARCE1/ERCC6L/RRS1/DMC1/SPC25/SMA[illegible]

GOBP_NUCLEAR_CHROMOSOME_SEGREGATION	GOBP_NUCLEAR_CHROMOSOME_SEGREGATION	1.00E-10	TOP2B/ANAPC1/M1AP/DIS3L2/NEK6/TPR/BAG6/MAD2L2/CHAMP1/SMARCD1/CTCF/SMC5/BRD7/SMC3/WAPL/CENPS/DSN1/SPDL1/CHMP6/EML3/PPP2R1A/CDC23/SMARCC1/MACROH2A1/TUBG1/ACTL6A/RB1/SEH1L/KPNB1/TENT4A/KAT5/ANAPC15/ESCO1/ANAPC5/SMARCC2/STAG1/CHMP2B/NUP62/SGO2/TRIP13/SMARCB1/PDCD6IP/SFPQ/RAB11A/CHMP7/PSMC3IP/MCMBP/CHMP1A/NAA50/KMT5A/VPS4B/INO80/AKAP8/EHMT2/HNRNPU/SLF1/FANCM/SLX4/NAA10/ANAPC2/NUF2/CHMP5/NEK2/EME1/MUS81/RAN/DSCC1/NCAPH2/ZW10/PINX1/KATNB1/RAD21/BCL7A/SMC4/KNSTRN/TTK/NCAPD2/PDS5A/KNTC1/SMC1A/PSRC1/SGO1/PRC1/DDX11/MAP1S/XRCC3/FBXO5/SIRT7/ZWILCH/AURKB/NDC80/BUB1/SMARCD2/BUB3/SPAG5/KI[illegible]

GOBP_MUSCLE_CELL_DEVELOPMENT	GOBP_MUSCLE_CELL_DEVELOPMENT	1.00E-10	MYH11/LDB3/ACTN2/PGM5/FLNC/LMOD1/CASQ1/NKX2-5/MYL9/ANKRD1/ANK2/BVES/AKAP6/IGF1/TMOD1/KLHL41/DMD/ASB2/CFL2/DNER/PI16/RGS4/SGCD/P2RX2/CSRP1/ACTA1/AGT/TPM1/CNTNAP1/ACTC1/SYPL2/NFATC4/EDNRA/RGS2/LMOD3/TBX18/SLC8A1/COMP/SMO/FHOD3/PDGFRA/ALPK3/EFEMP2/ISL1/CSRP2/UCHL1/WNT10B/SORBS2

GOBP_EXTERNAL_ENCAPSULATING_STRUCTURE_ORGANIZATION	GOBP_EXTERNAL_ENCAPSULATING_STRUCTURE_ORGANIZATION	1.00E-10	ELANE/COL2A1/MYH11/PRSS1/VTN/ANGPTL7/CTSG/CMA1/HPSE2/FLRT2/CCDC80/HAS1/COL4A6/COL14A1/ABI3BP/ADAMTS8/SFRP2/ADAMTS18/TNXB/ADAMTS16/FBLN1/ADAMTSL3/CYP1B1/DDR2/MFAP4/COL11A2/SMOC2/PHLDB2/HMCN1/GREM1/DPT/VIT/MMP8/GPM6B/LAMA2/WT1/ELN/FBLN5/PTX3/COL8A1/SERPINF2/PRSS2/FKBP10/AGT/ITGA8/FOXF1/RECK/TGM1/CTSV/ADAMTS1/LAMA1/TLL1/COL4A5/MATN3/CAV1/LUM/ECM2/MMP3/CCN1/TGFB2/ADAMTS3/CRISPLD2/COL24A1/PRDM5/TNFRSF11B/MMP24/MMP2/COMP/IL6/ADAMTSL1/MMP7/COL3A1/MMP19/EMILIN1/PDGFRA/MMP16/FOXF2/COL8A2/EFEMP2/ANTXR1/CTSK/FBLN2/COL23A1/ADAMTS10/OLFML2B/FOXC2/COL16A1/CCN2/MMP23B/PAPLN/TCF15/LOX/HAS2[illegible]

GOBP_MUSCLE_CONTRACTION	GOBP_MUSCLE_CONTRACTION	1.00E-10	SPX/KCNA1/CASQ2/CHRM2/CNN1/SYNM/MYH11/ATP1A2/SCN7A/DES/TACR2/CHRNA3/LMOD1/PLN/MYLK/TACR1/KCNMA1/CASQ1/MYOCD/NKX2-5/MYL9/CTNNA3/SGCA/MIR145/ANK2/PPP1R12B/CRYAB/TPM2/KCNA5/GNAO1/HTR2A/ABCC9/ACTA2/TMOD1/KLHL41/DMD/DTNA/MYL3/SMPX/CALD1/HTR2B/FLNA/CACNA1C/SGCD/P2RX2/PTGER3/SMTN/MYOM1/CACNA1H/SCN4B/ACTA1/GUCY1A1/KCNJ8/HRC/CACNB2/KCNE4/AGT/TPM1/KCNH2/PRKG1/CHGA/ADRA1B/CHRNB4/GJC1/P2RX1/ATP2B4/SSPN/APBB1/DMPK/CAV1/EDNRA/FGF13/RGS2/LMOD3/STAC/NMUR1/GAMT/SLC8A1/COMP/CACNA2D1

GOCC_ENDOPLASMIC_RETICULUM_LUMEN	GOCC_ENDOPLASMIC_RETICULUM_LUMEN	1.00E-10	AFP/ALB/GCG/APOA2/AHSG/BCHE/APOA1/CASQ2/PENK/COL2A1/ERP27/VTN/FGA/CASQ1/CHRDL1/ITIH2/SERPIND1/CES1/F2/SPARCL1/APOB/COL4A6/COL14A1/FGG/SERPINA10/COL21A1/GPC3/CDH2/SCG3/SPON1/COL11A2/TNC/TF/IGFBP1/COL26A1/F7/GIP/COL8A1/HRC/FKBP10/FMO1/THBS1/COL4A5/MATN3/SELENOM/LTBP1/FSTL1/CCN1/C4A/FBN1/COL24A1/IGFBP4/IL6/ADAMTSL1/COL3A1/APOA4/SHH/PRSS23/P4HA3/FN1/IGFBP7/F8/COL8A2/IGFBP5/SDC2/COL23A1/EVA1A/PDGFD/COL16A1/COL6A2/GPX7/COL6A3/VCAN/MTTP/MXRA8/FKBP7/SCG2/COL1A2/COL4A4/LAMC1/SERPING1/ARSI/PDGFC/CLU/COL15A1/F10/COL10A1/ADAMTSL4/GPX8/CERCAM/FLT3/LGALS1/ADAMTS5/SRL/COL1A1/STC2/COL5A1/T[illegible]

GOMF_SULFUR_COMPOUND_BINDING	GOMF_SULFUR_COMPOUND_BINDING	1.00E-10	ELANE/MPO/AZU1/CEL/SFRP1/APOH/VTN/THBS4/CTSG/FGF10/SERPIND1/HPSE2/SULT2A1/F2/PRELP/SLIT2/APOB/CCDC80/RSPO1/FGF7/ADAMTS8/SERPINA10/DPYSL3/TNXB/GREM2/SLIT3/RSPO3/PTN/SMOC2/FGF2/CXCL6/SOAT2/PCOLCE2/RSPO2/P2RX1/ADAMTS1/THBS1/ECM2/SELP/FSTL1/FGFR1/CCN1/ADAMTS3/SERPINE2/TENM1/FBN1/CRISPLD2/NRP2/COMP/SERPINA5/SOD3/FN1/CLEC3B/CCN5/EFEMP2/NELL1/CCN3/COL23A1/CCN4/CCN2/FGF1/SELL/LPL/CFH/RYR2/GPNMB/PCOLCE/TGFBR3/COLQ/ANOS1/CHST15/PTCH1/ANXA6/LTBP2/THBS2/ACACB/MPIG6B/RSPO4/ADAMTS5/CD34/LRP1/OGDHL/COL5A1/NDNF/NRP1/FBLN7/HS3ST5/LXN/FGFR2/POSTN/GSTM2/LIPC

GOBP_MUSCLE_TISSUE_DEVELOPMENT	GOBP_MUSCLE_TISSUE_DEVELOPMENT	1.00E-10	HAND1/SMYD1/NPPB/MYH11/ACTN2/PGM5/PLN/MYLK/CASQ1/JPH2/MYOCD/NKX2-5/MEOX2/ANKRD1/MIR145/POPDC2/BVES/ALDH1A2/AKAP6/IGF1/KLHL41/DMD/ASB2/CFL2/MYL3/DNER/RBM24/NOG/KCNK2/HLF/PI16/RGS4/ZFPM2/SVIL/ITGA7/SGCD/P2RX2/FGF2/CSRP1/SGCG/TNN/GREM1/HOXD10/IGF2/CDON/ACTA1/WT1/ELN/AGT/TPM1/CNTNAP1/ACTC1/ITGA8/RXRG/GJC1/GLI1/CAV1/EDNRA/RGS2/LMOD3/TBX18/TGFB2/RBP4/SLC8A1/HEYL/PKD2/COL3A1/HOPX/SHH/SMO/FHOD3/PDGFRA/OSR1/DKK1/ALPK3/EFEMP2/ISL1/FZD7/CSRP2/POPDC3/IGFBP5/SOX15/TCF21/WNT10B/GJA1/FOXC2/SORBS2/NEURL1/S1PR1/LOX/TENM4/HEG1/RYR2/NOX4/TGFBR3/MEF2C/MIR222/ADAMTS9/PTCH1/TWIST1[illegible]

GOBP_MUSCLE_ORGAN_DEVELOPMENT	GOBP_MUSCLE_ORGAN_DEVELOPMENT	1.00E-10	HAND1/SMYD1/DES/MYLK/CASQ1/JPH2/MYOCD/NKX2-5/FHL1/MEOX2/ANKRD1/TAGLN/SGCA/MIR145/POPDC2/BVES/CRYAB/SPEG/IGF1/KLHL41/DMD/ASB2/CFL2/MYL3/DNER/RBM24/CHODL/NOG/HLF/ZFPM2/SVIL/ITGA7/SGCD/P2RX2/MKX/SGCG/SMTN/KY/HOXD10/CACNA1H/IGF2/CDON/ACTA1/LAMA2/WT1/ELN/ITGB1BP2/TPM1/CNTNAP1/ACTC1/RXRG/MYMX/CAV1/USP2/EDNRA/EVC/LMOD3/TGFB2/HEYL/COL3A1/CTF1/SHH/SMO/EFEMP2/ISL1/SGCE/POPDC3/SOX15/TCF21/WNT10B/FOXC2/NEURL1/S1PR1/TCF15/LOX/COL6A3/HEG1/POU6F1/RYR2/TGFBR3/MEF2C/EGR3/TWIST1/RIPOR2/ADGRB1/HOXD9/MSC/ITGA11/LEF1/ACVR1/ADARB1

GOBP_REGULATION_OF_SYSTEM_PROCESS	GOBP_REGULATION_OF_SYSTEM_PROCESS	1.00E-10	APOA2/SPX/CARTPT/KCNA1/VIP/SOX10/APOA1/NPPB/CASQ2/TAFA4/CHRM2/RELN/CNN1/ATP1A2/AGTR1/DES/TACR2/HAND2/ADIPOQ/HSPB7/NEUROD1/CHRNA3/FGA/PLN/TACR1/KCNMA1/CASQ1/MYOCD/NKX2-5/FGF10/MYL9/CTNNA3/SGCA/MIR145/POPDC2/ANK2/PPP1R12B/BVES/GRIN2A/AKAP6/TMEM100/SCT/KCNA5/SLC8A2/HTR2A/ABCC9/IGF1/DMD/FGG/MYL3/NPTX2/KCNH6/IGSF11/PI16/RGS4/FLNA/ATP1B2/CACNA1C/ADRA1D/PTGER3/CACNG4/JAM2/TRPC1/CACNA1H/NLGN4X/NLGN1/SCN4B/NR4A3/SMTNL2/NPTX1/GUCY1A1/IL33/HRC/WASF3/PDE9A/CACNB2/KCNE4/AGT/TPM1/NPTXR/KCNH2/PRKG1/CHGA/FBXO32/ADRA1B/CHRNB4/GJC1/P2RX1/ATP2B4/DMPK/CAV1/EDNRA/FGF13/RGS2/HGF/NPR[illegible]

GOBP_MUSCLE_SYSTEM_PROCESS	GOBP_MUSCLE_SYSTEM_PROCESS	1.00E-10	SPX/KCNA1/CASQ2/CHRM2/CNN1/SYNM/MYH11/ATP1A2/SCN7A/DES/TACR2/HAND2/MYOC/CHRNA3/LMOD1/PLN/MYLK/TACR1/KCNMA1/CASQ1/MYOCD/NKX2-5/MYL9/CTNNA3/SGCA/MIR145/ANK2/PPP1R12B/AKAP6/CRYAB/TPM2/KCNA5/GNAO1/HTR2A/ABCC9/ACTA2/IGF1/TMOD1/KLHL41/DMD/DTNA/MYL3/SMPX/CALD1/HTR2B/PI16/RGS4/FLNA/CACNA1C/SGCD/P2RX2/PTGER3/SMTN/MYOM1/CACNA1H/SCN4B/NR4A3/ACTA1/GUCY1A1/KCNJ8/HRC/PDE9A/CACNB2/KCNE4/AGT/TPM1/KCNH2/PRKG1/CHGA/FBXO32/ADRA1B/CHRNB4/GJC1/P2RX1/ATP2B4/SSPN/APBB1/DMPK/CAV1/EDNRA/FGF13/RGS2/LMOD3/STAC/NMUR1/GAMT/SLC8A1/KLF15/COMP/CACNA2D1

GOBP_STRIATED_MUSCLE_CELL_DIFFERENTIATION	GOBP_STRIATED_MUSCLE_CELL_DIFFERENTIATION	1.00E-10	SMYD1/MYH11/LDB3/ACTN2/PGM5/FLNC/LMOD1/CASQ1/MYOCD/NKX2-5/MYL9/ANKRD1/POPDC2/BVES/AKAP6/ERVW-1/IGF1/TMOD1/KLHL41/ASB2/CFL2/DNER/RBM24/ADGRB3/CDH2/PI16/RGS4/SGCD/P2RX2/CSRP1/GREM1/JAM2/CACNA1H/IGF2/CDON/ACTA1/WT1/AGT/TPM1/CNTNAP1/ACTC1/PLPP7/MYMX/DMPK/RGS2/LMOD3/TBX18/SLC8A1/TMEM119/HOPX/MYEF2/SHH/AVPR1A/SMO/FHOD3/PDGFRA/DKK1/ALPK3/ISL1/FZD7/CSRP2/POPDC3/IGFBP5/CCN3/WNT10B/SORBS2/PLEKHO1/EHD2/NOX4/MEF2C/MIR222/MAMSTR/IRX3/RIPOR2/TBX5/ADGRB1

GOBP_MUSCLE_CELL_DIFFERENTIATION	GOBP_MUSCLE_CELL_DIFFERENTIATION	1.00E-10	SMYD1/MYH11/LDB3/ACTN2/PGM5/FLNC/LMOD1/CASQ1/MYOCD/NKX2-5/RBPMS2/FGF10/MYL9/ANKRD1/MIR145/POPDC2/ANK2/BVES/AKAP6/ERVW-1/SPEG/IGF1/TMOD1/KLHL41/DMD/ASB2/CFL2/DNER/RBM24/ADGRB3/CDH2/PI16/RGS4/SGCD/P2RX2/CSRP1/GREM1/JAM2/CACNA1H/IGF2/CDON/ACTA1/LAMA2/WT1/ZEB1/AGT/TPM1/CNTNAP1/ACTC1/ITGA8/FOXF1/SYPL2/NFATC4/LAMA1/PRDM6/PLPP7/MYMX/DMPK/EDNRA/SMARCD3/RGS2/LMOD3/TBX18/SLC8A1/TMEM119/SYNE1/COMP/HOPX/MYEF2/SHH/AVPR1A/SMO/FHOD3/PDGFRA/DKK1/ALPK3/EFEMP2/ISL1/FZD7/CSRP2/POPDC3/IGFBP5/CCN3/UCHL1/WNT10B/SORBS2/LOX/PLEKHO1/EHD2/RAMP2/QKI/NID1/NOX4/ACTN1/MEF2C/MIR222/[illegible]

GOBP_SECOND_MESSENGER_MEDIATED_SIGNALING	GOBP_SECOND_MESSENGER_MEDIATED_SIGNALING	1.00E-10	AZU1/NPPB/CASQ2/ATP1A2/AGTR1/NEUROD1/PLN/ADCYAP1R1/CASQ1/ADCYAP1/ANK2/GRIN2A/AKAP6/TMEM100/SCT/SLC8A2/RGN/IGF1/DMD/HTR2B/CAP2/ADCY2/PTGFR/IRAG1/PDE7B/CACNA1C/SGCD/P2RX2/RCAN2/ADCY1/GUCY1A1/GIP/HRC/PDE9A/AGT/PDE3A/CD36/PRKG1/MAPT/NFATC4/ATP2B4/THBS1/GUCY1B1/RASD2/DMPK/CX3CR1/SELP/LPAR1/PDE2A/RUNDC3A/NMUR1/NPR1/HPCA/SLC8A1/AVPR1A/SCTR/FFAR4/GLP1R/RASD1/LRRK2/GUCY1A2/PRNP/BST1/CXCR2/ITPR1/RYR2/FPR1/CLIC2/GNAI1/PDE5A/CXCR1/NPR2/SELE/ADGRB2/CXCR4/CCR7/ACKR3/NCALD/NDNF/MYOZ1/EDNRB/PDE3B/NPPC/APLNR/EDN2/FPR2/ACKR2/P2RY12/GSTM2/CHRM3/LMCD1/TBXA2R

GOBP_REGULATION_OF_MEMBRANE_POTENTIAL	GOBP_REGULATION_OF_MEMBRANE_POTENTIAL	1.00E-10	UCN3/KCNA1/CASQ2/TAFA4/RELN/ATP1A2/SCN7A/GRIK3/ACTN2/MYOC/CHRNA3/KCNB1/PLN/TACR1/KCNMA1/KCNK3/GRIK1/FHL1/CTNNA3/POPDC2/ANK2/BVES/GRIN2A/AKAP6/KCNA5/SEZ6/SLC8A2/DMD/GRIK5/KCNK2/KCNH6/GLRB/IGSF11/RGS4/FLNA/ATP1B2/CACNA1C/P2RX2/CNR1/BEST2/DCN/CACNA1H/NLGN4X/NLGN1/SCN4B/KCNJ8/CACNB2/KCNE4/CD36/KCNH2/CNTNAP1/MAPT/RGS7BP/CHRNB4/GJC1/P2RX1/PID1/DMPK/CAV1/CNR2/SCN3A/FGF13/TBX18/SLC1A7/SLC8A1/CACNA2D1/PRKN/INSYN1/ADRB2/SPART/PIEZO2/KCNQ3/POPDC3/MPP2/NLGN3/LRRK2/GJA1/TMEM108/DLG4/CELF4/CLDN19/SLC26A3/KCND2/RYR2/MEF2C/NRCAM/NLGN2/TCL1A/GNA14/FGF12/GABBR1/GABRR1/TBX5/CHRNB2

GOCC_VESICLE_LUMEN	GOCC_VESICLE_LUMEN	1.00E-10	ELANE/ALB/MPO/PRTN3/GCG/AZU1/DEFA4/AHSG/HP/APOA1/PENK/RNASE3/APOH/ACTN2/BPI/FGA/CTSG/PCSK1/APOB/IGF1/RNASE2/FGG/GRP/TTR/SCG3/TF/MMP8/IGF2/ITIH3/PTX3/GIP/F13A1/SERPINF2/PRSS2/MMRN1/TGFB3/LEFTY2/THBS1/CAMP/S100A8/HGF/SERPINA3/TGFB2/FCN1/CRISPLD2/SELENOP/QPCT/S100A9/SERPINE1/ORM2/PROS1/FN1/TIMP2/CLEC3B/PGLYRP1/F8/A2M/RETN/ISLR

GOBP_REGULATION_OF_TRANS_SYNAPTIC_SIGNALING	GOBP_REGULATION_OF_TRANS_SYNAPTIC_SIGNALING	1.00E-10	SYT4/BCHE/CHRM2/RELN/ATP1A2/FRRS1L/TACR2/GRIK3/ADIPOQ/CHRNA3/KCNB1/TACR1/CHRDL1/GRIK1/SCGN/GRIN2A/SCT/SLC8A2/HTR2A/NPTX2/GRIK5/NOG/IGSF11/CDH2/MAP1B/RGS4/ROR2/NGF/PLPPR4/PTN/MIR320C2/CACNG4/CNR1/MAP1A/NLGN4X/NLGN1/PTPRD/NPTX1/LAMA2/ADCY1/GIP/CNTN4/CACNB2/AGT/NPTXR/NLGN4Y/MAPT/NFATC4/CHRNB4/AKAP12/P2RX1/DMPK/PRKAR2B/CX3CR1/CNR2/SORCS2/SERPINE2/PLCL1/CLSTN2/PRRT1/PRKCB/PRKN/CDH11/CACNA1A/FAM107A/MIR342/SCTR/CCL2/SNAP25/DKK1/ADRB2/SYT11/NTF3/MPP2/RETN/OXTR/NLGN3/LRRK2/SNCG/BRSK1/TSHZ3/LRRC4/MIR320D1/TMEM108/DLG4/NEURL1/CELF4/PRNP/LRRC4C/MEF2C/NLGN2/S100B/APBA1/JPH3/GABBR1/SYT1[illegible]

GOCC_CELL_BODY	GOCC_CELL_BODY	1.00E-10	SYT4/KCNA1/GHRH/SEZ6L/VIP/TAC1/PENK/CHRM2/ACTG2/ATP1A2/GRIK3/STMN2/CHRNA3/KCNB1/TACR1/NGFR/PCSK1/EPHA7/ADCYAP1/ASCL1/GPM6A/NEGR1/GFRA1/TMEM100/CRYAB/APOD/SEZ6/SLC8A2/HTR2A/APOB/ACTA2/GAP43/PTPRN/GHR/RIC3/DNER/GRIK5/TUBB4A/KCNK2/DPYSL3/MAP1B/NPY/ROR2/FLNA/ATP1B2/CACNA1C/TTLL7/P2RX2/PRKAA2/IGF2BP1/TNN/CACNG4/PCSK2/NDN/MAP1A/PDE1A/DIXDC1/ACTA1/GIP/PDE9A/ACTC1/ITGA8/TGFB3/MAPT/DLG2/RGS7BP/AKAP12/SV2A/SERPINF1/PRKAR2B/CX3CR1/CNR2/LPAR1/CKB/SORCS2/PDE1C/TGFB2/C4A/ITGA1/L1CAM/HPCA/PDE1B/FEZ1/SLC8A1/RLBP1/FLRT1/CACNA1A/PTPN13/TMEM132E/SYT11/KIF5A/CCN3/TNS2/UCHL1/LRRK2/CD200/DPYSL5/[illegible]

GOBP_BEHAVIOR	GOBP_BEHAVIOR	1.00E-10	GCG/SYT4/CARTPT/GHRH/VIP/BCHE/TAC1/PENK/RELN/ATP1A2/TACR2/HAND2/CHRNA3/TACR1/SLC2A4/THBS4/OBP2B/NRXN3/NRXN2/ADCY5/NEGR1/GRPR/GRIN2A/SEZ6/SLC8A2/HTR2A/TMOD1/PTCHD1/NPTX2/NOG/HTR2B/KCNK2/ADGRB3/GRP/GLRB/NPY/CNTN1/NGF/ATP1B2/PTN/C3orf70/P2RX2/SLC18A2/DAB1/CNR1/MAP1A/HOXD10/NLGN4X/NLGN1/NR4A3/CXCL12/GLI3/ADCY1/GIP/LRRN4/RNF180/AGT/LEPR/LGI4/ITGA8/NLGN4Y/CHL1/MAPT/NFATC4/CHRNB4/P2RX1/THBS1/SERPINF1/SOBP/RASD2/VLDLR/USP2/PRKAR2B/CX3CR1/FGF13/C1QL1/MEIS1/SERPINE2/SELENOP/PDE1B/ITGA5/PRRT1/PRKN/CDH23/AVPR1A/PREX2/SNAP25/DKK1/SGIP1/SLITRK6/SYT11/PBX3/NTF3/GLP1R/EFNB3/RETN/UCHL1/OXTR[illegible]

GOCC_DENDRITIC_TREE	GOCC_DENDRITIC_TREE	1.00E-10	SYT4/KCNA1/PENK/CHRM2/NSG2/RELN/ATP1A2/GRIK3/ACTN2/CHRNA3/KCNB1/TACR1/NGFR/PSD/PCSK1/EPHA7/SCGN/GPM6A/FAT3/NEGR1/GRIN2A/CRYAB/APOD/SEZ6/SLC8A2/HTR2A/GAP43/NFASC/DNER/GRIK5/HTR2B/GLRB/ADCY2/MAP1B/MAP6/ROR2/KCNIP1/NGF/CACNA1C/TTLL7/PRKAA2/IGF2BP1/MPDZ/TNN/PCSK2/MAP1A/NLGN4X/NLGN1/ITGA8/CHL1/MAPT/RGS7BP/SV2A/NCS1/PRKAR2B/CX3CR1/CNR2/CHRM4/MARK1/FGF13/LPAR1/CKB/SORCS2/C4A/L1CAM/PDLIM4/HPCA/MAGEE1/BAG2/FEZ1/CLSTN2/SLC8A1/C4B/PALMD/SAMD4A/SMO/CTTNBP2/PALM/KIF1A/LAMP5/SYT11/BSN/KIF5A/NTF3/SGCE/CCN3/TNS2/MPP2/KIRREL1/LRRK2/DPYSL5/KCND1/SORBS2/LRRC4/SYNPO/CTNND2/TMEM108/DLG4/TRIM9/N[illegible]

GOBP_MITOTIC_CELL_CYCLE_CHECKPOINT_SIGNALING	GOBP_MITOTIC_CELL_CYCLE_CHECKPOINT_SIGNALING	1.15E-10	MAD2L1BP/CDK2/UIMC1/RPS27L/RPL26/TPR/MAD2L2/RPA2/CRADD/PPP1R10/NABP2/RAD9A/SPDL1/CDK5RAP3/NAE1/RAD9B/ANAPC15/NEK11/XPC/TRIP13/CHEK2/BRCC3/BRCA1/TOPBP1/PML/NUF2/BARD1/EME1/MUS81/DONSON/ZFYVE19/ZW10/TRIAP1/CCNG1/TAOK2/RINT1/TTK/PRKDC/KNTC1/PIDD1/XRCC3/FZR1/CASP2/ZWILCH/AURKB/NDC80/BUB1/BUB3/RFWD3/RBBP8/CDC14B/NBN/MSH2/TIPIN/GEN1/GTSE1/SPC25/CDK1/PLK2/EME2/CENPF/INCENP/CHEK1/SPC24/BUB1B/BLM/CDC20/TP53/TICRR/CLSPN/CHMP4C/FANCD2/CCNB1/HASPIN/MAD2L1/DTL/MDM2/PLK1/BIRC5/CDCA8/CCND1/ZWINT/CDT1/ORC1/MUC1/PBK

GOCC_CHROMOSOME_TELOMERIC_REGION	GOCC_CHROMOSOME_TELOMERIC_REGION	1.24E-10	H4C13/XRCC6/TP53BP1/CBX3/RIF1/RPA2/SMC5/ORC2/H3-3B/DDB1/PPP1R10/ATR/NABP2/THOC6/TERF2/TFIP11/HAT1/MACROH2A1/PPP1CC/H3-3A/RTEL1/XRCC5/HDAC2/SMCHD1/ACD/H4C9/CHEK2/RPA1/H4C4/STN1/XRCC1/H4C3/NAT10/SLF1/SLX4/PML/LRWD1/POLR2B/PTGES3/PIF1/MEN1/KDM1A/UPF1/HNRNPA2B1/H4C2/PARP1/RBBP4/H4C1/WRNIP1/WRN/BRCA2/PINX1/ZSCAN4/PRKDC/XRCC3/SIRT6/NBN/PPP1CA/GAR1/TELO2/MSH2/DCLRE1B/WRAP53/NHP2/RAD51AP1/MCM7/EZH2/DMC1/TNKS/MTA2/MCM6/THOC3/CDK1/ALYREF/CHEK1/PCNA/BLM/RAD51/MCM3/FEN1/DNA2/TERT/H2AX/MCM5/ORC5/RECQL4/POLD1/MCM4/MCM2/ORC1

GOBP_REGULATION_OF_BLOOD_CIRCULATION	GOBP_REGULATION_OF_BLOOD_CIRCULATION	1.46E-10	SPX/CASQ2/CHRM2/ATP1A2/AGTR1/DES/HSPB7/FGA/PLN/TACR1/NKX2-5/CTNNA3/POPDC2/ANK2/BVES/KCNA5/SLC8A2/HTR2A/ABCC9/DMD/FGG/MYL3/KCNH6/RGS4/FLNA/ATP1B2/CACNA1C/ADRA1D/TRPC1/CACNA1H/SCN4B/SMTNL2/HRC/CACNB2/KCNE4/AGT/TPM1/KCNH2/CHGA/ADRA1B/GJC1/P2RX1/ATP2B4/DMPK/CAV1/EDNRA/FGF13/RGS2/TBX18/TGFB2/SLC8A1/MMP2/CACNA2D1/CORIN/HOPX/AVPR1A/CYSLTR1/CELF2/ISL1/GLP1R/OXTR/THRB/GJA1/CCN2/AVPR2/RNLS/RYR2/STC1/FGB/CLIC2/PDE5A

GOBP_ADENYLATE_CYCLASE_MODULATING_G_PROTEIN_COUPLED_RECEPTOR_SIGNALING_PATHWAY	GOBP_ADENYLATE_CYCLASE_MODULATING_G_PROTEIN_COUPLED_RECEPTOR_SIGNALING_PATHWAY	2.29E-10	GCG/UCN3/GHRH/VIP/CHRM2/GRIK3/PLN/ADCYAP1R1/ADCYAP1/GLP2R/ADCY5/NPR3/GNAO1/ADGRB3/ADCY2/PTGFR/ADGRD1/FLNA/ADRA1D/PTGER3/GABBR2/CNR1/PTH1R/VIPR2/ADCY1/GIP/CHGA/ADRA1B/AKAP12/ATP2B4/LPAR3/EDNRA/CNR2/CHRM4/RGS2/LPAR1/PDE2A/S1PR3/GNAL/SCTR/PALM/ADRB2/GLP1R/SSTR2/PTHLH/PTGIR/S1PR1/GPR161/AVPR2/PTGER1/ABCA1/RAMP2/FPR1/GNAI1/GNA14/GABBR1/ITGB3/UCN2/GNAZ/ADGRB2/ADGRB1/P2RY1/GPER1/ADGRG2/PRKACB/S1PR5/ADGRL3/ADGRL2/RAMP3/FPR2/ADGRL4/P2RY12/RAMP1/CHRM3/TBXA2R/ADCY4/GPR37/PTH2R

GOMF_RIBONUCLEOPROTEIN_COMPLEX_BINDING	GOMF_RIBONUCLEOPROTEIN_COMPLEX_BINDING	4.32E-10	SNRPG/RPN2/OXA1L/FMR1/EIF6/EIF3K/STRAP/SRP9/SECISBP2/SMG6/NME1/TMEM223/XPO5/IFIH1/CPSF6/EIF1/PRPF6/PHF6/DNAJC2/BAG6/TMEM147/MAIP1/GEMIN5/NAA15/DERL1/ERAL1/CD2BP2/TIMM50/SRPRA/PRPF31/TMA16/DHX33/PPIH/WDR12/SRP72/PRMT7/ABCE1/NOMO3/SNRPB/NOMO1/EEFSEC/SRP68/RACK1/DHX9/PTCD3/LETMD1/TACO1/EIF4A3/CCDC47/EIF4B/HNRNPU/HSPA5/MTOR/NAA10/SEC61A1/DERL3/SND1/EIF4H/SNRPD1/PRMT3/EIF2S1/EEF2/CKAP5/SERBP1/GCN1/MRRF/OLA1/UNG/LARP1/PYM1/MTIF2/PES1/RPSA/MRPS27/SNRPA/UHMK1/NOMO2/PPP1CA/SPATA5/EZH2/PRMT5/SPATA5L1/NPM1/GUF1/NOLC1/ZNF598/ZC3H12A/EIF3C/BOP1/ERI1/GEMIN4/PIWIL1/C1QBP/EIF5A/MDM2/NCLN/L[illegible]

GOCC_PROTEIN_DNA_COMPLEX	GOCC_PROTEIN_DNA_COMPLEX	4.48E-10	H2AC19/SUZ12/ZBTB17/IRF4/H4C13/NFYC/XRCC6/RPA3/H3C12/EP400/SOX12/TCF7L2/RPA2/H3-3B/TOP1/POLR2H/BRD8/H2BC7/MRGBP/H1-10/TEAD4/TERF2/H3C10/ZNHIT1/MACROH2A1/ACTL6A/KAT5/H3-3A/XRCC5/H2AW/ACD/SPHK2/POLA1/ACTR6/H4C9/H3C1/PRIM2/H2BC11/RPA1/H4C4/STN1/RUVBL1/H4C3/SLF1/RUVBL2/H2BC9/H1-2/SP1/H4C2/H2BC18/PARP1/SRCAP/H2AC18/BRF2/GTF2H3/DONSON/BRF1/H4C1/KDM1B/PRKDC/GINS1/H2BC10/H1-3/POLA2/H3C13/H3C2/H3C7/POLD3/H2AC20/CENPA/WDR18/GINS2/PRIM1/H2BC12/MCM7/NPM1/MCM6/H2AZ1/H2BC15/POLD2/H2AC13/PCNA/GINS3/H2BC14/H3C6/MCM3/CDC45/TERT/H2AC21/H2AX/MCM5/JUP/H3C8/H2BC13[illegible]

GOMF_CATALYTIC_ACTIVITY_ACTING_ON_A_TRNA	GOMF_CATALYTIC_ACTIVITY_ACTING_ON_A_TRNA	6.57E-10	NSUN6/PRORP/AARS2/NSUN3/RARS2/GATC/TRNT1/TARBP1/POP5/ALKBH8/TYW3/DARS2/PTRHD1/LARS1/YARS2/TRIT1/PSTK/FARS2/THUMPD3/THUMPD2/TRMO/TRMT61B/AARS1/FTSJ1/TRPT1/MTFMT/WARS2/PARS2/TARS1/CARS2/GARS1/TSEN34/CARS1/DALRD3/HARS1/TSEN2/POP4/SARS2/TRMT11/QTRT2/METTL2A/FARSB/METTL2B/TRMT10A/TRMT2A/NSUN2/MRPL58/LRRC47/PUS1/GATB/TRMU/RARS1/NARS1/POP7/WDR4/MARS1/HARS2/DTWD2/EPRS1/KARS1/DTD2/EARS2/NT5C3A/RPP25/DUS2/THG1L/QARS1/QTRT1/IARS1/SARS1/TARS2/TRMT44/ELAC2/DUS1L/IARS2/VARS1/TRMT1/SEPSECS/YARS1/NARS2/DUS3L/LARS2/MARS2/FARSA/POP1/WARS1/QRSL1

GOCC_BLOOD_MICROPARTICLE	GOCC_BLOOD_MICROPARTICLE	7.66E-10	ALB/APOA2/AHSG/HP/BCHE/APOA1/ACTG2/PRSS1/VTN/FGA/ITIH2/F2/FGG/TF/ACTA1/F13A1/SERPINF2/HSPA2/AMBP/AGT/ACTC1/SERPINA3/C4A/C4B/APOA4/ORM2/HBB/PROS1/FN1/KRT1/A2M/C1S/HSPA7/STOM/C1R/IGHG4/CFH/FGB/HSPA1A/SERPING1/CLU

GOBP_PROTEIN_DNA_COMPLEX_SUBUNIT_ORGANIZATION	GOBP_PROTEIN_DNA_COMPLEX_SUBUNIT_ORGANIZATION	8.00E-10	H3C10/ZNHIT1/SMARCC1/MACROH2A1/ACTL6A/RB1/KAT5/H3-3A/SMARCC2/H2AW/POLR1E/MED14/TAF1C/GMNN/BAZ2A/TAF6L/SART3/ACTR6/MED25/H4C9/H3C1/SMARCB1/MED23/PIH1D1/MED16/H2BC11/H4C4/NAP1L4/SETSIP/RUVBL1/GRWD1/ITGB3BP/RRN3/RNF4/MYC/MIS18A/H4C3/NASP/CENPW/BRD2/RUVBL2/H2BC9/MED15/SET/MED9/SPTY2D1/H1-2/POLE3/THRA/H4C2/CENPI/H2BC18/SRCAP/ANP32B/ANP32E/MCM3AP/BRF2/BRF1/H4C1/MED26/HJURP/KNTC1/H2BC10/HMGB2/H1-3/TAF5/MED1/SSRP1/LIN54/CENPO/H3C13/H3C2/SMARCD2/SIRT6/MED18/H3C7/GTF2A2/CENPA/SMARCE1/SOX9/H2BC12/DMC1/NPM1/SMARCA4/H2BC15/CENPX/CENPK/CENPF/CENPV/HNF1B/HMGA1/[illegible]
GOBP_REGULATION_OF_LIPASE_ACTIVITY	GOBP_REGULATION_OF_LIPASE_ACTIVITY	1.06E-09	PLA2G1B/PNLIP/APOA2/APOA1/APOC3/AGTR1/APOH/ADCYAP1R1/HTR2A/PLA2G5/HTR2B/FGF2/GPIHBP1/AGT/EDNRA/RGS2/LPAR1/FGFR1/CCN1/NMUR1/HPCA/ESR1/APOA4/AVPR1A/PDGFRA

GOBP_STRIATED_MUSCLE_TISSUE_DEVELOPMENT	GOBP_STRIATED_MUSCLE_TISSUE_DEVELOPMENT	1.30E-09	HAND1/NPPB/MYH11/ACTN2/PGM5/PLN/JPH2/MYOCD/NKX2-5/ANKRD1/BVES/ALDH1A2/AKAP6/IGF1/ASB2/MYL3/NOG/KCNK2/PI16/RGS4/ZFPM2/SGCD/FGF2/SGCG/GREM1/WT1/AGT/TPM1/ACTC1/GJC1/GLI1/EDNRA/RGS2/TBX18/TGFB2/RBP4/SLC8A1/HOPX/SHH/FHOD3/PDGFRA/DKK1/ALPK3/ISL1/FZD7/SOX15/GJA1/FOXC2/SORBS2/S1PR1/LOX/TENM4/HEG1/RYR2/NOX4/TGFBR3/MEF2C/MIR222/ADAMTS9/TWIST1/IRX3/TBX5/MSC/LEF1/ACVR1/PDGFRB/HAMP/SEMA3C/NDRG4

GOCC_PERIKARYON	GOCC_PERIKARYON	2.32E-09	KCNA1/GHRH/VIP/PENK/GRIK3/KCNB1/NGFR/PCSK1/ADCYAP1/TMEM100/CRYAB/SLC8A2/GAP43/PTPRN/GRIK5/MAP1B/NPY/FLNA/CACNA1C/TTLL7/PCSK2/NDN/PDE9A/ITGA8/DLG2/RGS7BP/CNR2/SORCS2/ITGA1/HPCA/SYT11/KIF5A/LRRK2/PRPH/SYNPO/CTNND2/NEURL1/KCND2/SEPTIN4/SLC2A3

GOBP_DNA_INTEGRITY_CHECKPOINT_SIGNALING	GOBP_DNA_INTEGRITY_CHECKPOINT_SIGNALING	2.75E-09	CDK2/UIMC1/RPS27L/RPL26/TP53BP1/HINFP/RPA2/CRADD/FBXO4/PPP1R10/ATR/RAD9A/CDK5RAP3/NAE1/RAD9B/NEK11/USP28/XPC/CUL4A/CHEK2/PRPF19/BRCC3/BRCA1/INTS7/TOPBP1/PML/BARD1/EME1/MUS81/DONSON/ATRIP/BRCA2/TRIAP1/CCNG1/TAOK2/RINT1/PRKDC/PIDD1/FZR1/CASP2/CCAR2/RFWD3/CDC14B/NBN/TELO2/MSH2/TIPIN/GTSE1/CDK1/PLK2/EME2/TTI2/FBXO6/TIMELESS/CHEK1/E2F1/DOT1L/BLM/TP53/RAD51/TICRR/CLSPN/FANCD2/DNA2/CDC45/DTL/H2AX/MDM2/PLK1/CCND1/WDR76/BRIP1/CDT1/ORC1/MUC1/PBK

GOBP_TRNA_PROCESSING	GOBP_TRNA_PROCESSING	2.73E-09	CDK5RAP1/DUS4L/PUS3/RPP14/TRMT61A/TRMT10C/THADA/FAM98B/TRMT2B/RPP38/TRMT5/NSUN6/PRORP/AARS2/NSUN3/CLP1/FAM98A/LSM6/TRMT112/TRNT1/TARBP1/LAGE3/POP5/TRMT6/ALKBH8/TYW3/TRIT1/ELP1/FARS2/MTO1/THUMPD3/THUMPD2/ELP5/TRMO/ADAT1/TRMT61B/HSD17B10/AARS1/FTSJ1/TRPT1/MTFMT/THUMPD1/URM1/TRUB2/TSEN34/DALRD3/TSEN2/POP4/TRMT11/QTRT2/METTL2A/METTL2B/RTCB/TRMT10A/TRMT2A/NSUN2/ELP6/NAT10/TRUB1/CTU1/PUS1/TRMU/POP7/WDR6/WDR4/GON7/DTWD2/KARS1/ELP3/PUS7/RPP25/PUSL1/CTU2/DUS2/THG1L/POLR3K/ADAT2/QTRT1/RPUSD4/SARS1/YRDC/DDX1/TRMT44/ELAC2/DUS1L/TRMT1/GRSF1/TSEN54/RPP25L/C9orf64/GTPBP3/DUS3L/POP1/ADAT3

GOCC_SARCOLEMMA	GOCC_SARCOLEMMA	2.65E-09	SYNM/ATP1A2/DES/PGM5/FLNC/KCNB1/CASQ1/SLC2A4/SGCA/POPDC2/ANK2/BVES/AKAP6/SLC8A2/SYNC/DMD/RYR3/CDH2/CACNA1C/SGCD/SGCG/CACNG4/LAMA2/KCNJ8/PDE9A/CACNB2/TGFB3/ATP2B4/SSPN/CAV1/SLC27A6/FGF13/STAC/AHNAK2/SLC8A1/CACNA2D1/SGCE/POPDC3

GOBP_EPITHELIAL_CELL_PROLIFERATION	GOBP_EPITHELIAL_CELL_PROLIFERATION	2.73E-09	REG3G/FGL1/VIP/APOA1/AGTR1/MIR483/SFRP1/APOH/TACR1/NGFR/KRT4/THBS4/NKX2-5/FGF10/ALDH1A2/RETNLB/RGN/IGF1/PGR/FGF7/PTPRN/EPGN/AR/SFRP2/CCL26/NOG/HTR2B/LIMS2/GPC3/BMPER/PTN/FGF2/NR4A3/IGF2/CXCL12/ZEB1/COL8A1/PRKD1/VSTM4/TGM1/AKT3/THBS1/GLI1/SERPINF1/NKX6-1/CAV1/ATOH8/HGF/NODAL/FGFR1/CYP7B1/ANGPT1/TGFB2/KLF9/NRP2/IGFBP4/GPBAR1/IL6/ESR1/MCC/SHH/MIR342/SMO/OSR1/DAB2/CCL2/COL8A2/MIR27B/ISL1/FZD7/IGFBP5/CDKN1C/CCN3/WNT10B/SLURP1/SNAI2/CCL11/FGF1/MIR149/HAS2/CRNN/FAP/SCG2/TGFBR3/TEK/VASH2/MTSS1/MEF2C/EGR3/MIR222/TNFSF12/SPARC/LAMC1/PROK2/MIR10B/PTPRM/PTCH1/TWIST1/ITGB3/MMRN2[illegible]
GOBP_POSITIVE_REGULATION_OF_LIPASE_ACTIVITY	GOBP_POSITIVE_REGULATION_OF_LIPASE_ACTIVITY	3.22E-09	PLA2G1B/PNLIP/APOA1/AGTR1/APOH/ADCYAP1R1/HTR2A/PLA2G5/HTR2B/FGF2/GPIHBP1/AGT/EDNRA/LPAR1/FGFR1/CCN1/NMUR1/HPCA/ESR1/APOA4/AVPR1A/PDGFRA

GOBP_MESENCHYME_DEVELOPMENT	GOBP_MESENCHYME_DEVELOPMENT	3.80E-09	HAND1/SOX10/CER1/ACTG2/SFRP1/HAND2/NKX2-5/FGF10/ALDH1A2/TMEM100/DLL3/DACT3/ACTA2/IGF1/BNC2/SIX2/SFRP2/RBM24/NOG/FERMT2/HTR2B/CDH2/EPHA3/FLNA/ZFPM2/PHLDB2/FRZB/GREM1/ACTA1/WT1/TGFB1I1/SEMA3E/ACTC1/FOXF1/TGFB3/RADIL/EDNRA/MARK1/CRB2/HGF/NODAL/FGFR1/TGFB2/WWTR1/NRP2/HEYL/PKD2/IL6/SHH/MIR342/ZNF750/SMO/OSR1/DAB2/FN1/FOXF2/ISL1/FAM83D/MEOX1/TCF21/SNAI2/FOXC2/DCHS1/SEMA6A/SEMA6D/MIR149/TCF15/HAS2/PDPN/PHLDB1/TGFBR3/MEF2C/MIR222/LDLRAD4/ANXA6/TWIST1/SEMA6C/TBX5/SEMA3G/HOXA5/VASN/SEMA5B/SEMA3D/ROBO1/LEF1/COL1A1/ACVR1/PDGFRB/SEMA3C/EDNRB/NRP1

GOBP_REGULATION_OF_TRANSMEMBRANE_TRANSPORT	GOBP_REGULATION_OF_TRANSMEMBRANE_TRANSPORT	3.84E-09	PLA2G1B/KCNA1/CASQ2/PLP1/RELN/ATP1A2/SCN7A/DPP6/ACTN2/ADIPOQ/KCNB1/PLN/ADCYAP1R1/KCNMA1/CASQ1/KCNK3/JPH2/FHL1/KCNMB1/ANK2/SORBS1/GRIN2A/AKAP6/F2/KCNA5/ABCC9/RGN/IGF1/DMD/KCNH6/GRP/GPC3/RGS4/CLIP3/KCNIP1/FLNA/ATP1B2/CACNA1C/P2RX2/CACNG4/TRPC1/CACNA1H/NLGN1/SCN4B/NR4A3/FXYD6/KCNJ8/GIP/HRC/HSPA2/PRKD1/CACNB2/KCNE4/AGT/KCNH2/P2RX1/ATP2B4/THBS1/PID1/CAV1/GEM/EDNRA/SCN3A/FGF13/RGS2/STAC/HPCA/ABCB1/ITLN1/SLC8A1/PKD2/KLF15/BDKRB1/CACNA2D3/CACNA2D1/PRRT1/PRKCB/CACNA1A/CLIC4/KCNG1/OSR1/CCL2/ADRB2/FFAR4/KCNQ3/NLGN3/REM1/SLN/KCND1/MIR326/STOM/DLG4/KCNS1/PRNP/KCND2/RRAD/RYR2/CLIC2/THY1/[illegible]

GOBP_MULTICELLULAR_ORGANISMAL_SIGNALING	GOBP_MULTICELLULAR_ORGANISMAL_SIGNALING	3.93E-09	CARTPT/KCNA1/CASQ2/ATP1A2/SCN7A/PLN/NKX2-5/CTNNA3/ANK2/KCNA5/SLC8A2/ABCC9/NFASC/KCNH6/FLNA/ATP1B2/CACNA1C/CACNG4/TRPC1/JAM3/CACNA1H/SCN4B/HRC/CACNB2/KCNE4/AGT/KCNH2/CNTNAP1/CHRNB4/GJC1/P2RX1/ATP2B4/CAV1/SCN3A/TBX18/SLC8A1/CACNA2D1/CORIN/AVPR1A/ISL1/GJA1/S1PR1/CLDN19/KCND2/RYR2/NRCAM/FGF12/IRX3/TBX5

GOCC_ORGANELLAR_RIBOSOME	GOCC_ORGANELLAR_RIBOSOME	4.91E-09	MRPS23/DAP3/MRPL24/MRPS15/MRPL57/MRPL48/MRPL9/MRPL40/NSUN3/MRPL27/MRPS10/MRPL45/MRPS7/MRPL36/MRPL32/MRPL20/MRPS33/MRPL17/MRPS30/MRPL14/MTG2/MRPL30/MRPL3/MRPL18/MRPL22/MRPL21/MRPL52/MRPL13/MRPS11/MRPL19/MRPL35/MRPS18B/MRPS9/MRPL42/MRPL43/MRPL34/NSUN4/MRPL28/MRPL47/MRPS18A/MRPS22/MRPL39/PTCD3/MRPL2/MRPL58/MRPL49/MRPS26/MRPS5/CHCHD1/MTG1/MRPL1/MRPL15/MRPL50/MRPL46/GADD45GIP1/MRPL54/MRPL11/MRPL16/MPV17L2/MRPS18C/MRPS12/MRPS25/MRPL41/AURKAIP1/MRPS28/MRPS27/MRPL12/MRPS16/MRPL23/MRPL37/MRPL44/MRPS34/MRPS35/MRPS2/MRPL4

GOBP_OSSIFICATION	GOBP_OSSIFICATION	5.59E-09	AHSG/PENK/CER1/COL2A1/BMP3/SFRP1/HAND2/MYOC/TPH1/TACR1/CHRDL1/OMD/MGP/CYP24A1/IGF1/CHRDL2/SIX2/SFRP2/NOG/FERMT2/GPC3/TWIST2/ROR2/DDR2/PTN/FGF2/TNC/TNN/ZBTB16/GREM1/PTH1R/ASPN/IGF2/GPM6B/LRRC17/GLI3/GDF10/CHRD/MN1/PRKD1/RSPO2/TGFB3/GLI1/IGSF10/HGF/CCN1/TGFB2/WWTR1/SLC8A1/MMP2/TMEM119/COMP/IL6/CDH11/HOXA2/SHH/SMO/OSR1/DHH/PBX1/CLEC3B/MMP16/DKK1/ADRB2/NELL1/FFAR4/IGFBP5/CTSK/WNT10B/ID4/SLC24A3/SNAI2/FOXC2/DCHS1/CCN4/CCN2/PDLIM7/PTHLH/S1PR1/LOX/ILK/BMPR1B/VCAN/STC1/KL/MEF2C/LTBP3/COL1A2/CLEC11A/NPR2/MRC2/GABBR1/PTCH1/TWIST1/GPLD1/SCUBE3/VEGFC/CTHRC1/ITGA11/IL6ST/LEF1/COL1A1/ACV[illegible]

GOBP_POSITIVE_REGULATION_OF_CHROMOSOME_ORGANIZATION	GOBP_POSITIVE_REGULATION_OF_CHROMOSOME_ORGANIZATION	6.25E-09	MAD2L1BP/CDK2/CCT6A/TPR/PNKP/GTF2H2/SMC5/MAP3K4/SETDB1/FBXO4/PPP1R10/ATR/NABP2/PARN/TERF2/MACROH2A1/ACTL6A/KAT5/RTEL1/MAP2K7/XRCC5/NAF1/UCHL5/ACD/INO80D/SFPQ/RUVBL1/INO80E/INO80/DKC1/SLF1/SLX4/MAPK3/PML/RUVBL2/TCP1/NEK2/HNRNPD/CCT8/MCRS1/CCT2/HNRNPA2B1/CCT3/ATF7IP2/GNL3/NFRKB/RAD21/SMC4/CCT4/NCAPD2/CCT7/TRIM28/DDX11/CCT5/AURKB/SIRT6/NBN/HNRNPA1/PRKCQ/TNKS/SMC2/NCAPH/DNMT1/FEN1/MAPK15/NCAPG/CDT1

GOBP_NEGATIVE_REGULATION_OF_CHROMOSOME_ORGANIZATION	GOBP_NEGATIVE_REGULATION_OF_CHROMOSOME_ORGANIZATION	6.83E-09	MAD2L1BP/TENT4B/TPR/MAD2L2/WAPL/H3-3B/SPDL1/TERF2/ANAPC15/H3-3A/RTEL1/XRN1/ACD/TRIP13/STN1/XRCC1/SRC/NAT10/HNRNPU/HNRNPC/SLX4/NAA10/NUF2/EXOSC10/PIF1/PARP1/ZW10/PINX1/RAD21/TTK/KNTC1/XRCC3/FBXO5/ZWILCH/AURKB/NDC80/BUB1/BUB3/NBN/HNRNPA1/GEN1/TNKS/SPC25/PTTG1/CENPF/INCENP/SPC24/BUB1B/CDC20/CCNB1/ESPL1/HASPIN/MAD2L1/PLK1/BIRC5/CDCA8/TOP2A/ZWINT/MCM2/CDT1

GOBP_REGULATION_OF_HEART_CONTRACTION	GOBP_REGULATION_OF_HEART_CONTRACTION	7.24E-09	SPX/CASQ2/CHRM2/ATP1A2/DES/HSPB7/PLN/NKX2-5/CTNNA3/POPDC2/ANK2/BVES/KCNA5/SLC8A2/ABCC9/DMD/MYL3/KCNH6/RGS4/FLNA/ATP1B2/CACNA1C/ADRA1D/TRPC1/CACNA1H/SCN4B/HRC/CACNB2/KCNE4/AGT/TPM1/KCNH2/CHGA/ADRA1B/GJC1/ATP2B4/DMPK/CAV1/EDNRA/FGF13/RGS2/TBX18/TGFB2/SLC8A1/CACNA2D1/CORIN/HOPX/AVPR1A/CELF2/ISL1/GLP1R/THRB/GJA1/CCN2/RNLS/RYR2/STC1/CLIC2/PDE5A/IRX3/TBX5/ADORA3

GOCC_REPLICATION_FORK	GOCC_REPLICATION_FORK	7.75E-09	RFC3/XRCC2/RFC1/SMARCAD1/ZMIZ2/POLA1/PRIM2/RPA1/POLH/RFC4/PIF1/EME1/MUS81/PARP1/DONSON/WRN/RAD18/HMCES/RFC5/XRCC3/POLA2/MMS22L/MCM10/CARM1/RFC2/TEX264/NBN/POLD3/TIPIN/PRIM1/WDHD1/EME2/POLD2/TIMELESS/CHEK1/PCNA/BLM/TP53/MCM3/DNMT1/H2AX/TONSL/POLD1/UHRF1

GOBP_REGULATION_OF_HORMONE_LEVELS	GOBP_REGULATION_OF_HORMONE_LEVELS	8.72E-09	PNLIP/AFP/GCG/UCN3/CARTPT/CELA2A/CEL/GHRH/VIP/BCHE/AGTR1/TACR2/SFRP1/ADIPOQ/NEUROD1/NNAT/KCNB1/SCG5/FGA/TACR1/CTSG/PCSK1/ADCYAP1/CMA1/ADH1B/ADCY5/ALDH1A2/SULT2A1/CPE/SCT/KCNA5/PTPRN/FGG/GHR/GRP/TTR/CYP1B1/CCKAR/UGT2B17/CNR1/PCSK2/CACNA1H/DIO1/GIP/RDH8/AGT/CHGA/CYP11A1/ADH1A/ADH7/RFX6/NKX6-1/SELENOM/CPA3/UGT1A7/RBP4/IL6/ESR1/PRKN/CORIN/NR1H4/SHH/PDGFRA/DAB2/CGA/SNAP25/HSD17B6/TPO/GDF9/ISL1/FFAR4/GLP1R/DIO2/CCN3/CTSK/RETN/CRABP2/GJA1/PCSK1N/PDE8B/GPR27/DKK3/IL11/ENPEP/CHST9/KLF7/CHST10/FGB/LRAT/NLGN2/INHBA/INHA/ALDH1A3/BLK/GABBR1/NR3C1/SLC16A2/GPLD1/CYP3A4/MYRIP/CPQ [illegible]

GOCC_SPLICEOSOMAL_COMPLEX	GOCC_SPLICEOSOMAL_COMPLEX	9.76E-09	GEMIN2/HNRNPH3/CWC22/RBM3/SNRNP35/PRPF39/RBM8A/PPIL3/SNU13/SNRPD2/IK/GCFC2/PRKRIP1/PRPF18/SNW1/MAGOHB/RBM17/SNRPD3/ISY1/LSM5/DHX8/GPKOW/CDC5L/WDR83/DDX46/DNAJC17/LSM6/PDCD7/USP39/RBM22/BCAS2/SNRPG/HNRNPA1L2/PRPF38B/DHX32/RALY/ESS2/CRNKL1/LUC7L2/HNRNPA3/SNRPC/PRPF6/SMNDC1/MAGOH/HTATSF1/TRA2B/SF3B1/TRA2A/CCDC12/PRPF38A/DHX16/PPIL2/API5/SF3A1/PRPF31/PPIL1/LSM3/ADAR/RBMX/SNRPF/TFIP11/MFAP1/SUGP1/WBP11/LSM2/SNRPA1/PPIH/HNRNPH1/HSPA8/SF1/MTREX/PPP1R8/SNRNP48/PHF5A/SMU1/CDC40/SART1/RBM28/SF3A3/SNRNP70/LSM7/ZCCHC8/DHX38/SNRPB/XAB2/RBMXL1/EFTUD2/PRPF19/TXNL4B/DDX41/PRPF8/LGALS3/SRRM [illegible]

GOCC_LARGE_RIBOSOMAL_SUBUNIT	GOCC_LARGE_RIBOSOMAL_SUBUNIT	9.95E-09	RPL27A/RPLP1/RPL13/RPL11/MRPL24/MRPL57/RPL34/RPL38/MRPL48/MRPL9/MRPL40/RPL24/NSUN3/MRPL27/RPLP2/MRPL45/UBA52/RPL22/RPL23A/MRPL36/RPL15/MRPL32/RPL10A/MRPL20/RPL32/MRPL17/MRPS30/RPL26/RPL6/MRPL14/RPL37A/RPL13A/RPL14/MRPL30/MRPL3/MRPL18/RPL4/MRPL22/MRPL21/RPL30/MRPL52/MRPL13/MRPL19/MRPL35/RPL12/RPL26L1/MRPL42/RPL18/MRPL43/MRPL34/NSUN4/FXR2/RPL7A/MRPL28/MRPL47/MRPS18A/RPL9/MRPL39/RPL35/RPL7/RPL3/MRPL2/MRPL58/MRPL49/RPL7L1/RPLP0/MRPL1/MRPL15/MRPL50/MRPL46/RPLP0P6/GADD45GIP1/MRPL54/MRPL11/RPL29/RPL19/MRPL16/MPV17L2/RPL36/MRPL41/RPL41/MRPL12/MRPL23/MRPL37/MRPL44/RPL18A/NPM1/RPL8/R[illegible]
GOCC_VACUOLAR_LUMEN	GOCC_VACUOLAR_LUMEN	1.02E-08	ELANE/MPO/PRTN3/AZU1/NSG2/OGN/RNASE3/KERA/BPI/CTSG/OMD/PRELP/APOB/RNASE2/GPC3/TTR/DCN/PRSS2/CUBN/CTSV/CSF3/LUM/CSPG4/SERPINA3/ORM2/CTSF/GPC6/SDC2/CTSK/RETN

GOCC_SITE_OF_DNA_DAMAGE	GOCC_SITE_OF_DNA_DAMAGE	1.05E-08	RNF168/RNF169/ELOA/SHLD2/SHLD3/TP53BP1/CBX3/WAS/MAD2L2/RPA3/PNKP/RIF1/RPA2/SMC5/SPINDOC/DDB1/NABP2/CYREN/CNTD1/RNF138/PARP9/ZBTB7A/NHEJ1/XRCC5/SMARCAD1/SMCHD1/XPC/HROB/PRPF19/POLL/RPA1/POLH/UFL1/XRCC1/ADPRS/LMNA/SLF1/ARPC4/PAXX/PARP1/UBQLN4/RAD18/HELB/OARD1/TRAIP/VCP/SIRT7/MMS22L/RFWD3/SIRT6/RBBP8/NBN/NKX3-1/WRAP53/CGAS/TIMELESS/DDB2/PARP3/TP53/RAD51/AUNIP/H2AX/TONSL/ESCO2/WDR76
GOBP_REGULATION_OF_DNA_TEMPLATED_DNA_REPLICATION	GOBP_REGULATION_OF_DNA_TEMPLATED_DNA_REPLICATION	1.13E-08	DSCC1/DONSON/WRNIP1/BRCA2/DBF4/RFC5/GINS1/FBXO5/RFC2/NBN/GINS2/TIPIN/STOML2/MCM7/MCM6/TIMELESS/PCNA/NUGGC/CHTF18/GINS3/BLM/TICRR/MCM3/ENDOG/MCM5/CDC7/MCIDAS/GINS4/MCM4/E2F8/MCM2/E2F7/CDT1

GOMF_ATP_DEPENDENT_ACTIVITY_ACTING_ON_DNA	GOMF_ATP_DEPENDENT_ACTIVITY_ACTING_ON_DNA	1.19E-08	MSH3/RECQL5/BTAF1/ERCC2/RAD54L2/TOP2B/MCM8/XRCC6/EP400/SMC3/CDK7/PMS2/MYO18A/PMS1/RFC3/RTEL1/XRCC5/XRCC2/RFC1/SMARCAD1/ATAD5/MSH6/DHX9/RUVBL1/INO80/FANCM/RUVBL2/RFC4/PIF1/RAD54B/G3BP1/UPF1/SRCAP/SUPV3L1/MCM9/RBBP4/DSCC1/WRNIP1/WRN/ASCC3/HELB/RFC5/DDX11/XRCC3/DDX1/RFC2/DDX12P/MSH2/ERCC6L/MCM7/DMC1/MCM6/SMARCA4/TWNK/CHTF18/BLM/POLQ/RAD51/TTF2/MCM3/DNA2/DQX1/MCM5/HELLS/RECQL4/TOP2A/RAD54L/MCM4/BRIP1/MCM2

GOBP_PROTEIN_LOCALIZATION_TO_CHROMOSOME	GOBP_PROTEIN_LOCALIZATION_TO_CHROMOSOME	1.41E-08	MCM8/H4C13/CCT6A/CHAMP1/SETD2/RPA2/CTCF/SMC5/WAPL/ATR/NABP2/SPDL1/TERF2/MACROH2A1/VCPIP1/RB1/SPIDR/XRCC5/ACD/H4C9/PIH1D1/H2BC11/RPA1/CHMP7/H4C4/H4C3/DKC1/SLF1/RUVBL2/LRWD1/TCP1/CCT8/CCT2/H4C2/MCM9/CCT3/H4C1/MTBP/ZW10/GNL3/BRCA2/PINX1/RAD21/CCT4/TTK/KNTC1/CCT7/CCT5/VRK1/VCP/ZWILCH/AURKB/NDC80/BUB3/SIRT6/CENPA/MSH2/WRAP53/EZH2/TNKS/CDK1/BUB1B/PARP3/RCC2/TERT/HASPIN/CDCA5/PLK1/KNL1/ESCO2/CDT1/H1-5

GOBP_COGNITION	GOBP_COGNITION	1.44E-08	SYT4/VIP/BCHE/RELN/ATP1A2/TACR2/TACR1/SLC2A4/NRXN3/NRXN2/GRPR/GRIN2A/SLC8A2/HTR2A/PTCHD1/NPTX2/NOG/KCNK2/ADGRB3/NGF/PTN/CNR1/MAP1A/NLGN4X/ADCY1/GIP/LRRN4/AGT/ITGA8/NLGN4Y/CHL1/MAPT/NFATC4/SERPINF1/SOBP/VLDLR/TUSC3/PRKAR2B/CX3CR1/FGF13/C1QL1/PDE1B/ITGA5/PRRT1/PRKN/FAM107A/SNAP25/DKK1/GPR155/SYT11/NTF3/GLP1R/OXTR/NLGN3/BRSK1/PDE8B/CCL11/DLG4/PRNP/PTPRZ1/CHST10/CLDN5/MEF2C/S100B/JPH3

GOBP_REGULATION_OF_TELOMERE_MAINTENANCE	GOBP_REGULATION_OF_TELOMERE_MAINTENANCE	1.62E-08	RAD50/MAPKAPK5/ACTR8/SMG6/CTNNB1/TENT4B/CCT6A/PNKP/SMC5/MAP3K4/FBXO4/PPP1R10/ATR/NABP2/PARN/TERF2/SMG1/ACTL6A/RTEL1/MAP2K7/SMG5/XRCC5/NAF1/UCHL5/XRN1/ACD/INO80D/STN1/RUVBL1/XRCC1/INO80E/USP7/INO80/MYC/SRC/NAT10/DKC1/HNRNPU/SLF1/HNRNPC/SLX4/MAPK3/PML/RUVBL2/TCP1/EXOSC10/NEK2/HNRNPD/PIF1/CCT8/MCRS1/CCT2/UPF1/HNRNPA2B1/PARP1/CCT3/GNL3/NFRKB/PINX1/CCT4/CCT7/CCT5/AURKB/SIRT6/NBN/HNRNPA1/PRKCQ/TNKS/MAPK15

GOBP_HEART_PROCESS	GOBP_HEART_PROCESS	1.74E-08	SPX/CASQ2/CHRM2/ATP1A2/DES/HSPB7/PLN/NKX2-5/CTNNA3/POPDC2/ANK2/BVES/KCNA5/SLC8A2/ABCC9/DMD/MYL3/KCNH6/RGS4/FLNA/ATP1B2/CACNA1C/ADRA1D/SGCD/SGCG/TRPC1/CACNA1H/SCN4B/KCNJ8/HRC/CACNB2/KCNE4/AGT/TPM1/KCNH2/ACTC1/CHGA/ADRA1B/GJC1/ATP2B4/DMPK/CAV1/EDNRA/FGF13/RGS2/TBX18/TGFB2/WWTR1/SLC8A1/CACNA2D1/CORIN/HOPX/AVPR1A/CELF2/ISL1/GLP1R/THRB/GJA1/CCN2/RNLS/RYR2/STC1/NOX4/CLIC2/PDE5A/FGF12/IRX3/TBX5/ADORA3/CXCR4/GSN/OXT/EDNRB/SCN3B/RAMP3/EDN2/MYL4/SCN1B/SLC4A3/GSTM2/SNTA1

GOBP_CYCLIC_NUCLEOTIDE_MEDIATED_SIGNALING	GOBP_CYCLIC_NUCLEOTIDE_MEDIATED_SIGNALING	1.86E-08	NPPB/ADCYAP1R1/ADCYAP1/AKAP6/SCT/HTR2B/CAP2/ADCY2/IRAG1/PDE7B/ADCY1/GUCY1A1/GIP/PDE9A/PDE3A/CD36/PRKG1/THBS1/GUCY1B1/RASD2/LPAR1/PDE2A/RUNDC3A/NPR1/SCTR/FFAR4/GLP1R/GUCY1A2/GNAI1/NPR2/EDNRB/PDE3B/NPPC/APLNR

GOBP_DNA_CONFORMATION_CHANGE	GOBP_DNA_CONFORMATION_CHANGE	2.08E-08	RECQL5/ERCC2/RAD54L2/TOP2B/MCM8/XRCC6/GTF2H2/TOP1/ASCC1/RFC3/ASCC2/RTEL1/XRCC5/SMARCAD1/TOP3A/RPA1/DHX9/HMGB3/RUVBL1/FANCM/RUVBL2/RFC4/PIF1/RAD54B/G3BP1/UPF1/HNRNPA2B1/SUPV3L1/MCM9/DSCC1/WRNIP1/WRN/ASCC3/HELB/RFC5/GINS1/HMGB2/DDX11/DDX1/TOP1MT/RFC2/NBN/DDX12P/GINS2/ERCC6L/MCM7/MCM6/TWNK/CHTF18/GINS3/HMGA1/BLM/POLQ/RAD51/MCM3/DNA2/CDC45/DQX1/MCM5/RECQL4/TOP2A/GINS4/RAD54L/MCM4/BRIP1/MCM2

GOBP_TELOMERE_MAINTENANCE_VIA_TELOMERE_LENGTHENING	GOBP_TELOMERE_MAINTENANCE_VIA_TELOMERE_LENGTHENING	2.58E-08	RAD50/MAPKAPK5/SMG6/HSP90AB1/NOP10/CTNNB1/TENT4B/CCT6A/PNKP/MAP3K4/FBXO4/ATR/PARN/TERF2/HSP90AA1/RTEL1/MAP2K7/SMG5/XRCC5/NAF1/RFC1/XRN1/ACD/RPA1/STN1/SRC/NAT10/DKC1/HNRNPU/HNRNPC/SLX4/MAPK3/TCP1/EXOSC10/PTGES3/NEK2/PIF1/CCT8/CCT2/HNRNPA2B1/PARP1/CCT3/PINX1/ZSCAN4/CCT4/CCT7/CCT5/AURKB/GAR1/TELO2/DCLRE1B/HNRNPA1/WRAP53/NHP2/PRKCQ/TNKS/RAD51/TERT/MAPK15

GOBP_REGULATION_OF_ION_TRANSMEMBRANE_TRANSPORT	GOBP_REGULATION_OF_ION_TRANSMEMBRANE_TRANSPORT	2.65E-08	PLA2G1B/KCNA1/CASQ2/PLP1/RELN/ATP1A2/SCN7A/DPP6/ACTN2/KCNB1/PLN/ADCYAP1R1/KCNMA1/CASQ1/KCNK3/JPH2/FHL1/KCNMB1/ANK2/GRIN2A/AKAP6/F2/KCNA5/ABCC9/RGN/DMD/KCNH6/GRP/RGS4/KCNIP1/FLNA/ATP1B2/CACNA1C/P2RX2/CACNG4/TRPC1/CACNA1H/NLGN1/SCN4B/FXYD6/KCNJ8/HRC/HSPA2/PRKD1/CACNB2/KCNE4/AGT/KCNH2/P2RX1/ATP2B4/CAV1/GEM/EDNRA/SCN3A/FGF13/RGS2/STAC/HPCA/ABCB1/SLC8A1/PKD2/BDKRB1/CACNA2D3/CACNA2D1/PRRT1/CACNA1A/CLIC4/KCNG1/OSR1/CCL2/ADRB2/KCNQ3/NLGN3/REM1/SLN/KCND1/STOM/DLG4/KCNS1/PRNP/KCND2/RRAD/RYR2/CLIC2/THY1/MEF2C/NLGN2/JPH3/FGF12/TWIST1/ITGB3/RASGRF2
GOBP_STRIATED_MUSCLE_CELL_DEVELOPMENT	GOBP_STRIATED_MUSCLE_CELL_DEVELOPMENT	3.15E-08	MYH11/LDB3/ACTN2/PGM5/FLNC/LMOD1/CASQ1/NKX2-5/MYL9/ANKRD1/TMOD1/KLHL41/CFL2/CSRP1/ACTA1/TPM1/ACTC1/LMOD3/FHOD3/PDGFRA/CSRP2

GOBP_NUCLEAR_EXPORT	GOBP_NUCLEAR_EXPORT	3.63E-08	PCID2/NXF1/RBM8A/PHAX/IWS1/YTHDC1/LSG1/CSE1L/TSC1/RAPGEF3/MAGOHB/EMD/NUP155/AGFG1/MALT1/NMD3/NUP107/NXT1/AKAP8L/RBM22/DDX19B/DESI1/NUP133/DUSP16/RIOK2/RANBP2/EIF6/POLDIP3/CTDSPL2/LTV1/NUP153/ALKBH5/AKAP13/SMG6/UBE2I/XPO5/NUP214/CPSF6/NCBP3/MAGOH/PABPN1/TPR/SMURF1/SETD2/TCF7L2/XPO4/SMG7/NUTF2/RBM15B/RANBP3/THOC6/PARK7/SMG1/XPO1/NUP160/SMG5/SRSF3/CHTOP/NUP62/ABCE1/NUP62CL/POLR2D/IFI27/NCBP1/KHDRBS1/DHX9/TXN/MDN1/FRAT1/POM121C/NUP85/NSUN2/EIF4A3/CALR/IL1B/NUP93/XPO6/BARD1/UPF1/HNRNPA2B1/CASC3/YWHAE/ANP32B/RITA1/MCM3AP/RAN/POM121/XPO7/NOP9/CCHCR1/NUP88/RPS15/HSPA9/CDK5/RANGAP1/[illegible]

GOCC_PLATELET_ALPHA_GRANULE	GOCC_PLATELET_ALPHA_GRANULE	3.78E-08	ALB/AHSG/ACTN2/FGA/IGF1/FGG/IGF2/F13A1/SERPINF2/MMRN1/CD36/TGFB3/LEFTY2/THBS1/SELP/HGF/SERPINA3/TGFB2/SERPINE2/SERPINA5/SERPINE1/ORM2/PROS1/FN1/F8/A2M/ISLR/PCDH7/FGB/ACTN1/SPARC/SERPING1/CLU/THBS2/ITGB3/VEGFC/TIMP1/PECAM1/SRGN/TREML1/CD109

GOBP_CONNECTIVE_TISSUE_DEVELOPMENT	GOBP_CONNECTIVE_TISSUE_DEVELOPMENT	4.16E-08	HAND1/OGN/CER1/COL2A1/BMP3/HAND2/MGP/ACTA2/CHRDL2/GHR/SIX2/SFRP2/GDF6/NOG/EFEMP1/NKX3-2/EBF2/COL11A2/FGF2/ZBTB16/FRZB/GREM1/PTH1R/WT1/GLI3/ZEB1/WNT9A/RSPO2/MATN3/SELENOM/SOX5/EVC/CCN1/CYTL1/COMP/COL3A1/NR1H4/OSR1/PRRX1/SPART/GDF7/CCN3/CTSK/WNT2B/WNT10B/EPYC/ID4/PDGFD/SNAI2/FOXC2/CCN4/CCN2/PTHLH/LOX/MAF/TGFBI/BMPR1B/KLF7/STC1/MEF2C/LTBP3/ANXA6/BGN/GPLD1/ITGB3/HOXA5/TRPS1/SH3PXD2B/SULF1/CD34/COL1A1/PDGFRB/COL5A1/TIMP1

GOMF_G_PROTEIN_COUPLED_RECEPTOR_ACTIVITY	GOMF_G_PROTEIN_COUPLED_RECEPTOR_ACTIVITY	4.45E-08	CHRM2/AGTR1/TACR2/GRIK3/MLNR/TACR1/ADCYAP1R1/NPFFR2/MRGPRF/GLP2R/GRPR/NPR3/HTR2A/HTR2B/ADGRB3/PTGFR/ADGRD1/NPY/CCKAR/ADRA1D/PTGER3/ACKR1/GABBR2/CNR1/PTH1R/PDGFRL/VIPR2/ELOVL4/LPAR4/GPR17/ADRA1B/LPAR3/GPR15/EDNRA/HCAR1/CX3CR1/FZD10/CNR2/CHRM4/GPR142/LPAR1/S1PR3/NMUR1/SORCS2/NPR1/BDKRB1/P2RY14/GPBAR1/AVPR1A/CYSLTR1/SMO/SCTR/GPR162/ADRB2/GPR135/CCKBR/GPR183/FFAR4/FZD7/GLP1R/OXTR/SSTR2/ADGRA2/GPR27/PTGIR/S1PR1/GPR161/AVPR2/PTGER1/GPR34/RAMP2/CXCR2/FPR1/CXCR1/GPR173/GABBR1/ADORA3/ADGRB2/ADGRB1/VN1R1/P2RY1/CXCR4/GPER1/ADGRG2/CCR7/ACKR3/PDGFRB/PTGDR/S1PR5/FFAR2/ADGRL3/NPY6R/UTS2R/[illegible]

GOMF_SIGNALING_RECEPTOR_REGULATOR_ACTIVITY	GOMF_SIGNALING_RECEPTOR_REGULATOR_ACTIVITY	4.48E-08	GCG/UCN3/SPX/CARTPT/CCL16/GHRH/VIP/APOA1/NPPB/TAFA4/PENK/OGN/CER1/BMP3/ADIPOQ/TMEM35A/THBS4/CTSG/FGF10/EPHA7/ADCYAP1/F2/SCT/FLRT2/RETNLB/IGF1/FGF7/EPGN/SFRP2/GDF6/CCL26/GRP/EFEMP1/FNDC5/GREM2/TTR/NPY/VSTM1/IL24/NGF/PTN/FGF2/C1QTNF4/GREM1/CXCL6/ALKAL1/IGF2/CXCL12/THPO/GDF10/SEMA3E/IL33/GIP/AGT/WNT9A/CCL21/TGFB3/LEFTY2/TSLP/ADH7/CCL19/CSF3/NDP/C10orf99/NRG2/FGF13/HGF/NODAL/ENDOU/TGFB2/FBN1/IL17B/MLN/TNFRSF11B/GUCA2A/IL6/PRRT1/HDGFL3/CTF1/SHH/CGA/CCL2/DKK1/GDF9/CRLF1/NTF3/GDF7/CCN3/RETN/WNT2B/WNT10B/SLURP1/PDGFD/IGSF1/DKKL1/CCN2/SEMA6A/CCL11/PTHLH/FGF1/SEMA6D/DKK3/IL11/INSL6/D[illegible]

GOCC_SYNAPTIC_MEMBRANE	GOCC_SYNAPTIC_MEMBRANE	4.73E-08	KCNA1/CHRM2/GRIK3/CHRNA3/KCNB1/KCNMA1/GRIK1/EPHA7/GPM6A/ANK2/CADM3/GRIN2A/CRYAB/HTR2A/DMD/GRIK5/GLRB/CDH2/CNTN1/PLPPR4/CACNA1C/ADAM23/GABBR2/CACNG4/CNR1/NLGN4X/NLGN1/PTPRD/FXYD6/ADCY1/CNTNAP1/ITGA8/NLGN4Y/DLG2/RGS7BP/CHRNB4/P2RX1/ATP2B4/SSPN/CHRM4/LIN7A/PDE2A/SORCS2/PDLIM4/MAGEE1/NRP2/CLSTN2/SLC8A1/SHC4/SYNE1/PRRT1/SNAP25/SYT11/EFNB3/NLGN3/KCND1/TMEM240/LRRC4/SYDE1/DLG4/LRRC4C/SRPX2/KCND2/SYNDIG1/IQSEC3/NRCAM/NLGN2/APBA1/GABBR1/GABRR1/SYT1/ITGB3/SYDE2/ADORA3/CHRNB2/PCDH17/SYP/P2RY1/GPER1/MUSK/ARC/GABRE/SNCAIP/NRP1/LRRC4B/IGSF21/GRID1/SNTA1/STX1B/DNM1/TAMALIN/CHRM3/NTNG2/GRI[illegible]

GOBP_SKELETAL_SYSTEM_DEVELOPMENT	GOBP_SKELETAL_SYSTEM_DEVELOPMENT	4.73E-08	HAND1/AHSG/OGN/CER1/COL2A1/BMP3/SFRP1/HAND2/MYOC/NPR3/MGP/PRELP/DLL3/PAPPA2/RGN/IGF1/CHRDL2/GHR/BNC2/SIX2/SFRP2/GDF6/NOG/EFEMP1/FLNA/NKX3-2/DDR2/COL11A2/FGF2/TNN/ZBTB16/FRZB/GREM1/PTH1R/SFRP4/HOXD10/FREM1/LRRC17/GLI3/THPO/GDF10/ZEB1/CHRD/LEPR/WNT9A/RSPO2/RAB23/TGFB3/HOXA4/TLL1/HHIP/MATN3/EDNRA/HOXD1/SOX5/EVC/NODAL/FGFR1/CCN1/TGFB2/MEIS1/FBN1/RBP4/CYTL1/TNFRSF11B/MMP2/TMEM119/COMP/CDH11/COL3A1/HOXA2/SHH/PDGFRA/OSR1/PBX1/MMP16/PRRX1/CCN3/CTSK/WNT2B/WNT10B/EPYC/GJA1/SNAI2/FOXC2/DCHS1/CCN4/CCN2/PTHLH/HOXD4/TCF15/LOX/MAF/HAS2/TGFBI/BMPR1B/TBX15/VCAN/STC1/MEF2C/LTBP3/COL1A2/NPR[illegible]

GOBP_REGULATION_OF_DNA_REPAIR	GOBP_REGULATION_OF_DNA_REPAIR	4.86E-08	ING3/ARID2/ARID1A/SLF2/MAGEF1/ACTR2/ERCC6/HMGB1/TAF9/MGMT/OGG1/SIRT1/TAF12/YEATS4/UBR5/ACTR8/TAF2/TADA3/TRRAP/TIGAR/PPP4R3B/TAF5L/RECQL5/UBE2N/USP22/TADA1/RNF168/UIMC1/RNF169/SHLD2/SHLD3/TP53BP1/WAS/MAD2L2/SMARCD1/EP400/PNKP/TADA2B/SETD2/RIF1/RPA2/BRD7/BRD8/ATR/MRGBP/CCDC117/CYREN/TERF2/RPS3/TFIP11/DEK/SMARCC1/PIAS4/ACTL6A/PARP9/KAT5/NUDT16L1/PARG/SPIDR/RTEL1/SMARCC2/FIGNL1/OTUB1/SMCHD1/UCHL5/HDAC10/HDGFL2/INO80D/MMS19/TAF6L/ATXN7L3/CUL4A/SMARCB1/FUS/POLH/BRCC3/DHX9/BRCA1/RUVBL1/XRCC1/DTX3L/INO80E/INO80/HSF1/SLF1/PML/RUVBL2/KDM1A/MCRS1/BARD1/FANCB/PARP1/EGFR/UBQLN4/SPIRE2/W[illegible]

GOBP_ACTOMYOSIN_STRUCTURE_ORGANIZATION	GOBP_ACTOMYOSIN_STRUCTURE_ORGANIZATION	5.50E-08	APOA1/CNN1/MYH11/SFRP1/LDB3/ACTN2/PGM5/MYOC/FLNC/LMOD1/TACR1/CASQ1/NKX2-5/MYL9/ANKRD1/SORBS1/TMOD1/KLHL41/CFL2/FERMT2/CSRP1/PHLDB2/ACTA1/ELN/SERPINF2/TPM1/ACTC1/TGFB3/PPM1E/CGNL1/LPAR1/LMOD3/FRMD6/MYH10/FHOD3/PDGFRA/CSRP2/DLC1/CCN2/EPB41L3/ARHGAP28/MIR149/S1PR1

GOBP_SYNAPSE_ORGANIZATION	GOBP_SYNAPSE_ORGANIZATION	5.76E-08	SEZ6L/RELN/FRRS1L/INA/CHRDL1/EPHA7/GPM6A/NRXN2/NEGR1/SEZ6/SPARCL1/SLC8A2/FLRT2/GAP43/NFASC/DNER/ADGRB3/GLRB/CDH2/PCDHB5/PDZRN3/ZDHHC15/MAP1B/FLNA/PAK3/P2RX2/TNC/LGI2/KY/NLGN4X/NLGN1/PTPRD/NPTX1/SEMA3E/NTN1/ADD2/WASF3/CACNB2/CNTNAP1/SDK2/NLGN4Y/TUBA1A/MAPT/PCDHB4/NFATC4/COL4A5/DMPK/PCDHB6/CX3CR1/FGF13/C1QL1/L1CAM/LRRN1/NRP2/CLSTN2/PRRT1/PCDHGC3/CTTNBP2/PALM/DKK1/GPC6/KIF1A/SLITRK6/BSN/PCDHB2/CAMKV/OXTR/NLGN3/LRRK2/SNCG/LRRC4/SYNPO/CTNND2/TMEM108/DLG4/NEURL1/NEFL/PRNP/LRRC4C/SRPX2/PCDHB3/SYNDIG1/COLQ/ACTN1/MEF2C/NRCAM/WASF1/NLGN2/FCGR2B/NFIA/THBS2/CHRNB2/ADGRB1/PCDH17/MUSK/AR[illegible]

GOBP_RNA_LOCALIZATION	GOBP_RNA_LOCALIZATION	6.19E-08	NUP155/STAU2/AGFG1/NUP107/SIDT2/PNPT1/NXT1/AKAP8L/DDX19B/HNRNPA1L2/NUP133/FMR1/RANBP2/SENP2/POLDIP3/G3BP2/NUP153/ALKBH5/SMG6/CETN3/XPO5/NOP10/NUP98/NUP214/ZNHIT3/CPSF6/NCBP3/PRPF6/MAGOH/CCT6A/PABPN1/TPR/SHQ1/SRSF7/SETD2/AAAS/SMG7/NUP54/NUTF2/PRPF31/ATR/PARN/RBM15B/THOC6/AHCTF1/IGF2BP2/MRPL18/SMG1/XPO1/NUP160/SEH1L/KPNB1/SMG5/SRSF3/NUP35/CHTOP/NAF1/NUP62/NUP62CL/POLR2D/PIH1D1/NCBP1/KHDRBS1/DHX9/NOP58/RUVBL1/POM121C/NUP85/NSUN2/NUP43/EIF4A3/SRSF1/HSF1/DKC1/HNRNPU/RUVBL2/FBL/TCP1/NUP58/EXOSC10/NUP93/CCT8/CCT2/UPF1/HNRNPA2B1/CASC3/YBX1/MCM3AP/CCT3/RAN/POM121/CKAP5/ZC3H3/NUP205/[illegible]

GOBP_TRANSMEMBRANE_RECEPTOR_PROTEIN_SERINE_THREONINE_KINASE_SIGNALING_PATHWAY	GOBP_TRANSMEMBRANE_RECEPTOR_PROTEIN_SERINE_THREONINE_KINASE_SIGNALING_PATHWAY	6.27E-08	AFP/APOA1/CER1/BMP3/SFRP1/DSG4/CHRDL1/MYOCD/RBPMS2/CIDEA/FGF10/RGMA/AMHR2/TMEM100/CHRDL2/CILP/SFRP2/GDF6/NOG/FERMT2/GREM2/GPC3/BMPER/TF/GREM1/ASPN/SFRP4/TGFB1I1/GDF10/ZEB1/CHRD/ITGA8/TGFB3/LEFTY2/THBS1/CAV1/LTBP1/ZNF423/ATOH8/FSTL1/CRB2/NODAL/CCN1/DACT1/TGFB2/FBN1/WWTR1/ONECUT1/RBPMS/SMAD9/COMP/COL3A1/SHH/MIR342/EMILIN1/DAB2/DKK1/SPART/GDF9/GDF7/CDKN1C/CCN3/ZEB2/RGMB/IGSF1/VIM/DKK3/LOX/ILK/BMPR1B/LRRC32/FMOD/TGFBR3/CLDN5/LTBP3/HSPA1A/COL1A2/INHBA/INHA/LDLRAD4/LTBP2/TET1/SCUBE3/VASN/SULF1/ELAPOR2/TMPRSS6/LEF1/ACVR1/LRP1

GOBP_REGULATION_OF_CELLULAR_RESPONSE_TO_GROWTH_FACTOR_STIMULUS	GOBP_REGULATION_OF_CELLULAR_RESPONSE_TO_GROWTH_FACTOR_STIMULUS	7.03E-08	CER1/SFRP1/VTN/NGFR/CHRDL1/MYOCD/RBPMS2/CIDEA/FGF10/RGMA/SLIT2/CHRDL2/SFRP2/NOG/GREM2/GPC3/BMPER/SMOC2/FGF2/GREM1/ASPN/SFRP4/DCN/TGFB1I1/ZEB1/CHRD/AGT/ITGA8/TGFB3/ATP2B4/THBS1/HHIP/CAV1/DOK5/LTBP1/ZNF423/FSTL1/CRB2/FGFR1/CCN1/ADAMTS3/FBN1/ITGA5/ONECUT1/PRKCB/MIR342/EMILIN1/DAB2/CCBE1/DKK1/SPART/CDKN1C/ZEB2/SEMA6A/FGF1/ADGRA2/TMEM108/MIR149/DKK3/PTP4A3/LOX/ILK/TGFBR3/HSPA1A/WASF1/MIR10B/LDLRAD4/ITGB3/MMRN2/TET1/SCUBE3/VEGFC/VASN/ROBO1/SULF1/ELAPOR2/SHISA2/TMPRSS6/NREP/NRP1/FZD4/HTRA1/TMEM204/SPRED3/CD109/FZD1/FAM20C/SOX11/FUZ/LTBP4/NEDD4

GOBP_REGULATION_OF_DNA_REPLICATION	GOBP_REGULATION_OF_DNA_REPLICATION	7.93E-08	ATF1/FAF1/SMC3/WAPL/JADE2/ATR/PPP2R1A/ACTR5/ACTL6A/KCTD13/USP37/RFC3/ESCO1/UCHL5/GMNN/INO80D/ATAD5/UCN/STN1/DHX9/RUVBL1/INO80E/INO80/EHMT2/RUVBL2/RFC4/ANKRD17/CDAN1/POLE3/MCRS1/EGFR/DSCC1/DONSON/WRNIP1/BRCA2/NFRKB/INO80C/DBF4/PDS5A/RFC5/GINS1/FBXO5/RFC2/NBN/GINS2/TIPIN/STOML2/MCM7/MCM6/CDK1/TIMELESS/PCNA/NUGGC/CHTF18/GINS3/BLM/TP53/TICRR/MCM3/DNA2/ENDOG/MCM5/ORC5/CDC7/MCIDAS/CCNA2/GINS4/MCM4/ESCO2/E2F8/MCM2/E2F7/CDT1

GOBP_RNA_MODIFICATION	GOBP_RNA_MODIFICATION	8.18E-08	APOBEC3F/AARS2/DIMT1/NSUN3/APOBEC3B/METTL3/PUS7L/SNRPG/TRMT112/TARBP1/TRMT6/ALKBH8/TYW3/ALKBH5/VIRMA/APOBEC3D/TRIT1/NOP10/RAMAC/DNAJB11/ELP1/MTO1/BUD23/THUMPD3/THUMPD2/ELP5/TFB2M/TRMO/TRMT61B/TSR3/PARN/RBM15B/ADAR/HSD17B10/AARS1/FTSJ1/SNRPF/RPUSD3/MTFMT/THUMPD1/FTSJ3/MEPCE/URM1/NAF1/METTL15/TRUB2/APOBEC3G/DALRD3/SNRPB/NSUN4/APOBEC3H/TRMT11/QTRT2/METTL2A/METTL2B/APOBEC1/TRMT10A/ZCCHC4/FDXACB1/TRMT2A/NSUN2/ELP6/NAT10/DKC1/NSUN5/TRUB1/FBL/CTU1/MRM1/PUS1/TRMU/CMTR2/SNRPD1/NSUN5P1/WDR6/WDR4/GON7/DTWD2/ELP3/TGS1/PUS7/MRM3/PUSL1/CTU2/DUS2/RPUSD2/THG1L/TFB1M/ADAT2/QTRT1/RBM15/RPUSD[illegible]

GOCC_COLLAGEN_TRIMER	GOCC_COLLAGEN_TRIMER	8.49E-08	COL2A1/ADIPOQ/COL4A6/COL14A1/C1QTNF7/COL21A1/COL11A2/COLEC12/C1QTNF2/COL26A1/COL8A1/COLEC11/SCARA3/COL4A5/LUM/C1QL1/FCN1/COL24A1/COL3A1/EMILIN1/CCBE1/COL8A2/COL23A1/COL16A1/COL6A2/LOX/COL6A3/COLQ/COL1A2/COL4A4/EDA/COL15A1/COL10A1/CTHRC1/COL1A1/COL5A1/COL7A1/COL9A1/COL12A1/COL5A2/COL4A2

GOBP_REGULATION_OF_METAL_ION_TRANSPORT	GOBP_REGULATION_OF_METAL_ION_TRANSPORT	8.79E-08	PLA2G1B/GCG/KCNA1/VIP/CASQ2/PLP1/ATP1A2/DPP6/ACTN2/PLN/MYLK/ADCYAP1R1/CASQ1/KCNK3/JPH2/NKX2-5/FHL1/KCNMB1/ANK2/AKAP6/F2/KCNA5/HTR2A/ABCC9/RGN/DMD/NKAIN1/GRP/RGS4/CNTN1/KCNIP1/FLNA/ATP1B2/CACNA1C/P2RX2/TF/TRPC1/SCN4B/FXYD6/CXCL12/HRC/HSPA2/PRKD1/CACNB2/KCNE4/AGT/KCNH2/P2RX1/ATP2B4/DMPK/CAV1/GEM/FGF13/STAC/SERPINE2/HPCA/SLC8A1/PKD2/BDKRB1/CACNA2D1/KCNG1/OSR1/CCL2/ADRB2/REM1/SLN/STOM/KCNS1/PRNP/RRAD/RYR2/STC1/CLIC2/THY1/LILRA2/JPH3/NKAIN4/FGF12/ITGB3/SLC30A10

GOBP_POSITIVE_REGULATION_OF_ION_TRANSPORT	GOBP_POSITIVE_REGULATION_OF_ION_TRANSPORT	9.35E-08	PLA2G1B/GCG/SYT4/KCNA1/PLP1/RELN/TACR2/ACTN2/KCNB1/MYLK/ADCYAP1R1/CASQ1/KCNK3/JPH2/NKX2-5/FHL1/KCNMB1/ANK2/AKAP6/F2/RGN/DMD/SLC38A3/CNTN1/FLNA/ATP1B2/P2RX2/CACNG4/TRPC1/SCN4B/CXCL12/HSPA2/CACNB2/AGT/KCNH2/P2RX1/CAV1/EDNRA/STAC/ABCB1/PKD2/BDKRB1/CACNA2D1/AVPR1A/CCL2/ADRB2/OXTR/NLGN3/RAB3B/RYR2/STC1/THY1/LILRA2/FGF12/GABBR1/SYT1/CHRNB2/P2RY1/GPER1/ARC/PDGFRB/OXT/SCN3B/RAMP3/APLNR/CLTRN/CNKSR3/SCN1B/P2RY12/GSTM2/LRRC26/AMIGO1/KCNIP2/PLA2R1
GOBP_REGULATION_OF_POTASSIUM_ION_TRANSPORT	GOBP_REGULATION_OF_POTASSIUM_ION_TRANSPORT	9.49E-08	KCNA1/VIP/CASQ2/DPP6/ACTN2/KCNK3/FHL1/KCNMB1/ANK2/AKAP6/KCNA5/HTR2A/ABCC9/GRP/RGS4/KCNIP1/FLNA/ATP1B2/KCNE4/KCNH2/CAV1

GOBP_NEGATIVE_REGULATION_OF_LOCOMOTION	GOBP_NEGATIVE_REGULATION_OF_LOCOMOTION	1.22E-07	ELANE/MIR483/SFRP1/APOH/ADIPOQ/NGFR/MYOCD/MEOX2/MIR145/APOD/SLIT2/RGN/HAS1/SFRP2/NOG/DPYSL3/FBLN1/IL24/CYP1B1/ATP1B2/NAV3/FGF2/PHLDB2/TNN/PODN/GREM1/DCN/CXCL12/SEMA3E/IL33/CHRD/TPM1/PRKG1/CCL21/RECK/ATP2B4/THBS1/SERPINF1/CX3CR1/NODAL/MCC/COL3A1/SERPINE1/SHH/CLIC4/EMILIN1/CCL2/DLC1/IGFBP5/CCN3/DPEP1/CD200/GJA1/SLURP1/SEMA6A/BMERB1/SEMA6D/MIR149/CLDN19/DUSP1/RHOB/STC1/CLDN5/THY1/MEF2C/TAFA5/ADAMTS9/GPR173/PTPRM/LDLRAD4/RIPOR2/SEMA6C/MMRN2/TET1/TBX5/SEMA3G/ADORA3/ADGRB1/SEMA5B/SEMA3D/ROBO1/MIR196A1/SULF1/STARD13/LRP1/ADARB1/TIMP1/SEMA3C/NDRG4/NRP1/PFN2/RRAS/TIE1/BMP5/ANGPT2/TB[illegible]

GOBP_POSITIVE_REGULATION_OF_TRANSMEMBRANE_TRANSPORT	GOBP_POSITIVE_REGULATION_OF_TRANSMEMBRANE_TRANSPORT	1.25E-07	PLA2G1B/KCNA1/PLP1/RELN/ACTN2/ADIPOQ/ADCYAP1R1/CASQ1/KCNK3/JPH2/KCNMB1/ANK2/SORBS1/AKAP6/F2/RGN/IGF1/DMD/GPC3/CLIP3/FLNA/ATP1B2/P2RX2/CACNG4/TRPC1/NR4A3/GIP/HSPA2/CACNB2/AGT/KCNH2/P2RX1/CAV1/EDNRA/STAC/ABCB1/ITLN1/PKD2/KLF15/BDKRB1/CACNA2D1/CCL2/ADRB2

GOCC_POSTSYNAPTIC_MEMBRANE	GOCC_POSTSYNAPTIC_MEMBRANE	1.28E-07	KCNA1/CHRM2/GRIK3/CHRNA3/KCNB1/KCNMA1/GRIK1/EPHA7/ANK2/GRIN2A/HTR2A/DMD/GRIK5/GLRB/CDH2/CNTN1/PLPPR4/CACNA1C/GABBR2/CACNG4/NLGN4X/NLGN1/FXYD6/ADCY1/NLGN4Y/DLG2/RGS7BP/CHRNB4/P2RX1/SSPN/CHRM4/LIN7A/SORCS2/PDLIM4/MAGEE1/NRP2/CLSTN2/SLC8A1/SHC4/SYNE1/PRRT1/EFNB3/NLGN3/KCND1/LRRC4/DLG4/LRRC4C/KCND2/SYNDIG1/IQSEC3/NRCAM/NLGN2/GABBR1/GABRR1/ITGB3/CHRNB2/PCDH17/P2RY1/MUSK/ARC/GABRE/NRP1/LRRC4B/IGSF21/GRID1/SNTA1/TAMALIN/CHRM3/GRIP1/KCTD12/SLC6A4/CNIH2

GOCC_NUCLEOSOME	GOCC_NUCLEOSOME	1.47E-07	H2AC19/IRF4/H4C13/H3C12/EP400/H3-3B/BRD8/H2BC7/MRGBP/H1-10/H3C10/ZNHIT1/MACROH2A1/ACTL6A/KAT5/H3-3A/H2AW/SPHK2/ACTR6/H4C9/H3C1/H2BC11/H4C4/RUVBL1/H4C3/SLF1/RUVBL2/H2BC9/H1-2/H4C2/H2BC18/SRCAP/H2AC18/H4C1/KDM1B/H2BC10/H1-3/H3C13/H3C2/H3C7/H2AC20/CENPA/H2BC12/H2AZ1/H2BC15/H2AC13/H2BC14/H3C6/H2AC21/H2AX/H3C8/H2BC13/H2BC17/H3C11/H1-4/H3C3/H2AC12/H2AC16/H1-5

GOBP_CARTILAGE_DEVELOPMENT	GOBP_CARTILAGE_DEVELOPMENT	1.61E-07	HAND1/OGN/CER1/COL2A1/BMP3/HAND2/MGP/CHRDL2/GHR/SIX2/SFRP2/GDF6/NOG/EFEMP1/NKX3-2/COL11A2/FGF2/ZBTB16/FRZB/GREM1/PTH1R/GLI3/ZEB1/WNT9A/RSPO2/MATN3/SOX5/EVC/CCN1/CYTL1/COMP/COL3A1/OSR1/PRRX1/CCN3/CTSK/WNT2B/WNT10B/EPYC/SNAI2/CCN4/CCN2/PTHLH/MAF/TGFBI/BMPR1B/STC1/MEF2C/LTBP3/ANXA6/BGN/GPLD1/HOXA5/TRPS1/SULF1/COL1A1/TIMP1

GOBP_RIBOSOMAL_SMALL_SUBUNIT_BIOGENESIS	GOBP_RIBOSOMAL_SMALL_SUBUNIT_BIOGENESIS	1.63E-07	RPL38/RRP36/RPS24/RPS21/SRFBP1/LSM6/RIOK1/RIOK2/RPS8/RPS16/MRPS7/LTV1/RIOK3/UTP25/ERCC2/RPS27L/DDX52/RPS14/DCAF13/NOM1/WDR46/KRI1/ERAL1/HEATR1/NOL10/TSR3/UTP3/RPS5/MRPS11/UTP4/XRCC5/METTL17/RPS7/IMP3/WDR43/RPS28/ABT1/RPS19/RRP7A/BMS1/RPS6/UTP20/NAT10/RCL1/NOL11/DHX37/RPS17/TSR1/BYSL/NOP9/HELB/PRKDC/RPS15/RPSA/WDR3/TBL3/IMP4/RRS1/NOB1/NPM1/SURF6/PWP2/NOP14

GOBP_NEGATIVE_REGULATION_OF_TRANSPORT	GOBP_NEGATIVE_REGULATION_OF_TRANSPORT	1.71E-07	PRTN3/SYT4/APOA2/SPX/CARTPT/VIP/CASQ2/APOC3/ATP1A2/TACR2/SFRP1/ACTN2/ADIPOQ/KCNB1/PLN/CRYAB/SCT/APOD/HTR2A/GRP/EPHA3/MAP1B/RGS4/PTGER3/CNR1/SFRP4/GPM6B/PRKD1/KCNE4/CD36/KCNH2/CHGA/FOXF1/RAB23/THBS1/PID1/CAV1/GEM/PIP4P2/RGS2/ANGPT1/SERPINE2/PKD2/PRKCB/PRKN/PKIA/SHH/OSR1/PKIG/SYT11/MIR27B/FFAR4/CCN3/OXTR/LRRK2/REM1/CD200/SLN/GJA1/MIR326/DLG4/IL11/AVPR2/KLF7/RRAD/STC1/CLIC2/INHBA/INHA/GABBR1/FCGR2B/TWIST1/ITGB3/IL13RA2

GOBP_POSITIVE_REGULATION_OF_MAPK_CASCADE	GOBP_POSITIVE_REGULATION_OF_MAPK_CASCADE	1.91E-07	PLA2G1B/ELANE/GCG/CARTPT/CCL16/HAND2/FGA/FGF10/ADCYAP1/HTR2A/ACTA2/IGF1/FGG/GHR/EPGN/AR/PLA2G5/GDF6/CCL26/FERMT2/HTR2B/CDH2/NPY/ROR2/BMPER/DDR2/ADRA1D/FGF2/MMP8/DIXDC1/ALKAL1/IGF2/CDON/THPO/TNFAIP8L3/SERPINF2/CD36/CCL21/TGFB3/ADRA1B/AKAP12/DOK6/THBS1/CCL19/LPAR3/DOK5/CSPG4/FZD10/IGFBP6/LPAR1/HGF/NODAL/EDA2R/FGFR1/ANGPT1/TGFB2/ITGA1/TENM1/IGFBP4/GPBAR1/IL6/PDGFRA/CCL2/ANKRD6/DKK1/ADRB2/MIR27B/GPR183/NTF3/FFAR4/FZD7/LRRK2/KLB/PDGFD/CCN2/CCL11/FGF1/IL11/SH3RF3/FGB/GPNMB/KL/NOX4/TEK/PDE5A/MEF2C/MIR222/INHBA/S100A12/PDGFC/GADD45G/FCGR2B/ITGB3/SLC30A10/ROBO1/P2RY1/FLT3/GPER1/CCR7[illegible]

GOBP_RNA_TEMPLATED_DNA_BIOSYNTHETIC_PROCESS	GOBP_RNA_TEMPLATED_DNA_BIOSYNTHETIC_PROCESS	2.15E-07	TEP1/RAD50/MAPKAPK5/SMG6/HSP90AB1/NOP10/CTNNB1/TENT4B/CCT6A/PNKP/MAP3K4/FBXO4/ATR/PARN/TERF2/HSP90AA1/MAP2K7/SMG5/XRCC5/NAF1/RFC1/XRN1/ACD/RPA1/STN1/SRC/NAT10/DKC1/HNRNPU/HNRNPC/MAPK3/TCP1/EXOSC10/PTGES3/NEK2/PIF1/CCT8/CCT2/CCT3/PINX1/CCT4/CCT7/CCT5/AURKB/GAR1/TELO2/HNRNPA1/WRAP53/NHP2/PRKCQ/TNKS/TERT/MAPK15

GOBP_CALCIUM_ION_TRANSPORT	GOBP_CALCIUM_ION_TRANSPORT	2.41E-07	PLA2G1B/GCG/CSN2/CASQ2/PLP1/ATP1A2/PLN/MYLK/ADCYAP1R1/CASQ1/JPH2/GPM6A/ANK2/GRIN2A/AKAP6/F2/SLC8A2/HTR2A/RGN/DMD/RYR3/HTR2B/RGS4/FLNA/CACNA1C/P2RX2/FGF2/TRPC4/CACNG4/TRPC1/CACNA1H/CXCL12/HRC/HSPA2/PRKD1/CACNB2/AGT/CCL21/NCS1/P2RX1/ATP2B4/CCL19/TRPM6/MICU3/CAV1/GEM/EDNRA/STAC/NMUR1/CLCA1/HPCA/SLC8A1/PKD2/BDKRB1/CACNA2D3/CACNA2D1/PRKCB/CDH23/CACNA1A/CYSLTR1/CCL2/REM1/SLN/SLC24A3/CCN2/PRNP/RAMP2/ITPR1/RRAD/RYR2/STC1/CLIC2/THY1/LILRA2/JPH3/ANXA6/MCOLN3/ITGB3/CHRNB2/CXCR4/GPER1/CCR7/CACNG6/PDGFRB/STC2/BEST1

GOBP_REGULATION_OF_CHROMOSOME_SEGREGATION	GOBP_REGULATION_OF_CHROMOSOME_SEGREGATION	2.43E-07	NCAPH2/ZW10/RAD18/RAD21/BCL7A/SMC4/TTK/KNTC1/XRCC3/FBXO5/ZWILCH/AURKB/NDC80/BUB1/SMARCD2/BUB3/SMARCE1/GEN1/SPC25/SMARCA4/SMC2/PTTG1/CENPF/INCENP/NCAPG2/SPC24/BUB1B/DLGAP5/CDC20/RMI2/RCC2/NCAPH/CCNB1/ESPL1/KIF2C/TACC3/HASPIN/MAPK15/MAD2L1/CENPE/PLK1/BIRC5/CDCA8/MKI67/NCAPG/ZWINT/CDT1/CDCA2

GOBP_REGULATION_OF_CATION_TRANSMEMBRANE_TRANSPORT	GOBP_REGULATION_OF_CATION_TRANSMEMBRANE_TRANSPORT	2.98E-07	PLA2G1B/KCNA1/CASQ2/PLP1/RELN/ATP1A2/DPP6/ACTN2/PLN/ADCYAP1R1/CASQ1/KCNK3/JPH2/FHL1/KCNMB1/ANK2/GRIN2A/AKAP6/F2/ABCC9/RGN/DMD/GRP/RGS4/KCNIP1/FLNA/ATP1B2/CACNA1C/P2RX2/CACNG4/TRPC1/NLGN1/SCN4B/FXYD6/HRC/HSPA2/PRKD1/CACNB2/KCNE4/AGT/KCNH2/P2RX1/ATP2B4/CAV1/GEM/EDNRA/FGF13/RGS2/STAC/HPCA/SLC8A1/PKD2/BDKRB1/CACNA2D1/PRRT1/KCNG1/OSR1/CCL2/ADRB2/NLGN3/REM1/SLN/STOM/DLG4/KCNS1/PRNP/RRAD/RYR2/CLIC2/THY1/MEF2C/NLGN2/JPH3/FGF12/TWIST1/ITGB3/RASGRF2

GOMF_DNA_HELICASE_ACTIVITY	GOMF_DNA_HELICASE_ACTIVITY	3.72E-07	RFC3/RTEL1/XRCC5/SMARCAD1/DHX9/RUVBL1/FANCM/RUVBL2/RFC4/PIF1/RAD54B/G3BP1/UPF1/SUPV3L1/MCM9/DSCC1/WRNIP1/WRN/ASCC3/HELB/RFC5/DDX11/RFC2/DDX12P/ERCC6L/MCM7/MCM6/TWNK/CHTF18/BLM/POLQ/RAD51/MCM3/DNA2/DQX1/MCM5/RECQL4/RAD54L/MCM4/BRIP1/MCM2

GOBP_CARDIOCYTE_DIFFERENTIATION	GOBP_CARDIOCYTE_DIFFERENTIATION	4.00E-07	MYH11/HAND2/ACTN2/MYOCD/NKX2-5/MIR145/BVES/AKAP6/IGF1/ASB2/PI16/RGS4/SGCD/GREM1/WT1/AGT/PRICKLE1/ACTC1/EDNRA/RGS2/TBX18/TGFB2/SLC8A1/FHOD3/PDGFRA/DKK1/ALPK3/ISL1/FZD7/SORBS2/TENM4/SOX17/NOX4/TGFBR3/MEF2C/MIR222/INHBA/TWIST1/IRX3/TBX5/GPER1/ACVR1/PDGFRB/HAMP/SEMA3C

GOBP_POSITIVE_REGULATION_OF_TELOMERE_MAINTENANCE	GOBP_POSITIVE_REGULATION_OF_TELOMERE_MAINTENANCE	4.24E-07	CCT6A/PNKP/MAP3K4/FBXO4/PPP1R10/ATR/NABP2/PARN/TERF2/ACTL6A/RTEL1/MAP2K7/XRCC5/NAF1/UCHL5/ACD/INO80D/RUVBL1/INO80E/INO80/DKC1/SLX4/MAPK3/PML/RUVBL2/TCP1/NEK2/HNRNPD/CCT8/MCRS1/CCT2/HNRNPA2B1/CCT3/GNL3/NFRKB/CCT4/CCT7/CCT5/AURKB/SIRT6/NBN/HNRNPA1/PRKCQ/TNKS/MAPK15

GOBP_SPINDLE_ORGANIZATION	GOBP_SPINDLE_ORGANIZATION	4.21E-07	CHMP6/TUBGCP5/EML3/PPP2R1A/GOLGA2/RPS3/HDAC3/TUBG1/KPNB1/GOLGA2P5/STAG1/CHMP2B/NUP62/HAUS2/PDCD6IP/CHEK2/RAB11A/HAUS4/CHMP7/INTS13/KIF2A/CHMP1A/TPX2/CEP192/VPS4B/RNF4/INO80/LIMK2/GPSM2/HNRNPU/NUF2/CHMP5/NEK2/RAN/CKAP5/HAUS6/SASS6/HAUS5/KNSTRN/TTK/STMN1/CEP72/SMC1A/PSRC1/PRC1/NTMT1/TUBGCP4/MAP1S/TUBGCP2/FAM110A/CCSAP/HAUS8/PCNT/VCP/FBXO5/AURKB/NDC80/ASPM/SPAG5/KIF23/WRAP73/PTPA/AURKA/MZT1/TNKS/SPC25/NUDC/KIFC1/WDR62/PLK2/BORA/RGS14/CCNB2/INCENP/SAC3D1/KIF4A/RACGAP1/TPPP/PARP3/DLGAP5/CDC20/AUNIP/CHMP4C/KIFC2/CCNB1/ESPL1/TACC3/MAPK15/CENPE/MISP/PLK1/BIRC5/CENPH/CDCA8/KIF15/RCC[illegible]

GOBP_ACTION_POTENTIAL	GOBP_ACTION_POTENTIAL	4.48E-07	KCNA1/ATP1A2/SCN7A/KCNB1/TACR1/CTNNA3/ANK2/AKAP6/KCNA5/SLC8A2/DMD/KCNH6/FLNA/CACNA1C/CNR1/CACNA1H/SCN4B/KCNJ8/CACNB2/KCNE4/CD36/KCNH2/CNTNAP1/CHRNB4/GJC1/P2RX1/CAV1/CNR2/SCN3A/FGF13/TBX18/SLC8A1/CACNA2D1/GJA1/CLDN19/KCND2/RYR2/NRCAM/GNA14/FGF12/CHRNB2

GOBP_SIGNAL_RELEASE	GOBP_SIGNAL_RELEASE	5.09E-07	GCG/SYT4/UCN3/CARTPT/CELA2A/GHRH/VIP/CHRM2/AGTR1/TACR2/SFRP1/ADIPOQ/NEUROD1/CHRNA3/NNAT/KCNB1/SCG5/FGA/TACR1/ADCYAP1/NRXN2/ADCY5/CPE/SCT/KCNA5/HTR2A/PTPRN/FGG/GRIK5/GRP/CCKAR/CNR1/NLGN1/CXCL12/ADCY1/GIP/CACNB2/AGT/CHGA/CHRNB4/SV2A/P2RX1/RFX6/NKX6-1/SELENOM/LIN7A/RBP4/IL6/PRKCB/PRKN/NR1H4/DAB2/CGA/SNAP25/SYT11/GDF9/ISL1/FFAR4/GLP1R/CCN3/RETN/OXTR/LRRK2/SNCG/GJA1/BRSK1/GPR27/IL11/ABCA1/KLF7/FGB/MEF2C/NLGN2/INHBA/INHA/APBA1/BLK/GABBR1/SLC16A2/GPLD1/SYT1/MYRIP/WLS/ADORA3/CHRNB2/SLC16A10/SYP/P2RY1/GPER1/SYT5/FFAR2/OXT/CADPS/SNCAIP/EDNRB/FZD4/BACE1/PFN2

GOBP_REGULATION_OF_CHROMOSOME_SEPARATION	GOBP_REGULATION_OF_CHROMOSOME_SEPARATION	5.25E-07	CUL3/ANAPC4/MAD2L1BP/ANAPC1/NEK6/TPR/MAD2L2/SMARCD1/BRD7/SPDL1/CDC23/SMARCC1/ACTL6A/RB1/ANAPC15/ANAPC5/SMARCC2/TRIP13/SMARCB1/ANAPC2/NUF2/NCAPH2/ZW10/RAD21/BCL7A/SMC4/TTK/KNTC1/XRCC3/FBXO5/ZWILCH/AURKB/NDC80/BUB1/SMARCD2/BUB3/SMARCE1/GEN1/SPC25/SMARCA4/SMC2/PTTG1/CENPF/INCENP/NCAPG2/SPC24/BUB1B/DLGAP5/CDC20/NCAPH/CCNB1/ESPL1/TACC3/HASPIN/MAPK15/MAD2L1/CENPE/PLK1/BIRC5/CDCA8/NCAPG/ZWINT/CDT1

GOMF_HELICASE_ACTIVITY	GOMF_HELICASE_ACTIVITY	5.72E-07	DDX51/DHX33/RFC3/DDX20/MTREX/RTEL1/XRCC5/SMARCAD1/DHX38/DHX58/DDX41/DHX9/RUVBL1/AQR/DDX56/EIF4A3/SNRNP200/FANCM/RUVBL2/DDX28/DDX21/RFC4/DDX23/DHX37/PIF1/RAD54B/DHX34/G3BP1/DDX60/UPF1/SRCAP/SUPV3L1/MCM9/DSCC1/YTHDC2/WRNIP1/WRN/ASCC3/DDX49/HELB/RFC5/DDX11/DDX31/DHX30/DDX1/ZGRF1/RFC2/DHX15/DDX12P/MOV10/DDX54/ERCC6L/MCM7/MCM6/SMARCA4/TWNK/HELZ2/DDX39A/CHTF18/BLM/POLQ/RAD51/TTF2/MCM3/DNA2/DQX1/MCM5/HELLS/RECQL4/RAD54L/MCM4/BRIP1/MCM2
GOBP_CELLULAR_COMPONENT_ASSEMBLY_INVOLVED_IN_MORPHOGENESIS	GOBP_CELLULAR_COMPONENT_ASSEMBLY_INVOLVED_IN_MORPHOGENESIS	5.67E-07	MYH11/LDB3/ACTN2/PGM5/FLNC/LMOD1/CASQ1/NKX2-5/MYL9/ANKRD1/CCDC136/ANK2/TMOD1/KLHL41/CFL2/CNTN1/CSRP1/PHLDB2/ACTA1/TPM1/CNTNAP1/ACTC1/LMOD3/FHOD3/PDGFRA/CSRP2/EPB41L3/TENM4/PHLDB1/PMP22

GOCC_INTRINSIC_COMPONENT_OF_SYNAPTIC_MEMBRANE	GOCC_INTRINSIC_COMPONENT_OF_SYNAPTIC_MEMBRANE	5.71E-07	KCNA1/CHRM2/EPHA7/GPM6A/CADM3/GRIN2A/HTR2A/GRIK5/CDH2/CNTN1/PLPPR4/ADAM23/CACNG4/CNR1/NLGN4X/NLGN1/PTPRD/FXYD6/ADCY1/NLGN4Y/RGS7BP/P2RX1/ATP2B4/NRP2/CLSTN2/SLC8A1/EFNB3/NLGN3/LRRC4/DLG4/LRRC4C/KCND2/NRCAM/NLGN2/GABBR1/ITGB3/CHRNB2/PCDH17/P2RY1

GOBP_SIGNAL_TRANSDUCTION_IN_RESPONSE_TO_DNA_DAMAGE	GOBP_SIGNAL_TRANSDUCTION_IN_RESPONSE_TO_DNA_DAMAGE	5.76E-07	CDK2/SMYD2/UIMC1/RPS27L/RPL26/TP53BP1/MAD2L2/HINFP/RPA2/CRADD/BATF/COPS3/FBXO4/PPP1R10/ATR/RAD9A/NDRG1/CDK5RAP3/BID/ZNHIT1/BCL3/RAD9B/KAT5/NEK11/USP10/MYO6/USP28/GRB2/XPC/ATAD5/CUL4A/CHEK2/PRPF19/BRCC3/BRCA1/INTS7/KMT5A/TOPBP1/PML/KDM1A/SESN2/BARD1/EME1/MUS81/PPM1D/PAXIP1/DONSON/ATRIP/BRCA2/TRIAP1/CCNG1/TAOK2/RINT1/PRKDC/PIDD1/YJU2/MSX1/FZR1/CASP2/CCAR2/RFWD3/CDC14B/TFAP4/NBN/TELO2/MSH2/TIPIN/GTSE1/CD74/NPM1/CDK1/PLK2/EME2/TTI2/FOXM1/FBXO6/CHEK1/PMAIP1/E2F1/DOT1L/BLM/TP53/RAD51/TICRR/CLSPN/FANCD2/DTL/H2AX/MDM2/PLK1/CCND1/WDR76/BRIP1/E2F7/MUC1/ACER2/PBK

GOBP_AXON_DEVELOPMENT	GOBP_AXON_DEVELOPMENT	5.75E-07	PLP1/RELN/NGFR/EPHA7/RGMA/NRXN3/BOC/APOD/SLIT2/FLRT2/GAP43/NEXN/NFASC/CHODL/NOG/GFRA3/ARHGEF25/CDH2/SLIT3/EPHA3/MAP1B/MAP6/CNTN1/FLNA/NGF/CRTAC1/PLPPR4/PAK3/CCKAR/PTN/TNC/TNN/DAB1/PLXNA4/CNR1/NDN/MAP1A/DIXDC1/NPTX1/LAMA2/CXCL12/GLI3/ADCY1/SEMA3E/NCAM2/NTN1/CNTN4/VSTM2L/CNTNAP1/CHL1/MAPT/RND2/LAMA1/APBB1/LPAR3/NKX6-1/EDNRA/NCAM1/PALLD/FGF13/L1CAM/NRP2/FEZ1/MMP2/TSPAN2/CDH11/HOXA2/APOA4/SHH/SMO/FN1/SLITRK6/SPART/KIF5A/ISL1/EFNB3/GDF7/UCHL1/NLGN3/CRABP2/DPYSL5/BRSK1/ATOH1/SEMA6A/SEMA6D/NEFL/LRRC4C/BMPR1B/KLF7/ISLR2/PTPRZ1/THY1/VASH2/MIR222/NRCAM/S100B/ANOS1/PTPRM/PTCH1/SEM[illegible]

GOBP_SMOOTH_MUSCLE_CONTRACTION	GOBP_SMOOTH_MUSCLE_CONTRACTION	5.91E-07	SPX/CHRM2/CNN1/MYH11/ATP1A2/TACR2/CHRNA3/MYLK/TACR1/KCNMA1/MYOCD/MIR145/HTR2A/ACTA2/HTR2B/P2RX2/PTGER3/SMTN/GUCY1A1/AGT/PRKG1/CHRNB4/P2RX1/ATP2B4/APBB1/CAV1/EDNRA/RGS2/NMUR1/SLC8A1/COMP/ADRB2/OXTR/SSTR2

GOBP_CHROMOSOME_SEPARATION	GOBP_CHROMOSOME_SEPARATION	6.23E-07	CUL3/ANAPC4/RECQL5/MAD2L1BP/TOP2B/ANAPC1/M1AP/DIS3L2/NEK6/TPR/MAD2L2/SMARCD1/BRD7/CENPS/SPDL1/PPP2R1A/CDC23/SMARCC1/ACTL6A/RB1/ANAPC15/ANAPC5/SMARCC2/SMARCAD1/TOP3A/TRIP13/SMARCB1/FANCM/SLX4/ANAPC2/NUF2/EME1/MUS81/NCAPH2/ZW10/RAD21/BCL7A/SMC4/TTK/KNTC1/XRCC3/FBXO5/ZWILCH/AURKB/NDC80/BUB1/SMARCD2/BUB3/RMI1/SMARCE1/GEN1/SPC25/SMARCA4/SMC2/PTTG1/EME2/CENPX/CENPF/INCENP/NCAPG2/SPC24/BUB1B/DLGAP5/CDC20/NCAPH/CCNB1/ESPL1/TACC3/HASPIN/MAPK15/MAD2L1/CENPE/PLK1/BIRC5/CDCA8/TOP2A/NCAPG/ZWINT/CDT1

GOBP_RIBOSOMAL_LARGE_SUBUNIT_BIOGENESIS	GOBP_RIBOSOMAL_LARGE_SUBUNIT_BIOGENESIS	6.75E-07	RPL11/RPL38/RPF1/CINP/GTPBP4/RPL24/NOL9/NIFK/DDX18/EIF6/RPL23A/FASTKD2/PAK1IP1/RPL10A/MRPL20/ZNHIT3/MAK16/BRIX1/RPL26/RPL6/RPF2/NSA2/RPL14/TMA16/LAS1L/WDR12/FTSJ3/RPL26L1/RSL1D1/RSL24D1/RPL7A/LTO1/C1orf109/RPL35/RPL7/MDN1/RPL7L1/NIP7/DDX28/HEATR3/RPLP0/MRPL1/RPLP0P6/WDR74/EBNA1BP2/PES1/DHX30/TRAF7/NLE1/NOP16/URB1/SPATA5/NHP2/RRS1/SPATA5L1/NPM1/MRTO4/SURF6/BOP1/NOC2L/PPAN

GOBP_REGULATION_OF_MUSCLE_SYSTEM_PROCESS	GOBP_REGULATION_OF_MUSCLE_SYSTEM_PROCESS	7.68E-07	SPX/KCNA1/CASQ2/CHRM2/CNN1/ATP1A2/TACR2/HAND2/CHRNA3/PLN/TACR1/KCNMA1/CASQ1/MYOCD/NKX2-5/MYL9/CTNNA3/SGCA/MIR145/ANK2/PPP1R12B/AKAP6/IGF1/DMD/MYL3/PI16/RGS4/CACNA1C/NR4A3/GUCY1A1/HRC/PDE9A/AGT/TPM1/PRKG1/CHGA/FBXO32/ADRA1B/CHRNB4/P2RX1/ATP2B4/DMPK/CAV1/FGF13/RGS2/SLC8A1/ADRB2/IGFBP5/OXTR/SLN/CCN2/SSTR2

GOCC_TRANSFERASE_COMPLEX_TRANSFERRING_PHOSPHORUS_CONTAINING_GROUPS	GOCC_TRANSFERASE_COMPLEX_TRANSFERRING_PHOSPHORUS_CONTAINING_GROUPS	7.71E-07	GTF2B/RPRD1A/CDC73/ERN1/DAPK1/CSNK2A1/GTF2A1/DBF4B/PHKB/ERCC3/SMCR8/CRCP/CISH/CDK13/TAF9/CDKN2D/TAF12/ATG13/CSNK2A2/CCNT2/TAF2/TADA3/RPAP2/TRRAP/POLR3D/CHRAC1/PRKAG1/ACVR1B/TAF5L/PRPSAP2/RECQL5/POLR2C/USP22/CDK2/ERCC2/XRCC6/CDK12/TENT2/MAD2L2/GTF2H2/BCCIP/CCNQ/CCNT1/GTF2H2C/SOCS7/TCEA1/PHKG2/CDK7/POLR2H/CCND3/PRPSAP1/IKBKB/CTR9/CCNK/CDK5R1/LEO1/POLR1C/LAS1L/POLR2L/XRCC5/POLR1E/MYO6/PIK3R3/CCNC/POLR2D/MMS19/POLA1/TAF6L/ATXN7L3/CDK16/VAC14/POLR3E/PEX2/TAF4B/PRIM2/POLR3H/RPAP1/CCNJ/CNPPD1/POLE2/PIK3CB/MAP3K5/ACVR1C/POLR2E/CCNI2/POLR1A/POLR3B/STK11/POLR3A/POLR2B/PYCARD/SLA2/CKS[illegible]

GOBP_MUSCLE_CELL_PROLIFERATION	GOBP_MUSCLE_CELL_PROLIFERATION	8.10E-07	ELANE/VIP/OGN/CNN1/ADIPOQ/TACR1/MYOCD/NKX2-5/RBPMS2/MIR145/NPR3/APOD/IGF1/NOG/KCNK2/DDR2/ZFPM2/FGF2/VIPR2/NR4A3/ELN/SERPINF2/AGT/TPM1/PRKG1/TGFB3/ADAMTS1/THBS1/GLI1/HGF/NPR1/ANGPT1/TGFB2/RBP4/MMP2/IL6/SHH/EFEMP2/MIR27B/IGFBP5/CCN3/GJA1/PDGFD/FOXC2/CCN4/PTGIR/S1PR1/TENM4/TGFBR3/MEF2C/MIR222/TAFA5

GOBP_NUCLEOBASE_CONTAINING_COMPOUND_TRANSPORT	GOBP_NUCLEOBASE_CONTAINING_COMPOUND_TRANSPORT	8.42E-07	SIDT2/PNPT1/NXT1/AKAP8L/DDX19B/HNRNPA1L2/SLC35C2/NUP133/FMR1/RANBP2/SENP2/POLDIP3/G3BP2/NUP153/ALKBH5/SMG6/CETN3/RIPK1/XPO5/NUP98/NUP214/CPSF6/NCBP3/SLC29A3/MAGOH/PABPN1/SLC35B3/TPR/LRRC8D/SRSF7/SETD2/AAAS/SMG7/SLC25A23/NUP54/NUTF2/RBM15B/THOC6/AHCTF1/ABCD1/IGF2BP2/MRPL18/SMG1/SLC25A17/XPO1/NUP160/SEH1L/KPNB1/SLC35A1/SMG5/SRSF3/LRRC8A/NUP35/CHTOP/SLC35D1/NUP62/NUP62CL/POLR2D/NCBP1/KHDRBS1/DHX9/POM121C/NUP85/NSUN2/NUP43/EIF4A3/SRSF1/CD47/HSF1/SLC25A5/SLC35B1/GJB1/SLC35A2/NUP58/NUP93/SLC17A9/UPF1/HNRNPA2B1/CASC3/SLC25A33/YBX1/MCM3AP/RAN/POM121/CKAP5/SLC35D2/ZC3H3/SLC25A24/NUP[illegible]
GOCC_PLATELET_ALPHA_GRANULE_LUMEN	GOCC_PLATELET_ALPHA_GRANULE_LUMEN	8.66E-07	ALB/AHSG/ACTN2/FGA/IGF1/FGG/IGF2/F13A1/SERPINF2/MMRN1/TGFB3/LEFTY2/THBS1/HGF/SERPINA3/TGFB2/SERPINE1/ORM2/PROS1/FN1/F8/A2M/ISLR/FGB/ACTN1/SPARC/SERPING1/CLU/VEGFC/TIMP1/SRGN

GOBP_REGULATION_OF_BLOOD_PRESSURE	GOBP_REGULATION_OF_BLOOD_PRESSURE	9.13E-07	SPX/CARTPT/NPPB/ATP1A2/AGTR1/ADIPOQ/TACR1/CTSG/CMA1/NPR3/ABCC9/ACTA2/AR/ADAMTS16/NPY/ADRA1D/TAC3/P2RX2/CNR1/GUCY1A1/SERPINF2/AGT/TPM1/CHGA/ADRA1B/P2RX1/CPA3/EDNRA/NPR1/BDKRB1/CORIN/AVPR1A/HBB/EMILIN1/ADRB2/TRHDE/MIR27B/GLP1R/OXTR/AVPR2/ENPEP/RAMP2/PTGS1/RNLS/KL/COL1A2/NPR2/CORO2B/VEGFC/CD34/NCALD/OXT/UTS2R/EDNRB

GOCC_NUCLEAR_PORE	GOCC_NUCLEAR_PORE	9.24E-07	NUP155/AGFG1/NUP107/NXT1/DDX19B/NUP133/RANBP2/SENP2/SUMO1/NUP153/CETN3/UBE2I/VPS4A/NUP98/NUP214/TPR/AAAS/IPO5/XPO4/NUP54/NUTF2/RANBP3/CHMP6/AHCTF1/NUP160/SEH1L/KPNB1/NUP35/CHMP2B/NUP62/NUP62CL/CHMP7/CHMP1A/VPS4B/POM121C/NUP85/NUP43/CHMP5/NUP58/NUP93/MCM3AP/RAN/POM121/XPO7/RANBP1/NUP205/NUP37/NUP88/RANGAP1/SEC13/NUP50/MVP/GLE1/TNKS/NDC1/NUP188/CHMP4C/POM121B/EIF5A/MAD2L1/EIF5AL1

GOMF_DAMAGED_DNA_BINDING	GOMF_DAMAGED_DNA_BINDING	9.33E-07	MSH3/NEIL2/ERCC2/TDG/XRCC6/TP53BP1/RPA3/PNKP/RPA2/DDB1/RAD23B/RPS3/XRCC5/XPC/MSH6/RPA1/POLH/BRCA1/XRCC1/POLB/APTX/PARP1/ERCC5/MPG/NTHL1/WRN/RAD18/RAD23A/HMGB2/UNG/FANCG/SIRT6/RBBP8/NBN/MSH2/DCLRE1B/NEIL3/DDB2/PCNA/BLM/POLQ/DCLRE1A/AUNIP/FEN1/H2AX/RECQL4/POLD1

GOMF_STRUCTURAL_CONSTITUENT_OF_CHROMATIN	GOMF_STRUCTURAL_CONSTITUENT_OF_CHROMATIN	9.55E-07	H2AC19/H4C13/H3C12/H3-3B/H2BC7/H1-10/H3C10/MACROH2A1/H3-3A/H2AW/H4C9/H3C1/H2BC11/H4C4/H4C3/H2BC9/H1-2/H4C2/H2BC18/H2AC18/H4C1/H2BC10/H1-3/H3C13/H3C2/H3C7/H2AC20/CENPA/H2BC12/H2AZ1/H2BC15/H2AC13/HMGA1/H2BC14/H3C6/H2AC21/H2AX/H3C8/H2BC13/H2BC17/H3C11/H1-4/LMNTD2/H3C3/ASF1B/H2AC12/H2AC16/H1-5

GOBP_MITOCHONDRIAL_MEMBRANE_ORGANIZATION	GOBP_MITOCHONDRIAL_MEMBRANE_ORGANIZATION	9.44E-07	TIMM50/TOMM70/MFN2/PEX5/TIMM22/TMEM11/BID/TIMM8B/SAMM50/BAD/HSP90AA1/CHCHD6/TOMM22/CALM3/BCL2L1/MTX1/MTX2/APOO/SLC35F6/GSK3A/HSPA4/MICOS13/HEBP2/BCS1L/RTL10/AFG3L2/ALKBH7/TIMM29/BAX/TOMM5/SLC25A5/IMMT/NDUFA13/RHOT2/UQCC3/AP3B1/ATP5IF1/ZNF205/HSPA9/CHCHD3/GHITM/TIMM13/DNAJC11/TOMM40/HIP1R/PPIF/COX18/CHCHD10/VDAC2/PMAIP1/TP53/BAK1/TMEM102/LETM1/BBC3/HK2/SLC9A1/GZMB
GOBP_REGULATION_OF_SYNAPTIC_TRANSMISSION_GLUTAMATERGIC	GOBP_REGULATION_OF_SYNAPTIC_TRANSMISSION_GLUTAMATERGIC	9.51E-07	RELN/ATP1A2/FRRS1L/GRIK3/GRIK1/GRIN2A/HTR2A/CDH2/ROR2/PLPPR4/CACNG4/CNR1/NLGN1/SERPINE2/CCL2/DKK1/OXTR/NLGN3/LRRK2/TSHZ3/MEF2C/NLGN2/SYT1

GOBP_ERK1_AND_ERK2_CASCADE	GOBP_ERK1_AND_ERK2_CASCADE	9.78E-07	GCG/CCL16/APOA1/HAND2/ADIPOQ/FGA/FGF10/EPHA7/ADCYAP1/MIR145/HTR2A/ACTA2/IGF1/FGG/LMO3/PLA2G5/CCL26/FERMT2/HTR2B/RPS6KA6/FBLN1/NPY/ROR2/BMPER/DDR2/FGF2/TF/DUSP26/ALKAL1/THPO/TNFAIP8L3/SERPINF2/AGT/CD36/CCL21/ROS1/AKAP12/CCL19/NODAL/CCN1/ANGPT1/GPBAR1/EMILIN1/PDGFRA/DAB2/FN1/CCL2/MIR27B/GPR183/FFAR4/FAM83D/OXTR/PDGFD/CCN2/SEMA6A/CCL11/FGF1/DUSP9/FGB/GPNMB/NOX4/TGFBR3/TEK/MIR222/TIMP3/INHBA/PDGFC/ITGB3/SLC30A10/P2RY1/GPER1/CCR7/ROR1/ACKR3/PDGFRB/NDRG4/NRP1/MAP3K12/RAMP3/RRAS/SPRED3/FPR2/CNKSR3

GOBP_CYTOPLASMIC_TRANSLATION	GOBP_CYTOPLASMIC_TRANSLATION	9.99E-07	RPS25/RPL27A/MCTS1/RPLP1/RPL13/RPS15A/ETF1/ZNF385A/RPL11/RPS13/RPL34/RPL38/RPS24/DRG2/PAIP1/RPS21/EIF4A1/RPL24/METTL3/RPLP2/FMR1/RPS11/UBA52/DPH5/EIF3M/RPS8/RPS16/EIF3K/RPL22/RPS4X/RPS12/RPL23A/EIF3J/DRG1/RPL15/RPL10A/MIF4GD/RPS10/FAU/RPL32/EIF2S3/RPL26/RPS14/RPL6/RPS9/DENR/EIF2D/RPL37A/RPL13A/EIF3B/RPL14/EIF3D/RPS18/RPS3/AARS1/FTSJ1/ZC3H15/RPS5/DPH1/RPL4/EIF3H/RPS23/RPL30/DPH7/YTHDF2/RPS3A/RPL12/RPL26L1/RPL18/EIF3E/RPS7/EIF3I/RPL7A/RPS28/NCBP1/RPL9/RACK1/DHX9/EIF4G1/RPL35/RPS19/RPL7/RPL3/SYNCRIP/RPS6/EIF3A/EIF3G/EIF4B/HNRNPU/MTOR/RPLP0/HNRNPD/EIF4H/RPS17/PABPC1/RPLP0P6/YBX[illegible]

GOBP_NUCLEIC_ACID_PHOSPHODIESTER_BOND_HYDROLYSIS	GOBP_NUCLEIC_ACID_PHOSPHODIESTER_BOND_HYDROLYSIS	1.01E-06	CPSF6/DBR1/DXO/ERCC2/PLD6/DIS3L2/RNASET2/SLFN13/CNOT8/EXOSC8/PNKP/KRI1/DNASE1L1/MGME1/CPSF2/USB1/PARN/RAD9A/TSN/PMS2/CSTF3/TATDN3/CNOT6L/CPSF3/DIS3L/LAS1L/DIS3/RIDA/PAN2/KHNYN/CPSF4/TATDN2/PPP1R8/ISG20L2/XRN1/TSEN34/ATAD5/AZGP1/TSEN2/FAN1/POP4/TUT1/ABT1/NCBP1/DNASE2/FIP1L1/BMS1/UTP20/APEX2/EXD3/FANCM/RCL1/CNOT7/EXOSC10/INTS11/SND1/EXOSC2/EXOSC7/APTX/G3BP1/EME1/MUS81/EXOSC5/NUDT21/POP7/ERCC5/TSR1/TDP1/PDE12/NTHL1/REXO4/WRN/NOP9/POLG/RPP25/CNOT6/AGO2/EXOSC9/CSTF2/POLR3K/YBEY/EXOSC4/NUDT12/EDC4/REXO1/DDX1/CNOT1/TOE1/ELAC2/TBL3/RBBP8/CPSF1/DCPS/MRPL44/DCLRE1B/ERI2/NHP2/GEN1/RRS [illegible]

GOBP_SISTER_CHROMATID_COHESION	GOBP_SISTER_CHROMATID_COHESION	1.10E-06	CTCF/SMC5/SMC3/WAPL/PPP2R1A/MACROH2A1/RB1/TENT4A/ESCO1/STAG1/SGO2/SFPQ/MCMBP/NAA50/SLF1/NAA10/DSCC1/RAD21/PDS5A/SMC1A/SGO1/DDX11/BUB1/DDX12P/PHB2/TNKS/AXIN2/BUB1B/CDC20/KIF22/FEN1/ESPL1/HASPIN/CDCA5/PLK1/ESCO2/REC8
GOBP_AMINE_TRANSPORT	GOBP_AMINE_TRANSPORT	1.17E-06	SYT4/CARTPT/VIP/ATP1A2/TACR2/CHRNA3/KCNB1/HTR2A/SLC38A3/RGS4/SLC18A2/CNR1/CXCL12/AGT/CHGA/SV2A/RGS2/PRKN/AVPR1A/RHCG/SYT11/OXTR/SNCG/RAB3B/SLC18A1/GABBR1/SYT1/ADORA3/CHRNB2/P2RY1/SYT5/OXT

GOBP_VASCULAR_PROCESS_IN_CIRCULATORY_SYSTEM	GOBP_VASCULAR_PROCESS_IN_CIRCULATORY_SYSTEM	1.17E-06	AZU1/NPPB/ATP1A2/AGTR1/TACR2/FGA/TACR1/KCNMA1/SLC2A4/KCNMB1/NPR3/KCNA5/SLIT2/SLC8A2/HTR2A/ABCC9/ACTA2/FGG/FERMT2/HTR2B/SLC38A3/ATP1B2/ADRA1D/SLC7A2/SMTNL2/GUCY1A1/KCNJ8/SERPINF2/AGT/LEPR/PDE3A/CD36/PRKG1/VSTM4/ADRA1B/AKAP12/P2RX1/ATP2B4/CAV1/EDNRA/RGS2/PDE2A/NPR1/ANGPT1/ITGA1/ABCB1/SLC8A1/MMP2/COMP/SH3GL2/AVPR1A/CYSLTR1/HBB/SOD3/SLC16A7/ADRB2/OXTR/SLC24A3/FOXC2/SLC29A4/PTP4A3/AVPR2/RAMP2/CXCR2/FGB/CLDN5/TEK/ABCC2/SLC2A3

GOBP_CELL_JUNCTION_ASSEMBLY	GOBP_CELL_JUNCTION_ASSEMBLY	1.16E-06	SFRP1/ACTN2/MYOC/CDH19/EPHA7/GPM6A/NRXN2/NEGR1/ANK2/SORBS1/APOD/FLRT2/GAP43/STON1/DNER/FERMT2/ADGRB3/CDH2/PCDHB5/EPHA3/MAP1B/CLDN6/PHLDB2/MPDZ/GREM1/LGI2/CLDN11/JAM3/NLGN4X/NLGN1/PTPRD/GPM6B/NPTX1/NTN1/ADD2/AGT/CNTNAP1/SDK2/NLGN4Y/PCDHB4/GJC1/THBS1/PCDHB6/CAV1/FGF13/LRRN1/CLSTN2/ITGA5/GPBAR1/CDH11/HOPX/NR1H4/FAM107A/FN1/DKK1/GPC6/SLITRK6/BSN/DLC1/PCDHB2/OXTR/NLGN3/CLDN9/GJA1/SNAI2/LRRC4/DCHS1/COL16A1/CTNND2/EPB41L3/CLDN19/SRPX2/HEG1/RAMP2/CDH6/PCDHB3/SYNDIG1/CLDN5/TEK/COLQ/ACTN1/THY1/MEF2C/DST/NRCAM/LAMC1/NLGN2/CORO2B/IRX3/THBS2/ITGB3/TBX5/CHRNB2/ADGRB1/PCDH17/PKP1/MUSK/MPP[illegible]

GOMF_LIGASE_ACTIVITY	GOMF_LIGASE_ACTIVITY	1.25E-06	GMPS/AARS1/MCCC1/NAE1/WARS2/PARS2/TARS1/CARS2/ATP5F1EP2/HLCS/GARS1/CARS1/DALRD3/HARS1/SARS2/FARSB/RTCB/AACS/ATP5PO/RTCA/PPCS/GSS/LRRC47/ACSF3/NADSYN1/PCCB/ACSS2/DIP2A/ATP5PB/ACSM1/GATB/UBA1/RARS1/NARS1/GART/MARS1/ADSS1/TTLL4/HARS2/ASS1/EPRS1/KARS1/EARS2/PFAS/QARS1/CAD/SLC27A4/CTPS1/IARS1/ATP5F1A/SARS1/UBA7/TARS2/UBA6/FPGS/IARS2/VARS1/ATP5F1B/SLC27A5/ATP5ME/YARS1/NARS2/TRIM25/LARS2/MARS2/MTHFD1L/TTLL6/MCCC2/FARSA/ATP5F1D/ACSL5/NAPRT/LIG1/MTHFD1/ACSM3/PAICS/TTLL12/MDM2/SLC27A2/CARNS1/WARS1/CPS1/QRSL1

GOBP_REGULATION_OF_WOUND_HEALING	GOBP_REGULATION_OF_WOUND_HEALING	1.29E-06	REG3G/APOH/VTN/FGA/MYLK/F2/ACTA2/FGG/ADAMTS18/FERMT2/DDR2/SMOC2/FGF2/PHLDB2/F7/SERPINF2/MMRN1/CD36/PRKG1/THBS1/CAV1/SERPINE2/WFDC1/SERPINE1/EMILIN1/PDGFRA/PROS1/KRT1/FOXC2/CCN4/CLDN19/THBD/FAP/FGB/TAFA5/SERPING1/TFPI
GOBP_POSITIVE_REGULATION_OF_ION_TRANSMEMBRANE_TRANSPORT	GOBP_POSITIVE_REGULATION_OF_ION_TRANSMEMBRANE_TRANSPORT	1.32E-06	PLA2G1B/KCNA1/PLP1/RELN/ACTN2/ADCYAP1R1/CASQ1/KCNK3/JPH2/KCNMB1/ANK2/AKAP6/F2/RGN/DMD/FLNA/ATP1B2/P2RX2/CACNG4/TRPC1/HSPA2/CACNB2/AGT/KCNH2/P2RX1/CAV1/EDNRA/STAC/ABCB1/PKD2/BDKRB1/CACNA2D1/CCL2/ADRB2

GOBP_SYNAPTIC_TRANSMISSION_GLUTAMATERGIC	GOBP_SYNAPTIC_TRANSMISSION_GLUTAMATERGIC	1.37E-06	RELN/ATP1A2/FRRS1L/GRIK3/GRIK1/GRIN2A/HTR2A/GRIK5/CDH2/ROR2/PLPPR4/CACNG4/CNR1/NLGN1/SLC17A7/P2RX1/SERPINE2/PRKN/CCL2/DKK1/OXTR/NLGN3/LRRK2/TSHZ3/MEF2C/NLGN2/SYT1
GOBP_CELL_CYCLE_DNA_REPLICATION	GOBP_CELL_CYCLE_DNA_REPLICATION	1.39E-06	RTEL1/ZPR1/GMNN/POLA1/ATAD5/INO80/UPF1/DONSON/WRN/BRCA2/DBF4/GINS1/FBXO5/TIPIN/MCM6/PCNA/NUGGC/GINS3/BLM/RAD51/MCM3/LIG1/FEN1/DNA2/CDC45/CDC7/MCM4/E2F8/MCM2/E2F7/CDT1

GOBP_NEGATIVE_REGULATION_OF_CANONICAL_WNT_SIGNALING_PATHWAY	GOBP_NEGATIVE_REGULATION_OF_CANONICAL_WNT_SIGNALING_PATHWAY	1.39E-06	SOX10/SFRP1/NKX2-5/DACT3/SFRP2/NOG/GPC3/CDH2/RBMS3/SHISA3/TNN/FRZB/GREM1/SFRP4/IGFBP1/GLI3/BICC1/PRICKLE1/GLI1/CAV1/IGFBP6/DACT1/TBX18/WWTR1/IGFBP4/PRKN/MCC/SHH/DAB2/ANKRD6/DKK1/ISL1/SNAI2/DKKL1/TMEM88/DKK3/TMEM170B/DKK2/SOX17
GOBP_NEGATIVE_REGULATION_OF_NUCLEAR_DIVISION	GOBP_NEGATIVE_REGULATION_OF_NUCLEAR_DIVISION	1.45E-06	MTBP/ZW10/TOM1L1/RAD21/TTK/KNTC1/AURKAIP1/XRCC3/FBXO5/ZWILCH/AURKB/NDC80/BUB1/BUB3/GEN1/SPC25/CENPF/INCENP/CHEK1/SPC24/BUB1B/CDC20/CCNB1/HASPIN/MAD2L1/PLK1/BIRC5/CDCA8/ZWINT/CDT1

GOBP_REGULATION_OF_POSTSYNAPTIC_MEMBRANE_POTENTIAL	GOBP_REGULATION_OF_POSTSYNAPTIC_MEMBRANE_POTENTIAL	1.48E-06	KCNA1/RELN/GRIK3/CHRNA3/GRIK1/GRIN2A/SEZ6/SLC8A2/GRIK5/GLRB/IGSF11/RGS4/P2RX2/NLGN4X/NLGN1/RGS7BP/CHRNB4/P2RX1/DMPK/SLC1A7/INSYN1/ADRB2/MPP2/NLGN3/LRRK2/TMEM108/DLG4/CELF4/KCND2/MEF2C/NLGN2/GABBR1/GABRR1/CHRNB2

GOBP_HOMOPHILIC_CELL_ADHESION_VIA_PLASMA_MEMBRANE_ADHESION_MOLECULES	GOBP_HOMOPHILIC_CELL_ADHESION_VIA_PLASMA_MEMBRANE_ADHESION_MOLECULES	1.49E-06	PCDH10/DSG4/CDH19/FAT3/PCDH9/CADM3/NEXN/IGSF11/CDH2/PCDHB5/HMCN1/PCDH19/CNTN4/VSTM2L/SDK2/CDHR1/DSC3/PCDHB4/TENM3/PCDHB6/PCDHAC2/TRO/PALLD/FAT4/PCDHB18P/L1CAM/CLSTN2/PCDHGA12/PCDHGA2/CDH23/CDH11/PCDHGB7/PCDHGC3/PCDHGB6/PCDHB2/PCDH7/DCHS1/CDH6/PCDHB3/PCDH18/PCDHGA4/PTPRM/PCDH17/PCDHB7/PCDHGB5/ROBO1/PCDHGB1/PECAM1/AMIGO2/IGSF21/PCDHB10/PCDHGA5/PCDHGA9/PCDHB12/CADM1/AMIGO1/PKD1/PCDHB15/SDK1/CLSTN3/OBSL1/CDH5/DSG1/PCDHGA6/DCHS2/PCDHGA7/PCDHB11

GOBP_MITOTIC_DNA_INTEGRITY_CHECKPOINT_SIGNALING	GOBP_MITOTIC_DNA_INTEGRITY_CHECKPOINT_SIGNALING	1.55E-06	CDK2/UIMC1/RPS27L/RPL26/RPA2/CRADD/PPP1R10/RAD9A/CDK5RAP3/NAE1/RAD9B/NEK11/XPC/CHEK2/BRCC3/BRCA1/TOPBP1/PML/EME1/MUS81/DONSON/TRIAP1/CCNG1/TAOK2/RINT1/PRKDC/PIDD1/FZR1/CASP2/RFWD3/CDC14B/NBN/MSH2/TIPIN/GTSE1/CDK1/PLK2/EME2/CHEK1/BLM/TP53/TICRR/CLSPN/FANCD2/DTL/MDM2/PLK1/CCND1/ORC1/MUC1/PBK

GOMF_INTEGRIN_BINDING	GOMF_INTEGRIN_BINDING	1.59E-06	ACTN2/VTN/THBS4/GFRA1/IGF1/ADAMTS8/SFRP2/FERMT2/TNXB/FBLN1/ITGA7/PTN/FGF2/TNN/ADAM23/JAM2/JAM3/IGF2/CXCL12/FBLN5/ITGB1BP2/ITGA8/ITGA9/ITGBL1/THBS1/LGALS12/ECM2/S1PR3/CCN1/ITGA1/FBN1/ITGA5/COMP/COL3A1/EMILIN1/FN1/CCN5/CCN3/CCN4/COL16A1/CCN2/FGF1/TGFBI/FAP/PTPRZ1/FCER2/GPNMB/ACTN1/THY1/DST/ITGB3/ADAMTS5/ITGA11/PLPP3/COL5A1

GOCC_BASEMENT_MEMBRANE	GOCC_BASEMENT_MEMBRANE	1.63E-06	COL2A1/VTN/THBS4/CCDC80/ACTA2/COL4A6/FBLN1/SMOC2/TNC/HMCN1/FREM1/LAMA2/NTN1/COL8A1/ADAMTS1/LAMA1/COL4A5/SERPINF1/FBN1/FN1/COL8A2/EFEMP2/LAMA4/TGFBI/NID1/COLQ/DST/COL4A4/TIMP3/SPARC/LAMC1/COL15A1/THBS2/MMRN2/COL5A1/TIMP1/COL7A1/COL9A1/EGFLAM/COL4A2/AMTN/NTNG2/COL4A3/PXDN/EGFL6/NTN4
GOBP_REGULATION_OF_HEART_RATE	GOBP_REGULATION_OF_HEART_RATE	1.71E-06	SPX/CASQ2/PLN/CTNNA3/POPDC2/ANK2/BVES/KCNA5/DMD/KCNH6/RGS4/CACNA1C/ADRA1D/SCN4B/HRC/CACNB2/KCNE4/AGT/TPM1/KCNH2/ADRA1B/CAV1/EDNRA/SLC8A1/CACNA2D1/AVPR1A/ISL1

GOMF_SERINE_HYDROLASE_ACTIVITY	GOMF_SERINE_HYDROLASE_ACTIVITY	1.78E-06	CTRB1/ELANE/CTRB2/PRTN3/AZU1/CELA2A/HP/RELN/DPP6/PRSS1/CTSG/PCSK1/CMA1/F2/PCSK2/MMP8/F7/MASP1/PRSS2/CTSV/TLL1/MMP3/HGF/ENDOU/MMP2/MMP7/CORIN/MMP19/PRSS23/NAALAD2/C1S/CTSK/CELA3B/C1R/FAP/CPVL/KLK12/TPSG1/F10/TPSAB1
GOBP_DNA_TEMPLATED_DNA_REPLICATION_MAINTENANCE_OF_FIDELITY	GOBP_DNA_TEMPLATED_DNA_REPLICATION_MAINTENANCE_OF_FIDELITY	1.89E-06	BRCA1/FANCM/BARD1/EME1/MUS81/PARP1/MCM9/DONSON/WRN/BRCA2/TRAIP/DDX11/MMS22L/CARM1/ZGRF1/RFWD3/RBBP8/NBN/TIPIN/GEN1/POLE/EME2/ALYREF/CENPX/TIMELESS/PCNA/BLM/RAD51/DNA2/TONSL/POLD1

GOBP_CARDIAC_CONDUCTION	GOBP_CARDIAC_CONDUCTION	1.92E-06	CASQ2/ATP1A2/PLN/NKX2-5/CTNNA3/ANK2/KCNA5/SLC8A2/ABCC9/KCNH6/FLNA/ATP1B2/CACNA1C/TRPC1/SCN4B/HRC/CACNB2/KCNE4/AGT/KCNH2/GJC1/ATP2B4/CAV1/TBX18/SLC8A1/CACNA2D1/CORIN/ISL1/GJA1
GOBP_AMEBOIDAL_TYPE_CELL_MIGRATION	GOBP_AMEBOIDAL_TYPE_CELL_MIGRATION	2.12E-06	SOX10/APOA1/CER1/MIR483/HAND2/APOH/TACR1/FGF10/MEOX2/MIR145/SLIT2/ACTA2/HAS1/TNS1/FGF7/HTR2B/GPC3/CDH2/BMPER/CYP1B1/DDR2/PAK3/PTN/SMOC2/FGF2/GREM1/DAAM2/DCN/SEMA3E/PRKD1/AGT/VSTM4/AKAP12/AKT3/RADIL/ATP2B4/THBS1/SERPINF1/KANK2/LGALS12/EDNRA/ATOH8/FSTL1/CRB2/NODAL/FGFR1/ANGPT1/TGFB2/RHOJ/NRP2/SLC8A1/AMOTL1/MCC/SHH/MIR342/SMO/FN1/CCBE1/MIR27B/ISL1/CCN3/ZEB2/GJA1/SNAI2/FOXC2/SYDE1/SEMA6A/FGF1/ADGRA2/SEMA6D/MIR149/PTP4A3/ILK/HAS2/FAP/SRPX2/SOX17/RHOB/HSPB1/STC1/SCG2/TEK/MEF2C/EGR3/MIR222/TNFSF12/SPARC/MIR10B/ADAMTS9/PTPRM/ANXA6/NANOS1/TWIST1/GPLD1/SEMA6C/ITGB3/MMRN2/SEMA3G/VEGFC[illegible]

GOMF_HORMONE_BINDING	GOMF_HORMONE_BINDING	2.22E-06	ATP1A2/MLNR/ADCYAP1R1/GLP2R/AMHR2/NPR3/GHR/AR/TTR/CCKAR/PTH1R/VIPR2/LEPR/PDE3A/NPR1/SHBG/AVPR1A/SCTR/CCKBR/GLP1R/OXTR/THRB/RAMP2/NPR2/INHBA/ALDH1A3/NR3C1/UCN2/GPER1/ACVR1/EDNRB/PRLR
GOBP_CALCIUM_ION_TRANSMEMBRANE_IMPORT_INTO_CYTOSOL	GOBP_CALCIUM_ION_TRANSMEMBRANE_IMPORT_INTO_CYTOSOL	2.32E-06	PLA2G1B/CASQ2/ATP1A2/PLN/ADCYAP1R1/CASQ1/JPH2/ANK2/GRIN2A/AKAP6/F2/SLC8A2/HTR2A/DMD/RYR3/HTR2B/FLNA/CACNA1C/P2RX2/FGF2/TRPC1/HRC/PRKD1/CCL21/P2RX1/ATP2B4/CCL19/CAV1/SLC8A1/PKD2/BDKRB1/CACNA2D1/CACNA1A/CCN2/PRNP/ITPR1/RYR2/CLIC2/THY1/JPH3/MCOLN3/ITGB3/GPER1/CCR7

GOBP_ENDOTHELIAL_CELL_PROLIFERATION	GOBP_ENDOTHELIAL_CELL_PROLIFERATION	2.35E-06	VIP/APOA1/AGTR1/MIR483/APOH/NGFR/THBS4/FGF10/ALDH1A2/FGF7/CCL26/HTR2B/BMPER/FGF2/IGF2/CXCL12/PRKD1/VSTM4/AKT3/THBS1/CAV1/ATOH8/FGFR1/ANGPT1/NRP2/MIR342/CCL2/MIR27B/CCL11/MIR149/SCG2/TEK/VASH2/MEF2C/EGR3/MIR222/TNFSF12/SPARC/PROK2/MIR10B/PTPRM/ITGB3/MMRN2/VEGFC/SULF1/CD34

GOBP_RNA_EXPORT_FROM_NUCLEUS	GOBP_RNA_EXPORT_FROM_NUCLEUS	2.55E-06	PCID2/NXF1/RBM8A/PHAX/IWS1/YTHDC1/TSC1/MAGOHB/NUP155/AGFG1/NUP107/NXT1/AKAP8L/DDX19B/NUP133/POLDIP3/NUP153/ALKBH5/SMG6/XPO5/NUP214/CPSF6/NCBP3/MAGOH/PABPN1/TPR/SETD2/SMG7/RBM15B/THOC6/SMG1/NUP160/SMG5/SRSF3/CHTOP/NUP62/NUP62CL/POLR2D/NCBP1/KHDRBS1/DHX9/POM121C/NUP85/NSUN2/EIF4A3/NUP93/UPF1/HNRNPA2B1/CASC3/MCM3AP/RAN/POM121/NUP88/HNRNPA1/GLE1/NPM1/THOC3/ALYREF/DDX39A/NOL6/NUP188/POM121B/NXF3
GOCC_MAIN_AXON	GOCC_MAIN_AXON	2.64E-06	UCN3/KCNA1/MYOC/NFASC/TUBB4A/MAP1B/MAP1A/CNTNAP1/MAPT/DLG2/IQCJ-SCHIP1/SPOCK1/KCNQ3/EPB41L3/DLG4/CLDN5/THY1/NRCAM/GABBR1/MAP2/KCNAB1/SCN1B

GOBP_REGULATION_OF_HORMONE_SECRETION	GOBP_REGULATION_OF_HORMONE_SECRETION	2.64E-06	GCG/UCN3/CARTPT/CELA2A/GHRH/AGTR1/TACR2/SFRP1/ADIPOQ/NEUROD1/NNAT/KCNB1/SCG5/FGA/TACR1/ADCYAP1/ADCY5/SCT/KCNA5/FGG/GRP/CCKAR/CNR1/GIP/AGT/CHGA/RFX6/NKX6-1/RBP4/IL6/PRKN/NR1H4/DAB2/SNAP25/GDF9/ISL1/FFAR4/CCN3/RETN/GJA1/GPR27/IL11/KLF7/FGB/NLGN2/INHBA/INHA/BLK/GABBR1/GPLD1/MYRIP

GOBP_TRNA_MODIFICATION	GOBP_TRNA_MODIFICATION	2.76E-06	ALKBH1/ELP2/LCMT2/CDK5RAP1/DUS4L/PUS3/TRMT61A/TRMT10C/THADA/TRMT2B/TRMT5/NSUN6/AARS2/NSUN3/TRMT112/TARBP1/TRMT6/ALKBH8/TYW3/TRIT1/ELP1/MTO1/THUMPD3/THUMPD2/ELP5/TRMO/TRMT61B/HSD17B10/AARS1/FTSJ1/MTFMT/THUMPD1/URM1/DALRD3/TRMT11/QTRT2/METTL2A/METTL2B/TRMT10A/TRMT2A/NSUN2/ELP6/NAT10/TRUB1/CTU1/PUS1/TRMU/WDR6/WDR4/GON7/DTWD2/ELP3/PUS7/PUSL1/CTU2/DUS2/THG1L/ADAT2/QTRT1/RPUSD4/YRDC/TRMT44/DUS1L/TRMT1/C9orf64/GTPBP3/DUS3L

GOBP_OSTEOBLAST_DIFFERENTIATION	GOBP_OSTEOBLAST_DIFFERENTIATION	2.75E-06	PENK/BMP3/SFRP1/HAND2/MYOC/CYP24A1/IGF1/SFRP2/NOG/FERMT2/TWIST2/DDR2/FGF2/TNC/TNN/GREM1/PTH1R/IGF2/LRRC17/GLI3/GDF10/CHRD/PRKD1/RSPO2/GLI1/HGF/CCN1/WWTR1/TMEM119/IL6/HOXA2/SHH/SMO/DHH/NELL1/FFAR4/IGFBP5/WNT10B/ID4/SNAI2/CCN4/PDLIM7/PTHLH/LOX/ILK/BMPR1B/VCAN/MEF2C/MRC2/GABBR1/PTCH1/TWIST1/SCUBE3/VEGFC/CTHRC1/ITGA11/IL6ST/LEF1/COL1A1/ACVR1

GOCC_PRESYNAPSE	GOCC_PRESYNAPSE	2.87E-06	SYT4/UCN3/KCNA1/GHRH/PENK/CHRM2/SLC18A3/GRIK3/SYNGR4/SLC2A4/PCSK1/SCGN/GPM6A/NRXN2/CADM3/GRIN2A/SLC8A2/HTR2A/PTPRN/STON1/GRIK5/NOG/KCNK2/CDH2/NPY/CNTN1/NGF/TNN/ADAM23/SLC18A2/CNR1/SV2B/NLGN4X/DIXDC1/NLGN1/PTPRD/ATP6V1G2/FXYD6/AMPH/CACNB2/SLC17A7/CNTNAP1/NLGN4Y/SYPL2/RGS7BP/SV2A/P2RX1/ATP2B4/GUCY1B1/LIN7A/PDE2A/C1QL1/SLC1A7/SLC8A1/PRRT1/PRKCB/PRKN/SH3GL2/CTTNBP2/SNAP25/LAMP5/SYT11/BSN/NTF3/RAB6B/EFNB3/NLGN3/LRRK2/SNCG/BRSK1/SLC29A4/DLG4/TRIM9/NEFL/RAB3B/PRNP/SEPTIN4/SYNDIG1/NLGN2/SLC18A1/APBA1/RAB3C/GABBR1/SYT1/ADORA3/CHRNB2/PCDH17/SYP/P2RY1/GPER1/ROR1/AP3B2/SYT5/SYNGR1/OXT/[illegible]

GOBP_WOUND_HEALING	GOBP_WOUND_HEALING	2.91E-06	REG3G/CELA2A/APOH/VTN/FGA/MYLK/CTSG/FGF10/SERPIND1/MYL9/F2/ACTA2/IGF1/FGG/SERPINA10/ADAMTS18/NOG/FERMT2/FBLN1/IL24/FLNA/DDR2/SMOC2/FGF2/CSRP1/PHLDB2/TFPI2/GLI3/F7/IL33/F13A1/SERPINF2/FKBP10/MMRN1/TPM1/CD36/PRKG1/TGFB3/DGKG/P2RX1/THBS1/SPRR3/CAV1/S100A8/SELP/CCN1/TGFB2/SERPINE2/ITGA5/COMP/IL6/WFDC1/COL3A1/SERPINE1/SHH/HBB/EMILIN1/PDGFRA/PROS1/FN1/KRT1/F8/SYT11/TSPAN32/MCAM/FZD7/FOXC2/CCN4/FGF1/LOX/CLDN19/THBD/ILK/SCUBE1/FAP/PDPN/HSPB1/FGB/ACTN1/DST/TAFA5/SERPING1/TFPI/BLK/ANXA6/F10/ITGB3/MPIG6B/P2RY1/CXCR4/CD34/IL6ST/COL1A1/PDGFRB/PLPP3/COL5A1/TIMP1/NDNF/MYOZ1/AXL/DCBLD2/ARH[illegible]

GOBP_REGULATION_OF_TELOMERE_MAINTENANCE_VIA_TELOMERE_LENGTHENING	GOBP_REGULATION_OF_TELOMERE_MAINTENANCE_VIA_TELOMERE_LENGTHENING	3.03E-06	MAPKAPK5/SMG6/CTNNB1/TENT4B/CCT6A/PNKP/MAP3K4/FBXO4/ATR/PARN/TERF2/RTEL1/MAP2K7/SMG5/XRCC5/NAF1/XRN1/ACD/STN1/SRC/NAT10/DKC1/HNRNPU/HNRNPC/SLX4/MAPK3/TCP1/EXOSC10/NEK2/PIF1/CCT8/CCT2/HNRNPA2B1/PARP1/CCT3/PINX1/CCT4/CCT7/CCT5/AURKB/HNRNPA1/PRKCQ/TNKS/MAPK15

GOBP_SMOOTH_MUSCLE_CELL_PROLIFERATION	GOBP_SMOOTH_MUSCLE_CELL_PROLIFERATION	3.09E-06	ELANE/VIP/OGN/CNN1/ADIPOQ/TACR1/MYOCD/RBPMS2/MIR145/NPR3/APOD/IGF1/DDR2/FGF2/VIPR2/NR4A3/ELN/SERPINF2/AGT/TPM1/PRKG1/TGFB3/ADAMTS1/THBS1/NPR1/MMP2/IL6/EFEMP2/MIR27B/IGFBP5/CCN3/GJA1/PDGFD/CCN4/PTGIR/S1PR1/MEF2C/MIR222/TAFA5

GOBP_POSITIVE_REGULATION_OF_SECRETION	GOBP_POSITIVE_REGULATION_OF_SECRETION	3.09E-06	PLA2G1B/GCG/SYT4/UCN3/CARTPT/GHRH/VIP/NPB/TACR2/NNAT/KCNB1/FGA/TACR1/PCSK1/ADCYAP1/ANKRD1/SCT/IGF1/FGG/GRP/CNR1/MYOM1/CACNA1H/NLGN1/CXCL12/GIP/CACNB2/AGT/TGFB3/APBB1/RFX6/NKX6-1/S100A8/NPR1/TGFB2/RBP4/MYH10/NR1H4/AVPR1A/DAB2/ISL1/FFAR4/RETN/OXTR/GPR27/FGB/NLGN2/INHBA/INHA/BLK/GABBR1/GPLD1/SYT1/MYRIP/WLS/VEGFC/CHRNB2

GOCC_ION_CHANNEL_COMPLEX	GOCC_ION_CHANNEL_COMPLEX	3.21E-06	KCNA1/CASQ2/SCN7A/GRIK3/DPP6/CHRNA3/KCNB1/KCNMA1/KCNMB1/GRIN2A/AKAP6/KCNA5/ABCC9/RYR3/GRIK5/KCNK2/GLRB/KCNIP1/CACNA1C/TRPC4/CACNG4/TRPC1/BEST2/CACNA1H/SCN4B/KCNJ8/HSPA2/CACNB2/KCNH2/CHRNB4/MICU3/SCN3A/PKD2/CACNA2D3/CACNA2D1/CACNA1A/CLIC4/KCNG1/KCNQ3/KCND1/CNGA1/DLG4/CPT1C/KCNS1/KCND2/RYR2/CLIC2/GABRR1/CHRNB2/CACNG6/GABRE/BEST1/KCNAB1/SCN3B/SCN1B/SCN9A/CLIC5/LRRC26/KCNIP2/KCNS3/PKD1/CNIH2/KCNJ5/CATSPERG/CLCN1/CACNA2D2/PDE4B/KCNH4/KCNIP3/CNIH3/KCNN1/OSTM1/KCNMB3/TRPC6/TTYH2
GOBP_SKELETAL_MUSCLE_ORGAN_DEVELOPMENT	GOBP_SKELETAL_MUSCLE_ORGAN_DEVELOPMENT	3.23E-06	SMYD1/DES/CASQ1/MYOCD/MEOX2/ANKRD1/POPDC2/BVES/KLHL41/DMD/ASB2/CFL2/DNER/RBM24/HLF/SVIL/ITGA7/P2RX2/HOXD10/IGF2/CDON/ACTA1/WT1/ELN/CNTNAP1/RXRG/MYMX/CAV1/LMOD3/HEYL/SHH/SMO/POPDC3/TCF21/WNT10B

GOBP_GLIOGENESIS	GOBP_GLIOGENESIS	3.36E-06	AZU1/NKX2-2/SOX10/PLP1/PENK/RELN/MYOC/VTN/FGF10/ASCL1/F2/GAP43/DNER/NOG/CDH2/ROR2/CNTN1/ATP1B2/PTN/FGF2/TRPC4/DAB1/NDN/DAAM2/GLI3/IL33/NTN1/WASF3/LEPR/LGI4/CNTNAP1/GPR17/MAPT/NKX6-1/S100A8/CSPG4/CX3CR1/LPAR1/MAL/TGFB2/ASPA/SERPINE2/MMP24/KLF15/IL6/S100A9/TSPAN2/COL3A1/SHH/DUSP15/SMO/CCL2/GPR183/ID4/ATOH1/VIM/POU3F1/BMERB1/ILK/TENM4/MXRA8/PTPRZ1/MIR222/CLU/VEGFC/PRDM8/P2RY1/CXCR4/ROR1/IL6ST/IL34/LEF1/LRP1/PLPP3

GOCC_RNA_POLYMERASE_COMPLEX	GOCC_RNA_POLYMERASE_COMPLEX	3.43E-06	GTF2H1/POLR2F/TAF6/ENY2/RPRD2/RTF1/PRIMPOL/ATXN7/POLR2G/TBPL1/POLR3C/CCNH/TAF1/GTF2E1/TBP/MNAT1/RPRD1B/TAF13/TAF4/POLR1H/GTF2B/RPRD1A/CDC73/GTF2A1/ERCC3/CRCP/TAF9/TAF12/TAF2/TADA3/RPAP2/TRRAP/POLR3D/TAF5L/RECQL5/POLR2C/USP22/ERCC2/TENT2/GTF2H2/GTF2H2C/TCEA1/CDK7/POLR2H/CTR9/LEO1/POLR1C/POLR2L/POLR1E/MYO6/POLR2D/MMS19/POLA1/TAF6L/ATXN7L3/POLR3E/PEX2/TAF4B/PRIM2/POLR3H/RPAP1/POLR2E/POLR1A/POLR3B/POLR3A/POLR2B/POLR1B/GTF2H3/GTF2F1/KAT2A/POLR2A/POLR3K/TAF5/POLA2/GTF2E2/GTF2A2/PRIM1/POLRMT/MCM3/TERT/POLR3G

GOBP_CELL_CELL_ADHESION_VIA_PLASMA_MEMBRANE_ADHESION_MOLECULES	GOBP_CELL_CELL_ADHESION_VIA_PLASMA_MEMBRANE_ADHESION_MOLECULES	3.48E-06	APOA1/PCDH10/ADIPOQ/DSG4/MPZ/CDH19/FAT3/PCDH9/CADM3/SPARCL1/NEXN/IGSF11/CDH2/PCDHB5/CLDN6/HMCN1/DAB1/CLDN11/NLGN1/PTPRD/PCDH19/CNTN4/VSTM2L/SDK2/CDHR1/DSC3/PCDHB4/TENM3/PCDHB6/PCDHAC2/TRO/PALLD/FAT4/SELP/CRB2/PCDHB18P/TGFB2/TENM1/L1CAM/CLSTN2/ITGA5/MMP24/PCDHGA12/PCDHGA2/CDH23/CDH11/PCDHGB7/PCDHGC3/PCDHGB6/GPC6/PCDHB2/PCDH7/CLDN9/LRRC4/DCHS1/CLDN19/SELL/TENM4/LRRC4C/CDH6/PCDHB3/PCDH18/PCDHGA4/CLDN5/PTPRM/SCARF2/SELE/PCDH17/PCDHB7/PCDHGB5/ROBO1/PCDHGB1/MYADM/ACVR1/ADGRL3/PECAM1/LGALS7B/AMIGO2/LRRC4B/IGSF21/PCDHB10/PCDHGA5/PCDHGA9/PCDHB12/CADM1/AMIGO1/NTNG2/PKD1/PCDHB15

GOBP_CELL_CELL_SIGNALING_BY_WNT	GOBP_CELL_CELL_SIGNALING_BY_WNT	3.56E-06	SOX10/CER1/SFRP1/MYOC/NKX2-5/FGF10/MIR145/CPE/BARX1/DACT3/RSPO1/SFRP2/PRICKLE2/NOG/FERMT2/GPC3/CDH2/ROR2/RSPO3/RBMS3/PRKAA2/FGF2/SHISA3/TNN/FRZB/GREM1/PYGO1/SFRP4/DAAM2/DIXDC1/IGFBP1/GLI3/TGFB1I1/BICC1/PRICKLE1/WNT9A/RECK/RSPO2/NFATC4/GLI1/CAV1/EDNRA/TCF7L1/NDP/FZD10/MARK1/IGFBP6/TRABD2B/DACT1/TBX18/WWTR1/PKD2/KLF15/IGFBP4/AMOTL1/PRKN/MCC/SHH/APCDD1L/DAB2/ANKRD6/DKK1/GPC6/ISL1/FZD7/WNT2B/LRRK2/WNT10B/ZEB2/SNAI2/CCN4/DKKL1/CTNND2/ADGRA2/TMEM88/DKK3/TMEM170B/ILK/DKK2/SOX17/CMAHP/MIR32/EDA/WLS/RSPO4/CXXC4/HIC1/CTHRC1/NXN/SULF1/ROR1/SHISA2/VANGL2/LEF1/COL1A1/LRP1/P[illegible]
GOBP_ADULT_BEHAVIOR	GOBP_ADULT_BEHAVIOR	3.80E-06	CARTPT/ATP1A2/CHRNA3/NRXN3/NRXN2/SEZ6/HTR2A/TMOD1/GLRB/NPY/DAB1/HOXD10/NLGN4X/CXCL12/GIP/RNF180/LGI4/CHL1/CHRNB4/PRKN/PREX2/SLITRK6/PBX3/EFNB3/UCHL1/NLGN3/SNCG/NLGN2/FGF12/CHRNB2/PCDH17/HOXD9

GOMF_METALLOCARBOXYPEPTIDASE_ACTIVITY	GOMF_METALLOCARBOXYPEPTIDASE_ACTIVITY	3.85E-06	CPA1/CPA2/CPXM2/CPE/CPN1/CPA3/NAALAD2/CPXM1/CPA4/CPA6/AEBP1/VASH2
GOBP_REGULATION_OF_DOUBLE_STRAND_BREAK_REPAIR	GOBP_REGULATION_OF_DOUBLE_STRAND_BREAK_REPAIR	3.92E-06	ING3/ARID2/ARID1A/SLF2/MAGEF1/ACTR2/ERCC6/MGMT/OGG1/YEATS4/UBR5/TRRAP/PPP4R3B/RECQL5/UBE2N/RNF169/SHLD2/SHLD3/TP53BP1/WAS/MAD2L2/SMARCD1/EP400/PNKP/SETD2/RIF1/RPA2/BRD7/BRD8/ATR/MRGBP/CYREN/TFIP11/DEK/SMARCC1/PIAS4/ACTL6A/PARP9/KAT5/NUDT16L1/SPIDR/RTEL1/SMARCC2/FIGNL1/OTUB1/SMCHD1/HDGFL2/MMS19/SMARCB1/FUS/RUVBL1/DTX3L/HSF1/SLF1/PML/RUVBL2/KDM1A/FANCB/PARP1/UBQLN4/SPIRE2/ATRIP/PPP4C/HELB/BCL7A/PRKDC/DDX11/KMT5C/SIRT7/SMARCD2/SIRT6/RBBP8/SMARCE1/WRAP53/RAD51AP1/SMARCA4/FOXM1/PARPBP/CGAS/TIMELESS/CHEK1/OTUB2/PARP3/POLQ/RAD51/AUNIP/NSD2/RMI2

GOBP_AMINO_ACID_ACTIVATION	GOBP_AMINO_ACID_ACTIVATION	4.21E-06	AARS1/WARS2/PARS2/TARS1/CARS2/GARS1/CARS1/DALRD3/HARS1/SARS2/FARSB/LRRC47/GATB/RARS1/NARS1/MARS1/HARS2/EPRS1/KARS1/EARS2/QARS1/IARS1/SARS1/TARS2/IARS2/VARS1/YARS1/NARS2/LARS2/MARS2/FARSA/WARS1/QRSL1
GOMF_TRNA_BINDING	GOMF_TRNA_BINDING	4.23E-06	NSUN6/AARS2/NSUN3/TRNT1/ALKBH8/DARS2/YARS2/XPO5/PSTK/ELP1/FARS2/EIF2S3/SLFN13/THUMPD3/ELP5/EEF1A1/AIMP1/HSD17B10/AARS1/TARS1/CARS1/DALRD3/SARS2/TRMT11/TRMT10A/EEFSEC/NSUN2/NAT10/CTU1/PUS1/TRMU/RARS1/MARS1/KARS1/DTD2/ELP3/EARS2/CTU2/DUS2/THG1L/RPUSD4/IARS1/SARS1/YRDC/MRPS27/EIF2S3B/IARS2/TRMT1/SEPSECS/YARS1/FARSA/TERT

GOMF_EXONUCLEASE_ACTIVITY	GOMF_EXONUCLEASE_ACTIVITY	4.22E-06	DXO/DIS3L2/CNOT8/EXOSC8/MGME1/USB1/PARN/RAD9A/CNOT6L/CPSF3/DIS3L/DIS3/PAN2/ISG20L2/XRN1/FAN1/APEX2/EXD3/CNOT7/EXOSC10/EXOSC2/EXOSC7/APTX/EXOSC5/TDP1/PDE12/REXO4/WRN/POLG/CNOT6/EXOSC9/EXOSC4/REXO1/DDX1/CNOT1/TOE1/DCPS/DCLRE1B/ERI2/POLE/ISG20/POLRMT/ZC3H12A/ERI1/DCLRE1A/EXO1/REXO5/FEN1/AEN/POLD1

GOCC_CATALYTIC_STEP_2_SPLICEOSOME	GOCC_CATALYTIC_STEP_2_SPLICEOSOME	4.18E-06	SNW1/MAGOHB/SNRPD3/ISY1/DHX8/CDC5L/WDR83/RBM22/BCAS2/SNRPG/HNRNPA1L2/RALY/ESS2/CRNKL1/HNRNPA3/PRPF6/MAGOH/SF3B1/PPIL2/SF3A1/PPIL1/LSM3/RBMX/SNRPF/TFIP11/LSM2/SNRPA1/HNRNPH1/MTREX/CDC40/SART1/SF3A3/LSM7/ZCCHC8/DHX38/SNRPB/XAB2/EFTUD2/PRPF19/DDX41/PRPF8/SRRM2/SNRNP40/AQR/HNRNPR/SYNCRIP/EIF4A3/HNRNPK/SNRNP200/SRSF1/SF3B2/HNRNPU/HNRNPC/CACTIN/DDX23/PABPC1/SNRPD1/HNRNPA2B1/SF3A2/SRRM1/HNRNPM/SF3B3/HNRNPF/HNRNPA1/ALYREF/PLRG1

GOCC_PRERIBOSOME	GOCC_PRERIBOSOME	4.48E-06	RIOK3/KRR1/UTP25/MAK16/UTP14A/UTP11/NOP56/NSA2/DCAF13/WDR46/KRI1/HEATR1/NOL10/UTP3/LAS1L/WDR12/FTSJ3/UTP4/XRCC5/RSL1D1/RPS7/IMP3/WDR36/NOP58/RRP7A/MDN1/BMS1/UTP20/NIP7/FBL/RRP1B/TSR1/WDR74/BYSL/EBNA1BP2/NOP9/PRKDC/NOC4L/PES1/WDR3/TBL3/RRP1/PDCD11/IMP4/RRS1/NOB1/RRP9/MRTO4/BOP1/NOL6/PWP2/NOC2L/NOP14/PPAN

GOBP_CALCIUM_ION_TRANSMEMBRANE_TRANSPORT	GOBP_CALCIUM_ION_TRANSMEMBRANE_TRANSPORT	4.59E-06	PLA2G1B/CASQ2/PLP1/ATP1A2/PLN/ADCYAP1R1/CASQ1/JPH2/GPM6A/ANK2/GRIN2A/AKAP6/F2/SLC8A2/HTR2A/RGN/DMD/RYR3/HTR2B/FLNA/CACNA1C/P2RX2/FGF2/TRPC4/CACNG4/TRPC1/CACNA1H/HRC/HSPA2/PRKD1/CACNB2/CCL21/NCS1/P2RX1/ATP2B4/CCL19/TRPM6/MICU3/CAV1/GEM/EDNRA/STAC/HPCA/SLC8A1/PKD2/BDKRB1/CACNA2D3/CACNA2D1/CACNA1A/REM1/SLN/SLC24A3/CCN2/PRNP/ITPR1/RRAD/RYR2/CLIC2/THY1/JPH3/ANXA6/MCOLN3/ITGB3

GOBP_REGULATION_OF_CATION_CHANNEL_ACTIVITY	GOBP_REGULATION_OF_CATION_CHANNEL_ACTIVITY	4.66E-06	KCNA1/CASQ2/RELN/ACTN2/PLN/CASQ1/KCNK3/JPH2/ANK2/AKAP6/ABCC9/DMD/GRP/CACNG4/NLGN1/HRC/CACNB2/KCNE4/CAV1/GEM/EDNRA/FGF13/STAC/HPCA/PKD2/CACNA2D1/PRRT1/KCNG1/CCL2/ADRB2/NLGN3/REM1/STOM/DLG4/KCNS1/RRAD/CLIC2/MEF2C/NLGN2/JPH3/FGF12/RASGRF2
GOBP_DNA_STRAND_ELONGATION	GOBP_DNA_STRAND_ELONGATION	4.73E-06	ACTR5/ACTL6A/RFC3/UCHL5/INO80D/POLA1/RUVBL1/INO80E/INO80/RUVBL2/RFC4/POLE3/MCRS1/NFRKB/INO80C/GINS1/NBN/POLD3/DCLRE1B/MCM7/POLE/POLD2/PCNA/MCM3/LIG1/DNA2/TERT/MCM4

GOBP_ORGAN_GROWTH	GOBP_ORGAN_GROWTH	4.69E-06	CER1/MAEL/NKX2-5/FGF10/AKAP6/IGF1/FGF7/BNC2/AR/NOG/KCNK2/PI16/RGS4/DDR2/ZFPM2/FGF2/NLGN4X/IGF2/WT1/AGT/LEPR/RSPO2/GLI1/EVC/RGS2/TGFB2/RBP4/COMP/ESR1/SHH/SMO/CGA/FOXC2/SORBS2/DUSP9/S1PR1/TENM4/HEG1/STC1/TGFBR3/MEF2C/MIR222/ANXA6/ACACB/TBX5/PDGFRB/HAMP/NDRG4/NPPC/PRLR

GOBP_RIBONUCLEOPROTEIN_COMPLEX_SUBUNIT_ORGANIZATION	GOBP_RIBONUCLEOPROTEIN_COMPLEX_SUBUNIT_ORGANIZATION	4.71E-06	CDC73/KHDC4/DDX46/RPL24/CLP1/DNAJC17/RNU5A-1/USP39/SNRPG/SCAF11/EIF3M/TAF9/EIF6/EIF3K/STRAP/RPL23A/MRPS7/CRNKL1/GEMIN7/FASTKD2/SRSF10/EIF3J/CPSF7/HSP90AB1/LUC7L2/RAMAC/MRPL20/ZNHIT3/CPSF6/SNRPC/PRPF6/BRIX1/RPS27L/EIF2S3/HTATSF1/SF3B1/RPS14/RPL6/RPF2/SHQ1/GEMIN5/ERAL1/DENR/EIF2D/CD2BP2/SF3A1/RPL13A/EIF3B/PRPF31/ATR/ADAR/EIF3D/RBMX/SNRPF/TFIP11/RPS5/LSM2/SNRPA1/EIF3H/HSP90AA1/RNU5B-1/DDX20/MRPS11/SF1/RNU5F-1/XRCC5/PHF5A/NAF1/PRMT7/SART1/EIF3E/SF3A3/POLR2D/EIF3I/SART3/SNRPB/XAB2/RPS28/PIH1D1/PRPF19/ABT1/NCBP1/PRPF8/DHX9/RUVBL1/RPS19/RRP7A/BUD13/[illegible]
GOBP_DNA_UNWINDING_INVOLVED_IN_DNA_REPLICATION	GOBP_DNA_UNWINDING_INVOLVED_IN_DNA_REPLICATION	4.77E-06	WRN/GINS1/GINS2/MCM7/MCM6/TWNK/GINS3/HMGA1/BLM/RAD51/MCM3/CDC45/MCM5/RECQL4/GINS4/MCM4/MCM2

GOCC_ENDOPLASMIC_RETICULUM_PROTEIN_CONTAINING_COMPLEX	GOCC_ENDOPLASMIC_RETICULUM_PROTEIN_CONTAINING_COMPLEX	4.92E-06	EMC7/B2M/SEC11A/DERL2/ORMDL2/ERN1/OSTC/KRTCAP2/UBXN1/RPN2/GPAA1/SPCS1/NPLOC4/SRP9/SPCS3/HSD17B12/AMFR/SPCS2/UFD1/DNAJB11/DNAJC10/PIGU/DPM2/PPIB/RHBDD2/FAF1/DERL1/FAF2/SRPRA/MAGT1/EMC9/SSR4/PDIA3/PIGS/MZB1/RPN1/OS9/HM13/SPTLC1/SELENOS/INSIG1/SDF2L1/PDIA6/EMC1/AUP1/PIGQ/ARL6IP1/SYVN1/MAP3K5/STT3B/TAPBP/CALR/EMC8/GET3/HSPA5/SEC61A1/DERL3/TRAF2/GANAB/SRPRB/STT3A/SREBF2/ZW10/HSP90B1/RINT1/HLA-A/P4HA1/DDOST/VCP/SPTLC2/P4HB/NBAS/PRKCSH/MLEC/ORMDL3/TAP2/HYOU1/EMC6/SPTSSB/ELOVL6/ERN2/TAP1
GOBP_NEGATIVE_REGULATION_OF_METAPHASE_ANAPHASE_TRANSITION_OF_CELL_CYCLE	GOBP_NEGATIVE_REGULATION_OF_METAPHASE_ANAPHASE_TRANSITION_OF_CELL_CYCLE	4.99E-06	ZW10/RAD21/TTK/KNTC1/XRCC3/FBXO5/ZWILCH/AURKB/NDC80/BUB1/BUB3/GEN1/SPC25/CENPF/INCENP/SPC24/BUB1B/CDC20/CCNB1/HASPIN/MAD2L1/PLK1/BIRC5/CDCA8/ZWINT/CDT1

GOBP_HORMONE_TRANSPORT	GOBP_HORMONE_TRANSPORT	5.15E-06	GCG/UCN3/CARTPT/CELA2A/GHRH/VIP/AGTR1/TACR2/SFRP1/ADIPOQ/NEUROD1/NNAT/KCNB1/SCG5/FGA/TACR1/ADCYAP1/ADCY5/CPE/SCT/KCNA5/PTPRN/FGG/GRP/TTR/CCKAR/CNR1/GIP/AGT/CHGA/RFX6/NKX6-1/SELENOM/RBP4/IL6/PRKN/NR1H4/DAB2/CGA/SNAP25/GDF9/ISL1/FFAR4/GLP1R/CCN3/RETN/GJA1/GPR27/IL11/KLF7/FGB/NLGN2/INHBA/INHA/BLK/GABBR1/SLC16A2/GPLD1/MYRIP/SLC16A10/P2RY1

GOBP_CELLULAR_RESPIRATION	GOBP_CELLULAR_RESPIRATION	5.40E-06	PNPT1/NDUFB8/NDUFC2/ETFB/PUM2/OXA1L/IL10RB/CHCHD5/BLOC1S1/OGDH/NDUFB5/DNAJC15/IDH3G/NDUFC1/NDUFA1/DHTKD1/COX7B/SUCLA2/CHCHD2/NDUFB4/UQCRB/ATP5MG/COX6B1/RHOA/NDUFS8/UQCR11/NDUFB2/COA6/ATP5MF/DGUOK/ETFDH/COX7C/ETFA/NDUFA8/ETFRF1/SDHAF4/COX7A2L/ATP5F1C/NDUFA6/NDUFB10/ATP5PD/C2orf69/DLD/FXN/SUCLG2/ACO1/NDUFS5/COX7A2P2/SLC25A23/NDUFB3/NDUFA12/NDUFA5/IREB2/UQCRQ/ABCD1/MRPS36/GPD1/PARK7/BID/NDUFA9/NDUFB6/MDH1/NDUFA11/NDUFAF1/NDUFS6/UQCRFS1/AFG1L/NDUFA10/NDUFS2/ATP5F1EP2/NDUFA2/NDUFS1/COX7A2/NDUFS3/UCN/SDHD/GPD2/DLST/NDUFB7/IDH3B/COX5B/ATP5PO/NDUFAB1/UQCR10/IDE/PDHA1/COX4I1/PRDM16/[illegible]

GOBP_REGULATION_OF_DNA_RECOMBINATION	GOBP_REGULATION_OF_DNA_RECOMBINATION	5.59E-06	ING3/ARID2/MAGEF1/ZRANB3/ACTR2/ERCC6/CD40/RAD50/YEATS4/TRRAP/FOXP3/MSH3/RECQL5/ERCC2/SHLD2/SHLD3/TP53BP1/WAS/MAD2L2/EP400/SETD2/RIF1/RPA2/BRD8/MRGBP/H1-10/TERF2/PIAS4/ACTL6A/KAT5/SPIDR/RTEL1/FIGNL1/SMARCAD1/SMCHD1/HDGFL2/MMS19/ATAD5/TBX21/FUS/MSH6/RUVBL1/RUVBL2/TFRC/H1-2/KDM1A/FANCB/PARP1/UBQLN4/PAXIP1/PPP4C/ZSCAN4/HELB/HMCES/H1-3/KMT5C/IL27RA/SIRT6/RBBP8/MSH2/WRAP53/RAD51AP1/KPNA2/ALYREF/PARPBP/CGAS/TIMELESS/CHEK1/PARP3/BLM/POLQ/RAD51/NSD2/RMI2/H1-4/TNFSF13/H1-5/IL10

GOBP_MITOTIC_METAPHASE_PLATE_CONGRESSION	GOBP_MITOTIC_METAPHASE_PLATE_CONGRESSION	5.76E-06	SPDL1/CHMP6/EML3/CDC23/SEH1L/KPNB1/KAT5/CHMP2B/NUP62/PDCD6IP/RAB11A/CHMP7/CHMP1A/VPS4B/HNRNPU/NUF2/CHMP5/ZW10/PINX1/PSRC1/AURKB/NDC80/KIF14/RRS1/NUDC/KIFC1/INCENP/KIF18A/KIF22/CHMP4C/CCNB1/KIF2C/CENPE/CDCA5/BIRC5/CEP55/CDCA8/CDT1

GOBP_REGULATION_OF_TRANSMEMBRANE_RECEPTOR_PROTEIN_SERINE_THREONINE_KINASE_SIGNALING_PATHWAY	GOBP_REGULATION_OF_TRANSMEMBRANE_RECEPTOR_PROTEIN_SERINE_THREONINE_KINASE_SIGNALING_PATHWAY	5.80E-06	CER1/BMP3/SFRP1/CHRDL1/MYOCD/RBPMS2/CIDEA/FGF10/RGMA/CHRDL2/CILP/SFRP2/GDF6/NOG/GREM2/GPC3/BMPER/GREM1/ASPN/SFRP4/TGFB1I1/GDF10/ZEB1/CHRD/ITGA8/TGFB3/LEFTY2/THBS1/CAV1/LTBP1/ZNF423/FSTL1/CRB2/NODAL/CCN1/DACT1/TGFB2/FBN1/ONECUT1/RBPMS/SHH/MIR342/EMILIN1/DAB2/DKK1/SPART/GDF9/GDF7/CDKN1C/CCN3/ZEB2/IGSF1/DKK3/LOX/ILK/TGFBR3/HSPA1A/INHBA/INHA/LDLRAD4/TET1/SCUBE3/VASN/SULF1/ELAPOR2/TMPRSS6/ACVR1/LRP1

GOBP_CELL_MORPHOGENESIS_INVOLVED_IN_NEURON_DIFFERENTIATION	GOBP_CELL_MORPHOGENESIS_INVOLVED_IN_NEURON_DIFFERENTIATION	5.93E-06	RELN/CHRNA3/NGFR/EPHA7/NRXN3/BOC/SLIT2/FLRT2/GAP43/NEXN/NFASC/CHODL/NOG/GFRA3/ADGRB3/ARHGEF25/CDH2/SLIT3/EPHA3/ZDHHC15/MAP1B/MAP6/CNTN1/NGF/PLPPR4/PAK3/CCKAR/PTN/IGF2BP1/TNN/DAB1/PLXNA4/NDN/MAP1A/DIXDC1/NLGN1/PTPRD/NPTX1/LAMA2/CXCL12/GLI3/ADCY1/SEMA3E/NTN1/CNTN4/VSTM2L/CNTNAP1/CDHR1/CHL1/MAPT/RND2/NFATC4/LAMA1/APBB1/LPAR3/NKX6-1/VLDLR/EDNRA/NCAM1/PALLD/FGF13/L1CAM/NRP2/FEZ1/CDH23/CDH11/HOXA2/SHH/SMO/FN1/PREX2/KIF1A/SLITRK6/SPART/KIF5A/ISL1/SDC2/EFNB3/GDF7/UCHL1/NLGN3/LRRK2/CRABP2/DPYSL5/BRSK1/ATOH1/SEMA6A/CTNND2/SEMA6D/DLG4/NEFL/LRRC4C/BMPR1B/KLF7/ISLR2/PTPRZ1/TH[illegible]

GOBP_NEGATIVE_REGULATION_OF_WNT_SIGNALING_PATHWAY	GOBP_NEGATIVE_REGULATION_OF_WNT_SIGNALING_PATHWAY	5.97E-06	SOX10/CER1/SFRP1/NKX2-5/BARX1/DACT3/SFRP2/NOG/GPC3/CDH2/RBMS3/SHISA3/TNN/FRZB/GREM1/SFRP4/IGFBP1/GLI3/BICC1/PRICKLE1/NFATC4/GLI1/CAV1/IGFBP6/TRABD2B/DACT1/TBX18/WWTR1/IGFBP4/PRKN/MCC/SHH/APCDD1L/DAB2/ANKRD6/DKK1/ISL1/SNAI2/DKKL1/TMEM88/DKK3/TMEM170B/DKK2/SOX17/CXXC4/HIC1/CTHRC1/NXN/SHISA2/LRP1

GOBP_REGULATION_OF_TRANSPORTER_ACTIVITY	GOBP_REGULATION_OF_TRANSPORTER_ACTIVITY	6.17E-06	APOA2/KCNA1/CASQ2/RELN/ATP1A2/ACTN2/ADIPOQ/PLN/CASQ1/KCNK3/JPH2/FHL1/ANK2/AKAP6/ABCC9/RGN/DMD/GRP/ATP1B2/CACNG4/NLGN1/SCN4B/FXYD6/HRC/HSPA2/CACNB2/KCNE4/CAV1/GEM/EDNRA/FGF13/STAC/HPCA/ABCB1/PKD2/CACNA2D1/PRRT1/KCNG1/OSR1/CCL2/ADRB2/NLGN3/REM1/SLN/MIR326/STOM/DLG4/KCNS1/PRNP/RRAD/RYR2/CLIC2/MEF2C/NLGN2/JPH3/FGF12/TWIST1/RASGRF2/ARC/MIR613/KCNAB1/SCN3B/CLTRN/KCTD7/CNKSR3/SCN1B/GSTM2/LRRC26/AMIGO1/CHRM3/KCNIP2/SLC5A11/NEDD4/PM20D1

GOBP_POSITIVE_REGULATION_OF_CYTOSOLIC_CALCIUM_ION_CONCENTRATION	GOBP_POSITIVE_REGULATION_OF_CYTOSOLIC_CALCIUM_ION_CONCENTRATION	6.31E-06	PLA2G1B/TAC1/AGTR1/TACR1/ADCYAP1R1/JPH2/ADCYAP1/ADCY5/HTR2A/RIC3/PTGFR/CACNA1C/ADRA1D/P2RX2/PTGER3/CNR1/CACNB2/AGT/LPAR4/CD36/GPR17/ADRA1B/LPAR3/CAV1/EDNRA/CX3CR1/LPAR1/S1PR3/SLC8A1/PKD2/BDKRB1/CACNA2D1/ESR1/CACNA1A/AVPR1A/CYSLTR1/PDGFRA/CCKBR/FFAR4/GLP1R/OXTR/PTGIR/DLG4/S1PR1/PTGER1/CXCR2/RYR2/FPR1/CXCR1/PROK2/JPH3/P2RY1/CXCR4/GPER1/CCR7/ACKR3/PTGDR/OXT/EDNRB/GPR20/EDN2/FPR2/ACKR2

GOCC_CATION_CHANNEL_COMPLEX	GOCC_CATION_CHANNEL_COMPLEX	6.45E-06	KCNA1/CASQ2/SCN7A/GRIK3/DPP6/KCNB1/KCNMA1/KCNMB1/GRIN2A/AKAP6/KCNA5/ABCC9/RYR3/GRIK5/KCNK2/KCNIP1/CACNA1C/TRPC4/CACNG4/TRPC1/CACNA1H/SCN4B/KCNJ8/HSPA2/CACNB2/KCNH2/MICU3/SCN3A/PKD2/CACNA2D3/CACNA2D1/CACNA1A/KCNG1/KCNQ3/KCND1/CNGA1/DLG4/CPT1C/KCNS1/KCND2/RYR2
GOMF_LIGASE_ACTIVITY_FORMING_CARBON_OXYGEN_BONDS	GOMF_LIGASE_ACTIVITY_FORMING_CARBON_OXYGEN_BONDS	6.50E-06	AARS1/WARS2/PARS2/TARS1/CARS2/GARS1/CARS1/DALRD3/HARS1/SARS2/FARSB/LRRC47/RARS1/NARS1/MARS1/HARS2/EPRS1/KARS1/EARS2/QARS1/IARS1/SARS1/TARS2/IARS2/VARS1/YARS1/NARS2/LARS2/MARS2/FARSA/WARS1

GOBP_REGULATION_OF_MITOTIC_SISTER_CHROMATID_SEGREGATION	GOBP_REGULATION_OF_MITOTIC_SISTER_CHROMATID_SEGREGATION	6.62E-06	HNRNPU/NUF2/ZW10/RAD21/TTK/KNTC1/XRCC3/FBXO5/ZWILCH/AURKB/NDC80/BUB1/BUB3/GEN1/SPC25/PTTG1/CENPF/INCENP/SPC24/BUB1B/CDC20/CCNB1/HASPIN/MAD2L1/PLK1/BIRC5/CDCA8/ZWINT/CDT1
GOBP_REGULATION_OF_CELL_DEVELOPMENT	GOBP_REGULATION_OF_CELL_DEVELOPMENT	6.68E-06	SYT4/NKX2-2/SOX10/APOA1/RELN/ADIPOQ/MYOC/FGA/EPHA7/ASCL1/BNC1/F2/SLIT2/DLL3/IGF1/FGG/CHODL/NOG/FERMT2/LIMS2/MAP1B/FBLN1/MAP6/FLNA/NGF/PAK3/PTN/FGF2/PRTG/FRZB/DAB1/PLXNA4/DAAM2/DIXDC1/PTPRD/CXCL12/GLI3/SEMA3E/IL33/NTN1/PDE3A/RECK/MAPT/RND2/NFATC4/LPAR3/SERPINF1/NKX6-1/CX3CR1/SMARCD3/FGF13/S1PR3/LMOD3/NODAL/ASPA/SERPINE2/L1CAM/FBN1/HEYL/IL6/SHH/SMO/DAB2/FN1/SPART/EFNB3/LRRK2/CRABP2/DPYSL5/ID4/ATOH1/SEMA6A/CCL11/PTHLH/ADGRA2/SEMA6D/NEURL1/NEFL/ILK/HAS2/TENM4/ISLR2/PTPRZ1/FGB/CLDN5/PDE5A/PAEP/THY1/MIR222/NPR2/SEMA6C/ITGB3/SEMA3G/VEGFC/MAP2/SEMA5B/SEMA3D/ROBO1/CXCR[illegible]

GOBP_REGULATION_OF_NEURON_PROJECTION_DEVELOPMENT	GOBP_REGULATION_OF_NEURON_PROJECTION_DEVELOPMENT	6.91E-06	RELN/SFRP1/STMN2/CHRNA3/EPHA7/RGMA/FAT3/NEGR1/SLIT2/SEZ6/SFRP2/CHODL/ADGRB3/DPYSL3/CDH2/EPHA3/ZDHHC15/MAP1B/MAP6/ROR2/CNTN1/FLNA/NGF/PAK3/ATP1B2/PTN/TNN/DAB1/PLXNA4/CNR1/DIXDC1/NLGN1/PTPRD/ALKAL1/CXCL12/SEMA3E/NTN1/PRKD1/AGT/DGKG/MAPT/RND2/NFATC4/NCS1/TENM3/LPAR3/SERPINF1/VLDLR/MARK1/FGF13/RGS2/LPAR1/HGF/NR2F1/L1CAM/FEZ1/S100A9/SPOCK1/DAB2/FN1/SNAP25/PRRX1/DKK1/KIF1A/SPART/SDC2/EFNB3/LRRK2/CRABP2/DPYSL5/DENND5A/BRSK1/SEMA6A/VIM/SEMA6D/NEFL/LRRC4C/ISLR2/CAMK1D/PMP22/THY1/MIR222/NRCAM/EFHC2/SEMA6C/SEMA3G/CHRNB2/MAP2/SEMA5B/SEMA3D/ROBO1/ROR1/ARC/NDNF/SEMA3C/NDRG4/NRP1/CRMP1/SC[illegible]
GOBP_NEUTROPHIL_MEDIATED_IMMUNITY	GOBP_NEUTROPHIL_MEDIATED_IMMUNITY	7.08E-06	PLA2G1B/ELANE/AZU1/CTSG/F2/CXCL6

GOCC_SITE_OF_DOUBLE_STRAND_BREAK	GOCC_SITE_OF_DOUBLE_STRAND_BREAK	7.27E-06	RNF168/RNF169/SHLD2/SHLD3/TP53BP1/WAS/MAD2L2/RPA3/PNKP/RIF1/RPA2/SMC5/DDB1/NABP2/CYREN/CNTD1/RNF138/ZBTB7A/NHEJ1/SMARCAD1/SMCHD1/PRPF19/POLL/POLH/UFL1/LMNA/SLF1/ARPC4/PAXX/PARP1/RAD18/HELB/VCP/SIRT7/RFWD3/SIRT6/RBBP8/NBN/WRAP53/CGAS/TIMELESS/PARP3/TP53/RAD51/H2AX/TONSL/ESCO2

GOBP_IMMUNOGLOBULIN_PRODUCTION	GOBP_IMMUNOGLOBULIN_PRODUCTION	7.24E-06	TRAF2/TFRC/VAMP3/IGLV10-54/PAXIP1/MCM3AP/HLA-DMB/HLA-E/IGKV2-24/HLA-DOB/HLA-DRA/HMCES/PRKDC/TRAF3IP2/HLA-DQA2/UNG/IGKV1-8/KMT5C/HLA-DQA1/TRAV14DV4/IGLV2-11/IGLV2-33/IGLV3-1/IL27RA/NBN/IGKV2D-24/VPREB3/MSH2/HSPD1/HLA-DRB5/PHB2/IGKV2D-30/IGKV6D-41/EPHB2/IGLV3-21/CGAS/HLA-DRB1/IGKV2-40/HLA-DOA/PARP3/GPI/POLQ/IGLV4-3/IGKV3D-11/EXO1/NSD2/TCF3/HLA-DQB1/HLA-DQB2/HLA-DMA/IGLV2-14/IGKV2D-29/IGKV1-12/IGKV1D-13/IGKV1-16/IGLV3-27/IGLV5-45/IGLV5-37/IGLV4-60/IGLV5-48/IGLV2-[illegible]
GOBP_POSITIVE_REGULATION_OF_PHOSPHOLIPASE_ACTIVITY	GOBP_POSITIVE_REGULATION_OF_PHOSPHOLIPASE_ACTIVITY	7.47E-06	PLA2G1B/AGTR1/ADCYAP1R1/HTR2A/PLA2G5/HTR2B/FGF2/AGT/EDNRA/LPAR1/FGFR1/CCN1/NMUR1/HPCA/ESR1/AVPR1A/PDGFRA

GOBP_REGULATION_OF_CANONICAL_WNT_SIGNALING_PATHWAY	GOBP_REGULATION_OF_CANONICAL_WNT_SIGNALING_PATHWAY	7.46E-06	SOX10/SFRP1/NKX2-5/FGF10/MIR145/DACT3/RSPO1/SFRP2/NOG/GPC3/CDH2/RSPO3/RBMS3/FGF2/SHISA3/TNN/FRZB/GREM1/SFRP4/DAAM2/IGFBP1/GLI3/BICC1/PRICKLE1/RECK/RSPO2/GLI1/CAV1/IGFBP6/DACT1/TBX18/WWTR1/IGFBP4/PRKN/MCC/SHH/DAB2/ANKRD6/DKK1/ISL1/FZD7/LRRK2/WNT10B/ZEB2/SNAI2/DKKL1/CTNND2/ADGRA2/TMEM88/DKK3/TMEM170B/ILK/DKK2/SOX17
GOBP_LIPID_DIGESTION	GOBP_LIPID_DIGESTION	7.51E-06	PNLIP/APOA2/CEL/PNLIPRP2/APOA1/SOAT2/CD36/APOA4

GOBP_MITOCHONDRIAL_TRANSPORT	GOBP_MITOCHONDRIAL_TRANSPORT	7.86E-06	PRKAA1/TIMM10/CNP/THEM4/NAIF1/TIMM8A/UBE2L3/USP36/FZD9/TOMM40L/MAPK8/HTRA2/BCL2L11/NMT1/LMAN1/PAM16/TIMM17B/HAX1/OXA1L/DNAJC15/STPG1/SAE1/SLC25A46/IMMP1L/ATG13/TIMM23/CSNK2A2/TIMM23B/HUWE1/FLVCR1/TIMM17A/MUL1/PSEN1/UBE2D3/BLOC1S2/FBXO7/BAP1/RAC2/MAIP1/UCP2/ABCB10/TIMM50/UBE2J2/TOMM70/STARD3/MFN2/TIMM22/BID/TIMM8B/SAMM50/TOMM34/BAD/HSP90AA1/TOMM22/BCL2L1/MTX1/MTX2/CHCHD4/SLC35F6/GSK3A/CPT1A/PMPCB/HSPA4/GRPEL2/HPS4/HEBP2/BCS1L/RTL10/ALKBH7/TIMM29/BAX/TOMM5/SLC25A5/NDUFA13/RHOT2/BAG4/SREBF2/BCAP31/ATP5IF1/ZNF205/SLC25A24/AIFM1/TIMM21/MIPEP/MICALL2/GRPEL1/FZD5/CPT2/TIMM13/HSPD1[illegible]
GOBP_OVULATION_CYCLE	GOBP_OVULATION_CYCLE	8.16E-06	AFP/SLIT2/PGR/PTPRN/SLIT3/CYP1B1/PTN/GDF10/PTX3/TGFB3/ADAMTS1/SERPINF1/TGFB2/MMP2/ESR1/TIMP4/MMP19/PDGFRA/CGA/A2M/GDF9/RETN/OXTR/HAS2/BMPR1B

GOBP_REGULATION_OF_WNT_SIGNALING_PATHWAY	GOBP_REGULATION_OF_WNT_SIGNALING_PATHWAY	8.75E-06	SOX10/CER1/SFRP1/NKX2-5/FGF10/MIR145/BARX1/DACT3/RSPO1/SFRP2/NOG/GPC3/CDH2/RSPO3/RBMS3/FGF2/SHISA3/TNN/FRZB/GREM1/SFRP4/DAAM2/DIXDC1/IGFBP1/GLI3/BICC1/PRICKLE1/RECK/RSPO2/NFATC4/GLI1/CAV1/TCF7L1/IGFBP6/TRABD2B/DACT1/TBX18/WWTR1/KLF15/IGFBP4/PRKN/MCC/SHH/APCDD1L/DAB2/ANKRD6/DKK1/ISL1/FZD7/LRRK2/WNT10B/ZEB2/SNAI2/CCN4/DKKL1/CTNND2/ADGRA2/TMEM88/DKK3/TMEM170B/ILK/DKK2/SOX17/CMAHP/EDA/WLS/RSPO4/CXXC4/HIC1/CTHRC1/NXN/SULF1/SHISA2/LEF1/COL1A1/LRP1/PLPP3/MDFIC
GOBP_DNA_REPLICATION_INITIATION	GOBP_DNA_REPLICATION_INITIATION	8.99E-06	GMNN/POLA1/PRIM2/CDC34/TOPBP1/NOC3L/LRWD1/WRNIP1/POLA2/MCM10/ORC6/NBN/PRIM1/MCM7/MCM6/GINS3/TICRR/MCM3/CDC45/MCM5/ORC5/MCIDAS/MCM4/CCNE2/MCM2/CDT1/ORC1

GOCC_MITOCHONDRIAL_LARGE_RIBOSOMAL_SUBUNIT	GOCC_MITOCHONDRIAL_LARGE_RIBOSOMAL_SUBUNIT	9.48E-06	MRPL24/MRPL57/MRPL48/MRPL9/MRPL40/NSUN3/MRPL27/MRPL45/MRPL36/MRPL32/MRPL20/MRPL17/MRPS30/MRPL14/MRPL30/MRPL3/MRPL18/MRPL22/MRPL21/MRPL52/MRPL13/MRPL19/MRPL35/MRPL42/MRPL43/MRPL34/NSUN4/MRPL28/MRPL47/MRPS18A/MRPL39/MRPL2/MRPL58/MRPL49/MRPL1/MRPL15/MRPL50/MRPL46/GADD45GIP1/MRPL54/MRPL11/MRPL16/MPV17L2/MRPL41/MRPL12/MRPL23/MRPL37/MRPL44/MRPL4
GOMF_SINGLE_STRANDED_DNA_HELICASE_ACTIVITY	GOMF_SINGLE_STRANDED_DNA_HELICASE_ACTIVITY	9.73E-06	DHX9/RFC4/PIF1/MCM9/DSCC1/WRNIP1/HELB/RFC5/RFC2/MCM7/MCM6/CHTF18/POLQ/RAD51/MCM3/DNA2/MCM5/MCM4/MCM2

GOBP_MATURATION_OF_SSU_RRNA	GOBP_MATURATION_OF_SSU_RRNA	9.81E-06	RRP36/RPS21/SRFBP1/LSM6/RIOK1/RIOK2/RPS8/RPS16/RIOK3/UTP25/ERCC2/DDX52/RPS14/DCAF13/WDR46/KRI1/HEATR1/NOL10/TSR3/UTP3/MRPS11/UTP4/IMP3/WDR43/RPS28/ABT1/RPS19/BMS1/UTP20/NAT10/RCL1/NOL11/DHX37/TSR1/BYSL/NOP9/HELB/WDR3/TBL3/IMP4/RRS1/NOB1/PWP2/NOP14

GOBP_POSITIVE_REGULATION_OF_EPITHELIAL_CELL_PROLIFERATION	GOBP_POSITIVE_REGULATION_OF_EPITHELIAL_CELL_PROLIFERATION	9.83E-06	REG3G/VIP/AGTR1/SFRP1/TACR1/THBS4/NKX2-5/FGF10/IGF1/FGF7/PTPRN/EPGN/AR/CCL26/NOG/HTR2B/PTN/FGF2/NR4A3/IGF2/CXCL12/PRKD1/TGM1/AKT3/NODAL/FGFR1/CYP7B1/NRP2/GPBAR1/SHH/SMO/OSR1/MIR27B/FZD7/CCL11/FGF1/HAS2/CRNN/SCG2/TEK/VASH2/EGR3/TNFSF12/LAMC1/MIR10B/TWIST1/ITGB3/VEGFC/ST8SIA1

GOMF_PEPTIDE_RECEPTOR_ACTIVITY	GOMF_PEPTIDE_RECEPTOR_ACTIVITY	9.98E-06	AGTR1/TACR2/MLNR/TACR1/ADCYAP1R1/NPFFR2/GLP2R/GRPR/NPR3/CCKAR/PTH1R/VIPR2/GPR17/EDNRA/CX3CR1/NMUR1/SORCS2/NPR1/BDKRB1/AVPR1A/CYSLTR1/SCTR/CCKBR/GLP1R/OXTR/SSTR2/AVPR2/RAMP2/CXCR2/FPR1/CXCR1/NPR2/CXCR4/CCR7/ACKR3/NPY6R/UTS2R/EDNRB/RAMP3/APLNR/FPR2/ACKR2/RAMP1/F2RL2/GPR37/PTH2R/F2R/F2RL3/OGFRL1/GP1BA
GOBP_REGULATION_OF_CENTROSOME_CYCLE	GOBP_REGULATION_OF_CENTROSOME_CYCLE	1.01E-05	XPO1/ALMS1/CHMP2B/NUP62/CHORDC1/PDCD6IP/CEP295/BRCA1/CHMP1A/PPP1R35/VPS4B/NAT10/NUBP1/CHMP5/FBXW5/RBM14/SASS6/KAT2A/MCPH1/XRCC3/AURKA/GEN1/NPM1/CEP131/WDR62/PLK2/PLK4/CHMP4C/CCNF/STIL/ATF5

GOCC_AZUROPHIL_GRANULE_LUMEN	GOCC_AZUROPHIL_GRANULE_LUMEN	1.01E-05	ELANE/MPO/PRTN3/AZU1/RNASE3/BPI/CTSG/RNASE2/TTR/PRSS2/SERPINA3/ORM2/RETN
GOBP_METAPHASE_ANAPHASE_TRANSITION_OF_CELL_CYCLE	GOBP_METAPHASE_ANAPHASE_TRANSITION_OF_CELL_CYCLE	1.02E-05	CUL3/ANAPC4/MAD2L1BP/ANAPC1/NEK6/TPR/MAD2L2/SMARCD1/BRD7/SPDL1/CDC23/SMARCC1/ACTL6A/RB1/ANAPC15/ANAPC5/SMARCC2/TRIP13/SMARCB1/ANAPC2/NUF2/ZW10/RAD21/BCL7A/TTK/KNTC1/XRCC3/FBXO5/ZWILCH/AURKB/NDC80/BUB1/SMARCD2/BUB3/SMARCE1/GEN1/SPC25/SMARCA4/CENPF/INCENP/SPC24/BUB1B/DLGAP5/CDC20/CCNB1/ESPL1/TACC3/HASPIN/MAPK15/MAD2L1/CENPE/PLK1/BIRC5/CDCA8/ZWINT/CDT1

GOMF_COLLAGEN_BINDING	GOMF_COLLAGEN_BINDING	1.03E-05	VTN/SPARCL1/COL14A1/TNXB/DDR2/PODN/ASPN/DCN/PCOLCE2/THBS1/LUM/ECM2/ITGA1/COMP/FN1/CCBE1/ANTXR1/CTSK/COL6A2/LOX/TGFBI/AEBP1/NID1/PCOLCE/SPARC/MRC2/MUSK/ITGA11
GOBP_POTASSIUM_ION_TRANSPORT	GOBP_POTASSIUM_ION_TRANSPORT	1.06E-05	KCNA1/VIP/CASQ2/ATP1A2/DPP6/ACTN2/KCNB1/KCNMA1/KCNK3/FHL1/KCNMB1/ANK2/AKAP6/KCNA5/HTR2A/ABCC9/KCNK2/KCNH6/GRP/KCNT2/RGS4/KCNIP1/FLNA/ATP1B2/KCNJ8/KCNE4/SLC17A7/KCNH2/CAV1/PKD2/KCNG1/SNAP25/SLC9A9/KCNQ3/KCND1/SLC24A3/KCNS1/PRNP/KCND2
GOBP_INTESTINAL_LIPID_ABSORPTION	GOBP_INTESTINAL_LIPID_ABSORPTION	1.08E-05	PNLIP/APOA2/CEL/APOA1/SOAT2/CD36/APOA4

GOBP_SYNAPSE_ASSEMBLY	GOBP_SYNAPSE_ASSEMBLY	1.09E-05	EPHA7/GPM6A/NRXN2/NEGR1/FLRT2/GAP43/DNER/ADGRB3/CDH2/PCDHB5/MAP1B/LGI2/NLGN4X/NLGN1/PTPRD/NPTX1/NTN1/ADD2/SDK2/NLGN4Y/PCDHB4/PCDHB6/FGF13/LRRN1/CLSTN2/DKK1/GPC6/SLITRK6/BSN/PCDHB2/OXTR/NLGN3/LRRC4/SRPX2/PCDHB3/SYNDIG1/COLQ/MEF2C/NRCAM/NLGN2/THBS2/CHRNB2/ADGRB1/PCDH17/MUSK/ADGRL3/AMIGO2/OXT/LRRC4B/PCDHB10/FZD1/AMIGO1/NTNG2/GHRL/SDK1/CLSTN3/OBSL1/NTRK2/SNCA/TLR2/LRP4
GOBP_DEFENSE_RESPONSE_TO_GRAM_NEGATIVE_BACTERIUM	GOBP_DEFENSE_RESPONSE_TO_GRAM_NEGATIVE_BACTERIUM	1.12E-05	ELANE/AZU1/DEFA4/RNASE3/BPI/CTSG/LYPD8/F2/RNASE2/SSC5D/DEFA6/CXCL6/PRKD1/CHGA/CAMP/SELP/IL6/SERPINE1/DEFB126

GOMF_NUCLEOSOME_BINDING	GOMF_NUCLEOSOME_BINDING	1.12E-05	RNF168/RNF169/MLLT6/SUPT16H/HMGN4/H3-3B/H1-10/ZNHIT1/SMARCC1/MACROH2A1/ACTL6A/EED/H3-3A/SMARCC2/HDAC2/ACTR6/SMARCB1/HMGN1/NAP1L4/MBD3/HIRA/RNF4/HNRNPC/H1-2/HMGN3/PARP1/RBBP4/H1-3/SSRP1/HMGN2/SMARCD2/CABIN1/SIRT6/HDAC1/SMARCE1/MTA2/SMARCA4/HMGN5/H2AZ1/CGAS/NOC2L/RCC1/H1-4/H1-5

GOMF_NUCLEASE_ACTIVITY	GOMF_NUCLEASE_ACTIVITY	1.19E-05	PLD6/DIS3L2/RNASET2/SLFN13/CNOT8/EXOSC8/PNKP/DNASE1L1/MGME1/USB1/PARN/RAD9A/TSN/PMS2/TATDN3/CNOT6L/RPS3/CPSF3/DIS3L/LAS1L/DIS3/RIDA/PAN2/NUDT16L1/KHNYN/TATDN2/PPP1R8/ISG20L2/XRN1/TSEN34/AZGP1/TSEN2/FAN1/POP4/DNASE2/XRCC1/PIWIL4/APEX2/EXD3/FANCM/RCL1/CNOT7/EXOSC10/INTS11/SND1/EXOSC2/EXOSC7/APTX/G3BP1/EME1/MUS81/EXOSC5/POP7/ERCC5/TDP1/PDE12/NTHL1/REXO4/WRN/POLG/RPP25/CNOT6/AGO2/EXOSC9/YBEY/EXOSC4/NUDT12/MAP1S/XRCC3/REXO1/DDX1/CNOT1/TOE1/ELAC2/ZGRF1/RBBP8/DCPS/MRPL44/DCLRE1B/ERI2/GEN1/NOB1/POLE/ISG20/EME2/HELZ2/POLRMT/ZC3H12A/DNASE1L2/ERI1/POP1/DCLRE1A/EXO1/DFFB/REXO5/FEN1/DNA[illegible]
GOBP_ATTACHMENT_OF_SPINDLE_MICROTUBULES_TO_KINETOCHORE	GOBP_ATTACHMENT_OF_SPINDLE_MICROTUBULES_TO_KINETOCHORE	1.22E-05	RB1/SEH1L/KAT5/HNRNPU/NUF2/NEK2/KNSTRN/SGO1/AURKB/NDC80/BUB3/SPAG5/SPC25/INCENP/SPC24/RACGAP1/RCC2/CCNB1/ECT2/KIF2C/CENPE/BIRC5/CDCA8/KNL1/CDT1

GOMF_NUCLEOSOMAL_DNA_BINDING	GOMF_NUCLEOSOMAL_DNA_BINDING	1.26E-05	HMGN4/H3-3B/H1-10/SMARCC1/MACROH2A1/ACTL6A/H3-3A/SMARCC2/HDAC2/SMARCB1/HMGN1/MBD3/HNRNPC/H1-2/HMGN3/RBBP4/H1-3/HMGN2/SMARCD2/HDAC1/SMARCE1/MTA2/SMARCA4/HMGN5/H2AZ1/RCC1/H1-4/H1-5
GOBP_REGULATION_OF_RESPONSE_TO_WOUNDING	GOBP_REGULATION_OF_RESPONSE_TO_WOUNDING	1.27E-05	REG3G/APOH/VTN/FGA/MYLK/RGMA/F2/ACTA2/FGG/ADAMTS18/FERMT2/FLNA/DDR2/PTN/SMOC2/FGF2/PHLDB2/F7/SERPINF2/MMRN1/CD36/PRKG1/THBS1/CAV1/SERPINE2/WFDC1/SERPINE1/EMILIN1/PDGFRA/PROS1/KRT1/FOXC2/CCN4/CLDN19/THBD/FAP/FGB/MIR222/TAFA5/SERPING1/TFPI

GOBP_MEMORY	GOBP_MEMORY	1.30E-05	SYT4/RELN/TACR1/SLC24A4/GRIN2A/SLC8A2/HTR2A/PTCHD1/KCNK2/NGF/PTN/CNR1/MAP1A/ADCY1/GIP/LRRN4/ITGA8/MAPT/NFATC4/SERPINF1/VLDLR/CX3CR1/FGF13/ITGA5/SYT11/NTF3/OXTR/PRNP/CHST10/S100B/JPH3/CHRNB2/MUSK/ARC
GOCC_COMPLEX_OF_COLLAGEN_TRIMERS	GOCC_COMPLEX_OF_COLLAGEN_TRIMERS	1.30E-05	COL2A1/COL4A6/COL11A2/COL8A1/COL4A5/LUM/COL3A1/COL16A1/COL1A2/COL4A4/COL10A1/COL1A1/COL5A1/COL7A1/COL5A2/COL4A2/COL4A3

GOBP_NEGATIVE_REGULATION_OF_MITOTIC_CELL_CYCLE	GOBP_NEGATIVE_REGULATION_OF_MITOTIC_CELL_CYCLE	1.31E-05	BRCC3/BRCA1/BTN2A2/PTPN3/TOPBP1/PML/NUF2/MIIP/BARD1/EME1/MUS81/CDK2AP2/TRIM35/YTHDC2/DONSON/MTBP/ZFYVE19/ZW10/SLC9A3R1/PINX1/TOM1L1/TRIAP1/RAD21/CCNG1/KLF4/TAOK2/RINT1/TTK/PRKDC/KNTC1/PIDD1/AURKAIP1/XRCC3/FBXO5/FZR1/CASP2/ZWILCH/AURKB/NDC80/BUB1/NLE1/BUB3/RFWD3/RBBP8/CDC14B/NBN/MSH2/TIPIN/GEN1/EZH2/GTSE1/SPC25/CDK1/PLK2/EME2/SCRIB/CENPF/INCENP/CHEK1/SPC24/BUB1B/BLM/CDC20/TP53/TICRR/CLSPN/CHMP4C/FANCD2/CCNB1/HASPIN/MAD2L1/DTL/MDM2/PLK1/BIRC5/GFI1B/CDCA8/CCND1/FOXC1/ZWINT/E2F7/CDT1/ORC1/MUC1/PBK/IL10
GOMF_EXTRACELLULAR_MATRIX_STRUCTURAL_CONSTITUENT_CONFERRING_TENSILE_STRENGTH	GOMF_EXTRACELLULAR_MATRIX_STRUCTURAL_CONSTITUENT_CONFERRING_TENSILE_STRENGTH	1.39E-05	COL2A1/COL4A6/COL14A1/COL21A1/COL11A2/COL8A1/COL4A5/COL24A1/COL3A1/COL8A2/COL23A1/COL16A1/COL6A2/COL6A3/COL1A2/COL4A4/COL15A1/COL10A1/COL1A1/COL5A1/COL7A1/COL9A1/COL12A1/COL5A2/COL4A2

GOBP_HORMONE_METABOLIC_PROCESS	GOBP_HORMONE_METABOLIC_PROCESS	1.38E-05	PNLIP/AFP/CEL/BCHE/SCG5/CTSG/PCSK1/CMA1/ADH1B/ALDH1A2/SULT2A1/CPE/GHR/TTR/CYP1B1/UGT2B17/PCSK2/CACNA1H/DIO1/RDH8/CYP11A1/ADH1A/ADH7/SELENOM/CPA3/UGT1A7/RBP4/ESR1/CORIN/SHH/PDGFRA/DAB2/CGA/HSD17B6/TPO/DIO2/CTSK/CRABP2/PCSK1N/PDE8B/DKK3/ENPEP/CHST9/CHST10/LRAT/ALDH1A3/NR3C1/SLC16A2/CYP3A4/CPQ/CYP1A1/SLC16A10/RBP1/MBOAT4/ADH4

GOMF_CATION_CHANNEL_ACTIVITY	GOMF_CATION_CHANNEL_ACTIVITY	1.39E-05	KCNA1/SCN7A/GRIK3/CHRNA3/KCNB1/KCNMA1/KCNK3/GRIK1/GPM6A/KCNMB1/GRIN2A/KCNA5/ABCC9/RYR3/GRIK5/KCNK2/KCNH6/KCNT2/KCNIP1/CACNA1C/P2RX2/TRPC4/CACNG4/TMEM63C/CNR1/TRPC1/CACNA1H/SCN4B/KCNJ8/CACNB2/KCNE4/KCNH2/CHRNB4/NCS1/P2RX1/TRPM6/SCN3A/PKD2/CACNA2D3/CACNA2D1/CACNA1A/KCNG1/SNAP25/RHCG/PIEZO2/KCNQ3/KCND1/SLC24A3/RHBG/CNGA1/CALHM5/KCNS1/KCND2/ITPR1/RYR2

GOBP_CELL_SUBSTRATE_ADHESION	GOBP_CELL_SUBSTRATE_ADHESION	1.37E-05	APOA1/SFRP1/ACTN2/MYOC/VTN/FGA/SORBS1/BVES/APOD/CCDC80/FGG/ABI3BP/STON1/FERMT2/TNXB/LIMS2/EPHA3/FBLN1/NPY/FLNA/ATP1B2/ITGA7/PHLDB2/TNN/GREM1/VIT/JAM3/COL26A1/LYVE1/GPM6B/FREM1/SEMA3E/FBLN5/COL8A1/CD36/ITGA8/CCL21/ITGA9/FOXF1/ITGBL1/RADIL/THBS1/ECM2/CCN1/ANGPT1/ITGA1/L1CAM/ITGA5/ONECUT1/COL3A1/SERPINE1/FAM107A/SPOCK1/EMILIN1/DAB2/FN1/EFEMP2/FZD7/SGCE/ANTXR1/DLC1/FBLN2/PARVA/COL16A1/CCN2/BCAM/ILK/HAS2/BST1/PDPN/FGB/NID1/TEK/ACTN1/THY1/LAMC1/ADAMTS9/EDA/CORO2B/ITGB3/SNED1/EPDR1/CCR7/CD34/ITGA11/MYADM/COL1A1/NDNF/AXL/NRP1/FZD4/MERTK/RRAS/EDIL3/EGFLAM/ANGPT2/CDKN2A/NTNG2/PKD1/DU[illegible]
GOBP_INTERSTRAND_CROSS_LINK_REPAIR	GOBP_INTERSTRAND_CROSS_LINK_REPAIR	1.43E-05	FANCF/RNF168/MCM8/CENPS/ATR/FAAP100/HROB/FAN1/FANCM/SLX4/FANCB/FANCL/MCM9/FANCC/FANCG/VCP/XRCC3/RFWD3/DCLRE1B/FANCE/RAD51AP1/NEIL3/CENPX/DCLRE1A/RAD51/FANCI/FANCD2/FANCA

GOBP_REPLICATION_FORK_PROCESSING	GOBP_REPLICATION_FORK_PROCESSING	1.45E-05	CENPS/ATR/RTEL1/FAM111A/BRCA1/FANCM/BARD1/EME1/MUS81/PARP1/WRN/BRCA2/TRAIP/DDX11/MMS22L/CARM1/ZGRF1/RFWD3/RBBP8/NBN/GEN1/EME2/ALYREF/CENPX/PCNA/BLM/RAD51/DNA2/TONSL
GOMF_SINGLE_STRANDED_DNA_BINDING	GOMF_SINGLE_STRANDED_DNA_BINDING	1.52E-05	RPA2/SMC5/TOP1/POLR2H/RAD23B/NABP2/TSN/PMS2/TERF2/PARK7/WBP11/RNF138/NUP35/SWSAP1/XPC/POLA1/FAM111A/TOP3A/HROB/FUBP1/RPA1/STN1/DHX9/HSF1/SSBP4/HNRNPU/APTX/JCHAIN/HNRNPA2B1/YBX1/ERCC5/MCM9/TDP1/REXO4/BRCA2/RAD18/SMC4/LRPPRC/HMCES/RAD23A/HMGB2/DDX11/MMS22L/MCM10/RBBP8/PCBP1/MSH2/HNRNPA1/HSPD1/RAD51AP1/MCM7/DMC1/NEIL3/MCM6/SMC2/TWNK/LONP1/BLM/TP53/RAD51/MCM3/CDC45/MCM5/RECQL4/RAD54L/MCM4/IGHM/MCM2

GOBP_REGULATION_OF_NERVOUS_SYSTEM_PROCESS	GOBP_REGULATION_OF_NERVOUS_SYSTEM_PROCESS	1.52E-05	SPX/CARTPT/VIP/SOX10/TAFA4/RELN/GRIN2A/TMEM100/SLC8A2/NPTX2/IGSF11/RGS4/CACNG4/JAM2/NLGN4X/NLGN1/NPTX1/IL33/WASF3/AGT/NPTXR/CHRNB4/DMPK/HGF/AVPR1A/ADRB2/CCN3/NLGN3/LRRK2/TMEM108/DLG4/CELF4/TENM4
GOBP_LEARNING	GOBP_LEARNING	1.52E-05	BCHE/RELN/ATP1A2/TACR2/TACR1/NRXN3/NRXN2/GRIN2A/SLC8A2/NPTX2/NOG/ADGRB3/PTN/MAP1A/NLGN4X/LRRN4/AGT/NLGN4Y/PRKAR2B/FGF13/C1QL1/PDE1B/PRKN/SNAP25/DKK1/SYT11/NLGN3/BRSK1/PDE8B/DLG4/CHST10/CLDN5/JPH3/CHRNB2/ARC/KIT

GOBP_REGULATION_OF_ACTIN_FILAMENT_BASED_PROCESS	GOBP_REGULATION_OF_ACTIN_FILAMENT_BASED_PROCESS	1.61E-05	APOA1/ATP1A2/SYNPO2/SFRP1/ACTN2/MYOC/LMOD1/PLN/TACR1/CTNNA3/ANK2/SLIT2/ACTA2/TMOD1/CFL2/CCL26/FERMT2/EPHA3/RGS4/FLNA/PAK3/SVIL/CACNA1C/PHLDB2/JAM3/DAAM2/DIXDC1/GPM6B/CXCL12/ELN/SEMA3E/ADD2/SERPINF2/WASF3/TPM1/CDC42EP3/CCL21/TGFB3/PPM1E/RND2/KANK4/CSF3/CAV1/CGNL1/FZD10/FGF13/LPAR1/LMOD3/TENM1/PRKN/FRMD6/FAM107A/FHOD3/PDGFRA/NTF3/DLC1/KIRREL1/CCN2/SYNPO/CCL11/ARHGAP28/MIR149/S1PR1/BST1/RHOB/PLEKHH2/PDPN/RYR2/STC1/TEK/TMSB15A/MTSS1/WASF1/CORO2B/ITGB3/CYRIA/MAGEL2/DSTN/CCR7/RHOQ/MYADM/PDGFRB/LRP1/ARHGEF15/GSN/NRP1/RDX/ARHGAP40/PFN2
GOCC_COSTAMERE	GOCC_COSTAMERE	1.63E-05	SYNM/PGM5/FLNC/ANK2/DMD/SMPX/SVIL

GOBP_POSITIVE_REGULATION_OF_ERK1_AND_ERK2_CASCADE	GOBP_POSITIVE_REGULATION_OF_ERK1_AND_ERK2_CASCADE	1.65E-05	GCG/CCL16/HAND2/FGA/FGF10/ADCYAP1/HTR2A/ACTA2/IGF1/FGG/PLA2G5/CCL26/FERMT2/HTR2B/NPY/ROR2/BMPER/DDR2/FGF2/ALKAL1/THPO/TNFAIP8L3/SERPINF2/CD36/CCL21/AKAP12/CCL19/NODAL/ANGPT1/GPBAR1/PDGFRA/CCL2/MIR27B/GPR183/FFAR4/PDGFD/CCN2/CCL11/FGF1/FGB/GPNMB/NOX4/TEK/MIR222/INHBA/PDGFC/ITGB3/SLC30A10/P2RY1/GPER1/CCR7/ROR1/ACKR3/PDGFRB/NDRG4/NRP1/MAP3K12/RAMP3
GOBP_POSITIVE_REGULATION_OF_BLOOD_PRESSURE	GOBP_POSITIVE_REGULATION_OF_BLOOD_PRESSURE	1.66E-05	SPX/CARTPT/TACR1/ADRA1D/TAC3/CNR1/AGT/TPM1/ADRA1B/AVPR1A/GLP1R/OXTR/AVPR2

GOBP_ADENYLATE_CYCLASE_ACTIVATING_G_PROTEIN_COUPLED_RECEPTOR_SIGNALING_PATHWAY	GOBP_ADENYLATE_CYCLASE_ACTIVATING_G_PROTEIN_COUPLED_RECEPTOR_SIGNALING_PATHWAY	1.69E-05	GCG/UCN3/GHRH/VIP/PLN/ADCYAP1/ADCY5/ADGRB3/ADCY2/PTGFR/ADGRD1/ADRA1D/PTGER3/CNR1/PTH1R/ADCY1/GIP/CHGA/ADRA1B/ATP2B4/LPAR3/CNR2/LPAR1/S1PR3/GNAL/ADRB2/GLP1R/PTHLH/PTGIR/S1PR1/GPR161/PTGER1/ABCA1/RAMP2/UCN2/ADGRB2/ADGRB1/GPER1/ADGRG2/S1PR5/ADGRL3/ADGRL2/RAMP3/ADGRL4/RAMP1/TBXA2R/ADCY4
GOBP_CENTROMERE_COMPLEX_ASSEMBLY	GOBP_CENTROMERE_COMPLEX_ASSEMBLY	1.71E-05	H3-3B/CENPS/RB1/H3-3A/ITGB3BP/RNF4/MIS18A/NASP/CENPW/CENPI/HJURP/KNTC1/CENPO/SIRT6/CENPA/CENPX/CENPK/CENPF/CENPV/DLGAP5/CENPN/OIP5/CENPE/HELLS/CENPH

GOBP_ESTABLISHMENT_OF_RNA_LOCALIZATION	GOBP_ESTABLISHMENT_OF_RNA_LOCALIZATION	1.73E-05	SIDT2/PNPT1/NXT1/AKAP8L/DDX19B/HNRNPA1L2/NUP133/FMR1/RANBP2/SENP2/POLDIP3/G3BP2/NUP153/ALKBH5/SMG6/CETN3/XPO5/NUP98/NUP214/CPSF6/NCBP3/MAGOH/PABPN1/TPR/SRSF7/SETD2/AAAS/SMG7/NUP54/NUTF2/ATR/RBM15B/THOC6/AHCTF1/IGF2BP2/MRPL18/SMG1/XPO1/NUP160/SEH1L/KPNB1/SMG5/SRSF3/NUP35/CHTOP/NUP62/NUP62CL/POLR2D/NCBP1/KHDRBS1/DHX9/POM121C/NUP85/NSUN2/NUP43/EIF4A3/SRSF1/HSF1/NUP58/NUP93/UPF1/HNRNPA2B1/CASC3/YBX1/MCM3AP/RAN/POM121/CKAP5/ZC3H3/NUP205/LRPPRC/NUP37/NUP88/SEC13/NUP50/KHSRP/HNRNPA1/MVP/GLE1/TNKS/SLBP/NPM1/THOC3/ALYREF/DDX39A/NOL6/TST/NDC1/NUP188/POM121B/EIF5AL1/NXF3

GOBP_REGULATION_OF_CELL_JUNCTION_ASSEMBLY	GOBP_REGULATION_OF_CELL_JUNCTION_ASSEMBLY	1.75E-05	SFRP1/MYOC/EPHA7/NEGR1/APOD/FLRT2/FERMT2/ADGRB3/EPHA3/PHLDB2/GREM1/NLGN1/PTPRD/GPM6B/NPTX1/NTN1/AGT/THBS1/CAV1/LRRN1/CLSTN2/GPBAR1/HOPX/FAM107A/DKK1/GPC6/SLITRK6/DLC1/OXTR/NLGN3/SNAI2/COL16A1/CLDN19/SRPX2/SYNDIG1/CLDN5/TEK/COLQ/THY1/MEF2C/NLGN2/IRX3/THBS2/TBX5/CHRNB2/ADGRB1/MUSK/AMIGO2/OXT/NRP1/LRRC4B/APLNR/FZD1/AMIGO1/NPHP1/NTNG2/DUSP3/SNAI1/GHRL/TLN1/LIMCH1/CLSTN3/NTRK2/SNCA/TLR2

GOBP_REGULATION_OF_OSTEOBLAST_DIFFERENTIATION	GOBP_REGULATION_OF_OSTEOBLAST_DIFFERENTIATION	1.84E-05	SFRP1/HAND2/IGF1/SFRP2/NOG/FERMT2/TWIST2/DDR2/FGF2/TNN/GREM1/GLI3/GDF10/CHRD/PRKD1/GLI1/HGF/CCN1/WWTR1/TMEM119/IL6/HOXA2/NELL1/FFAR4/IGFBP5/WNT10B/SNAI2/CCN4/PDLIM7/ILK/BMPR1B/MEF2C/PTCH1/TWIST1/SCUBE3/VEGFC/CTHRC1/IL6ST/ACVR1/NPPC/FGFR2/FAM20C/SOX11/BMP6

GOBP_REGULATION_OF_MUSCLE_CONTRACTION	GOBP_REGULATION_OF_MUSCLE_CONTRACTION	1.84E-05	SPX/KCNA1/CASQ2/CHRM2/CNN1/ATP1A2/TACR2/CHRNA3/PLN/TACR1/KCNMA1/CASQ1/MYOCD/NKX2-5/MYL9/CTNNA3/MIR145/ANK2/PPP1R12B/DMD/MYL3/CACNA1C/GUCY1A1/HRC/TPM1/PRKG1/CHGA/ADRA1B/CHRNB4/P2RX1/DMPK/CAV1/FGF13/RGS2/SLC8A1/ADRB2/OXTR/CCN2/SSTR2/RYR2/STC1/CLIC2/PDE5A/PROK2

GOMF_PEPTIDASE_ACTIVITY	GOMF_PEPTIDASE_ACTIVITY	1.86E-05	CTRB1/ELANE/CTRB2/PRTN3/CPA1/CPA2/AZU1/CELA2A/HP/RELN/SFRP1/DPP6/PRSS1/CTSG/PCSK1/CMA1/ADAM33/CPXM2/F2/CPE/PAPPA2/ADAMTS8/ADAMTS18/ADAMTS16/CLC/ADAMTSL3/ADAM23/PCSK2/KY/MMP8/CPN1/DPEP3/F7/MASP1/PRSS2/PIP/CTSV/ADAMTS1/CAPN11/TLL1/USP2/CPA3/MMP3/HGF/TRABD2B/ENDOU/FGL2/CLCA1/ADAMTS3/USP51/XPNPEP2/MMP24/MMP2/CAPN6/ADAMTSL1/GGT5/MMP7/CORIN/MMP19/SHH/PRSS23/CTSF/DHH/NAALAD2/MMP16/CPXM1/TRHDE/C1S/CTSK/UCHL1/DPEP1/ADAMTS10/MMP23B/CELA3B/CPA4/PAPLN/C1R/CPA6/ENPEP/FAP/CPVL/AEBP1/CAPNS2/VASH2/PAPPA/ADAMTS9/KLK12/TPSG1/F10/TPSAB1/CPQ
GOCC_NUCLEOID	GOCC_NUCLEOID	1.88E-05	TFB2M/FASTKD5/HADHA/HSD17B10/TFAM/CLPX/SLC25A5/DDX28/SUPV3L1/MPG/LRRC59/ACADVL/POLG/LRPPRC/TFB1M/VDAC1/HSPA9/DHX30/TOP1MT/ELAC2/ATP5F1B/GRSF1/TWNK/TUFM/POLRMT/LONP1/VDAC2/DNA2/TERT/ATAD3A/SHMT2/CPS1

GOBP_CANONICAL_WNT_SIGNALING_PATHWAY	GOBP_CANONICAL_WNT_SIGNALING_PATHWAY	1.90E-05	SOX10/SFRP1/NKX2-5/FGF10/MIR145/DACT3/RSPO1/SFRP2/NOG/GPC3/CDH2/RSPO3/RBMS3/FGF2/SHISA3/TNN/FRZB/GREM1/PYGO1/SFRP4/DAAM2/DIXDC1/IGFBP1/GLI3/BICC1/PRICKLE1/WNT9A/RECK/RSPO2/GLI1/CAV1/EDNRA/TCF7L1/FZD10/IGFBP6/DACT1/TBX18/WWTR1/IGFBP4/PRKN/MCC/SHH/DAB2/ANKRD6/DKK1/ISL1/FZD7/WNT2B/LRRK2/WNT10B/ZEB2/SNAI2/DKKL1/CTNND2/ADGRA2/TMEM88/DKK3/TMEM170B/ILK/DKK2/SOX17

GOMF_NUCLEOTIDYLTRANSFERASE_ACTIVITY	GOMF_NUCLEOTIDYLTRANSFERASE_ACTIVITY	1.93E-05	ZCCHC7/POLR2C/SELENOO/NUDT5/TENT4B/FLAD1/MTPAP/TENT2/OAS1/FPGT/RNGTT/TERF2/TAMM41/TUT7/POLR1C/POLR2L/TENT4A/OAS2/CMAS/COASY/POLA1/TUT1/POLL/POLH/POLR3H/RPAP1/POLM/POLE2/POLR2E/POLB/TENT5A/POLR1A/DKC1/POLR3B/POLR3A/CTU1/POLR2B/PTGES3/POLE3/NMNAT1/POLR1B/KARS1/POLG/GMPPA/POLR2A/CTU2/THG1L/POLR3K/PAPSS1/OAS3/YRDC/PCYT2/GPHN/POLD3/PRIM1/GMPPB/POLE/POLRMT/OASL/CGAS/POLQ/CDS1/TERT/NANS/POLD1/POLR3G

GOMF_GATED_CHANNEL_ACTIVITY	GOMF_GATED_CHANNEL_ACTIVITY	1.97E-05	KCNA1/SCN7A/GRIK3/CHRNA3/KCNB1/KCNMA1/KCNK3/GRIK1/KCNMB1/GRIN2A/KCNA5/ABCC9/RYR3/GRIK5/KCNK2/KCNH6/GLRB/KCNT2/KCNIP1/CACNA1C/P2RX2/CACNG4/TMEM63C/CNR1/CACNA1H/SCN4B/KCNJ8/CACNB2/KCNE4/KCNH2/CHRNB4/NCS1/P2RX1/SCN3A/CLCA1/SLC1A7/ANO5/PKD2/CACNA2D3/CACNA2D1/CACNA1A/CLIC4/TMEM150C/KCNG1/SNAP25/PIEZO2/KCNQ3/KCND1/CNGA1/KCNS1/KCND2/ITPR1/RYR2/CLIC2/ANXA6/MCOLN3/GABRR1/CHRNB2

GOMF_RIBOSOME_BINDING	GOMF_RIBOSOME_BINDING	1.97E-05	DNAJC2/BAG6/TMEM147/MAIP1/GEMIN5/NAA15/SRP72/NOMO3/NOMO1/SRP68/RACK1/LETMD1/TACO1/CCDC47/HSPA5/MTOR/NAA10/SEC61A1/PRMT3/EIF2S1/EEF2/SERBP1/GCN1/OLA1/PYM1/RPSA/MRPS27/NOMO2/GUF1/ZNF598/ZC3H12A/EIF3C/ERI1/C1QBP/EIF5A/NCLN/LETM1/EIF5AL1/EFL1

GOBP_MESENCHYMAL_CELL_DIFFERENTIATION	GOBP_MESENCHYMAL_CELL_DIFFERENTIATION	1.98E-05	SOX10/SFRP1/HAND2/FGF10/ALDH1A2/TMEM100/DACT3/IGF1/SIX2/SFRP2/NOG/FERMT2/HTR2B/CDH2/EPHA3/FLNA/PHLDB2/FRZB/GREM1/TGFB1I1/SEMA3E/TGFB3/RADIL/EDNRA/MARK1/CRB2/HGF/FGFR1/TGFB2/WWTR1/NRP2/HEYL/IL6/SHH/MIR342/ZNF750/SMO/OSR1/DAB2/FN1/FOXF2/ISL1/FAM83D/TCF21/SNAI2/FOXC2/SEMA6A/SEMA6D/MIR149/HAS2/PDPN/PHLDB1/TGFBR3/MEF2C/MIR222/LDLRAD4/ANXA6/TWIST1/SEMA6C/TBX5/SEMA3G/VASN/SEMA5B/SEMA3D/LEF1/COL1A1/ACVR1/SEMA3C/EDNRB/NRP1/SPRED3/BMP5/FGFR2/SOX11/FUZ/PITX2/ENG/SNAI1/SEMA6B/GLIPR2/SERPINB3/RFLNB/SPRY1/SEMA5A

GOMF_CYTOKINE_BINDING	GOMF_CYTOKINE_BINDING	2.01E-05	ELANE/CER1/NLRP7/CHRDL1/CHRDL2/GHR/NOG/GREM2/FGF2/ACKR1/GREM1/CHRD/LEPR/CD36/TGFB3/THBS1/LTBP1/IL1R1/CX3CR1/NRP2/COMP/A2M/CRLF1/BMPR1B/LRRC32/CXCR2/PDPN/TGFBR3/LTBP3/CXCR1/ITGB3/SCUBE3/IL13RA2/VASN/CXCR4/IL20RB/LIFR/CCR7/ACKR3/IL6ST/KIT/ACVR1/NRP1/FZD4/PRLR/CD109/ACKR2/CNTFR/LTBP4/ENG/PXDN

GOBP_RESPONSE_TO_INORGANIC_SUBSTANCE	GOBP_RESPONSE_TO_INORGANIC_SUBSTANCE	2.09E-05	MPO/CPA1/SYT4/KCNA1/HP/CASQ2/PENK/CER1/FABP4/KCNB1/FGA/TACR1/KCNMA1/KCNK3/PCSK1/ASCL1/KCNMB1/CRYAB/KCNA5/SLC13A5/APOB/FGG/RYR3/MAP1B/CYP1B1/DDR2/PRKAA2/TF/TRPC1/ASPN/CACNA1H/NLGN1/NR4A3/ACTA1/NPTX1/ADCY1/FBLN5/CYBRD1/GIP/CD36/MAPT/DLG2/NFATC4/ATP2B4/THBS1/CCL19/SERPINF1/GUCY1B1/CAV1/S100A8/SELENOM/MMP3/HGF/RASGRP2/SLC30A2/FIBIN/SCGB1A1/HPCA/FOSB/SELENOP/TNFRSF11B/SLC8A1/PKD2/MMP2/IL6/PRKN/APOA4/SHH/MIR342/AVPR1A/CLIC4/HBB/SOD3/SYT11/ACER1/LRRK2/DPEP1/PDGFD/DLG4/NEFL/CPNE8/PRNP/RHOB/MTTP/RYR2/FGB/MEF2C/PTCH1/ITPKB/GPLD1/SYT1/SLC30A10/CYP1A1/RASA4B/H19/CCR7/KIT/COL1A1/PDGFRB/[illegible]

GOBP_SPINDLE_ASSEMBLY	GOBP_SPINDLE_ASSEMBLY	2.11E-05	RHOA/STAG2/DRG1/POLDIP2/TUBB/CCDC61/HAUS1/NEK6/TPR/HAUS3/AAAS/BCCIP/SMC3/CHMP6/TUBGCP5/EML3/GOLGA2/RPS3/HDAC3/KPNB1/GOLGA2P5/STAG1/CHMP2B/HAUS2/PDCD6IP/CHEK2/RAB11A/HAUS4/CHMP7/KIF2A/CHMP1A/TPX2/CEP192/VPS4B/RNF4/INO80/GPSM2/HNRNPU/CHMP5/NEK2/HAUS6/SASS6/HAUS5/SMC1A/PRC1/TUBGCP4/TUBGCP2/CCSAP/HAUS8/FBXO5/AURKB/NDC80/ASPM/SPAG5/KIF23/WRAP73/AURKA/MZT1/TNKS/KIFC1/CCNB2/INCENP/SAC3D1/KIF4A/RACGAP1/CDC20/CHMP4C/KIFC2/MAPK15/MISP/PLK1/BIRC5/CDCA8/KIF15/RCC1/KIF11/STIL
GOBP_NUCLEOSIDE_MONOPHOSPHATE_BIOSYNTHETIC_PROCESS	GOBP_NUCLEOSIDE_MONOPHOSPHATE_BIOSYNTHETIC_PROCESS	2.12E-05	RFK/DGUOK/DHODH/ADSS2/AMPD2/UMPS/GMPS/APRT/IMPDH2/ADK/UCK2/TK2/DCTD/GART/NUDT2/ATIC/ADSS1/IMPDH1/PFAS/CAD/PRPS2/CDA/DCK/DUT/CMPK1/SHMT1/PPAT/PAICS/ADA/TYMS/TK1/ENTPD8

GOCC_INTRINSIC_COMPONENT_OF_POSTSYNAPTIC_MEMBRANE	GOCC_INTRINSIC_COMPONENT_OF_POSTSYNAPTIC_MEMBRANE	2.20E-05	KCNA1/CHRM2/EPHA7/GRIN2A/HTR2A/GRIK5/CDH2/CNTN1/PLPPR4/CACNG4/NLGN4X/NLGN1/FXYD6/ADCY1/NLGN4Y/RGS7BP/P2RX1/NRP2/CLSTN2/SLC8A1/EFNB3/NLGN3/LRRC4/DLG4/LRRC4C/KCND2/NRCAM/NLGN2/GABBR1/CHRNB2/PCDH17
GOMF_CARBOXYPEPTIDASE_ACTIVITY	GOMF_CARBOXYPEPTIDASE_ACTIVITY	2.25E-05	CPA1/CPA2/CPXM2/CPE/CPN1/CPA3/NAALAD2/CPXM1/CPA4/CPA6/CPVL/AEBP1/VASH2/CPQ
GOBP_CAMP_MEDIATED_SIGNALING	GOBP_CAMP_MEDIATED_SIGNALING	2.27E-05	ADCYAP1R1/ADCYAP1/AKAP6/SCT/CAP2/ADCY2/PDE7B/ADCY1/GIP/PDE3A/RASD2/LPAR1/PDE2A/SCTR/FFAR4/GLP1R

GOBP_REGULATION_OF_SUPRAMOLECULAR_FIBER_ORGANIZATION	GOBP_REGULATION_OF_SUPRAMOLECULAR_FIBER_ORGANIZATION	2.40E-05	APOA1/SYNPO2/SFRP1/STMN2/ACTN2/MYOC/LMOD1/TACR1/CRYAB/SLIT2/TMOD1/CFL2/CCL26/TUBB4A/FERMT2/TNXB/MAP1B/RGS4/CLIP3/FLNA/PAK3/SVIL/NAV3/PHLDB2/MAP1A/DAAM2/CXCL12/ELN/ADD2/SERPINF2/WASF3/TPM1/CDC42EP3/CCL21/TGFB3/PPM1E/MAPT/KANK4/CSF3/CGNL1/FGF13/LPAR1/LMOD3/TENM1/HDGFL3/PRKN/EMILIN1/FHOD3/ANKRD53/EFEMP2/DLC1/KIRREL1/CCN2/SYNPO/CCL11/BMERB1/ARHGAP28/MIR149/S1PR1/AEBP1/PLEKHH2/TMSB15A/MTSS1/HSPA1A/WASF1/CLU/CORO2B/CYRIA/MAGEL2/MAP2/DSTN/CCR7/MYADM/ARHGEF15/GSN/NRP1/RDX/ARHGAP40/PFN2
GOBP_OVULATION_CYCLE_PROCESS	GOBP_OVULATION_CYCLE_PROCESS	2.46E-05	AFP/SLIT2/PGR/PTPRN/SLIT3/PTX3/TGFB3/ADAMTS1/TGFB2/MMP2/ESR1/MMP19/PDGFRA/CGA/A2M/GDF9/RETN/BMPR1B/INHBA

GOCC_NUCLEAR_REPLICATION_FORK	GOCC_NUCLEAR_REPLICATION_FORK	2.52E-05	SMARCAD1/ZMIZ2/POLA1/PRIM2/RPA1/EME1/MUS81/PARP1/POLA2/MMS22L/MCM10/CARM1/POLD3/TIPIN/PRIM1/WDHD1/EME2/POLD2/TIMELESS/PCNA/MCM3/TONSL/POLD1
GOBP_LOCOMOTORY_BEHAVIOR	GOBP_LOCOMOTORY_BEHAVIOR	2.63E-05	PENK/RELN/ATP1A2/CHRNA3/ADCY5/NEGR1/SEZ6/TMOD1/GLRB/CNTN1/SLC18A2/DAB1/HOXD10/CXCL12/GIP/LGI4/CHL1/CHRNB4/SOBP/RASD2/USP2/C1QL1/MEIS1/SELENOP/PDE1B/PRKN/CDH23/PREX2/SNAP25/SLITRK6/PBX3/EFNB3/UCHL1/LRRK2/SNCG/SLURP1/DLG4/KCND2/NLGN2/APBA1/ALDH1A3/FGF12/MCOLN3/CHRNB2/HOXD9

GOBP_COLLAGEN_FIBRIL_ORGANIZATION	GOBP_COLLAGEN_FIBRIL_ORGANIZATION	2.71E-05	COL2A1/COL14A1/SFRP2/TNXB/CYP1B1/DDR2/COL11A2/GREM1/DPT/SERPINF2/FKBP10/TLL1/LUM/TGFB2/ADAMTS3/COMP/COL3A1/EMILIN1/EFEMP2/FOXC2/LOX/AEBP1/FMOD/COL1A2/COL1A1/COL5A1/COL12A1/COL5A2
GOBP_HEART_MORPHOGENESIS	GOBP_HEART_MORPHOGENESIS	2.73E-05	HAND1/COL2A1/HAND2/NKX2-5/ANKRD1/ALDH1A2/TMEM100/CPE/SLIT2/FLRT2/ASB2/MYL3/SFRP2/NOG/HTR2B/SLIT3/FLNA/ZFPM2/ELN/TPM1/ACTC1/FOXF1/TGFB3/ADAMTS1/EDNRA/SMARCD3/NODAL/CCN1/TGFB2/RBP4/NRP2/HEYL/PKD2/SHH/SMO/DKK1/ISL1/DLC1/PARVA/SNAI2/FOXC2/DCHS1/S1PR1/ILK/HAS2/HEG1/SOX17/RYR2/TGFBR3/CLDN5/TEK/MEF2C/PTCH1/TWIST1/TBX5/ROBO1/VANGL2/ACVR1/COL5A1/SEMA3C/NDRG4/NRP1/APLNR/MICAL2/BMP5/FGFR2/FZD1/SOX11/PITX2/GJA5/ENG/SNAI1

GOBP_CARDIAC_MUSCLE_CELL_DIFFERENTIATION	GOBP_CARDIAC_MUSCLE_CELL_DIFFERENTIATION	2.75E-05	MYH11/ACTN2/MYOCD/NKX2-5/BVES/AKAP6/IGF1/ASB2/PI16/RGS4/SGCD/GREM1/WT1/AGT/ACTC1/RGS2/TBX18/SLC8A1/FHOD3/PDGFRA/DKK1/ALPK3/ISL1/FZD7/SORBS2/NOX4/MEF2C/MIR222/IRX3/TBX5/ACVR1/PDGFRB/HAMP
GOBP_MRNA_TRANSPORT	GOBP_MRNA_TRANSPORT	2.77E-05	NUP155/AGFG1/NUP107/NXT1/AKAP8L/DDX19B/HNRNPA1L2/NUP133/FMR1/RANBP2/SENP2/POLDIP3/G3BP2/NUP153/ALKBH5/SMG6/CETN3/NUP98/NUP214/NCBP3/MAGOH/PABPN1/TPR/SRSF7/SETD2/AAAS/SMG7/NUP54/NUTF2/RBM15B/THOC6/AHCTF1/IGF2BP2/SMG1/XPO1/NUP160/SEH1L/SMG5/SRSF3/NUP35/CHTOP/NUP62/POLR2D/NCBP1/DHX9/POM121C/NUP85/NSUN2/NUP43/EIF4A3/SRSF1/HSF1/NUP58/NUP93/UPF1/HNRNPA2B1/CASC3/MCM3AP/POM121/ZC3H3/NUP205/LRPPRC/NUP37/NUP88/SEC13/NUP50/KHSRP/HNRNPA1/MVP/GLE1/TNKS/SLBP/THOC3/ALYREF/DDX39A/NDC1/NUP188/POM121B/EIF5AL1/NXF3

GOMF_G_PROTEIN_COUPLED_RECEPTOR_BINDING	GOMF_G_PROTEIN_COUPLED_RECEPTOR_BINDING	2.96E-05	GCG/UCN3/CCL16/GHRH/TAC1/PENK/AGTR1/SFRP1/MYOC/ADCYAP1/NPFFR2/SCT/GNAO1/PTCH2/RSPO1/CCL26/NPY/RSPO3/FLNA/REEP2/REEP1/TUB/CXCL6/CXCL12/GIP/AGT/WNT9A/CCL21/CCL19/NDP/CX3CR1/C10orf99/CNRIP1/FCN1/MLN/GNAL/PRKN/AVPR1A/PALM/CCL2/CCKBR/FZD7/UCHL1/WNT2B/WNT10B/GPRASP2/CCL11/DLG4/S1PR1/PTGER1/PRNP/FPR1/GNAI1/HSPA1A/TAFA5/PROK2/GNA14/PTCH1/UCN2/GNAZ/CTHRC1/P2RY1
GOBP_INTESTINAL_CHOLESTEROL_ABSORPTION	GOBP_INTESTINAL_CHOLESTEROL_ABSORPTION	3.04E-05	PNLIP/APOA2/CEL/APOA1/SOAT2/CD36/APOA4

GOBP_NEGATIVE_REGULATION_OF_MITOTIC_CELL_CYCLE_PHASE_TRANSITION	GOBP_NEGATIVE_REGULATION_OF_MITOTIC_CELL_CYCLE_PHASE_TRANSITION	3.04E-05	BRCC3/BRCA1/BTN2A2/TOPBP1/PML/NUF2/MIIP/BARD1/CDK2AP2/DONSON/ZFYVE19/ZW10/PINX1/TRIAP1/RAD21/CCNG1/KLF4/TAOK2/RINT1/TTK/PRKDC/KNTC1/PIDD1/XRCC3/FBXO5/FZR1/CASP2/ZWILCH/AURKB/NDC80/BUB1/BUB3/RFWD3/RBBP8/CDC14B/NBN/GEN1/EZH2/GTSE1/SPC25/CDK1/PLK2/CENPF/INCENP/CHEK1/SPC24/BUB1B/BLM/CDC20/TP53/TICRR/CLSPN/CHMP4C/CCNB1/HASPIN/MAD2L1/DTL/MDM2/PLK1/BIRC5/GFI1B/CDCA8/CCND1/ZWINT/E2F7/CDT1/ORC1/MUC1
GOBP_NEGATIVE_REGULATION_OF_POTASSIUM_ION_TRANSPORT	GOBP_NEGATIVE_REGULATION_OF_POTASSIUM_ION_TRANSPORT	3.06E-05	VIP/CASQ2/ACTN2/HTR2A/GRP/RGS4/KCNE4/KCNH2/CAV1

GOCC_INTRINSIC_COMPONENT_OF_MITOCHONDRIAL_INNER_MEMBRANE	GOCC_INTRINSIC_COMPONENT_OF_MITOCHONDRIAL_INNER_MEMBRANE	3.18E-05	PPOX/TIMM17B/OXA1L/C12orf73/TIMM23/PISD/TIMM23B/TMEM223/ETFDH/TMEM177/TIMM17A/PET100/TMEM11/SAMM50/CHCHD6/MTX1/MTX2/APOO/MICOS13/MCUB/MPC1/AFG3L2/SLC25A3/SCO1/IMMT/UQCC3/SFXN4/HSPA9/CHCHD3/SLC25A19/GHITM/L2HGDH/COQ2/SFXN2/DNAJC11/SFXN1/COX18/CHCHD10/CCDC51/MCU/SCO2

GOBP_STRIATED_MUSCLE_CONTRACTION	GOBP_STRIATED_MUSCLE_CONTRACTION	3.18E-05	CASQ2/SYNM/ATP1A2/PLN/CASQ1/NKX2-5/CTNNA3/ANK2/KCNA5/ABCC9/KLHL41/DMD/DTNA/MYL3/SMPX/FLNA/CACNA1C/SGCD/SCN4B/KCNJ8/HRC/CACNB2/KCNE4/TPM1/KCNH2/CHGA/ADRA1B/GJC1/DMPK/CAV1/FGF13/RGS2/LMOD3/STAC/SLC8A1/CACNA2D1

GOBP_REGULATION_OF_NERVOUS_SYSTEM_DEVELOPMENT	GOBP_REGULATION_OF_NERVOUS_SYSTEM_DEVELOPMENT	3.19E-05	SYT4/NKX2-2/SOX10/RELN/EPHA7/ASCL1/F2/SLIT2/FLRT2/DLL3/CHODL/NOG/ADGRB3/MAP1B/MAP6/NGF/PAK3/PTN/FGF2/PRTG/DAB1/PLXNA4/JAM2/DAAM2/DIXDC1/NLGN1/PTPRD/CXCL12/GLI3/SEMA3E/IL33/NTN1/WASF3/LGI4/RXRG/MAPT/RND2/NFATC4/LPAR3/SERPINF1/NKX6-1/CX3CR1/SMARCD3/FGF13/HGF/ASPA/SERPINE2/L1CAM/LRRN1/CLSTN2/HEYL/IL6/SHH/SMO/FN1/DKK1/SLITRK6/SPART/EFNB3/OXTR/NLGN3/CRABP2/DPYSL5/ID4/ATOH1/SEMA6A/CCL11/SEMA6D/NEURL1/NEFL/TENM4/SRPX2/ISLR2/PTPRZ1/SYNDIG1/THY1/MIR222/NLGN2/SEMA6C/THBS2/SEMA3G/VEGFC/ADGRB1/MAP2/SEMA5B/SEMA3D/ROBO1/CXCR4/GPER1/ELAPOR2/IL6ST/IL34/LEF1/KIT/AMIGO2/SEMA3C/OXT/[illegible]

GOCC_PRESYNAPTIC_MEMBRANE	GOCC_PRESYNAPTIC_MEMBRANE	3.21E-05	KCNA1/CHRM2/GRIK3/GPM6A/CADM3/GRIN2A/HTR2A/GRIK5/CDH2/CNTN1/ADAM23/CNR1/PTPRD/FXYD6/CNTNAP1/RGS7BP/P2RX1/ATP2B4/PDE2A/SNAP25/SYT11/EFNB3/NLGN2/APBA1/GABBR1/SYT1/ADORA3/CHRNB2/PCDH17/SYP/P2RY1/GPER1/SNCAIP/LRRC4B/IGSF21/STX1B/DNM1/NTNG2/STX2/KCTD12/SLC6A4

GOBP_POSITIVE_REGULATION_OF_SUPRAMOLECULAR_FIBER_ORGANIZATION	GOBP_POSITIVE_REGULATION_OF_SUPRAMOLECULAR_FIBER_ORGANIZATION	3.24E-05	APOA1/SYNPO2/SFRP1/STMN2/ACTN2/MYOC/LMOD1/TACR1/CFL2/CCL26/FERMT2/TNXB/MAP1B/NAV3/SERPINF2/WASF3/TPM1/CDC42EP3/CCL21/TGFB3/PPM1E/MAPT/CSF3/LPAR1/TENM1/ANKRD53/EFEMP2/KIRREL1/CCN2/SYNPO/CCL11/MTSS1/HSPA1A/WASF1/CLU/MAGEL2/DSTN/CCR7/ARHGEF15/GSN/NRP1/PFN2

GOBP_CARDIAC_MUSCLE_CONTRACTION	GOBP_CARDIAC_MUSCLE_CONTRACTION	3.26E-05	CASQ2/ATP1A2/PLN/NKX2-5/CTNNA3/ANK2/KCNA5/ABCC9/DMD/MYL3/FLNA/CACNA1C/SGCD/SCN4B/KCNJ8/HRC/CACNB2/KCNE4/TPM1/KCNH2/CHGA/ADRA1B/GJC1/CAV1/FGF13/RGS2/SLC8A1/CACNA2D1/GJA1/CCN2/RYR2/STC1/CLIC2/PDE5A/FGF12/CXCR4/GSN/SCN3B/MYL4/SCN1B/GSTM2/SNTA1

GOBP_REGULATION_OF_CHEMOTAXIS	GOBP_REGULATION_OF_CHEMOTAXIS	3.33E-05	ELANE/AZU1/THBS4/FGF10/SLIT2/CCL26/PTN/SMOC2/FGF2/GREM1/PLXNA4/JAM3/CXCL12/F7/SEMA3E/PRKD1/CCL21/THBS1/CCL19/CX3CR1/LPAR1/FGFR1/IL6/SERPINE1/PDGFRA/CCL2/GPR183/NTF3/CCN3/PDGFD/SEMA6A/FGF1/ADGRA2/SEMA6D/MIR149/S1PR1/DUSP1/BST1/CXCR2/RARRES2/HSPB1/CAMK1D/SCG2/RIPOR2/SEMA6C/SEMA3G/VEGFC/MPP1/SEMA5B/SEMA3D/ROBO1/CXCR4/CCR7/IL34/PDGFRB/SEMA3C/NRP1/TNFSF18/EDN2/FPR2/P2RY12/ANGPT2/NOVA2/DUSP3/MET/SEMA6B/C5/CCL7/SEMA5A/VEGFB/C3AR1/C5AR1/XCL1/CCR4/CSF1R/AIF1/C5AR2/TUBB2B

GOBP_CALCIUM_MEDIATED_SIGNALING	GOBP_CALCIUM_MEDIATED_SIGNALING	3.35E-05	AZU1/CASQ2/ATP1A2/AGTR1/PLN/CASQ1/ANK2/GRIN2A/AKAP6/TMEM100/SLC8A2/RGN/IGF1/DMD/HTR2B/PTGFR/CACNA1C/SGCD/P2RX2/RCAN2/HRC/MAPT/NFATC4/ATP2B4/DMPK/CX3CR1/SELP/NMUR1/HPCA/SLC8A1/AVPR1A/LRRK2/PRNP/BST1/CXCR2/ITPR1/RYR2/CLIC2/CXCR1/SELE/ADGRB2/CXCR4/CCR7/ACKR3/NCALD/MYOZ1/EDNRB/EDN2/FPR2/ACKR2/P2RY12/GSTM2/CHRM3/LMCD1
GOBP_KINETOCHORE_ORGANIZATION	GOBP_KINETOCHORE_ORGANIZATION	3.39E-05	RNF4/CENPW/NUF2/SMC4/KNTC1/NDC80/CENPA/SMC2/CENPX/CENPK/CENPF/DLGAP5/CENPN/CENPE/CENPH/CDT1

GOBP_RESPONSE_TO_CALCIUM_ION	GOBP_RESPONSE_TO_CALCIUM_ION	3.39E-05	SYT4/CASQ2/PENK/KCNB1/FGA/KCNMA1/PCSK1/KCNMB1/FGG/RYR3/PRKAA2/TRPC1/NLGN1/ADCY1/THBS1/CAV1/RASGRP2/HPCA/FOSB/PKD2/CLIC4/SYT11/ACER1/DPEP1/CPNE8/MTTP/RYR2/FGB/MEF2C/ITPKB/GPLD1/SYT1/RASA4B
GOBP_PROTEIN_LOCALIZATION_TO_EXTRACELLULAR_REGION	GOBP_PROTEIN_LOCALIZATION_TO_EXTRACELLULAR_REGION	3.42E-05	PLA2G1B/GCG/SYT4/UCN3/CARTPT/CELA2A/VIP/CER1/TACR2/SFRP1/NEUROD1/NNAT/KCNB1/FGA/PCSK1/ADCYAP1/ANKRD1/ADCY5/KCNA5/IGF1/PTPRN/FGG/ARL4D/CNR1/MYOM1/GIP/CHGA/TGFB3/APBB1/RFX6/CAVIN1/NKX6-1/LTBP1/TGFB2/FBN1/RBP4/COMP/IL6/PRKN/MYH10/NR1H4/SNAP25/ISL1/CCN3/CD200/SNCG/GPR27/RAB3B/ABCA1/MTTP/KLF7/SCG2/FGB/NLGN2/RAB3C/BLK/GPLD1/LTBP2/MYRIP/WLS/VEGFC

GOBP_CARDIAC_CELL_DEVELOPMENT	GOBP_CARDIAC_CELL_DEVELOPMENT	3.44E-05	MYH11/ACTN2/NKX2-5/BVES/AKAP6/IGF1/ASB2/PI16/RGS4/SGCD/AGT/ACTC1/RGS2/TBX18/SLC8A1/FHOD3/PDGFRA/ALPK3/ISL1/SORBS2/TGFBR3/INHBA/TBX5/PDGFRB/HAMP
GOCC_IMMUNOGLOBULIN_COMPLEX	GOCC_IMMUNOGLOBULIN_COMPLEX	3.53E-05	IGLL5/IGLV2-11/IGLV2-33/IGLV3-1/IGHA1/IGKV2D-24/IGKV2D-30/IGKV6D-41/IGHD/IGLV3-21/IGHV3-72/IGHV3-64D/IGKV2-40/IGHV3-33/IGLV4-3/CD79B/IGKV3D-11/IGHV3-48/IGHV3-66/IGLV2-14/PIGR/IGKV2D-29/IGKV1-12/IGKV1D-13/IGKV1-16/IGHA2/IGHV1-58/IGLV3-27/IGLV5-45/IGLV5-37/IGHM/IGLV4-60/IGLV5-48/IGHV3-49/IGLV2-18/IGLV4-69/IGLV3-9/IGKV1D-12/IGLV3-12/IGKV5-2/IGLV7-46/IGHV3-13/IGLL1

GOCC_SMALL_RIBOSOMAL_SUBUNIT	GOCC_SMALL_RIBOSOMAL_SUBUNIT	3.55E-05	RPS20/DHX29/MRPS17/RPS26/RPS25/MRPS23/DAP3/RPS15A/MRPS15/RPS13/RPS24/RPS21/MRPS10/RPS11/UBA52/RPS8/RPS16/RPS4X/RPS12/MRPS7/MRPS33/RPS10/FAU/RPS27L/RPS14/RPS9/RPS18/RPS3/RPS5/RPS23/MRPS11/RPS3A/MRPS18B/MRPS9/MRPL42/RPS7/RPS28/MRPS18A/RACK1/MRPS22/RPS19/PTCD3/RPS6/MRPS26/MRPS5/CHCHD1/RPS17/MRPS18C/MRPS12/MRPS25/AURKAIP1/RPS15/MRPS28/RPSA/MRPS27/MRPS16/RPS2/MRPS34/MRPS35/NPM1/MRPS2
GOBP_RESPONSE_TO_FOOD	GOBP_RESPONSE_TO_FOOD	3.56E-05	CLPS/MPO/SPX/CARTPT/GHRH/GHR/NPY

GOBP_AEROBIC_RESPIRATION	GOBP_AEROBIC_RESPIRATION	3.58E-05	OXA1L/BLOC1S1/OGDH/NDUFB5/DNAJC15/IDH3G/NDUFC1/NDUFA1/DHTKD1/COX7B/SUCLA2/CHCHD2/NDUFB4/UQCRB/ATP5MG/COX6B1/RHOA/NDUFS8/UQCR11/NDUFB2/COA6/ATP5MF/DGUOK/COX7C/NDUFA8/COX7A2L/ATP5F1C/NDUFA6/NDUFB10/ATP5PD/C2orf69/DLD/FXN/SUCLG2/ACO1/NDUFS5/COX7A2P2/SLC25A23/NDUFB3/NDUFA12/NDUFA5/IREB2/UQCRQ/ABCD1/MRPS36/PARK7/BID/NDUFA9/NDUFB6/MDH1/NDUFA11/NDUFAF1/NDUFS6/UQCRFS1/AFG1L/NDUFA10/NDUFS2/ATP5F1EP2/NDUFA2/NDUFS1/COX7A2/NDUFS3/UCN/SDHD/DLST/NDUFB7/IDH3B/COX5B/ATP5PO/NDUFAB1/UQCR10/IDE/PDHA1/COX4I1/ATP5PB/COX6A1/NDUFA13/SLC25A33/UQCC3/PDE12/CYCS/SDHB/CS/MTFR1/COQ9/COX15/MTFR2/COX5A/M[illegible]

GOCC_U2_TYPE_SPLICEOSOMAL_COMPLEX	GOCC_U2_TYPE_SPLICEOSOMAL_COMPLEX	3.71E-05	PRPF39/RBM8A/SNU13/SNRPD2/IK/GCFC2/PRPF18/SNW1/MAGOHB/SNRPD3/ISY1/LSM5/DHX8/CDC5L/LSM6/RBM22/BCAS2/SNRPG/CRNKL1/LUC7L2/SNRPC/PRPF6/HTATSF1/SF3B1/CCDC12/PRPF38A/DHX16/SF3A1/PRPF31/PPIL1/LSM3/SNRPF/TFIP11/MFAP1/LSM2/SNRPA1/PHF5A/SMU1/CDC40/SART1/SF3A3/SNRNP70/LSM7/SNRPB/XAB2/EFTUD2/PRPF19/PRPF8/SRRM2/SNRNP40/PRPF40A/AQR/BUD13/EIF4A3/SNRNP200/SF3B2/PRPF4/SNRPD1/CASC3/TXNL4A/SF3A2/SF3B5/YJU2/SF3B3/U2AF2/LSM4/PLRG1

GOBP_POSITIVE_REGULATION_OF_CELL_DEVELOPMENT	GOBP_POSITIVE_REGULATION_OF_CELL_DEVELOPMENT	3.71E-05	NKX2-2/SOX10/APOA1/RELN/ADIPOQ/MYOC/FGA/ASCL1/BNC1/SLIT2/FGG/CHODL/FERMT2/LIMS2/MAP1B/MAP6/FLNA/NGF/PAK3/PTN/FGF2/PLXNA4/DIXDC1/PTPRD/CXCL12/GLI3/IL33/NTN1/PDE3A/MAPT/RND2/LPAR3/SERPINF1/NKX6-1/CX3CR1/SMARCD3/LMOD3/ASPA/SERPINE2/L1CAM/IL6/SHH/SMO/DAB2/FN1/CRABP2/ID4/NEURL1/NEFL/ILK/HAS2/TENM4/ISLR2/PTPRZ1/FGB/CLDN5/PDE5A/MIR222/ITGB3/VEGFC/ROBO1/CXCR4/GPER1/MYADM/IL6ST/IL34/KIT

GOMF_EXONUCLEASE_ACTIVITY_ACTIVE_WITH_EITHER_RIBO_OR_DEOXYRIBONUCLEIC_ACIDS_AND_PRODUCING_5_PHOSPHOMONOESTERS	GOMF_EXONUCLEASE_ACTIVITY_ACTIVE_WITH_EITHER_RIBO_OR_DEOXYRIBONUCLEIC_ACIDS_AND_PRODUCING_5_PHOSPHOMONOESTERS	3.74E-05	DIS3L2/CNOT8/MGME1/USB1/PARN/RAD9A/CNOT6L/CPSF3/DIS3L/DIS3/PAN2/ISG20L2/XRN1/APEX2/CNOT7/EXOSC10/EXOSC2/EXOSC7/APTX/EXOSC5/PDE12/CNOT6/EXOSC9/EXOSC4/CNOT1/TOE1/DCLRE1B/ERI2/POLE/ISG20/POLRMT/ERI1/DCLRE1A/EXO1/FEN1/POLD1
GOBP_CGMP_MEDIATED_SIGNALING	GOBP_CGMP_MEDIATED_SIGNALING	3.79E-05	NPPB/HTR2B/IRAG1/GUCY1A1/PDE9A/PDE3A/CD36/PRKG1/THBS1/GUCY1B1/PDE2A/RUNDC3A/NPR1/GUCY1A2/NPR2/EDNRB/NPPC

GOBP_MITOTIC_SPINDLE_ORGANIZATION	GOBP_MITOTIC_SPINDLE_ORGANIZATION	3.84E-05	STAG1/CHMP2B/NUP62/PDCD6IP/CHEK2/RAB11A/CHMP7/INTS13/KIF2A/CHMP1A/TPX2/CEP192/VPS4B/GPSM2/HNRNPU/NUF2/CHMP5/NEK2/RAN/CKAP5/SASS6/TTK/STMN1/SMC1A/PSRC1/PRC1/MAP1S/FAM110A/CCSAP/PCNT/VCP/AURKB/NDC80/KIF23/WRAP73/PTPA/AURKA/MZT1/TNKS/SPC25/NUDC/KIFC1/WDR62/PLK2/BORA/INCENP/KIF4A/RACGAP1/PARP3/DLGAP5/CDC20/CHMP4C/KIFC2/CCNB1/TACC3/CENPE/MISP/PLK1/BIRC5/CENPH/CDCA8/KIF15/RCC1/KIF11/STIL

GOBP_CYTOKINESIS	GOBP_CYTOKINESIS	3.84E-05	SETD2/EXOC1/STAMBP/ARF1/SEPTIN9/TMEM250/RHOC/CHMP6/AHCTF1/CDC25B/BIRC6/PIN1/CALM3/BCL2L1/ALKBH4/CHMP2B/NUP62/PDCD6IP/RTKN/CFL1/RAB11A/CHMP7/CHMP1A/PRPF40A/CKAP2/VPS4B/CHMP5/ANK3/SPTBN1/PLEC/RAB11FIP4/SPIRE2/ZFYVE19/BRCA2/KIF20A/PKN2/STMN1/PRC1/GIT1/ANXA11/AURKB/ENTR1/PDXP/KIF20B/CDC14B/KIF23/AURKA/CENPA/EXOC6/BIN3/KIF14/CNTROB/DRD2/GIPC1/PLK2/TTC19/CIT/ANLN/INCENP/CENPV/MYO19/KIF4A/RACGAP1/PLK4/CHMP4C/ESPL1/ECT2/PLK1/BIRC5/CEP55/CDCA8/E2F8/E2F7/TRIM36/IGF1R/SSTR5

GOBP_HEMOSTASIS	GOBP_HEMOSTASIS	3.89E-05	CELA2A/APOH/VTN/FGA/TPH1/CTSG/SERPIND1/MYL9/F2/FGG/SERPINA10/ADAMTS18/FBLN1/FLNA/CSRP1/TFPI2/F7/F13A1/SERPINF2/MMRN1/CD36/PRKG1/DGKG/P2RX1/THBS1/CAV1/SELP/SERPINE2/COMP/IL6/COL3A1/SERPINE1/SHH/HBB/EMILIN1/PDGFRA/PROS1/FN1/KRT1/F8/TSPAN32/AVPR2/THBD/ILK/SCUBE1/FAP/PDPN/HSPB1/FGB/ACTN1/SERPING1/TFPI/BLK/F10/ITGB3/MPIG6B/P2RY1/CD34/IL6ST/AXL/TREML1/MERTK/ENTPD1/P2RY12/PEAR1/F2RL2/TBXA2R
GOBP_POSITIVE_REGULATION_OF_CATION_CHANNEL_ACTIVITY	GOBP_POSITIVE_REGULATION_OF_CATION_CHANNEL_ACTIVITY	3.93E-05	KCNA1/RELN/ACTN2/CASQ1/KCNK3/JPH2/ANK2/AKAP6/CACNG4/CACNB2/EDNRA/STAC/PKD2/CACNA2D1/CCL2/ADRB2/NLGN3

GOMF_TRANSLATION_FACTOR_ACTIVITY_RNA_BINDING	GOMF_TRANSLATION_FACTOR_ACTIVITY_RNA_BINDING	3.98E-05	EIF3J/GFM1/EIF1/EIF2S3/HBS1L/GSPT1/DENR/EEF1A1P5/EIF2D/EIF2B5/EIF1AX/EIF3B/EEF1A1/EIF4E2/EIF3D/EIF2B2/EIF3H/ABTB1/GTPBP1/EIF3E/EIF3I/EEF2K/EEF1D/EEF1B2/EEFSEC/EIF4G1/COPS5/MRPL58/EIF3A/EIF3G/EIF4B/EIF1AD/EIF4H/EIF2S1/EEF2/EIF4E/GFM2/GCN1/AGO2/MTIF2/EIF3CL/EIF2S3B/TUFM/EIF3C/EIF5A/EEF1A2/EIF5AL1/EFL1
GOBP_PROTEIN_LOCALIZATION_TO_CELL_SURFACE	GOBP_PROTEIN_LOCALIZATION_TO_CELL_SURFACE	3.99E-05	ACTN2/TMEM35A/FGF10/ANK2/FGF7/RIC3/FLNA/JAM3/MAP1A/GPIHBP1/GPM6B/FBLN5/ANGPT1/FCN1/PRRT1

GOBP_MEIOTIC_CELL_CYCLE	GOBP_MEIOTIC_CELL_CYCLE	4.20E-05	CDC25B/TUBGCP5/PPP2R1A/GOLGA2/CDC23/CNTD1/TUBG1/ANAPC15/YTHDF2/ANAPC5/XRCC2/FIGNL1/STAG1/SGO2/TOP3A/TRIP13/OVOL1/ING2/MSH6/RPA1/PSMC3IP/PIWIL4/MNS1/NSUN2/CALR/EHMT2/FANCM/SLX4/ANAPC2/NUF2/NEK2/RAD54B/EME1/MUS81/YTHDC2/SPIRE2/NCAPH2/ZW10/BRCA2/RAD21/SMC4/TTK/NCAPD2/CYP26B1/SMC1A/SGO1/TUBGCP4/TUBGCP2/MSX1/FBXO5/FZR1/SIRT7/NDC80/BUB1/BUB3/RBBP8/ASPM/NBN/RMI1/AURKA/PRR19/RAD51AP1/CDC25C/DMC1/LFNG/SMC2/CKS2/PTTG1/EME2/MSX2/CENPX/CCNB2/TESMIN/INCENP/KIF18A/PDIK1L/BUB1B/NDC1/CDC20/RAD51/EXO1/NCAPH/FANCD2/ESPL1/PIWIL1/NCAPD3/MAPK15/H2AX/PLK1/RSPH1/TOP2A/RAD54L/FANCA/CDC25A/CCNE2/SY[illegible]

GOBP_MITOCHONDRIAL_RESPIRATORY_CHAIN_COMPLEX_ASSEMBLY	GOBP_MITOCHONDRIAL_RESPIRATORY_CHAIN_COMPLEX_ASSEMBLY	4.24E-05	NDUFAF4/NDUFB9/CHCHD7/FASTKD3/COA1/DMAC2/UQCC2/NDUFS4/COX16/SDHAF2/COA8/NDUFB8/COX19/NDUFC2/OMA1/OXA1L/NDUFB5/NDUFC1/NDUFA1/C12orf73/NDUFB4/DMAC1/NDUFS8/TMEM223/NDUFB2/NDUFA8/SMIM20/SDHAF4/PET100/SDHAF1/ACAD9/NDUFA6/NDUFB10/NDUFS5/COA4/NDUFB3/NDUFA12/NDUFA5/NDUFAF5/NDUFAF2/NDUFAF6/TMEM186/NDUFA9/SAMM50/NDUFB6/NDUFA11/NDUFAF1/CHCHD4/UQCRFS1/NDUFA10/NDUFS2/NDUFA2/NDUFS1/NDUFS3/TFAM/NDUFB7/SDHAF3/BCS1L/NDUFAB1/TACO1/SCO1/NDUFA13/SLC25A33/UQCC3/AIFM1/TIMM21/ECSIT/FOXRED1/TTC19/COX18/NDUFS7/SCO2
GOBP_NEGATIVE_REGULATION_OF_CELLULAR_RESPONSE_TO_GROWTH_FACTOR_STIMULUS	GOBP_NEGATIVE_REGULATION_OF_CELLULAR_RESPONSE_TO_GROWTH_FACTOR_STIMULUS	4.23E-05	CER1/SFRP1/NGFR/CHRDL1/RBPMS2/SLIT2/CHRDL2/SFRP2/NOG/GREM2/BMPER/FGF2/GREM1/DCN/CHRD/AGT/ATP2B4/THBS1/FBN1/MIR342/EMILIN1/DKK1/SPART/SEMA6A/ADGRA2/MIR149

GOBP_NUCLEOSOME_ORGANIZATION	GOBP_NUCLEOSOME_ORGANIZATION	4.27E-05	H3C12/SMARCD1/SETD2/SUPT16H/H3-3B/H2BC7/H1-10/H3C10/ZNHIT1/SMARCC1/MACROH2A1/ACTL6A/KAT5/H3-3A/SMARCC2/H2AW/SART3/ACTR6/H4C9/H3C1/SMARCB1/H2BC11/H4C4/NAP1L4/SETSIP/RUVBL1/GRWD1/H4C3/NASP/BRD2/RUVBL2/H2BC9/SET/SPTY2D1/H1-2/POLE3/H4C2/H2BC18/SRCAP/ANP32B/ANP32E/MCM3AP/H4C1/H2BC10/HMGB2/H1-3/SSRP1/LIN54/H3C13/H3C2/SMARCD2/H3C7/SMARCE1/SOX9/H2BC12/NPM1/SMARCA4/H2BC15/HMGA1/H2BC14/H3C6/H2AX/CHAF1A/H3C8/H2BC13/H2BC17/CHAF1B/DNAJC9/H3C11/H1-4/H3C3/ASF1B/MCM2/H1-5

GOBP_UROGENITAL_SYSTEM_DEVELOPMENT	GOBP_UROGENITAL_SYSTEM_DEVELOPMENT	4.27E-05	CER1/AGTR1/SFRP1/ADIPOQ/MYOCD/FGF10/EPHA7/GFRA1/ALDH1A2/SLIT2/RGN/ACTA2/TMEM59L/AR/SIX2/GDF6/NOG/ADAMTS16/GPC3/BMPER/FGF2/TNC/ZBTB16/GREM1/PYGO1/WT1/GLI3/BICC1/KCNJ8/GREB1L/AGT/ITGA8/FOXF1/ADAMTS1/GLI1/SERPINF1/KANK2/EDNRA/CYP7B1/ANGPT1/TBX18/TGFB2/FBN1/RBP4/WWTR1/HEYL/PKD2/KLF15/MMP2/ESR1/SHH/SMO/PDGFRA/OSR1/PBX1/CRLF1/TNS2/WNT2B/UPK3A/TCF21/LRRK2/ID4/PDGFD/FOXC2/DCHS1/FGF1/CC2D2A/ENPEP/ILK/HAS2/SOX17/CXCR2/NID1/TEK/MTSS1/MEF2C/COL4A4/PTCH1/IRX3/NFIA/GLIS2/ITGB3/DYNC2H1/SULF1/CD34/VANGL2/ACVR1/PDGFRB/PECAM1/GLI2/EDNRB/NRP1
GOBP_SMOOTH_MUSCLE_CELL_MIGRATION	GOBP_SMOOTH_MUSCLE_CELL_MIGRATION	4.29E-05	ADIPOQ/VTN/TACR1/MYOCD/SLIT2/IGF1/CYP1B1/DDR2/NR4A3/AGT/TPM1/PRKG1/ADAMTS1/LPAR1/SERPINE1/IGFBP5/CCN3/PARVA/PDGFD/CCN4/SEMA6D/HAS2/MEF2C/TAFA5/ITGB3/PDGFRB/LRP1/NDRG4/NRP1

GOBP_REGULATION_OF_VESICLE_MEDIATED_TRANSPORT	GOBP_REGULATION_OF_VESICLE_MEDIATED_TRANSPORT	4.30E-05	PRTN3/SYT4/AZU1/APOA2/AHSG/APOA1/CHRM2/APOC3/ACTN2/ADIPOQ/VTN/KCNB1/FGA/BVES/HTR2A/RSPO1/FGG/STON1/PLA2G5/GRIK5/GPC3/EPHA3/CLIP3/TF/GAS1/GREM1/CNR1/TUB/SFRP4/CACNA1H/NLGN1/ADCY1/PTX3/CALY/PRKD1/CACNB2/SLC17A7/CD36/COLEC11/CCL21/FOXF1/P2RX1/CCL19/CAV1/PIP4P2/ANGPT1/C4A/FCN1/HPCA/C4B/PRKCB/PRKN/SERPINE1/DAB2/CCL2/RAB31/DKK1/SGIP1/SYT11/BSN/MIR27B/NTF3/LRRK2/DLG4/RAB3B/EHD2/USH1G/SEPTIN4/CAMK1D/FGB/APBA1/CLU/RAB3C/BLK/FCGR2B/SYT1/ITGB3/SELE/IL13RA2/SYP/P2RY1/ARC/LRP1/SYT5/CADPS/AXL/NDRG4/NRP1/RDX/BACE1/PFN2/MERTK
GOBP_PROTEIN_CONTAINING_COMPLEX_REMODELING	GOBP_PROTEIN_CONTAINING_COMPLEX_REMODELING	4.37E-05	MPO/APOA2/APOA1/APOC3/AGTR1/APOB/PLA2G5/AGT/NR1H4/APOA4/LPL/MTTP

GOBP_POSITIVE_REGULATION_OF_DNA_REPAIR	GOBP_POSITIVE_REGULATION_OF_DNA_REPAIR	4.54E-05	ING3/ARID2/ARID1A/SLF2/ACTR2/ERCC6/HMGB1/MGMT/SIRT1/YEATS4/ACTR8/TRRAP/TIGAR/UBE2N/RNF168/UIMC1/SHLD2/SHLD3/WAS/MAD2L2/SMARCD1/EP400/PNKP/RIF1/BRD7/BRD8/MRGBP/CCDC117/RPS3/SMARCC1/PIAS4/ACTL6A/PARP9/KAT5/SPIDR/SMARCC2/SMCHD1/UCHL5/HDAC10/HDGFL2/INO80D/MMS19/SMARCB1/FUS/BRCC3/DHX9/BRCA1/RUVBL1/XRCC1/DTX3L/INO80E/INO80/SLF1/RUVBL2/MCRS1/FANCB/PARP1/EGFR/SPIRE2/NFRKB/BCL7A/PRKDC/NPAS2/TRIM28/DDX11/KMT5C/SMARCD2/EYA3/USP1/SIRT6/RBBP8/SMARCE1/WRAP53/RAD51AP1/SMARCA4/TMEM161A/FOXM1/TIMELESS/PCNA/PARP3/PRKCG/H2AX
GOMF_MONOCARBOXYLIC_ACID_BINDING	GOMF_MONOCARBOXYLIC_ACID_BINDING	4.68E-05	PLA2G1B/AFP/ALB/FABP4/UGT2B17/S100A8/RBP7/FABP3/UGT1A7/S100A9/SERPINA5/NR1H4/FFAR4/CRABP2/PTGDS/ACOX2/LRAT/ACACB/RBP1/RBP5/CYP2W1/GSTM2/LCN12/ACOXL/PYGL/OXER1/RBP2/SNCA/UGT2B15

GOBP_ORGANIC_HYDROXY_COMPOUND_TRANSPORT	GOBP_ORGANIC_HYDROXY_COMPOUND_TRANSPORT	4.86E-05	PNLIP/SYT4/APOA2/CARTPT/CEL/VIP/APOA1/APOC3/AGTR1/ADIPOQ/KCNB1/ABCA8/CES1/HTR2A/APOB/SLC18A2/CNR1/SOAT2/GPM6B/CXCL12/AGT/CD36/CHGA/P2RX1/CAV1/SELENOM/RBP4/AQP10/PRKN/APOA4/SHH/DAB2/SLC16A7/SYT11/MIR27B/SLC10A4/OXTR/SNCG/SLC29A4/RAB3B/ABCA1/MTTP/ABCC2/SLC18A1/CLU/GABBR1/SLC10A6/PTCH1/SYT1/ITGB3/ADORA3/CHRNB2/P2RY1/PLTP/SLC5A12/ENPP7/LRP1/SYT5/MIR613/OXT

GOBP_REGULATION_OF_CALCIUM_ION_TRANSMEMBRANE_TRANSPORT	GOBP_REGULATION_OF_CALCIUM_ION_TRANSMEMBRANE_TRANSPORT	4.98E-05	PLA2G1B/CASQ2/PLP1/ATP1A2/PLN/ADCYAP1R1/CASQ1/JPH2/ANK2/AKAP6/F2/RGN/DMD/CACNA1C/P2RX2/TRPC1/HRC/HSPA2/PRKD1/CACNB2/P2RX1/CAV1/GEM/STAC/HPCA/SLC8A1/PKD2/BDKRB1/CACNA2D1/REM1/SLN/PRNP/RRAD/RYR2/CLIC2/THY1/JPH3/ITGB3

GOBP_MRNA_EXPORT_FROM_NUCLEUS	GOBP_MRNA_EXPORT_FROM_NUCLEUS	5.02E-05	PCID2/NXF1/RBM8A/IWS1/YTHDC1/MAGOHB/NUP155/AGFG1/NUP107/NXT1/AKAP8L/DDX19B/NUP133/POLDIP3/ALKBH5/SMG6/NUP214/NCBP3/MAGOH/PABPN1/TPR/SETD2/SMG7/RBM15B/THOC6/SMG1/NUP160/SMG5/SRSF3/CHTOP/POLR2D/NCBP1/NUP85/NSUN2/EIF4A3/NUP93/UPF1/HNRNPA2B1/CASC3/MCM3AP/NUP88/GLE1/THOC3/ALYREF/DDX39A/NXF3

GOMF_METALLOPEPTIDASE_ACTIVITY	GOMF_METALLOPEPTIDASE_ACTIVITY	5.03E-05	CPA1/CPA2/ADAM33/CPXM2/CPE/PAPPA2/ADAMTS8/ADAMTS18/ADAMTS16/ADAMTSL3/ADAM23/MMP8/CPN1/DPEP3/PRSS2/ADAMTS1/TLL1/CPA3/MMP3/TRABD2B/CLCA1/ADAMTS3/XPNPEP2/MMP24/MMP2/ADAMTSL1/MMP7/MMP19/NAALAD2/MMP16/CPXM1/TRHDE/DPEP1/ADAMTS10/MMP23B/CPA4/PAPLN/CPA6/ENPEP/AEBP1/VASH2/PAPPA/ADAMTS9
GOBP_MUSCLE_CELL_MIGRATION	GOBP_MUSCLE_CELL_MIGRATION	5.08E-05	ADIPOQ/VTN/TACR1/MYOCD/THBS4/SLIT2/IGF1/CYP1B1/DDR2/NR4A3/AGT/TPM1/PRKG1/ADAMTS1/LPAR1/SERPINE1/SMO/IGFBP5/CCN3/PARVA/PDGFD/CCN4/SEMA6D/PLEKHO1/HAS2/MEF2C/TAFA5/ITGB3/PDGFRB/LRP1/NDRG4/NRP1

GOBP_REGULATION_OF_PHOSPHOLIPASE_ACTIVITY	GOBP_REGULATION_OF_PHOSPHOLIPASE_ACTIVITY	5.11E-05	PLA2G1B/AGTR1/ADCYAP1R1/HTR2A/PLA2G5/HTR2B/FGF2/AGT/EDNRA/RGS2/LPAR1/FGFR1/CCN1/NMUR1/HPCA/ESR1/AVPR1A/PDGFRA/SELE/RASGRP4/KIT/PDGFRB/LRP1/P2RY12/FGFR2
GOBP_POSITIVE_REGULATION_OF_OSTEOBLAST_DIFFERENTIATION	GOBP_POSITIVE_REGULATION_OF_OSTEOBLAST_DIFFERENTIATION	5.22E-05	IGF1/SFRP2/FERMT2/DDR2/FGF2/GLI3/GDF10/PRKD1/HGF/CCN1/WWTR1/TMEM119/IL6/NELL1/FFAR4/WNT10B/CCN4/PDLIM7/ILK/BMPR1B/MEF2C/SCUBE3/CTHRC1/IL6ST/ACVR1/NPPC/FAM20C/SOX11/BMP6

GOCC_LUMENAL_SIDE_OF_MEMBRANE	GOCC_LUMENAL_SIDE_OF_MEMBRANE	5.29E-05	HLA-DPB1/HSPA8/HM13/HLA-H/SPPL2B/TAPBP/CALR/HLA-DPA1/CANX/HLA-E/BCAP31/HLA-DRA/HLA-B/HLA-A/HLA-DQA2/HLA-DQA1/HLA-DRB5/CD74/HLA-F/HLA-C/BDH1/HLA-DRB1/HLA-DQB1/HLA-DQB2/AQP2
GOBP_NEGATIVE_REGULATION_OF_SMOOTH_MUSCLE_CELL_PROLIFERATION	GOBP_NEGATIVE_REGULATION_OF_SMOOTH_MUSCLE_CELL_PROLIFERATION	5.52E-05	VIP/OGN/CNN1/ADIPOQ/MYOCD/MIR145/NPR3/APOD/VIPR2/TPM1/PRKG1/TGFB3/NPR1/EFEMP2/IGFBP5/PTGIR/MEF2C/TAFA5

GOBP_AMIDE_TRANSPORT	GOBP_AMIDE_TRANSPORT	5.55E-05	GCG/UCN3/CARTPT/CELA2A/GHRH/VIP/TACR2/SFRP1/NEUROD1/NNAT/KCNB1/FGA/ADCYAP1/ADCY5/CPE/SCT/KCNA5/PTPRN/FGG/GRP/SLC38A3/CNR1/GIP/CHGA/RFX6/NKX6-1/S100A8/RBP4/ABCB1/IL6/AQP10/PRKN/NR1H4/SNAP25/FOLR2/ISL1/FFAR4/CCN3/UPK3A/GJA1/SLC14A1/GPR27/ABCA1/MTTP/KLF7/PDPN/FGB/NLGN2/BLK/GABBR1/SLC16A2/GPLD1/MYRIP/SLC16A10/PLTP/GPER1/SLC46A1
GOCC_MHC_PROTEIN_COMPLEX	GOCC_MHC_PROTEIN_COMPLEX	5.58E-05	HLA-H/HLA-DPA1/HLA-DMB/HLA-E/HLA-DOB/HLA-DRA/HLA-B/HLA-A/HLA-DQA2/HLA-DQA1/HLA-DRB5/CD74/HLA-F/HLA-C/HLA-DRB1/HLA-DOA/HLA-DQB1/HLA-DQB2/HLA-DMA

GOBP_CHROMOSOME_LOCALIZATION	GOBP_CHROMOSOME_LOCALIZATION	5.59E-05	SPDL1/CHMP6/EML3/CDC23/SEH1L/KPNB1/KAT5/CHMP2B/NUP62/PDCD6IP/RAB11A/CHMP7/CHMP1A/VPS4B/LMNA/NDE1/HNRNPU/NUF2/CHMP5/ZW10/PINX1/KATNB1/PSRC1/MAP1S/AURKB/NDC80/SPAG5/KIF14/RRS1/NUDC/KIFC1/CENPF/INCENP/KIF18A/DLGAP5/KIF22/CHMP4C/CCNB1/KIF2C/CENPE/CDCA5/BIRC5/CEP55/CDCA8/CDT1
GOBP_NEGATIVE_REGULATION_OF_SYNAPTIC_TRANSMISSION	GOBP_NEGATIVE_REGULATION_OF_SYNAPTIC_TRANSMISSION	5.85E-05	SYT4/BCHE/GRIK3/ADIPOQ/KCNB1/HTR2A/NLGN4X/MAPT/CNR2/SORCS2/PRRT1/PRKN/SYT11/LRRK2/CELF4/GABBR1/PCDH17/ARC

GOCC_METHYLTRANSFERASE_COMPLEX	GOCC_METHYLTRANSFERASE_COMPLEX	5.97E-05	NCOA6/KANSL1/SNRPE/TAF6/WDR82/WTAP/METTL14/KDM6A/CBLL1/CLNS1A/HDAC9/CHD8/TAF1/BOD1/SNRPD2/TRMT61A/TRMT10C/KDM6B/TAF4/SNRPD3/SETD1B/E2F6/METTL3/RIOK1/RBBP7/SNRPG/TAF9/TRMT6/VIRMA/MAX/RAMAC/ASH2L/SUZ12/SENP3/RBBP5/TRMT61B/PRPF31/ERH/RBM15B/AEBP2/TEX10/HSD17B10/SNRPF/PELP1/PRDM4/LAS1L/EED/HDAC2/KMT2B/SETD1A/SNRPB/RUVBL1/RUVBL2/KMT2D/MEN1/MCRS1/SNRPD1/PAXIP1/WDR4/RBBP4/PAGR1/WDR5/INO80C/WDR77/RBM15/PRMT1/HCFC1/CXXC1/EZH2/PRMT5
GOBP_RELEASE_OF_SEQUESTERED_CALCIUM_ION_INTO_CYTOSOL_BY_ENDOPLASMIC_RETICULUM	GOBP_RELEASE_OF_SEQUESTERED_CALCIUM_ION_INTO_CYTOSOL_BY_ENDOPLASMIC_RETICULUM	5.99E-05	CASQ2/ATP1A2/PLN/CASQ1/ANK2/AKAP6/DMD/CACNA1C/HRC/SLC8A1/RYR2/CLIC2

GOMF_MHC_PROTEIN_COMPLEX_BINDING	GOMF_MHC_PROTEIN_COMPLEX_BINDING	6.02E-05	TAPBPL/HLA-DPB1/HSPA8/HSP90AA1/KLRC1/KLRD1/CYRIB/HLA-DPA1/YWHAE/HLA-DMB/HLA-DOB/HLA-DRA/KLRC2/HLA-DQA2/ANXA11/HLA-DQA1/CD8A/HLA-DRB5/CD74/HLA-DRB1/HLA-DOA/HLA-DQB1/HLA-DQB2/HLA-DMA/PKM/ATP1B1
GOMF_VOLTAGE_GATED_CATION_CHANNEL_ACTIVITY	GOMF_VOLTAGE_GATED_CATION_CHANNEL_ACTIVITY	6.07E-05	KCNA1/KCNB1/KCNMA1/KCNK3/GRIN2A/KCNA5/ABCC9/KCNK2/KCNH6/KCNT2/CACNA1C/CACNG4/CNR1/CACNA1H/KCNJ8/CACNB2/KCNE4/KCNH2/NCS1/PKD2/CACNA2D3/CACNA2D1/CACNA1A/KCNG1/SNAP25/KCNQ3/KCND1/KCNS1/KCND2

GOBP_BONE_MINERALIZATION	GOBP_BONE_MINERALIZATION	6.08E-05	AHSG/CER1/OMD/MGP/IGF1/GPC3/ROR2/DDR2/PTN/GREM1/PTH1R/ASPN/GPM6B/RSPO2/TGFB3/CCN1/SLC8A1/TMEM119/COMP/OSR1/CLEC3B/ADRB2/NELL1/WNT10B/SLC24A3/PTHLH/S1PR1/LOX/BMPR1B/KL/MEF2C/LTBP3/COL1A2/TWIST1
GOMF_VOLTAGE_GATED_CHANNEL_ACTIVITY	GOMF_VOLTAGE_GATED_CHANNEL_ACTIVITY	6.14E-05	KCNA1/SCN7A/KCNB1/KCNMA1/KCNK3/GRIN2A/KCNA5/ABCC9/KCNK2/KCNH6/KCNT2/KCNIP1/CACNA1C/CACNG4/CNR1/CACNA1H/SCN4B/KCNJ8/CACNB2/KCNE4/KCNH2/NCS1/SCN3A/PKD2/CACNA2D3/CACNA2D1/CACNA1A/CLIC4/KCNG1/SNAP25/KCNQ3/KCND1/KCNS1/KCND2/CLIC2

GOCC_PLASMA_MEMBRANE_RAFT	GOCC_PLASMA_MEMBRANE_RAFT	6.16E-05	PRTN3/ATP1A2/CHRNA3/ADCYAP1R1/KCNMA1/PTGIS/SORBS1/BVES/AKAP6/KCNA5/HTR2A/CDH2/TRPC4/ADD2/GASK1A/CD36/ADRA1B/ATP2B4/CAVIN1/CAV1/CAVIN2/MAL/SMO/DLC1/LRRK2/EHD2/HAS2/KCND2/TFPI/PTCH1/SELE/CHRNB2/MS4A4A
GOBP_REGULATION_OF_VASCULATURE_DEVELOPMENT	GOBP_REGULATION_OF_VASCULATURE_DEVELOPMENT	6.21E-05	NPPB/HSPB6/SFRP1/APOH/THBS4/ANGPTL7/CMA1/MIR145/PTGIS/ISM1/SFRP2/ADGRB3/BMPER/CYP1B1/SMOC2/FGF2/TNN/GREM1/DCN/SEMA3E/PRKD1/AGT/RECK/AKT3/ATP2B4/ADAMTS1/THBS1/CAMP/SERPINF1/CX3CR1/HGF/NODAL/NPR1/TGFB2/RHOJ/ITGA5/IL6/PRKCB/SERPINE1/CYSLTR1/EMILIN1/CCBE1/KRT1/MIR27B/ISL1/FOXC2/SEMA6A/CCL11/FGF1/ADGRA2/RHOB/RAMP2/CXCR2/HSPB1/GPNMB/CLDN5/TEK/VASH2/MIR222/TNFSF12/TAFA5/SPARC/ADAMTS9/PTPRM/TWIST1/THBS2/ITGB3/VEGFC/ADGRB2/ADGRB1/HOXA5/CXCR4/SULF1/CD34/GAB1/ECSCR/NRP1/PDE3B/APLNR/RRAS/TIE1

GOBP_POSITIVE_REGULATION_OF_SYNAPTIC_TRANSMISSION	GOBP_POSITIVE_REGULATION_OF_SYNAPTIC_TRANSMISSION	6.52E-05	RELN/TACR2/TACR1/SCGN/GRIN2A/SLC8A2/NOG/IGSF11/ROR2/PTN/MIR320C2/CACNG4/CNR1/NLGN1/LAMA2/ADCY1/GIP/CACNB2/NFATC4/CX3CR1/SERPINE2/CLSTN2/PRRT1/FAM107A/MIR342/CCL2/SNAP25/MPP2/RETN/OXTR/NLGN3/TSHZ3/MIR320D1/DLG4/PRNP/NLGN2/SYT1/RASGRF2/MIR95/GPER1/ARC

GOBP_SENSORY_ORGAN_DEVELOPMENT	GOBP_SENSORY_ORGAN_DEVELOPMENT	6.61E-05	MAB21L2/COL2A1/HAND2/KERA/NEUROD1/KCNK3/CHRDL1/ANGPTL7/FGF10/ASCL1/GPM6A/FAT3/ALDH1A2/CRYAB/TMOD1/SALL2/BNC2/SIX2/ADAMTS18/NOG/KCNK2/EFEMP1/NKX3-2/BMPER/CYP1B1/PLPPR4/CACNA1C/FGF2/FRZB/TUB/CDON/WT1/GLI3/ZEB1/MFAP5/NTN1/COL8A1/SLC17A7/SDK2/ITGA8/WNT9A/VSTM4/TGFB3/PRPH2/ATP2B4/TENM3/LAMA1/SERPINF1/SOBP/EDNRA/NDP/SMARCD3/LIN7A/CRB2/TBX18/TGFB2/MEIS1/RHOJ/HPCA/FBN1/RBP4/CYTL1/CDH23/HOXA2/SHH/CLIC4/PDGFRA/OSR1/PBX1/TMEM132E/PRRX1/SLITRK6/FOXF2/COL8A2/PLAAT1/PBX3/WNT2B/WNT10B/THRB/FOXC2/DCHS1/ATOH1/VIM/CC2D2A/CELF4/TCF15/MAF/BMPR1B/USH1G/MEIS3P1/THY1/INHBA/ALDH1A3/[illegible]

GOBP_REGULATION_OF_CALCIUM_ION_TRANSPORT	GOBP_REGULATION_OF_CALCIUM_ION_TRANSPORT	6.68E-05	PLA2G1B/GCG/CASQ2/PLP1/ATP1A2/PLN/MYLK/ADCYAP1R1/CASQ1/JPH2/ANK2/AKAP6/F2/RGN/DMD/RGS4/CACNA1C/P2RX2/TRPC1/CXCL12/HRC/HSPA2/PRKD1/CACNB2/AGT/P2RX1/CAV1/GEM/STAC/HPCA/SLC8A1/PKD2/BDKRB1/CACNA2D1/CCL2/REM1/SLN/PRNP/RRAD/RYR2/STC1/CLIC2/THY1/LILRA2/JPH3/ITGB3/CXCR4/GPER1/CACNG6/PDGFRB/STC2/BEST1
GOBP_DEOXYRIBONUCLEOTIDE_METABOLIC_PROCESS	GOBP_DEOXYRIBONUCLEOTIDE_METABOLIC_PROCESS	6.71E-05	MBD4/NEIL1/SMUG1/NUDT16/NT5C2/OGG1/GDA/DGUOK/NT5M/NEIL2/TDG/DPYD/CMPK2/NUDT15/GUK1/ADK/DCTD/NT5C/PNP/NTHL1/RRM2B/XDH/NUDT18/UNG/DTYMK/DCK/DUT/DCTPP1/RRM1/SHMT1/TYMP/ADA/TYMS/RRM2

GOBP_METAPHASE_PLATE_CONGRESSION	GOBP_METAPHASE_PLATE_CONGRESSION	6.79E-05	SPDL1/CHMP6/EML3/CDC23/SEH1L/KPNB1/KAT5/CHMP2B/NUP62/PDCD6IP/RAB11A/CHMP7/CHMP1A/VPS4B/HNRNPU/NUF2/CHMP5/ZW10/PINX1/PSRC1/MAP1S/AURKB/NDC80/SPAG5/KIF14/RRS1/NUDC/KIFC1/CENPF/INCENP/KIF18A/KIF22/CHMP4C/CCNB1/KIF2C/CENPE/CDCA5/BIRC5/CEP55/CDCA8/CDT1

GOBP_CELL_GROWTH	GOBP_CELL_GROWTH	6.82E-05	SYT4/NPPB/AGTR1/SFRP1/MYOCD/EPHA7/FHL1/RERG/AKAP6/F2/CRYAB/SLIT2/DACT3/PTCH2/PAPPA2/IGF1/SFRP2/PI16/SLIT3/MAP1B/RGS4/NGF/TNC/TNN/FRZB/GREM1/PLXNA4/NDN/MEG3/IGFBP1/CXCL12/WT1/SEMA3E/FBLN5/NTN1/PRSS2/AGT/MAPT/RND2/ROS1/LPAR3/NKX6-1/S100A8/EDNRA/FGF13/RGS2/RASGRP2/NPR1/TGFB2/SERPINE2/L1CAM/NRP2/IGFBP4/BDKRB1/S100A9/PRKN/SH3GL2/WFDC1/FLRT1/FAM107A/AVPR1A/SPOCK1/DAB2/FN1/IGFBP7/SPART/IGFBP5/CCN3/NLGN3/CRABP2/GJA1/SORBS2/SEMA6A/EPB41L3/TMEM108/SEMA6D/SOX17/ISLR2/HSPA1A/NRCAM/WASF1/INHBA/NANOS1/SEMA6C/SYT1/SEMA3G/DCLK1/MAP2/SEMA5B/SEMA3D/CXCR4/TSPYL2/TEAD1
GOCC_TRIGLYCERIDE_RICH_PLASMA_LIPOPROTEIN_PARTICLE	GOCC_TRIGLYCERIDE_RICH_PLASMA_LIPOPROTEIN_PARTICLE	6.86E-05	APOA2/APOA1/APOC3/APOH/APOB/VLDLR/APOA4/LPL
GOBP_MEMBRANE_REPOLARIZATION	GOBP_MEMBRANE_REPOLARIZATION	6.95E-05	CASQ2/ATP1A2/ANK2/AKAP6/KCNA5/KCNH6/FLNA/ATP1B2/SCN4B/KCNJ8/KCNE4/KCNH2/CAV1/CACNA2D1

GOCC_ACTIN_FILAMENT_BUNDLE	GOCC_ACTIN_FILAMENT_BUNDLE	6.97E-05	SYNPO2/LDB3/PGM5/MYLK/MYL9/SORBS1/CRYAB/PDLIM3/ACTA2/FERMT2/FLNA/ACTA1/TPM1/LPP/PALLD/PDLIM4/MYH10/FAM107A/SYNPO/PDLIM7/ILK/TEK/ACTN1
GOBP_GOLGI_VESICLE_TRANSPORT	GOBP_GOLGI_VESICLE_TRANSPORT	7.01E-05	CORO7/LMAN1/GOLT1A/RAB2A/SPAST/TRIP11/COPB2/SAR1B/TEX261/TFG/COG3/CSNK1D/DOP1B/MPPE1/RAB6A/KIF13A/AP2A1/KLHL12/LLGL1/SEC23IP/CCDC93/VAPB/TRAPPC12/EXOC2/COG6/VPS52/ARF4/TRAPPC9/COPA/STX6/SCAP/PGAP1/TMED2/MIA3/COG5/CUL3/ERGIC1/IER3IP1/ARCN1/COG8/KIF1C/ARF3/COPB1/GOLGA4/GBF1/TRAPPC4/GOSR1/ANKFY1/SCAMP3/TRAPPC11/BET1L/COG1/GGA3/TMED4/EXOC1/RAB10/VPS13A/USO1/ARFGAP2/SEC22B/ARFGAP3/MYO18A/ARF5/ARL1/COG4/GOLGA2/KDELR1/MIA2/TRAPPC2L/PDCD6/COPG2/STX5/SEC24D/KIF16B/TRAPPC10/VPS13C/DOP1A/RAB14/TMEM115/TMED5/YKT6/PKDCC/KDELR2/SEC24A/TMED7/SLC10A7/SCYL1/COG2/INSIG1/YIF1A/AP3D1/SEC31B/CO[illegible]

GOBP_REGULATION_OF_PHAGOCYTOSIS	GOBP_REGULATION_OF_PHAGOCYTOSIS	7.14E-05	PRTN3/AZU1/APOA2/AHSG/APOA1/ADIPOQ/PLA2G5/TUB/PTX3/CD36/COLEC11/PIP4P2/C4A/FCN1/C4B/CCL2/RAB31/SYT11
GOBP_REGULATION_OF_CALCIUM_ION_TRANSMEMBRANE_TRANSPORTER_ACTIVITY	GOBP_REGULATION_OF_CALCIUM_ION_TRANSMEMBRANE_TRANSPORTER_ACTIVITY	7.18E-05	CASQ2/ATP1A2/PLN/CASQ1/JPH2/ANK2/AKAP6/RGN/DMD/HRC/HSPA2/CACNB2/GEM/STAC/HPCA/PKD2/CACNA2D1/REM1/SLN/RRAD/RYR2/CLIC2/JPH3

GOBP_GLIAL_CELL_DIFFERENTIATION	GOBP_GLIAL_CELL_DIFFERENTIATION	7.17E-05	NKX2-2/SOX10/PLP1/RELN/MYOC/VTN/FGF10/ASCL1/F2/GAP43/DNER/NOG/CDH2/ROR2/CNTN1/PTN/FGF2/TRPC4/DAB1/DAAM2/GLI3/IL33/WASF3/LGI4/CNTNAP1/GPR17/MAPT/NKX6-1/S100A8/LPAR1/MAL/ASPA/SERPINE2/MMP24/KLF15/IL6/S100A9/TSPAN2/SHH/DUSP15/SMO/ID4/VIM/POU3F1/ILK/TENM4/MXRA8/PTPRZ1/CLU/PRDM8/CXCR4/ROR1/IL6ST/IL34/LEF1/LRP1/PLPP3
GOMF_PEPTIDE_HORMONE_BINDING	GOMF_PEPTIDE_HORMONE_BINDING	7.26E-05	ADCYAP1R1/GLP2R/NPR3/GHR/CCKAR/PTH1R/VIPR2/LEPR/NPR1/AVPR1A/SCTR/CCKBR/GLP1R/OXTR/RAMP2/NPR2/INHBA/ACVR1/EDNRB/PRLR/RAMP1/PTH2R

GOBP_NUCLEOTIDE_EXCISION_REPAIR	GOBP_NUCLEOTIDE_EXCISION_REPAIR	7.38E-05	RNF111/ERCC2/USP45/RPA3/SMARCD1/PNKP/GTF2H2/RPA2/BRD7/GTF2H2C/DDB1/RAD23B/SMARCC1/ACTL6A/KAT5/SMARCC2/XPC/MMS19/POLA1/XAB2/CUL4A/FAN1/SMARCB1/POLL/HMGN1/RPA1/UVSSA/POLB/USP7/SLX4/ERCC5/SLC30A9/GTF2H3/NTHL1/BRCA2/FANCC/BCL7A/RAD23A/SMARCD2/POLD3/SMARCE1/POLE/SMARCA4/DDB2/TP53/POLD1/BRIP1

GOBP_NEURON_PROJECTION_GUIDANCE	GOBP_NEURON_PROJECTION_GUIDANCE	7.54E-05	RELN/NGFR/EPHA7/NRXN3/BOC/SLIT2/FLRT2/GAP43/NEXN/NFASC/NOG/GFRA3/ARHGEF25/SLIT3/EPHA3/CNTN1/PLXNA4/LAMA2/CXCL12/GLI3/SEMA3E/NTN1/CNTN4/VSTM2L/CHL1/LAMA1/EDNRA/NCAM1/PALLD/L1CAM/NRP2/FEZ1/HOXA2/SHH/SMO/KIF5A/ISL1/EFNB3/GDF7/DPYSL5/ATOH1/SEMA6A/SEMA6D/DPYSL4/BMPR1B/KLF7/NRCAM/ANOS1/PTPRM/PTCH1/SEMA6C/SEMA3G/SEMA5B/SEMA3D/ROBO1/ENAH/GLI2/SEMA3C/NRP1/CRMP1

GOBP_EPIDERMIS_DEVELOPMENT	GOBP_EPIDERMIS_DEVELOPMENT	7.63E-05	CASP3/LGR4/PPARD/SPRR2D/SOX21/EVPL/USH1C/SLC44A4/HDAC1/JAG1/NAB1/HOXB13/SOX9/EZH2/TFDP1/OVOL3/S100A7/KEAP1/SCRIB/CDH3/MSX2/ADAM9/ESRP1/HLA-DRB1/TPRN/DNASE1L2/KLK14/COL17A1/STS/GRHL2/HPSE/KRT17/ST14/KRT80/SPINT1/LAMC2/PLS1/KRT6B/VDR/SFN/AGPAT2/PLAAT4/KDF1/FLNB/FOXQ1/LAMB3/FST/FOXC1/KLK5/OVOL2/KRT7/EDAR/ZBED2/EXPH5/FERMT1/POU2F3/APCDD1/FA2H/SOSTDC1/CST6/EPHA2/KRT8/LAMA3/KCNQ1/ABCA12/GJB5/KRT16/HES5/SPRR2E/KLK7/KRT81/DKK4/FGF20/KRTDAP/LCE3E/LCE3D/KRT27/SPRR2B/SPRR2G
GOBP_POSITIVE_REGULATION_OF_POTASSIUM_ION_TRANSPORT	GOBP_POSITIVE_REGULATION_OF_POTASSIUM_ION_TRANSPORT	7.70E-05	KCNA1/ACTN2/KCNK3/FHL1/KCNMB1/ANK2/AKAP6/FLNA/ATP1B2/KCNH2
GOBP_PROTEIN_LIPID_COMPLEX_SUBUNIT_ORGANIZATION	GOBP_PROTEIN_LIPID_COMPLEX_SUBUNIT_ORGANIZATION	7.77E-05	MPO/APOA2/APOA1/APOC3/AGTR1/APOB/PLA2G5/SOAT2/AGT/NR1H4/APOA4/ABCA1/LPL/MTTP

GOBP_REGULATION_OF_CARDIAC_MUSCLE_CONTRACTION_BY_REGULATION_OF_THE_RELEASE_OF_SEQUESTERED_CALCIUM_	GOBP_REGULATION_OF_CARDIAC_MUSCLE_CONTRACTION_BY_REGULATION_OF_THE_RELEASE_OF_SEQUESTERED_CALCIUM_ION	7.99E-05	CASQ2/ATP1A2/PLN/ANK2/DMD/CACNA1C/HRC/SLC8A1/RYR2/CLIC2
GOBP_REGULATION_OF_VASOCONSTRICTION	GOBP_REGULATION_OF_VASOCONSTRICTION	8.10E-05	ATP1A2/AGTR1/FGA/TACR1/KCNA5/HTR2A/FGG/ADRA1D/SMTNL2/AGT/ADRA1B/P2RX1/CAV1/MMP2/AVPR1A/CYSLTR1/OXTR/AVPR2/FGB/EDN2/SNTA1/HRH2/CHRM3/TBXA2R/GJA5/F2R/ADM/ACE2
GOBP_POSITIVE_REGULATION_OF_TRANSPORTER_ACTIVITY	GOBP_POSITIVE_REGULATION_OF_TRANSPORTER_ACTIVITY	8.19E-05	KCNA1/RELN/ACTN2/ADIPOQ/CASQ1/KCNK3/JPH2/ANK2/AKAP6/RGN/DMD/ATP1B2/CACNG4/HSPA2/CACNB2/EDNRA/STAC/ABCB1/PKD2/CACNA2D1/CCL2/ADRB2/NLGN3

GOBP_MICROTUBULE_CYTOSKELETON_ORGANIZATION_INVOLVED_IN_MITOSIS	GOBP_MICROTUBULE_CYTOSKELETON_ORGANIZATION_INVOLVED_IN_MITOSIS	8.24E-05	PDCD6IP/CHEK2/RAB11A/CHMP7/INTS13/KIF2A/CHMP1A/TPX2/CEP192/VPS4B/GPSM2/NDE1/HNRNPU/NUF2/CHMP5/NEK2/RAN/CKAP5/ZW10/SASS6/TTK/STMN1/SMC1A/MCPH1/PSRC1/PRC1/INSC/MAP1S/FAM110A/CCSAP/PCNT/VCP/AURKB/NDC80/KIF23/HTT/WRAP73/PTPA/AURKA/CENPA/MZT1/TNKS/SPC25/NUDC/KIFC1/WDR62/PLK2/BORA/INCENP/KIF4A/RACGAP1/PARP3/DLGAP5/CDC20/CHMP4C/KIFC2/CCNB1/ESPL1/TACC3/MAD2L1/CENPE/MISP/PLK1/BIRC5/CENPH/CDCA8/KIF15/RCC1/KIF11/STIL/SAPCD2

GOBP_ACTIN_MEDIATED_CELL_CONTRACTION	GOBP_ACTIN_MEDIATED_CELL_CONTRACTION	8.29E-05	ATP1A2/PLN/CTNNA3/ANK2/KCNA5/ABCC9/ACTA2/FLNA/CACNA1C/SGCD/SCN4B/KCNJ8/CACNB2/KCNE4/TPM1/KCNH2/ACTC1/GJC1/CAV1/FGF13/CACNA2D1/FRMD6/PARVA/GJA1/PDPN/RYR2/STC1/FGF12/EPDR1/GSN/SCN3B/SCN1B/SNTA1/GJA5/KCNJ5/LIMCH1
GOMF_MHC_CLASS_II_PROTEIN_COMPLEX_BINDING	GOMF_MHC_CLASS_II_PROTEIN_COMPLEX_BINDING	8.33E-05	HLA-DPB1/HSPA8/HSP90AA1/HLA-DPA1/YWHAE/HLA-DMB/HLA-DOB/HLA-DRA/HLA-DQA2/ANXA11/HLA-DQA1/HLA-DRB5/CD74/HLA-DRB1/HLA-DOA/HLA-DQB1/HLA-DQB2/HLA-DMA/PKM/ATP1B1

GOBP_KERATINIZATION	GOBP_KERATINIZATION	8.56E-05	KRT17/KRT80/KRT6B/SFN/KLK5/KRT7/KRT8/ABCA12/KRT16/SPRR2E/KRT81/LCE3E/LCE3D/SPRR2B/SPRR2G
GOBP_NEGATIVE_REGULATION_OF_WOUND_HEALING	GOBP_NEGATIVE_REGULATION_OF_WOUND_HEALING	8.58E-05	APOH/VTN/FGA/F2/FGG/ADAMTS18/FGF2/PHLDB2/SERPINF2/MMRN1/PRKG1/THBS1/SERPINE2/WFDC1/SERPINE1/PDGFRA/PROS1/KRT1/CLDN19/THBD/FAP/FGB/TAFA5/SERPING1/TFPI
GOBP_CHOLESTEROL_EFFLUX	GOBP_CHOLESTEROL_EFFLUX	8.65E-05	APOA2/APOA1/APOC3/ADIPOQ/ABCA8/CES1/APOB/SOAT2/CAV1/APOA4/SHH/MIR27B/ABCA1/PTCH1/PLTP/LRP1/MIR613

GOBP_REGULATION_OF_NEUROTRANSMITTER_TRANSPORT	GOBP_REGULATION_OF_NEUROTRANSMITTER_TRANSPORT	8.87E-05	SYT4/CHRM2/ATP1A2/TACR2/CHRNA3/HTR2A/CNR1/NLGN1/GPM6B/ADCY1/CACNB2/CHRNB4/P2RX1/PRKCB/PRKN/SYT11/LRRK2/SNCG/RAB3B/MEF2C/APBA1/SYT1/ITGB3/SYP/P2RY1/GPER1/SNCAIP/BACE1/PFN2
GOCC_PROTEASOME_COMPLEX	GOCC_PROTEASOME_COMPLEX	8.96E-05	PSMD1/PSMC6/PSMD4/PSMD10/PSMA1/UBE3A/USP14/PSMA7/PSME3/PSMB5/PSMD5/PSMD6/PSMB4/TXNL1/PSMC5/PSMF1/PSMB1/PSMA3/PSMD13/PSMB6/RAD23B/PSMD7/PSMC3/PSMC2/PSMC4/PSMD2/PSME4/PSMD9/PSMB3/UBQLN1/PSMD8/UCHL5/PSMC1/PSMB7/PSMA6/PSMD12/PSMB2/PSMD14/ECPAS/PSMA4/UBE3C/ADRM1/RAD23A/PSMA5/VCP/PSME1/PSMB9/PSME2/PSMB8/PSMD3/PSMB10

GOBP_VASOCONSTRICTION	GOBP_VASOCONSTRICTION	9.23E-05	ATP1A2/AGTR1/FGA/TACR1/KCNA5/HTR2A/ACTA2/FGG/HTR2B/ADRA1D/SMTNL2/AGT/ADRA1B/P2RX1/CAV1/EDNRA/SLC8A1/MMP2/COMP/AVPR1A/CYSLTR1/OXTR/AVPR2/FGB/EDNRB/EDN2/SNTA1/HRH2/CHRM3/TBXA2R/GJA5/SLC6A4
GOBP_CHROMOSOME_CONDENSATION	GOBP_CHROMOSOME_CONDENSATION	9.29E-05	ACIN1/SMC5/WAPL/H3-3B/H1-10/TENT4A/H3-3A/CHMP1A/KMT5A/AKAP8/H1-2/NCAPH2/AIFM1/SMC4/NCAPD2/MCPH1/H1-3/SMC2/CDK1/NCAPG2/DFFB/NCAPH/NCAPD3/CDCA5/PLK1/TOP2A/NCAPG/H1-4/ERN2/H1-5

GOBP_POSITIVE_REGULATION_OF_CELL_SUBSTRATE_ADHESION	GOBP_POSITIVE_REGULATION_OF_CELL_SUBSTRATE_ADHESION	9.30E-05	APOA1/SFRP1/MYOC/VTN/FGA/CCDC80/FGG/ABI3BP/FERMT2/LIMS2/NPY/FLNA/VIT/COL26A1/COL8A1/CD36/CCL21/FOXF1/ECM2/CCN1/ITGA5/EMILIN1/DAB2/FN1/EFEMP2/FBLN2/COL16A1/ILK/HAS2/FGB/NID1/TEK/THY1/ITGB3/CCR7/MYADM/NDNF/NRP1/RRAS/EDIL3/EGFLAM
GOBP_CELLULAR_EXTRAVASATION	GOBP_CELLULAR_EXTRAVASATION	9.39E-05	ELANE/PRTN3/AZU1/JAM2/JAM3/LYVE1/CXCL12/ADD2/CCL21/IL1R1/CX3CR1/SELP/ITGA1/CCL2/PDGFD/SELL/BST1/THY1/RIPOR2/SELE/CD99L2
GOBP_REGULATION_OF_RYANODINE_SENSITIVE_CALCIUM_RELEASE_CHANNEL_ACTIVIT	GOBP_REGULATION_OF_RYANODINE_SENSITIVE_CALCIUM_RELEASE_CHANNEL_ACTIVITY	9.46E-05	CASQ2/PLN/JPH2/AKAP6/DMD/HRC/PKD2/CLIC2/JPH3

GOBP_REGULATION_OF_MITOTIC_CELL_CYCLE_PHASE_TRANSITION	GOBP_REGULATION_OF_MITOTIC_CELL_CYCLE_PHASE_TRANSITION	9.64E-05	CUL3/ANAPC4/ECD/AMBRA1/MAD2L1BP/CDK2/CDK10/ERCC2/UIMC1/RPS27L/RBL1/RPL26/NEK6/APPL2/FBXO7/TPR/MAD2L2/SMARCD1/RPA2/BRD7/CRADD/DDB1/CDK7/RPTOR/NABP2/WEE1/CCND3/SPDL1/CDK5RAP3/CDC25B/BID/CPSF3/CDC23/SMARCC1/NAE1/TMOD3/ACTL6A/ZFP36L2/RB1/MEPCE/ANAPC15/NEK11/ANAPC5/SMARCC2/XPC/ATAD5/PTPN6/CUL4A/TRIP13/SMARCB1/CHEK2/RAB11A/BRCC3/BRCA1/EIF4G1/BTN2A2/VPS4B/INO80/TOPBP1/PML/NUF2/ANKRD17/MIIP/AKT1/BARD1/CDKN2C/EGFR/CDK2AP2/KLHL18/DONSON/MTBP/ZFYVE19/ZW10/PINX1/TRIAP1/RAD21/SASS6/CCNG1/BCL7A/KLF4/TAOK2/RINT1/TTK/RRM2B/PRKDC/KNTC1/PIDD1/XRCC3/FBXO5/FZR1/CASP2/ZWILCH/AURKB/NDC80/BUB1/SM[illegible]
GOMF_SNORNA_BINDING	GOMF_SNORNA_BINDING	0.000100979	NOP10/UTP25/NUDT5/NOP56/NUDT1/HEATR1/NUDT16L1/XRCC5/NUDT4/IMP3/NOP58/BMS1/DKC1/DDX21/DHX37/TSR1/BYSL/PRKDC/WDR3/TBL3/IMP4/GAR1/NHP2/RRP9/ISG20/NOLC1/NOP14

GOBP_CENTRIOLE_ASSEMBLY	GOBP_CENTRIOLE_ASSEMBLY	0.000101878	CDK5RAP2/CCP110/TRIM37/C2CD3/CCDC61/CEP135/CDK2/CCDC57/ALMS1/NUP62/CEP295/BRCA1/CEP192/PPP1R35/VPS4B/CEP152/E2F4/RTTN/RBM14/SASS6/KAT2A/CEP72/POC1B/CNTROB/NPM1/WDR62/PLK2/PLK4/MCIDAS/STIL/CCDC78
GOBP_REGULATION_OF_DNA_TEMPLATED_TRANSCRIPTION_ELONGATION	GOBP_REGULATION_OF_DNA_TEMPLATED_TRANSCRIPTION_ELONGATION	0.000101826	ELL/BRD4/CDK13/ERCC6/NELFE/MED30/CCNT2/CTNNB1/RECQL5/MED8/RNF168/MED11/HTATSF1/MED27/CDK12/SUPT16H/CCNT1/CCNK/TCERG1/VHL/LEO1/ZC3H4/SUPT5H/INTS12/MED14/MED25/WDR43/MED23/NCBP1/MED16/HMGN1/INTS13/EAF1/INTS7/AXIN1/INTS14/INTS9/HNRNPU/INTS8/MED15/INTS11/MED9/INTS1/INTS4/NELFCD/NELFB/GTF2F1/INTS5/MED26/MED1/CCAR2/SIRT6/MED18/NELFA/INTS2/EZH2/ALYREF/PWWP2B/INTS10/MED24/ELL3

GOBP_REGULATION_OF_DOUBLE_STRAND_BREAK_REPAIR_VIA_HOMOLOGOUS_RECOMBINATION	GOBP_REGULATION_OF_DOUBLE_STRAND_BREAK_REPAIR_VIA_HOMOLOGOUS_RECOMBINATION	0.000102169	ING3/ARID2/MAGEF1/ACTR2/ERCC6/YEATS4/TRRAP/RECQL5/SHLD2/SHLD3/TP53BP1/WAS/MAD2L2/EP400/SETD2/RIF1/RPA2/BRD8/MRGBP/PIAS4/ACTL6A/KAT5/SPIDR/RTEL1/FIGNL1/SMCHD1/HDGFL2/MMS19/FUS/RUVBL1/RUVBL2/KDM1A/FANCB/PARP1/UBQLN4/PPP4C/HELB/SIRT6/RBBP8/WRAP53/RAD51AP1/PARPBP/CGAS/TIMELESS/CHEK1/POLQ/RAD51/RMI2

GOBP_ENDOPLASMIC_RETICULUM_TO_GOLGI_VESICLE_MEDIATED_TRANSPORT	GOBP_ENDOPLASMIC_RETICULUM_TO_GOLGI_VESICLE_MEDIATED_TRANSPORT	0.000102526	SORL1/NRBP2/LMAN1/GOLT1A/RAB2A/SPAST/TRIP11/COPB2/SAR1B/TEX261/TFG/COG3/CSNK1D/MPPE1/KLHL12/SEC23IP/VAPB/TRAPPC12/ARF4/TRAPPC9/COPA/SCAP/PGAP1/TMED2/MIA3/CUL3/ERGIC1/IER3IP1/ARCN1/COPB1/GBF1/TRAPPC4/GOSR1/TRAPPC11/TMED4/USO1/SEC22B/GOLGA2/KDELR1/MIA2/TRAPPC2L/PDCD6/COPG2/STX5/SEC24D/TRAPPC10/TMED5/YKT6/KDELR2/SEC24A/TMED7/INSIG1/YIF1A/SEC31B/VTI1A/TMED9/PREB/WHAMM/KDELR3/BCAP31/ZW10/RAB1B/YIF1B/COPE/TMED3/RINT1/LMAN2/SEC13/VCP/STX18/COPG1/SEC24C/SEC23B/SEC16A/TMED1/HYOU1/CTAGE4/ANK1/MAPK15

GOCC_SPINDLE_POLE	GOCC_SPINDLE_POLE	0.0001032	VPS4A/CTNNB1/CUL3/TOPORS/HAUS1/PPP2CB/CEP104/NEK6/AAAS/UBXN2B/NUDCD2/BCCIP/SMC3/CEP128/SPDL1/RALBP1/CDC25B/TUBGCP5/GOLGA2/TUBG1/BIRC6/KAT5/CALM3/ALPK1/ALMS1/STAG1/NUP62/ODF2/CNTRL/FAM110C/RAB11A/BRCC3/KIF2A/TPX2/CKAP2/VPS4B/TOPBP1/HSF1/GPSM2/NDE1/HNRNPU/NEK2/CTDP1/CKAP5/ZW10/NUBP2/KATNB1/RAD21/KNSTRN/KNTC1/POC1B/SMC1A/PSRC1/SGO1/PRC1/RMDN3/DDX11/RASSF1/GIT1/TUBGCP4/TUBGCP2/FAM110A/HAUS8/AURKB/KIF20B/CKAP2L/CDC14B/ASPM/SPAG5/AURKA/TNKS/NPM1/TMEM201/WDR62/PLK2/RGS14/CENPF/DLGAP5/PLK4/CDC20/AUNIP/CCNB1/TACC3/MAD2L1/CEP85/MISP/PLK1/POC1A/KIF11/IRAG2
GOMF_TRIGLYCERIDE_LIPASE_ACTIVITY	GOMF_TRIGLYCERIDE_LIPASE_ACTIVITY	0.00010436	PNLIP/PNLIPRP1/CEL/PNLIPRP2

GOBP_SMOOTHENED_SIGNALING_PATHWAY	GOBP_SMOOTHENED_SIGNALING_PATHWAY	0.000104261	NKX2-2/SFRP1/FGF10/BOC/PTCH2/PTCHD1/NOG/GPC3/GAS1/CDON/GLI3/FOXF1/DZIP1/GLI1/NKX6-1/HHIP/EVC/SERPINE2/SHH/SMO/DHH/PRRX1/EVC2/WNT10B/CC2D2A/GPR161/RAB34/SCUBE1/PTCH1/GLIS2/DYNC2H1/PRKACB/GLI2/FGFR2/RORA/CIBAR1/FUZ/DCDC2/ARMC9

GOBP_FAT_CELL_DIFFERENTIATION	GOBP_FAT_CELL_DIFFERENTIATION	0.000103867	MIR483/SFRP1/ADIPOQ/FABP4/TPH1/SLC2A4/FGF10/HTR2A/LMO3/SFRP2/GDF6/FERMT2/FNDC5/MEDAG/CLIP3/ZFPM2/EBF2/RUNX1T1/ZBTB16/FRZB/NR4A3/TGFB1I1/GDF10/PID1/LGALS12/RGS2/FABP3/WWTR1/IL6/PDGFRA/STEAP4/ADRB2/MIR27B/FFAR4/DIO2/RETN/WNT10B/ID4/SNAI2/CCN4/DKKL1/IL11/LPL/RARRES2

GOMF_CARBOHYDRATE_BINDING	GOMF_CARBOHYDRATE_BINDING	0.000103654	REG3G/LGALS14/ASGR2/PRG4/VTN/REG1B/PPP1R3C/NPTX2/GALNT16/CHODL/CLC/CNTN1/PTN/COLEC12/GALNT15/GALNT8/FREM1/LAYN/PTX3/AMBP/CHI3L2/COLEC11/GALNT13/SIGLEC6/LGALS12/SELP/ENDOU/FCN1/ITLN1/CLEC2L/C4B/P4HA3/GALNT17/CD302/CLEC3B/KRT1/PLOD2/SELL/ASGR1/VCAN/FCER2/CLEC11A/SLC2A3/MRC2/CLEC1A/PPP1R3G/SELE/CLEC10A/LGALS1/CD248/CD34/CLEC14A/ADGRL3/CLEC2B/LGALS7B/CD207/ADGRL2/COL9A1/CD69/KLRG2/OLR1/CD93/GALNT14/PLA2R1/PKD1/MRC1/PYGL/KLRF1/ENG
GOCC_INTRINSIC_COMPONENT_OF_PRESYNAPTIC_MEMBRANE	GOCC_INTRINSIC_COMPONENT_OF_PRESYNAPTIC_MEMBRANE	0.000106926	KCNA1/CHRM2/GPM6A/CADM3/HTR2A/GRIK5/CDH2/CNTN1/ADAM23/CNR1/PTPRD/FXYD6/RGS7BP/P2RX1/ATP2B4/EFNB3/GABBR1/CHRNB2/PCDH17/P2RY1

GOBP_REGULATION_OF_MITOTIC_NUCLEAR_DIVISION	GOBP_REGULATION_OF_MITOTIC_NUCLEAR_DIVISION	0.000107044	IL1B/NUF2/TGFA/NEK2/FBXW5/MTBP/ZW10/SH2B1/TOM1L1/RAD21/TTK/KNTC1/AURKAIP1/XRCC3/FBXO5/ZWILCH/AURKB/PDXP/NDC80/BUB1/BUB3/KIF20B/AURKA/GEN1/CDC25C/PRMT5/SPC25/BORA/CENPF/INCENP/CHEK1/SPC24/BUB1B/DLGAP5/CDC20/CCNB1/ESPL1/HASPIN/MAD2L1/PLK1/BIRC5/CDCA8/RCC1/MKI67/HOXA13/ZWINT/PKMYT1/CDT1/CDCA2/SMPD3

GOBP_PATTERN_SPECIFICATION_PROCESS	GOBP_PATTERN_SPECIFICATION_PROCESS	0.000108271	HAND1/NKX2-2/RELN/CER1/SFRP1/HAND2/NEUROD1/CHRDL1/NKX2-5/FGF10/MEOX2/ASCL1/ALDH1A2/BARX1/DLL3/ASB2/AR/SIX2/SFRP2/NOG/GREM2/GPC3/NKX3-2/FGF2/ZBTB16/GREM1/DAAM2/HOXD10/CDON/WT1/GLI3/ZEB1/BICC1/CHRD/FOXF1/HOXA4/GLI1/TLL1/HHIP/SMARCD3/CRB2/NODAL/TBX18/MEIS1/NRP2/HEYL/PKD2/TDRD10/HOXA2/SHH/SMO/OSR1/PBX1/DKK1/PBX3/ISL1/MEOX1/WNT2B/FOXC2/DCHS1/PIFO/FGF1/HOXD4/CC2D2A/GPR161/TCF15/BMPR1B/SOX17/STC1/MEIS3P1/MEF2C/PTCH1/IRX3/WLS/TBX5/DYNC2H1/MEIS3/CXXC4/HOXA5/ROBO1/HOXD9/ARC/VANGL2/LEF1/ACVR1/PRKACB/GLI2/SEMA3C/NDRG4/NRP1/APLNR/MICAL2/BMP5/FGFR2/C3/HOXA3/SP8[illegible]

GOBP_REGULATION_OF_NEUROTRANSMITTER_LEVELS	GOBP_REGULATION_OF_NEUROTRANSMITTER_LEVELS	0.000112565	SYT4/BCHE/CHRM2/ATP1A2/TACR2/CHRNA3/PRIMA1/NRXN2/HTR2A/PAH/GRIK5/SLC18A2/CNR1/NLGN1/GPM6B/ADCY1/CACNB2/CHRNB4/SV2A/P2RX1/LIN7A/SLC1A7/PDE1B/PRKCB/PRKN/SNAP25/SYT11/MOXD1/LRRK2/SNCG/BRSK1/SLC29A4/RAB3B/COLQ/MEF2C/SLC18A1/APBA1/SYT1/ITGB3/SYP/P2RY1/GPER1/SYT5/CADPS/SNCAIP/BACE1/PFN2
GOBP_NEGATIVE_REGULATION_OF_RESPONSE_TO_WOUNDING	GOBP_NEGATIVE_REGULATION_OF_RESPONSE_TO_WOUNDING	0.000113809	APOH/VTN/FGA/RGMA/F2/FGG/ADAMTS18/FGF2/PHLDB2/SERPINF2/MMRN1/PRKG1/THBS1/SERPINE2/WFDC1/SERPINE1/PDGFRA/PROS1/KRT1/CLDN19/THBD/FAP/FGB/TAFA5/SERPING1/TFPI

GOBP_POSITIVE_REGULATION_OF_CALCIUM_ION_TRANSPORT	GOBP_POSITIVE_REGULATION_OF_CALCIUM_ION_TRANSPORT	0.000115987	PLA2G1B/GCG/PLP1/MYLK/ADCYAP1R1/CASQ1/JPH2/ANK2/AKAP6/F2/RGN/P2RX2/TRPC1/CXCL12/HSPA2/CACNB2/P2RX1/CAV1/STAC/PKD2/BDKRB1/CACNA2D1/CCL2
GOBP_REGULATION_OF_TRANSCRIPTION_ELONGATION_BY_RNA_POLYMERASE_II	GOBP_REGULATION_OF_TRANSCRIPTION_ELONGATION_BY_RNA_POLYMERASE_II	0.000117429	EAF2/INTS3/CDC73/ELL/BRD4/CDK13/ERCC6/NELFE/MED30/CTNNB1/RECQL5/MED8/RNF168/MED11/MED27/CDK12/SUPT16H/CCNT1/CCNK/TCERG1/VHL/LEO1/INTS12/MED14/MED25/WDR43/MED23/NCBP1/MED16/INTS13/EAF1/INTS7/AXIN1/INTS14/INTS9/HNRNPU/INTS8/MED15/INTS11/MED9/INTS1/INTS4/NELFCD/NELFB/GTF2F1/INTS5/MED26/MED1/SIRT6/MED18/NELFA/INTS2/EZH2/PWWP2B/INTS10/MED24/ELL3

GOBP_MEMBRANE_DEPOLARIZATION_DURING_ACTION_POTENTIAL	GOBP_MEMBRANE_DEPOLARIZATION_DURING_ACTION_POTENTIAL	0.000117809	ATP1A2/SCN7A/ANK2/CACNA1C/CACNA1H/SCN4B/CACNB2/KCNH2/SCN3A/SLC8A1/CACNA2D1
GOBP_RESPONSE_TO_FUNGUS	GOBP_RESPONSE_TO_FUNGUS	0.000119185	ELANE/MPO/DEFA4/CTSG/PLA2G5/DEFA6/PTX3/CHGA/S100A8/CX3CR1/C10orf99/S100A9
GOBP_REGULATION_OF_ANATOMICAL_STRUCTURE_SIZE	GOBP_REGULATION_OF_ANATOMICAL_STRUCTURE_SIZE	0.000119152	NPPB/ATP1A2/AGTR1/ACTN2/FGA/LMOD1/TACR1/KCNMA1/EPHA7/KCNMB1/NPR3/SCT/KCNA5/SLIT2/HTR2A/ACTA2/TMOD1/FGG/CFL2/CCL26/HTR2B/MAP1B/NGF/PAK3/SVIL/ADRA1D/PLXNA4/DAAM2/SMTNL2/CXCL12/GUCY1A1/ELN/SEMA3E/NTN1/ADD2/SERPINF2/AGT/CDC42EP3/PRKG1/CCL21/VSTM4/ADRA1B/MAPT/RND2/KANK4/AKT3/P2RX1/LPAR3/NKX6-1/CSF3/CAV1/EDNRA/FGF13/RGS2/LMOD3/NPR1/ITGA1/TENM1/L1CAM/SLC8A1/MMP2/COMP/AVPR1A/CYSLTR1/HBB/FHOD3/SOD3/FN1/SCTR/ADRB2/SPART/KIRREL1/OXTR/LRRK2/CRABP2/FOXC2/SEMA6A/CCL11/ARHGAP28/SEMA6D/NEFL/RAB3B/AVPR2/ISLR2/PLEKHH2/FGB/TMSB15A/NRCAM/SEMA6C/CYRIA/SEMA3G/MAP2/SEMA5B/SEMA3D/DSTN/[illegible]

GOBP_MITOCHONDRIAL_TRANSMEMBRANE_TRANSPORT	GOBP_MITOCHONDRIAL_TRANSMEMBRANE_TRANSPORT	0.000120494	ABCB7/TOMM40L/PAM16/PNPT1/TIMM17B/DNAJC15/MICU2/TIMM23/TIMM23B/MRS2/TIMM17A/SLC25A28/MAIP1/UCP2/SLC25A29/SLC25A23/TIMM50/TOMM70/MRPL18/SLC25A1/CHCHD4/CPT1A/PMPCB/GRPEL2/MCUB/MPC1/AFG3L2/MICU1/SLC25A3/SLC25A39/SLC25A38/SLC25A5/ABCB8/SLC25A37/SLC8B1/SLC25A33/SFXN4/SLC25A15/AIFM1/TIMM21/VDAC1/SLC25A40/GRPEL1/CPT2/HSPD1/STOML2/SFXN2/TOMM40/SFXN1/TIMM44/TST/CCDC51/SLC25A22/LETM1/MCU
GOBP_REGULATION_OF_CAMP_MEDIATED_SIGNALING	GOBP_REGULATION_OF_CAMP_MEDIATED_SIGNALING	0.00012141	ADCYAP1R1/SCT/GIP/PDE3A/RASD2/LPAR1/PDE2A/SCTR/FFAR4/GNAI1/PDE3B/APLNR
GOBP_REVERSE_CHOLESTEROL_TRANSPORT	GOBP_REVERSE_CHOLESTEROL_TRANSPORT	0.000121155	APOA2/APOA1/APOC3/CES1/APOA4/ABCA1/CLU

GOBP_REGULATION_OF_PLASMA_LIPOPROTEIN_PARTICLE_LEVELS	GOBP_REGULATION_OF_PLASMA_LIPOPROTEIN_PARTICLE_LEVELS	0.000121775	MPO/APOA2/APOA1/APOC3/AGTR1/ADIPOQ/APOB/PLA2G5/SOAT2/GPIHBP1/AGT/CD36/VLDLR/NR1H4/APOA4/MIR27B/ABCA1/LPL/MTTP
GOBP_REGULATION_OF_POTASSIUM_ION_TRANSMEMBRANE_TRANSPORTER_ACTIVITY	GOBP_REGULATION_OF_POTASSIUM_ION_TRANSMEMBRANE_TRANSPORTER_ACTIVITY	0.000124633	KCNA1/CASQ2/ACTN2/KCNK3/FHL1/ANK2/AKAP6/ABCC9/GRP/ATP1B2/KCNE4/CAV1/KCNG1
GOBP_COLLAGEN_METABOLIC_PROCESS	GOBP_COLLAGEN_METABOLIC_PROCESS	0.000125672	PRTN3/MIR145/F2/TNXB/DDR2/MFAP4/MMP8/SERPINF2/PRSS2/TGFB3/MMP3/ADAMTS3/MMP24/MMP2/LARP6/IL6/MMP7/MMP19/EMILIN1/MMP16/TNS2/CTSK/CCN2/VIM/MMP23B/MIR149/FAP/PCOLCE/COL1A2/INHBA/MRC2/TMPRSS6/COL1A1/PDGFRB/COL5A1/NPPC

GOMF_CORECEPTOR_ACTIVITY	GOMF_CORECEPTOR_ACTIVITY	0.000127051	NGFR/RGMA/ROR2/RECK/GPR15/CSPG4/GPC6/FCRL1/LILRA4/RGMB/IGSF1/RAMP2/TGFBR3/LY96/ITGB3/CXCR4/ROR1/ACKR3/NRP1/RAMP3/CD28/RAMP1/ENG
GOBP_CATECHOLAMINE_SECRETION	GOBP_CATECHOLAMINE_SECRETION	0.000127511	SYT4/CARTPT/VIP/KCNB1/HTR2A/CNR1/CXCL12/AGT/CHGA/PRKN/SYT11/OXTR/SNCG/GABBR1/SYT1/ADORA3/CHRNB2/P2RY1/SYT5/OXT
GOBP_REGULATION_OF_WNT_SIGNALING_PATHWAY_PLANAR_CELL_POLARITY_PATHWAY	GOBP_REGULATION_OF_WNT_SIGNALING_PATHWAY_PLANAR_CELL_POLARITY_PATHWAY	0.000127489	SFRP1/SFRP2/GPC3/RSPO3/DACT1/DAB2/ANKRD6/DKK1
GOBP_POSITIVE_REGULATION_OF_CALCIUM_ION_TRANSMEMBRANE_TRANSPORT	GOBP_POSITIVE_REGULATION_OF_CALCIUM_ION_TRANSMEMBRANE_TRANSPORT	0.000127797	PLA2G1B/PLP1/ADCYAP1R1/CASQ1/JPH2/ANK2/AKAP6/F2/RGN/P2RX2/TRPC1/HSPA2/CACNB2/P2RX1/CAV1/STAC/PKD2/BDKRB1/CACNA2D1

GOBP_REGULATION_OF_PEPTIDE_TRANSPORT	GOBP_REGULATION_OF_PEPTIDE_TRANSPORT	0.000128352	GCG/UCN3/CARTPT/CELA2A/GHRH/SFRP1/NEUROD1/NNAT/KCNB1/FGA/ADCYAP1/ADCY5/SCT/KCNA5/FGG/GRP/CNR1/GIP/CHGA/RFX6/NKX6-1/S100A8/RBP4/IL6/PRKN/NR1H4/SNAP25/ISL1/FFAR4/CCN3/GPR27/KLF7/FGB/NLGN2/BLK/GABBR1/GPLD1/MYRIP
GOBP_MUSCLE_ORGAN_MORPHOGENESIS	GOBP_MUSCLE_ORGAN_MORPHOGENESIS	0.000129032	HAND1/MYLK/NKX2-5/ANKRD1/MIR145/MYL3/NOG/ZFPM2/TPM1/ACTC1/EDNRA/TGFB2/COL3A1/EFEMP2/ISL1/FOXC2/S1PR1/TCF15/HEG1/RYR2/TGFBR3
GOBP_DEFENSE_RESPONSE_TO_FUNGUS	GOBP_DEFENSE_RESPONSE_TO_FUNGUS	0.000129637	ELANE/MPO/DEFA4/CTSG/PLA2G5/DEFA6/CHGA/S100A8/CX3CR1/C10orf99/S100A9

GOBP_SARCOPLASMIC_RETICULUM_CALCIUM_ION_TRANSPORT	GOBP_SARCOPLASMIC_RETICULUM_CALCIUM_ION_TRANSPORT	0.000131219	CASQ2/ATP1A2/PLN/CASQ1/ANK2/AKAP6/DMD/CACNA1C/HRC/SLC8A1/SLN/RYR2/CLIC2
GOBP_REGULATION_OF_CYTOKINESIS	GOBP_REGULATION_OF_CYTOKINESIS	0.000133636	RAB11FIP4/ZFYVE19/BRCA2/KIF20A/PKN2/PRC1/GIT1/AURKB/ENTR1/PDXP/KIF20B/CDC14B/KIF23/AURKA/KIF14/DRD2/GIPC1/PLK2/CIT/INCENP/CENPV/MYO19/RACGAP1/PLK4/CHMP4C/ECT2/PLK1/BIRC5/CDCA8/E2F8/E2F7/IGF1R/SSTR5
GOBP_ENDOCRINE_SYSTEM_DEVELOPMENT	GOBP_ENDOCRINE_SYSTEM_DEVELOPMENT	0.000137812	NKX2-2/GHRH/APOA1/NEUROD1/NKX2-5/FGF10/PCSK1/ASCL1/ALDH1A2/INSM1/NOG/CDH2/CYP1B1/FGF2/WT1/GIP/GLI1/RFX6/NKX6-1/EDNRA/ONECUT1/IL6/SHH/SMO/PDGFRA/PBX1/CGA/ISL1

GOBP_NUCLEOSIDE_MONOPHOSPHATE_METABOLIC_PROCESS	GOBP_NUCLEOSIDE_MONOPHOSPHATE_METABOLIC_PROCESS	0.000139766	RFK/DGUOK/NT5M/DHODH/ADSS2/DPYD/AMPD2/CMPK2/UMPS/DNPH1/MAGI3/GMPS/TJP2/GUK1/APRT/IMPDH2/ADK/UCK2/CASK/TK2/DCTD/NT5C/GART/NUDT2/PNP/ATIC/ADSS1/AK1/IMPDH1/AK2/PFAS/XDH/CAD/DTYMK/PRPS2/CDA/DCK/DUT/CMPK1/AK7/SHMT1/PPAT/TYMP/PAICS/ADA/TYMS/TK1/ENTPD8
GOBP_POSITIVE_REGULATION_OF_NEURON_DIFFERENTIATION	GOBP_POSITIVE_REGULATION_OF_NEURON_DIFFERENTIATION	0.00014008	NKX2-2/NEUROD1/NKX2-5/ASCL1/GDF6/MAP1B/NAP1L2/NGF/FGF2/PCP4/DAB1/CDON/CXCL12/ZEB1/BEND6/NKX6-1/FGFR1/FEZ1/HEYL/DKK1/GDF7/ATOH1/BRINP3/MEF2C/NRCAM/TCF4/IRX3/MMD

GOBP_MESONEPHROS_DEVELOPMENT	GOBP_MESONEPHROS_DEVELOPMENT	0.000141067	CER1/SFRP1/FGF10/SLIT2/TMEM59L/SIX2/NOG/ADAMTS16/GPC3/BMPER/FGF2/ZBTB16/GREM1/WT1/GLI3/GREB1L/AGT/PKD2/SHH/SMO/OSR1/PBX1/CRLF1/WNT2B/TCF21/FOXC2/DCHS1/FGF1/ILK
GOMF_RRNA_BINDING	GOMF_RRNA_BINDING	0.000141748	RPS11/RPS4X/RPL23A/MRPS7/FASTKD2/UTP25/MRPL20/BRIX1/RPS14/RPF2/RPS9/ERAL1/FASTKD5/RCC1L/RPS18/MRPL18/RPS3/RPS5/MRPS11/RPL12/KDM2B/IMP3/NSUN4/MRPS18A/RPL9/PTCD3/RPL3/TACO1/DDX28/DDX21/RPLP0/RPLP0P6/MRPL11/MRPL16/MRPS18C/RPUSD4/MRPS27/IMP4/RRS1/NPM1/RPL8/ERI1/TST/PPAN/MDM2

GOCC_POTASSIUM_CHANNEL_COMPLEX	GOCC_POTASSIUM_CHANNEL_COMPLEX	0.000147251	KCNA1/GRIK3/DPP6/KCNB1/KCNMA1/KCNMB1/KCNA5/ABCC9/GRIK5/KCNK2/KCNIP1/KCNJ8/KCNH2/KCNG1/KCNQ3/KCND1/KCNS1/KCND2
GOBP_DIGESTION	GOBP_DIGESTION	0.000147261	CTRB1/CLPS/CTRB2/PNLIP/APOA2/UCN3/CEL/PNLIPRP2/APOA1/NEUROD1/PRSS1/TACR1/FGF10/NPR3/SCT/PTGER3/SOAT2/PRSS2/CD36/SERPINA3/RBP4/APOA4/CLPSL2/CCKBR/OXTR/CYP39A1
GOBP_REGULATION_OF_VASCULAR_PERMEABILITY	GOBP_REGULATION_OF_VASCULAR_PERMEABILITY	0.000148846	AZU1/NPPB/TACR2/TACR1/SLIT2/FERMT2/PDE3A/AKAP12/PDE2A/NPR1/ANGPT1/SH3GL2/PTP4A3/RAMP2/CXCR2/CLDN5/TEK

GOCC_ACTOMYOSIN	GOCC_ACTOMYOSIN	0.000150531	SYNPO2/LDB3/PGM5/MYLK/MYL9/SORBS1/PDLIM3/ACTA2/FERMT2/ACTA1/TPM1/LPP/PALLD/PDLIM4/MYH10/FAM107A/SYNPO/PDLIM7/ILK/TEK/ACTN1
GOBP_NEGATIVE_REGULATION_OF_EPITHELIAL_CELL_PROLIFERATION	GOBP_NEGATIVE_REGULATION_OF_EPITHELIAL_CELL_PROLIFERATION	0.000150406	MIR483/SFRP1/APOH/NGFR/KRT4/RGN/AR/SFRP2/LIMS2/GPC3/ZEB1/THBS1/SERPINF1/CAV1/ATOH8/TGFB2/KLF9/MCC/MIR342/DAB2/CCL2/ISL1/CDKN1C/WNT10B/SLURP1/SNAI2/MIR149/SCG2/TGFBR3/MTSS1/MEF2C/MIR222/SPARC/PTPRM/PTCH1/MMRN2/ROBO1/SULF1
GOBP_PROTEIN_KINASE_C_SIGNALING	GOBP_PROTEIN_KINASE_C_SIGNALING	0.00015331	AZU1/SEZ6L/ANKRD1/SEZ6/HTR2B/DGKG/AKAP12/FIBIN

GOBP_MITOCHONDRIAL_RNA_METABOLIC_PROCESS	GOBP_MITOCHONDRIAL_RNA_METABOLIC_PROCESS	0.000154307	TEFM/CDK5RAP1/FASTKD3/TRMT10C/PRKAA1/TRMT5/PRORP/AARS2/PNPT1/TRNT1/DARS2/SLIRP/YARS2/FASTKD2/TRIT1/MTO1/TFB2M/FASTKD5/THAP11/TRMT61B/HSD17B10/WARS2/FASTK/GARS1/TFAM/FASTKD1/SARS2/PUS1/SLC25A33/SUPV3L1/PDE12/PPARGC1B/EARS2/LRPPRC/TFB1M/RPUSD4/TARS2/TBRG4/MRPL12/ELAC2/GRSF1/TWNK/POLRMT/CHCHD10

GOBP_POSITIVE_REGULATION_OF_CELL_CYCLE_PROCESS	GOBP_POSITIVE_REGULATION_OF_CELL_CYCLE_PROCESS	0.000156387	PPP1R35/VPS4B/INO80/GPSM2/HNRNPU/SLF1/PLCG2/IL1B/ANKRD17/TGFA/ZNF16/AKT1/EGFR/KLHL18/RANBP1/MTBP/NCAPH2/PAGR1/SH2B1/RAD21/SASS6/SMC4/DBF4/PKN2/RRM2B/MED1/DDX11/DDRGK1/MSX1/XRCC3/FBXO5/AURKB/NDC80/KIF20B/CDC14B/SPAG5/KIF23/AURKA/STOX1/RAD51AP1/KIF14/GEN1/EZH2/PHB2/TFDP1/CDC25C/DRD2/NPM1/GIPC1/SMC2/CDK1/CIT/MSX2/INCENP/RRM1/NCAPG2/CENPV/RACGAP1/DLGAP5/PLK4/PLRG1/RCC2/NCAPH/FEN1/CCNB1/ESPL1/TERT/ECT2/MAPK15/HYAL1/MAD2L1/DTL/CDC7/MDM2/CDCA5/BIRC5/CDCA8/CCND1/STIL/CDC25A/NCAPG/HOXA13/E2F8/RRM2/E2F7/CDT1/PBK/SMPD3/IGF1R/SSTR5
GOMF_3_5_EXONUCLEASE_ACTIVITY	GOMF_3_5_EXONUCLEASE_ACTIVITY	0.00016008	DIS3L2/CNOT8/USB1/PARN/RAD9A/CNOT6L/DIS3L/DIS3/PAN2/ISG20L2/APEX2/EXD3/CNOT7/EXOSC10/EXOSC2/EXOSC7/EXOSC5/PDE12/REXO4/WRN/POLG/CNOT6/EXOSC9/EXOSC4/CNOT1/TOE1/ERI2/POLE/ISG20/POLRMT/ERI1/POLD1

GOBP_RNA_METHYLATION	GOBP_RNA_METHYLATION	0.000159925	SNRPE/TRMT13/METTL5/WTAP/METTL14/METTL16/PCIF1/CBLL1/LCMT2/SNRPD2/TRMT61A/TRMT10C/THADA/TRMT2B/SNRPD3/TRMT5/NSUN6/DIMT1/NSUN3/METTL3/SNRPG/TRMT112/TARBP1/TRMT6/ALKBH8/TYW3/VIRMA/RAMAC/MTO1/BUD23/THUMPD3/THUMPD2/TFB2M/TRMO/TRMT61B/RBM15B/HSD17B10/FTSJ1/SNRPF/FTSJ3/MEPCE/METTL15/DALRD3/SNRPB/NSUN4/TRMT11/METTL2A/METTL2B/TRMT10A/ZCCHC4/FDXACB1/TRMT2A/NSUN2/NSUN5/FBL/MRM1/CMTR2/SNRPD1/NSUN5P1/WDR6/WDR4/TGS1/MRM3/TFB1M/RBM15/TRMT44/TRMT1/GTPBP3
GOBP_RESPONSE_TO_CATECHOLAMINE	GOBP_RESPONSE_TO_CATECHOLAMINE	0.000160098	PENK/CHRM2/NSG2/ADIPOQ/ADCY5/GNAO1/HTR2A/HTR2B/RGS4/FLNA/NR4A3/CALY/ATP2B4/CHRM4/GNAL/PRKN/PALM/LRRK2/PTGER1/RNLS/RYR2/GNA14/GNAZ

GOBP_REGULATION_OF_ANIMAL_ORGAN_MORPHOGENESIS	GOBP_REGULATION_OF_ANIMAL_ORGAN_MORPHOGENESIS	0.000160159	SFRP1/NGFR/FGF10/FGF7/AR/SIX2/SFRP2/PRICKLE2/NOG/GPC3/ROR2/RSPO3/FGF2/GREM1/WT1/AGT/PRICKLE1/RSPO2/HGF/DACT1/TNFRSF11B/SHH/SMO/DAB2/ANKRD6/DKK1/GPC6/FZD7/WNT2B/SNAI2/FGF1/CTHRC1/ROBO1/SULF1/CD34/ROR1/VANGL2
GOBP_DEVELOPMENT_OF_PRIMARY_FEMALE_SEXUAL_CHARACTERISTICS	GOBP_DEVELOPMENT_OF_PRIMARY_FEMALE_SEXUAL_CHARACTERISTICS	0.00016104	AFP/SFRP1/ADCYAP1R1/AMHR2/SLIT2/PGR/PTPRN/SLIT3/ZFPM2/WT1/PTX3/ADAMTS1/ZFP42/MMP2/ESR1/MMP19/PDGFRA/CGA/A2M/GDF9/RETN/BMPR1B/PCYT1B/INHBA/INHA

GOCC_PROTEIN_COMPLEX_INVOLVED_IN_CELL_ADHESION	GOCC_PROTEIN_COMPLEX_INVOLVED_IN_CELL_ADHESION	0.000162893	PLP1/VTN/ITGA7/TNC/TNN/JAM2/JAM3/NLGN1/LAMA2/MMRN1/ITGA8/ITGA9/ITGBL1/LAMA1/ITGA1/ITGA5/EMILIN1/TSPAN32/NID1/LAMC1/ITGB3/LGALS1/ITGA11
GOBP_KILLING_BY_HOST_OF_SYMBIONT_CELLS	GOBP_KILLING_BY_HOST_OF_SYMBIONT_CELLS	0.000165424	ELANE/AZU1/CTSG/F2/CXCL6/CAMP
GOMF_POTASSIUM_CHANNEL_ACTIVITY	GOMF_POTASSIUM_CHANNEL_ACTIVITY	0.000164849	KCNA1/GRIK3/KCNB1/KCNMA1/KCNK3/GRIK1/KCNMB1/KCNA5/ABCC9/GRIK5/KCNK2/KCNH6/KCNT2/KCNIP1/KCNJ8/KCNE4/KCNH2/PKD2/KCNG1/SNAP25/KCNQ3/KCND1/KCNS1/KCND2

GOBP_REGULATION_OF_NUCLEAR_DIVISION	GOBP_REGULATION_OF_NUCLEAR_DIVISION	0.000165268	CALR/IL1B/NUF2/TGFA/NEK2/FBXW5/MTBP/ZW10/SH2B1/TOM1L1/RAD21/TTK/KNTC1/AURKAIP1/MSX1/XRCC3/FBXO5/FZR1/ZWILCH/AURKB/PDXP/NDC80/BUB1/BUB3/KIF20B/AURKA/RAD51AP1/GEN1/CDC25C/PRMT5/SPC25/BORA/MSX2/CENPF/INCENP/CHEK1/SPC24/BUB1B/DLGAP5/CDC20/CCNB1/ESPL1/HASPIN/MAD2L1/PLK1/BIRC5/CDCA8/RCC1/MKI67/HOXA13/ZWINT/PKMYT1/CDT1/CDCA2/SMPD3

GOBP_SPROUTING_ANGIOGENESIS	GOBP_SPROUTING_ANGIOGENESIS	0.000169788	AGTR1/MIR483/NGFR/MEOX2/SLIT2/RSPO3/BMPER/FGF2/TNN/GREM1/SEMA3E/VSTM4/RECK/AKT3/THBS1/ANGPT1/RHOJ/ITGA5/MIR342/CCBE1/MIR27B/PARVA/FOXC2/SEMA6A/FGF1/ADGRA2/MIR149/SRPX2/RAMP2/TEK/EGR3/MIR222/MIR10B/ADAMTS9/GPLD1/MMRN2/VEGFC/ROBO1/MIR196A1/LEF1/STARD13/CLEC14A/NRP1/APLNR
GOBP_POSITIVE_REGULATION_OF_AMINE_TRANSPORT	GOBP_POSITIVE_REGULATION_OF_AMINE_TRANSPORT	0.00017142	SYT4/CARTPT/VIP/TACR2/KCNB1/SLC38A3/CXCL12/AGT/AVPR1A/OXTR/RAB3B/GABBR1/SYT1/CHRNB2/OXT/CLTRN
GOCC_AZUROPHIL_GRANULE	GOCC_AZUROPHIL_GRANULE	0.000171309	ELANE/MPO/PRTN3/AZU1/DEFA4/RNASE3/BPI/CTSG/RNASE2/TTR/PRSS2/SERPINA3/ORM2/RETN/GLIPR1/STOM

GOBP_REGULATION_OF_SYSTEMIC_ARTERIAL_BLOOD_PRESSURE_MEDIATED_BY_A_CHEMICAL_SIGNAL	GOBP_REGULATION_OF_SYSTEMIC_ARTERIAL_BLOOD_PRESSURE_MEDIATED_BY_A_CHEMICAL_SIGNAL	0.000172105	AGTR1/TACR1/CTSG/CMA1/ADRA1D/SERPINF2/AGT/TPM1/ADRA1B/CPA3/CORIN/AVPR1A/ADRB2/OXTR/AVPR2/ENPEP
GOBP_PEPTIDE_TRANSPORT	GOBP_PEPTIDE_TRANSPORT	0.000177707	GCG/UCN3/CARTPT/CELA2A/GHRH/VIP/TACR2/SFRP1/NEUROD1/NNAT/KCNB1/FGA/ADCYAP1/ADCY5/CPE/SCT/KCNA5/PTPRN/FGG/GRP/CNR1/GIP/CHGA/RFX6/NKX6-1/S100A8/RBP4/IL6/PRKN/NR1H4/SNAP25/ISL1/FFAR4/CCN3/GJA1/GPR27/ABCA1/KLF7/FGB/NLGN2/BLK/GABBR1/SLC16A2/GPLD1/MYRIP/SLC16A10

GOBP_REGULATION_OF_CELL_SUBSTRATE_ADHESION	GOBP_REGULATION_OF_CELL_SUBSTRATE_ADHESION	0.000178437	APOA1/SFRP1/MYOC/VTN/FGA/APOD/CCDC80/FGG/ABI3BP/FERMT2/LIMS2/EPHA3/FBLN1/NPY/FLNA/PHLDB2/GREM1/VIT/COL26A1/GPM6B/SEMA3E/COL8A1/CD36/CCL21/FOXF1/THBS1/ECM2/CCN1/ITGA5/ONECUT1/SERPINE1/FAM107A/SPOCK1/EMILIN1/DAB2/FN1/EFEMP2/FZD7/DLC1/FBLN2/COL16A1/ILK/HAS2/BST1/PDPN/FGB/NID1/TEK/THY1/CORO2B/ITGB3/CCR7/MYADM/COL1A1/NDNF/NRP1/FZD4/RRAS/EDIL3/EGFLAM

GOBP_LEUKOCYTE_MIGRATION	GOBP_LEUKOCYTE_MIGRATION	0.000178874	PLA2G1B/ELANE/PRTN3/AZU1/CCL16/TAFA4/TACR1/THBS4/CTSG/APOD/SLIT2/ASB2/CCL26/ROR2/ITGA7/PTN/GREM1/JAM2/JAM3/CXCL6/LYVE1/CXCL12/F7/IL33/ADD2/CHGA/CCL21/ITGA9/CH25H/THBS1/GP2/CCL19/S100A8/GPR15/IL1R1/CX3CR1/CNR2/SELP/C10orf99/CYP7B1/TGFB2/ITGA1/MMP2/BDKRB1/IL6/S100A9/SERPINE1/EMILIN1/CCL2/FOLR2/GPR183/CCN3/CD200/PDGFD/CCL11/S1PR1/DUSP1/SELL/BST1/CXCR2/CD300H/RARRES2/CAMK1D/SCG2/THY1/CXCR1/S100A12/RIPOR2/ITGB3/SELE/VEGFC/CD99L2/MPP1/CXCR4/CCR7/CD34/IL34/KIT/FFAR2/PECAM1/EDNRB/TNFSF18/EDN2/FPR2/BMP5/XG

GOCC_NEURON_TO_NEURON_SYNAPSE	GOCC_NEURON_TO_NEURON_SYNAPSE	0.000179777	PENK/CHRM2/CHRNA3/BAALC/NGFR/PSD/EPHA7/GRIN2A/CRYAB/SLC8A2/GAP43/GRIK5/IGSF11/CDH2/CAP2/ZDHHC15/MAP1B/PLPPR4/PAK3/ATP1B2/CACNA1C/MPDZ/CACNG4/NLGN4X/NLGN1/PTPRD/ADCY1/ADD2/ITGA8/NLGN4Y/DLG2/RGS7BP/NCS1/LIN7A/SORCS2/CLSTN2/SLC8A1/PRRT1/PRKN/SPOCK1/INSYN1/PALM/SYT11/BSN/EFNB3/MPP2/NLGN3/KCND1/LRRC4/SYNPO/CTNND2/TMEM108/DLG4/NEURL1/PRNP/LRRC4C/KCND2/ITPR1/SYNDIG1/IQSEC3/NRCAM/NLGN2/PTCH1/SYT1/DCLK1/ADGRB1/P2RY1/GPER1/ARC
GOCC_LYSOSOMAL_LUMEN	GOCC_LYSOSOMAL_LUMEN	0.000180418	NSG2/OGN/KERA/OMD/PRELP/APOB/GPC3/DCN/CUBN/CTSV/CSF3/LUM/CSPG4/CTSF/GPC6/SDC2/CTSK/VCAN/FMOD/BGN/EPDR1/CD1E/PDGFRB

GOBP_NON_CANONICAL_WNT_SIGNALING_PATHWAY	GOBP_NON_CANONICAL_WNT_SIGNALING_PATHWAY	0.000183489	SFRP1/MYOC/SFRP2/PRICKLE2/GPC3/ROR2/RSPO3/FRZB/SFRP4/DAAM2/PRICKLE1/FZD10/DACT1/DAB2/ANKRD6/DKK1/GPC6/FZD7
GOBP_REGULATION_OF_GLUCOSE_TRANSMEMBRANE_TRANSPORT	GOBP_REGULATION_OF_GLUCOSE_TRANSMEMBRANE_TRANSPORT	0.000183838	PLA2G1B/ADIPOQ/SORBS1/IGF1/GPC3/CLIP3/NR4A3/GIP/PID1/EDNRA/ITLN1/KLF15/PRKCB/FFAR4
GOBP_RESPONSE_TO_BMP	GOBP_RESPONSE_TO_BMP	0.000184114	CER1/COL2A1/SFRP1/DSG4/CHRDL1/RBPMS2/RGMA/TMEM100/CHRDL2/SFRP2/GDF6/NOG/GREM2/GPC3/BMPER/GREM1/SFRP4/CHRD/TGFB3/LEFTY2/ZNF423/FSTL1/CRB2/NODAL/CCN1/TGFB2/FBN1/HEYL/SMAD9/COMP/DKK1/SPART/GDF9/GDF7/RGMB/ILK/BMPR1B/TGFBR3/SCUBE3/SULF1/ELAPOR2/TMPRSS6/LEF1/ACVR1

GOBP_EXTRACELLULAR_MATRIX_ASSEMBLY	GOBP_EXTRACELLULAR_MATRIX_ASSEMBLY	0.000185017	MYH11/HAS1/TNXB/MFAP4/PHLDB2/GPM6B/FBLN5/FKBP10/AGT/COL3A1/EMILIN1/EFEMP2/ANTXR1/LOX/HAS2/RAMP2/PHLDB1/LTBP3/COL1A2/THSD4/TIE1/NTNG2/PXDN/NTN4
GOBP_POSITIVE_REGULATION_OF_NERVOUS_SYSTEM_DEVELOPMENT	GOBP_POSITIVE_REGULATION_OF_NERVOUS_SYSTEM_DEVELOPMENT	0.000184676	NKX2-2/SOX10/RELN/ASCL1/SLIT2/FLRT2/CHODL/ADGRB3/MAP1B/MAP6/NGF/PAK3/PTN/FGF2/PLXNA4/DIXDC1/NLGN1/PTPRD/CXCL12/GLI3/IL33/NTN1/MAPT/RND2/LPAR3/SERPINF1/NKX6-1/CX3CR1/SMARCD3/ASPA/SERPINE2/L1CAM/LRRN1/CLSTN2/IL6/SHH/SMO/FN1/SLITRK6/OXTR/NLGN3/CRABP2/ID4/NEURL1/NEFL/TENM4/SRPX2/ISLR2/PTPRZ1/SYNDIG1/MIR222/NLGN2/THBS2/VEGFC/ADGRB1/ROBO1/CXCR4/GPER1/IL6ST/IL34/KIT/AMIGO2/OXT/NRP1/LRRC4B

GOMF_EXORIBONUCLEASE_ACTIVITY	GOMF_EXORIBONUCLEASE_ACTIVITY	0.000195489	DIS3L2/CNOT8/EXOSC8/USB1/PARN/CNOT6L/CPSF3/DIS3L/DIS3/PAN2/ISG20L2/XRN1/CNOT7/EXOSC10/EXOSC2/EXOSC7/EXOSC5/PDE12/CNOT6/EXOSC9/EXOSC4/CNOT1/TOE1/DCPS/ERI2/ISG20/POLRMT/ZC3H12A/ERI1
GOMF_ACTININ_BINDING	GOMF_ACTININ_BINDING	0.000198064	SYNPO2/LDB3/KCNA5/PDLIM3/CACNA1C/CSRP1/PALLD/PDLIM4/PKD2/CSRP2/PDLIM7
GOBP_NEGATIVE_REGULATION_OF_COAGULATION	GOBP_NEGATIVE_REGULATION_OF_COAGULATION	0.000198482	APOH/VTN/FGA/F2/FGG/ADAMTS18/SERPINF2/PRKG1/THBS1/SERPINE2/SERPINE1/PDGFRA/PROS1/KRT1/THBD/FAP/FGB/SERPING1/TFPI

GOBP_PHOSPHOLIPASE_C_ACTIVATING_G_PROTEIN_COUPLED_RECEPTOR_SIGNALING_PATHWAY	GOBP_PHOSPHOLIPASE_C_ACTIVATING_G_PROTEIN_COUPLED_RECEPTOR_SIGNALING_PATHWAY	0.000198073	VIP/CHRM2/AGTR1/ACTN2/TACR1/GRPR/NPR3/F2/HTR2A/HTR2B/GRP/CCKAR/ADRA1D/PTGER3/PTH1R/AGT/LPAR4/CHGA/GPR17/ADRA1B/CX3CR1/LPAR1/NMUR1/ESR1/CCKBR/FFAR4/GPR27/S1PR1/CXCR2/FPR1/GNA14
GOBP_ACUTE_INFLAMMATORY_RESPONSE	GOBP_ACUTE_INFLAMMATORY_RESPONSE	0.000197405	ELANE/REG3G/APOA2/AHSG/HP/TACR1/F2/NPY/PTGER3/CNR1/SERPINF2/S100A8/CREB3L3/SERPINA3/IL6/ORM2/FN1/F8/A2M/CXCR2/KL/FCGR2B/IL20RB/CCR7/IL6ST/ACVR1/FFAR2/HAMP/EDNRB

GOBP_REGULATION_OF_OSSIFICATION	GOBP_REGULATION_OF_OSSIFICATION	0.000199017	AHSG/SFRP1/TPH1/TACR1/OMD/MGP/SIX2/DDR2/PTN/ZBTB16/GREM1/GPM6B/GDF10/TGFB3/CCN1/TGFB2/SLC8A1/TMEM119/COMP/OSR1/PBX1/DKK1/ADRB2/NELL1/WNT10B/S1PR1/BMPR1B/KL/MEF2C/LTBP3/TWIST1/ACVR1/OXT/SRGN
GOBP_NEUROTRANSMITTER_TRANSPORT	GOBP_NEUROTRANSMITTER_TRANSPORT	0.000200112	SYT4/CHRM2/SLC18A3/ATP1A2/TACR2/CHRNA3/NRXN2/HTR2A/GRIK5/SLC18A2/CNR1/SV2B/NLGN1/GPM6B/ADCY1/CACNB2/SLC17A7/CHRNB4/SV2A/P2RX1/LIN7A/SLC1A7/PRKCB/PRKN/SNAP25/SYT11/LRRK2/SNCG/BRSK1/SLC29A4/SLC6A16/RAB3B/MEF2C/SLC18A1/APBA1/SYT1/ITGB3/SYP/P2RY1/GPER1/SYT5/CADPS/SNCAIP/BACE1/PFN2

GOCC_INTRINSIC_COMPONENT_OF_POSTSYNAPTIC_SPECIALIZATION_MEMBRANE	GOCC_INTRINSIC_COMPONENT_OF_POSTSYNAPTIC_SPECIALIZATION_MEMBRANE	0.00020122	EPHA7/GRIN2A/GRIK5/CDH2/PLPPR4/CACNG4/NLGN4X/NLGN1/ADCY1/NLGN4Y/RGS7BP/CLSTN2/EFNB3/NLGN3/LRRC4/DLG4/LRRC4C/KCND2/NRCAM/NLGN2/CHRNB2/LRRC4B/GRID1
GOBP_POSITIVE_REGULATION_OF_CHROMOSOME_SEGREGATION	GOBP_POSITIVE_REGULATION_OF_CHROMOSOME_SEGREGATION	0.00020249	HNRNPU/NCAPH2/RAD18/SMC4/AURKB/SMC2/INCENP/NCAPG2/RCC2/NCAPH/CCNB1/BIRC5/CDCA8/NCAPG/CDT1

GOBP_CENTROSOME_DUPLICATION	GOBP_CENTROSOME_DUPLICATION	0.000203197	SIRT1/CDK5RAP2/CCP110/TRIM37/C2CD3/CEP135/CDK2/USP33/CCDC57/XPO1/ALMS1/CHMP2B/NUP62/CHORDC1/PDCD6IP/CEP295/BRCA1/CHMP1A/CEP192/PPP1R35/VPS4B/NAT10/NDE1/NUBP1/CHMP5/FBXW5/CEP152/RTTN/CKAP5/BRCA2/RBM14/SASS6/KAT2A/CEP72/POC1B/XRCC3/NDC80/GEN1/CNTROB/NPM1/CEP131/WDR62/PLK2/SAC3D1/PLK4/CHMP4C/CCNF/STIL

GOCC_NEURON_SPINE	GOCC_NEURON_SPINE	0.000205556	ATP1A2/ACTN2/NGFR/PSD/GPM6A/GRIN2A/CRYAB/SEZ6/SLC8A2/MAP1B/IGF2BP1/NLGN1/ITGA8/MAPT/RGS7BP/PRKAR2B/LPAR1/SORCS2/PDLIM4/HPCA/SLC8A1/PALMD/CTTNBP2/PALM/SYT11/MPP2/KCND1/LRRC4/SYNPO/DLG4/NEURL1/KCND2/SYNDIG1/ACTN1/APBA1/GABBR1/FCGR2B/ADGRB1/GPER1/ARC
GOBP_POSITIVE_REGULATION_OF_CHROMOSOME_SEPARATION	GOBP_POSITIVE_REGULATION_OF_CHROMOSOME_SEPARATION	0.000206001	NCAPH2/SMC4/AURKB/SMC2/INCENP/NCAPG2/DLGAP5/NCAPH/ESPL1/MAPK15/BIRC5/CDCA8/NCAPG/CDT1

GOBP_TISSUE_MIGRATION	GOBP_TISSUE_MIGRATION	0.000207258	APOA1/ACTG2/MIR483/APOH/TACR1/FGF10/MEOX2/SLIT2/ACTA2/FGF7/BMPER/CYP1B1/PTN/SMOC2/FGF2/GREM1/DAAM2/DCN/ACTA1/PRKD1/AGT/ACTC1/FOXF1/VSTM4/AKT3/ATP2B4/THBS1/SERPINF1/KANK2/LGALS12/ATOH8/FSTL1/FGFR1/ANGPT1/TGFB2/RHOJ/NRP2/MCC/MIR342/CCBE1/MIR27B/CCN3/ZEB2/SNAI2/FOXC2/FGF1/ADGRA2/MIR149/PTP4A3/HAS2/FAP/SRPX2/RHOB/HSPB1/STC1/SCG2/TEK/MEF2C/EGR3/MIR222/TNFSF12/SPARC/MIR10B/ADAMTS9/PTPRM/NANOS1/GPLD1/ITGB3/MMRN2/VEGFC/ADGRB1/ROBO1/MIR196A1/KIT/STARD13/CLEC14A/PLPP3/NRP1/PFN2/RRAS/EDN2

GOBP_POSITIVE_REGULATION_OF_LIPID_METABOLIC_PROCESS	GOBP_POSITIVE_REGULATION_OF_LIPID_METABOLIC_PROCESS	0.0002091	APOA2/APOA1/ADIPOQ/SORBS1/CES1/F2/SCT/HTR2A/RGN/HTR2B/FGF2/NR4A3/TNFAIP8L3/PRKD1/CCL21/CCL19/CCN1/FABP3/ZBTB20/NR1H4/APOA4/AVPR1A/PDGFRA/DAB2/CGA/FGF1/TEK/TWIST1/GPLD1/FLT3/ENPP7/ELOVL5/CCR7/KIT/CCDC3/PDGFRB
GOBP_ORGAN_OR_TISSUE_SPECIFIC_IMMUNE_RESPONSE	GOBP_ORGAN_OR_TISSUE_SPECIFIC_IMMUNE_RESPONSE	0.000209562	PLA2G1B/DEFA4/RNASE3/RNASE2/DEFA6/CAMP/GP2/IL6/APOA4

GOBP_KIDNEY_EPITHELIUM_DEVELOPMENT	GOBP_KIDNEY_EPITHELIUM_DEVELOPMENT	0.000211091	CER1/SFRP1/ADIPOQ/EPHA7/SLIT2/TMEM59L/SIX2/NOG/ADAMTS16/GPC3/BMPER/FGF2/GREM1/WT1/GLI3/GREB1L/AGT/KANK2/EDNRA/WWTR1/HEYL/PKD2/KLF15/SHH/SMO/OSR1/PBX1/CRLF1/WNT2B/TCF21/FOXC2/DCHS1/FGF1/ILK/CXCR2/MTSS1/MEF2C/PTCH1/IRX3
GOMF_NEUROPEPTIDE_HORMONE_ACTIVITY	GOMF_NEUROPEPTIDE_HORMONE_ACTIVITY	0.000212578	SPX/CARTPT/GHRH/VIP/PENK/ADCYAP1/GRP/NPY

GOBP_POSITIVE_REGULATION_OF_DOUBLE_STRAND_BREAK_REPAIR	GOBP_POSITIVE_REGULATION_OF_DOUBLE_STRAND_BREAK_REPAIR	0.000212285	ING3/ARID2/ARID1A/SLF2/ACTR2/ERCC6/MGMT/YEATS4/TRRAP/UBE2N/SHLD2/SHLD3/WAS/MAD2L2/SMARCD1/EP400/PNKP/RIF1/BRD7/BRD8/MRGBP/SMARCC1/PIAS4/ACTL6A/PARP9/KAT5/SPIDR/SMARCC2/SMCHD1/HDGFL2/MMS19/SMARCB1/FUS/RUVBL1/DTX3L/SLF1/RUVBL2/FANCB/PARP1/SPIRE2/BCL7A/PRKDC/DDX11/KMT5C/SMARCD2/SIRT6/RBBP8/SMARCE1/WRAP53/RAD51AP1/SMARCA4/FOXM1/TIMELESS/PARP3
GOBP_ADENYLATE_CYCLASE_INHIBITING_G_PROTEIN_COUPLED_RECEPTOR_SIGNALING_PATHWAY	GOBP_ADENYLATE_CYCLASE_INHIBITING_G_PROTEIN_COUPLED_RECEPTOR_SIGNALING_PATHWAY	0.000214649	CHRM2/GRIK3/ADCY5/NPR3/FLNA/GABBR2/AKAP12/EDNRA/CHRM4/RGS2/LPAR1/PDE2A/S1PR3/PALM/SSTR2/S1PR1/GNAI1/GABBR1/ITGB3/GNAZ/P2RY1/FPR2/P2RY12/CHRM3/GPR37/OXER1/GPR176

GOBP_REGULATION_OF_ACTIN_FILAMENT_ORGANIZATION	GOBP_REGULATION_OF_ACTIN_FILAMENT_ORGANIZATION	0.000215214	APOA1/SYNPO2/SFRP1/ACTN2/MYOC/LMOD1/TACR1/SLIT2/TMOD1/CFL2/CCL26/FERMT2/RGS4/FLNA/PAK3/SVIL/PHLDB2/DAAM2/CXCL12/ELN/ADD2/SERPINF2/WASF3/TPM1/CDC42EP3/CCL21/TGFB3/PPM1E/KANK4/CSF3/CGNL1/LPAR1/LMOD3/TENM1/PRKN/FHOD3/DLC1/KIRREL1/CCN2/SYNPO/CCL11/ARHGAP28/MIR149/S1PR1/PLEKHH2/TMSB15A/MTSS1/WASF1/CORO2B/CYRIA/MAGEL2/DSTN/CCR7/MYADM/ARHGEF15/GSN/NRP1/RDX/ARHGAP40/PFN2

GOBP_POSITIVE_REGULATION_OF_CELL_JUNCTION_ASSEMBLY	GOBP_POSITIVE_REGULATION_OF_CELL_JUNCTION_ASSEMBLY	0.000220578	SFRP1/MYOC/FLRT2/FERMT2/ADGRB3/NLGN1/PTPRD/AGT/CAV1/LRRN1/CLSTN2/HOPX/SLITRK6/OXTR/NLGN3/COL16A1/CLDN19/SRPX2/SYNDIG1/CLDN5/TEK/THY1/NLGN2/IRX3/THBS2/TBX5/ADGRB1/AMIGO2/OXT/NRP1/LRRC4B/AMIGO1/NPHP1/GHRL/CLSTN3/NTRK2

GOBP_CELL_MATRIX_ADHESION	GOBP_CELL_MATRIX_ADHESION	0.000220422	SFRP1/ACTN2/MYOC/VTN/FGA/SORBS1/APOD/FGG/STON1/FERMT2/TNXB/EPHA3/ITGA7/PHLDB2/TNN/GREM1/JAM3/LYVE1/GPM6B/FREM1/SEMA3E/FBLN5/CD36/ITGA8/CCL21/ITGA9/ITGBL1/THBS1/ECM2/ITGA1/L1CAM/ITGA5/ONECUT1/COL3A1/SERPINE1/FAM107A/EMILIN1/FN1/EFEMP2/SGCE/DLC1/COL16A1/CCN2/BCAM/ILK/BST1/FGB/NID1/TEK/ACTN1/THY1/ADAMTS9/EDA/CORO2B/ITGB3/SNED1/EPDR1/CCR7/CD34/ITGA11
GOBP_SARCOMERE_ORGANIZATION	GOBP_SARCOMERE_ORGANIZATION	0.000222082	LDB3/ACTN2/FLNC/CASQ1/ANKRD1/KLHL41/CFL2/CSRP1/TPM1/FHOD3/CSRP2

GOBP_REGULATION_OF_ENDOCYTOSIS	GOBP_REGULATION_OF_ENDOCYTOSIS	0.000222943	SYT4/APOC3/ADIPOQ/VTN/RSPO1/STON1/GPC3/EPHA3/CLIP3/TF/GREM1/SFRP4/NLGN1/CALY/PRKD1/SLC17A7/CD36/CCL21/CCL19/CAV1/ANGPT1/HPCA/SERPINE1/DAB2/RAB31/DKK1/SGIP1/SYT11/MIR27B/NTF3/LRRK2/DLG4/USH1G/CLU/ITGB3/SELE

GOCC_MEMBRANE_MICRODOMAIN	GOCC_MEMBRANE_MICRODOMAIN	0.000226953	PRTN3/ATP1A2/CHRNA3/BAALC/ADCYAP1R1/KCNMA1/SLC2A4/SGCA/PTGIS/ANK2/SORBS1/BVES/AKAP6/KCNA5/HTR2A/DMD/CDH2/ADCY2/CLIP3/CNTN1/TRPC4/CNR1/GPM6B/ADCY1/ADD2/GASK1A/CD36/ADRA1B/MAPT/P2RX1/ATP2B4/GP2/CAVIN1/CAV1/PRKAR2B/CAVIN2/ANGPT1/MAL/ITGA1/ITLN1/SHH/SMO/ARID3A/DLC1/LRRK2/RGMB/GJA1/STOM/S1PR1/EHD2/HAS2/PRNP/ABCA1/KCND2/PDPN/TEK/THY1/TFPI/GABBR1/PTCH1/SELE/CHRNB2/MS4A4A/SULF1/RHOQ/ARC/MYADM/IL6ST/PLPP3/PECAM1/EDNRB/CR1/BACE1/PLPP1/CAVIN4/ABCG2/OLR1

GOCC_OXIDOREDUCTASE_COMPLEX	GOCC_OXIDOREDUCTASE_COMPLEX	0.000227951	BCKDHB/NDUFB8/NDUFC2/ETFB/NCF4/OGDH/NDUFB5/IDH3G/NDUFC1/GPD1L/NCF1C/NDUFA1/RAC1/NDUFB4/UQCRB/NDUFS8/PDHX/UQCR11/NDUFB2/ETFA/NDUFA8/NDUFA6/RAC2/NDUFB10/DLD/NDUFS5/NDUFB3/NDUFA12/NDUFA5/UQCRQ/MRPS36/GPD1/NDUFA9/NDUFB6/NDUFA11/NDUFAF1/NDUFS6/UQCRFS1/NDUFA10/NCF1/NDUFS2/NDUFA2/NDUFS1/NDUFS3/SDHD/GPD2/DLST/NDUFB7/IDH3B/BCS1L/CBR4/NDUFAB1/UQCR10/PDHA1/DUOX2/NDUFA13/LDHA/SDHB/CYB5B/PDK1/P4HA1/UQCRC2/UQCRFS1P1/UQCRH/SDHA/IDH3A/P4HB/NDUFV1/CYBA/FOXRED1/DUOX1/NOXA1/DLAT/RRM1/NDUFS7/UQCRC1/UQCRHL/CYC1/RRM2

GOBP_MICROTUBULE_ORGANIZING_CENTER_ORGANIZATION	GOBP_MICROTUBULE_ORGANIZING_CENTER_ORGANIZATION	0.000228148	SIRT1/CDK5RAP2/CCP110/CROCC/TRIM37/CETN3/C2CD3/TUBE1/CTNNB1/CCDC61/HAUS1/CEP135/NSFL1C/CDK2/HAUS3/PCM1/UBXN2B/USP33/CCDC57/GOLGA2/XPO1/XRCC2/ALMS1/CHMP2B/NUP62/ODF2/CHORDC1/HAUS2/PDCD6IP/CEP295/HAUS4/BRCA1/CHMP1A/CEP192/PPP1R35/VPS4B/NAT10/NDE1/NUBP1/CHMP5/NEK2/FBXW5/CEP152/E2F4/RTTN/CKAP5/RANBP1/BRCA2/RBM14/HAUS6/SASS6/KAT2A/HAUS5/SSX2IP/CEP72/POC1B/MCPH1/SGO1/HAUS8/XRCC3/NDC80/AURKA/PARD6A/FOXJ1/PATJ/GEN1/CNTROB/NPM1/PCLAF/CEP131/CDK1/WDR62/PLK2/CHEK1/SLC16A1/SAC3D1/PLK4/CHMP4C/CEP85/MCIDAS/PLK1/KIF11/CCNF/STIL/ATF5/CCDC78
GOCC_T_TUBULE	GOCC_T_TUBULE	0.000231105	ATP1A2/CASQ1/SLC2A4/ANK2/AKAP6/CACNA1C/CACNG4/CACNB2/TGFB3/ATP2B4/STAC/AHNAK2/SLC8A1/CACNA2D1

GOBP_STEROL_HOMEOSTASIS	GOBP_STEROL_HOMEOSTASIS	0.000235878	APOA2/APOA1/APOC3/FABP4/CES1/APOB/SOAT2/GPIHBP1/PLA2G12B/CAV1/CYP7B1/FABP3/NR1H4/APOA4/MIR27B/MALRD1/CYP39A1/ABCA1/LPL/MTTP
GOCC_MHC_CLASS_II_PROTEIN_COMPLEX	GOCC_MHC_CLASS_II_PROTEIN_COMPLEX	0.000240678	HLA-DPA1/HLA-DMB/HLA-DOB/HLA-DRA/HLA-DQA2/HLA-DQA1/HLA-DRB5/CD74/HLA-DRB1/HLA-DOA/HLA-DQB1/HLA-DQB2/HLA-DMA
GOBP_FEMALE_SEX_DIFFERENTIATION	GOBP_FEMALE_SEX_DIFFERENTIATION	0.000240783	AFP/SFRP1/ADCYAP1R1/FGF10/AMHR2/SLIT2/PGR/PTPRN/SLIT3/ZFPM2/WT1/PTX3/ADAMTS1/ZFP42/RBP4/MMP2/ESR1/MMP19/PDGFRA/CGA/A2M/GDF9/RETN/BMPR1B/PCYT1B/INHBA/INHA

GOBP_DIGESTIVE_SYSTEM_DEVELOPMENT	GOBP_DIGESTIVE_SYSTEM_DEVELOPMENT	0.000240893	NKX2-2/SOX10/SFRP1/MYOCD/RBPMS2/FGF10/ASCL1/ALDH1A2/SCT/BARX1/SIX2/SFRP2/CLMP/NPY/NKX3-2/IGF2/GLI3/GIP/FOXF1/TGFB3/GLI1/NODAL/DACT1/TGFB2/COL3A1/SHH/SMO/PDGFRA/FOXF2/CCKBR/TCF21/OXTR/DCHS1/SOX17/RARRES2/PDGFC/WLS/CYP1A1/HOXA5/IL6ST/KIT/GLI2/EDNRB
GOBP_RENAL_TUBULAR_SECRETION	GOBP_RENAL_TUBULAR_SECRETION	0.000245967	SPX/NPPB/AGTR1/TACR1/AGT/NPR1/CORIN/AVPR1A/AVPR2/STC1/OXT/EDNRB/ABCG2

GOBP_REGULATION_OF_EXTRACELLULAR_MATRIX_ORGANIZATION	GOBP_REGULATION_OF_EXTRACELLULAR_MATRIX_ORGANIZATION	0.00024657	ANGPTL7/TNXB/DDR2/PHLDB2/LAMA2/AGT/LAMA1/PRDM5/IL6/EMILIN1/EFEMP2/ANTXR1/TCF15/HAS2/FAP/AEBP1/PDPN/NID1/PHLDB1/LAMC1/ITGB3/LRP1/TIE1
GOBP_SENSORY_PERCEPTION_OF_PAIN	GOBP_SENSORY_PERCEPTION_OF_PAIN	0.000246964	SPX/KCNA1/VIP/TAC1/TAFA4/PENK/TACR1/GRIN2A/TMEM100/HTR2A/CNR1/NDN/CXCL12/IL33/GIP/HOXD1/CNR2/MMP24/BDKRB1/CCL2/CCN3/UCHL1/KCND2/NLGN2/PROK2/CHRNB2/P2RY1/CXCR4/OXT/EDNRB/SCN3B/BACE1/LXN/SCN9A

GOBP_REGULATION_OF_DEVELOPMENTAL_GROWTH	GOBP_REGULATION_OF_DEVELOPMENTAL_GROWTH	0.000246258	SYT4/GHRH/MAEL/NKX2-5/EPHA7/AKAP6/IGF1/GHR/AR/NOG/KCNK2/PI16/MAP1B/RGS4/NGF/ZFPM2/FGF2/PLXNA4/IGF2/CXCL12/WT1/SEMA3E/NTN1/MAPT/RND2/GLI1/LPAR3/NKX6-1/FGF13/RGS2/L1CAM/RBP4/GAMT/PRKN/HOPX/SMO/FN1/CGA/ADRB2/SPART/SOX15/CRABP2/FOXC2/SEMA6A/FOXS1/DUSP9/SEMA6D/ISLR2/TGFBR3/COLQ/MEF2C/MIR222/NRCAM/PTCH1/SEMA6C/SYT1/ACACB/TBX5/SEMA3G/MAP2/SEMA5B/SEMA3D/CXCR4/MUSK/STC2/HAMP/SEMA3C/MYOZ1/NRP1/NPPC

GOMF_PASSIVE_TRANSMEMBRANE_TRANSPORTER_ACTIVITY	GOMF_PASSIVE_TRANSMEMBRANE_TRANSPORTER_ACTIVITY	0.000245268	AQP8/KCNA1/SCN7A/GRIK3/AQP12A/CHRNA3/KCNB1/KCNMA1/KCNK3/GRIK1/GPM6A/KCNMB1/GRIN2A/KCNA5/ABCC9/RYR3/GRIK5/KCNK2/KCNH6/GLRB/KCNT2/KCNIP1/CACNA1C/P2RX2/TRPC4/CACNG4/TMEM63C/CNR1/TRPC1/BEST2/CACNA1H/SCN4B/KCNJ8/CACNB2/KCNE4/SLC17A7/KCNH2/CHRNB4/NCS1/GJC1/P2RX1/TRPM6/SCN3A/CLCA1/SLC1A7/ANO5/PKD2/CACNA2D3/CACNA2D1/AQP10/CACNA1A/CLIC4/TMEM150C/KCNG1/SNAP25/RHCG/PIEZO2/KCNQ3/KCND1/GJA1/SLC24A3/SLC14A1/RHBG/CNGA1/CALHM5/KCNS1/KCND2/PDPN/ITPR1/RYR2/CLIC2
GOCC_MITOCHONDRIAL_SMALL_RIBOSOMAL_SUBUNIT	GOCC_MITOCHONDRIAL_SMALL_RIBOSOMAL_SUBUNIT	0.000247469	MRPS11/MRPS18B/MRPS9/MRPL42/MRPS18A/MRPS22/PTCD3/MRPS26/MRPS5/CHCHD1/MRPS18C/MRPS12/MRPS25/AURKAIP1/MRPS28/MRPS27/MRPS16/MRPS34/MRPS35/MRPS2

GOBP_TETRAHYDROFOLATE_METABOLIC_PROCESS	GOBP_TETRAHYDROFOLATE_METABOLIC_PROCESS	0.000254912	MTHFD2L/ALDH1L1/GART/ATIC/MTHFR/GCH1/DHFRP1/SHMT1/MTHFD2/MTHFD1L/DHFR/MTHFD1/SHMT2/TYMS
GOBP_REGULATION_OF_COAGULATION	GOBP_REGULATION_OF_COAGULATION	0.000257842	APOH/VTN/FGA/F2/FGG/ADAMTS18/F7/SERPINF2/CD36/PRKG1/THBS1/CAV1/SERPINE2/SERPINE1/EMILIN1/PDGFRA/PROS1/KRT1/THBD/FAP/FGB/SERPING1/TFPI
GOBP_TELOMERE_MAINTENANCE_IN_RESPONSE_TO_DNA_DAMAGE	GOBP_TELOMERE_MAINTENANCE_IN_RESPONSE_TO_DNA_DAMAGE	0.000260198	SHLD2/SHLD3/MAD2L2/RIF1/TERF2/TFIP11/ACTL6A/RTEL1/UCHL5/ACD/INO80D/RUVBL1/XRCC1/INO80E/INO80/RUVBL2/MCRS1/NFRKB/DCLRE1B/DCLRE1A
GOBP_PROTEIN_KINASE_A_SIGNALING	GOBP_PROTEIN_KINASE_A_SIGNALING	0.000260609	GCG/ADIPOQ/AKAP6/MYOM1/AKAP12/EDNRA/ADRB2/LRRK2/PRKACB/RDX/RAMP3

GOBP_POSITIVE_REGULATION_OF_TELOMERE_MAINTENANCE_VIA_TELOMERE_LENGTHENING	GOBP_POSITIVE_REGULATION_OF_TELOMERE_MAINTENANCE_VIA_TELOMERE_LENGTHENING	0.000261726	CCT6A/PNKP/MAP3K4/FBXO4/ATR/PARN/RTEL1/MAP2K7/XRCC5/NAF1/ACD/DKC1/MAPK3/TCP1/NEK2/CCT8/CCT2/HNRNPA2B1/CCT3/CCT4/CCT7/CCT5/AURKB/HNRNPA1/PRKCQ/TNKS/MAPK15
GOBP_REGULATION_OF_TUBE_SIZE	GOBP_REGULATION_OF_TUBE_SIZE	0.000269634	NPPB/ATP1A2/AGTR1/FGA/TACR1/KCNMA1/KCNMB1/NPR3/KCNA5/HTR2A/ACTA2/FGG/HTR2B/ADRA1D/SMTNL2/GUCY1A1/SERPINF2/AGT/PRKG1/VSTM4/ADRA1B/P2RX1/CAV1/EDNRA/RGS2/NPR1/ITGA1/SLC8A1/MMP2/COMP/AVPR1A/CYSLTR1/HBB/SOD3/ADRB2/OXTR/FOXC2

GOBP_ANTIMICROBIAL_HUMORAL_RESPONSE	GOBP_ANTIMICROBIAL_HUMORAL_RESPONSE	0.000275778	PLA2G1B/ELANE/PRTN3/REG3G/AZU1/DEFA4/RNASE3/FGA/REG1B/CTSG/F2/RNASE2/TF/DEFA6/CXCL6/PRSS2/COLEC11/CAMP/S100A9/DEFB126/PGLYRP1
GOBP_REGULATION_OF_NEURON_DIFFERENTIATION	GOBP_REGULATION_OF_NEURON_DIFFERENTIATION	0.000277259	NKX2-2/RELN/SFRP1/NEUROD1/NKX2-5/ASCL1/SFRP2/GDF6/MAP1B/NAP1L2/NGF/FGF2/PCP4/DAB1/DIXDC1/NLGN1/CDON/CXCL12/GLI3/ZEB1/BEND6/CNTN4/NKX6-1/FGFR1/MEIS1/FEZ1/HEYL/HOXA2/SHH/PBX1/DKK1/ISL1/NTF3/GDF7/LRRK2/ID4/ATOH1/BRINP3/MEF2C/NRCAM/TCF4
GOBP_REGULATION_OF_APPETITE	GOBP_REGULATION_OF_APPETITE	0.000277936	SPX/CARTPT/SCT/NPY/SCTR
GOBP_REGULATION_OF_DNA_TEMPLATED_DNA_REPLICATION_INITIATION	GOBP_REGULATION_OF_DNA_TEMPLATED_DNA_REPLICATION_INITIATION	0.000280489	WRNIP1/NBN/MCM7/MCM6/TICRR/MCM3/MCM5/MCIDAS/MCM4/MCM2/CDT1

GOBP_PROTEIN_LOCALIZATION_TO_MITOCHONDRION	GOBP_PROTEIN_LOCALIZATION_TO_MITOCHONDRION	0.000284463	TIMM8A/UBE2L3/USP36/TOMM40L/MAPK8/HTRA2/NMT1/LMAN1/PAM16/TIMM17B/HAX1/OXA1L/DNAJC15/SAE1/IMMP1L/ATG13/TIMM23/CSNK2A2/TIMM23B/HUWE1/TIMM17A/UBE2D3/FBXO7/BAP1/RAC2/MAIP1/TIMM50/MTCH2/UBE2J2/TOMM70/MFN2/TIMM22/BID/RALA/TIMM8B/SAMM50/TOMM34/HSP90AA1/TOMM22/CALM3/MTX1/MTX2/CHCHD4/MARCHF5/GSK3A/PMPCB/HSPA4/GRPEL2/HPS4/BCS1L/TIMM29/BAX/TOMM5/DNAJA1/AKT1/NDUFA13/BAG4/AP3B1/SREBF2/BCAP31/ATP5IF1/AIFM1/TIMM21/MIPEP/MICALL2/GRPEL1/FZD5/TIMM13/HSPD1/PMPCA/TOMM40/TIMM44/COX18/BBC3/TSPO/HK2/GZMB/SREBF1
GOBP_RNA_PHOSPHODIESTER_BOND_HYDROLYSIS_EXONUCLEOLYTIC	GOBP_RNA_PHOSPHODIESTER_BOND_HYDROLYSIS_EXONUCLEOLYTIC	0.000285987	DIS3L2/CNOT8/EXOSC8/USB1/PARN/CNOT6L/CPSF3/DIS3L/DIS3/PAN2/ISG20L2/XRN1/CNOT7/EXOSC10/EXOSC2/EXOSC7/EXOSC5/PDE12/CNOT6/EXOSC9/EXOSC4/CNOT1/TOE1/DCPS/ERI2/ISG20/POLRMT/ZC3H12A/ERI1

GOMF_ALPHA_ACTININ_BINDING	GOMF_ALPHA_ACTININ_BINDING	0.000288655	SYNPO2/LDB3/KCNA5/PDLIM3/CACNA1C/PALLD/PDLIM4/PKD2/PDLIM7
GOCC_GLUTAMATERGIC_SYNAPSE	GOCC_GLUTAMATERGIC_SYNAPSE	0.000288849	SYT4/KCNA1/CHRM2/GRIK3/EPHA7/GPM6A/GRIN2A/SPARCL1/HTR2A/NPTX2/GRIK5/PLPPR4/PAK3/ADAM23/CACNG4/CNR1/NLGN4X/NLGN1/PTPRD/FXYD6/NPTX1/ADCY1/GUCY1A1/CALY/WASF3/NPTXR/ACTC1/NLGN4Y/RGS7BP/SV2A/P2RX1/ATP2B4/GUCY1B1/PRKAR2B/HPCA/NRP2/CLSTN2/SH3GL2/CDH11/CTTNBP2/SNAP25/BSN/EFNB3/CAMKV/NLGN3/LRRK2/LRRC4/DLG4/CPT1C/LRRC4C/KCND2/ACTN1/NRCAM/APBA1/GABBR1/GABRR1/SYT1/ITGB3/PCDH17/P2RY1/ARC/ADGRL3/CADPS/NRP1/FZD4/LRRC4B/PFN2/GRID1/DLGAP3/DNM1/TAMALIN/NTNG2/PLAT/CAMK4/CNIH2/GHRL/ARHGAP22

GOBP_REGULATION_OF_SYNAPTIC_PLASTICITY	GOBP_REGULATION_OF_SYNAPTIC_PLASTICITY	0.000291416	SYT4/RELN/KCNB1/CHRDL1/SCGN/GRIN2A/SCT/SLC8A2/NOG/IGSF11/MAP1B/PTN/MIR320C2/MAP1A/ADCY1/GIP/CNTN4/AGT/MAPT/NFATC4/CX3CR1/SORCS2/SERPINE2/PRRT1/FAM107A/MIR342/SCTR/SNAP25/MPP2/BRSK1/TSHZ3/MIR320D1/DLG4/NEURL1/PRNP/MEF2C/S100B/JPH3/RASGRF2/MIR95/ADGRB1/SYP/ARC/KIT/SYNGR1

GOBP_ENDOTHELIAL_CELL_MIGRATION	GOBP_ENDOTHELIAL_CELL_MIGRATION	0.000292704	APOA1/MIR483/APOH/MEOX2/SLIT2/BMPER/CYP1B1/PTN/SMOC2/FGF2/GREM1/DCN/PRKD1/AGT/VSTM4/AKT3/ATP2B4/THBS1/SERPINF1/LGALS12/ATOH8/FSTL1/FGFR1/ANGPT1/RHOJ/NRP2/MIR342/CCBE1/MIR27B/CCN3/SNAI2/FOXC2/FGF1/ADGRA2/MIR149/PTP4A3/FAP/SRPX2/RHOB/HSPB1/STC1/SCG2/TEK/MEF2C/EGR3/TNFSF12/SPARC/MIR10B/ADAMTS9/PTPRM/GPLD1/ITGB3/MMRN2/VEGFC/ADGRB1/ROBO1/MIR196A1/STARD13/CLEC14A/PLPP3/NRP1/RRAS/EDN2

GOBP_RNA_PHOSPHODIESTER_BOND_HYDROLYSIS	GOBP_RNA_PHOSPHODIESTER_BOND_HYDROLYSIS	0.000293919	CPSF6/DBR1/PLD6/DIS3L2/RNASET2/SLFN13/CNOT8/EXOSC8/KRI1/CPSF2/USB1/PARN/CSTF3/CNOT6L/CPSF3/DIS3L/DIS3/RIDA/PAN2/KHNYN/CPSF4/PPP1R8/ISG20L2/XRN1/TSEN34/AZGP1/TSEN2/POP4/TUT1/ABT1/NCBP1/FIP1L1/BMS1/UTP20/RCL1/CNOT7/EXOSC10/INTS11/SND1/EXOSC2/EXOSC7/EXOSC5/NUDT21/POP7/TSR1/PDE12/NOP9/RPP25/CNOT6/AGO2/EXOSC9/CSTF2/POLR3K/YBEY/EXOSC4/NUDT12/CNOT1/TOE1/ELAC2/TBL3/CPSF1/DCPS/ERI2/NHP2/RRS1/NOB1/ISG20/HELZ2/POLRMT/ZC3H12A/BOP1/ERI1/POP1/EXO1/FEN1/ENDOG/PIWIL1/NOP14/RNASE7/ERN2
GOBP_BASE_EXCISION_REPAIR	GOBP_BASE_EXCISION_REPAIR	0.000298669	LIG3/MBD4/NEIL1/SMUG1/ERCC6/OGG1/HUWE1/NEIL2/TDG/RPA3/PNKP/RPA2/RPS3/PARG/POLL/RPA1/XRCC1/ADPRS/POLB/APEX2/ERCC5/MPG/NTHL1/WRN/POLG/UNG/SIRT6/NEIL3/POLE/PCNA/POLQ/LIG1/FEN1/DNA2/POLD1

GOBP_REGULATION_OF_NON_CANONICAL_WNT_SIGNALING_PATHWAY	GOBP_REGULATION_OF_NON_CANONICAL_WNT_SIGNALING_PATHWAY	0.00030191	SFRP1/SFRP2/GPC3/RSPO3/SFRP4/DAAM2/DACT1/DAB2/ANKRD6/DKK1
GOBP_DNA_SYNTHESIS_INVOLVED_IN_DNA_REPAIR	GOBP_DNA_SYNTHESIS_INVOLVED_IN_DNA_REPAIR	0.000304226	SIRT1/CDKN2D/POLDIP2/RCHY1/MAD2L2/RFC3/USP10/POLA1/HROB/POLH/POLE2/WRNIP1/WRN/RRM2B/VCP/USP1/POLD3/PARP10/POLE/PCLAF/POLD2/RRM1/PCNA/DTL/POLD1/USP43
GOBP_POSITIVE_REGULATION_OF_HORMONE_SECRETION	GOBP_POSITIVE_REGULATION_OF_HORMONE_SECRETION	0.000305591	GCG/UCN3/GHRH/NNAT/FGA/TACR1/ADCYAP1/SCT/FGG/GRP/GIP/RFX6/NKX6-1/RBP4/NR1H4/DAB2/ISL1/FFAR4/RETN/GPR27/FGB/NLGN2/INHBA/INHA/BLK/GABBR1/GPLD1/MYRIP/P2RY1/GPER1

GOBP_POSITIVE_REGULATION_OF_RESPONSE_TO_WOUNDING	GOBP_POSITIVE_REGULATION_OF_RESPONSE_TO_WOUNDING	0.00031125	REG3G/APOH/VTN/MYLK/F2/FERMT2/FLNA/DDR2/PTN/SMOC2/F7/SERPINF2/CD36/THBS1/SERPINE1/EMILIN1/FOXC2/CCN4/THBD/MIR222
GOBP_CARDIAC_MUSCLE_TISSUE_MORPHOGENESIS	GOBP_CARDIAC_MUSCLE_TISSUE_MORPHOGENESIS	0.00031639	HAND1/NKX2-5/ANKRD1/MYL3/NOG/ZFPM2/TPM1/ACTC1/EDNRA/TGFB2/ISL1/FOXC2/S1PR1/HEG1/RYR2/TGFBR3
GOBP_ADULT_LOCOMOTORY_BEHAVIOR	GOBP_ADULT_LOCOMOTORY_BEHAVIOR	0.000316481	ATP1A2/SEZ6/TMOD1/GLRB/DAB1/HOXD10/CXCL12/GIP/LGI4/CHL1/PRKN/PREX2/SLITRK6/PBX3/EFNB3/UCHL1/SNCG/NLGN2/FGF12/HOXD9

GOBP_MORPHOGENESIS_OF_A_BRANCHING_STRUCTURE	GOBP_MORPHOGENESIS_OF_A_BRANCHING_STRUCTURE	0.00031565	SOX10/SFRP1/FGF10/EPHA7/SLIT2/PGR/FGF7/TMEM59L/AR/SIX2/SFRP2/NOG/ADAMTS16/GPC3/RSPO3/FGF2/GREM1/WT1/GLI3/SEMA3E/GREB1L/AGT/FOXF1/RSPO2/NFATC4/LAMA1/HHIP/EDNRA/FGF13/HGF/PKD2/ESR1/SHH/CLIC4/SMO/PBX1/GDF7/WNT2B/TCF21/LRRK2/SNAI2/FOXC2/DCHS1/CCL11/FGF1
GOBP_REGULATION_OF_BONE_MINERALIZATION	GOBP_REGULATION_OF_BONE_MINERALIZATION	0.000318064	AHSG/OMD/MGP/DDR2/PTN/GREM1/GPM6B/TGFB3/CCN1/SLC8A1/TMEM119/COMP/OSR1/ADRB2/NELL1/WNT10B/S1PR1/BMPR1B/KL/MEF2C/LTBP3/TWIST1/ACVR1/SRGN

GOBP_REGULATION_OF_MUSCLE_CELL_DIFFERENTIATION	GOBP_REGULATION_OF_MUSCLE_CELL_DIFFERENTIATION	0.000322307	SMYD1/MYOCD/NKX2-5/RBPMS2/MIR145/AKAP6/IGF1/RBM24/PI16/RGS4/IGF2/LAMA2/ZEB1/LAMA1/PRDM6/PLPP7/DMPK/RGS2/LMOD3/TMEM119/HOPX/SHH/DKK1/EFEMP2/FZD7/CCN3/NID1/MEF2C/MIR222/LAMC1/MAMSTR
GOBP_RENAL_SODIUM_EXCRETION	GOBP_RENAL_SODIUM_EXCRETION	0.000327641	SPX/NPPB/AGTR1/TACR1/AGT/NPR1/CORIN/AVPR1A/AVPR2/OXT/EDNRB
GOCC_POSTSYNAPTIC_SPECIALIZATION_MEMBRANE	GOCC_POSTSYNAPTIC_SPECIALIZATION_MEMBRANE	0.000328408	EPHA7/GRIN2A/GRIK5/CDH2/PLPPR4/CACNA1C/CACNG4/NLGN4X/NLGN1/ADCY1/NLGN4Y/DLG2/RGS7BP/LIN7A/SORCS2/CLSTN2/PRRT1/EFNB3/NLGN3/LRRC4/DLG4/LRRC4C/KCND2/SYNDIG1/NRCAM/NLGN2/CHRNB2/ARC/LRRC4B/GRID1

GOBP_CYCLIC_NUCLEOTIDE_METABOLIC_PROCESS	GOBP_CYCLIC_NUCLEOTIDE_METABOLIC_PROCESS	0.000330753	NPPB/ADCY5/ADCY2/PDE7B/ADCY1/GUCY1A1/PDE9A/GUCY1B1/PDE2A/NPR1/PDE8B/GUCY1A2/PTHLH/PDE5A/NPR2/NPPC/RORA/ADCY4
GOBP_PROTEIN_LOCALIZATION_TO_CHROMOSOME_CENTROMERIC_REGION	GOBP_PROTEIN_LOCALIZATION_TO_CHROMOSOME_CENTROMERIC_REGION	0.000331936	H4C13/CHAMP1/CTCF/SMC5/SPDL1/RB1/H4C9/H2BC11/H4C4/H4C3/H4C2/H4C1/MTBP/ZW10/TTK/KNTC1/ZWILCH/AURKB/NDC80/BUB3/CENPA/CDK1/BUB1B/RCC2/HASPIN/KNL1/CDT1
GOBP_POSITIVE_REGULATION_OF_WOUND_HEALING	GOBP_POSITIVE_REGULATION_OF_WOUND_HEALING	0.000333968	REG3G/APOH/VTN/MYLK/F2/FERMT2/DDR2/SMOC2/F7/SERPINF2/CD36/THBS1/SERPINE1/EMILIN1/FOXC2/CCN4/THBD

GOBP_NEGATIVE_REGULATION_OF_TRANSMEMBRANE_RECEPTOR_PROTEIN_SERINE_THREONINE_KINASE_SIGNALING_PATHWAY	GOBP_NEGATIVE_REGULATION_OF_TRANSMEMBRANE_RECEPTOR_PROTEIN_SERINE_THREONINE_KINASE_SIGNALING_PATHWAY	0.000337069	CER1/SFRP1/CHRDL1/RBPMS2/CIDEA/CHRDL2/CILP/SFRP2/NOG/GREM2/BMPER/GREM1/ASPN/TGFB1I1/CHRD/TGFB3/CAV1/LTBP1/FBN1/ONECUT1/MIR342/EMILIN1/DKK1/SPART/CCN3/IGSF1/TGFBR3/HSPA1A/LDLRAD4/TET1/VASN/TMPRSS6/ACVR1/LRP1/HTRA1/SPRED3/CD109/FZD1

GOBP_DNA_BIOSYNTHETIC_PROCESS	GOBP_DNA_BIOSYNTHETIC_PROCESS	0.000337519	MAP2K7/SMG5/XRCC5/NAF1/RFC1/USP10/XRN1/ACD/POLA1/PARP4/HROB/POLL/PTK2B/RPA1/POLH/STN1/LIN9/POLM/POLE2/POLB/MYC/SRC/NAT10/DKC1/HNRNPU/PRKD2/HNRNPC/MAPK3/RFC4/TCP1/EXOSC10/TK2/PTGES3/NEK2/HNRNPD/PIF1/MEN1/POLE3/CCT8/CCT2/HNRNPA2B1/CCT3/DSCC1/GFER/WRNIP1/WRN/SH2B1/POLG/PINX1/TOM1L1/KLF4/CCT4/RRM2B/RFC5/CCT7/GINS1/CCT5/VCP/AURKB/USP1/RFC2/FGFR4/POLD3/GAR1/TELO2/GINS2/HNRNPA1/WRAP53/PARP10/NHP2/PRKCQ/TFDP1/TNKS/POLE/PCLAF/NIBAN2/POLD2/CENPF/RRM1/CHEK1/PCNA/CHTF18/PARP3/GINS3/POLQ/TP53/GRHL2/LIG1/TERT/MAPK15/DTL/GINS4/POLD1/TYMS/TK1/USP43/SMPD3
GOMF_FIBRONECTIN_BINDING	GOMF_FIBRONECTIN_BINDING	0.000339934	MYOC/CCDC80/SFRP2/SSC5D/FBLN1/THBS1/IGFBP6/MMP2/IGFBP5/CTSK/CCN2
GOCC_ENDOSOME_LUMEN	GOCC_ENDOSOME_LUMEN	0.00034084	PRF1/JAK2/SFTPB/PGA3/PGA5/PGA4/CSH1

GOBP_REGULATION_OF_PROTEIN_SECRETION	GOBP_REGULATION_OF_PROTEIN_SECRETION	0.000347831	PLA2G1B/GCG/SYT4/UCN3/CARTPT/CELA2A/SFRP1/NEUROD1/NNAT/KCNB1/FGA/PCSK1/ANKRD1/ADCY5/KCNA5/IGF1/FGG/CNR1/MYOM1/GIP/CHGA/TGFB3/APBB1/RFX6/NKX6-1/TGFB2/RBP4/IL6/PRKN/MYH10/NR1H4/SNAP25/ISL1/CCN3/CD200/GPR27/KLF7/FGB/NLGN2/BLK/GPLD1/MYRIP/WLS/VEGFC
GOBP_MONOSACCHARIDE_CATABOLIC_PROCESS	GOBP_MONOSACCHARIDE_CATABOLIC_PROCESS	0.000350548	GLB1/GLYCTK/GALK1/RBKS/TIGAR/FUT6/NUDT5/ENO2/FOXK2/FOXK1/BAD/FUT9/XYLB/DHDH/LRP5/HK3/FUT8/GALM/PGK1/PFKL/TPI1/TKFC/FUT4/GALE/TP53/FUT10/MIR210/ENO1/PFKP/PFKFB2/FUT2/PKM/HK2

GOMF_4_IRON_4_SULFUR_CLUSTER_BINDING	GOMF_4_IRON_4_SULFUR_CLUSTER_BINDING	0.000353686	NDUFS8/ETFDH/ERCC2/DPYD/ACO1/IREB2/DPH1/RTEL1/NDUFS2/ABCE1/NDUFS1/PRIM2/NUBP1/LIAS/NTHL1/CIAPIN1/ELP3/NUBP2/SDHB/CIAO3/DDX11/ACO2/DPH2/NDUFV1/POLE/NDUFS7/PPAT/DNA2/POLD1/BRIP1
GOBP_EPITHELIAL_TUBE_MORPHOGENESIS	GOBP_EPITHELIAL_TUBE_MORPHOGENESIS	0.000355649	HAND1/SFRP1/HAND2/NKX2-5/FGF10/EPHA7/TRIM71/RGMA/SLIT2/PGR/ASB2/TMEM59L/AR/SIX2/SFRP2/NOG/ADAMTS16/GPC3/FGF2/GREM1/WT1/GLI3/SEMA3E/NTN1/GREB1L/AGT/PRICKLE1/FOXF1/RSPO2/NFATC4/LAMA1/SALL4/HHIP/EDNRA/NODAL/TGFB2/PKD2/ESR1/SHH/CLIC4/SMO/OSR1/PBX1/DLC1/GDF7/WNT2B/TCF21/FOXC2/DCHS1/CCL11/FGF1/CC2D2A/ILK/SOX17/RHOB/CXCR2/RYR2/MTSS1/MEF2C/PTCH1/NR3C1/TWIST1/IRX3/MMRN2/HOXA5/CTHRC1/CXCR4/VANGL2/LEF1/STARD13/ACVR1/PRKACB/GLI2/RASIP1/NDRG4/NRP1/APLNR/TIE1/MICAL2/BMP5/FGFR2/FZD1/KLHL3

GOBP_INNATE_IMMUNE_RESPONSE_IN_MUCOSA	GOBP_INNATE_IMMUNE_RESPONSE_IN_MUCOSA	0.000358061	PLA2G1B/DEFA4/RNASE3/RNASE2/DEFA6/CAMP/APOA4
GOBP_CLEAVAGE_INVOLVED_IN_RRNA_PROCESSING	GOBP_CLEAVAGE_INVOLVED_IN_RRNA_PROCESSING	0.000358833	ABT1/BMS1/UTP20/RCL1/EXOSC10/EXOSC2/EXOSC7/TSR1/NOP9/EXOSC9/TBL3/ERI2/NHP2/RRS1/NOB1/BOP1/ERI1/NOP14
GOBP_NEGATIVE_REGULATION_OF_POTASSIUM_ION_TRANSMEMBRANE_TRANSPORT	GOBP_NEGATIVE_REGULATION_OF_POTASSIUM_ION_TRANSMEMBRANE_TRANSPORT	0.000359634	CASQ2/ACTN2/GRP/RGS4/KCNE4/KCNH2/CAV1
GOCC_PRECATALYTIC_SPLICEOSOME	GOCC_PRECATALYTIC_SPLICEOSOME	0.000364364	SNU13/SNRPD2/IK/MAGOHB/SNRPD3/LSM5/LSM6/SNRPG/PRPF38B/PRPF6/SF3B1/PRPF38A/DHX16/SF3A1/PRPF31/LSM3/SNRPF/MFAP1/LSM2/SNRPA1/PHF5A/SMU1/SART1/SF3A3/LSM7/SNRPB/EFTUD2/PRPF8/SRRM2/BUD13/SNRNP200/SF3B2/PRPF4/SNRPD1/TXNL4A/SF3A2/SF3B5/SF3B3/LSM4

GOBP_HEART_GROWTH	GOBP_HEART_GROWTH	0.000364003	NKX2-5/AKAP6/IGF1/NOG/KCNK2/PI16/RGS4/ZFPM2/FGF2/WT1/AGT/GLI1/RGS2/TGFB2/RBP4/FOXC2/SORBS2/DUSP9/S1PR1/TENM4/HEG1/TGFBR3/MEF2C/MIR222/ACACB/TBX5/HAMP/NDRG4
GOBP_SERINE_FAMILY_AMINO_ACID_BIOSYNTHETIC_PROCESS	GOBP_SERINE_FAMILY_AMINO_ACID_BIOSYNTHETIC_PROCESS	0.000366492	DHFRP1/SHMT1/DHFR/MTHFD1/SHMT2/PSAT1/CTH
GOBP_MONOAMINE_TRANSPORT	GOBP_MONOAMINE_TRANSPORT	0.000367171	SYT4/CARTPT/VIP/KCNB1/HTR2A/SLC18A2/CNR1/GPM6B/CXCL12/AGT/CHGA/P2RX1/PRKN/SYT11/OXTR/SNCG/SLC29A4/RAB3B/SLC18A1/GABBR1/SYT1/ITGB3/ADORA3/CHRNB2/P2RY1/SYT5/OXT

GOBP_SMAD_PROTEIN_SIGNAL_TRANSDUCTION	GOBP_SMAD_PROTEIN_SIGNAL_TRANSDUCTION	0.000367526	AFP/BMP3/CILP/GDF6/TF/GDF10/TGFB3/LEFTY2/ATOH8/NODAL/TGFB2/WWTR1/RBPMS/SMAD9/DAB2/GDF9/GDF7/CCN3/VIM/INHBA/INHA
GOMF_EXOPEPTIDASE_ACTIVITY	GOMF_EXOPEPTIDASE_ACTIVITY	0.000367046	CPA1/CPA2/DPP6/CPXM2/CPE/CPN1/DPEP3/CPA3/XPNPEP2/NAALAD2/MMP16/CPXM1/TRHDE/DPEP1/CPA4/CPA6/ENPEP/FAP/CPVL/AEBP1/VASH2
GOBP_PLATELET_AGGREGATION	GOBP_PLATELET_AGGREGATION	0.000368791	CELA2A/FGA/CTSG/MYL9/FGG/ADAMTS18/FLNA/CSRP1/MMRN1/PRKG1/SERPINE2/COMP/IL6/HBB/PDGFRA/FN1/TSPAN32/ILK/PDPN/HSPB1/FGB/ACTN1/BLK/ITGB3
GOBP_FIBRINOLYSIS	GOBP_FIBRINOLYSIS	0.000371075	APOH/VTN/FGA/F2/FGG/SERPINF2/THBS1/SERPINE1/PROS1/KRT1/THBD/FAP/FGB/SERPING1

GOBP_OVULATION	GOBP_OVULATION	0.000371929	AFP/PGR/ADAMTS1/RGS2/MMP2/MMP19
GOBP_REGULATION_OF_RENAL_SYSTEM_PROCESS	GOBP_REGULATION_OF_RENAL_SYSTEM_PROCESS	0.000374799	SPX/NPPB/AGTR1/ADIPOQ/TACR1/AGT/NPR1/CORIN/AVPR1A/AVPR2/STC1/CORO2B/OXT/EDNRB
GOBP_MATURATION_OF_SSU_RRNA_FROM_TRICISTRONIC_RRNA_TRANSCRIPT_SSU_RRNA_5_8S_RRNA_LSU_RRNA	GOBP_MATURATION_OF_SSU_RRNA_FROM_TRICISTRONIC_RRNA_TRANSCRIPT_SSU_RRNA_5_8S_RRNA_LSU_RRNA	0.000377316	RRP36/RPS21/RPS8/RPS16/UTP25/ERCC2/RPS14/DCAF13/WDR46/KRI1/HEATR1/NOL10/UTP3/MRPS11/UTP4/WDR43/ABT1/RPS19/BMS1/UTP20/RCL1/DHX37/TSR1/BYSL/NOP9/HELB/TBL3/RRS1/PWP2/NOP14

GOBP_NEGATIVE_REGULATION_OF_ION_TRANSPORT	GOBP_NEGATIVE_REGULATION_OF_ION_TRANSPORT	0.000378475	VIP/CASQ2/ATP1A2/ACTN2/PLN/HTR2A/GRP/RGS4/CNR1/SFRP4/GPM6B/KCNE4/KCNH2/CAV1/GEM/RGS2/SERPINE2/PKD2/OSR1/SYT11/REM1/SLN/RRAD/STC1/CLIC2/GABBR1/TWIST1/P2RY1/GABRE/HAMP/KCNAB1
GOBP_RESPONSE_TO_METAL_ION	GOBP_RESPONSE_TO_METAL_ION	0.000378594	CPA1/SYT4/KCNA1/CASQ2/PENK/CER1/FABP4/KCNB1/FGA/KCNMA1/KCNK3/PCSK1/ASCL1/KCNMB1/SLC13A5/FGG/RYR3/PRKAA2/TF/TRPC1/CACNA1H/NLGN1/ACTA1/NPTX1/ADCY1/CYBRD1/MAPT/DLG2/NFATC4/THBS1/SERPINF1/CAV1/S100A8/RASGRP2/SLC30A2/FIBIN/HPCA/FOSB/TNFRSF11B/PKD2/PRKN/SHH/CLIC4/SOD3/SYT11/ACER1/LRRK2/DPEP1/DLG4/CPNE8/PRNP/MTTP/RYR2/FGB/MEF2C/ITPKB/GPLD1/SYT1/SLC30A10/CYP1A1/RASA4B

GOBP_CELL_COMMUNICATION_BY_ELECTRICAL_COUPLING	GOBP_CELL_COMMUNICATION_BY_ELECTRICAL_COUPLING	0.000382522	KCNA1/CASQ2/ATP1A2/ATP1B2/CACNA1C/HRC/GJC1/CAV1/SLC8A1/GJA1/RYR2/IRX3/TBX5
GOBP_DOUBLE_STRAND_BREAK_REPAIR_VIA_NONHOMOLOGOUS_END_JOINING	GOBP_DOUBLE_STRAND_BREAK_REPAIR_VIA_NONHOMOLOGOUS_END_JOINING	0.000382471	SHLD2/SHLD3/XRCC6/TP53BP1/WAS/MAD2L2/PNKP/RIF1/SMC5/CYREN/TFIP11/DEK/PARP9/ZBTB7A/NHEJ1/NUDT16L1/XRCC5/SMCHD1/POLA1/PRPF19/POLL/POLM/XRCC1/DTX3L/PSMD14/POLB/HSF1/PAXX/HMCES/PRKDC/HMGB2/KMT5C/SIRT7/DCLRE1B/WRAP53/PARP3/POLQ/DCLRE1A/AUNIP/NSD2

GOBP_REGULATION_OF_MAP_KINASE_ACTIVITY	GOBP_REGULATION_OF_MAP_KINASE_ACTIVITY	0.000384502	PLA2G1B/ELANE/SFRP1/ADIPOQ/HTR2A/GHR/EPGN/SFRP2/HTR2B/TNXB/ROR2/FGF2/AGT/THBS1/CCL19/CAV1/FZD10/RGS2/FGFR1/TENM1/DKK1/NTF3/UCHL1/LRRK2/PDGFD/FGF1/DUSP1/NOX4/PDE5A/MEF2C/S100A12/PDGFC/ROBO1/FLT3/IL34/KIT/PDGFRB/DIRAS1/FZD4/MAP3K12

GOBP_NUCLEAR_TRANSCRIBED_MRNA_CATABOLIC_PROCESS	GOBP_NUCLEAR_TRANSCRIBED_MRNA_CATABOLIC_PROCESS	0.00039036	DXO/TENT4B/RC3H2/MAGOH/MTPAP/DIS3L2/CNOT8/TENT2/EXOSC8/GSPT1/CNOT3/SMG7/TOB1/PARN/CNOT6L/SMG1/TUT7/ZFP36L2/DIS3/TENT4A/PAN2/SMG5/THRAP3/XRN1/EIF3E/POLR2D/LSM7/CNOT11/TUT1/NCBP1/APOBEC1/SMG9/DHX9/SYNCRIP/CNOT10/EIF4A3/HNRNPU/CNOT7/EXOSC10/HNRNPD/DHX34/EXOSC2/EXOSC7/PABPC1/UPF1/CASC3/EXOSC5/YBX1/RNPS1/PDE12/CNOT6/PATL1/AGO2/EXOSC9/CNOT9/EXOSC4/PYM1/EDC4/CNOT1/DCPS/NBAS/LSM4/MRTO4/HELZ2/ZC3H12A/ERI1/HNRNPAB/NANOS3
GOCC_90S_PRERIBOSOME	GOCC_90S_PRERIBOSOME	0.000393963	KRI1/HEATR1/WDR12/UTP4/RSL1D1/IMP3/WDR36/RRP7A/BMS1/UTP20/NOP9/NOC4L/PES1/WDR3/TBL3/IMP4/BOP1/NOL6/PWP2/NOC2L/NOP14

GOBP_DNA_REPLICATION_DEPENDENT_CHROMATIN_ASSEMBLY	GOBP_DNA_REPLICATION_DEPENDENT_CHROMATIN_ASSEMBLY	0.000392876	H4C13/H3C12/H3C10/HAT1/H4C9/H3C1/H4C4/H4C3/NASP/H4C2/RBBP4/H4C1/H3C2/H3C7/H3C6/CHAF1A/H3C8/CHAF1B/H3C11/H3C3/ASF1B
GOCC_GLYCOPROTEIN_COMPLEX	GOCC_GLYCOPROTEIN_COMPLEX	0.000393348	PGM5/SGCA/DMD/FLNA/SGCD/SGCG/SSPN/SGCE
GOBP_VASCULAR_ASSOCIATED_SMOOTH_MUSCLE_CELL_PROLIFERATION	GOBP_VASCULAR_ASSOCIATED_SMOOTH_MUSCLE_CELL_PROLIFERATION	0.000391962	CNN1/ADIPOQ/MYOCD/IGF1/DDR2/FGF2/NR4A3/AGT/TPM1/PRKG1/TGFB3/ADAMTS1/MMP2/EFEMP2/MIR27B/IGFBP5/GJA1/MEF2C/MIR222/TAFA5
GOBP_REGULATION_OF_ACTIN_FILAMENT_BUNDLE_ASSEMBLY	GOBP_REGULATION_OF_ACTIN_FILAMENT_BUNDLE_ASSEMBLY	0.000397194	APOA1/SYNPO2/SFRP1/MYOC/TACR1/FERMT2/FLNA/PHLDB2/SERPINF2/TPM1/TGFB3/PPM1E/CGNL1/LPAR1/PRKN/DLC1/CCN2/SYNPO/ARHGAP28/MIR149/S1PR1/MTSS1/CORO2B/ARHGEF15/NRP1/RDX/PFN2

GOBP_VASCULAR_ASSOCIATED_SMOOTH_MUSCLE_CELL_MIGRATION	GOBP_VASCULAR_ASSOCIATED_SMOOTH_MUSCLE_CELL_MIGRATION	0.00040214	ADIPOQ/MYOCD/DDR2/NR4A3/AGT/TPM1/PRKG1/ADAMTS1/IGFBP5/MEF2C/TAFA5
GOBP_POSTSYNAPTIC_SPECIALIZATION_ORGANIZATION	GOBP_POSTSYNAPTIC_SPECIALIZATION_ORGANIZATION	0.000403852	RELN/NRXN2/GAP43/CDH2/NLGN1/PTPRD/NPTX1/CNTNAP1/LRRC4/TMEM108/DLG4/NLGN2
GOBP_NEGATIVE_REGULATION_OF_BMP_SIGNALING_PATHWAY	GOBP_NEGATIVE_REGULATION_OF_BMP_SIGNALING_PATHWAY	0.000404427	CER1/SFRP1/CHRDL1/RBPMS2/CHRDL2/SFRP2/NOG/GREM2/BMPER/GREM1/CHRD/FBN1/DKK1/SPART

GOBP_POSITIVE_REGULATION_OF_RESPONSE_TO_DNA_DAMAGE_STIMULUS	GOBP_POSITIVE_REGULATION_OF_RESPONSE_TO_DNA_DAMAGE_STIMULUS	0.000407247	ING3/ARID2/ARID1A/SLF2/FMR1/ACTR2/ERCC6/HMGB1/MGMT/SIRT1/YEATS4/EEF1E1/ACTR8/TRRAP/TIGAR/UBE2N/RNF168/UIMC1/RPL26/SHLD2/SHLD3/WAS/BCLAF1/MAD2L2/SMARCD1/EP400/PNKP/RIF1/BRD7/BRD8/ATR/RAD9A/MRGBP/CCDC117/RPS3/ZNHIT1/SMARCC1/PIAS4/ACTL6A/PARP9/KAT5/SPIDR/SMARCC2/SMCHD1/UCHL5/HDAC10/HDGFL2/INO80D/MMS19/FXR2/SMARCB1/FUS/BRCC3/DHX9/BRCA1/RUVBL1/XRCC1/DTX3L/INO80E/INO80/MYC/SLF1/RUVBL2/PRKCD/MCRS1/FANCB/PARP1/EGFR/PAXIP1/SPIRE2/NFRKB/BCL7A/PRKDC/NPAS2/TRIM28/DDX11/MSX1/KMT5C/SMARCD2/CCAR2/EYA3/USP1/SIRT6/RBBP8/NKX3-1/TELO2/SMARCE1/WRAP53/RAD51AP1/SMARCA4/TMEM161A/TTI2/FOXM1/[illegible]

GOBP_METAL_ION_HOMEOSTASIS	GOBP_METAL_ION_HOMEOSTASIS	0.000408253	ELANE/SPX/KCNA1/NPPB/CASQ2/ATP1A2/AGTR1/SCN7A/PLN/TACR1/KCNMA1/CASQ1/JPH2/SCGN/ANK2/AKAP6/F2/KCNA5/SLC8A2/HTR2A/RGN/DMD/RYR3/HTR2B/PRND/FLNA/ATP1B2/CACNA1C/SGCD/FGF2/TRPC4/TF/PTH1R/TRPC1/CXCL12/CYBRD1/HRC/SLC22A17/PRKD1/AGT/KCNH2/CCL21/SYPL2/SV2A/P2RX1/ATP2B4/CCL19/MICU3/DMPK/CAV1/S100A8/EDNRA/TMEM178A/SLC30A2/NPR1/SLC8A1/PKD2/BDKRB1/S100A9/PRKCB/PRKN/CDH23/CORIN/AVPR1A/STEAP4/UPK3A/SLC24A3/SCARA5/CCL11/AVPR2/PRNP/ITPR1/RYR2/STC1/KL/CLIC2/THY1/FAM20A/JPH3/ANXA6/MCOLN3/ITGB3/SLC30A10
GOBP_CELL_COMMUNICATION_INVOLVED_IN_CARDIAC_CONDUCTION	GOBP_CELL_COMMUNICATION_INVOLVED_IN_CARDIAC_CONDUCTION	0.000409435	CASQ2/ATP1A2/CTNNA3/ANK2/KCNA5/FLNA/ATP1B2/CACNA1C/SCN4B/HRC/CACNB2/GJC1/CAV1/TBX18/SLC8A1/CACNA2D1/GJA1/RYR2/IRX3/TBX5
GOBP_NEGATIVE_REGULATION_OF_INTERLEUKIN_8_	GOBP_NEGATIVE_REGULATION_OF_INTERLEUKIN_8_PRODUCTION	0.000411677	ELANE/BPI/MIR520C/SSC5D

GOBP_EXCRETION	GOBP_EXCRETION	0.000425622	SPX/NPPB/AGTR1/CHRNA3/TACR1/KCNMA1/AGT/CHRNB4/NPR1/CORIN/AVPR1A/AVPR2/STC1/CHRNB2/OXT/EDNRB/ABCG2
GOBP_POSITIVE_REGULATION_OF_DEVELOPMENTAL_GROWTH	GOBP_POSITIVE_REGULATION_OF_DEVELOPMENTAL_GROWTH	0.000425181	SYT4/GHRH/AKAP6/IGF1/GHR/MAP1B/NGF/ZFPM2/FGF2/IGF2/CXCL12/WT1/NTN1/MAPT/RND2/GLI1/LPAR3/L1CAM/PRKN/HOPX/SMO/FN1/SOX15/CRABP2/FOXS1/ISLR2/TGFBR3/MEF2C/MIR222/SYT1/ACACB/TBX5/CXCR4
GOBP_TRANSMISSION_OF_NERVE_IMPULSE	GOBP_TRANSMISSION_OF_NERVE_IMPULSE	0.000426436	CARTPT/KCNA1/SCN7A/NFASC/CACNG4/JAM3/CACNA1H/AGT/CNTNAP1/CHRNB4/P2RX1/SCN3A/AVPR1A/S1PR1/CLDN19/KCND2/NRCAM/FGF12/GPER1/SCN1B/SCN9A/GHRL/CLCN1/NTRK2

GOBP_POSITIVE_REGULATION_OF_CYTOSKELETON_ORGANIZATION	GOBP_POSITIVE_REGULATION_OF_CYTOSKELETON_ORGANIZATION	0.000429567	APOA1/SYNPO2/SFRP1/STMN2/ACTN2/MYOC/LMOD1/TACR1/CFL2/CCL26/FERMT2/MAP1B/NAV3/SERPINF2/WASF3/TPM1/CDC42EP3/CCL21/TGFB3/PPM1E/MAPT/CSF3/LPAR1/TENM1/ANKRD53/NTF3/KIRREL1/CCN2/SYNPO/CCL11/TEK/MTSS1/HSPA1A/WASF1/MAGEL2/DSTN/CCR7/ARHGEF15/GSN/NRP1/PFN2
GOBP_MEMBRANE_DEPOLARIZATION	GOBP_MEMBRANE_DEPOLARIZATION	0.000442212	ATP1A2/SCN7A/MYOC/FHL1/ANK2/CACNA1C/BEST2/DCN/CACNA1H/SCN4B/CACNB2/KCNH2/CAV1/SCN3A/SLC8A1/CACNA2D1/LRRK2/TBX5/CHRNB2/MLLT11/SCN3B/SCN1B/SCN9A

GOCC_INNER_MITOCHONDRIAL_MEMBRANE_PROTEIN_COMPLEX	GOCC_INNER_MITOCHONDRIAL_MEMBRANE_PROTEIN_COMPLEX	0.000445802	ATP5MC2/PAM16/NDUFB8/TIMM17B/NDUFC2/NDUFB5/DNAJC15/NDUFC1/MICU2/NDUFA1/COX7B/IMMP1L/TIMM23/NDUFB4/UQCRB/ATP5MG/TIMM23B/COX6B1/NDUFS8/UQCR11/NDUFB2/ATP5MF/COX7C/NDUFA8/TIMM17A/COX7A2L/ATP5F1C/NDUFA6/NDUFB10/ATP5PD/NDUFS5/COX7A2P2/NDUFB3/TIMM50/NDUFA12/NDUFA5/TIMM22/UQCRQ/NDUFA9/SAMM50/NDUFB6/CHCHD6/NDUFA11/NDUFAF1/NDUFS6/MTX1/MTX2/UQCRFS1/APOO/NDUFA10/NDUFS2/ATP5F1EP2/NDUFA2/NDUFS1/COX7A2/NDUFS3/MICOS13/SDHD/GRPEL2/MCUB/NDUFB7/MPC1/COX5B/BCS1L/AFG3L2/MICU1/ATP5PO/NDUFAB1/UQCR10/TIMM29/COX4I1/ATP5MC1/ATP5PB/COX6A1/IMMT/NDUFA13/SDHB/TIMM21/COX5A/UQCRC2/HSPA9/ATP5MC3/CHCHD3/ATP[illegible]

GOBP_SKIN_DEVELOPMENT	GOBP_SKIN_DEVELOPMENT	0.00044853	CYP27B1/STMN1/CYP26B1/TRIM16/TRAF3IP2/ITGA3/MED1/SLC27A4/IRF6/ELOVL1/CASP3/LGR4/SPRR2D/SOX21/EVPL/ITGB6/HDAC1/JAG1/ITGA2/IL18/SOX9/DHCR24/EZH2/S100A7/CDH3/MSX2/ADAM9/DNASE1L2/GRHL2/HPSE/KRT17/ST14/KRT80/KRT6B/VDR/SFN/JUP/PLAAT4/KDF1/FLNB/FOXQ1/ITGA6/FST/FOXC1/KLK5/OVOL2/KRT7/EDAR/ZBED2/EXPH5/FERMT1/APCDD1/FA2H/GJB3/ALOX12B/SOSTDC1/EPHA2/KRT8/ITGB4/ABCA12/KRT16/SPRR2E/KRT81/DKK4/LCE3E/LCE3D/KRT27/SPRR2B/SPRR2G
GOBP_GLUCOSE_CATABOLIC_PROCESS	GOBP_GLUCOSE_CATABOLIC_PROCESS	0.000452135	ENO2/FOXK2/FOXK1/BAD/LRP5/HK3/PGK1/PFKL/TPI1/TP53/MIR210/ENO1/PFKP/PFKFB2/PKM/HK2

GOBP_PEPTIDE_ANTIGEN_ASSEMBLY_WITH_MHC_PROTEIN_COMPLEX	GOBP_PEPTIDE_ANTIGEN_ASSEMBLY_WITH_MHC_PROTEIN_COMPLEX	0.000453064	TAPBPL/HLA-DPB1/PDIA3/TAPBP/CALR/HLA-DPA1/HLA-DMB/HLA-DOB/HLA-DRA/HLA-DQA2/HLA-DQA1/HLA-DRB5/HLA-DRB1/HLA-DOA/HLA-DQB1/HLA-DQB2/HLA-DMA
GOBP_RNA_POLYADENYLATION	GOBP_RNA_POLYADENYLATION	0.00045602	AHCYL1/CPEB3/PCF11/APLP1/SCAF8/YTHDC1/ZC3H14/CDC73/CLP1/PNPT1/PAPOLA/WDR33/APP/VIRMA/SYMPK/CPSF7/ZCCHC7/CPSF6/TENT4B/MTPAP/SSU72/TENT2/CPSF2/CSTF3/CPSF3/LEO1/TENT4A/TUT1/NCBP1/FIP1L1/HSF1/PABPC1/NUDT21/ZC3H3/CSTF2/SNRPA/CPSF1/GRSF1

GOBP_RENAL_SYSTEM_VASCULATURE_DEVELOPMENT	GOBP_RENAL_SYSTEM_VASCULATURE_DEVELOPMENT	0.000463636	ACTA2/WT1/EDNRA/ANGPT1/PKD2/PDGFRA/OSR1/TCF21/PDGFD/FOXC2/TEK/ITGB3/CD34/PDGFRB/PECAM1/NRP1/ANGPT2
GOBP_LIPID_CATABOLIC_PROCESS	GOBP_LIPID_CATABOLIC_PROCESS	0.000463059	CLPS/PLA2G1B/PNLIP/APOA2/CEL/PNLIPRP2/APOA1/LCT/APOC3/ADIPOQ/CIDEA/PLCD4/PLCXD3/PLIN1/CES1/CYP24A1/SULT2A1/SCT/APOB/PLA2G5/CYP1B1/PLA2G2C/CNR1/GPIHBP1/PLA2G12B/LGALS12/PLA2G4C/HCAR1/APOA4/CLPSL2/HSD17B6/PLAAT1/HSD11B1/HSD17B14/ACER1/DPEP1/CYP39A1/ACOX2/CPT1C/LPL/RARRES2/CYP27A1/PNPLA1/TWIST1/GPLD1/CYP3A4/ACACB
GOBP_REGULATION_OF_MEMBRANE_REPOLARIZATION	GOBP_REGULATION_OF_MEMBRANE_REPOLARIZATION	0.000466716	CASQ2/ANK2/AKAP6/KCNA5/KCNH6/FLNA/SCN4B/KCNE4/KCNH2/CAV1/CACNA2D1

GOBP_CHONDROCYTE_DIFFERENTIATION	GOBP_CHONDROCYTE_DIFFERENTIATION	0.00046714	COL2A1/SIX2/SFRP2/GDF6/EFEMP1/NKX3-2/ZBTB16/GREM1/PTH1R/GLI3/WNT9A/SOX5/CYTL1/COMP/COL3A1/OSR1/CCN3/WNT2B/WNT10B/SNAI2/CCN4/CCN2/PTHLH/MAF/TGFBI/BMPR1B/MEF2C/LTBP3/ANXA6/GPLD1/TRPS1/SULF1
GOBP_REGIONALIZATION	GOBP_REGIONALIZATION	0.000465785	NKX2-2/RELN/CER1/SFRP1/NEUROD1/NKX2-5/FGF10/MEOX2/ASCL1/ALDH1A2/BARX1/DLL3/AR/SIX2/SFRP2/NOG/GREM2/GPC3/FGF2/ZBTB16/GREM1/HOXD10/CDON/WT1/GLI3/CHRD/FOXF1/HOXA4/GLI1/TLL1/HHIP/SMARCD3/CRB2/NODAL/TBX18/NRP2/HEYL/TDRD10/HOXA2/SHH/SMO/OSR1/PBX1/DKK1/PBX3/ISL1/MEOX1/WNT2B/FOXC2/PIFO/FGF1/HOXD4/GPR161/TCF15/BMPR1B/SOX17/MEF2C/PTCH1/IRX3/WLS/DYNC2H1/CXXC4/HOXA5/ROBO1/HOXD9/ARC/LEF1/ACVR1/PRKACB/GLI2/SEMA3C

GOBP_PROTEIN_MONOUBIQUITINATION	GOBP_PROTEIN_MONOUBIQUITINATION	0.000469068	UBE2E1/PCGF1/KLHL12/TRIM37/FANCF/HUWE1/CUL3/TOPORS/PCGF6/RNF168/UBE2D3/UBE2R2/DDB1/UBE3D/FAAP100/CTR9/PDCD6/LEO1/MGRN1/KDM2B/ATXN7L3/BRCC3/BRCA1/DTX3L/PCGF5/FANCM/RNF220/BCOR/BARD1/FANCB/FANCL/TRIM21/BRCA2/RNF40/RAD18/FANCC/TRIM41/FANCG/UBE2O/RNF126/FANCE/UBE2T/TRIM25/ZNF598/DDB2/RAD51/DTL/FANCA/UHRF1

GOBP_REGULATION_OF_NEUROGENESIS	GOBP_REGULATION_OF_NEUROGENESIS	0.000485207	SYT4/NKX2-2/SOX10/RELN/EPHA7/ASCL1/F2/SLIT2/DLL3/CHODL/NOG/MAP1B/MAP6/NGF/PAK3/PTN/FGF2/PRTG/DAB1/PLXNA4/DAAM2/DIXDC1/PTPRD/CXCL12/GLI3/SEMA3E/IL33/NTN1/MAPT/RND2/NFATC4/LPAR3/SERPINF1/NKX6-1/CX3CR1/SMARCD3/FGF13/ASPA/SERPINE2/L1CAM/HEYL/IL6/SHH/SMO/FN1/SPART/EFNB3/CRABP2/DPYSL5/ID4/ATOH1/SEMA6A/CCL11/SEMA6D/NEURL1/NEFL/TENM4/ISLR2/PTPRZ1/THY1/MIR222/SEMA6C/SEMA3G/VEGFC/MAP2/SEMA5B/SEMA3D/ROBO1/CXCR4/GPER1/IL6ST/IL34/LEF1/KIT

GOBP_DEVELOPMENTAL_CELL_GROWTH	GOBP_DEVELOPMENTAL_CELL_GROWTH	0.000488271	SYT4/EPHA7/AKAP6/SLIT2/IGF1/PI16/SLIT3/MAP1B/RGS4/NGF/TNN/PLXNA4/NDN/CXCL12/SEMA3E/NTN1/AGT/MAPT/RND2/LPAR3/NKX6-1/EDNRA/FGF13/RGS2/L1CAM/NRP2/PRKN/SH3GL2/FLRT1/FN1/SPART/NLGN3/CRABP2/SORBS2/SEMA6A/TMEM108/SEMA6D/ISLR2/NRCAM/WASF1/SEMA6C/SYT1/SEMA3G/DCLK1/MAP2/SEMA5B/SEMA3D/CXCR4/HAMP/SEMA3C/NRP1

GOBP_CARDIAC_CHAMBER_DEVELOPMENT	GOBP_CARDIAC_CHAMBER_DEVELOPMENT	0.000491497	HAND1/HAND2/MYOCD/NKX2-5/ANK2/CPE/SLIT2/MYL3/SFRP2/NOG/KCNK2/SLIT3/ZFPM2/GREB1L/TPM1/FOXF1/ADAMTS1/SALL4/EDNRA/SMARCD3/PDE2A/CCN1/TGFB2/RBP4/NRP2/HEYL/SMO/ISL1/PARVA/FOXC2/HEG1/RYR2/TGFBR3/TEK/MEF2C/TBX5/ROBO1/ACVR1/SEMA3C/NRP1/APLNR/BMP5/FGFR2/FZD1/SOX11

GOCC_SPINDLE_MICROTUBULE	GOCC_SPINDLE_MICROTUBULE	0.00049287	CHMP6/CCDC57/EML3/TUBG1/CALM3/SKA2/CHMP2B/PARP4/HAUS2/CEP295/RAB11A/HAUS4/CHMP7/KIF2A/CHMP1A/POLB/HNRNPU/CHMP5/SKA3/ZW10/HAUS6/HAUS5/KNTC1/PSRC1/PRC1/RMDN3/MAP1S/FAM110A/CCSAP/HAUS8/ZWILCH/AURKB/AURKA/CDK1/KIF18A/KIF18B/KIF4A/CHMP4C/SKA1/CENPE/MISP/PLK1/BIRC5/KIF11
GOBP_REGULATION_OF_FAT_CELL_DIFFERENTIATION	GOBP_REGULATION_OF_FAT_CELL_DIFFERENTIATION	0.00050053	MIR483/SFRP1/ADIPOQ/TPH1/HTR2A/LMO3/SFRP2/FERMT2/FNDC5/MEDAG/ZFPM2/RUNX1T1/ZBTB16/FRZB/TGFB1I1/LGALS12/WWTR1/IL6/MIR27B/FFAR4/WNT10B/ID4/SNAI2/CCN4/DKKL1/LPL/RARRES2

GOBP_SOMATIC_DIVERSIFICATION_OF_IMMUNE_RECEPTORS	GOBP_SOMATIC_DIVERSIFICATION_OF_IMMUNE_RECEPTORS	0.000503436	MSH3/RNF168/SHLD2/SHLD3/TP53BP1/MAD2L2/RIF1/BATF/ADAR/PMS2/CYREN/NHEJ1/ATAD5/TBX21/POLL/MSH6/POLB/BCL11B/TFRC/PAXIP1/MCM3AP/TCF7/HMCES/DCAF1/PRKDC/HMGB2/UNG/KMT5C/IL27RA/NBN/MSH2/HSPD1/PARP3/POLQ/EXO1/NSD2/TCF3/LIG1/TNFSF13/IL10
GOBP_POSITIVE_REGULATION_OF_MAP_KINASE_ACTIVITY	GOBP_POSITIVE_REGULATION_OF_MAP_KINASE_ACTIVITY	0.000502703	PLA2G1B/ELANE/HTR2A/GHR/EPGN/HTR2B/ROR2/FGF2/THBS1/CCL19/FZD10/FGFR1/TENM1/DKK1/NTF3/LRRK2/PDGFD/FGF1/NOX4/PDE5A/MEF2C/S100A12/PDGFC/ROBO1/FLT3/IL34/KIT/PDGFRB/DIRAS1/FZD4/MAP3K12

GOBP_SOMATIC_DIVERSIFICATION_OF_IMMUNOGLOBULINS	GOBP_SOMATIC_DIVERSIFICATION_OF_IMMUNOGLOBULINS	0.000513627	MSH3/RNF168/SHLD2/SHLD3/TP53BP1/MAD2L2/RIF1/BATF/PMS2/CYREN/NHEJ1/ATAD5/TBX21/POLL/MSH6/POLB/TFRC/PAXIP1/MCM3AP/HMCES/PRKDC/UNG/KMT5C/IL27RA/NBN/MSH2/HSPD1/PARP3/POLQ/EXO1/NSD2/TCF3/TNFSF13/IL10
GOBP_REGULATION_OF_STRIATED_MUSCLE_CELL_DIFFERENTIATION	GOBP_REGULATION_OF_STRIATED_MUSCLE_CELL_DIFFERENTIATION	0.000517414	SMYD1/MYOCD/NKX2-5/AKAP6/IGF1/RBM24/PI16/RGS4/PLPP7/DMPK/RGS2/LMOD3/TMEM119/HOPX/SHH/DKK1/FZD7/CCN3/MEF2C/MIR222/MAMSTR
GOBP_POSITIVE_REGULATION_OF_PEPTIDE_SECRETION	GOBP_POSITIVE_REGULATION_OF_PEPTIDE_SECRETION	0.000530514	GCG/UCN3/GHRH/NNAT/FGA/ADCYAP1/SCT/FGG/GRP/GIP/RFX6/NKX6-1/S100A8/RBP4/NR1H4/ISL1/FFAR4/GPR27/FGB/NLGN2/BLK/GABBR1/GPLD1/MYRIP

GOBP_HOMOLOGOUS_RECOMBINATION	GOBP_HOMOLOGOUS_RECOMBINATION	0.000541618	HDAC10/TRIP13/BRCA1/PSMC3IP/TOPBP1/FANCM/SLX4/RAD54B/BARD1/EME1/MUS81/RAD21/RBBP8/NBN/RMI1/RAD51AP1/DMC1/EME2/CENPX/RAD51/FANCD2/TOP2A/RAD54L/BRIP1/REC8/TEX19
GOCC_ER_TO_GOLGI_TRANSPORT_VESICLE_MEMBRANE	GOCC_ER_TO_GOLGI_TRANSPORT_VESICLE_MEMBRANE	0.0005402	B2M/LMAN1/VMA21/SAR1B/KLHL12/SEC23IP/CNIH1/SCAP/TMED2/USO1/SEC22B/PDCD6/STX5/SEC24D/HLA-DPB1/SEC24A/TMED7/SLC30A5/SEC31B/VTI1A/HLA-H/HLA-DPA1/FOLR1/TGFA/HLA-E/SREBF2/HLA-DRA/HLA-B/HLA-A/HLA-DQA2/SEC13/HLA-DQA1/SEC24C/SEC23B/HLA-DRB5/SEC16A/CD74/HLA-F/HLA-C/HLA-DRB1/HLA-DQB1/HLA-DQB2/SREBF1

GOBP_REGULATION_OF_SMOOTHENED_SIGNALING_PATHWAY	GOBP_REGULATION_OF_SMOOTHENED_SIGNALING_PATHWAY	0.000540815	SFRP1/FGF10/PTCH2/GPC3/GAS1/GLI3/GLI1/HHIP/EVC/SERPINE2/SHH/SMO/DHH/PRRX1/GPR161/RAB34/SCUBE1/PTCH1/GLIS2/DYNC2H1/PRKACB/FGFR2/RORA/CIBAR1/FUZ/DCDC2/ARMC9
GOBP_SENSORY_SYSTEM_DEVELOPMENT	GOBP_SENSORY_SYSTEM_DEVELOPMENT	0.000540113	MAB21L2/KERA/NEUROD1/CHRDL1/ANGPTL7/FGF10/GPM6A/FAT3/ALDH1A2/CRYAB/TMOD1/SALL2/ADAMTS18/EFEMP1/CYP1B1/CACNA1C/FGF2/TUB/CDON/WT1/GLI3/ZEB1/MFAP5/COL8A1/SLC17A7/SDK2/WNT9A/VSTM4/PRPH2/ATP2B4/TENM3/LAMA1/SERPINF1/NDP/SMARCD3/CRB2/TGFB2/MEIS1/RHOJ/HPCA/FBN1/RBP4/SHH/CLIC4/PDGFRA/PBX1/TMEM132E/SLITRK6/FOXF2/COL8A2/PLAAT1/PBX3/ISL1/WNT2B/THRB/FOXC2/VIM/CC2D2A/CELF4/MAF/BMPR1B/MEIS3P1/THY1/INHBA/ALDH1A3/CRYBA2/PTPRM/MFAP2/TWIST1/CYP1A1/MEIS3/GNGT1/SH3PXD2B/PDGFRB/ARHGEF15/COL5A1/NRP1/FZD4/COL5A2/MERTK/SPRED3/CRYBB1/P2RY12/PDE6B/C3/SOX11/BMP6/PITX2/NPHP1/PXDN/MITF/SDK1/OBSL1/FJX1/N[illegible]

GOBP_NEPHRON_DEVELOPMENT	GOBP_NEPHRON_DEVELOPMENT	0.000548307	ADIPOQ/ACTA2/TMEM59L/SIX2/NOG/ADAMTS16/GPC3/FGF2/GREM1/WT1/GLI3/GREB1L/AGT/EDNRA/ANGPT1/WWTR1/HEYL/PKD2/KLF15/SHH/SMO/PDGFRA/OSR1/PBX1/WNT2B/TCF21/PDGFD/FOXC2/DCHS1/FGF1/ENPEP/ILK/NID1/TEK/MTSS1/MEF2C/COL4A4/PTCH1/IRX3/ITGB3/SULF1/CD34/PDGFRB/PECAM1/EDNRB
GOBP_CARDIAC_VENTRICLE_MORPHOGENESIS	GOBP_CARDIAC_VENTRICLE_MORPHOGENESIS	0.000554176	HAND1/HAND2/NKX2-5/CPE/MYL3/SFRP2/NOG/ZFPM2/TPM1/FOXF1/EDNRA/SMARCD3/TGFB2/HEYL/ISL1/FOXC2/HEG1/RYR2/TGFBR3/MEF2C/TBX5

GOBP_CARDIAC_VENTRICLE_DEVELOPMENT	GOBP_CARDIAC_VENTRICLE_DEVELOPMENT	0.00055428	HAND1/HAND2/MYOCD/NKX2-5/CPE/SLIT2/MYL3/SFRP2/NOG/KCNK2/SLIT3/ZFPM2/GREB1L/TPM1/FOXF1/SALL4/EDNRA/SMARCD3/PDE2A/CCN1/TGFB2/HEYL/ISL1/FOXC2/HEG1/RYR2/TGFBR3/MEF2C/TBX5/ROBO1/ACVR1/SEMA3C/APLNR/FGFR2/FZD1/SOX11/GJA5/ENG
GOMF_EXTRACELLULAR_MATRIX_STRUCTURAL_CONSTITUENT_CONFERRING_COMPRESSION_RESISTANCE	GOMF_EXTRACELLULAR_MATRIX_STRUCTURAL_CONSTITUENT_CONFERRING_COMPRESSION_RESISTANCE	0.000558617	OGN/PRG4/PRELP/PODN/ASPN/DCN/LUM/VCAN/FMOD/BGN

GOCC_RNA_POLYMERASE_II_HOLOENZYME	GOCC_RNA_POLYMERASE_II_HOLOENZYME	0.000564599	GTF2H1/POLR2F/TAF6/ENY2/RPRD2/RTF1/ATXN7/POLR2G/TBPL1/CCNH/TAF1/GTF2E1/TBP/MNAT1/RPRD1B/TAF13/TAF4/GTF2B/RPRD1A/CDC73/GTF2A1/ERCC3/TAF9/TAF12/TAF2/TADA3/RPAP2/TRRAP/TAF5L/RECQL5/POLR2C/USP22/ERCC2/GTF2H2/GTF2H2C/TCEA1/CDK7/POLR2H/CTR9/LEO1/POLR2L/MYO6/POLR2D/MMS19/TAF6L/ATXN7L3/PEX2/TAF4B/POLR2E/POLR2B/GTF2H3/GTF2F1/KAT2A/POLR2A/TAF5/GTF2E2/GTF2A2

GOBP_BONE_DEVELOPMENT	GOBP_BONE_DEVELOPMENT	0.00057006	OGN/CER1/COL2A1/MYOC/PAPPA2/RGN/IGF1/GHR/BNC2/FLNA/DDR2/TNN/ZBTB16/GREM1/SFRP4/FREM1/LRRC17/GLI3/THPO/LEPR/RAB23/TGFB3/EVC/MEIS1/FBN1/TMEM119/COMP/COL3A1/MMP16/EPYC/GJA1/DCHS1/CCN4/LOX/HAS2/BMPR1B/STC1/MEF2C/LTBP3/NPR2/PDGFC/ANXA6/TWIST1/BGN/MPIG6B/SH3PXD2B/SULF1/KIT/COL1A1/NPPC/FOXN3/FGFR2/FAM20C/BMP6/PITX2/ENG/GHRL/THBS3/HOXB4/PAPSS2

GOBP_RHYTHMIC_PROCESS	GOBP_RHYTHMIC_PROCESS	0.000580551	AFP/CARTPT/GHRH/ADIPOQ/TPH1/NGFR/SLIT2/PGR/PTPRN/FBXL22/HLF/SLIT3/CYP1B1/PTN/C3orf70/PRKAA2/MAPK10/NLGN1/ENOX1/ADCY1/F7/GDF10/PTX3/TGFB3/ADAMTS1/SERPINF1/USP2/TGFB2/KLF9/MMP2/ESR1/TIMP4/MMP19/PDGFRA/CGA/A2M/GDF9/RETN/OXTR/ID4/PTGDS/HAS2/BMPR1B/MTTP/PASD1/KCND2/EGR3/PROK2/INHBA/TWIST1/MAGEL2/CHRNB2

GOBP_POSITIVE_REGULATION_OF_VASCULATURE_DEVELOPMENT	GOBP_POSITIVE_REGULATION_OF_VASCULATURE_DEVELOPMENT	0.00058633	HSPB6/CMA1/PTGIS/SFRP2/BMPER/CYP1B1/SMOC2/FGF2/TNN/GREM1/PRKD1/AKT3/THBS1/CAMP/CX3CR1/HGF/NODAL/ITGA5/PRKCB/SERPINE1/CYSLTR1/EMILIN1/CCBE1/MIR27B/ISL1/FOXC2/CCL11/FGF1/RHOB/RAMP2/CXCR2/HSPB1/TEK/VASH2/TNFSF12/TWIST1/ITGB3/VEGFC/CXCR4/CD34/GAB1/NRP1/APLNR/RRAS/TIE1/C3/ANGPT2/TBXA2R/ENG/GHRL/CDH5/C5/DLL1/ANGPTL4/SEMA5A/ADAM12/ADM/VEGFB/C3AR1/C5AR1
GOBP_POSITIVE_REGULATION_OF_PROTEIN_SECRETION	GOBP_POSITIVE_REGULATION_OF_PROTEIN_SECRETION	0.000594875	PLA2G1B/GCG/UCN3/NNAT/FGA/PCSK1/ANKRD1/IGF1/FGG/MYOM1/GIP/TGFB3/APBB1/RFX6/NKX6-1/TGFB2/RBP4/MYH10/NR1H4/ISL1/GPR27/FGB/NLGN2/BLK/GPLD1/MYRIP/WLS/VEGFC

GOBP_OXIDATIVE_PHOSPHORYLATION	GOBP_OXIDATIVE_PHOSPHORYLATION	0.000599002	NDUFB5/DNAJC15/NDUFC1/NDUFA1/COX7B/CHCHD2/NDUFB4/UQCRB/ATP5MG/COX6B1/RHOA/NDUFS8/UQCR11/NDUFB2/COA6/ATP5MF/DGUOK/COX7C/NDUFA8/COX7A2L/ATP5F1C/NDUFA6/NDUFB10/ATP5PD/C2orf69/DLD/FXN/NDUFS5/COX7A2P2/SLC25A23/NDUFB3/NDUFA12/NDUFA5/UQCRQ/ABCD1/PARK7/BID/NDUFA9/NDUFB6/NDUFA11/NDUFAF1/NDUFS6/UQCRFS1/AFG1L/NDUFA10/NDUFS2/ATP5F1EP2/NDUFA2/NDUFS1/COX7A2/NDUFS3/SDHD/NDUFB7/COX5B/ATP5PO/NDUFAB1/UQCR10/COX4I1/ATP5PB/COX6A1/NDUFA13/SLC25A33/UQCC3/PDE12/CYCS/SDHB/COQ9/COX15/COX5A/UQCRC2/ATP5F1A/UQCRFS1P1/UQCRH/GHITM/VCP/COX8A/ANTKMT/SDHA/ATP5F1B/MSH2/STOML2/ATP5ME/NDUFV1/CDK1/PPIF/NDUFS7/[illegible]

GOBP_CARDIAC_CHAMBER_MORPHOGENESIS	GOBP_CARDIAC_CHAMBER_MORPHOGENESIS	0.000602246	HAND1/HAND2/NKX2-5/CPE/SLIT2/MYL3/SFRP2/NOG/SLIT3/ZFPM2/TPM1/FOXF1/ADAMTS1/EDNRA/SMARCD3/CCN1/TGFB2/RBP4/NRP2/HEYL/SMO/ISL1/PARVA/FOXC2/HEG1/RYR2/TGFBR3/TEK/MEF2C/TBX5/ROBO1/ACVR1/SEMA3C/NRP1/APLNR/BMP5/FGFR2/FZD1/SOX11
GOBP_REGULATION_OF_ATTACHMENT_OF_SPINDLE_MICROTUBULES_TO_KINETOCHORE	GOBP_REGULATION_OF_ATTACHMENT_OF_SPINDLE_MICROTUBULES_TO_KINETOCHORE	0.000605493	HNRNPU/NEK2/KNSTRN/AURKB/SPAG5/INCENP/RACGAP1/RCC2/CCNB1/ECT2/BIRC5/CDCA8
GOBP_GLUCOSE_IMPORT	GOBP_GLUCOSE_IMPORT	0.000608692	PLA2G1B/ADIPOQ/SLC2A4/TRARG1/SORBS1/IGF1/GPC3/SLC2A2/PID1/ITLN1/KLF15

GOBP_KERATINOCYTE_DIFFERENTIATION	GOBP_KERATINOCYTE_DIFFERENTIATION	0.000611393	CYP27B1/CYP26B1/TRIM16/MED1/IRF6/CASP3/SPRR2D/EVPL/JAG1/EZH2/S100A7/CDH3/MSX2/ADAM9/DNASE1L2/GRHL2/KRT17/ST14/KRT80/KRT6B/VDR/SFN/PLAAT4/KDF1/FLNB/FOXC1/KLK5/OVOL2/KRT7/ZBED2/EXPH5/EPHA2/KRT8/ABCA12/KRT16/SPRR2E/KRT81/LCE3E/LCE3D/SPRR2B/SPRR2G

GOBP_RESPONSE_TO_CARBOHYDRATE	GOBP_RESPONSE_TO_CARBOHYDRATE	0.000615905	GCG/APOA2/UCN3/NKX2-2/ADIPOQ/NEUROD1/KCNB1/PCSK1/CMA1/ADCY5/APOB/PTPRN/GRIK5/MAP1B/P2RX2/PRKAA2/COLEC12/MIR320C2/NPTX1/GIP/THBS1/RFX6/SERPINF1/NKX6-1/SLC8A1/PRKCB/ZBTB20/NR1H4/COL6A2/CCN2/MIR320D1/GPR27/LPL/KLF7/NOX4
GOBP_PROTEIN_LOCALIZATION_TO_CONDENSED_CHROMOSOME	GOBP_PROTEIN_LOCALIZATION_TO_CONDENSED_CHROMOSOME	0.00062243	MTBP/ZW10/TTK/KNTC1/ZWILCH/AURKB/NDC80/BUB3/CDK1/RCC2/KNL1/CDT1

GOCC_MITOTIC_SPINDLE	GOCC_MITOTIC_SPINDLE	0.000625892	HAUS3/ZZZ3/AAAS/NUDCD2/BCCIP/SMC3/WAPL/CCDC117/EML3/GOLGA2/RPS3/HDAC3/TUBG1/KAT5/TAF1D/STAG1/NUP62/CNTRL/HAUS2/CEP295/HAUS4/TPX2/CKAP2/LIMK2/HSF1/GPSM2/HNRNPU/IQCB1/PYCR3/SKA3/HAUS6/EPB41/EML2/WDR5/KAT2A/HAUS5/KNSTRN/SMC1A/PRC1/RMDN3/GIT1/RANGAP1/MAP1S/CCSAP/HAUS8/AURKB/KIF20B/CKAP2L/CDC14B/ASPM/SPAG5/KIF23/PTPN7/WRAP73/AURKA/TNKS/NUDC/CTTN/KIFC1/CDK1/WDR62/KIF18A/DIAPH1/KIF18B/TUBB4B/RACGAP1/TPPP/RCC2/KIF22/KIFC2/ESPL1/ECT2/HNF4G/TACC3/SKA1/MAD2L1/CENPE/CDC7/MISP/PLK1/KIF11/ODAM
GOBP_BLOOD_VESSEL_ENDOTHELIAL_CELL_PROLIFERATION_INVOLVED_IN_SPROUTING_ANGIOGENESIS	GOBP_BLOOD_VESSEL_ENDOTHELIAL_CELL_PROLIFERATION_INVOLVED_IN_SPROUTING_ANGIOGENESIS	0.000631683	AGTR1/MIR483/NGFR/BMPER/FGF2/THBS1/MIR342/MIR27B/MIR149/MIR222/MIR10B/MMRN2
GOMF_CYCLIC_NUCLEOTIDE_PHOSPHODIESTERASE_ACTIVITY	GOMF_CYCLIC_NUCLEOTIDE_PHOSPHODIESTERASE_ACTIVITY	0.000640061	PDE7B/PDE1A/PDE9A/PDE3A/PDE2A/PDE1C/PDE1B/PDE8B/PDE5A/PDE3B/PDE6B/PDE4B

GOBP_MESONEPHRIC_TUBULE_MORPHOGENESIS	GOBP_MESONEPHRIC_TUBULE_MORPHOGENESIS	0.000638491	TMEM59L/SIX2/NOG/ADAMTS16/GPC3/FGF2/GREM1/WT1/GLI3/GREB1L/AGT/PKD2/SHH/SMO/OSR1/PBX1/WNT2B/TCF21/DCHS1/FGF1/ILK
GOCC_CENTRIOLE	GOCC_CENTRIOLE	0.000640312	CEP97/RABL2B/IFT43/KIAA0586/CEP350/TUBGCP3/DCTN1/NEDD1/SCLT1/CCP110/CROCC/MPHOSPH9/CETN3/C2CD3/AKNA/TOPORS/CEP135/CEP104/CPLANE2/HERC2/PCM1/BCCIP/CEP20/CEP128/CCDC57/NEURL4/CFAP20/TUBG1/ALMS1/ODF2/MKS1/CEP83/HAP1/SSNA1/CEP295/RAB11A/KIF2A/CEP192/PPP1R35/NPHP4/NUBP1/IQCB1/CEP152/CCDC120/RTTN/RAN/NUBP2/SASS6/SFI1/CCHCR1/RAB8A/POC1B/CCSAP/PCNT/LRRCC1/HTT/WRAP73/AURKA/CEP78/GLE1/CNTROB/TEDC2/CEP131/WDR62/PLK2/PARP3/PLK4/KIF24/WDR90/DYNC2I2/TEDC1/MAPK15/PLK1/CEP55/TOP2A/POC1A/CCNF/STIL/CCDC68/SAXO2/CCDC78/PLA2G3
GOBP_DNA_STRAND_ELONGATION_INVOLVED_IN_DNA_REPLICATION	GOBP_DNA_STRAND_ELONGATION_INVOLVED_IN_DNA_REPLICATION	0.000643762	RFC3/POLA1/RFC4/POLE3/GINS1/POLD3/MCM7/POLE/POLD2/PCNA/MCM3/LIG1/DNA2/MCM4

GOBP_EXPLORATION_BEHAVIOR	GOBP_EXPLORATION_BEHAVIOR	0.000648687	PENK/ATP1A2/NOG/GIP/CHL1/LRRK2/DLG4/NLGN2/JPH3
GOBP_POSITIVE_REGULATION_OF_DNA_RECOMBINATION	GOBP_POSITIVE_REGULATION_OF_DNA_RECOMBINATION	0.00064824	ING3/ARID2/ACTR2/ERCC6/CD40/YEATS4/TRRAP/ERCC2/SHLD2/SHLD3/TP53BP1/WAS/MAD2L2/EP400/RIF1/BRD8/MRGBP/PIAS4/ACTL6A/KAT5/HDGFL2/MMS19/ATAD5/TBX21/FUS/RUVBL1/RUVBL2/TFRC/FANCB/PARP1/PAXIP1/HMCES/KMT5C/RBBP8/MSH2/WRAP53/RAD51AP1/TIMELESS/NSD2/TNFSF13
GOMF_METALLOEXOPEPTIDASE_ACTIVITY	GOMF_METALLOEXOPEPTIDASE_ACTIVITY	0.000649405	CPA1/CPA2/CPXM2/CPE/CPN1/CPA3/XPNPEP2/NAALAD2/MMP16/CPXM1/TRHDE/DPEP1/CPA4/CPA6/ENPEP/AEBP1/VASH2/CPQ

GOBP_CELL_CYCLE_G2_M_PHASE_TRANSITION	GOBP_CELL_CYCLE_G2_M_PHASE_TRANSITION	0.000647908	CHEK2/KHDRBS1/RAB11A/BRCC3/BRCA1/VPS4B/TOPBP1/AKAP8/MIIP/BARD1/PPM1D/PAXIP1/DONSON/FBXL15/ZFYVE19/SKP2/PINX1/RAD21/CCNG1/TAOK2/RINT1/RRM2B/FBXO5/FZR1/AURKB/NDC80/FBXL8/USH1C/RBBP8/CDC14B/NBN/AURKA/STOX1/KIF14/CDC25C/NPM1/CDK1/FOXM1/CIT/CCNB2/CENPF/RRM1/CHEK1/MELK/BLM/TP53/TICRR/CLSPN/RCC2/DNM2/CHMP4C/CCNB1/FBXL6/DTL/CDC7/PLK1/CCNA2/CCND1/CDC25A/ATF5/PKMYT1/ORC1

GOBP_NEUROMUSCULAR_PROCESS	GOBP_NEUROMUSCULAR_PROCESS	0.00064698	KCNA1/PENK/SYNM/CASQ1/ASCL1/ADCY5/GRIN2A/DMD/GLRB/MAP1A/HOXD10/CNTNAP1/DMPK/EDNRA/STAC/COMP/PRKN/CDH23/TMEM150C/SLITRK6/SPART/UCHL1/SLURP1/PDE8B/FOXS1/DLG4/NEFL/TCF15/USH1G/ITPR1/NLGN2/ALDH1A3/JPH3/FGF12

GOBP_ELECTRON_TRANSPORT_CHAIN	GOBP_ELECTRON_TRANSPORT_CHAIN	0.000645604	NDUFB8/NDUFC2/ETFB/PUM2/DHRS3/NDUFB5/DNAJC15/NDUFC1/NDUFA1/FDX1/COX7B/CHCHD2/NDUFB4/UQCRB/COX6B1/NDUFS8/UQCR11/NDUFB2/COA6/DGUOK/ETFDH/COX7C/ETFA/NDUFA8/ETFRF1/P4HA2/COX7A2L/CYB5A/NDUFA6/NDUFB10/ALDH2/DLD/NDUFS5/COX7A2P2/NDUFB3/THAP11/NDUFA12/NDUFA5/PDIA5/UQCRQ/GPD1/PARK7/BID/NDUFA9/NDUFB6/NDUFAF1/NDUFS6/UQCRFS1/AFG1L/NDUFA10/NCF1/NDUFS2/QDPR/NDUFA2/NDUFS1/COX7A2/NDUFS3/SDHD/GPD2/SRD5A1/NDUFB7/IDH3B/COX5B/NQO2/NDUFAB1/UQCR10/ME2/COX4I1/COX6A1/PLEC/UQCC3/CYCS/CIAPIN1/AKR7A2/SDHB/NDOR1/RDH16/COQ9/COX15/COX5A/UQCRC2/UQCRFS1P1/UQCRH/GHITM/COX8A/SDHA/ASPH/NDUFV1/GPX2/CYBA/MYBBP1[illegible]

GOBP_IMMUNOGLOBULIN_PRODUCTION_INVOLVED_IN_IMMUNOGLOBULIN_MEDIATED_IMMUNE_RESPONSE	GOBP_IMMUNOGLOBULIN_PRODUCTION_INVOLVED_IN_IMMUNOGLOBULIN_MEDIATED_IMMUNE_RESPONSE	0.000652689	RNF168/SHLD2/SHLD3/TP53BP1/MAD2L2/RIF1/BATF/HLA-DPB1/ATAD5/TBX21/MSH6/HLA-DPA1/TFRC/PAXIP1/HLA-DMB/HLA-DOB/HLA-DRA/HMCES/TRAF3IP2/HLA-DQA2/UNG/KMT5C/HLA-DQA1/IL27RA/NBN/MSH2/HSPD1/HLA-DRB5/HLA-DRB1/HLA-DOA/PARP3/EXO1/NSD2/HLA-DQB1/HLA-DQB2/HLA-DMA/TNFSF13/IL10
GOMF_EXORIBONUCLEASE_ACTIVITY_PRODUCING_5_PHOSPHOMONOESTERS	GOMF_EXORIBONUCLEASE_ACTIVITY_PRODUCING_5_PHOSPHOMONOESTERS	0.000659087	DIS3L2/CNOT8/USB1/PARN/CNOT6L/CPSF3/DIS3L/DIS3/PAN2/ISG20L2/XRN1/CNOT7/EXOSC10/EXOSC2/EXOSC7/EXOSC5/PDE12/CNOT6/EXOSC9/EXOSC4/CNOT1/TOE1/ERI2/ISG20/POLRMT/ERI1

GOMF_VOLTAGE_GATED_POTASSIUM_CHANNEL_ACTIVITY	GOMF_VOLTAGE_GATED_POTASSIUM_CHANNEL_ACTIVITY	0.00066178	KCNA1/KCNB1/KCNMA1/KCNK3/KCNA5/ABCC9/KCNK2/KCNH6/KCNT2/KCNJ8/KCNE4/KCNH2/PKD2/KCNG1/SNAP25/KCNQ3/KCND1/KCNS1/KCND2
GOBP_TRNA_METHYLATION	GOBP_TRNA_METHYLATION	0.000671737	TRMT61A/TRMT10C/THADA/TRMT2B/TRMT5/NSUN6/NSUN3/TRMT112/TARBP1/TRMT6/ALKBH8/TYW3/MTO1/THUMPD3/THUMPD2/TRMO/TRMT61B/HSD17B10/FTSJ1/DALRD3/TRMT11/METTL2A/METTL2B/TRMT10A/TRMT2A/NSUN2/WDR6/WDR4/TRMT44/TRMT1/GTPBP3

GOBP_POSITIVE_REGULATION_OF_WNT_SIGNALING_PATHWAY	GOBP_POSITIVE_REGULATION_OF_WNT_SIGNALING_PATHWAY	0.000686615	SFRP1/FGF10/MIR145/RSPO1/SFRP2/GPC3/RSPO3/FGF2/SFRP4/DAAM2/DIXDC1/RECK/RSPO2/CAV1/DACT1/SHH/DAB2/ANKRD6/DKK1/LRRK2/WNT10B/ZEB2/CCN4/ADGRA2/ILK/DKK2/EDA/WLS/RSPO4/SULF1/LEF1/COL1A1
GOBP_FOREBRAIN_DEVELOPMENT	GOBP_FOREBRAIN_DEVELOPMENT	0.000689222	KCNA1/GHRH/RELN/ATP1A2/NEUROD1/FGF10/PCSK1/ASCL1/FOXP2/ALDH1A2/SCT/SLIT2/PTCHD1/NOG/CDH2/SLIT3/NPY/ATP1B2/CCKAR/FGF2/IGF2BP1/DCLK2/DAB1/PLXNA4/DIXDC1/CDON/CXCL12/GLI3/ZEB1/SEMA3E/PRKG1/ATP2B4/GLI1/FAT4/FGF13/LPAR1/NRP2/FEZ1/SLC8A1/COL3A1/SHH/AVPR1A/SMO/DKK1/ISL1/DLC1/GDF7/WNT2B/OXTR/LRRK2/ID4/ATOH1/SSTR2/POU3F1/BMERB1/TMEM108/LHX6/NEFL/AVPR2/INHBA/ALDH1A3/TACC1/PHACTR1/DYNC2H1/DCLK1/CHRNB2/ROBO1/CXCR4/CHD5/LEF1/GLI2/NDNF/AXL/NRP1

GOCC_HETEROCHROMATIN	GOCC_HETEROCHROMATIN	0.000695247	HSF1/PML/LRWD1/TCP1/ZBTB18/UBA1/EME1/RRP1B/KDM4C/TRIM28/SUV39H1/KMT5C/SIRT6/HDAC1/KDM4A/CBX2/CENPA/EZH2/H2AZ1/CBX8/INCENP/HMGA1/DNMT1/NCAPD3/HELLS/FOXC1/ESCO2/WDR76/H1-4/UHRF1/H1-5
GOBP_REGULATION_OF_CELL_CYCLE_G2_M_PHASE_TRANSITION	GOBP_REGULATION_OF_CELL_CYCLE_G2_M_PHASE_TRANSITION	0.00070065	RAB11A/BRCC3/BRCA1/VPS4B/TOPBP1/MIIP/BARD1/PAXIP1/DONSON/ZFYVE19/PINX1/RAD21/CCNG1/TAOK2/RINT1/RRM2B/FBXO5/FZR1/AURKB/RBBP8/CDC14B/NBN/AURKA/STOX1/KIF14/CDC25C/NPM1/CDK1/CENPF/RRM1/CHEK1/BLM/TP53/TICRR/CLSPN/RCC2/CHMP4C/CCNB1/DTL/CDC7/PLK1/CCND1/CDC25A/ATF5/ORC1

GOBP_TYPE_I_INTERFERON_PRODUCTION	GOBP_TYPE_I_INTERFERON_PRODUCTION	0.000704164	IRF7/OAS1/SETD2/TOMM70/ATG5/DHX33/GPATCH3/HSP90AA1/OAS2/RNF26/POLA1/DHX58/DHX9/POLR3B/PLCG2/IRF3/POLR3A/PYCARD/CACTIN/MYD88/G3BP1/PTPN22/NMI/SYK/TRIM21/RIPK2/TLR3/CHUK/IRF8/HMGB2/TRAIP/OAS3/DTX4/MMP12/TICAM1/IRAK1/HSPD1/STAT1/CGAS/GAPDH/POLR3G/TRIM15/IRF1/IL10

GOMF_ENDOPEPTIDASE_ACTIVITY	GOMF_ENDOPEPTIDASE_ACTIVITY	0.000704171	CTRB1/ELANE/CTRB2/PRTN3/AZU1/CELA2A/HP/SFRP1/PRSS1/CTSG/PCSK1/CMA1/ADAM33/F2/PAPPA2/ADAMTS8/ADAMTS18/ADAMTS16/CLC/ADAMTSL3/ADAM23/PCSK2/MMP8/F7/MASP1/PRSS2/PIP/CTSV/ADAMTS1/CAPN11/TLL1/USP2/MMP3/HGF/TRABD2B/CLCA1/ADAMTS3/MMP24/MMP2/CAPN6/ADAMTSL1/MMP7/CORIN/MMP19/SHH/PRSS23/CTSF/MMP16/C1S/CTSK/UCHL1/ADAMTS10/MMP23B/CELA3B/PAPLN/C1R
GOBP_ARTERY_DEVELOPMENT	GOBP_ARTERY_DEVELOPMENT	0.000710272	HAND2/MYLK/NGFR/MYOCD/MIR145/APOB/NOG/GLI3/FKBP10/PRICKLE1/FOXF1/AKT3/EDNRA/PDE2A/TGFB2/PKD2/COMP/COL3A1/SHH/PRRX1/EFEMP2/FOXC2/LOX/ADAMTS9/ROBO1/PDGFRB/LRP1/NRP1/APLNR

GOCC_CELL_CELL_JUNCTION	GOCC_CELL_CELL_JUNCTION	0.00071969	SYNM/ATP1A2/DES/LDB3/PGM5/DSG4/CDH19/ADCYAP1R1/NGFR/CTNNA3/SGCA/PCDH9/ANK2/CADM3/SORBS1/BVES/AKAP6/KCNA5/PDLIM3/FLRT2/NEXN/NFASC/CLMP/FERMT2/IGSF11/LIMS2/CDH2/SLC2A2/FLNA/CLDN6/TRPC4/MPDZ/HMCN1/JAM2/CLDN11/JAM3/SCN4B/PRKD1/CNTNAP1/TMEM47/DLG2/DSC3/SV2A/GJC1/LAMA1/CGNL1/LIN7A/CRB2/FGF13/PDLIM4/SLC8A1/ITGA5/PKD2/AMOTL1/PCDHGA12/FRMD6/CDH11/FLRT1/CLIC4/CCN3/MPP2/KIRREL1/OXTR/CLDN9/GJA1/DCHS1/SYNPO/PDLIM7/CTNND2/EPB41L3/DLG4/CLDN19/HEG1/MXRA8/CDH6/CLDN5/TEK/ACTN1/PMP22/ABCC2/PTPRM/ITGB3/CD99L2/ADGRB1/MPP1/PKP1/MYADM/VANGL2/KIT/GAB1/MPP7/PLPP3/SHROOM4/ADGRL3/WTIP/PECAM1/RASIP1/H[illegible]

GOBP_MYOTUBE_DIFFERENTIATION	GOBP_MYOTUBE_DIFFERENTIATION	0.000721697	SMYD1/MYOCD/NKX2-5/ERVW-1/IGF1/KLHL41/DNER/RBM24/ADGRB3/P2RX2/JAM2/CACNA1H/CDON/ACTA1/CNTNAP1/PLPP7/MYMX/DMPK/LMOD3/TMEM119/MYEF2/SHH/AVPR1A/SMO/CCN3/WNT10B/PLEKHO1/EHD2/MEF2C/MAMSTR/RIPOR2/ADGRB1/ADAMTS5
GOCC_NEUROMUSCULAR_JUNCTION	GOCC_NEUROMUSCULAR_JUNCTION	0.000728909	DES/SYNGR4/EPHA7/SYNC/PDZRN3/NLGN1/LAMA2/TUBA1A/DLG2/SV2A/COL4A5/SERPINE2/SPOCK1/DLG4/NEFL/COLQ/SYP/MUSK/SYNGR1/DLGAP3/SNTA1/STX1B

GOBP_ACTIN_FILAMENT_ORGANIZATION	GOBP_ACTIN_FILAMENT_ORGANIZATION	0.00072813	PLA2G1B/APOA1/SYNPO2/SFRP1/ACTN2/MYOC/LMOD1/TACR1/CTNNA3/SORBS1/TPM2/SLIT2/TMOD1/CFL2/CALD1/CCL26/FERMT2/DPYSL3/RGS4/FLNA/PAK3/SVIL/TF/PHLDB2/HMCN1/DAAM2/ACTA1/CXCL12/ELN/ADD2/SERPINF2/WASF3/CORO6/TPM1/CDC42EP3/ACTC1/CCL21/TGFB3/PPM1E/RND2/KANK4/CSF3/CGNL1/LPAR1/LMOD3/TENM1/WHAMMP3/PRKN/EFS/FAM107A/FHOD3/DLC1/KIRREL1/SORBS2/CCN2/SYNPO/GAS7/CCL11/ARHGAP28/MIR149/S1PR1/RHOB/PLEKHH2/TMSB15A/ACTN1/MTSS1/WASF1/CORO2B/PHACTR1/CYRIA/MAGEL2/ELMO1/DSTN/ENAH/CCR7/SPIRE1/MYADM/ARHGEF15/SHROOM4/GSN/NRP1/RDX/ARHGAP40/PFN2
GOBP_GLOMERULUS_DEVELOPMENT	GOBP_GLOMERULUS_DEVELOPMENT	0.000737969	ADIPOQ/ACTA2/NOG/WT1/EDNRA/ANGPT1/WWTR1/HEYL/KLF15/PDGFRA/OSR1/TCF21/PDGFD/FOXC2/ENPEP/NID1/TEK/MTSS1/MEF2C/COL4A4/ITGB3/SULF1/CD34/PDGFRB/PECAM1/EDNRB

GOBP_POSITIVE_REGULATION_OF_PHAGOCYTOSIS	GOBP_POSITIVE_REGULATION_OF_PHAGOCYTOSIS	0.000739187	AZU1/APOA2/AHSG/APOA1/PLA2G5/TUB/PTX3/CD36/COLEC11/C4A/FCN1/C4B/CCL2/RAB31
GOBP_MORPHOGENESIS_OF_AN_EPITHELIUM	GOBP_MORPHOGENESIS_OF_AN_EPITHELIUM	0.000740247	HAND1/SOX10/SFRP1/HAND2/NGFR/NKX2-5/FGF10/EPHA7/TRIM71/RGMA/ALDH1A2/SLIT2/PGR/FGF7/ASB2/TMEM59L/AR/SIX2/SFRP2/PRICKLE2/NOG/FERMT2/ADAMTS16/GPC3/ROR2/RSPO3/FLNA/FGF2/TNC/PHLDB2/FRZB/GREM1/WT1/GLI3/TGFB1I1/SEMA3E/NTN1/GREB1L/AGT/PRICKLE1/FOXF1/RSPO2/NFATC4/LAMA1/SALL4/HHIP/EDNRA/HGF/NODAL/CYP7B1/CCN1/DACT1/TBX18/TGFB2/ITGA5/PKD2/MMP2/ESR1/SHH/CLIC4/SMO/OSR1/DAB2/PBX1/ANKRD6/DKK1/GPC6/FOXF2/FZD7/DLC1/IGFBP5/GDF7/WNT2B/TCF21/ID4/GJA1/BRSK1/SNAI2/FOXC2/DCHS1/CCL11/FGF1/CC2D2A/TCF15/ILK/SOX17/RHOB/CXCR2/PDPN/RYR2/MTSS1/MEF2C/ALDH1A3/PTCH1/NR3C1/TWIST1/IRX3/MMRN2/TBX5/VEGFC/[illegible]

GOBP_REGULATION_OF_LIPID_METABOLIC_PROCESS	GOBP_REGULATION_OF_LIPID_METABOLIC_PROCESS	0.000742481	APOA2/APOA1/APOC3/ADIPOQ/CIDEA/SORBS1/CES1/F2/SCT/APOD/HTR2A/APOB/RGN/THRSP/HTR2B/FGF2/CNR1/C1QTNF2/NR4A3/TNFAIP8L3/GIP/PRKD1/FMO1/CCL21/PDK4/CCL19/LGALS12/CAV1/HCAR1/SERPINA3/CCN1/FABP3/ZBTB20/NR1H4/APOA4/AVPR1A/RUBCNL/PDGFRA/DAB2/DHH/IGFBP7/CGA/ACER1/MALRD1/SNAI2/PDE8B/DKKL1/FGF1/DKK3/FMO2/RARRES2/TEK/NR3C1/TWIST1/GPLD1/ACACB/FLT3/ENPP7/ELOVL5/GPER1/CCR7/KIT/CCDC3/PDGFRB

GOBP_RIBOSOME_ASSEMBLY	GOBP_RIBOSOME_ASSEMBLY	0.000749514	EIF6/RPL23A/MRPS7/FASTKD2/MRPL20/BRIX1/RPS27L/MTERF3/RPS14/RPL6/RPF2/ERAL1/RPS5/MRPL22/MRPS11/XRCC5/RPS28/ABT1/RPS19/RRP7A/MDN1/NIP7/DDX28/RPLP0/DHX37/RPLP0P6/MPV17L2/PRKDC/RPS15/DHX30/RPSA/TRAF7/NLE1/RRS1/NPM1/MRTO4/MRPS2/BOP1/PWP2/C1QBP/PPAN/EFL1
GOBP_LOW_DENSITY_LIPOPROTEIN_PARTICLE_REMODELING	GOBP_LOW_DENSITY_LIPOPROTEIN_PARTICLE_REMODELING	0.00075072	MPO/APOA2/AGTR1/APOB/PLA2G5/AGT

GOMF_OXIDOREDUCTASE_ACTIVITY_ACTING_ON_THE_CH_CH_GROUP_OF_DONORS	GOMF_OXIDOREDUCTASE_ACTIVITY_ACTING_ON_THE_CH_CH_GROUP_OF_DONORS	0.000758755	RSAD1/AKR1C2/TM7SF2/ACAD10/DHODH/SRD5A3/ACAD9/DPYD/MECR/ZADH2/GCDH/DHDH/ACADSB/ACOX1/SDHD/SRD5A1/ACOX3/ACAD8/ACADVL/SDHB/ACAA1/BLVRB/DUS2/COX15/IVD/TECR/DECR1/CPOX/DHCR7/SDHA/DUS1L/RETSAT/CYP2S1/DHCR24/PTGES2/DUS3L/FASN/ACADS

GOBP_MYELOID_LEUKOCYTE_MIGRATION	GOBP_MYELOID_LEUKOCYTE_MIGRATION	0.000757727	PLA2G1B/PRTN3/AZU1/CCL16/TAFA4/THBS4/CTSG/SLIT2/CCL26/ROR2/GREM1/JAM3/CXCL6/CXCL12/CHGA/CCL21/ITGA9/THBS1/GP2/CCL19/S100A8/IL1R1/CX3CR1/TGFB2/ITGA1/MMP2/IL6/S100A9/SERPINE1/EMILIN1/CCL2/FOLR2/CCN3/CD200/PDGFD/CCL11/DUSP1/BST1/CXCR2/CD300H/RARRES2/CAMK1D/SCG2/CXCR1/S100A12/RIPOR2/VEGFC/CD99L2/MPP1/CCR7/IL34/KIT/PECAM1/EDNRB/TNFSF18/EDN2/FPR2/XG/P2RY12
GOBP_MATURATION_OF_5_8S_RRNA	GOBP_MATURATION_OF_5_8S_RRNA	0.00076216	RPF1/RPS21/ERI3/NOL9/EIF6/MAK16/EXOSC8/NSA2/KRI1/LAS1L/WDR12/FTSJ3/MTREX/ABT1/UTP20/MPHOSPH6/RCL1/EXOSC10/EXOSC2/EXOSC7/NOP9/EXOSC9/PRKDC/EXOSC4/PES1/URB1/ERI2/RRS1/BOP1/ERI1/NOP14

GOCC_DISTAL_AXON	GOCC_DISTAL_AXON	0.000763222	UCN3/KCNA1/GHRH/PENK/CHRM2/SLC18A3/GRIK3/STMN2/NGFR/PCSK1/SCGN/GPM6A/PCDH9/BOC/SLC8A2/GAP43/PTPRN/GRIK5/KCNK2/DPYSL3/MAP1B/NPY/FLNA/IGF2BP1/TNN/SLC18A2/CNR1/DIXDC1/MAPT/APBB1/PALLD/FGF13/L1CAM/FEZ1/SLC8A1/PRKCB/SNAP25/LAMP5/LRRK2/SNCG/BRSK1/TSHZ3
GOBP_TELOMERE_CAPPING	GOBP_TELOMERE_CAPPING	0.000773708	RAD50/MAPKAPK5/SMG6/PNKP/MAP3K4/NABP2/TERF2/TFIP11/RTEL1/MAP2K7/ACD/STN1/XRCC1/USP7/MAPK3/NEK2/HNRNPD/PRKDC/AURKB/NBN/DCLRE1B/PRKCQ/TNKS/DCLRE1A/MAPK15

GOBP_ANTIGEN_PROCESSING_AND_PRESENTATION_OF_PEPTIDE_ANTIGEN_VIA_MHC_CLASS_I	GOBP_ANTIGEN_PROCESSING_AND_PRESENTATION_OF_PEPTIDE_ANTIGEN_VIA_MHC_CLASS_I	0.000779173	TAPBPL/IKBKB/ACE/PDIA3/AZGP1/IDE/HLA-H/TAPBP/CALR/ERAP1/HLA-E/HLA-B/HLA-A/MFSD6/HLA-F/HLA-C/ERAP2/TAP2/TAP1
GOBP_RESPONSE_TO_MECHANICAL_STIMULUS	GOBP_RESPONSE_TO_MECHANICAL_STIMULUS	0.000779139	MPO/KCNA1/ATP1A2/TACR1/ANKRD1/NRXN2/KCNA5/HTR2A/DMD/KRT5/KCNK2/CDH2/MAP1B/DDR2/PTN/FGF2/ACTA1/CXCL12/IL33/AGT/THBS1/PDE2A/SERPINE2/FOSB/SLC8A1/PKD2/MMP2/BDKRB1/TMEM150C/SLITRK6/PIEZO2/RETN

GOBP_CELL_CHEMOTAXIS	GOBP_CELL_CHEMOTAXIS	0.000789391	PLA2G1B/AZU1/CCL16/TAFA4/AGTR1/THBS4/CTSG/SLIT2/CCL26/PTN/SMOC2/FGF2/GREM1/JAM3/CXCL6/CXCL12/F7/PRKD1/CHGA/CCL21/ITGA9/CH25H/THBS1/CCL19/S100A8/CX3CR1/CNR2/C10orf99/LPAR1/HGF/FGFR1/CYP7B1/TGFB2/ITGA1/MMP2/IL6/S100A9/SERPINE1/PDGFRA/CCL2/FOLR2/GPR183/CCN3/PARVA/PDGFD/CCL11/FGF1/MIR149/S1PR1/LOX/DUSP1/BST1/CXCR2/CD300H/RARRES2/HSPB1/CAMK1D/SCG2/EGR3/CXCR1/S100A12/RIPOR2/VEGFC/MPP1/CXCR4/CCR7/ACKR3/IL34/LEF1/KIT/PDGFRB/GAB1/FFAR2/EDNRB/NRP1/TNFSF18/EDN2/FPR2

GOBP_B_CELL_MEDIATED_IMMUNITY	GOBP_B_CELL_MEDIATED_IMMUNITY	0.000790992	HLA-DPA1/PRKCD/SLA2/IGHV4-61/TFRC/MYD88/C4BPB/PAXIP1/IGHV3-30/HLA-DMB/HLA-E/NECTIN2/TRDC/HLA-DOB/HLA-DRA/CD46/HMCES/TRAF3IP2/HLA-DQA2/UNG/KMT5C/IGLL5/HLA-DQA1/IGHA1/IL27RA/NBN/MSH2/IL21R/HSPD1/HLA-DRB5/FOXJ1/CD74/TCIRG1/IGHD/CD27/IGHV3-72/IGHV3-64D/HLA-DRB1/HLA-DOA/IGHV3-33/PARP3/EXO1/NSD2/HLA-DQB1/HLA-DQB2/IGHV3-48/C1QBP/C8G/IGHV3-66/HLA-DMA/CD55/IGHA2/IGHV1-58/IGHM/IGHV3-49/TNFSF13/CD70/IGHV3-13/IL10/C6/IGLL1

GOMF_PEPTIDASE_REGULATOR_ACTIVITY	GOMF_PEPTIDASE_REGULATOR_ACTIVITY	0.000794118	AHSG/CSN2/NLRP7/ITIH2/SERPIND1/SERPINA10/SFRP2/GPC3/PI16/CST4/FBLN1/NGF/PI15/TFPI2/SERPINA6/ITIH3/SERPINF2/PCOLCE2/AMBP/AGT/ITIH5/RECK/SERPINF1/CAV1/CRB2/SERPINA3/MAL/C4A/SERPINE2/C4B/TIMP4/BEX3/SERPINA5/WFDC1/SERPINE1/SPOCK1/PROS1/FN1/CSTA/TIMP2/A2M/CST2/DPEP1/PCSK1N/R3HDML/PAPLN/WFDC3/CST5/COL6A3/PRNP/A2ML1/WFDC10B/PCOLCE/TIMP3/SERPING1/TFPI/ANOS1

GOBP_RESPONSE_TO_ALCOHOL	GOBP_RESPONSE_TO_ALCOHOL	0.000796195	UCN3/PENK/SFRP1/ADIPOQ/TACR1/ADCYAP1R1/SLC2A4/KCNMB1/CDO1/ADCY5/CES1/GRIN2A/SLIT2/SLIT3/ADCY2/PTGFR/RGS4/CYP1B1/PRKAA2/CNR1/ADCY1/F7/CCL21/ADH7/CCL19/CSF3/DYNAP/S100A8/RGS2/KLF9/FOSB/MIR342/AVPR1A/SMO/IGFBP7/A2M/CTSK/NEFL/TGFBR3/CLDN5/GNAI1/INHBA/GABBR1/PTCH1/GPLD1/CHRNB2

GOBP_MRNA_3_END_PROCESSING	GOBP_MRNA_3_END_PROCESSING	0.000799042	RPRD2/AHCYL1/CPEB3/PCF11/APLP1/SCAF8/YTHDC1/RPRD1B/CSTF1/ZC3H14/RPRD1A/CDC73/CLP1/PNPT1/PAPOLA/WDR33/APP/VIRMA/SYMPK/CPSF7/CPSF6/TENT4B/MTPAP/SSU72/TENT2/CPSF2/ZNF473/CSTF3/CPSF3/LEO1/CPSF4/POLR2D/TUT1/NCBP1/FIP1L1/HSF1/PABPC1/BARD1/NUDT21/ZC3H3/CSTF2/SNRPA/CPSF1/GRSF1/SLBP

GOBP_CELLULAR_AMINO_ACID_METABOLIC_PROCESS	GOBP_CELLULAR_AMINO_ACID_METABOLIC_PROCESS	0.000799775	QDPR/ACADSB/GARS1/OTC/CARS1/ASL/DALRD3/ADI1/HARS1/SARS2/DLST/FARSB/SMS/PLOD3/MRI1/ICMT/HMGCL/FN3K/GSS/LRRC47/ENOPH1/PCCB/ACAD8/MTHFD2L/AGMAT/GATB/RARS1/NARS1/BAAT/GART/MPST/GSTZ1/MARS1/CARNMT1/KYAT3/HARS2/ASS1/PYCR3/IL4I1/EPRS1/KARS1/AZIN1/EARS2/ASPG/ODC1/MTHFR/PFAS/MTAP/ALDH1A1/QARS1/CAD/THAP4/IVD/PCBD1/CTPS1/IARS1/HIBCH/SARS1/TARS2/FPGS/IARS2/VARS1/ILVBL/GOT2/BCAT2/DHFRP1/GFPT1/YARS1/NARS2/SHMT1/LARS2/MARS2/ALDH18A1/MSRA/MCCC2/FARSA/TST/PPAT/GCAT/DHFR/DDAH1/ALDH4A1/ECHS1/PYCR1/MTHFD1/GOT1/HPDL/SHMT2/PSAT1/NOS2/BCAT1/GPT2/PRODH/SLC7A11/CARNS1/GAD1/CTH/GPT/WARS1/CPS1/IDO1/Q[illegible]
GOBP_POSITIVE_REGULATION_OF_CYTOKINESIS	GOBP_POSITIVE_REGULATION_OF_CYTOKINESIS	0.000803327	PKN2/AURKB/KIF20B/CDC14B/KIF23/KIF14/DRD2/GIPC1/CIT/INCENP/CENPV/RACGAP1/ECT2/BIRC5/CDCA8/IGF1R/SSTR5
GOBP_G_PROTEIN_COUPLED_RECEPTOR_SIGNALING_PATHWAY_COUPLED_TO_CYCLIC_NUCLEOTIDE_SECOND_MESSENGER	GOBP_G_PROTEIN_COUPLED_RECEPTOR_SIGNALING_PATHWAY_COUPLED_TO_CYCLIC_NUCLEOTIDE_SECOND_MESSENGER	0.000803505	CHRM2/HTR2A/HTR2B/NPY/CNR1/PTH1R/AGT/LPAR3/CNR2/CHRM4/CCL2/SSTR2/PTGIR

GOBP_REGULATION_OF_BIOMINERALIZATION	GOBP_REGULATION_OF_BIOMINERALIZATION	0.000819119	AHSG/OMD/MGP/DDR2/PTN/GREM1/ASPN/GPM6B/TGFB3/CCN1/SLC8A1/TMEM119/COMP/OSR1/ADRB2/NELL1/WNT10B/S1PR1/BMPR1B/KL/MEF2C/LTBP3/TWIST1/ACVR1/SRGN/FBLN7/AMTN/FAM20C/BMP6
GOBP_NADH_METABOLIC_PROCESS	GOBP_NADH_METABOLIC_PROCESS	0.00082078	ENO2/FOXK2/FOXK1/GPD1/MDH1/GPD2/IDH3B/HK3/PCK2/PGK1/MDH2/NUDT12/VCP/PFKL/TPI1/NQO1/TP53/MIR210/ENO1/PFKP/PKM/HK2
GOBP_POSITIVE_REGULATION_OF_NON_CANONICAL_WNT_SIGNALING_	GOBP_POSITIVE_REGULATION_OF_NON_CANONICAL_WNT_SIGNALING_PATHWAY	0.000830441	SFRP1/GPC3/RSPO3/DAB2/ANKRD6/DKK1

GOBP_PROTEIN_TARGETING_TO_MITOCHONDRION	GOBP_PROTEIN_TARGETING_TO_MITOCHONDRION	0.000831037	TIMM8A/UBE2L3/USP36/TOMM40L/HTRA2/LMAN1/PAM16/TIMM17B/HAX1/DNAJC15/SAE1/IMMP1L/ATG13/TIMM23/CSNK2A2/TIMM23B/HUWE1/TIMM17A/UBE2D3/FBXO7/BAP1/RAC2/TIMM50/UBE2J2/TOMM70/MFN2/TIMM22/BID/TIMM8B/SAMM50/TOMM34/HSP90AA1/TOMM22/MTX1/MTX2/CHCHD4/GSK3A/PMPCB/HSPA4/GRPEL2/HPS4/TIMM29/TOMM5/NDUFA13/BAG4/SREBF2/BCAP31/ATP5IF1/AIFM1/TIMM21/MIPEP/MICALL2/GRPEL1/FZD5/TIMM13/HSPD1/PMPCA/TOMM40/TIMM44/TSPO/SREBF1
GOBP_CARDIAC_MUSCLE_CELL_CONTRACTION	GOBP_CARDIAC_MUSCLE_CELL_CONTRACTION	0.0008388	ATP1A2/PLN/CTNNA3/ANK2/KCNA5/ABCC9/FLNA/CACNA1C/SGCD/SCN4B/KCNJ8/CACNB2/KCNE4/KCNH2/GJC1/CAV1/FGF13/CACNA2D1/GJA1/RYR2/STC1/FGF12

GOCC_SARCOPLASM	GOCC_SARCOPLASM	0.000841435	CASQ2/BAALC/PLN/CASQ1/SLC2A4/JPH2/THBS4/AKAP6/KLHL41/RYR3/IRAG1/SGCD/CMYA5/HRC/THBS1/DMPK/SCN3A/CACNA2D1/SPOCK1/RASD1/SLN/ITPR1/RYR2/MEF2C/JPH3/CTHRC1/SRL/GSN
GOBP_ANTIMICROBIAL_HUMORAL_IMMUNE_RESPONSE_MEDIATED_BY_ANTIMICROBIAL_PEPTIDE	GOBP_ANTIMICROBIAL_HUMORAL_IMMUNE_RESPONSE_MEDIATED_BY_ANTIMICROBIAL_PEPTIDE	0.000856874	PLA2G1B/ELANE/REG3G/DEFA4/RNASE3/REG1B/F2/DEFA6/CXCL6/CAMP/S100A9/DEFB126/PGLYRP1

GOBP_MEIOTIC_CELL_CYCLE_PROCESS	GOBP_MEIOTIC_CELL_CYCLE_PROCESS	0.000857781	NSUN2/CALR/EHMT2/FANCM/SLX4/NUF2/RAD54B/EME1/MUS81/SPIRE2/NCAPH2/BRCA2/RAD21/SMC4/TTK/NCAPD2/CYP26B1/SMC1A/SGO1/MSX1/FBXO5/FZR1/SIRT7/NDC80/BUB1/ASPM/RMI1/AURKA/RAD51AP1/CDC25C/DMC1/SMC2/CKS2/PTTG1/EME2/MSX2/CENPX/CCNB2/TESMIN/INCENP/KIF18A/BUB1B/NDC1/CDC20/RAD51/NCAPH/FANCD2/ESPL1/NCAPD3/MAPK15/PLK1/TOP2A/RAD54L/FANCA/CDC25A/CCNE2/SYCE2/BRIP1/REC8/TEX19

GOMF_TRANSLATION_REGULATOR_ACTIVITY_NUCLEIC_ACID_BINDING	GOMF_TRANSLATION_REGULATOR_ACTIVITY_NUCLEIC_ACID_BINDING	0.000863903	EIF3J/GFM1/MIF4GD/EIF1/RPS27L/EIF2S3/HBS1L/GSPT1/DENR/EEF1A1P5/EIF2D/EIF2B5/EIF1AX/EIF3B/EEF1A1/EIF4E2/EIF3D/EIF2B2/EIF3H/ABTB1/GTPBP1/EIF3E/EIF3I/EEF2K/EEF1D/EEF1B2/EEFSEC/EIF4G1/COPS5/MRPL58/EIF3A/EIF3G/EIF4B/EIF1AD/EIF4H/PABPC1/EIF2S1/EEF2/EIF4E/GFM2/GCN1/AGO2/LARP1/MTIF2/EIF3CL/EIF2S3B/DHFRP1/TUFM/SHMT1/EIF3C/DHFR/EIF5A/SHMT2/TYMS/EEF1A2/EIF5AL1/EFL1

GOBP_MITOTIC_SPINDLE_ASSEMBLY	GOBP_MITOTIC_SPINDLE_ASSEMBLY	0.000868243	RHOA/STAG2/DRG1/POLDIP2/CCDC61/TPR/AAAS/BCCIP/SMC3/CHMP6/EML3/GOLGA2/KPNB1/STAG1/CHMP2B/PDCD6IP/CHEK2/RAB11A/CHMP7/KIF2A/CHMP1A/TPX2/CEP192/VPS4B/HNRNPU/CHMP5/NEK2/SMC1A/PRC1/CCSAP/AURKB/KIF23/WRAP73/MZT1/KIFC1/INCENP/KIF4A/RACGAP1/CDC20/CHMP4C/KIFC2/MISP/PLK1/BIRC5/CDCA8/KIF15/KIF11
GOMF_EXTRACELLULAR_MATRIX_BINDING	GOMF_EXTRACELLULAR_MATRIX_BINDING	0.000867097	VTN/SLIT2/SPARCL1/SSC5D/ITGA7/SMOC2/DCN/ELN/THBS1/CCN1/SHH/FBLN2/OLFML2B/BCAM/TGFBI/NID1/SPARC/BGN/ITGB3/LGALS1/ADAMTS5/CD248/CLEC14A/THSD1/PXDN/NTN4/OLFML2A

GOBP_SEQUESTERING_OF_CALCIUM_ION	GOBP_SEQUESTERING_OF_CALCIUM_ION	0.000884808	CASQ2/ATP1A2/PLN/CASQ1/JPH2/ANK2/AKAP6/F2/HTR2A/DMD/RYR3/HTR2B/FLNA/CACNA1C/FGF2/TRPC1/HRC/PRKD1/CCL21/CCL19/SLC8A1/PKD2/BDKRB1/ITPR1/RYR2/CLIC2/THY1/JPH3/ANXA6/MCOLN3/ITGB3/GPER1/CCR7
GOBP_RESPONSE_TO_STEROID_HORMONE	GOBP_RESPONSE_TO_STEROID_HORMONE	0.000884389	APOA2/UCN3/NKX2-2/BCHE/SOX10/ATP1A2/SFRP1/ADIPOQ/TACR1/PCSK1/CDO1/SLIT2/PGR/LMO3/GHR/AR/SLIT3/CYP1B1/CPN1/NR4A3/ACTA1/RXRG/FBXO32/TGFB3/THBS1/SERPINF1/KANK2/CAV1/NODAL/CYP7B1/FIBIN/TGFB2/SCGB1A1/KLF9/FOSB/HEYL/IL6/ESR1/ESRRG/FAM107A/AVPR1A/DAB2/IGFBP7/A2M/ISL1/TCF21/OXTR/CCN2/SSTR2/SCGB2A1/PTGDS/NEFL/LOX/STC1/PAPPA/HSPA1A/TFPI

GOMF_METAL_ION_TRANSMEMBRANE_TRANSPORTER_ACTIVITY	GOMF_METAL_ION_TRANSMEMBRANE_TRANSPORTER_ACTIVITY	0.000901089	KCNA1/ATP1A2/SCN7A/GRIK3/KCNB1/KCNMA1/KCNK3/GRIK1/GPM6A/KCNMB1/GRIN2A/KCNA5/SLC13A5/SLC8A2/ABCC9/RYR3/GRIK5/KCNK2/KCNH6/SLC38A3/KCNT2/KCNIP1/CACNA1C/TRPC4/SLC18A2/CACNG4/CNR1/TRPC1/CACNA1H/SCN4B/NIPAL4/KCNJ8/CACNB2/KCNE4/SLC17A7/KCNH2/SLC39A2/NCS1/ATP2B4/TRPM6/TUSC3/SCN3A/SLC30A2/SLC1A7/SLC8A1/PKD2/CACNA2D3/CACNA2D1/CACNA1A/KCNG1/SNAP25/SLC9A9/SLC10A4/KCNQ3/SLC17A4/KCND1/SLC24A3/SLC5A9/KCNS1/KCND2/ITPR1/RYR2/SLC34A3/SLC18A1/SLC10A6/ANXA6/MCOLN3/SLC30A10
GOBP_ANTIGEN_PROCESSING_AND_PRESENTATION_OF_ENDOGENOUS_PEPTIDE_ANTIGEN	GOBP_ANTIGEN_PROCESSING_AND_PRESENTATION_OF_ENDOGENOUS_PEPTIDE_ANTIGEN	0.000907022	AZGP1/IDE/HLA-H/TAPBP/ERAP1/HLA-E/HLA-DRA/HLA-B/HLA-A/HLA-F/HLA-C/ERAP2/TAP2/HLA-DRB1/TAP1
GOBP_POSITIVE_REGULATION_OF_BEHAVIOR	GOBP_POSITIVE_REGULATION_OF_BEHAVIOR	0.000906932	GHRH/PENK/GRPR/GRP/NPY/NLGN1/NR4A3/SGIP1

GOMF_POLYUBIQUITIN_MODIFICATION_DEPENDENT_PROTEIN_BINDING	GOMF_POLYUBIQUITIN_MODIFICATION_DEPENDENT_PROTEIN_BINDING	0.000911752	SQSTM1/SPRTN/FAAP20/OPTN/BABAM2/ZBTB1/ABRAXAS2/WDR81/PSMD4/IKBKG/ZRANB3/UBXN1/HDAC6/SHARPIN/NPLOC4/UFD1/RNF168/UIMC1/RNF169/BAG6/RAD23B/UBQLN1/ZFAND2B/AGAP3/UBL7/PRPF8/BRCC3/UBQLN4/ATRIP/RAD18/AGL/TNIP2/IKBKE/RAD23A/VCP/PARP10

GOBP_GASTRULATION	GOBP_GASTRULATION	0.000914885	HAND1/APOA1/CER1/SFRP1/ADIPOQ/VTN/MIR145/SIX2/SFRP2/NOG/GPC3/ITGA7/PHLDB2/MMP8/NR4A3/COL8A1/ITGA8/FOXF1/ATOH8/CRB2/NODAL/ITGA5/MMP2/OSR1/FN1/DKK1/FZD7/GJA1/FOXC2/DUSP1/TENM4/SOX17/PHLDB1/INHBA/ITGB3/WLS/MYADM/LEF1/ACVR1/PLPP3/COL5A1/COL7A1/COL12A1/COL5A2/APLNR/FGFR2/COL4A2
GOBP_POSITIVE_REGULATION_OF_PEPTIDYL_THREO	GOBP_POSITIVE_REGULATION_OF_PEPTIDYL_THREONINE_PHOSPHO	0.000920413	GCG/AZU1/TRIM6

GOBP_T_CELL_RECEPTOR_SIGNALING_PATHWAY	GOBP_T_CELL_RECEPTOR_SIGNALING_PATHWAY	0.000920374	HHLA2/UBE2N/PSEN1/CD247/RC3H2/RELA/EIF2B5/UBASH3A/BTN1A1/IKBKB/RPS3/BTN2A1/EIF2B2/PAWR/DGKZ/HLA-DPB1/RBCK1/CD226/TEC/PTPN6/BCL10/BTN3A3/KHDRBS1/DENND1B/TNFRSF21/LGALS3/BTN2A2/BTNL9/PRKD2/PLCG2/STK11/SHB/SLA2/LIME1/PTPN22/RIPK2/NECTIN2/BTN3A2/TRDC/BTN2A3P/HLA-A/FCHO1/HRAS/BTNL8/CD8A/CSK/STOML2/BCAR1/ZC3H12A/HLA-DRB1/PTPRJ/KCNN4/HLA-DQB1/BTN3A1/ADA/GBP1/CTLA4/LCK/EZR

GOMF_ENZYME_INHIBITOR_ACTIVITY	GOMF_ENZYME_INHIBITOR_ACTIVITY	0.000919573	APOA2/AHSG/CSN2/APOC3/SCG5/PLN/NLRP7/ITIH2/SERPIND1/PPP1R1A/PPP1R12B/SLIT2/PPP1R14A/SERPINA10/GPC3/PI16/CST4/NGF/PI15/PTN/TFPI2/SERPINA6/ITIH3/SERPINF2/AMBP/AGT/ITIH5/RECK/ATP2B4/SERPINF1/PRKAR2B/CRB2/RGS2/SERPINA3/C4A/SCGB1A1/SERPINE2/UGT1A7/C4B/TIMP4/SERPINA5/PKIA/WFDC1/SERPINE1/SPOCK1/PROS1/CSTA/TIMP2/PKIG/A2M/CDKN1C/LRRK2/PARVA/CST2/DPEP1/SLN/PCSK1N/R3HDML/PAPLN/WFDC3/CST5/COL6A3/PRNP/A2ML1/PINLYP/HSPB1/WFDC10B/TIMP3/SERPING1/TFPI/ANOS1/INKA2/PHACTR1/LRRC43

GOBP_NEGATIVE_REGULATION_OF_RESPONSE_TO_EXTERNAL_STIMULUS	GOBP_NEGATIVE_REGULATION_OF_RESPONSE_TO_EXTERNAL_STIMULUS	0.00093429	ELANE/SPX/CARTPT/APOA1/APOH/ADIPOQ/VTN/FGA/RGMA/MIR145/PTGIS/F2/SLIT2/IGF1/FGG/ADAMTS18/NPY/FGF2/PHLDB2/GREM1/SEMA3E/SERPINF2/MMRN1/PRKG1/FOXF1/GPR17/THBS1/SERPINF1/CNR2/HGF/FGL2/SERPINE2/WFDC1/NR1H4/SERPINE1/PDGFRA/PROS1/CCL2/KRT1/PGLYRP1/A2M/SYT11/ISL1/FFAR4/CCN3/CD200/SEMA6A/SEMA6D/MIR149/CLDN19/DUSP1/THBD/FAP/FGB/TEK/MIR222/LILRA2/TAFA5/SERPING1/TFPI/FCGR2B/SEMA6C/SEMA3G/SEMA5B/SEMA3D/ROBO1/IL20RB/GPER1/CD34/HAMP/SEMA3C/MYOZ1/NRP1/CR1/HTRA1/BACE1
GOCC_POLYSOMAL_RIBOSOME	GOCC_POLYSOMAL_RIBOSOME	0.00094651	RPS26/RPL11/RPL38/RPS21/NUFIP2/RPL24/PNPT1/RPL10A/RPL32/RPL6/EIF3H/RPS23/RPL30/RPL18/LARP4B/BTF3/RPL7A/RPS28/DHX9/RPL19/RPL36/LARP1/RPL41/RPL18A/RPL8

GOMF_OXIDOREDUCTASE_ACTIVITY_ACTING_ON_CH_OH_GROUP_OF_DONORS	GOMF_OXIDOREDUCTASE_ACTIVITY_ACTING_ON_CH_OH_GROUP_OF_DONORS	0.000945885	DHDH/NSDHL/SDR9C7/SDR42E1/GPD2/IDH3B/HSD17B4/IMPDH2/CBR4/DHRS7B/D2HGDH/ME2/LDHA/DCXR/CTBP2/HADH/AKR7A2/IMPDH1/RDH12/CTBP1/DHRS4L2/RDH13/RDH16/GRHPR/ADH1C/MDH2/DHRS11/AKR1A1/L2HGDH/HSD17B1/DHRS4/IDH3A/IDH2/CHDH/IDH1/BDH1/HSD3B7/DHRS2/PGD/DHRS1/SPR/SORD/DHRS13/SDR16C5/HMGCR/LIPF/FASN/PRXL2B/HSD17B2/GFUS/HSD11B2/LDHD/AKR1B10/MIOX/AKR7L/ALDH3A1/AKR7A3/AKR1B15

GOBP_EPIDERMAL_CELL_DIFFERENTIATION	GOBP_EPIDERMAL_CELL_DIFFERENTIATION	0.00094306	SLC9A3R1/FLG/KLF4/CYP27B1/CYP26B1/TRIM16/MED1/SULT2B1/IRF6/CASP3/SPRR2D/EVPL/USH1C/SLC44A4/HDAC1/JAG1/EZH2/OVOL3/S100A7/KEAP1/SCRIB/CDH3/MSX2/ADAM9/ESRP1/TPRN/DNASE1L2/GRHL2/KRT17/ST14/KRT80/PLS1/KRT6B/VDR/SFN/PLAAT4/KDF1/FLNB/FOXC1/KLK5/OVOL2/KRT7/ZBED2/EXPH5/FA2H/EPHA2/KRT8/KCNQ1/ABCA12/KRT16/HES5/SPRR2E/KRT81/FGF20/LCE3E/LCE3D/SPRR2B/SPRR2G

GOBP_REGULATION_OF_ENDOTHELIAL_CELL_MIGRATION	GOBP_REGULATION_OF_ENDOTHELIAL_CELL_MIGRATION	0.000946592	MIR483/APOH/MEOX2/SLIT2/BMPER/PTN/SMOC2/FGF2/DCN/PRKD1/AGT/AKT3/ATP2B4/THBS1/SERPINF1/ATOH8/FGFR1/ANGPT1/RHOJ/NRP2/MIR342/CCBE1/MIR27B/FOXC2/FGF1/ADGRA2/MIR149/SRPX2/RHOB/HSPB1/STC1/TEK/MEF2C/SPARC/MIR10B/ADAMTS9/PTPRM/GPLD1/ITGB3/MMRN2/VEGFC/ADGRB1/MIR196A1/STARD13/PLPP3
GOBP_RESPONSE_TO_MONOSACCHARIDE	GOBP_RESPONSE_TO_MONOSACCHARIDE	0.000949237	GCG/APOA2/UCN3/NKX2-2/ADIPOQ/NEUROD1/KCNB1/PCSK1/CMA1/ADCY5/PTPRN/GRIK5/PRKAA2/MIR320C2/NPTX1/GIP/THBS1/RFX6/SERPINF1/NKX6-1/SLC8A1/ZBTB20/NR1H4/COL6A2/CCN2/MIR320D1/GPR27/LPL/KLF7/NOX4

GOBP_ESTABLISHMENT_OF_PROTEIN_LOCALIZATION_TO_MITOCHONDRIAL_MEMBRANE	GOBP_ESTABLISHMENT_OF_PROTEIN_LOCALIZATION_TO_MITOCHONDRIAL_MEMBRANE	0.000954108	MAIP1/TOMM70/TIMM22/TIMM8B/SAMM50/HSP90AA1/TOMM22/CALM3/MTX1/MTX2/HSPA4/BCS1L/TIMM29/BAX/TOMM5/NDUFA13/AP3B1/TIMM13/TOMM40/COX18/BBC3/GZMB
GOBP_POSITIVE_REGULATION_OF_FIBROBLAST_PROLIFERATION	GOBP_POSITIVE_REGULATION_OF_FIBROBLAST_PROLIFERATION	0.000956615	PLA2G1B/NGFR/FGF10/IGF1/DDR2/AGT/ESR1/PDGFRA/FN1/PDGFD/PDGFC/ITGB3/CD248/PDGFRB

GOBP_NEGATIVE_REGULATION_OF_HYDROLASE_ACTIVITY	GOBP_NEGATIVE_REGULATION_OF_HYDROLASE_ACTIVITY	0.000956632	APOA2/AHSG/CSN2/APOA1/APOC3/VTN/NLRP7/ITIH2/SERPIND1/CRYAB/RGN/SERPINA10/SFRP2/GPC3/PI16/CST4/NGF/PI15/TFPI2/SERPINA6/ITIH3/PTX3/SERPINF2/AMBP/AGT/LEPR/ITIH5/RECK/THBS1/SERPINF1/FZD10/CRB2/RGS2/HGF/SERPINA3/TGFB2/C4A/SERPINE2/C4B/TIMP4/SERPINA5/WFDC1/SERPINE1/SPOCK1/PROS1/CSTA/TIMP2/A2M/LRRK2/CST2/DPEP1/PCSK1N/R3HDML/PAPLN/WFDC3/CST5/COL6A3/PRNP/A2ML1/WFDC10B/TIMP3/SERPING1/TFPI/ANOS1/MAGEA3
GOBP_VENTRICULAR_CARDIAC_MUSCLE_TISSUE_MORPHOGENESIS	GOBP_VENTRICULAR_CARDIAC_MUSCLE_TISSUE_MORPHOGENESIS	0.00095811	HAND1/NKX2-5/MYL3/NOG/ZFPM2/TPM1/EDNRA/TGFB2/ISL1/FOXC2/HEG1/RYR2/TGFBR3
GOBP_MATURATION_OF_5_8S_RRNA_FROM_TRICISTRONIC_RRNA_TRANSCRIPT_SSU_RRNA_5_8S_RRNA_LSU_RRNA	GOBP_MATURATION_OF_5_8S_RRNA_FROM_TRICISTRONIC_RRNA_TRANSCRIPT_SSU_RRNA_5_8S_RRNA_LSU_RRNA	0.000959823	EXOSC8/KRI1/WDR12/FTSJ3/ABT1/UTP20/RCL1/EXOSC10/EXOSC2/EXOSC7/NOP9/EXOSC9/PES1/URB1/ERI2/RRS1/BOP1/ERI1/NOP14

GOBP_MISMATCH_REPAIR	GOBP_MISMATCH_REPAIR	0.000961023	MSH3/MCM8/TDG/RPA3/SETD2/RPA2/PMS2/PMS1/HDAC10/XPC/MSH6/RPA1/MCM9/POLD3/MSH2/AXIN2/PCNA/EXO1/LIG1/PRKCG/TP73
GOBP_TRANSLATIONAL_ELONGATION	GOBP_TRANSLATIONAL_ELONGATION	0.000969832	SRP9/SECISBP2/GFM1/SELENOT/HBS1L/EEF1A1P5/EEF1A1/AARS1/DPH1/ABTB1/DPH7/ASCC2/GTPBP1/EEF2K/EEF1D/EEF1B2/EEFSEC/RACK1/EIF4A3/EEF2/ASCC3/GFM2/DPH2/MRPL44/SEPSECS/TUFM/ZNF598/EIF5A/EEF1A2/EIF5AL1/EFL1
GOBP_REGULATION_OF_BEHAVIOR	GOBP_REGULATION_OF_BEHAVIOR	0.000974033	GHRH/PENK/RELN/GRPR/HTR2B/GRP/NPY/CNR1/NLGN1/NR4A3/LEPR/SGIP1/RETN/PTGDS/DLG4/MEF2C/CHRNB2

GOBP_NEUROBLAST_PROLIFERATION	GOBP_NEUROBLAST_PROLIFERATION	0.000974033	KCNA1/SOX10/ASCL1/PTN/FGF2/EML1/GLI3/HHIP/CX3CR1/SMARCD3/SOX5/FGF13/SHH/SMO/LRRK2/ID4
GOBP_ACUTE_PHASE_RESPONSE	GOBP_ACUTE_PHASE_RESPONSE	0.000978294	REG3G/AHSG/HP/F2/PTGER3/CNR1/SERPINF2/SERPINA3/IL6/ORM2/FN1/F8/A2M
GOBP_VENTRICULAR_CARDIAC_MUSCLE_TISSUE_DEVELOPMENT	GOBP_VENTRICULAR_CARDIAC_MUSCLE_TISSUE_DEVELOPMENT	0.000978733	HAND1/NKX2-5/MYL3/NOG/ZFPM2/TPM1/EDNRA/TGFB2/HOPX/ISL1/FOXC2/HEG1/RYR2/TGFBR3/ADAMTS9/IRX3/TBX5
GOBP_PROTEIN_LIPID_COMPLEX_ASSEMBLY	GOBP_PROTEIN_LIPID_COMPLEX_ASSEMBLY	0.000992601	APOA2/APOA1/APOC3/APOB/SOAT2/APOA4/ABCA1/MTTP
GOBP_REGULATION_OF_PHOSPHOLIPASE_C_ACTIVITY	GOBP_REGULATION_OF_PHOSPHOLIPASE_C_ACTIVITY	0.001004007	ADCYAP1R1/HTR2A/HTR2B/FGF2/AGT/EDNRA/LPAR1/FGFR1/NMUR1/ESR1/AVPR1A/PDGFRA/SELE/RASGRP4/KIT/PDGFRB
GOBP_SYMPATHETIC_NERVOUS_SYSTEM_DEVELOPMENT	GOBP_SYMPATHETIC_NERVOUS_SYSTEM_DEVELOPMENT	0.001005485	HAND2/ASCL1/INSM1/GFRA3/PLXNA4/EDNRA/NRP2

GOBP_CARDIAC_RIGHT_VENTRICLE_MORPHOGENESIS	GOBP_CARDIAC_RIGHT_VENTRICLE_MORPHOGENESIS	0.001007	HAND1/HAND2/NKX2-5/ZFPM2/SMARCD3/TGFB2/ISL1
GOBP_NUCLEOSOME_ASSEMBLY	GOBP_NUCLEOSOME_ASSEMBLY	0.00101	SART3/H4C9/H3C1/H2BC11/H4C4/NAP1L4/SETSIP/GRWD1/H4C3/BRD2/H2BC9/SET/SPTY2D1/H1-2/POLE3/H4C2/H2BC18/ANP32B/H4C1/H2BC10/HMGB2/H1-3/SSRP1/H3C13/H3C2/H3C7/SOX9/H2BC12/NPM1/H2BC15/H2BC14/H3C6/H2AX/CHAF1A/H3C8/H2BC13/H2BC17/CHAF1B/DNAJC9/H3C11/H1-4/H3C3/ASF1B/MCM2/H1-5
GOBP_POSITIVE_REGULATION_OF_OSSIFICATION	GOBP_POSITIVE_REGULATION_OF_OSSIFICATION	0.001021736	TACR1/PTN/ZBTB16/GPM6B/TGFB3/CCN1/TGFB2/SLC8A1/TMEM119/OSR1/ADRB2/NELL1/WNT10B/BMPR1B/KL/MEF2C/ACVR1/OXT/FAM20C/SOX11/BMP6

GOCC_MIDBODY	GOCC_MIDBODY	0.001022207	CHMP6/EML3/RALA/BCL3/BIRC6/USP3/PIN1/PPP1CC/ALKBH4/CHMP2B/NUP62/CNTRL/PDCD6IP/SSNA1/CHMP7/RACK1/CHMP1A/PIK3CB/VPS4B/NAT10/ARL2BP/HNRNPU/HSPA5/CHMP5/NEK2/SAFB/CTDP1/RAB11FIP4/RAN/ZFYVE19/GNL3/ARF6/CIAO2B/HSP90B1/KATNB1/RAD21/URB2/CCDC124/KIF20A/PKN2/RAB8A/PSRC1/PRC1/DDX11/ANXA11/AURKB/ENTR1/PDXP/KIF20B/ASPM/SPAG5/KIF23/AURKA/EXOC6/KIF14/RASGEF1B/NUDC/SHCBP1/KEAP1/CDK1/TTC19/CIT/ANLN/ANXA2/CENPF/VPS37B/INCENP/CENPV/SLC2A1/KIF4A/RACGAP1/LAP3/APC2/RCC2/DNM2/CHMP4C/AGAP2/ECT2/CENPE/PITPNM1/TTLL12/PLK1/BIRC5/CEP55/CDCA8
GOCC_CAVEOLA	GOCC_CAVEOLA	0.001023865	ATP1A2/ADCYAP1R1/KCNMA1/PTGIS/SORBS1/BVES/AKAP6/KCNA5/HTR2A/TRPC4/GASK1A/CD36/ADRA1B/ATP2B4/CAVIN1/CAV1/CAVIN2/SMO/DLC1/LRRK2/EHD2/TFPI/PTCH1/SELE

GOBP_PANCREAS_DEVELOPMENT	GOBP_PANCREAS_DEVELOPMENT	0.00102959	NKX2-2/NEUROD1/FGF10/PCSK1/ALDH1A2/INSM1/CDH2/NKX3-2/IGF2/GIP/FOXF1/RFX6/NKX6-1/ONECUT1/IL6/SHH/SMO/ISL1
GOBP_NEGATIVE_REGULATION_OF_CARTILAGE_DEVELOPMENT	GOBP_NEGATIVE_REGULATION_OF_CARTILAGE_DEVELOPMENT	0.001032434	NOG/EFEMP1/NKX3-2/FRZB/GREM1/GLI3/WNT9A/CTSK/SNAI2/CCN4/PTHLH/LTBP3
GOBP_CALCIUM_ION_TRANSPORT_INTO_CYTOSOL	GOBP_CALCIUM_ION_TRANSPORT_INTO_CYTOSOL	0.001036859	PLA2G1B/ADCYAP1R1/JPH2/CACNA1C/P2RX2/CAV1/SLC8A1/CACNA2D1/RYR2/JPH3
GOBP_ESTABLISHMENT_OF_TISSUE_POLARITY	GOBP_ESTABLISHMENT_OF_TISSUE_POLARITY	0.001035606	SFRP1/SFRP2/PRICKLE2/GPC3/ROR2/RSPO3/PRICKLE1/DACT1/DAB2/ANKRD6/DKK1/GPC6/FOXF2/FZD7/BRSK1/CTHRC1/ROR1/VANGL2/JHY/FZD1/FUZ/PLEKHA4/NPHP1/BRSK2/TP63/NPHP3/PKHD1

GOCC_LUMENAL_SIDE_OF_ENDOPLASMIC_RETICULUM_MEMBRANE	GOCC_LUMENAL_SIDE_OF_ENDOPLASMIC_RETICULUM_MEMBRANE	0.001059265	HLA-DPB1/HM13/HLA-H/SPPL2B/TAPBP/CALR/HLA-DPA1/CANX/HLA-E/BCAP31/HLA-DRA/HLA-B/HLA-A/HLA-DQA2/HLA-DQA1/HLA-DRB5/CD74/HLA-F/HLA-C/HLA-DRB1/HLA-DQB1/HLA-DQB2
GOBP_GLUTAMATE_RECEPTOR_SIGNALING_PATHWAY	GOBP_GLUTAMATE_RECEPTOR_SIGNALING_PATHWAY	0.001072113	PLP1/FRRS1L/GRIK3/KCNB1/GRIK1/GRIN2A/GRIK5
GOMF_DNA_REPLICATION_ORIGIN_BINDING	GOMF_DNA_REPLICATION_ORIGIN_BINDING	0.001081186	POLA1/DHX9/GRWD1/DDX11/MCM10/HSPD1/CDC45/MCM5/ORC5/MCM2/ORC1
GOBP_PATHWAY_RESTRICTED_SMAD_PROTEIN_PHOSPHORYLATION	GOBP_PATHWAY_RESTRICTED_SMAD_PROTEIN_PHOSPHORYLATION	0.001092475	BMP3/AMHR2/GDF6/NOG/BMPER/GREM1/GDF10/TGFB3/LEFTY2/NODAL/TGFB2/RBPMS/EMILIN1/DAB2/DKK1/GDF9/GDF7/TGFBR3/INHBA/INHA/LDLRAD4/ACVR1/LRP1

GOBP_REGULATION_OF_AMINO_ACID_TRANSPORT	GOBP_REGULATION_OF_AMINO_ACID_TRANSPORT	0.001099931	SYT4/ATP1A2/SLC38A3/RGS4/AGT/SV2A/RGS2/AVPR1A
GOCC_DNA_REPAIR_COMPLEX	GOCC_DNA_REPAIR_COMPLEX	0.001103293	XRCC6/TP53BP1/ASCC1/NHEJ1/ASCC2/XRCC5/BRCA1/PAXX/KDM1A/PALB2/BRCA2/ASCC3/PRKDC/RCOR1/FANCI/FANCD2
GOCC_SARCOPLASMIC_RETICULUM	GOCC_SARCOPLASMIC_RETICULUM	0.001106036	CASQ2/PLN/CASQ1/SLC2A4/JPH2/THBS4/AKAP6/KLHL41/RYR3/IRAG1/SGCD/CMYA5/HRC/THBS1/DMPK/CACNA2D1/RASD1/SLN/ITPR1/RYR2/JPH3
GOBP_TETRAPYRROLE_BIOSYNTHETIC_PROCESS	GOBP_TETRAPYRROLE_BIOSYNTHETIC_PROCESS	0.001118661	NFE2L1/TMEM14B/PGRMC1/ABCB7/PPOX/RSAD1/SUCLA2/IBA57/FLVCR1/FXN/ABCB10/IREB2/SLC11A2/SLC25A39/SLC25A38/ATP5IF1/SLC6A9/COX15/COX10/TMEM14DP/CPOX/ALAS1/HMBS/TSPO

GOBP_POSITIVE_REGULATION_OF_POTASSIUM_ION_TRANSMEMBRANE_	GOBP_POSITIVE_REGULATION_OF_POTASSIUM_ION_TRANSMEMBRANE_TRANSPORTER_ACTI	0.001129035	KCNA1/ACTN2/KCNK3/ANK2/AKAP6/ATP1B2
GOMF_POTASSIUM_ION_TRANSMEMBRANE_TRANSPORTER_ACTIVITY	GOMF_POTASSIUM_ION_TRANSMEMBRANE_TRANSPORTER_ACTIVITY	0.001136583	KCNA1/ATP1A2/GRIK3/KCNB1/KCNMA1/KCNK3/GRIK1/KCNMB1/KCNA5/ABCC9/GRIK5/KCNK2/KCNH6/KCNT2/KCNIP1/KCNJ8/KCNE4/SLC17A7/KCNH2/PKD2/KCNG1/SNAP25/SLC9A9/KCNQ3/KCND1/SLC24A3/KCNS1/KCND2
GOBP_POSTSYNAPTIC_MEMBRANE_ORGANIZATION	GOBP_POSTSYNAPTIC_MEMBRANE_ORGANIZATION	0.001140271	RELN/FRRS1L/CHRDL1/NRXN2/GLRB/CDH2/NLGN4X/NLGN1/NLGN4Y/NLGN3/LRRC4/DLG4/COLQ/NLGN2/MUSK

GOMF_ACETYLTRANSFERASE_ACTIVITY	GOMF_ACETYLTRANSFERASE_ACTIVITY	0.001144729	USP22/NAA60/NAA20/PAFAH1B2/NAA15/HADHA/NAA80/HAT1/GTF3C4/KAT5/ESCO1/NAT9/NAA25/NAA50/MOGAT2/NAT10/NAA10/SRCAP/MCM3AP/BRCA2/ELP3/PAFAH2/NAA40/KAT2A/ACAA1/NAT1/SMARCE1/NAT2/PAFAH1B3/ACAT2/DLAT/LPCAT1/GCAT/FASN/GNPNAT1/MED24/ESCO2/LPCAT4
GOBP_ACTIN_FILAMENT_BASED_MOVEMENT	GOBP_ACTIN_FILAMENT_BASED_MOVEMENT	0.001150419	ATP1A2/PLN/CTNNA3/ANK2/KCNA5/ABCC9/ACTA2/FLNA/CACNA1C/SGCD/SCN4B/KCNJ8/CACNB2/KCNE4/TPM1/KCNH2/ACTC1/GJC1/CAV1/FGF13/CACNA2D1/FRMD6/MYH10/PARVA/GJA1/PDPN/RYR2/STC1/FGF12/EPDR1/GSN/SCN3B/SCN1B/SNTA1/GJA5/WIPF3/KCNJ5/LIMCH1

GOBP_DEVELOPMENTAL_GROWTH_INVOLVED_IN_MORPHOGENESIS	GOBP_DEVELOPMENTAL_GROWTH_INVOLVED_IN_MORPHOGENESIS	0.001167086	SYT4/SFRP1/FGF10/EPHA7/SLIT2/SFRP2/SLIT3/MAP1B/NGF/TNN/PLXNA4/NDN/CXCL12/SEMA3E/NTN1/MAPT/RND2/LPAR3/NKX6-1/EDNRA/FGF13/L1CAM/NRP2/ESR1/PRKN/SH3GL2/FLRT1/SHH/FN1/SPART/NLGN3/CRABP2/SEMA6A/CCL11/FGF1/TMEM108/SEMA6D/S1PR1/ISLR2/NRCAM/WASF1/SEMA6C/SYT1/SEMA3G/DCLK1/MAP2/SEMA5B/SEMA3D/CXCR4
GOBP_POSITIVE_REGULATION_OF_PHOSPHATIDYLINOSITOL_3_KINASE_SIGNALING	GOBP_POSITIVE_REGULATION_OF_PHOSPHATIDYLINOSITOL_3_KINASE_SIGNALING	0.001172802	RELN/MYOC/F2/IGF1/ROR2/FGF2/DCN/SEMA3E/AGT/CSF3/SELP/HGF/FGFR1/ANGPT1/TGFB2/PDGFRA/FN1/PDGFD/TEK/PDGFC/FLT3/GPER1/ROR1/KIT/PDGFRB

GOBP_PLASMA_LIPOPROTEIN_PARTICLE_CLEARANCE	GOBP_PLASMA_LIPOPROTEIN_PARTICLE_CLEARANCE	0.001180322	APOA2/APOA1/APOC3/ADIPOQ/APOB/SOAT2/GPIHBP1/CD36/VLDLR/NR1H4/MIR27B
GOBP_DNA_REPLICATION_CHECKPOINT_SIGNALING	GOBP_DNA_REPLICATION_CHECKPOINT_SIGNALING	0.00119167	RAD9A/NAE1/RAD9B/TOPBP1/TIPIN/TIMELESS/TICRR/CLSPN/DNA2/CDC45/CDT1/ORC1
GOBP_REGULATION_OF_LIPID_CATABOLIC_PROCESS	GOBP_REGULATION_OF_LIPID_CATABOLIC_PROCESS	0.001195544	APOA2/APOA1/APOC3/CIDEA/SCT/CNR1/LGALS12/HCAR1/APOA4/RARRES2/TWIST1/GPLD1/ACACB/ENPP7
GOMF_FATTY_ACID_BINDING	GOMF_FATTY_ACID_BINDING	0.001202233	AFP/ALB/FABP4/S100A8/RBP7/FABP3/S100A9/FFAR4/CRABP2/PTGDS/ACOX2/RBP1/RBP5/GSTM2/ACOXL/OXER1/RBP2/SNCA
GOBP_FOLIC_ACID_CONTAINING_COMPOUND_METABOLIC_PROCESS	GOBP_FOLIC_ACID_CONTAINING_COMPOUND_METABOLIC_PROCESS	0.001206266	FOLR1/MTHFD2L/ALDH1L1/GART/ATIC/MTHFR/GCH1/FPGS/DHFRP1/SHMT1/MTHFD2/MTHFD1L/DHFR/MTHFD1/SLC19A1/SHMT2/TYMS

GOBP_CELLULAR_RESPONSE_TO_ORGANIC_CYCLIC_COMPOUND	GOBP_CELLULAR_RESPONSE_TO_ORGANIC_CYCLIC_COMPOUND	0.001208154	AQP8/SOX10/CASQ2/PENK/CHRM2/NSG2/ATP1A2/SFRP1/HAND2/ADIPOQ/KCNMB1/ADCY5/CES1/AKAP6/CYP24A1/GNAO1/HTR2A/PGR/LMO3/AR/RYR3/HTR2B/UGT3A2/ADCY2/RGS4/FLNA/CYP1B1/PAK3/COLEC12/NR4A3/WT1/ADCY1/CALY/MN1/PDE3A/RXRG/FOXF1/FBXO32/ATP2B4/SERPINF1/KANK2/DYNAP/CAV1/CHRM4/PDE2A/NODAL/CYP7B1/KLF9/SLC8A1/HEYL/PKD2/MMP2/GPBAR1/GNAL/ESR1/PRKN/ESRRG/NR1H4/FAM107A/MIR342/SMO/DAB2/PALM/CCL2/FOLR2/ISL1/IGFBP5/TCF21/LRRK2/WNT10B/IFIT1/SNAI2/SSTR2/SCGB2A1/PTGER1/SLC26A3/RYR2/STC1/GNAI1/MEF2C/HSPA1A/TFPI/INHBA/GNA14/PTCH1/NR3C1/GPLD1/GNAZ/CYP1A1/P2RY1/FLT3
GOBP_RESPONSE_TO_LITHIUM_ION	GOBP_RESPONSE_TO_LITHIUM_ION	0.001218762	FABP4/ASCL1/SLC13A5/ACTA1/ADCY1/NFATC4/SHH

GOCC_COPII_COATED_ER_TO_GOLGI_TRANSPORT_VESICLE	GOCC_COPII_COATED_ER_TO_GOLGI_TRANSPORT_VESICLE	0.00122466	B2M/LMAN1/VMA21/SAR1B/TEX261/APP/KLHL12/SEC23IP/CNIH1/SCAP/TMED2/ERGIC1/IER3IP1/TMED4/USO1/SEC22B/GOLGA2/PDCD6/STX5/SEC24D/HLA-DPB1/TMED5/SEC24A/TMED7/SLC30A5/CTSC/YIF1A/SEC31B/VTI1A/TMED9/HLA-H/HLA-DPA1/FOLR1/ECPAS/TGFA/HLA-E/SREBF2/HLA-DRA/YIF1B/TMED3/HLA-B/LMAN2/HLA-A/HLA-DQA2/SEC13/HLA-DQA1/SEC24C/SEC23B/HLA-DRB5/SEC16A/TMED1/CD74/HLA-F/HLA-C/HLA-DRB1/HLA-DQB1/HLA-DQB2/PCSK9/SREBF1
GOBP_PROTEIN_LOCALIZATION_TO_CHROMOSOME_TELOMERIC_REGION	GOBP_PROTEIN_LOCALIZATION_TO_CHROMOSOME_TELOMERIC_REGION	0.001243067	CCT6A/ATR/NABP2/TERF2/MACROH2A1/XRCC5/ACD/DKC1/TCP1/CCT8/CCT2/CCT3/GNL3/BRCA2/PINX1/CCT4/CCT7/CCT5/WRAP53/TNKS/TERT
GOBP_MITOCHONDRIAL_DNA_METABOLIC_PROCESS	GOBP_MITOCHONDRIAL_DNA_METABOLIC_PROCESS	0.001248312	SESN2/PARP1/POLG/RRM2B/DNAJA3/STOML2/STOX1/TWNK/RRM1/LONP1/TP53/DNA2/ENDOG

GOBP_MITOTIC_G1_S_TRANSITION_CHECKPOINT_SIGNALING	GOBP_MITOTIC_G1_S_TRANSITION_CHECKPOINT_SIGNALING	0.001246812	CDK2/RPS27L/RPL26/RPA2/CRADD/CHEK2/PML/TRIAP1/PRKDC/PIDD1/CASP2/RFWD3/GTSE1/PLK2/TP53/MDM2/CCND1/MUC1
GOBP_ER_NUCLEUS_SIGNALING_PATHWAY	GOBP_ER_NUCLEUS_SIGNALING_PATHWAY	0.001252834	SPRING1/EIF2A/INSIG2/NFE2L2/GSK3B/MBTPS1/ATF4/ARHGEF10L/CREBZF/BCL2L11/ATF6B/AMFR/SCAP/TMED2/QRICH1/EIF2AK3/ERLIN2/PPP1R15B/LPCAT3/SELENOS/INSIG1/CCDC47/HSPA5/ATP2A2/EIF2S1/SREBF2/ZBTB7B/ERLIN1/TMEM33/TP53/ABCA7/SREBF1/AGR2

GOBP_POSITIVE_REGULATION_OF_CHEMOTAXIS	GOBP_POSITIVE_REGULATION_OF_CHEMOTAXIS	0.001260877	AZU1/THBS4/FGF10/SLIT2/CCL26/PTN/SMOC2/FGF2/CXCL12/F7/PRKD1/CCL21/THBS1/CCL19/CX3CR1/LPAR1/FGFR1/IL6/SERPINE1/NTF3/PDGFD/S1PR1/CXCR2/RARRES2/HSPB1/CAMK1D/SCG2/RIPOR2/VEGFC/CXCR4/CCR7/IL34/PDGFRB/NRP1/TNFSF18/EDN2/FPR2/P2RY12
GOBP_POSITIVE_REGULATION_OF_MUSCLE_CELL_DIFFERENTIATION	GOBP_POSITIVE_REGULATION_OF_MUSCLE_CELL_DIFFERENTIATION	0.001283288	SMYD1/MYOCD/MIR145/AKAP6/IGF1/RBM24/LAMA2/LAMA1/LMOD3/HOPX/SHH/EFEMP2/NID1/MEF2C/LAMC1/MAMSTR/GPER1/KIT/HAMP
GOBP_POSITIVE_REGULATION_OF_ACTIN_FILAMENT_BUNDLE_ASSEMBLY	GOBP_POSITIVE_REGULATION_OF_ACTIN_FILAMENT_BUNDLE_ASSEMBLY	0.001291388	APOA1/SYNPO2/SFRP1/MYOC/TACR1/FERMT2/SERPINF2/TPM1/TGFB3/PPM1E/LPAR1/CCN2/SYNPO/MTSS1/ARHGEF15/NRP1/PFN2

GOCC_CELL_CELL_CONTACT_ZONE	GOCC_CELL_CELL_CONTACT_ZONE	0.001299318	ATP1A2/DES/PGM5/CTNNA3/PCDH9/ANK2/AKAP6/KCNA5/CDH2/JAM2/JAM3/SCN4B/GJC1/FGF13/SLC8A1
GOBP_DICARBOXYLIC_ACID_METABOLIC_PROCESS	GOBP_DICARBOXYLIC_ACID_METABOLIC_PROCESS	0.001320266	ASL/DLST/SDHAF3/IDH3B/D2HGDH/ME2/ACSF3/FOLR1/MTHFD2L/ALDH1L1/PCK2/ATIC/KYAT3/ASS1/SDHB/GRHPR/MDH2/L2HGDH/SDHA/FPGS/FH/IDH2/GOT2/DHFRP1/IDH1/ACLY/SHMT1/ALDH18A1/MTHFD2/MTHFD1L/DHFR/ALDH4A1/LIPF/PCK1/MTHFD1/GOT1/SLC19A1/SHMT2/GPT2/PRODH/SLC7A11/GAD1

GOCC_CYTOSOLIC_RIBOSOME	GOCC_CYTOSOLIC_RIBOSOME	0.001319576	RPS25/RPL27A/RPLP1/RPL13/RPS15A/RPL11/RPS13/RPL34/RPL38/RPS24/RPS21/RPL24/RPLP2/RPS11/UBA52/RPS8/RPS16/RPL22/RPS4X/RPS12/RPL23A/RPL15/RPL10A/RPS10/FAU/RPL32/RPS27L/RPL26/RPS14/RPL6/RPS9/RPL37A/RPL13A/RPL14/RPS18/RPS3/RPS5/RPL4/RPS23/RPL30/MRPS11/RPS3A/RPL12/RPL26L1/RPL18/RPS7/FXR2/RPL7A/RPS28/RPL9/RACK1/RPL35/RPS19/RPL7/RPL3/RPS6/RPL7L1/RPLP0/MRPL1/RPS17/RPLP0P6/RPL29/RPL19/RPL36/RPS15/RPL41/RPSA/RPS2/RPL18A/RPL8/RPL17

GOBP_RESPONSE_TO_TRANSFORMING_GROWTH_FACTOR_BETA	GOBP_RESPONSE_TO_TRANSFORMING_GROWTH_FACTOR_BETA	0.001320201	APOA1/PENK/SFRP1/MYOCD/CIDEA/ANKRD1/AMHR2/CILP/FERMT2/DDR2/ASPN/TGFB1I1/GDF10/ZEB1/PDE3A/ITGA8/TGFB3/THBS1/CAV1/LTBP1/CX3CR1/SOX5/PDE2A/NODAL/TGFB2/FBN1/ONECUT1/SMAD9/COL3A1/MIR342/EMILIN1/DAB2/CLEC3B/GDF9/CDKN1C/CTSK/ZEB2/PDGFD/DKK3/LOX/LRRC32/FMOD/TGFBR3/CLDN5/MEF2C/LTBP3/HSPA1A/COL1A2/NR3C1/LDLRAD4/LTBP2/TET1/VASN/COL1A1/ACVR1/NREP/HTRA1/SPRED3/CD109/FGFR2/COL4A2
GOBP_REGULATION_OF_MUSCLE_TISSUE_DEVELOPMENT	GOBP_REGULATION_OF_MUSCLE_TISSUE_DEVELOPMENT	0.001326077	JPH2/TNN/IGF2/CDON/LMOD3/SHH/IGFBP5/SOX15/WNT10B/LOX/MEF2C/TWIST1/LEF1/ACVR1
GOBP_RESPONSE_TO_DOPAMINE	GOBP_RESPONSE_TO_DOPAMINE	0.00132716	CHRM2/NSG2/ADCY5/GNAO1/HTR2A/HTR2B/RGS4/FLNA/CALY/CHRM4/GNAL/PRKN/PALM/LRRK2/PTGER1/GNA14/GNAZ

GOBP_ALPHA_BETA_T_CELL_PROLIFERATION	GOBP_ALPHA_BETA_T_CELL_PROLIFERATION	0.001333847	PTPN22/SYK/HLA-E/IL23A/RIPK2/HLA-A/TYK2/LGALS9B/SH3RF1/ZBTB7B/IL18/PRKCQ/TNFRSF14/LGALS9/CD55/LGALS9C/IRF1/JAK2/CD274
GOBP_BRANCHING_MORPHOGENESIS_OF_AN_EPITHELIAL_TUBE	GOBP_BRANCHING_MORPHOGENESIS_OF_AN_EPITHELIAL_TUBE	0.001338858	FGF10/SLIT2/PGR/TMEM59L/AR/SIX2/SFRP2/NOG/ADAMTS16/GPC3/FGF2/GREM1/WT1/GLI3/SEMA3E/GREB1L/AGT/FOXF1/RSPO2/NFATC4/LAMA1/HHIP/EDNRA/PKD2/ESR1/SHH/CLIC4/SMO/PBX1/GDF7/WNT2B/TCF21/FOXC2/DCHS1/CCL11/FGF1/ILK/PTCH1/HOXA5/LEF1/ACVR1/GLI2/RASIP1/NRP1

GOBP_REGULATION_OF_ESTABLISHMENT_OF_PLANAR_POLARITY	GOBP_REGULATION_OF_ESTABLISHMENT_OF_PLANAR_POLARITY	0.00134477	SFRP1/SFRP2/PRICKLE2/GPC3/ROR2/RSPO3/PRICKLE1/DACT1/DAB2/ANKRD6/DKK1/GPC6/FZD7/CTHRC1/ROR1/VANGL2/JHY/FZD1/PLEKHA4
GOBP_NEGATIVE_REGULATION_OF_TRANSPORTER_ACTIVITY	GOBP_NEGATIVE_REGULATION_OF_TRANSPORTER_ACTIVITY	0.001347528	APOA2/CASQ2/ATP1A2/ACTN2/PLN/GRP/KCNE4/CAV1/GEM/PKD2/OSR1/REM1/SLN/MIR326/RRAD/CLIC2/TWIST1/MIR613/KCNAB1

GOMF_GROWTH_FACTOR_BINDING	GOMF_GROWTH_FACTOR_BINDING	0.001349022	COL2A1/NLRP7/NGFR/CHRDL1/EPHA7/GHR/IGFBP1/CD36/TGFB3/THBS1/LTBP1/IL1R1/IGFBP6/FGFR1/CCN1/NRP2/IGFBP4/COL3A1/PDGFRA/IGFBP7/A2M/IGFBP5/KLB/CCN2/SRPX2/LRRC32/KL/TGFBR3/LTBP3/COL1A2/LTBP2/ITGB3/VASN/FLT3/LIFR/IL6ST/COL1A1/ACVR1/PDGFRB/COL5A1/NRP1/HTRA1/CD109/FGFR2/LTBP4/NKD2/ENG/PXDN/NTRK2/IGFBP3/COL6A1
GOBP_STEROL_TRANSPORT	GOBP_STEROL_TRANSPORT	0.001354901	PNLIP/APOA2/CEL/APOA1/APOC3/ADIPOQ/ABCA8/CES1/APOB/SOAT2/CD36/CAV1/APOA4/SHH/MIR27B/ABCA1/MTTP/CLU/PTCH1/PLTP/ENPP7/LRP1/MIR613

GOBP_RIBONUCLEOSIDE_MONOPHOSPHATE_BIOSYNTHETIC_PROCESS	GOBP_RIBONUCLEOSIDE_MONOPHOSPHATE_BIOSYNTHETIC_PROCESS	0.001364087	RFK/DHODH/ADSS2/AMPD2/UMPS/GMPS/APRT/IMPDH2/ADK/UCK2/GART/NUDT2/ATIC/ADSS1/IMPDH1/PFAS/CAD/PRPS2/CDA/DCK/CMPK1/PPAT/PAICS/ADA
GOBP_NEGATIVE_REGULATION_OF_STRIATED_MUSCLE_CELL_APOPTOT	GOBP_NEGATIVE_REGULATION_OF_STRIATED_MUSCLE_CELL_APOPTOTIC_PROCESS	0.001363624	HAND2/MYOCD/NKX2-5/MIR145/SFRP2
GOBP_ASTROCYTE_DIFFERENTIATION	GOBP_ASTROCYTE_DIFFERENTIATION	0.001362981	NKX2-2/PLP1/F2/GAP43/NOG/ROR2/DAB1/MAPT/S100A8/SERPINE2/IL6/S100A9/TSPAN2/SHH/SMO/ID4/VIM/ROR1/IL6ST/LRP1/PLPP3

GOCC_CYTOSOLIC_LARGE_RIBOSOMAL_SUBUNIT	GOCC_CYTOSOLIC_LARGE_RIBOSOMAL_SUBUNIT	0.001365922	RPL27A/RPLP1/RPL13/RPL11/RPL34/RPL38/RPL24/RPLP2/UBA52/RPL22/RPL23A/RPL15/RPL10A/RPL32/RPL26/RPL6/RPL37A/RPL13A/RPL14/RPL4/RPL30/RPL12/RPL26L1/RPL18/FXR2/RPL7A/RPL9/RPL35/RPL7/RPL3/RPL7L1/RPLP0/MRPL1/RPLP0P6/RPL29/RPL19/RPL36/RPL41/RPL18A/RPL8/RPL17

GOBP_DEFENSE_RESPONSE_TO_SYMBIONT	GOBP_DEFENSE_RESPONSE_TO_SYMBIONT	0.001369701	APOBEC3G/DNAJC3/RNF26/IFI27/DHX58/APOBEC3H/POLR3E/NCBP1/POLR3H/DHX9/MLKL/DTX3L/DDX56/IRF2/POLR3B/IL1B/PML/IRF3/DDX21/POLR3A/ANKRD17/CNOT7/PYCARD/RTP4/TARBP2/MYD88/G3BP1/DDX60/ZDHHC11/PTPN22/AKAP1/EXOSC5/TRIM35/TTC4/TRIM21/IL23A/PDE12/ATG16L1/TRIM27/SKP2/NT5C3A/TRIM11/TLR3/IL12RB1/POLR3K/IKBKE/TRAF3IP2/TRIM28/TRIM41/EXOSC4/OAS3/TRIM26/ILF3/DDX1/MMP12/DHX15/TICAM1/IFITM1/UNC93B1/TKFC/MOV10/ARMC5/F2RL1/PHB2/STAT1/MICB/ISG20/OASL/TRIM25/CGAS/ZC3H12A/BST2/PMAIP1/DDIT4/FADD/BIRC3/C1QBP/CASP1/ATAD3A/GBP3/GBP1/POLR3G/PRF1/NLRC5/TRIM15/IRF1/IFNG/CXCL9/CXCL10/IFNE/DMBT1/AIM2/BPIFA1

GOBP_PROTON_MOTIVE_FORCE_DRIVEN_ATP_SYNTHESIS	GOBP_PROTON_MOTIVE_FORCE_DRIVEN_ATP_SYNTHESIS	0.001382703	ATP5MC2/NDUFB8/NDUFC2/NDUFB5/NDUFC1/NDUFA1/NDUFB4/ATP5MG/NDUFS8/NDUFB2/ATP5MF/NDUFA8/ATP6V1A/ATP5F1C/NDUFA6/NDUFB10/ATP5PD/NDUFS5/NDUFB3/NDUFA12/NDUFA5/NDUFA9/NDUFB6/NDUFA11/NDUFS6/NDUFA10/NDUFS2/ATP5F1EP2/NDUFA2/NDUFS1/NDUFS3/SDHD/NDUFB7/ATP5PO/NDUFAB1/ATP5MC1/ATP5PB/NDUFA13/SDHB/ATP5MC3/ATP5F1A/ANTKMT/SDHA/ATP5F1B/STOML2/ATP5ME/NDUFV1/NDUFS7/ATP5F1D
GOBP_UBIQUINONE_METABOLIC_PROCESS	GOBP_UBIQUINONE_METABOLIC_PROCESS	0.001387169	NDUFA9/UBIAD1/PPTC7/COQ8B/PDSS2/PDSS1/COQ9/COQ4/COQ3/NQO1/COQ2/AIFM2/FDXR

GOBP_MAINTENANCE_OF_LOCATION	GOBP_MAINTENANCE_OF_LOCATION	0.001386649	ALB/APOA1/CASQ2/CER1/ATP1A2/PLN/CASQ1/JPH2/CIDEA/ANK2/CES1/AKAP6/F2/HTR2A/APOB/DMD/RYR3/GRIK5/HTR2B/IGSF11/FLNA/CACNA1C/FGF2/SLC18A2/TRPC1/HRC/PRKD1/CD36/CCL21/DZIP1/CCL19/CAV1/PLA2G4C/S100A8/LTBP1/SLC30A2/FBN1/SLC8A1/PKD2/SYNE1/BDKRB1/IL6/S100A9/ABCA1/LPL/ITPR1/RYR2/FCER2/CLIC2/TMSB15A/THY1/MIR10B/JPH3/ANXA6/MCOLN3/ITGB3/ACACB/PLIN2/GPER1/CCR7
GOBP_INNER_MITOCHONDRIAL_MEMBRANE_ORGANIZATION	GOBP_INNER_MITOCHONDRIAL_MEMBRANE_ORGANIZATION	0.001393795	MAIP1/TOMM70/TIMM22/TMEM11/TIMM8B/SAMM50/CHCHD6/MTX1/MTX2/APOO/MICOS13/AFG3L2/TIMM29/IMMT/NDUFA13/UQCC3/HSPA9/CHCHD3/GHITM/TIMM13/DNAJC11/CHCHD10/LETM1

GOMF_CARBOXYLIC_ACID_BINDING	GOMF_CARBOXYLIC_ACID_BINDING	0.001395661	PLA2G1B/AFP/ALB/ADIPOQ/FABP4/GLRB/UGT2B17/SIGLEC6/S100A8/SELP/RBP7/FABP3/FCN1/UGT1A7/S100A9/SERPINA5/NR1H4/P4HA3/PLOD2/FOLR2/FFAR4/CRABP2/FTCD/PTGDS/ACOX2/LRAT/SELE/ACACB/EPDR1/RBP1/RBP5/SLC46A1
GOCC_ATPASE_COMPLEX	GOCC_ATPASE_COMPLEX	0.001401098	ING3/ARID2/ARID1A/CSNK2A1/RBBP7/BAZ1B/NCR1/ERCC6/SMARCA5/ACTR8/TRRAP/CHRAC1/VPS4A/SF3B1/SMARCD1/EP400/BRD7/BRD8/MTA1/ACTR5/DEK/ZNHIT1/SMARCC1/ACTL6A/RB1/KAT5/SMARCC2/HDAC2/UCHL5/INO80D/ACTR6/SMARCB1/MBD3/RUVBL1/INO80E/VPS4B/INO80/BCL11B/RUVBL2/DDX21/POLE3/MCRS1/SRCAP/ANP32E/RBBP4/NFRKB/BCL7A/INO80C/VCP/SMARCD2/GATAD2A/HDAC1/PTPA/SMARCE1/MTA2/SMARCA4/MYBBP1A

GOBP_NEGATIVE_REGULATION_OF_PEPTIDASE_ACTIVITY	GOBP_NEGATIVE_REGULATION_OF_PEPTIDASE_ACTIVITY	0.001412328	AHSG/CSN2/VTN/NLRP7/ITIH2/SERPIND1/CRYAB/SERPINA10/SFRP2/GPC3/PI16/CST4/NGF/PI15/TFPI2/SERPINA6/ITIH3/SERPINF2/AMBP/AGT/ITIH5/RECK/THBS1/SERPINF1/CRB2/HGF/SERPINA3/C4A/SERPINE2/C4B/TIMP4/SERPINA5/WFDC1/SERPINE1/SPOCK1/PROS1/CSTA/TIMP2/A2M/CST2/DPEP1/PCSK1N/R3HDML/PAPLN/WFDC3/CST5/COL6A3/PRNP/A2ML1/WFDC10B/TIMP3/SERPING1/TFPI/ANOS1/MAGEA3
GOBP_REGULATION_OF_SMOOTH_MUSCLE_CONTRACTION	GOBP_REGULATION_OF_SMOOTH_MUSCLE_CONTRACTION	0.001414905	SPX/CHRM2/CNN1/ATP1A2/TACR2/CHRNA3/TACR1/KCNMA1/MYOCD/MIR145/GUCY1A1/PRKG1/CHRNB4/P2RX1/CAV1/RGS2/ADRB2/OXTR

GOBP_POSITIVE_REGULATION_OF_ENDOTHELIAL_CELL_PROLIFERATION	GOBP_POSITIVE_REGULATION_OF_ENDOTHELIAL_CELL_PROLIFERATION	0.001427558	VIP/AGTR1/THBS4/FGF10/FGF7/CCL26/HTR2B/FGF2/IGF2/CXCL12/PRKD1/AKT3/FGFR1/NRP2/MIR27B/CCL11/SCG2/TEK/VASH2/EGR3/TNFSF12/MIR10B/ITGB3/VEGFC
GOBP_REGULATION_OF_SISTER_CHROMATID_COHESION	GOBP_REGULATION_OF_SISTER_CHROMATID_COHESION	0.001432308	CTCF/SMC5/WAPL/MACROH2A1/RB1/SFPQ/SLF1/NAA10/RAD21/DDX11/BUB1/TNKS/AXIN2/FEN1/ESPL1/CDCA5
GOBP_MYELOID_LEUKOCYTE_MEDIATED_IMMUNITY	GOBP_MYELOID_LEUKOCYTE_MEDIATED_IMMUNITY	0.001433628	PLA2G1B/ELANE/AZU1/CTSG/F2/GRP/CXCL6/NR4A3/CHGA/FOXF1/CX3CR1/IL6/PTGDS/BLK/FCGR2B/IL13RA2/CLNK/KIT/PTGDR
GOBP_REGULATION_OF_MUSCLE_ADAPTATION	GOBP_REGULATION_OF_MUSCLE_ADAPTATION	0.001431094	HAND2/CASQ1/SGCA/MIR145/AKAP6/IGF1/PI16/RGS4/NR4A3/PDE9A/AGT/FBXO32/ATP2B4/RGS2

GOBP_ACTIN_FILAMENT_BUNDLE_ORGANIZATION	GOBP_ACTIN_FILAMENT_BUNDLE_ORGANIZATION	0.001441999	APOA1/SYNPO2/SFRP1/MYOC/TACR1/SORBS1/CALD1/FERMT2/DPYSL3/FLNA/PHLDB2/ELN/ADD2/SERPINF2/TPM1/TGFB3/PPM1E/CGNL1/LPAR1/PRKN/FAM107A/DLC1/CCN2/SYNPO/ARHGAP28/MIR149/S1PR1/ACTN1/MTSS1/CORO2B/PHACTR1/SPIRE1/ARHGEF15/NRP1/RDX/PFN2
GOCC_CALCIUM_CHANNEL_COMPLEX	GOCC_CALCIUM_CHANNEL_COMPLEX	0.001455906	CASQ2/AKAP6/RYR3/CACNA1C/TRPC4/CACNG4/CACNA1H/HSPA2/CACNB2/MICU3/CACNA2D3/CACNA2D1/CACNA1A

GOBP_REGULATION_OF_INSULIN_SECRETION	GOBP_REGULATION_OF_INSULIN_SECRETION	0.001459436	GCG/UCN3/CARTPT/CELA2A/SFRP1/NEUROD1/NNAT/KCNB1/ADCY5/KCNA5/CNR1/GIP/CHGA/RFX6/NKX6-1/RBP4/IL6/PRKN/NR1H4/SNAP25/ISL1/CCN3/GPR27/KLF7/NLGN2/BLK/GPLD1/MYRIP
GOBP_REGULATION_OF_CARDIAC_MUSCLE_CONTRACTION	GOBP_REGULATION_OF_CARDIAC_MUSCLE_CONTRACTION	0.001464298	CASQ2/ATP1A2/PLN/NKX2-5/CTNNA3/ANK2/DMD/CACNA1C/HRC/CHGA/ADRA1B/CAV1/FGF13/RGS2/SLC8A1/CCN2/RYR2/STC1/CLIC2/PDE5A
GOMF_STRUCTURAL_CONSTITUENT_OF_NUCLEAR_PORE	GOMF_STRUCTURAL_CONSTITUENT_OF_NUCLEAR_PORE	0.001491987	NUP155/NUP107/NUP133/NUP153/NUP98/NUP214/TPR/NUP54/NUTF2/NUP160/NUP35/NUP62/NUP62CL/POM121C/NUP85/NUP58/NUP93/POM121/NUP205/NUP88/NDC1/NUP188/POM121B

GOCC_A_BAND	GOCC_A_BAND	0.001505912	ANK2/PPP1R12B/CRYAB/KLHL41/MYL3/SMPX/MYOM1/CMYA5/SMTNL2/LMOD3
GOBP_PERIPHERAL_NERVOUS_SYSTEM_DEVELOPMENT	GOBP_PERIPHERAL_NERVOUS_SYSTEM_DEVELOPMENT	0.001510899	SOX10/HAND2/MYOC/ASCL1/NFASC/GFRA3/NGF/PLXNA4/HOXD10/LGI4/CNTNAP1/RXRG/ISL1/NTF3/POU3F1/ILK/PMP22/EGR3/ADGRB2/ADGRB1/HOXD9/NEFH/EDNRB
GOBP_NEGATIVE_REGULATION_OF_DNA_RECOMBINATION	GOBP_NEGATIVE_REGULATION_OF_DNA_RECOMBINATION	0.001516326	FOXP3/MSH3/RECQL5/SHLD2/SHLD3/TP53BP1/MAD2L2/RIF1/H1-10/TERF2/RTEL1/SMCHD1/MSH6/H1-2/FANCB/UBQLN4/ZSCAN4/HELB/H1-3/MSH2/PARPBP/CGAS/PARP3/BLM/POLQ/RMI2/H1-4/H1-5

GOBP_MITOTIC_SISTER_CHROMATID_COHESION	GOBP_MITOTIC_SISTER_CHROMATID_COHESION	0.001529999	SMC5/SMC3/MACROH2A1/RB1/ESCO1/NAA50/SLF1/NAA10/DSCC1/PDS5A/SMC1A/SGO1/TNKS/CDC20/HASPIN/CDCA5/ESCO2
GOBP_PHENOL_CONTAINING_COMPOUND_METABOLIC_PROCESS	GOBP_PHENOL_CONTAINING_COMPOUND_METABOLIC_PROCESS	0.001528801	HAND2/TPH1/GRIN2A/SULT2A1/INSM1/PAH/NPY/TRPC1/DIO1/RNF180/EDNRA/NPR1/TGFB2/PDE1B/PRKN/CGA/TPO/DIO2/CTSK/MOXD1/ZEB2/HDC/KL/SLC16A2/CPQ/CHRNB2/SLC16A10/TYRP1

GOBP_ANTIGEN_PROCESSING_AND_PRESENTATION_OF_PEPTIDE_ANTIGEN	GOBP_ANTIGEN_PROCESSING_AND_PRESENTATION_OF_PEPTIDE_ANTIGEN	0.001535417	CTSD/TAPBPL/IKBKB/ACE/HLA-DPB1/PDIA3/CTSS/AZGP1/IDE/HLA-H/TAPBP/CALR/ERAP1/HLA-DPA1/PYCARD/HLA-DMB/HLA-E/HLA-DOB/HLA-DRA/HLA-B/HLA-A/HLA-DQA2/MFSD6/HLA-DQA1/CTSE/HLA-DRB5/CD74/HLA-F/HLA-C/ERAP2/TAP2/HLA-DRB1/HLA-DOA/HLA-DQB1/DNM2/HLA-DQB2/HLA-DMA/TAP1
GOBP_POSITIVE_REGULATION_OF_INTERLEUKIN_8_PRODUCTION	GOBP_POSITIVE_REGULATION_OF_INTERLEUKIN_8_PRODUCTION	0.001546521	PLA2G1B/ELANE/APOA2/ADIPOQ/FCN1/IL6/SERPINE1

GOBP_HISTONE_UBIQUITINATION	GOBP_HISTONE_UBIQUITINATION	0.001551445	PCGF3/UBE2E1/PCGF1/UBR5/TRIM37/HUWE1/UBE2N/PCGF6/USP22/RNF168/SUZ12/DDB1/CTR9/PARK7/LEO1/OTUB1/KDM2B/ATXN7L3/BRCC3/BRCA1/DTX3L/PCGF5/BCOR/KDM1A/BARD1/BRCA2/RNF40/CBX8/DDB2/OTUB2/RAD51/UHRF1
GOBP_REGULATION_OF_RELEASE_OF_SEQUESTERED_CALCIUM_ION_INTO_CYTOSOL	GOBP_REGULATION_OF_RELEASE_OF_SEQUESTERED_CALCIUM_ION_INTO_CYTOSOL	0.001556443	CASQ2/ATP1A2/PLN/CASQ1/JPH2/ANK2/AKAP6/F2/DMD/CACNA1C/TRPC1/HRC/PRKD1/SLC8A1/PKD2/BDKRB1/RYR2/CLIC2/THY1/JPH3/ITGB3/GPER1

GOBP_PROTEIN_POLYMERIZATION	GOBP_PROTEIN_POLYMERIZATION	0.001558886	CASQ2/STMN2/VTN/FGA/LMOD1/CASQ1/SLIT2/TMOD1/KRT5/FGG/CCL26/TUBB4A/MAP1B/CLIP3/PAK3/SVIL/NAV3/DAAM2/TRIM6/ELN/ADD2/WASF3/CDC42EP3/CCL21/MAPT/KANK4/CSF3/FGF13/LMOD3/TENM1/HDGFL3/PRKN/FAM107A/FHOD3/ANKRD53/KIRREL1/GAS7/CCL11/ARHGAP28/MAP7D3/NEFL/FGB/TMSB15A/HSPA1A/WASF1/TUBG2/CYRIA/MAP2/CCR7/SPIRE1/MYADM/GSN/RDX/ARHGAP40/PFN2
GOBP_REGULATION_OF_EPITHELIAL_CELL_APOPTOTIC_PROCESS	GOBP_REGULATION_OF_EPITHELIAL_CELL_APOPTOTIC_PROCESS	0.001565857	PLA2G1B/HAND2/NEUROD1/FGA/NKX2-5/FGG/SFRP4/THBS1/ANGPT1/IL6/ESR1/SERPINE1/CCL2/ATOH1/IL11/RAMP2/SCG2/FGB/TEK/H19/CD248/GPER1/ECSCR/NDNF/GSN

GOMF_TRANSLATION_INITIATION_FACTOR_ACTIVITY	GOMF_TRANSLATION_INITIATION_FACTOR_ACTIVITY	0.001568844	EIF2B4/DHX29/EIF2B3/MCTS1/EIF4G3/EIF4A1/EIF3M/EIF6/EIF3K/EIF3J/EIF1/EIF2S3/DENR/EIF2D/EIF2B5/EIF1AX/EIF3B/EIF4E2/EIF3D/EIF2B2/EIF3H/EIF3E/EIF3I/EIF4G1/COPS5/EIF3A/EIF3G/EIF4B/EIF1AD/EIF4H/EIF2S1/EIF4E/AGO2/MTIF2/EIF3CL/EIF2S3B/EIF3C
GOMF_DNA_POLYMERASE_BINDING	GOMF_DNA_POLYMERASE_BINDING	0.001587746	HMGB1/SMG6/HSP90AB1/NABP2/HSP90AA1/NHEJ1/RTEL1/ACD/NAT10/PTGES3/PAXX/SMARCA4/LONP1/PCNA/RAD51/FANCI/FANCD2/CDT1
GOBP_REGULATION_OF_CARDIAC_MUSCLE_CONTRACTION_BY_CALCIUM_ION_SIGNALING	GOBP_REGULATION_OF_CARDIAC_MUSCLE_CONTRACTION_BY_CALCIUM_ION_SIGNALING	0.001614456	CASQ2/ATP1A2/PLN/ANK2/DMD/CACNA1C/HRC/SLC8A1/RYR2/CLIC2

GOBP_DEOXYRIBONUCLEOSIDE_MONOPHOSPHATE_METABOLIC_PROCESS	GOBP_DEOXYRIBONUCLEOSIDE_MONOPHOSPHATE_METABOLIC_PROCESS	0.001622834	NT5C2/GDA/DGUOK/NT5M/DPYD/DNPH1/ADK/TK2/DCTD/NT5C/PNP/XDH/DCK/DUT/SHMT1/TYMP/ADA/TYMS/TK1
GOBP_POSITIVE_REGULATION_OF_CELL_CYCLE_CHECKPOINT	GOBP_POSITIVE_REGULATION_OF_CELL_CYCLE_CHECKPOINT	0.001642473	XRCC3/AURKB/NDC80/CCAR2/TELO2/GEN1/TTI2/INCENP/MAD2L1/BIRC5/CDCA8/PBK
GOBP_BODY_MORPHOGENESIS	GOBP_BODY_MORPHOGENESIS	0.001655951	MAB21L2/NOG/GREM2/GPC3/PHLDB2/CRISPLD1/CDON/TGFB3/CRISPLD2/MMP2/PDGFRA/DKK1/PHLDB1/CLDN5/LEF1/COL1A1/RRAS
GOCC_SPECIFIC_GRANULE_LUMEN	GOCC_SPECIFIC_GRANULE_LUMEN	0.001656202	ELANE/DEFA4/HP/BPI/MMP8/PTX3/CAMP/QPCT/ORM2/TIMP2/PGLYRP1/RETN

GOBP_POSITIVE_REGULATION_OF_SMALL_MOLECULE_METABOLIC_PROCESS	GOBP_POSITIVE_REGULATION_OF_SMALL_MOLECULE_METABOLIC_PROCESS	0.001663373	GCG/APOA1/ADIPOQ/ADCYAP1R1/SORBS1/CES1/HTR2A/RGN/IGF1/PRKAA2/PTH1R/NR4A3/IGF2/NCS1/PID1/GUCA2A/ZBTB20/NR1H4/APOA4/AVPR1A/DAB2/FGF1/PPP1R3G/TWIST1/NNMT/GPLD1/P2RY1/ELOVL5/GPER1
GOBP_NEGATIVE_REGULATION_OF_TELOMERE_MAINTENANCE	GOBP_NEGATIVE_REGULATION_OF_TELOMERE_MAINTENANCE	0.001669637	RAD50/SMG6/TENT4B/TERF2/RTEL1/XRN1/ACD/STN1/XRCC1/SRC/NAT10/HNRNPU/HNRNPC/SLX4/EXOSC10/PIF1/PARP1/PINX1/NBN/HNRNPA1/TNKS
GOBP_NEGATIVE_REGULATION_OF_ENDOTHELIAL_CELL_PROLIFERATION	GOBP_NEGATIVE_REGULATION_OF_ENDOTHELIAL_CELL_PROLIFERATION	0.001686167	MIR483/APOH/NGFR/THBS1/CAV1/ATOH8/MIR342/CCL2/MIR149/SCG2/MEF2C/MIR222/SPARC/PTPRM/MMRN2/SULF1/COL4A3/GHRL/CAV2/ALOX5/TGFBR1/RGCC

GOBP_POSITIVE_REGULATION_OF_VASOCONSTRICTION	GOBP_POSITIVE_REGULATION_OF_VASOCONSTRICTION	0.001695629	FGA/TACR1/HTR2A/FGG/ADRA1D/SMTNL2/ADRA1B/CAV1/AVPR1A/CYSLTR1/OXTR/AVPR2/FGB
GOBP_NEGATIVE_REGULATION_OF_TELOMERE_MAINTENANCE_VIA_TELOMERE_LENGTHENING	GOBP_NEGATIVE_REGULATION_OF_TELOMERE_MAINTENANCE_VIA_TELOMERE_LENGTHENING	0.001698823	TERF2/XRN1/ACD/STN1/SRC/NAT10/HNRNPU/HNRNPC/SLX4/EXOSC10/PIF1/PARP1/PINX1/HNRNPA1/TNKS
GOBP_NEGATIVE_REGULATION_OF_OXIDOREDUCTASE_ACTIVITY	GOBP_NEGATIVE_REGULATION_OF_OXIDOREDUCTASE_ACTIVITY	0.001702994	HP/AOC3/CNR1/ATP2B4/CAV1/CNR2/PRKN/MIR27B/LRRK2
GOCC_M_BAND	GOCC_M_BAND	0.001702994	ANK2/CRYAB/KLHL41/SMPX/MYOM1/CMYA5/SMTNL2/LMOD3
GOBP_REGULATION_OF_ORGAN_GROWTH	GOBP_REGULATION_OF_ORGAN_GROWTH	0.001713385	MAEL/NKX2-5/AKAP6/IGF1/NOG/KCNK2/PI16/RGS4/ZFPM2/FGF2/IGF2/WT1/GLI1/RGS2/RBP4/SMO/CGA/FOXC2/DUSP9/TGFBR3/MEF2C/MIR222/ACACB/TBX5

GOBP_MITOTIC_CHROMOSOME_CONDENSATION	GOBP_MITOTIC_CHROMOSOME_CONDENSATION	0.001721029	TENT4A/CHMP1A/KMT5A/AKAP8/NCAPH2/SMC4/NCAPD2/SMC2/NCAPH/NCAPD3/CDCA5/PLK1/NCAPG
GOBP_GLUCOSE_6_PHOSPHATE_METABOLIC_PROCESS	GOBP_GLUCOSE_6_PHOSPHATE_METABOLIC_PROCESS	0.001736366	RPTOR/RPE/PGLS/HKDC1/SHPK/TALDO1/HK3/MTOR/MLST8/TKT/PRPS2/PGAM1/PGD/GPI/TP53/HK2
GOBP_POSITIVE_REGULATION_OF_TYPE_I_INTERFERON_PRODUCTION	GOBP_POSITIVE_REGULATION_OF_TYPE_I_INTERFERON_PRODUCTION	0.001744108	POLR3D/RIOK3/IFIH1/IRF7/OAS1/SETD2/TOMM70/DHX33/HSP90AA1/OAS2/DHX58/DHX9/POLR3B/PLCG2/IRF3/POLR3A/MYD88/G3BP1/PTPN22/SYK/RIPK2/TLR3/CHUK/HMGB2/OAS3/MMP12/TICAM1/IRAK1/HSPD1/STAT1/CGAS/GAPDH/POLR3G/TRIM15/IRF1
GOBP_HYDROGEN_PEROXIDE_CATABOLIC_PROCESS	GOBP_HYDROGEN_PEROXIDE_CATABOLIC_PROCESS	0.001750778	MPO/HP/APOA4/HBB/GPX3/TPO
GOBP_NEGATIVE_REGULATION_OF_AMINE_TRANSPORT	GOBP_NEGATIVE_REGULATION_OF_AMINE_TRANSPORT	0.001750778	SYT4/RGS4/CNR1/CHGA/RGS2/SYT11/GABBR1/P2RY1

GOBP_REGULATION_OF_CELL_GROWTH_INVOLVED_IN_CARDIAC_MUSCL	GOBP_REGULATION_OF_CELL_GROWTH_INVOLVED_IN_CARDIAC_MUSCLE_CELL_DEVELOPME	0.001770302	AKAP6/IGF1/PI16/RGS4/RGS2
GOBP_TELOMERASE_RNA_LOCALIZATION	GOBP_TELOMERASE_RNA_LOCALIZATION	0.001787606	NOP10/CCT6A/SHQ1/PARN/NAF1/RUVBL1/DKC1/RUVBL2/TCP1/EXOSC10/CCT8/CCT2/CCT3/CCT4/CCT7/CCT5/WRAP53/NHP2
GOBP_REGULATION_OF_PLATELET_ACTIVATION	GOBP_REGULATION_OF_PLATELET_ACTIVATION	0.001789314	CELA2A/CTSG/F2/FGG/ADAMTS18/FLNA/MMRN1/PRKG1/SELP/SERPINE2/IL6/PDGFRA/THBD/PDPN/BLK
GOBP_RETINOL_METABOLIC_PROCESS	GOBP_RETINOL_METABOLIC_PROCESS	0.001789314	PNLIP/CEL/ADH1B/ALDH1A2/TTR/CYP1B1/RDH8/ADH1A/ADH7/RBP4/HSD17B6/LRAT/ALDH1A3/CYP3A4/CYP1A1/ADH4
GOBP_KINETOCHORE_ASSEMBLY	GOBP_KINETOCHORE_ASSEMBLY	0.001795991	RNF4/CENPW/KNTC1/CENPA/CENPX/CENPK/CENPF/DLGAP5/CENPN/CENPE/CENPH
GOBP_RETROGRADE_AXONAL_TRANSPORT	GOBP_RETROGRADE_AXONAL_TRANSPORT	0.001798022	MAP1A/CALY/DLG2/KIF1A/KIF5A/TMEM108/NEFL/DST

GOBP_DIVALENT_INORGANIC_CATION_HOMEOSTASIS	GOBP_DIVALENT_INORGANIC_CATION_HOMEOSTASIS	0.001807434	ELANE/KCNA1/CASQ2/ATP1A2/PLN/CASQ1/JPH2/SCGN/ANK2/AKAP6/F2/SLC8A2/HTR2A/RGN/DMD/RYR3/HTR2B/FLNA/CACNA1C/SGCD/FGF2/TRPC4/PTH1R/TRPC1/CXCL12/HRC/PRKD1/CCL21/SYPL2/SV2A/P2RX1/ATP2B4/CCL19/MICU3/DMPK/CAV1/S100A8/EDNRA/TMEM178A/SLC30A2/SLC8A1/PKD2/BDKRB1/S100A9/PRKCB/PRKN/CDH23/SLC24A3/CCL11/ITPR1/RYR2/STC1/KL/CLIC2/THY1/FAM20A/JPH3/ANXA6/MCOLN3/ITGB3/SLC30A10
GOBP_SIGNALING_RECEPTOR_LIGAND_PRECURSOR_PROCESSING	GOBP_SIGNALING_RECEPTOR_LIGAND_PRECURSOR_PROCESSING	0.001817196	BCHE/SCG5/CTSG/PCSK1/CMA1/CPE/PCSK2/CPA3/CORIN/PCSK1N/ENPEP
GOBP_REGULATION_OF_SYSTEMIC_ARTERIAL_BLOOD_PRESSURE_BY_HORMONE	GOBP_REGULATION_OF_SYSTEMIC_ARTERIAL_BLOOD_PRESSURE_BY_HORMONE	0.001824071	AGTR1/TACR1/CTSG/CMA1/SERPINF2/AGT/CPA3/CORIN/AVPR1A/OXTR/AVPR2/ENPEP/EDNRB/EDN2/RASL10B

GOBP_RENAL_SYSTEM_PROCESS	GOBP_RENAL_SYSTEM_PROCESS	0.001836635	SPX/NPPB/AGTR1/ADIPOQ/CHRNA3/TACR1/KCNMA1/NPR3/TMEM63C/SERPINF2/AGT/CHRNB4/EDNRA/NPR1/CORIN/AVPR1A/HBB/MCAM/KIRREL1/AVPR2/HAS2/STC1/CORO2B/CHRNB2/SULF1/CD34/PRKACB/GSN/OXT/EDNRB
GOBP_PROTEIN_TRANSMEMBRANE_IMPORT_INTO_INTRACELLULAR_ORGANELLE	GOBP_PROTEIN_TRANSMEMBRANE_IMPORT_INTO_INTRACELLULAR_ORGANELLE	0.001840402	PEX6/TOMM40L/PAM16/TIMM17B/PEX26/DNAJC15/PEX16/TIMM23/TIMM23B/TIMM17A/PEX13/TIMM50/TOMM70/PEX5/CHCHD4/GRPEL2/PEX2/PEX1/AIFM1/PEX14/PEX7/TIMM21/PEX10/GRPEL1/HSPD1/TOMM40/TIMM44

GOMF_CARBOHYDRATE_DERIVATIVE_TRANSMEMBRANE_TRANSPORTER_ACTIVITY	GOMF_CARBOHYDRATE_DERIVATIVE_TRANSMEMBRANE_TRANSPORTER_ACTIVITY	0.001858876	ABCD1/SLC25A17/SLC35A1/LRRC8A/SLC35D1/SLC15A3/SLC50A1/SLC37A4/SLC25A5/SLC35B1/SLC35A2/SLC17A9/SLC35D2/SLC25A24/SLC17A5/ABCC3/SLC5A1/SLC35A4/SLC35C1/MFSD2A/SLC29A2/SLC28A3/SLC5A2/SLC35A3/SLC22A1/SLC19A1/SLC35D3/SLC37A1/SLC28A2
GOBP_POSITIVE_REGULATION_OF_FIBROBLAST_MIGRATION	GOBP_POSITIVE_REGULATION_OF_FIBROBLAST_MIGRATION	0.001863222	MIR145/ACTA2/DDR2/PAK3/AKAP12/THBS1/SLC8A1
GOBP_REGULATION_OF_LIPOPROTEIN_LIPASE_ACTIVITY	GOBP_REGULATION_OF_LIPOPROTEIN_LIPASE_ACTIVITY	0.001863222	APOA1/APOC3/APOH/GPIHBP1/APOA4
GOBP_NEGATIVE_REGULATION_OF_MUSCLE_CELL_APOPTOTIC_PROCESS	GOBP_NEGATIVE_REGULATION_OF_MUSCLE_CELL_APOPTOTIC_PROCESS	0.001858237	HAND2/APOH/MYOCD/NKX2-5/MIR145/IGF1/SFRP2
GOBP_REGULATION_OF_EXECUTION_PHASE_OF_APOP	GOBP_REGULATION_OF_EXECUTION_PHASE_OF_APOPTOSIS	0.001847832	GCG/CIDEA/PTGIS/DLC1/FAP
GOMF_STRUCTURAL_CONSTITUENT_OF_MUSCLE	GOMF_STRUCTURAL_CONSTITUENT_OF_MUSCLE	0.001863494	SYNM/MYH11/ACTN2/MYL9/TPM2/DMD/NEXN/MYL3/CSRP1/SMTN/MYOM1/TPM1

GOBP_REGENERATION	GOBP_REGENERATION	0.001844588	APOA2/VTN/FGF10/RGMA/SGCA/APOD/RGN/IGF1/GAP43/SERPINA10/MAP1B/FLNA/PTN/TNC/IGFBP1/NR4A3/CXCL12/GLI3/F7/GLI1/MYMX/HGF/MMP2/IL6/HOPX/APOA4/PRRX1/ISL1/FZD7/CCN3/SOX15/WNT10B/NEFL/TGFBR3/THY1/MIR222/PTCH1/NNMT/CPQ/FLT3/HAMP/NREP/MYOZ1/AXL

GOBP_NEGATIVE_REGULATION_OF_CELL_GROWTH	GOBP_NEGATIVE_REGULATION_OF_CELL_GROWTH	0.001863903	NPPB/SFRP1/EPHA7/FHL1/RERG/CRYAB/SLIT2/DACT3/SFRP2/PI16/SLIT3/RGS4/FRZB/GREM1/MEG3/WT1/SEMA3E/NTN1/AGT/FGF13/RGS2/NPR1/TGFB2/SERPINE2/BDKRB1/WFDC1/DAB2/SPART/CCN3/GJA1/SEMA6A/SEMA6D/SOX17/HSPA1A/INHBA/SEMA6C/SEMA3G/MAP2/SEMA5B/SEMA3D/TSPYL2/SEMA3C/DCBLD2/NRP1

GOBP_LIPID_LOCALIZATION	GOBP_LIPID_LOCALIZATION	0.001853722	PLA2G1B/PNLIP/APOA2/SPX/CEL/APOA1/APOC3/AGTR1/APOH/ADIPOQ/FABP4/CIDEA/ABCA8/CES1/APOD/APOB/ABCA9/PLA2G5/PLPPR4/ABCA6/PLA2G2C/SOAT2/TNFAIP8L3/AGT/CD36/PLA2G12B/THBS1/PLSCR4/VLDLR/CAV1/SLC27A6/PLA2G4C/SELENOM/RBP7/FABP3/RBP4/ATP8B2/ABCB1/IL6/PRKN/SERPINA5/APOA4/SHH/DAB2/MIR27B/GDF9/ABCA10/SLC10A4/RETN/CRABP2/APOLD1/ABCA1/LPL/MTTP/LRAT/ABCC2/MIR10B/INHBA/CLU/ATP8B4/SLC10A6/PTCH1/SLCO2A1/ITGB3/ACACB/PLIN2/PLTP/ENPP7/RBP1/RBP5/LRP1/FFAR2/MIR613/OXT

GOBP_RESPONSE_TO_PEPTIDE	GOBP_RESPONSE_TO_PEPTIDE	0.001851869	PLA2G1B/REG3G/AHSG/CELA2A/IRS4/AGTR1/ACTN2/ADIPOQ/NGFR/REG1B/SLC2A4/TRARG1/PCSK1/CDO1/MIR145/GLP2R/SORBS1/GRIN2A/IGF1/GHR/MAP1B/TWIST2/CYP1B1/DDR2/IGFBP1/NR4A3/IGF2/CXCL12/F7/GIP/AGT/CD36/CYP11A1/PDK4/LDOC1/PID1/SERPINF1/NKX6-1/CAV1/EDNRA/MMP3/FBN1/KLF15/MMP2/CACNA2D1/TIMP4/PRKCB/COL3A1/NR1H4/CACNA1A/RAB31/ADRB2/GLP1R/IGFBP5/TNS2/CTSK/RETN/OXTR/GJA1/FOXC2/CCN2/VIM/NEFL/SESN3/PRNP/LPL/RARRES2/KL/TGFBR3/GNAI1/GPR173/INHBA/FCGR2B/GPLD1/ITGB3/SLC30A10/SOGA1/GPER1/RHOQ/COL1A1/LRP1/GAB1/STC2/TIMP1/OXT/EDNRB/PDE3B/BACE1/RAMP3

GOMF_GROWTH_FACTOR_ACTIVITY	GOMF_GROWTH_FACTOR_ACTIVITY	0.001868638	OGN/BMP3/THBS4/FGF10/F2/IGF1/FGF7/EPGN/GDF6/EFEMP1/NGF/PTN/FGF2/IGF2/CXCL12/THPO/GDF10/AGT/TGFB3/LEFTY2/CSF3/NRG2/FGF13/HGF/NODAL/ENDOU/TGFB2/IL6/HDGFL3/DKK1/GDF9/NTF3/GDF7/CCN3/PDGFD/CCN2/FGF1/IL11/CLEC11A/INHBA/INHA/PDGFC/FGF12/VEGFC

GOBP_POSITIVE_REGULATION_OF_PROTEIN_KINASE_ACTIVITY	GOBP_POSITIVE_REGULATION_OF_PROTEIN_KINASE_ACTIVITY	0.001877806	PLA2G1B/ELANE/GCG/AZU1/CARTPT/RELN/ADIPOQ/CHRNA3/ADCYAP1/SLC8A2/HTR2A/IGF1/GHR/EPGN/FERMT2/HTR2B/ROR2/DDR2/FGF2/GREM1/DAB1/ALKAL1/IGF2/AGT/CCL21/ATP2B4/THBS1/CCL19/DYNAP/VLDLR/FZD10/FGF13/FGFR1/CCN1/ANGPT1/TGFB2/TENM1/PKD2/DKK1/ADRB2/NTF3/LRRK2/DOCK3/PDGFD/FGF1/DLG4/NEURL1/CAB39L/PRNP/BMPR1B/NOX4/PDE5A/MEF2C/FAM20A/TCL1A/S100A12/PDGFC/CLU/ITGB3/VEGFC/MMD/ROBO1/FLT3/CCR7/IL34/KIT/PDGFRB/DIRAS1
GOBP_MUSCLE_ADAPTATION	GOBP_MUSCLE_ADAPTATION	0.0018813	HAND2/MYOC/CASQ1/SGCA/MIR145/AKAP6/IGF1/PI16/RGS4/NR4A3/ACTA1/PDE9A/AGT/FBXO32/ATP2B4/RGS2/KLF15/IGFBP5

GOBP_PRIMARY_ALCOHOL_METABOLIC_PROCESS	GOBP_PRIMARY_ALCOHOL_METABOLIC_PROCESS	0.00190724	PNLIP/CEL/ADH1B/ALDH1A2/SULT2A1/IGF1/TTR/CYP1B1/CACNA1H/RDH8/SULT1C4/CYP11A1/ADH1A/ADH7/RBP4/DAB2/HSD17B6/ALDH1B1/DKK3/LRAT/ALDH1A3/CYP3A4/CYP1A1/ADH4
GOCC_INO80_TYPE_COMPLEX	GOCC_INO80_TYPE_COMPLEX	0.001918111	ACTR8/TRRAP/EP400/BRD8/ACTR5/ZNHIT1/ACTL6A/KAT5/UCHL5/INO80D/ACTR6/RUVBL1/INO80E/INO80/RUVBL2/MCRS1/SRCAP/ANP32E/NFRKB/INO80C
GOBP_NEGATIVE_REGULATION_OF_PLATELET_ACTIVATION	GOBP_NEGATIVE_REGULATION_OF_PLATELET_ACTIVATION	0.001922022	F2/FGG/ADAMTS18/PRKG1/SERPINE2/PDGFRA/THBD
GOBP_CELL_DIFFERENTIATION_INVOLVED_IN_KIDNEY_DEVELOPMENT	GOBP_CELL_DIFFERENTIATION_INVOLVED_IN_KIDNEY_DEVELOPMENT	0.00191805	ADIPOQ/ACTA2/SIX2/GREM1/WT1/GLI3/EDNRA/WTR1/KLF15/SHH/SMO/OSR1/TCF21/FOXC2/MTSS1/MEF2C/PTCH1/GLIS2/CD34/EDNRB

GOCC_TRANSPORTER_COMPLEX	GOCC_TRANSPORTER_COMPLEX	0.001920775	KCNA1/CASQ2/ATP1A2/SCN7A/GRIK3/DPP6/CHRNA3/KCNB1/PLN/KCNMA1/KCNMB1/GRIN2A/AKAP6/KCNA5/ABCC9/RYR3/GRIK5/KCNK2/GLRB/KCNIP1/ATP1B2/CACNA1C/TRPC4/CACNG4/TRPC1/BEST2/CACNA1H/SCN4B/KCNJ8/HSPA2/CACNB2/KCNH2/CHRNB4/MICU3/SCN3A/ATP8B2/PKD2/CACNA2D3/CACNA2D1/CACNA1A/CLIC4/KCNG1/KCNQ3/KCND1/CNGA1/DLG4/CPT1C/KCNS1/KCND2/RYR2/CLIC2/ATP8B4/GABRR1/CHRNB2/CACNG6/GABRE/BEST1/KCNAB1/SCN3B/SCN1B/SCN9A/CLIC5/LRRC26/MT-ND6/KCNIP2/KCNS3/PKD1/MT-ND3/CNIH2/KCNJ5/CATSPERG/CLCN1/CACNA2D2/PDE4B/KCNH4/ATP10A/KCNIP3/CNIH3/KCNN1/OSTM1/MT-ND5/MT-ND1/MT-[illegible]
GOBP_MUSCLE_HYPERTROPHY	GOBP_MUSCLE_HYPERTROPHY	0.001930303	HAND2/MYOC/MIR145/AKAP6/IGF1/HTR2B/PI16/RGS4/NR4A3/PDE9A/AGT/ATP2B4/RGS2/KLF15/IGFBP5/SORBS2/RYR2/PDE5A
GOMF_PEPTIDE_HORMONE_RECEPTOR_BINDING	GOMF_PEPTIDE_HORMONE_RECEPTOR_BINDING	0.001936131	UCN3/GHRH/VIP/ADCYAP1/GNAO1

GOCC_DNA_POLYMERASE_COMPLEX	GOCC_DNA_POLYMERASE_COMPLEX	0.001964591	POLA1/PRIM2/POLE2/POLE3/POLG/POLA2/POLD3/PRIM1/POLE/POLD2/MCM3/POLD1
GOBP_OUTFLOW_TRACT_MORPHOGENESIS	GOBP_OUTFLOW_TRACT_MORPHOGENESIS	0.001992907	HAND2/NKX2-5/SFRP2/NOG/ZFPM2/ELN/EDNRA/TGFB2/NRP2/HEYL/ISL1/PARVA/FOXC2/ILK/SOX17/TGFBR3/CLDN5/MEF2C/TWIST1/ROBO1/ACVR1/SEMA3C/NRP1/FGFR2/FZD1/SOX11/PITX2/GJA5/ENG
GOBP_MAINTENANCE_OF_LOCATION_IN_CELL	GOBP_MAINTENANCE_OF_LOCATION_IN_CELL	0.001998318	ALB/CASQ2/ATP1A2/PLN/CASQ1/JPH2/ANK2/AKAP6/F2/HTR2A/DMD/RYR3/GRIK5/HTR2B/FLNA/CACNA1C/FGF2/TRPC1/HRC/PRKD1/CCL21/DZIP1/CCL19/CAV1/S100A8/SLC30A2/SLC8A1/PKD2/SYNE1/BDKRB1/S100A9

GOBP_INTEGRIN_MEDIATED_SIGNALING_PATHWAY	GOBP_INTEGRIN_MEDIATED_SIGNALING_PATHWAY	0.002012901	APOA1/FERMT2/LIMS2/FBLN1/FLNA/ITGA7/PTN/LAMA2/PRKD1/ITGA8/ITGA9/ITGBL1/ADAMTS1/LAMA1/ITGA1/ITGA5/COL3A1/DAB2/FN1/TSPAN32/COL16A1/CCN2/ILK/BST1/NID1/THY1/DST/LAMC1/ITGB3/MPIG6B/ITGA11/NME2/PLPP3/TIMP1/NRP1

GOBP_POSITIVE_REGULATION_OF_CELL_CYCLE	GOBP_POSITIVE_REGULATION_OF_CELL_CYCLE	0.002014947	PPP1R35/VPS4B/INO80/SRC/HSF1/CALR/GPSM2/HNRNPU/SLF1/PLCG2/IL1B/SHB/ANKRD17/TGFA/CKS1B/ZNF16/AKT1/EGFR/KLHL18/TRIM21/EIF4E/YTHDC2/RANBP1/MTBP/NCAPH2/PAGR1/BRCA2/SH2B1/RAD21/FOSL1/SASS6/SMC4/TBX3/DBF4/PKN2/RRM2B/RAD23A/PSRC1/MED1/PTK6/DDX11/DDRGK1/MSX1/HCFC1/XRCC3/FBXO5/AURKB/NDC80/EIF4EBP1/KIF20B/CDC14B/SPAG5/KIF23/NKX3-1/AURKA/STOX1/RAD51AP1/KIF14/GEN1/EZH2/PHB2/TFDP1/CDC25C/DRD2/LFNG/NPM1/GIPC1/SMC2/CKS2/CDK1/CIT/MSX2/INCENP/RRM1/NCAPG2/CENPV/CHEK1/RACGAP1/SPDYC/DLGAP5/PLK4/PLRG1/RCC2/NCAPH/FEN1/CCNB1/ESPL1/TERT/FOXA1/ECT2/MAPK15/HYAL1/MAD2L1/DTL/CDC7/MD[illegible]

GOBP_VESICLE_MEDIATED_TRANSPORT_IN_SYNAPSE	GOBP_VESICLE_MEDIATED_TRANSPORT_IN_SYNAPSE	0.002019069	SYT4/CHRM2/SLC2A4/HTR2A/STON1/GRIK5/CDH2/SLC18A2/CNR1/NLGN4X/NLGN1/ADCY1/CALY/AMPH/CACNB2/SLC17A7/NLGN4Y/SV2A/P2RX1/HPCA/PRKCB/SH3GL2/SNAP25/BSN/NLGN3/LRRK2/SNCG/BRSK1/RAB3B/SYNDIG1/NLGN2/SLC18A1/APBA1/SYT1/ITGB3/PCDH17/SYP/P2RY1/ARC/AP3B2/SYT5/CADPS/BACE1/PFN2
GOBP_IRE1_MEDIATED_UNFOLDED_PROTEIN_RESPONSE	GOBP_IRE1_MEDIATED_UNFOLDED_PROTEIN_RESPONSE	0.002027557	BFAR/PARP16/UFL1/COPS5/BAX/HSPA5/DDRGK1/DAB2IP/TMEM33/BAK1/ERN2/AGR2

GOMF_N_ACYLTRANSFERASE_ACTIVITY	GOMF_N_ACYLTRANSFERASE_ACTIVITY	0.00203368	USP22/NAA60/NAA20/NAA15/PLAAT5/NAA80/CERS2/HAT1/GTF3C4/KAT5/ESCO1/NAT9/CERS6/NAA25/NAA50/NAT10/NAA10/BAAT/SRCAP/MCM3AP/BRCA2/NAA40/KAT2A/SIRT7/NAT1/SMARCE1/NAT2/ALAS1/PLAAT4/GNPNAT1/MED24/PLAAT2/ESCO2/PLA2G4E/GLYATL2
GOBP_REGULATION_OF_CELL_CYCLE_CHECKPOINT	GOBP_REGULATION_OF_CELL_CYCLE_CHECKPOINT	0.002052671	CDK5RAP2/PROX1/LCMT1/MAD2L1BP/TPR/RPA2/FBXO4/PPP1R10/ANAPC15/CUL4A/CHEK2/BRCC3/BRCA1/BARD1/BRCA2/XRCC3/AURKB/NDC80/CCAR2/RFWD3/TELO2/GEN1/TTI2/INCENP/RAD51/CCNB1/MAD2L1/BIRC5/CDCA8/WDR76/CDT1/PBK
GOBP_ACUTE_INFLAMMATORY_RESPONSE_TO_ANTIGENIC_STIMULUS	GOBP_ACUTE_INFLAMMATORY_RESPONSE_TO_ANTIGENIC_STIMULUS	0.002059585	ELANE/NPY/CNR1/A2M/CXCR2/FCGR2B/IL20RB/CCR7

GOBP_REGULATION_OF_MORPHOGENESIS_OF_A_BRANCHING_STRUCTURE	GOBP_REGULATION_OF_MORPHOGENESIS_OF_A_BRANCHING_STRUCTURE	0.002100495	SFRP1/FGF10/FGF7/AR/SIX2/NOG/GREM1/AGT/HGF/ESR1/SHH/SMO/WNT2B/LRRK2/SNAI2
GOBP_NEURON_MIGRATION	GOBP_NEURON_MIGRATION	0.00210787	RELN/ASCL1/GPM6A/FAT3/FLRT2/DNER/GFRA3/MAP1B/CCKAR/TNN/DAB1/NDN/CXCL12/SEMA3E/NTN1/PRKG1/CHL1/NKX6-1/IGSF10/MARK1/FGF13/FGFR1/NRP2/COL3A1/SPOCK1/ATOH1/SEMA6A/LHX6/SEPTIN4/MEF2C/NRCAM/GPR173/TWIST1/PHACTR1/DCLK1/CXCR4/ADGRL3/NDNF/LRP12/AXL/NRP1

GOBP_GLIAL_CELL_DEVELOPMENT	GOBP_GLIAL_CELL_DEVELOPMENT	0.002115739	NKX2-2/SOX10/PLP1/MYOC/ASCL1/ROR2/CNTN1/WASF3/LGI4/CNTNAP1/MAPT/S100A8/LPAR1/MAL/ASPA/IL6/S100A9/TSPAN2/SHH/SMO/ID4/VIM/POU3F1/ILK/TENM4/MXRA8/CLU/PRDM8/ROR1/LRP1
GOCC_NEURON_PROJECTION_TERMINUS	GOCC_NEURON_PROJECTION_TERMINUS	0.002120353	UCN3/KCNA1/GHRH/PENK/CHRM2/SLC18A3/SCRG1/GRIK3/PCSK1/SCGN/SLC8A2/DMD/PTPRN/GRIK5/KCNK2/NPY/TNN/SLC18A2/DIXDC1/SLC8A1/PRKCB/FLRT1/BSN/UCHL1/LRRK2/SNCG/DLG4/PRNP/SLC18A1/SYT1/SYP/GPER1/ROR1

GOCC_INTRINSIC_COMPONENT_OF_ENDOPLASMIC_RETICULUM_MEMBRANE	GOCC_INTRINSIC_COMPONENT_OF_ENDOPLASMIC_RETICULUM_MEMBRANE	0.002127709	ACER3/SARAF/HLA-DPB1/PDIA3/PIGS/SGMS1/HACD2/BFAR/HM13/EBP/SELENOS/GRAMD2A/INSIG1/DPAGT1/DNAJB12/EMC1/AUP1/BNIP1/SLC37A4/ARL6IP1/SYVN1/PREB/HLA-H/SPPL2B/TAPT1/TAPBP/CALR/EMC8/CCDC47/HSPA5/HLA-DPA1/SLC35B1/CANX/SEC61A1/DERL3/HLA-E/TBL2/ELOVL3/SREBF2/PIGG/BCAP31/HLA-DRA/DOLPP1/HLA-B/HLA-A/RCE1/HLA-DQA2/ELOVL1/HLA-DQA1/TECR/FITM1/DHCR7/CYP2E1/SLC27A5/HLA-DRB5/EDEM1/ATP13A1/CD74/HLA-F/HLA-C/SGPL1/TMEM33/HACD3/TAP2/SCD/ZFYVE27/HLA-DRB1/EMC6/ELOVL6/HLA-DQB1/HLA-DQB2/SLC27A2/SLC37A1/[illegible]
GOBP_ENSHEATHMENT_OF_NEURONS	GOBP_ENSHEATHMENT_OF_NEURONS	0.002132829	SOX10/PLP1/MPZ/MYOC/ANK2/NFASC/CNTN1/PTN/JAM2/CLDN11/JAM3/WASF3/LGI4/CNTNAP1/RXRG/LPAR1/HGF/MAL/ASPA/TSPAN2/DHH/ID4/POU3F1/EPB41L3/ILK/TENM4/PTPRZ1/QKI/CLDN5/PMP22/CLU/CXCR4/MARVELD1

GOCC_POSTSYNAPTIC_DENSITY_MEMBRANE	GOCC_POSTSYNAPTIC_DENSITY_MEMBRANE	0.002151928	EPHA7/GRIN2A/GRIK5/PLPPR4/CACNA1C/CACNG4/NLGN4X/ADCY1/NLGN4Y/DLG2/RGS7BP/LIN7A/SORCS2/CLSTN2/PRRT1/EFNB3/LRRC4/DLG4/LRRC4C/SYNDIG1/NRCAM
GOBP_ATTACHMENT_OF_MITOTIC_SPINDLE_MICROTUBULES_TO_KINETOCHORE	GOBP_ATTACHMENT_OF_MITOTIC_SPINDLE_MICROTUBULES_TO_KINETOCHORE	0.002168601	SEH1L/KAT5/HNRNPU/NUF2/AURKB/NDC80/INCENP/KIF2C/CENPE/BIRC5/CDCA8/CDT1
GOBP_CELL_MIGRATION_INVOLVED_IN_SPROUTING_ANGIOGENESIS	GOBP_CELL_MIGRATION_INVOLVED_IN_SPROUTING_ANGIOGENESIS	0.002176109	MIR483/MEOX2/SLIT2/FGF2/GREM1/AKT3/THBS1/RHOJ/MIR27B/FOXC2/MIR149/SRPX2/EGR3/MIR10B/GPLD1/MMRN2/ROBO1/MIR196A1/STARD13/CLEC14A/NRP1/TBXA2R/HDAC5

GOBP_STEM_CELL_DIFFERENTIATION	GOBP_STEM_CELL_DIFFERENTIATION	0.002181902	SOX10/SFRP1/HAND2/MYOCD/NKX2-5/GPM6A/ALDH1A2/SFRP2/RBM24/NOG/HTR2B/CDH2/NAP1L2/PTN/FGF2/FRZB/GREM1/TRIM6/SEMA3E/PRICKLE1/RADIL/EDNRA/SOX5/TGFB2/NRP2/SHC4/ESR1/SHH/SMO/PDGFRA/OSR1/FN1/A2M/ISL1/MEOX1/SNAI2/FOXC2/SEMA6A/HOXD4/SEMA6D/TCF15/SOX17/MEF2C/LTBP3/ANXA6/TWIST1/SEMA6C/TBX5/SEMA3G/SEMA5B/SEMA3D/KIT/ACVR1/SEMA3C/EDNRB/NRP1
GOBP_REGULATION_OF_RESPONSE_TO_EXTRACELLULAR_STIMULUS	GOBP_REGULATION_OF_RESPONSE_TO_EXTRACELLULAR_STIMULUS	0.002197108	SPX/CARTPT/NPY/MN1/KANK2/NR1H4/SNAI2

GOBP_NEGATIVE_REGULATION_OF_VASCULATURE_DEVELOPMENT	GOBP_NEGATIVE_REGULATION_OF_VASCULATURE_DEVELOPMENT	0.002196336	NPPB/APOH/THBS4/ANGPTL7/MIR145/ISM1/ADGRB3/DCN/SEMA3E/AGT/ATP2B4/ADAMTS1/THBS1/SERPINF1/NPR1/TGFB2/SERPINE1/EMILIN1/SEMA6A/CLDN5/TEK/MIR222/TAFA5/SPARC/ADAMTS9/PTPRM/THBS2/ADGRB2/ADGRB1/HOXA5/SULF1/ECSCR/PDE3B/TIE1/COL4A2/ANGPT2
GOBP_GLAND_MORPHOGENESIS	GOBP_GLAND_MORPHOGENESIS	0.002203529	FGL1/SFRP1/FGF10/PGR/FGF7/AR/NOG/LIMS2/PTN/TNC/GLI3/NTN1/TGFB3/LAMA1/GLI1/CAV1/HGF/CYP7B1/TGFB2/MMP2/IL6/ESR1/SHH/IGFBP5/GDF7/ID4/SNAI2/CCL11/FGF1/EDA/PTCH1/NR3C1

GOBP_REGULATION_OF_CENTRIOLE_REPLICATION	GOBP_REGULATION_OF_CENTRIOLE_REPLICATION	0.002218673	ALMS1/NUP62/CEP295/BRCA1/PPP1R35/VPS4B/RBM14/SASS6/KAT2A/NPM1/PLK2/PLK4/STIL
GOBP_NEURON_RECOGNITION	GOBP_NEURON_RECOGNITION	0.002235905	GAP43/NEXN/EPHA3/CRTAC1/TNN/CNR1/NDN/NCAM2/CNTN4/VSTM2L/PALLD/EFNB3/NRCAM/NTM/ROBO1/CXCR4/NRP1
GOMF_ACYLTRANSFERASE_ACTIVITY	GOMF_ACYLTRANSFERASE_ACTIVITY	0.002238236	LCLAT1/PIGW/ZDHHC18/GTF3C4/KAT5/ESCO1/SCP2/YKT6/CPT1A/ZDHHC5/LPCAT3/CLYBL/SPTLC1/DLST/MBOAT7/NAT9/CERS6/ZDHHC13/ABHD14B/NAA25/CBR4/NAA50/GPAT3/AGPAT3/ZDHHC16/MOGAT2/NAT10/ZDHHC23/NAA10/ACSM1/ZDHHC11/BAAT/SRCAP/MCM3AP/GPAT4/ELOVL3/BRCA2/ELP3/PAFAH2/ASPG/NAA40/CS/KAT2A/ACAA1/QPCTL/ZDHHC12/TMEM68/OXSM/ZDHHC3/ELOVL1/PNPLA4/ZDHHC20/GGT6/SIRT7/SPTLC2/CPT2/SIRT6/CRLS1/MBOAT2/NAT1/MCAT/SMARCE1/NAT2/DGAT1/ALAS1/PAFAH1B3/ACLY/ACAT2/DLAT/LPCAT1/MBOAT1/GCAT/SPTSSB/ELOVL6/FASN/ACSM3/AGPAT2/PLAAT4/GNPNAT1/MED24/AGPAT5/PLAAT2/ESCO2/LPCAT4/CIITA/PLA2G4A/PLA2G4E/GLYATL2

GOBP_REGULATION_OF_SYSTEMIC_ARTERIAL_BLOOD_PRESSURE_BY_RENIN_ANGIOTENS	GOBP_REGULATION_OF_SYSTEMIC_ARTERIAL_BLOOD_PRESSURE_BY_RENIN_ANGIOTENSIN	0.002242063	AGTR1/TACR1/CTSG/CMA1/SERPINF2/AGT/CPA3
GOBP_NEGATIVE_REGULATION_OF_SMOOTH_MUSCLE_CELL_MIGRATION	GOBP_NEGATIVE_REGULATION_OF_SMOOTH_MUSCLE_CELL_MIGRATION	0.002258039	ADIPOQ/MYOCD/SLIT2/TPM1/PRKG1/SERPINE1/IGFBP5/SEMA6D/MEF2C/TAFA5/LRP1/NDRG4
GOMF_3_5_CYCLIC_AMP_PHOSPHODIESTERASE_ACTIVITY	GOMF_3_5_CYCLIC_AMP_PHOSPHODIESTERASE_ACTIVITY	0.002256589	PDE7B/PDE1A/PDE3A/PDE2A/PDE1C/PDE1B/PDE8B
GOBP_GLYCOLYTIC_PROCESS_THROUGH_FRUCTOSE_6_PHOSPHATE	GOBP_GLYCOLYTIC_PROCESS_THROUGH_FRUCTOSE_6_PHOSPHATE	0.002268218	ENO2/FOXK2/FOXK1/ADPGK/HK3/PGK1/PFKL/TPI1/ENO1/PFKP/PKM/HK2
GOMF_RNA_POLYMERASE_CORE_ENZYME_BINDING	GOMF_RNA_POLYMERASE_CORE_ENZYME_BINDING	0.00228074	RPRD2/RTF1/PCIF1/ELP2/PCF11/ZNF326/SCAF8/RPRD1B/AGO1/GTF2B/RPRD1A/CDC73/MAF1/BRD4/RPAP2/RECQL5/SMYD2/SCAF1/SCAF4/CTR9/LEO1/WDR43/DHX9/UVSSA/RRN3/HNRNPU/SPTY2D1/ELOF1/ERCC5/AGO2/CCAR2

GOBP_SENSORY_PERCEPTION_OF_CHEMICAL_STIMULUS	GOBP_SENSORY_PERCEPTION_OF_CHEMICAL_STIMULUS	0.00228075	PIGR/GJB4/TRPM5/OMP/CALHM3/OR2I1P/SCNN1G/TAS1R3/SLC6A3/GNG13/LCN1/OR8K5
GOBP_CALCIUM_ION_IMPORT_ACROSS_PLASMA_MEMBRANE	GOBP_CALCIUM_ION_IMPORT_ACROSS_PLASMA_MEMBRANE	0.002285184	SLC8A2/CACNA1C/P2RX1/ATP2B4/SLC8A1/CACNA2D1/CACNA1A/PRNP
GOCC_PRERIBOSOME_LARGE_SUBUNIT_PRECURSOR	GOCC_PRERIBOSOME_LARGE_SUBUNIT_PRECURSOR	0.002292016	MAK16/NSA2/LAS1L/WDR12/FTSJ3/MDN1/NIP7/RRP1B/WDR74/EBNA1BP2/PES1/RRP1/RRS1/MRTO4/BOP1/NOC2L/PPAN
GOBP_AMMONIUM_ION_METABOLIC_PROCESS	GOBP_AMMONIUM_ION_METABOLIC_PROCESS	0.002300109	BCHE/TPH1/GRIN2A/RNF180/PDE1B/HDC/SLC29A4
GOBP_REGULATION_OF_ACTION_POTENTIAL	GOBP_REGULATION_OF_ACTION_POTENTIAL	0.002297987	KCNB1/TACR1/CTNNA3/ANK2/SLC8A2/FLNA/CACNA1C/CNR1/CD36/CAV1/CNR2/FGF13/TBX18/CACNA2D1

GOBP_EPITHELIAL_TO_MESENCHYMAL_TRANSITION	GOBP_EPITHELIAL_TO_MESENCHYMAL_TRANSITION	0.002295096	SFRP1/TMEM100/DACT3/IGF1/SFRP2/NOG/FERMT2/EPHA3/FLNA/PHLDB2/GREM1/TGFB1I1/TGFB3/EDNRA/MARK1/CRB2/HGF/FGFR1/TGFB2/WWTR1/HEYL/IL6/ZNF750/DAB2/FOXF2/ISL1/FAM83D/SNAI2/MIR149/HAS2/PDPN/PHLDB1/TGFBR3/MIR222/LDLRAD4/TWIST1/TBX5/VASN/LEF1/COL1A1/ACVR1/SPRED3/BMP5/FGFR2/FUZ/ENG/SNAI1/GLIPR2/SERPINB3/RFLNB/SPRY1/WNT16/TGFBR1/GATA3/HMGA2/TIAM1/RGCC/MIR221/RTN4/EPHA4/ACVRL1
GOBP_REGULATION_OF_FIBROBLAST_PROLIFERATION	GOBP_REGULATION_OF_FIBROBLAST_PROLIFERATION	0.002309514	PLA2G1B/SFRP1/NGFR/FGF10/IGF1/DDR2/AGT/CAV1/ESR1/PDGFRA/FN1/PDGFD/PDGFC/ITGB3/CD248/PDGFRB
GOBP_IMMUNE_RESPONSE_TO_TUMOR_CELL	GOBP_IMMUNE_RESPONSE_TO_TUMOR_CELL	0.002328688	NECTIN2/IL4I1/HLA-A/HSPD1/PVR/NKG7/HLA-DRB1/ADAM15/PRF1/CD274

GOBP_CYCLIC_NUCLEOTIDE_BIOSYNTHETIC_PROCESS	GOBP_CYCLIC_NUCLEOTIDE_BIOSYNTHETIC_PROCESS	0.002325809	NPPB/ADCY5/ADCY2/ADCY1/GUCY1A1/GUCY1B1/NPR1/GUCY1A2/NPR2/NPPC/ADCY4
GOBP_PROTEIN_MATURATION	GOBP_PROTEIN_MATURATION	0.00232634	HP/BCHE/APOH/NNAT/SCG5/FGA/NLRP7/CTSG/PCSK1/CMA1/GRIN2A/CPXM2/CPE/FGG/FBLN1/FLNA/SPON1/GAS1/PCSK2/CPN1/GLI3/F7/F13A1/SERPINF2/THBS1/TLL1/CPA3/NAA11/ADAMTS3/SERPINE2/BAG2/COMP/CORIN/SERPINE1/SHH/FN1/DHH/CCBE1/CLEC3B/MMP16/CPXM1/F8/TSPAN32/LRRK2/PCSK1N/C1R/ENPEP/THBD/PRNP/AEBP1/FGB/MAGEA3/PTCH1/LDLRAD3/DYNC2H1
GOBP_POSITIVE_REGULATION_OF_SMOOTH_MUSCLE_CELL_MIGRATION	GOBP_POSITIVE_REGULATION_OF_SMOOTH_MUSCLE_CELL_MIGRATION	0.002346669	VTN/IGF1/CYP1B1/DDR2/NR4A3/AGT/ADAMTS1/LPAR1/IGFBP5/PDGFD/CCN4/SEMA6D/HAS2/ITGB3/PDGFRB/NRP1

GOBP_NEUROTRANSMITTER_SECRETION	GOBP_NEUROTRANSMITTER_SECRETION	0.002344773	SYT4/CHRM2/TACR2/CHRNA3/NRXN2/HTR2A/GRIK5/CNR1/NLGN1/ADCY1/CACNB2/CHRNB4/SV2A/P2RX1/LIN7A/PRKCB/PRKN/SNAP25/SYT11/LRRK2/SNCG/BRSK1/MEF2C/APBA1/SYT1/SYP/P2RY1/GPER1/SYT5/CADPS/SNCAIP/BACE1/PFN2
GOMF_TELOMERASE_RNA_BINDING	GOMF_TELOMERASE_RNA_BINDING	0.002366552	DCP2/SNRPD3/TEP1/SMG6/NOP10/TENT4B/SMG7/PARN/SMG5/NAF1/XRN1/SNRPB/DKC1/HNRNPU/HNRNPC/EXOSC10/PINX1/GAR1/WRAP53/NHP2/TERT
GOBP_LEUKOCYTE_MIGRATION_INVOLVED_IN_INFLAMMATORY_RESPONSE	GOBP_LEUKOCYTE_MIGRATION_INVOLVED_IN_INFLAMMATORY_RESPONSE	0.0023647	ELANE/PTN/JAM3/S100A8/S100A9/SELE/FFAR2
GOCC_ENDOCYTIC_VESICLE_LUMEN	GOCC_ENDOCYTIC_VESICLE_LUMEN	0.002370171	MPO/HP/APOA1/APOB/CSF3/HBB/PGLYRP1/SCGB3A2/SPARC

GOBP_MATURATION_OF_LSU_RRNA	GOBP_MATURATION_OF_LSU_RRNA	0.0023837	SNU13/RPF1/GTPBP4/NOL9/NIFK/DDX18/EIF6/RPL10A/ZNHIT3/MAK16/RPF2/NSA2/LAS1L/WDR12/FTSJ3/RSL1D1/RPL7A/RPL35/RPL7/RPL7L1/MRPL1/PES1/URB1/NHP2/BOP1
GOBP_EMBRYONIC_ORGAN_MORPHOGENESIS	GOBP_EMBRYONIC_ORGAN_MORPHOGENESIS	0.002392414	HAND1/COL2A1/HAND2/NEUROD1/NKX2-5/RBPMS2/FGF10/ASB2/SIX2/NOG/EFEMP1/NKX3-2/FRZB/HOXD10/GLI3/ZEB1/MFAP5/NTN1/ITGA8/FOXF1/TGFB3/HOXA4/GLI1/SOBP/EDNRA/CRB2/NODAL/TBX18/FBN1/RBP4/PKD2/CDH23/HOXA2/SHH/SMO/PDGFRA/OSR1/MMP16/PRRX1/SLITRK6/FOXF2/TCF21/FOXC2/ATOH1/HOXD4/TBX15/SOX17/USH1G/RYR2/MEF2C/ALDH1A3/MFAP2/TWIST1/HOXA5/CTHRC1/HOXD9/VANGL2/ACVR1

GOBP_REGULATION_OF_MITOCHONDRIAL_MEMBRANE_PERMEABILITY	GOBP_REGULATION_OF_MITOCHONDRIAL_MEMBRANE_PERMEABILITY	0.002400082	BID/BAD/BCL2L1/SLC35F6/GSK3A/HEBP2/RTL10/ALKBH7/BAX/SLC25A5/RHOT2/ATP5IF1/ZNF205/HIP1R/PPIF/CHCHD10/VDAC2/PMAIP1/TP53/BAK1/TMEM102/BBC3/HK2/SLC9A1/GZMB
GOBP_CYTOKINETIC_PROCESS	GOBP_CYTOKINETIC_PROCESS	0.002400149	ARF1/CHMP6/ALKBH4/CHMP2B/NUP62/PDCD6IP/RTKN/CHMP7/CHMP1A/VPS4B/CHMP5/PLEC/SPIRE2/KIF20A/ANXA11/AURKB/KIF20B/BIN3/CNTROB/ANLN/RACGAP1/CHMP4C/ECT2/CEP55
GOBP_REGULATION_OF_RECEPTOR_MEDIATED_ENDOCYTOSIS	GOBP_REGULATION_OF_RECEPTOR_MEDIATED_ENDOCYTOSIS	0.002416234	APOC3/ADIPOQ/VTN/RSPO1/TF/GREM1/SFRP4/CCL21/CCL19/ANGPT1/HPCA/SERPINE1/DAB2/DKK1/SGIP1/MIR27B/NTF3/DLG4/USH1G/CLU/ITGB3/SELE/ARC

GOBP_AMP_METABOLIC_PROCESS	GOBP_AMP_METABOLIC_PROCESS	0.002433497	APRT/ADK/GART/NUDT2/ATIC/ADSS1/AK1/AK2/PFAS/XDH/PRPS2/PPAT/PAICS/ADA
GOBP_REGULATION_OF_BODY_FLUID_LEVELS	GOBP_REGULATION_OF_BODY_FLUID_LEVELS	0.002435555	CSN2/CELA2A/CEL/VIP/NPPB/APOH/VTN/FGA/TPH1/TACR1/CTSG/FGF10/SERPIND1/MYL9/CDO1/NPR3/F2/SCT/FGG/SERPINA10/ADAMTS18/FBLN1/FLNA/CSRP1/TFPI2/F7/F13A1/SERPINF2/MMRN1/CD36/PRKG1/DGKG/P2RX1/THBS1/CAV1/SELP/NPR1/SERPINE2/COMP/IL6/COL3A1/SERPINE1/SHH/HBB/EMILIN1/PDGFRA/PROS1/FN1/KRT1/F8/TSPAN32/ACER1/OXTR/NEURL1/AVPR2/THBD/ILK/HAS2/SCUBE1/FAP/HEG1/PDPN/HSPB1/FGB/ACTN1/SERPING1/TFPI/BLK/F10/CORO2B/ITGB3/MPIG6B/P2RY1/CD34/IL6ST/PRKACB/OXT/AXL/EDNRB/TREML1/APLNR/MERTK/PRLR/ENTPD1
GOCC_GLIAL_CELL_PROJECTION	GOCC_GLIAL_CELL_PROJECTION	0.002454736	SYT4/SCN7A/NFASC/KCNK2/ATP1B2/WASF3/MAPT

GOCC_ANCHORED_COMPONENT_OF_MEMBRANE	GOCC_ANCHORED_COMPONENT_OF_MEMBRANE	0.00245439	LY6L/LYPD8/RGMA/NEGR1/GFRA1/GFRA3/GPC3/PRND/CNTN1/GAS1/GFRA2/DPEP3/GPIHBP1/CNTN4/RECK/RGS7BP/GP2/NCAM1/XPNPEP2/ITLN1/LSAMP/GPC6/FOLR2/DPEP1/RGMB/CA4/RHBG/DLG4/RAB3B/PRNP/RAET1G/BST1/THY1/LYNX1/TFPI/RAB3C/NTM
GOBP_CELL_CELL_SIGNALING_INVOLVED_IN_CARDIAC_CONDUCTION	GOBP_CELL_CELL_SIGNALING_INVOLVED_IN_CARDIAC_CONDUCTION	0.002458109	CASQ2/ANK2/KCNA5/FLNA/CACNA1C/SCN4B/CACNB2/GJC1/TBX18/CACNA2D1/GJA1/RYR2/TBX5/SCN3B/SCN1B/GJA5/KCNJ5

GOMF_CHANNEL_REGULATOR_ACTIVITY	GOMF_CHANNEL_REGULATOR_ACTIVITY	0.002481126	DPP6/KCNMB1/NRXN2/ABCC9/NPY/KCNIP1/FLNA/CACNG4/SCN4B/FXYD6/AMBP/KCNE4/PRKG1/ATP2B4/CAV1/GEM/FGF13/PRKCB/ADRB2/REM1/KCNS1/ITPR1/RRAD/FGF12/GPLD1/CACNG6/KCNAB1/SCN3B/SCN1B/SNTA1/LRRC26/AMIGO1/KCNIP2/NEDD4/KCNS3
GOBP_LIVER_MORPHOGENESIS	GOBP_LIVER_MORPHOGENESIS	0.002487095	FGL1/LIMS2/PTN/GLI1/IL6/FGF1
GOBP_VENTRAL_SPINAL_CORD_DEVELOPMENT	GOBP_VENTRAL_SPINAL_CORD_DEVELOPMENT	0.002545312	NKX2-2/RELN/ASCL1/DAB1/HOXD10/GLI3/VLDLR/SHH/ISL1/PTCH1/DYNC2H1/GLI2
GOBP_REGULATION_OF_HEART_GROWTH	GOBP_REGULATION_OF_HEART_GROWTH	0.002545911	NKX2-5/AKAP6/IGF1/NOG/KCNK2/PI16/RGS4/ZFPM2/FGF2/WT1/GLI1/RGS2/RBP4/DUSP9/TGFBR3/MEF2C/MIR222/ACACB/TBX5

GOBP_GLAND_DEVELOPMENT	GOBP_GLAND_DEVELOPMENT	0.00254455	FGL1/CSN2/GHRH/SOX10/APOA1/SFRP1/HAND2/VTN/TPH1/NKX2-5/FGF10/PCSK1/ASCL1/CDO1/ALDH1A2/RGN/PGR/FGF7/INSM1/AR/SERPINA10/NOG/LIMS2/CYP1B1/PTN/FGF2/TNC/FRZB/IGF2/WT1/GLI3/NTN1/FOXF1/TGFB3/LAMA1/GLI1/SERPINF1/CAV1/EDNRA/HGF/NODAL/CYP7B1/TGFB2/SERPINE2/UGT1A7/PKD2/MMP2/ONECUT1/IL6/ESR1/SHH/SMO/PDGFRA/PBX1/CGA/CCKBR/ISL1/IGFBP5/GDF7/ACER1/TCF21/OXTR/ID4/SNAI2/CCL11/FGF1/NEURL1/DKK3
GOBP_CYTOPLASMIC_PATTERN_RECOGNITION_RECEPTOR_SIGNALING_PATHWAY_IN_RESPONSE_TO_VIRUS	GOBP_CYTOPLASMIC_PATTERN_RECOGNITION_RECEPTOR_SIGNALING_PATHWAY_IN_RESPONSE_TO_VIRUS	0.002566185	USP15/BIRC2/NLRX1/CLPB/PUM2/PUM1/NPLOC4/UFD1/RIOK3/IFIH1/IRF7/GPATCH3/DHX58/IRF3/ANKRD17/DDX60/OAS3/TKFC/PHB2/OASL/TRIM25/BIRC3/C1QBP/TRIM15

GOBP_SODIUM_ION_HOMEOSTASIS	GOBP_SODIUM_ION_HOMEOSTASIS	0.002571903	SPX/NPPB/ATP1A2/AGTR1/SCN7A/TACR1/ATP1B2/AGT/EDNRA/NPR1/SLC8A1/CORIN/AVPR1A/UPK3A/AVPR2
GOMF_CYTOKINE_ACTIVITY	GOMF_CYTOKINE_ACTIVITY	0.002570821	CCL16/CER1/BMP3/ADIPOQ/GDF6/CCL26/GREM2/VSTM1/IL24/FGF2/C1QTNF4/GREM1/CXCL6/ALKAL1/CXCL12/THPO/GDF10/IL33/WNT9A/CCL21/TGFB3/LEFTY2/TSLP/CCL19/CSF3/NDP/C10orf99/NODAL/TGFB2/IL17B/TNFRSF11B/IL6/CTF1/CCL2/GDF9/CRLF1/GDF7/WNT2B/WNT10B/SLURP1/CCL11/IL11/SCG2/TNFSF12/TAFA5/INHBA/INHA

GOBP_REGULATION_OF_PHOSPHATIDYLINOSITOL_3_KINASE_SIGNALING	GOBP_REGULATION_OF_PHOSPHATIDYLINOSITOL_3_KINASE_SIGNALING	0.002578666	RELN/MYOC/F2/IGF1/ROR2/FGF2/DCN/SEMA3E/AGT/CSF3/SELP/HGF/FGFR1/ANGPT1/TGFB2/SERPINE2/PDGFRA/PTPN13/FN1/PDGFD/FBXL2/CRNN/TEK/PDGFC/TWIST1/FLT3/GPER1/ROR1/KIT/PDGFRB
GOBP_ARTERY_MORPHOGENESIS	GOBP_ARTERY_MORPHOGENESIS	0.002584359	HAND2/MYLK/MYOCD/MIR145/APOB/NOG/FKBP10/FOXF1/AKT3/EDNRA/TGFB2/PKD2/COMP/COL3A1/PRRX1/EFEMP2/FOXC2
GOBP_REGULATION_OF_BMP_SIGNALING_PATHWAY	GOBP_REGULATION_OF_BMP_SIGNALING_PATHWAY	0.002587176	CER1/SFRP1/CHRDL1/RBPMS2/RGMA/CHRDL2/SFRP2/NOG/GREM2/GPC3/BMPER/GREM1/SFRP4/CHRD/ZNF423/FSTL1/CRB2/CCN1/FBN1/DKK1/SPART

GOBP_COLLAGEN_BIOSYNTHETIC_PROCESS	GOBP_COLLAGEN_BIOSYNTHETIC_PROCESS	0.002591592	MIR145/F2/DDR2/SERPINF2/TGFB3/ADAMTS3/LARP6/IL6/EMILIN1/CCN2/VIM/MIR149/PCOLCE/INHBA/COL1A1/PDGFRB/COL5A1/NPPC/RCN3/ENG/CYGB/F2R/RGCC/P3H3/SCX/P3H4
GOCC_HISTONE_METHYLTRANSFERASE_COMPLEX	GOCC_HISTONE_METHYLTRANSFERASE_COMPLEX	0.002604549	NCOA6/KANSL1/TAF6/WDR82/KDM6A/HDAC9/CHD8/TAF1/BOD1/KDM6B/TAF4/SETD1B/E2F6/RBBP7/TAF9/MAX/ASH2L/SUZ12/SENP3/RBBP5/PRPF31/AEBP2/TEX10/PELP1/PRDM4/LAS1L/EED/HDAC2/KMT2B/SETD1A/RUVBL1/RUVBL2/KMT2D/MEN1/MCRS1/PAXIP1/RBBP4/PAGR1/WDR5/INO80C/HCFC1/CXXC1/EZH2/PRMT5

GOBP_CYTOCHROME_COMPLEX_ASSEMBLY	GOBP_CYTOCHROME_COMPLEX_ASSEMBLY	0.002616146	FASTKD3/COA1/UQCC2/COX16/COA8/COX19/C12orf73/SLC25A46/TMEM223/COA6/SMIM20/PET100/COA4/HCCS/UQCRFS1/BCS1L/TACO1/SCO1/SLC25A33/UQCC3/TIMM21/COX15/COX10/CYBA/TTC19/COX18/SCO2
GOMF_DNA_POLYMERASE_ACTIVITY	GOMF_DNA_POLYMERASE_ACTIVITY	0.002628177	TEP1/CHRAC1/TENT4B/TERF2/POLA1/POLL/POLH/POLM/POLE2/POLB/DKC1/PTGES3/POLE3/POLG/POLD3/POLE/POLQ/TERT/POLD1
GOBP_WATER_HOMEOSTASIS	GOBP_WATER_HOMEOSTASIS	0.002635824	CLDN4/SCNN1B/LSR/PLEC/FLG/KLF4/ADCY6/STMN1/CYP26B1/ELOVL1/IL18/CYP4F12/SFN/KDF1/MYO5B/FA2H/ALOX12B/SCNN1G/ABCA12/KRT16/BPIFA1/AQP2

GOBP_ENDOPLASMIC_RETICULUM_UNFOLDED_PROTEIN_RESPONSE	GOBP_ENDOPLASMIC_RETICULUM_UNFOLDED_PROTEIN_RESPONSE	0.002641867	CREBZF/EP300/CREB3L4/BCL2L11/DERL2/ERN1/VAPB/ATF6B/AMFR/TMED2/QRICH1/DNAJC10/EIF2AK3/PPP1R15B/RHBDD2/DERL1/SERP1/CDK5RAP3/STUB1/CREB3/OS9/BFAR/SELENOS/RACK1/PARP16/UFL1/YOD1/COPS5/BAX/HSPA5/DERL3/EIF2S1/TBL2/DDRGK1/VCP/DAB2IP/TMEM33/CREB3L1/ERO1A/ABCA7/BAK1/ERMP1/CCND1/ERN2/AGR2/CTH
GOBP_NEUROEPITHELIAL_CELL_DIFFERENTIATION	GOBP_NEUROEPITHELIAL_CELL_DIFFERENTIATION	0.002645389	NKX2-2/ASCL1/INSM1/CDH2/MAP1B/FGF2/NODAL/SERPINE1
GOBP_NEGATIVE_REGULATION_OF_LIPID_CATABOLIC_PROCESS	GOBP_NEGATIVE_REGULATION_OF_LIPID_CATABOLIC_PROCESS	0.002659332	APOA2/APOC3/CIDEA/CNR1/HCAR1/GPLD1/ACACB/PDE3B

GOMF_PEPTIDE_ANTIGEN_BINDING	GOMF_PEPTIDE_ANTIGEN_BINDING	0.002668321	HLA-DPB1/MAML1/TRAV12-3/HLA-H/TAPBP/HLA-DPA1/HLA-E/HLA-DRA/HLA-B/HLA-A/TRAV23DV6/HLA-DQA1/HLA-DRB5/DHCR24/HLA-F/HLA-C/TAP2/HLA-DRB1/SLC7A5/HLA-DQB1/TAP1
GOBP_REGULATION_OF_CARDIOCYTE_DIFFERENTIATION	GOBP_REGULATION_OF_CARDIOCYTE_DIFFERENTIATION	0.002665607	MYOCD/NKX2-5/MIR145/PRICKLE1/TGFB2/DKK1/FZD7/SOX17/MEF2C/MIR222/TBX5/GPER1
GOBP_POSITIVE_REGULATION_OF_GLUCOSE_TRANSMEMBRANE_TRANSPORT	GOBP_POSITIVE_REGULATION_OF_GLUCOSE_TRANSMEMBRANE_TRANSPORT	0.00267194	ADIPOQ/SORBS1/IGF1/GPC3/CLIP3/NR4A3/GIP/ITLN1/KLF15
GOMF_BMP_BINDING	GOMF_BMP_BINDING	0.002675076	CER1/CHRDL1/CHRDL2/GREM2/GREM1/CHRD/COMP/BMPR1B/SCUBE3

GOBP_POSITIVE_REGULATION_OF_DOUBLE_STRAND_BREAK_REPAIR_VIA_HOMOLOGOUS_RECOMBINATION	GOBP_POSITIVE_REGULATION_OF_DOUBLE_STRAND_BREAK_REPAIR_VIA_HOMOLOGOUS_RECOMBINATION	0.002687194	ING3/ARID2/ACTR2/ERCC6/YEATS4/TRRAP/WAS/EP400/BRD8/MRGBP/PIAS4/ACTL6A/KAT5/HDGFL2/MMS19/FUS/RUVBL1/RUVBL2/FANCB/PARP1/RBBP8/WRAP53/RAD51AP1/TIMELESS
GOBP_REGULATION_OF_SYNAPSE_ASSEMBLY	GOBP_REGULATION_OF_SYNAPSE_ASSEMBLY	0.002705298	EPHA7/NEGR1/FLRT2/ADGRB3/NLGN1/PTPRD/NPTX1/NTN1/LRRN1/CLSTN2/DKK1/GPC6/SLITRK6/OXTR/NLGN3/SRPX2/SYNDIG1/COLQ/MEF2C/NLGN2/THBS2/CHRNB2/ADGRB1/MUSK/AMIGO2/OXT/LRRC4B/FZD1/AMIGO1/NTNG2/GHRL/CLSTN3/NTRK2/SNCA/TLR2
GOBP_NEGATIVE_REGULATION_OF_CALCIUM_ION_TRANSMEMBRANE_TRANSPORTER_ACTI	GOBP_NEGATIVE_REGULATION_OF_CALCIUM_ION_TRANSMEMBRANE_TRANSPORTER_ACTIVITY	0.002721822	CASQ2/ATP1A2/PLN/GEM/PKD2/REM1/SLN/RRAD/CLIC2

GOMF_CALCIUM_CHANNEL_REGULATOR_ACTIVITY	GOMF_CALCIUM_CHANNEL_REGULATOR_ACTIVITY	0.002721822	NRXN2/NPY/CACNG4/AMBP/PRKG1/GEM/PRKCB/REM1/ITPR1/RRAD/CACNG6
GOMF_CHROMATIN_DNA_BINDING	GOMF_CHROMATIN_DNA_BINDING	0.002715332	SMAD3/SUZ12/RELA/BAP1/KDM3B/CTCF/HMGN4/H3-3B/H1-10/SMARCC1/GRHL1/MACROH2A1/ACTL6A/H3-3A/LEMD3/SMARCC2/HDAC2/NR1H3/SMARCB1/HMGN1/NOTCH1/MBD3/DHX9/HSF1/HNRNPU/HNRNPC/RUVBL2/H1-2/POLE3/THRA/HMGN3/RBBP4/KLF4/H1-3/HMGN2/SMARCD2/SIRT6/HDAC1/TOX3/SMARCE1/EZH2/MTA2/REPIN1/SMARCA4/HMGN5/H2AZ1/SBNO2/GRHL2/RCC1/ZIC2/H1-4/NEUROG3/HR/H1-5

GOBP_POSITIVE_REGULATION_OF_KINASE_ACTIVITY	GOBP_POSITIVE_REGULATION_OF_KINASE_ACTIVITY	0.00271937	PLA2G1B/ELANE/GCG/AZU1/CARTPT/RELN/ADIPOQ/CHRNA3/EPHA7/ADCYAP1/F2/SLC8A2/HTR2A/IGF1/GHR/EPGN/FERMT2/HTR2B/EPHA3/ROR2/DDR2/FGF2/GREM1/DAB1/ALKAL1/IGF2/TNFAIP8L3/PRKD1/AGT/CCL21/MAPT/ROS1/ATP2B4/THBS1/CCL19/DYNAP/VLDLR/FZD10/FGF13/FGFR1/CCN1/ANGPT1/TGFB2/TENM1/PKD2/PDGFRA/DKK1/ADRB2/NTF3/LRRK2/DOCK3/PDGFD/PIFO/FGF1/DLG4/NEURL1/CAB39L/PRNP/BMPR1B/NOX4/TEK/PDE5A/MEF2C/FAM20A/TCL1A/S100A12/PDGFC/CLU/ITGB3/VEGFC/MMD/ROBO1/FLT3/CCR7/MUSK/ROR1/IL34/KIT/PDGFRB/DIRAS1
GOBP_GROWTH_HORMONE_RECEPTOR_SIGNALING_PAT	GOBP_GROWTH_HORMONE_RECEPTOR_SIGNALING_PATHWAY	0.002732075	GDF15/LYN/JAK2/CSH1
GOBP_REGULATION_OF_SYSTEMIC_ARTERIAL_BLOOD_PRESSURE	GOBP_REGULATION_OF_SYSTEMIC_ARTERIAL_BLOOD_PRESSURE	0.002731771	SPX/NPPB/AGTR1/TACR1/CTSG/CMA1/AR/ADAMTS16/ADRA1D/P2RX2/SERPINF2/AGT/TPM1/ADRA1B/CPA3/CORIN/AVPR1A/ADRB2/OXTR/AVPR2/ENPEP/KL

GOBP_PROTEIN_MODIFICATION_BY_SMALL_PROTEIN_REMOVAL	GOBP_PROTEIN_MODIFICATION_BY_SMALL_PROTEIN_REMOVAL	0.002727107	BABAM1/USP31/SENP8/TAF4/USP34/USP40/USP36/USP14/COPS4/DESI2/USP42/MYSM1/COPS2/USP39/UBXN1/OTUD4/DESI1/TAF9/SENP2/USP8/UCHL3/TAF12/TAF2/TADA3/TRRAP/TAF5L/OTUD3/USP22/SPATA2/TADA1/SENP1/UIMC1/OTULIN/BAP1/USP45/GPS1/USP19/TADA2B/STAMBP/SENP3/USP33/COPS3/HINT1/PARK7/USP5/JOSD1/VCPIP1/USP3/EPOP/USP37/OTUB1/USP10/UCHL5/USP28/OTUD6B/COPS7B/TAF6L/SART3/ATXN7L3/USP48/BRCC3/BRCA1/YOD1/COPS5/PSMD14/USP7/USP24/BARD1/USP46/TRIM21/USP38/TNIP1/KAT2A/STAMBPL1/SF3B5/TAF5/USP18/SF3B3/VCP/USP1/USP54/CDK1/ZC3H12A/OTUB2/SHMT2/USP43
GOCC_FILAMENTOUS_ACTIN	GOCC_FILAMENTOUS_ACTIN	0.002740716	LDB3/PDLIM3/DPYSL3/SMTN/JAM3/SMTNL2/PDLIM4/PDLIM7
GOCC_INTERCALATED_DISC	GOCC_INTERCALATED_DISC	0.002740883	ATP1A2/DES/PGM5/CTNNA3/ANK2/AKAP6/KCNA5/CDH2/SCN4B/GJC1/FGF13/SLC8A1

GOBP_CELLULAR_RESPONSE_TO_VASCULAR_ENDOTHELIAL_GROWTH_FACTOR_STIMULUS	GOBP_CELLULAR_RESPONSE_TO_VASCULAR_ENDOTHELIAL_GROWTH_FACTOR_STIMULUS	0.002740959	SMOC2/GAS1/DCN/PRKD1/ATP2B4/ADAMTS3/NRP2/MIR342/PDGFRA/CCBE1/MIR27B/SEMA6A/ADGRA2/PTP4A3/RAMP2/HSPB1/EGR3/VEGFC/ROBO1/FLT3/MIR196A1/PDGFRB/GAB1/NRP1
GOBP_ESTABLISHMENT_OF_PROTEIN_LOCALIZATION_TO_CHROMOSOME	GOBP_ESTABLISHMENT_OF_PROTEIN_LOCALIZATION_TO_CHROMOSOME	0.002750415	CCT6A/NABP2/MACROH2A1/SPIDR/ACD/PIH1D1/DKC1/RUVBL2/LRWD1/TCP1/CCT8/CCT2/CCT3/BRCA2/CCT4/CCT7/CCT5/WRAP53/TERT/H1-5
GOBP_CALCIUM_DEPENDENT_CELL_CELL_ADHESION_VIA_PLASMA_MEMBRANE_CELL_ADHESION_MOLECULES	GOBP_CALCIUM_DEPENDENT_CELL_CELL_ADHESION_VIA_PLASMA_MEMBRANE_CELL_ADHESION_MOLECULES	0.002751067	CDH19/CDH2/PCDHB5/NLGN1/PCDHB4/PCDHB6/SELP/CDH23/CDH11/PCDHGC3/PCDHB2/DCHS1/SELL/CDH6/PCDHB3

GOBP_PLATELET_ACTIVATION	GOBP_PLATELET_ACTIVATION	0.00274558	CELA2A/FGA/CTSG/MYL9/F2/FGG/ADAMTS18/FLNA/CSRP1/MMRN1/PRKG1/DGKG/P2RX1/SELP/SERPINE2/COMP/IL6/COL3A1/HBB/PDGFRA/FN1/TSPAN32/THBD/ILK/PDPN/HSPB1/FGB/ACTN1/BLK/ITGB3/P2RY1/IL6ST/AXL/TREML1/MERTK/P2RY12/PEAR1/F2RL2
GOMF_TRANSFORMING_GROWTH_FACTOR_BETA_BINDING	GOMF_TRANSFORMING_GROWTH_FACTOR_BETA_BINDING	0.002760556	CHRDL1/CD36/TGFB3/THBS1/LTBP1/LRRC32/TGFBR3/LTBP3/VASN/ACVR1/CD109/LTBP4/ENG/TGFBR1/TWSG1/ACVRL1/TGFBR2
GOCC_DENSE_CORE_GRANULE	GOCC_DENSE_CORE_GRANULE	0.002760556	SYT4/SPX/PENK/NPY/CHGA/KIF1A/SCG2/SYT1/MYRIP/SYT5/CADPS
GOBP_NEGATIVE_REGULATION_OF_VASCULAR_PERMEABILITY	GOBP_NEGATIVE_REGULATION_OF_VASCULAR_PERMEABILITY	0.002764246	SLIT2/FERMT2/PDE3A/AKAP12/PDE2A/ANGPT1/SH3GL2/RAMP2/CLDN5

GOBP_PURINE_NUCLEOSIDE_MONOPHOSPHATE_BIOSYNTHETIC_PROCESS	GOBP_PURINE_NUCLEOSIDE_MONOPHOSPHATE_BIOSYNTHETIC_PROCESS	0.002776499	DGUOK/ADSS2/AMPD2/GMPS/APRT/IMPDH2/ADK/GART/NUDT2/ATIC/ADSS1/IMPDH1/PFAS/PRPS2/DCK/PPAT/PAICS/ADA
GOBP_REGULATION_OF_SYNAPSE_STRUCTURE_OR_ACTIVITY	GOBP_REGULATION_OF_SYNAPSE_STRUCTURE_OR_ACTIVITY	0.002784458	RELN/EPHA7/GPM6A/NEGR1/FLRT2/ADGRB3/CDH2/ZDHHC15/PAK3/NLGN1/PTPRD/NPTX1/NTN1/SLC17A7/TUBA1A/NFATC4/DMPK/LRRN1/NRP2/CLSTN2/CTTNBP2/DKK1/GPC6/KIF1A/SLITRK6/CAMKV/OXTR/NLGN3/LRRK2/NEURL1/PRNP/SRPX2/SYNDIG1/COLQ/MEF2C/NRCAM/NLGN2/FCGR2B/THBS2/CHRNB2/ADGRB1/MUSK/ARC/ARHGEF15/AMIGO2/OXT/LRRC4B/FZD1/STX1B/AMIGO1/NTNG2/NEDD4/DISC1/GHRL/ARHGAP22/CLSTN3/NTRK2/SNCA/TLR2/FYN/LRP4
GOBP_REGULATION_OF_MORPHOGENESIS_OF_AN_EPITHELIUM	GOBP_REGULATION_OF_MORPHOGENESIS_OF_AN_EPITHELIUM	0.002799694	SFRP1/FGF10/FGF7/AR/SIX2/NOG/FGF2/GREM1/AGT/HGF/ESR1/SHH/SMO/WNT2B/GJA1/SNAI2/FGF1

GOMF_DNA_DIRECTED_DNA_POLYMERASE_ACTIVITY	GOMF_DNA_DIRECTED_DNA_POLYMERASE_ACTIVITY	0.002813766	POLA1/POLL/POLH/POLM/POLE2/POLB/POLE3/POLG/POLD3/POLE/POLQ/POLD1
GOBP_ADRENAL_GLAND_DEVELOPMENT	GOBP_ADRENAL_GLAND_DEVELOPMENT	0.002809414	APOA1/ASCL1/INSM1/CYP1B1/WT1/PDGFRA/PBX1/DKK3/NR3C1
GOBP_POSITIVE_REGULATION_OF_NEURON_PROJECTION_DEVELOPMENT	GOBP_POSITIVE_REGULATION_OF_NEURON_PROJECTION_DEVELOPMENT	0.002813562	RELN/STMN2/NEGR1/DPYSL3/EPHA3/ZDHHC15/ROR2/CNTN1/FLNA/PAK3/ATP1B2/PTN/TNN/CNR1/NLGN1/ALKAL1/PRKD1/AGT/TENM3/SERPINF1/VLDLR/RGS2/HGF/FEZ1/S100A9
GOBP_HISTONE_MONOUBIQUITINATION	GOBP_HISTONE_MONOUBIQUITINATION	0.002826077	PCGF2/CDC73/PCGF3/UBE2E1/PCGF1/TRIM37/PCGF6/RNF168/DDB1/CTR9/LEO1/KDM2B/ATXN7L3/BRCC3/BRCA1/DTX3L/PCGF5/BCOR/BARD1/BRCA2/RNF40/DDB2/RAD51/UHRF1

GOBP_POSITIVE_REGULATION_OF_TELOMERASE_RNA_LOCALIZATION_TO_CAJAL_BODY	GOBP_POSITIVE_REGULATION_OF_TELOMERASE_RNA_LOCALIZATION_TO_CAJAL_BODY	0.002835618	CCT6A/SHQ1/NAF1/RUVBL1/DKC1/RUVBL2/TCP1/CCT8/CCT2/CCT3/CCT4/CCT7/CCT5/NHP2
GOMF_NEUROTRANSMITTER_RECEPTOR_ACTIVITY	GOMF_NEUROTRANSMITTER_RECEPTOR_ACTIVITY	0.002836843	CHRM2/GRIK3/CHRNA3/GRIK1/GRIN2A/HTR2A/GRIK5/HTR2B/GLRB/CHRNB4/CHRM4
GOBP_PHOSPHATIDYLSERINE_METABOLIC_PROCESS	GOBP_PHOSPHATIDYLSERINE_METABOLIC_PROCESS	0.002848504	ABHD12/PLA2G10/ABHD16A/SERINC5/PTDSS2/OSBPL10/LPCAT3/PTDSS1/MBOAT2/SERINC2/MFSD2A/MBOAT1/ABHD12B/LPCAT4/PLA2G2F/PLA2G3
GOMF_TRANSLATION_ELONGATION_FACTOR_ACTIVITY	GOMF_TRANSLATION_ELONGATION_FACTOR_ACTIVITY	0.002856454	GFM1/HBS1L/EEF1A1P5/EEF1A1/ABTB1/GTPBP1/EEF1D/EEF1B2/EEFSEC/EEF2/GFM2/TUFM/EIF5A/EEF1A2/EIF5AL1/EFL1
GOBP_NEGATIVE_REGULATION_OF_OSTEOCLAST_DIFFERENTIATION	GOBP_NEGATIVE_REGULATION_OF_OSTEOCLAST_DIFFERENTIATION	0.002854318	CARTPT/SFRP1/LRRC17/TMEM178A/FBN1/TNFRSF11B

GOBP_EMBRYONIC_DIGESTIVE_TRACT_DEVELOPMENT	GOBP_EMBRYONIC_DIGESTIVE_TRACT_DEVELOPMENT	0.0028614	RBPMS2/FGF10/ALDH1A2/SCT/SIX2/GLI3/FOXF1/TGFB2/SHH/PDGFRA/FOXF2/TCF21/RARRES2
GOCC_DENDRITIC_SHAFT	GOCC_DENDRITIC_SHAFT	0.0028614	SEZ6/HTR2A/MAP1A/RGS7BP/PRKAR2B/LPAR1/SLC8A1/MPP2/KIRREL1/SYNDIG1/NLGN2/GABBR1/MAP2/GPER1
GOBP_PROTEIN_PROCESSING	GOBP_PROTEIN_PROCESSING	0.002870799	HP/BCHE/APOH/SCG5/FGA/NLRP7/CTSG/PCSK1/CMA1/GRIN2A/CPXM2/CPE/FGG/SPON1/GAS1/PCSK2/CPN1/GLI3/F7/SERPINF2/THBS1/TLL1/CPA3/ADAMTS3/SERPINE2/BAG2/COMP/CORIN/SERPINE1/SHH/DHH/CCBE1/CLEC3B/MMP16/CPXM1/LRRK2/PCSK1N/C1R/ENPEP/THBD/PRNP/AEBP1/FGB/MAGEA3/PTCH1/LDLRAD3/DYNC2H1

GOBP_POSITIVE_REGULATION_OF_STRESS_FIBER_ASSEMBLY	GOBP_POSITIVE_REGULATION_OF_STRESS_FIBER_ASSEMBLY	0.002878045	APOA1/SFRP1/MYOC/TACR1/FERMT2/SERPINF2/TPM1/TGFB3/PPM1E/LPAR1
GOMF_IMMUNOGLOBULIN_BINDING	GOMF_IMMUNOGLOBULIN_BINDING	0.002881078	FCER1A/AMBP/PIP/MS4A2/DLG4/FCER2/LILRA2/FCGR2B/FCGR2C/FCAR
GOBP_MODULATION_OF_EXCITATORY_POSTSYNAPTIC_POTENTIAL	GOBP_MODULATION_OF_EXCITATORY_POSTSYNAPTIC_POTENTIAL	0.002893386	RELN/GRIN2A/SLC8A2/IGSF11/RGS4/NLGN4X/NLGN1/DMPK/ADRB2/NLGN3/LRRK2/TMEM108/DLG4/CELF4/NLGN2
GOBP_INTERLEUKIN_8_PRODUCTION	GOBP_INTERLEUKIN_8_PRODUCTION	0.002892079	PLA2G1B/ELANE/APOA2/ADIPOQ/BPI/MIR520C/SSC5D/FCN1/IL6/SERPINE1
GOBP_COPULATION	GOBP_COPULATION	0.002919906	VIP/TAC1/TACR1/CNR1/P2RX1/SERPINE2/AVPR1A/OXTR/P2RY1/OXT/EDNRB

GOBP_POSTREPLICATION_REPAIR	GOBP_POSTREPLICATION_REPAIR	0.002927425	POLDIP2/RCHY1/UBE2N/MAD2L2/USP10/POLH/BRCA1/POLE2/RAD18/VCP/USP1/POLD3/MSH2/PARP10/PCLAF/POLD2/PCNA/DTL/POLD1/USP43
GOBP_REGULATION_OF_TELOMERASE_ACTIVITY	GOBP_REGULATION_OF_TELOMERASE_ACTIVITY	0.002941208	MAPKAPK5/SMG6/HSP90AB1/CTNNB1/PNKP/MAP3K4/NABP2/PARN/HSP90AA1/MAP2K7/XRCC5/NAF1/ACD/PARP4/MYC/SRC/DKC1/MAPK3/TCP1/PTGES3/NEK2/HNRNPD/PIF1/MEN1/CCT2/HNRNPA2B1/PINX1/KLF4/CCT4/AURKB/WRAP53/PRKCQ/TNKS/PARP3/TP53/GRHL2/MAPK15
GOBP_DIGESTIVE_SYSTEM_PROCESS	GOBP_DIGESTIVE_SYSTEM_PROCESS	0.002944139	PNLIP/APOA2/CEL/APOA1/NEUROD1/TACR1/FGF10/NPR3/SCT/PTGER3/SOAT2/CD36/SERPINA3/RBP4/APOA4/CCKBR/OXTR

GOBP_SYNAPTIC_TRANSMISSION_CHOLINERGIC	GOBP_SYNAPTIC_TRANSMISSION_CHOLINERGIC	0.002969818	TACR2/CHRNA3/TACR1/RIC3/LAMA2/CHRNB4
GOBP_MESENCHYME_MORPHOGENESIS	GOBP_MESENCHYME_MORPHOGENESIS	0.003001058	ACTG2/TMEM100/ACTA2/NOG/ACTA1/ACTC1/FOXF1/TGFB3/TGFB2/HEYL/OSR1/ISL1/SNAI2/FOXC2/DCHS1/TWIST1/ROBO1/LEF1/ACVR1/APLNR/BMP5/ENG/SNAI1
GOBP_POSITIVE_REGULATION_OF_CALCIUM_ION_TRANSMEMBRANE_TRANSPORTER_ACTIVITY	GOBP_POSITIVE_REGULATION_OF_CALCIUM_ION_TRANSMEMBRANE_TRANSPORTER_ACTIVITY	0.003017179	CASQ1/JPH2/ANK2/AKAP6/RGN/HSPA2/CACNB2/STAC/PKD2/CACNA2D1
GOBP_REGULATION_OF_GLUCOSE_IMPORT	GOBP_REGULATION_OF_GLUCOSE_IMPORT	0.003015539	PLA2G1B/ADIPOQ/SORBS1/IGF1/GPC3/PID1/ITLN1/KLF15

GOMF_MAGNESIUM_ION_BINDING	GOMF_MAGNESIUM_ION_BINDING	0.003029999	SIK1/FAN1/CDC42BPB/FARSB/IDH3B/MSH6/HACL1/HMGCL/MAP3K5/PGM3/ADPRS/ATP9B/OXSR1/GSS/SRPK1/ENOPH1/GTPBP10/MAST2/STK11/STK26/PIF1/YDJC/ADSS1/RAN/WRN/NT5C3A/PGM2/THG1L/ATP11A/NUDT18/TESC/MAP3K6/NUDT12/MARK2/PPA2/PPA1/PDXP/TKT/MVK/PRPS2/IDH3A/ATP8A1/ILVBL/EPHX2/IDH2/DUT/PDXK/DCTPP1/MSH2/PRIM1/GEN1/IDH1/CIB1/IMPA1/ATP8B1/ITPK1/RPS6KA4/MTHFD2/TPPP/FARSA/PCK1/ENO1/FEN1/ENDOG/NUDT8/ALPI/PLK1/ATP10B/TOP2A/PKM/CDC42BPG/RPS6KA1/ERN2/S100P/ALPP/ATP4A
GOBP_FOCAL_ADHESION_ASSEMBLY	GOBP_FOCAL_ADHESION_ASSEMBLY	0.00305167	SFRP1/ACTN2/MYOC/SORBS1/APOD/STON1/FERMT2/EPHA3/PHLDB2/GREM1/GPM6B/THBS1/FAM107A/DLC1/COL16A1/TEK/ACTN1/THY1/CORO2B

GOBP_NEGATIVE_REGULATION_OF_INNATE_IMMUNE_RESPONSE	GOBP_NEGATIVE_REGULATION_OF_INNATE_IMMUNE_RESPONSE	0.003070229	TRAFD1/OAS1/TYRO3/MICA/ADAR/DUSP10/LYAR/ATG5/ZDHHC18/YTHDF2/KLRC1/KLRD1/DHX58/NR1H3/SLAMF8/RPS19/CNOT7/CACTIN/NMI/PARP1/HLA-E/TRIM21/GFER/HLA-B/HLA-A/PARP14/USP18/OAS3/MMP12/DRD2/HLA-F/PPARG/LGALS9/KIR2DL4/TTLL12/NLRC5
GOBP_INTERFERON_GAMMA_MEDIATED_SIGNALING_PATHWAY	GOBP_INTERFERON_GAMMA_MEDIATED_SIGNALING_PATHWAY	0.003099844	NMI/MED1/PARP14/SOCS1/STAT1/CDC37/PPARG/TP53/NLRC5/IRF1/JAK2/IFNG
GOBP_SMOOTH_MUSCLE_TISSUE_DEVELOPMENT	GOBP_SMOOTH_MUSCLE_TISSUE_DEVELOPMENT	0.003102564	MYLK/MIR145/TNN/ITGA8/PKD2/COL3A1/SHH/SMO/OSR1/EFEMP2
GOBP_MEMBRANE_DEPOLARIZATION_DURING_CARDIAC_MUSCLE_CELL_ACTION_POTENTIAL	GOBP_MEMBRANE_DEPOLARIZATION_DURING_CARDIAC_MUSCLE_CELL_ACTION_POTENTIAL	0.00312502	ATP1A2/ANK2/CACNA1C/SCN4B/CACNB2/SLC8A1/CACNA2D1

GOMF_DEOXYRIBONUCLEASE_ACTIVITY	GOMF_DEOXYRIBONUCLEASE_ACTIVITY	0.00314092	DNASE1L1/MGME1/RAD9A/RPS3/FAN1/DNASE2/XRCC1/APEX2/APTX/MUS81/ERCC5/MAP1S/XRCC3/ZGRF1/RBBP8/DCLRE1B/GEN1/POLE/ISG20/DNASE1L2/DCLRE1A/EXO1/DFFB/FEN1/DNA2/ENDOG/POLD1
GOCC_CUL4_RING_E3_UBIQUITIN_LIGASE_COMPLEX	GOCC_CUL4_RING_E3_UBIQUITIN_LIGASE_COMPLEX	0.003150156	WDTC1/TRPC4AP/AMBRA1/DCAF17/DCAF13/GLMN/DCAF4/DDB1/DCAF16/DCAF11/CUL4A/DCAF7/FBXW5/DDA1/DCAF15/DCAF1/DCAF12/DDB2/DTL
GOBP_POSITIVE_REGULATION_OF_SYNAPTIC_TRANSMISSION_GLUTAMATERGIC	GOBP_POSITIVE_REGULATION_OF_SYNAPTIC_TRANSMISSION_GLUTAMATERGIC	0.003151422	RELN/GRIN2A/ROR2/CACNG4/NLGN1/CCL2/OXTR/NLGN3/TSHZ3/NLGN2
GOBP_REGULATION_OF_EXCRETION	GOBP_REGULATION_OF_EXCRETION	0.003165199	SPX/NPPB/AGTR1/TACR1/AGT/NPR1/CORIN/AVPR1A/AVPR2/STC1/OXT/EDNRB

GOMF_ALCOHOL_BINDING	GOMF_ALCOHOL_BINDING	0.003190533	APOA2/APOA1/APOC3/APOD/TRPC4/TRPC1/SOAT2/ADH7/CAV1/RBP7/RBP4/PLCL1/RLBP1/APOA4/CRABP2/ABCA1/ITPR1/LRAT/PTCH1/ANXA6/SYP/RBP1/ADH4/RBP5
GOBP_PTERIDINE_CONTAINING_COMPOUND_BIOSYNTHETIC_PROCESS	GOBP_PTERIDINE_CONTAINING_COMPOUND_BIOSYNTHETIC_PROCESS	0.003218773	QDPR/GART/ATIC/GCH1/PCBD1/FPGS/DHFRP1/SPR/MTHFD1L/DHFR/MTHFD1
GOBP_CALCIUM_ION_IMPORT	GOBP_CALCIUM_ION_IMPORT	0.003221465	GCG/PLN/TRPC4/CACNA1H/CXCL12/CACNB2/SLC8A1/PKD2/CCL2/SLN/STC1
GOBP_CELL_DIFFERENTIATION_INVOLVED_IN_METANEPHROS_DEVELOPMENT	GOBP_CELL_DIFFERENTIATION_INVOLVED_IN_METANEPHROS_DEVELOPMENT	0.003219377	ADIPOQ/SIX2/GREM1/WWTR1/SMO/OSR1/TCF21
GOBP_REGULATION_OF_ENDOTHELIAL_CELL_CHEMOTAXIS	GOBP_REGULATION_OF_ENDOTHELIAL_CELL_CHEMOTAXIS	0.003219377	SMOC2/FGF2/PRKD1/THBS1/FGFR1/FGF1/MIR149/HSPB1

GOBP_HOMOTYPIC_CELL_CELL_ADHESION	GOBP_HOMOTYPIC_CELL_CELL_ADHESION	0.003208899	CELA2A/FGA/CTSG/MYL9/CTNNA3/FGG/ADAMTS18/FLNA/CSRP1/MMRN1/PRKG1/SERPINE2/COMP/IL6/HBB/PDGFRA/FN1/TSPAN32/ILK/PDPN/HSPB1/FGB/ACTN1/BLK/ITGB3/IL6ST/PLPP3/RDX/XG/P2RY12/PEAR1
GOBP_RESPONSE_TO_PEPTIDE_HORMONE	GOBP_RESPONSE_TO_PEPTIDE_HORMONE	0.00321318	PLA2G1B/REG3G/AHSG/CELA2A/IRS4/AGTR1/ACTN2/ADIPOQ/REG1B/SLC2A4/TRARG1/PCSK1/CDO1/MIR145/GLP2R/SORBS1/GHR/MAP1B/TWIST2/CYP1B1/DDR2/IGFBP1/NR4A3/IGF2/CXCL12/F7/GIP/AGT/CYP11A1/PDK4/PID1/NKX6-1/CAV1/EDNRA/FBN1/KLF15/TIMP4/PRKCB/COL3A1/NR1H4/RAB31/GLP1R/IGFBP5/TNS2/CTSK/RETN/OXTR/FOXC2/CCN2/NEFL/SESN3/LPL/RARRES2/KL/TGFBR3/GNAI1/GPR173/INHBA/GPLD1/ITGB3/SLC30A10/SOGA1/GPER1/RHOQ/COL1A1/GAB1/STC2/TIMP1/OXT/EDNRB/PDE3B

GOCC_PROTEIN_LIPID_COMPLEX	GOCC_PROTEIN_LIPID_COMPLEX	0.003233606	APOA2/APOA1/APOC3/APOH/APOB/VLDLR/APOA4/LPL/CLU/PLTP
GOBP_CALCIUM_ION_HOMEOSTASIS	GOBP_CALCIUM_ION_HOMEOSTASIS	0.003249027	ELANE/CASQ2/ATP1A2/PLN/CASQ1/JPH2/SCGN/ANK2/AKAP6/F2/SLC8A2/HTR2A/RGN/DMD/RYR3/HTR2B/FLNA/CACNA1C/SGCD/FGF2/TRPC4/PTH1R/TRPC1/CXCL12/HRC/PRKD1/CCL21/SYPL2/SV2A/P2RX1/ATP2B4/CCL19/MICU3/DMPK/CAV1/EDNRA/TMEM178A/SLC8A1/PKD2/BDKRB1/PRKCB/CDH23/SLC24A3/CCL11/ITPR1/RYR2/STC1/KL/CLIC2/THY1/FAM20A/JPH3/ANXA6/MCOLN3/ITGB3/P2RY1/GPER1/CCR7
GOBP_MUSCLE_CELL_CELLULAR_HOMEOSTASIS	GOBP_MUSCLE_CELL_CELLULAR_HOMEOSTASIS	0.003269289	PLN/DMD/CFL2/CAV1/LOX
GOBP_POSITIVE_REGULATION_OF_HEART_GROWTH	GOBP_POSITIVE_REGULATION_OF_HEART_GROWTH	0.003292541	AKAP6/IGF1/ZFPM2/FGF2/WT1/GLI1/TGFBR3/MEF2C/MIR222/ACACB/TBX5/HAMP/FGFR2

GOBP_REGULATION_OF_EPITHELIAL_TO_MESENCHYMAL_TRANSITION	GOBP_REGULATION_OF_EPITHELIAL_TO_MESENCHYMAL_TRANSITION	0.003287981	SFRP1/DACT3/SFRP2/NOG/FERMT2/EPHA3/PHLDB2/GREM1/TGFB1I1/TGFB3/MARK1/CRB2/TGFB2/WWTR1/IL6/ZNF750/DAB2/ISL1/MIR149/PDPN/PHLDB1/MIR222/LDLRAD4/TWIST1/TBX5/VASN/LEF1/COL1A1/ACVR1/SPRED3/BMP5/FUZ/ENG/SNAI1/GLIPR2/SERPINB3/SPRY1/TGFBR1/GATA3/TIAM1/RGCC/MIR221

GOBP_HUMORAL_IMMUNE_RESPONSE	GOBP_HUMORAL_IMMUNE_RESPONSE	0.003293267	PLA2G1B/ELANE/PRTN3/REG3G/AZU1/DEFA4/RNASE3/C7/FGA/REG1B/CTSG/F2/RNASE2/MFAP4/TF/DEFA6/CXCL6/MASP1/PRSS2/COLEC11/CAMP/C4A/FCN1/C4B/IL6/IGHV1-69D/S100A9/DEFB126/CCL2/KRT1/PGLYRP1/A2M/GPR183/C1S/IGHV1-69-2/C1R/IGHG4/WFDC3/BST1/IGHV6-1/CFH/RARRES2/FCER2/FGB/WFDC10B/IGHV7-4-1/MEF2C/SERPING1/S100A12/CLU/RNASE6/FCGR2B
GOBP_LONG_TERM_SYNAPTIC_POTENTIATION	GOBP_LONG_TERM_SYNAPTIC_POTENTIATION	0.003308199	RELN/SCGN/GRIN2A/SLC8A2/NOG/IGSF11/PTN/MIR320C2/ADCY1/GIP/NFATC4/CX3CR1/SERPINE2/PRRT1/FAM107A/MIR342/SNAP25/MPP2/TSHZ3/MIR320D1/PRNP/RASGRF2/MIR95/ARC

GOBP_REGULATION_OF_ENDOPLASMIC_RETICULUM_UNFOLDED_PROTEIN_RESPONSE	GOBP_REGULATION_OF_ENDOPLASMIC_RETICULUM_UNFOLDED_PROTEIN_RESPONSE	0.003367404	BFAR/RACK1/UFL1/COPS5/BAX/HSPA5/DDRGK1/DAB2IP/TMEM33/ABCA7/BAK1/AGR2
GOMF_INSULIN_RECEPTOR_BINDING	GOMF_INSULIN_RECEPTOR_BINDING	0.003373559	IRS4/SORBS1/IGF1/IGF2
GOBP_APPENDAGE_MORPHOGENESIS	GOBP_APPENDAGE_MORPHOGENESIS	0.003372275	COL2A1/HAND2/FGF10/ALDH1A2/SFRP2/NOG/GPC3/CACNA1C/ZBTB16/GREM1/HOXD10/GLI3/RECK/RSPO2/AFF3/SALL4/MAP3K20/TGFB2/COL3A1/SHH/OSR1/PBX1/PRRX1/DKK1/CRABP2/PTCH1/TWIST1/TBX5/DYNC2H1/HOXD9/LEF1/SEMA3C/FGFR2/CIBAR1/SP8/SOX11

GOBP_POSITIVE_REGULATION_OF_NEUROGENESIS	GOBP_POSITIVE_REGULATION_OF_NEUROGENESIS	0.003375499	NKX2-2/SOX10/RELN/ASCL1/SLIT2/CHODL/MAP1B/MAP6/NGF/PAK3/PTN/FGF2/PLXNA4/DIXDC1/PTPRD/CXCL12/GLI3/IL33/NTN1/MAPT/RND2/LPAR3/SERPINF1/NKX6-1/CX3CR1/SMARCD3/ASPA/SERPINE2/L1CAM/IL6/SHH/SMO/FN1/CRABP2/ID4/NEURL1/NEFL/TENM4/ISLR2/PTPRZ1/MIR222/VEGFC/ROBO1/CXCR4/GPER1/IL6ST/IL34/KIT
GOBP_ENDOCRINE_PROCESS	GOBP_ENDOCRINE_PROCESS	0.003380195	AGTR1/TACR2/TACR1/CTSG/CMA1/SERPINF2/AGT/SELENOM/CPA3/CORIN/AVPR1A/DAB2/CGA/GDF9/RETN/OXTR/GJA1/AVPR2/ENPEP/INHBA/INHA/EDNRB/FZD4/EDN2/RASL10B/BMP6/GHRL/F2R/ACE2/INHBB/C1QTNF1/GATA3

GOBP_PHOSPHATIDYLINOSITOL_3_KINASE_SIGNALING	GOBP_PHOSPHATIDYLINOSITOL_3_KINASE_SIGNALING	0.003386873	RELN/MYOC/F2/HTR2A/IGF1/HTR2B/ROR2/FGF2/DCN/SEMA3E/AGT/CSF3/SELP/HGF/FGFR1/ANGPT1/TGFB2/SERPINE2/PDGFRA/PTPN13/FN1/PREX2/PDGFD/FBXL2/CRNN/TEK/PDGFC/TWIST1/FLT3/GPER1/ROR1/KIT/PDGFRB
GOBP_GLYCOLIPID_CATABOLIC_PR	GOBP_GLYCOLIPID_CATABOLIC_PROCESS	0.003402261	PNLIPRP2/LCT

GOBP_ANTIGEN_RECEPTOR_MEDIATED_SIGNALING_PATHWAY	GOBP_ANTIGEN_RECEPTOR_MEDIATED_SIGNALING_PATHWAY	0.003409293	UBASH3A/CD79A/BTN1A1/IGHV3-15/IKBKB/SH2B2/RPS3/BTN2A1/IGHV3-53/EIF2B2/PAWR/DGKZ/HLA-DPB1/CD19/RBCK1/IGHV3-23/CD226/IGHV4-4/IGHV1-3/TEC/PTPN6/IGHV4-59/IGLC6/BCL10/BTN3A3/KHDRBS1/DENND1B/TNFRSF21/LGALS3/BTN2A2/BTNL9/BAX/PRKD2/PLCG2/STK11/SHB/SLA2/LIME1/IGHV4-61/PTPN22/SYK/IGHV3-30/BLNK/RIPK2/NECTIN2/LPXN/BTN3A2/TRDC/BTN2A3P/HLA-A/FCHO1/HRAS/IGLL5/BTNL8/CD8A/IGHA1/CSK/STOML2/BCAR1/IGHD/IGHV3-72/IGHV3-64D/ZC3H12A/HLA-DRB1/PTPRJ/IGHV3-33/CD79B/KCNN4/HLA-DQB1/IGHV3-48/BTN3A1/IGHV3-[illegible]
GOBP_NEGATIVE_CHEMOTAXIS	GOBP_NEGATIVE_CHEMOTAXIS	0.003453945	APOA1/EPHA7/SLIT2/FLRT2/SLIT3/PLXNA4/SEMA3E/NTN1/NRP2/SEMA6A/SEMA6D/SEMA6C/ITGB3/SEMA3G/SEMA5B/SEMA3D/ROBO1/SEMA3C
GOBP_REGULATION_OF_MUSCLE_HYPERTROPHY	GOBP_REGULATION_OF_MUSCLE_HYPERTROPHY	0.003453945	HAND2/MIR145/AKAP6/IGF1/PI16/RGS4/NR4A3/PDE9A/AGT/ATP2B4/RGS2/IGFBP5

GOBP_RRNA_CATABOLIC_PROCESS	GOBP_RRNA_CATABOLIC_PROCESS	0.003473264	ZCCHC7/SLFN13/EXOSC8/DIS3L/DIS3/XRN1/DEDD2/EXOSC10/EXOSC2/EXOSC7/EXOSC5/DDX49/EXOSC9/EXOSC4/ERN2
GOCC_CYCLIN_DEPENDENT_PROTEIN_KINASE_HOLOENZYME_COMPLEX	GOCC_CYCLIN_DEPENDENT_PROTEIN_KINASE_HOLOENZYME_COMPLEX	0.003471142	CDK2/CDK12/BCCIP/CCNQ/CCNT1/CDK7/CCND3/CCNK/CDK5R1/CCNC/CDK16/CCNJ/CNPPD1/CCNI2/CKS1B/CCNG1/CDK5/CKS2/CDK1/CDK6/CCNB2/PCNA/CCNB1/CCNP/CCNA2/CCND1/CCNF/CCNE2/CCNO
GOBP_CELL_SUBSTRATE_JUNCTION_ORGANIZATION	GOBP_CELL_SUBSTRATE_JUNCTION_ORGANIZATION	0.003485898	SFRP1/ACTN2/MYOC/SORBS1/APOD/STON1/FERMT2/EPHA3/PHLDB2/GREM1/GPM6B/THBS1/ITGA5/FAM107A/FN1/DLC1/COL16A1/TEK/ACTN1/THY1/DST/LAMC1/CORO2B/ITGB3

GOBP_DEVELOPMENTAL_MATURATION	GOBP_DEVELOPMENTAL_MATURATION	0.003511951	SEZ6L/SOX10/RELN/MYOC/FEV/KCNB1/CHRDL1/ASCL1/BNC1/ALDH1A2/SEZ6/IGF1/PGR/PTPRN/FGG/ADGRB3/ZDHHC15/MAP1B/ZBTB16/GREM1/PTH1R/NLGN1/LGI4/PDE3A/CCL21/RECK/NFATC4/CCL19/NKX6-1/EDNRA/CX3CR1/C1QL1/TGFB2/MMP2/TDRD10/RHEX/CABYR/PALM/LRRK2/WNT10B/DCHS1/LHX6/DLG4/NEURL1/S1PR1/NEFL/SLC26A3/PAEP/NRCAM/NPR2/NFIA/HOXA5/SYP
GOBP_PTERIDINE_CONTAINING_COMPOUND_METABOLIC_PROCESS	GOBP_PTERIDINE_CONTAINING_COMPOUND_METABOLIC_PROCESS	0.003517437	FOLR1/MTHFD2L/ALDH1L1/GART/ATIC/MTHFR/GCH1/PCBD1/FPGS/DHFRP1/SPR/SHMT1/MTHFD2/MTHFD1L/DHFR/MTHFD1/SLC19A1/SHMT2/TYMS
GOBP_NUCLEOBASE_BIOSYNTHETIC_PROCESS	GOBP_NUCLEOBASE_BIOSYNTHETIC_PROCESS	0.003522072	APRT/MTOR/GART/CAD/CTPS1/CMPK1/SHMT1/PPAT/PAICS/ADA/SHMT2/CPS1

GOMF_CALMODULIN_BINDING	GOMF_CALMODULIN_BINDING	0.003531801	CNN1/MYH11/MYLK/NGFR/PNCK/SLC8A2/GAP43/CALD1/RYR3/MAP6/RGS4/CACNA1C/PCP4/PDE1A/ADCY1/ADD2/AKAP12/ATP2B4/GEM/RGS2/PDE1C/PDE1B/SLC8A1/MYH10/KCNQ3/CAMKV/REM1/FBXL2/PHKG1/AEBP1/CAMK1D/RRAD/RYR2/ITPKB/SYT1/RASGRF2/MAP2

GOCC_PROTEIN_ACETYLTRANSFERASE_COMPLEX	GOCC_PROTEIN_ACETYLTRANSFERASE_COMPLEX	0.003550712	TAF4/KAT6B/YEATS2/KANSL3/JADE1/EP300/ING3/KAT6A/KANSL2/NAA30/ING5/TAF9/TAF12/YEATS4/TAF2/TADA3/TRRAP/TAF5L/USP22/MLLT1/TADA1/DR1/NAA20/EP400/TADA2B/ZZZ3/NAA15/BRD8/JADE2/NAA35/MRGBP/ACTL6A/KAT5/TAF6L/ATXN7L3/BRPF1/NAA25/RUVBL1/NAA50/NAA10/RUVBL2/POLE3/MCRS1/NAA38/WDR5/KAT2A/SF3B5/TAF5/SF3B3/HCFC1/MSL1/ACTBL2
GOMF_G_PROTEIN_COUPLED_AMINE_RECEPTOR_ACTIVITY	GOMF_G_PROTEIN_COUPLED_AMINE_RECEPTOR_ACTIVITY	0.003570551	CHRM2/HTR2A/HTR2B/ADRA1D/ADRA1B/CHRM4/ADRB2/TAAR1/HRH2/CHRM3
GOBP_REGULATION_OF_CELL_MIGRATION_INVOLVED_IN_SPROUTING_ANGIOGENESIS	GOBP_REGULATION_OF_CELL_MIGRATION_INVOLVED_IN_SPROUTING_ANGIOGENESIS	0.003596299	MIR483/MEOX2/FGF2/AKT3/THBS1/RHOJ/MIR27B/FOXC2/MIR149/SRPX2/MIR10B/MMRN2/MIR196A1/STARD13/NRP1/TBXA2R/HDAC5

GOBP_NEGATIVE_REGULATION_OF_CHEMOTAXIS	GOBP_NEGATIVE_REGULATION_OF_CHEMOTAXIS	0.003593294	ELANE/SLIT2/GREM1/SEMA3E/THBS1/CCL2/CCN3/SEMA6A/SEMA6D/MIR149/DUSP1/SEMA6C/SEMA3G/SEMA5B/SEMA3D/ROBO1/SEMA3C/NRP1/ANGPT2/DUSP3/SEMA6B/C5/SEMA5A
GOBP_SMOOTH_MUSCLE_CELL_DIFFERENTIATION	GOBP_SMOOTH_MUSCLE_CELL_DIFFERENTIATION	0.003633885	MYOCD/RBPMS2/FGF10/MIR145/ZEB1/ITGA8/FOXF1/NFATC4/PRDM6/EDNRA/TBX18/COMP/SHH/EFEMP2/RAMP2/QKI/MEF2C/GPER1/KIT/ACVR1/EDNRB/TMEM204/APLNR/FGFR2/EREG/ENG

GOBP_GRANULOCYTE_MIGRATION	GOBP_GRANULOCYTE_MIGRATION	0.003644414	PLA2G1B/PRTN3/CCL16/THBS4/SLIT2/CCL26/JAM3/CXCL6/CCL21/ITGA9/THBS1/GP2/CCL19/S100A8/IL1R1/TGFB2/ITGA1/S100A9/CCL2/CCL11/BST1/CXCR2/CD300H/RARRES2/CAMK1D/SCG2/CXCR1/S100A12/RIPOR2/CD99L2/MPP1/CCR7/IL34/PECAM1/TNFSF18/EDN2/XG
GOBP_CELLULAR_RESPONSE_TO_ARSENIC_CONTAINING_SUBSTANCE	GOBP_CELLULAR_RESPONSE_TO_ARSENIC_CONTAINING_SUBSTANCE	0.003649918	HSF1/ZFAND1/MAPK13/VCP/HNRNPA1/PPIF/ZC3H12A/MKNK2/GSTO2
GOBP_REGULATION_OF_TELOMERE_MAINTENANCE_IN_RESPONSE_TO_DNA_DAMAGE	GOBP_REGULATION_OF_TELOMERE_MAINTENANCE_IN_RESPONSE_TO_DNA_DAMAGE	0.003666214	ACTL6A/RTEL1/UCHL5/INO80D/RUVBL1/XRCC1/INO80E/INO80/RUVBL2/MCRS1/NFRKB
GOBP_RETINA_VASCULATURE_DEVELOPMENT_IN_CAMERA_TYPE_EYE	GOBP_RETINA_VASCULATURE_DEVELOPMENT_IN_CAMERA_TYPE_EYE	0.003663646	CYP1B1/VSTM4/LAMA1/NDP/RHOJ/CLIC4/PDGFRA/PDGFRB/ARHGEF15/NRP1/FZD4

GOBP_POSITIVE_REGULATION_OF_CELL_PROJECTION_ORGANIZATION	GOBP_POSITIVE_REGULATION_OF_CELL_PROJECTION_ORGANIZATION	0.003673673	RELN/STMN2/GPM6A/NEGR1/SLIT2/CHODL/DPYSL3/EPHA3/ZDHHC15/MAP1B/MAP6/ROR2/CNTN1/FLNA/NGF/PAK3/ATP1B2/PTN/TNN/PLXNA4/CNR1/DIXDC1/NLGN1/PTPRD/ALKAL1/CXCL12/NTN1/PRKD1/AGT/CDC42EP3/CCL21/TGFB3/DZIP1/MAPT/RND2/TENM3/CCL19/LPAR3/SERPINF1/VLDLR/RGS2/HGF/TENM1/L1CAM/FEZ1/S100A9/FN1/PALM/CRABP2/DLG4/NEURL1/NEFL/ISLR2/DOCK11/CAMK1D/MIR222/RIPOR2/ROBO1/CCR7/RHOQ/ROR1/KIT/NDNF/NDRG4/NRP1/FZD4/SCN1B/BMP5/P2RY12/FZD1/SERPINI1/ARSB/AMIGO1/FUZ/GRIP1/PLXNC1
GOBP_DEFENSE_RESPONSE_TO_GRAM_POSITIVE_BACTERIUM	GOBP_DEFENSE_RESPONSE_TO_GRAM_POSITIVE_BACTERIUM	0.003691141	PLA2G1B/REG3G/DEFA4/RNASE3/CTSG/RNASE2/SSC5D/GBP6/DEFA6/CD36/CHGA/CAMP/C10orf99/IL6/PGLYRP1

GOBP_CARBOHYDRATE_DERIVATIVE_TRANSPORT	GOBP_CARBOHYDRATE_DERIVATIVE_TRANSPORT	0.003706201	SLC35B3/LRRC8D/PLEKHA8/SLC25A23/RALBP1/ABCD1/SLC25A17/RFT1/SLC35A1/LRRC8A/SLC35D1/SLC10A7/SLC15A3/SLC50A1/SLC37A4/CD47/SLC25A5/SLC35B1/GJB1/SLC35A2/SLC17A9/SLC35D2/SLC25A24/ABCC3/CPTP/SLC25A19/SLC5A1/SLC35A4/SLC35C1/MFSD2A/SLC29A2/SLC28A3/SLC5A2/SLC35A3/SLC19A1/SLC35D3/SLC37A1/CALHM3/SLC28A2
GOBP_REGULATION_OF_SYNAPTIC_VESICLE_EXOCYTOSIS	GOBP_REGULATION_OF_SYNAPTIC_VESICLE_EXOCYTOSIS	0.003730321	SYT4/CHRM2/HTR2A/CNR1/NLGN1/ADCY1/CACNB2/P2RX1/PRKCB/LRRK2/APBA1/SYT1/SYP/P2RY1/BACE1/PFN2
GOBP_LEUKOTRIENE_METABOLIC_PROCESS	GOBP_LEUKOTRIENE_METABOLIC_PROCESS	0.003741651	PLA2G1B/CPA1/PLA2G5/GGT5/GGTA1/DPEP1
GOBP_RESPONSE_TO_PROSTAGLANDIN	GOBP_RESPONSE_TO_PROSTAGLANDIN	0.003750697	SFRP1/APOB/PTGFR/PRKAA2/CCL21/CCL19/A2M/TGFBR3/CCR7/PTGDR/OXT

GOBP_EXTRACELLULAR_MATRIX_DISASSEMBLY	GOBP_EXTRACELLULAR_MATRIX_DISASSEMBLY	0.003760024	ELANE/PRSS1/CTSG/CMA1/DDR2/MMP8/PRSS2/CTSV/MMP3/MMP2/IL6/MMP7/MMP19/CTSK/FAP/PDPN/LAMC1/TPSAB1/SH3PXD2B/ADAMTS5/TMPRSS6/LRP1/GSN
GOBP_RESPIRATORY_ELECTRON_TRANSPORT_CHAIN	GOBP_RESPIRATORY_ELECTRON_TRANSPORT_CHAIN	0.003758049	NDUFB8/NDUFC2/ETFB/PUM2/NDUFB5/DNAJC15/NDUFC1/NDUFA1/COX7B/CHCHD2/NDUFB4/UQCRB/COX6B1/NDUFS8/UQCR11/NDUFB2/COA6/DGUOK/ETFDH/COX7C/ETFA/NDUFA8/ETFRF1/COX7A2L/NDUFA6/NDUFB10/DLD/NDUFS5/COX7A2P2/NDUFB3/NDUFA12/NDUFA5/UQCRQ/GPD1/PARK7/BID/NDUFA9/NDUFB6/NDUFAF1/NDUFS6/UQCRFS1/AFG1L/NDUFA10/NDUFS2/NDUFA2/NDUFS1/COX7A2/NDUFS3/SDHD/GPD2/NDUFB7/COX5B/NDUFAB1/UQCR10/COX4I1/COX6A1/PLEC/UQCC3/CYCS/SDHB/COQ9/COX15/COX5A/UQCRC2/UQCRFS1P1/UQCRH/GHITM/COX8A/SDHA/NDUFV1/MYBBP1A/CDK1/NDUFS7/AIFM2/UQCRC1/UQCRHL/CYC1/MIR210/SLC25A22/CCNB1/SCO2

GOBP_NEURAL_CREST_CELL_DIFFERENTIATION	GOBP_NEURAL_CREST_CELL_DIFFERENTIATION	0.003768701	SOX10/SFRP1/HAND2/ALDH1A2/HTR2B/CDH2/FRZB/SEMA3E/RADIL/EDNRA/NRP2/SHH/SMO/FN1/ISL1/SNAI2/FOXC2/SEMA6A/SEMA6D/MEF2C/ANXA6/TWIST1/SEMA6C/SEMA3G/SEMA5B/SEMA3D/ACVR1/SEMA3C/EDNRB/NRP1
GOBP_STRESS_FIBER_ASSEMBLY	GOBP_STRESS_FIBER_ASSEMBLY	0.003767991	APOA1/SFRP1/MYOC/TACR1/SORBS1/FERMT2/PHLDB2/ELN/SERPINF2/TPM1/TGFB3/PPM1E/CGNL1/LPAR1/DLC1/CCN2/ARHGAP28/MIR149/S1PR1/CORO2B/PHACTR1/ARHGEF15/NRP1/PFN2

GOBP_REGULATION_OF_AXONOGENESIS	GOBP_REGULATION_OF_AXONOGENESIS	0.003771356	EPHA7/SLIT2/CHODL/CDH2/MAP1B/MAP6/NGF/PAK3/DAB1/PLXNA4/DIXDC1/CXCL12/SEMA3E/NTN1/MAPT/RND2/LPAR3/FGF13/L1CAM/FN1/SPART/EFNB3/CRABP2/BRSK1/SEMA6A/SEMA6D/NEFL/LRRC4C/ISLR2/THY1/SEMA6C/SEMA3G/MAP2/SEMA5B/SEMA3D/ROBO1/SEMA3C/NRP1/AMIGO1/CHN1/BRSK2/PLXNC1/ULK2/DISC1/SEMA6B/NTRK2/SEMA5A/LRP4
GOCC_SMALL_SUBUNIT_PROCESSOME	GOCC_SMALL_SUBUNIT_PROCESSOME	0.003779013	KRR1/UTP25/UTP14A/UTP11/NOP56/DCAF13/WDR46/HEATR1/NOL10/UTP3/UTP4/XRCC5/RPS7/IMP3/WDR36/NOP58/UTP20/FBL/PRKDC/NOC4L/WDR3/TBL3/PDCD11/IMP4/RRP9/NOL6/PWP2/NOP14
GOBP_REGULATION_OF_PEPTIDYL_THREONINE_PHOS	GOBP_REGULATION_OF_PEPTIDYL_THREONINE_PHOSPHORYLATION	0.003777681	GCG/AZU1/TRIM6

GOCC_EXORIBONUCLEASE_COMPLEX	GOCC_EXORIBONUCLEASE_COMPLEX	0.003791658	DIS3L2/EXOSC8/DIS3L/LAS1L/DIS3/GTPBP1/MPHOSPH6/EXOSC10/EXOSC2/EXOSC7/EXOSC5/SUPV3L1/EXOSC9/EXOSC4
GOBP_INTRINSIC_APOPTOTIC_SIGNALING_PATHWAY_BY_P53_CLASS_MEDIATOR	GOBP_INTRINSIC_APOPTOTIC_SIGNALING_PATHWAY_BY_P53_CLASS_MEDIATOR	0.003788665	USP28/RPS7/ATAD5/CHEK2/ING2/HIPK1/RRN3/BAX/MYC/HNRNPK/PML/STK11/PYCARD/KDM1A/PERP/CASP6/BRCA2/TRIAP1/RRM2B/MSX1/MSH2/CD74/MYBBP1A/PMAIP1/DDIT4/E2F1/TP53/EIF5A/MDM2/AEN/TP73/MUC1/E2F2/ELL3
GOBP_ANTIBACTERIAL_HUMORAL_RESPONSE	GOBP_ANTIBACTERIAL_HUMORAL_RESPONSE	0.003801327	PLA2G1B/ELANE/DEFA4/RNASE3/FGA/CTSG/RNASE2/TF/DEFA6/CAMP

GOBP_DORSAL_VENTRAL_PATTERN_FORMATION	GOBP_DORSAL_VENTRAL_PATTERN_FORMATION	0.003795786	NKX2-2/CER1/SFRP1/ASCL1/NOG/GREM2/GREM1/GLI3/CHRD/GLI1/TLL1/HHIP/HOXA2/SHH/SMO/GPR161/BMPR1B/PTCH1/DYNC2H1/CXXC4/ACVR1/PRKACB/GLI2
GOBP_CARBOHYDRATE_TRANSMEMBRANE_TRANSPORT	GOBP_CARBOHYDRATE_TRANSMEMBRANE_TRANSPORT	0.00380002	PLA2G1B/ADIPOQ/SLC2A4/TRARG1/SORBS1/IGF1/GPC3/CLIP3/SLC2A2/NR4A3/GIP/PID1/EDNRA/SLC45A1/ITLN1/KLF15/PRKCB/AQP10/FFAR4

GOCC_EXOCYTIC_VESICLE	GOCC_EXOCYTIC_VESICLE	0.003799956	SYT4/PENK/SYNGR4/GRIN2A/IGF1/PTPRN/STON1/DPYSL3/NGF/SLC18A2/SV2B/ATP6V1G2/AMPH/SLC17A7/SYPL2/SV2A/PRRT1/CTTNBP2/SNAP25/LAMP5/SYT11/BSN/NTF3/LRRK2/BRSK1/DLG4/TRIM9/RAB3B/RAB3IL1/SEPTIN4/SYNDIG1/SLC18A1/APBA1/RAB3C/GABBR1/SYT1/SYP/SYT5/SYNGR1/SNCAIP/BACE1
GOMF_MHC_CLASS_I_PROTEIN_BINDING	GOMF_MHC_CLASS_I_PROTEIN_BINDING	0.003806297	PILRB/TUBB/DERL1/CD244/TAPBP/HLA-E/BCAP31/ATP5F1A/VCP/CD8A/ATP5F1B/TAP2/TUBB4B/TAP1

GOBP_POSITIVE_REGULATION_OF_DNA_TEMPLATED_TRANSCRIPTION_ELONGATION	GOBP_POSITIVE_REGULATION_OF_DNA_TEMPLATED_TRANSCRIPTION_ELONGATION	0.003820021	MED28/SCAF8/MED22/PWWP2A/MED10/CDC73/ELL/BRD4/CDK13/ERCC6/MED30/CCNT2/CTNNB1/MED8/MED11/MED27/CDK12/SUPT16H/CCNT1/CCNK/TCERG1/LEO1/SUPT5H/MED14/MED25/MED23/NCBP1/MED16/HMGN1/MED15/MED9/GTF2F1/MED26/MED1/MED18/ALYREF/PWWP2B/MED24/ELL3

GOMF_METAL_CLUSTER_BINDING	GOMF_METAL_CLUSTER_BINDING	0.003851863	RSAD1/FDX1/GLRX5/NDUFS8/ETFDH/ERCC2/CISD2/DPYD/FXN/ACO1/IREB2/RPS3/DPH1/GLRX3/RTEL1/UQCRFS1/NDUFS2/ABCE1/BOLA2B/NDUFS1/PRIM2/NUBP1/LIAS/NTHL1/CIAPIN1/ELP3/NUBP2/SDHB/CIAO3/XDH/DDX11/UQCRFS1P1/ACO2/DPH2/NDUFV1/CISD3/POLE/NDUFS7/KIF4A/PPAT/DNA2/POLD1/BRIP1
GOBP_CELLULAR_HORMONE_METABOLIC_PROCESS	GOBP_CELLULAR_HORMONE_METABOLIC_PROCESS	0.003873881	PNLIP/AFP/CEL/ADH1B/ALDH1A2/TTR/CYP1B1/UGT2B17/CACNA1H/RDH8/CYP11A1/ADH1A/ADH7/UGT1A7/RBP4/ESR1/SHH/PDGFRA/DAB2/HSD17B6/CRABP2/DKK3/CHST10/LRAT/ALDH1A3/CYP3A4/CYP1A1/RBP1/ADH4

GOBP_CARDIAC_MUSCLE_CELL_ACTION_POTENTIAL	GOBP_CARDIAC_MUSCLE_CELL_ACTION_POTENTIAL	0.003888259	ATP1A2/CTNNA3/ANK2/KCNA5/DMD/FLNA/CACNA1C/SCN4B/KCNJ8/CACNB2/KCNE4/KCNH2/GJC1/CAV1/FGF13/TBX18/SLC8A1/CACNA2D1/GJA1/RYR2/FGF12/SCN3B/SCN1B/SLC4A3/SNTA1/KCNIP2/GJA5/KCNJ5
GOMF_CARBOXYLIC_ESTER_HYDROLASE_ACTIVITY	GOMF_CARBOXYLIC_ESTER_HYDROLASE_ACTIVITY	0.003895278	PLA2G1B/PNLIP/PNLIPRP1/CEL/PNLIPRP2/BCHE/CES1/RGN/PLA2G5/CLC/PLA2G2C/PLA2G12B/PLA2G4C

GOBP_RESPONSE_TO_EXTRACELLULAR_STIMULUS	GOBP_RESPONSE_TO_EXTRACELLULAR_STIMULUS	0.003904027	CLPS/ALB/MPO/UCN3/SPX/CARTPT/GHRH/BCHE/PENK/SFRP1/ADIPOQ/KCNB1/MYOCD/PCSK1/ASCL1/ALDH1A2/CYP24A1/SCT/GHR/SFRP2/LGSN/SLC38A3/MAP1B/NPY/CYP1B1/PRKAA2/CNR1/ACTA1/F7/ZEB1/GIP/MN1/PRKD1/PDK4/KANK2/MAP1LC3C/TNFRSF11B/SLC8A1/NR1H4/FAM107A/AVPR1A/SCTR/CLPSL2/ADRB2/SGIP1/A2M/FOLR2/CTSK/WNT2B/LRRK2/PCSK1N/BRSK1/SNAI2/SSTR2/SESN3/ASGR1/LPL/RRAGD/STC1/KL/LRAT
GOBP_MITOTIC_INTRA_S_DNA_DAMAGE_CHECKPOINT_SIGNALING	GOBP_MITOTIC_INTRA_S_DNA_DAMAGE_CHECKPOINT_SIGNALING	0.003921249	RAD9A/RAD9B/NEK11/XPC/CHEK2/EME1/MUS81/MSH2/TIPIN/EME2/FANCD2
GOBP_PROTEIN_KINASE_C_ACTIVATING_G_PROTEIN_COUPLED_RECEP	GOBP_PROTEIN_KINASE_C_ACTIVATING_G_PROTEIN_COUPLED_RECEPTOR_SIGNALING_PA	0.003918144	AZU1/GAP43/HTR2B/DGKG/BDKRB1

GOBP_SPINAL_CORD_DEVELOPMENT	GOBP_SPINAL_CORD_DEVELOPMENT	0.003921612	NKX2-2/RELN/ASCL1/NOG/DAB1/VIT/DAAM2/HOXD10/GLI3/CHRD/VLDLR/PKD2/SHH/SMO/PBX3/ISL1/GDF7/NEFL/PTCH1/DYNC2H1/ADARB1/GLI2/PTBP2/SOX11/PLXDC1/PKD1
GOBP_RESPONSE_TO_FIBROBLAST_GROWTH_FACTOR	GOBP_RESPONSE_TO_FIBROBLAST_GROWTH_FACTOR	0.003929187	SFRP1/NGFR/FGF10/TRIM71/FLRT2/FGF7/NOG/SMOC2/FGF2/THBS1/HHIP/FGFR1/SCGB1A1/FLRT1/CCL2/KLB/CCN2/FGF1/MIR149/KL/EGR3/FGF12/SULF1/SHISA2/COL1A1/NDNF/FGFR2/FAM20C/FUZ/MIR339

GOBP_INSULIN_SECRETION	GOBP_INSULIN_SECRETION	0.00393449	GCG/UCN3/CARTPT/CELA2A/SFRP1/NEUROD1/NNAT/KCNB1/ADCYAP1/ADCY5/KCNA5/PTPRN/CNR1/GIP/CHGA/RFX6/NKX6-1/RBP4/IL6/PRKN/NR1H4/SNAP25/ISL1/CCN3/GPR27/KLF7/NLGN2/BLK/GPLD1/MYRIP
GOBP_CARDIAC_MUSCLE_CELL_ACTION_POTENTIAL_INVOLVED_IN_CONTRACTION	GOBP_CARDIAC_MUSCLE_CELL_ACTION_POTENTIAL_INVOLVED_IN_CONTRACTION	0.003944176	CTNNA3/ANK2/KCNA5/FLNA/CACNA1C/SCN4B/KCNJ8/CACNB2/KCNE4/KCNH2/GJC1/CAV1/CACNA2D1/GJA1/RYR2/FGF12/SCN3B/SCN1B/SNTA1/GJA5/KCNJ5
GOMF_DELAYED_RECTIFIER_POTASSIUM_CHANNEL_ACTIVITY	GOMF_DELAYED_RECTIFIER_POTASSIUM_CHANNEL_ACTIVITY	0.003951455	KCNA1/KCNB1/KCNA5/KCNE4/KCNH2/KCNG1/KCNQ3/KCNS1
GOBP_POSITIVE_REGULATION_OF_MUSCLE_HYPERTROPHY	GOBP_POSITIVE_REGULATION_OF_MUSCLE_HYPERTROPHY	0.003976768	HAND2/AKAP6/IGF1/NR4A3/PDE9A/AGT/PDE5A/IL6ST/HAMP
GOBP_MYOBLAST_PROLIFERATION	GOBP_MYOBLAST_PROLIFERATION	0.003976768	KCNA5/IGF1/KLHL41/FGF7/ATOH8/HGF/SOX15

GOMF_AMYLOID_BETA_BINDING	GOMF_AMYLOID_BETA_BINDING	0.003972552	BCHE/APOA1/NGFR/GRIN2A/CRYAB/NLGN1/CD36/APBB1/TGFB2/CLSTN2/CACNA1A/ADRB2/GPRASP2/PRNP/APBA1/CLU/FCGR2B/LDLRAD3/LRP1/FZD4/BACE1/RAMP3/FPR2/DLGAP3
GOBP_REGULATION_OF_FIBROBLAST_MIGRATION	GOBP_REGULATION_OF_FIBROBLAST_MIGRATION	0.003984503	MIR145/ACTA2/HAS1/DDR2/PAK3/FGF2/AKAP12/THBS1/SLC8A1
GOBP_REGULATION_OF_WATER_LOSS_VIA_SKIN	GOBP_REGULATION_OF_WATER_LOSS_VIA_SKIN	0.00399252	CLDN4/LSR/PLEC/FLG/KLF4/STMN1/CYP26B1/ELOVL1/IL18/SFN/KDF1/FA2H/ALOX12B/ABCA12/KRT16
GOCC_HIGH_DENSITY_LIPOPROTEIN_PARTICLE	GOCC_HIGH_DENSITY_LIPOPROTEIN_PARTICLE	0.004041536	APOA2/APOA1/APOC3/APOH/APOB

GOBP_PEPTIDYL_LYSINE_TRIMETHYLATION	GOBP_PEPTIDYL_LYSINE_TRIMETHYLATION	0.004047875	OGT/IWS1/PWP1/KMT5B/SETD1B/BRD4/ZNF335/SIRT1/SETD4/NSD3/EEF2KMT/NFYA/SUV39H2/NFYC/SETD2/CTR9/SMYD5/SETD1A/TET2/SMARCB1/PIH1D1/TET3/LMNA/KMT2D/H1-2/KDM4C/SUV39H1/H1-3/KMT5C/ANTKMT/KDM4A/CSKMT/H1-4
GOBP_RECEPTOR_CLUSTERING	GOBP_RECEPTOR_CLUSTERING	0.004077875	RELN/FRRS1L/CHRDL1/NRXN2/GRIK5/GLRB/CDH2/FLNA/PTN/NLGN1/DLG2/DLG4/COLQ/THY1/NLGN2/MUSK/GSN
GOBP_HETEROPHILIC_CELL_CELL_ADHESION_VIA_PLASMA_MEMBRANE_CELL_ADHESION_MOLECULES	GOBP_HETEROPHILIC_CELL_CELL_ADHESION_VIA_PLASMA_MEMBRANE_CELL_ADHESION_MOLECULES	0.004087414	CADM3/CDH2/HMCN1/NLGN1/PTPRD/TENM3/FAT4/SELP/CRB2/TENM1/ITGA5/DCHS1/TENM4/SCARF2/SELE/LGALS7B/AMIGO2/IGSF21/CADM1/AMIGO1

GOBP_POSITIVE_REGULATION_OF_AXONOGENESIS	GOBP_POSITIVE_REGULATION_OF_AXONOGENESIS	0.004084443	SLIT2/CHODL/MAP1B/MAP6/NGF/PLXNA4/DIXDC1/CXCL12/NTN1/MAPT/RND2/LPAR3/L1CAM/FN1/CRABP2/NEFL/ISLR2/ROBO1/NRP1/AMIGO1/PLXNC1/DISC1/NTRK2/SEMA5A
GOBP_POSITIVE_REGULATION_OF_PROTEIN_SERINE_THREONINE_KINASE_ACTIVITY	GOBP_POSITIVE_REGULATION_OF_PROTEIN_SERINE_THREONINE_KINASE_ACTIVITY	0.004092873	PLA2G1B/ELANE/ADIPOQ/SLC8A2/HTR2A/GHR/EPGN/FERMT2/HTR2B/ROR2/FGF2/IGF2/ATP2B4/THBS1/CCL19/FZD10/FGFR1/TENM1/PKD2/DKK1/ADRB2/NTF3/LRRK2/PDGFD/FGF1/CAB39L/BMPR1B/NOX4/PDE5A/MEF2C/FAM20A/TCL1A/S100A12/PDGFC/ROBO1/FLT3/IL34/KIT/PDGFRB/DIRAS1/FZD4/MAP3K12

GOBP_NEGATIVE_REGULATION_OF_SECRETION	GOBP_NEGATIVE_REGULATION_OF_SECRETION	0.004122039	SYT4/SPX/CARTPT/TACR2/SFRP1/ADIPOQ/KCNB1/SCT/PTGER3/CNR1/CHGA/FOXF1/PRKN/SYT11/FFAR4/CCN3/OXTR/CD200/GJA1/IL11/AVPR2/KLF7/INHBA/INHA/GABBR1/FCGR2B/IL13RA2/P2RY1
GOBP_MITOTIC_CYTOKINESIS	GOBP_MITOTIC_CYTOKINESIS	0.004133838	CHMP2B/NUP62/PDCD6IP/RTKN/CFL1/CHMP7/CHMP1A/CKAP2/VPS4B/CHMP5/ANK3/SPTBN1/KIF20A/STMN1/AURKB/KIF20B/KIF23/CENPA/EXOC6/CNTROB/TTC19/CIT/ANLN/INCENP/KIF4A/RACGAP1/CHMP4C/ESPL1/ECT2/PLK1/BIRC5/CEP55/CDCA8/TRIM36
GOBP_PSEUDOURIDINE_SYNTHESIS	GOBP_PSEUDOURIDINE_SYNTHESIS	0.004159756	TSR3/RPUSD3/NAF1/TRUB2/DKC1/TRUB1/PUS1/PUS7/PUSL1/RPUSD2/RPUSD4/GAR1/NHP2/RPUSD1

GOBP_AMELOGENESIS	GOBP_AMELOGENESIS	0.004162016	PERP/SLC4A2/ITGB6/TCIRG1/RELT/MSX2/TBX1/NECTIN1/KLK5/CNNM4/KLK4
GOMF_VOLTAGE_GATED_CALCIUM_CHANNEL_ACTIVITY	GOMF_VOLTAGE_GATED_CALCIUM_CHANNEL_ACTIVITY	0.004170624	CACNA1C/CACNG4/CNR1/CACNA1H/CACNB2/NCS1/PKD2/CACNA2D3/CACNA2D1/CACNA1A
GOBP_COLLAGEN_CATABOLIC_PROCESS	GOBP_COLLAGEN_CATABOLIC_PROCESS	0.004177743	PRTN3/MMP8/PRSS2/MMP3/ADAMTS3/MMP24/MMP2/MMP7/MMP19/MMP16/CTSK/MMP23B/FAP/MRC2/TMPRSS6
GOBP_NEGATIVE_REGULATION_OF_CELL_CYCLE_G2_M_PHASE_TRANSITION	GOBP_NEGATIVE_REGULATION_OF_CELL_CYCLE_G2_M_PHASE_TRANSITION	0.004186742	BRCC3/BRCA1/TOPBP1/MIIP/BARD1/DONSON/ZFYVE19/PINX1/RAD21/CCNG1/TAOK2/RINT1/FZR1/AURKB/RBBP8/CDC14B/NBN/CDK1/CHEK1/BLM/TICRR/CLSPN/CHMP4C/DTL/PLK1/ATF5/ORC1
GOBP_CARBOHYDRATE_PHOSPHORYLATION	GOBP_CARBOHYDRATE_PHOSPHORYLATION	0.004191325	PFKFB4/XYLB/GALK2/HKDC1/HK3/FCSK/IPPK/POMK/TKFC/KHK/ITPK1/PFKFB2/HK2

GOBP_TRANSLESION_SYNTHESIS	GOBP_TRANSLESION_SYNTHESIS	0.004208574	POLDIP2/RCHY1/MAD2L2/USP10/POLH/POLE2/VCP/USP1/POLD3/PARP10/PCLAF/POLD2/PCNA/DTL/POLD1/USP43
GOBP_DEOXYRIBONUCLEOTIDE_CATABOLIC_PROCESS	GOBP_DEOXYRIBONUCLEOTIDE_CATABOLIC_PROCESS	0.004208574	UPP1/MBD4/NEIL1/SMUG1/NUDT16/OGG1/GDA/NT5M/NEIL2/TDG/DPYD/NUDT15/NT5C/PNP/NTHL1/XDH/NUDT18/UNG/DUT/DCTPP1/TYMP/ADA
GOBP_CELLULAR_RESPONSE_TO_VITAMIN_D	GOBP_CELLULAR_RESPONSE_TO_VITAMIN_D	0.004209717	PENK/SFRP1/CYP24A1/MN1/KANK2/SNAI2
GOBP_INTESTINAL_ABSORPTION	GOBP_INTESTINAL_ABSORPTION	0.004214819	PNLIP/APOA2/CEL/APOA1/SOAT2/CD36
GOBP_POSTSYNAPSE_ASSEMBLY	GOBP_POSTSYNAPSE_ASSEMBLY	0.004226307	NRXN2/GAP43/CDH2/NLGN4X/NLGN1/PTPRD/NPTX1/NLGN4Y/NLGN3/NLGN2
GOMF_WNT_PROTEIN_BINDING	GOMF_WNT_PROTEIN_BINDING	0.004237268	SFRP1/SFRP2/ROR2/FRZB/SFRP4/RECK/FZD10/TRABD2B/APCDD1L/FZD7/WLS/CTHRC1/ROR1/FZD4/FZD1/FZD8

GOBP_TISSUE_REMODELING	GOBP_TISSUE_REMODELING	0.004236559	CARTPT/SFRP1/HAND2/TPH1/THBS4/FGF10/RSPO3/FLNA/DDR2/PTN/TF/GREM1/PTH1R/AGT/LEPR/TGFB3/PDK4/CAV1/CSPG4/EDNRA/TNFRSF11B/MMP2/TMEM119/IL6/ADRB2/MIR27B/IGFBP5/CTSK/GJA1/FOXC2/S1PR1/GPNMB/NOX4/MEF2C/LTBP3/ITGB3/CTHRC1/TIMP1/HAMP/SEMA3C/AXL
GOBP_REGULATION_OF_MITOCHONDRIAL_GENE_EXPRESSION	GOBP_REGULATION_OF_MITOCHONDRIAL_GENE_EXPRESSION	0.004246011	METTL8/NGRN/MALSU1/ALKBH1/CDK5RAP1/FASTKD3/TRMT10C/PRKAA1/UQCC2/NSUN3/FASTKD2/RMND1/THAP11/MTG2/RCC1L/RPUSD3/TRUB2/TACO1/MTG1/MPV17L2/LRPPRC/RPUSD4/MRPS27/CHCHD10/C1QBP/SHMT2

GOBP_REGULATION_OF_CARDIAC_CONDUCTION	GOBP_REGULATION_OF_CARDIAC_CONDUCTION	0.004260027	NKX2-5/ANK2/SLC8A2/TRPC1/AGT/ATP2B4/SLC8A1/CORIN/RYR2/TBX5
GOBP_REPRODUCTIVE_SYSTEM_DEVELOPMENT	GOBP_REPRODUCTIVE_SYSTEM_DEVELOPMENT	0.004274054	AFP/SFRP1/MYOCD/FGF10/AMHR2/GFRA1/SLIT2/PGR/PTPRN/AR/SFRP2/NOG/SLIT3/ROR2/CYP1B1/ZFPM2/TNC/TEX11/WT1/GLI3/PTX3/GREB1L/ADAMTS1/GLI1/SERPINF1/CYP7B1/ZFP42/TGFB2/SERPINE2/RBP4/MMP2/ESR1/MMP19/SHH/PDGFRA/OSR1/DHH/CGA/FOXF2/A2M/GDF9/GDF7/RETN/SOX15/WNT2B/TCF21/ID4

GOBP_NEGATIVE_REGULATION_OF_GROWTH	GOBP_NEGATIVE_REGULATION_OF_GROWTH	0.004287125	NPPB/SFRP1/EPHA7/FHL1/RERG/CRYAB/SLIT2/DACT3/SFRP2/NOG/KCNK2/GPC3/PI16/SLIT3/RGS4/FRZB/GREM1/MEG3/WT1/SEMA3E/NTN1/AGT/FGF13/RGS2/NPR1/TGFB2/SERPINE2/RBP4/BDKRB1/WFDC1/DAB2/CGA/ADRB2/SPART/IGFBP5/CCN3/GJA1/SEMA6A/SEMA6D/SOX17/HSPA1A/INHBA/PTCH1/SEMA6C/TBX5/SEMA3G/MAP2/SEMA5B/SEMA3D/TSPYL2/STC2/SEMA3C/MYOZ1/DCBLD2/NRP1
GOBP_POSITIVE_REGULATION_OF_SUBSTRATE_ADHESION_DEPENDENT_CELL_SPREADING	GOBP_POSITIVE_REGULATION_OF_SUBSTRATE_ADHESION_DEPENDENT_CELL_SPREADING	0.004300708	APOA1/MYOC/FGA/FGG/FERMT2/LIMS2/FLNA/DAB2/ILK/HAS2/FGB/ITGB3/MYADM/NRP1
GOBP_RELAXATION_OF_MUSCLE	GOBP_RELAXATION_OF_MUSCLE	0.004313616	ATP1A2/PLN/KCNMA1/GUCY1A1/HRC/PRKG1/CHGA/RGS2/SLC8A1/SLN/PDE5A/P2RY1/GSN/GSTM2

GOMF_ANTIOXIDANT_ACTIVITY	GOMF_ANTIOXIDANT_ACTIVITY	0.004346368	ALB/MPO/HP/S100A9/APOA4/HBB/SOD3/GPX3/TPO/GPX7/PTGS1/CLIC2/GPX8/NXN/LTC4S/HBA2
GOCC_KINETOCHORE_MICROTUBULE	GOCC_KINETOCHORE_MICROTUBULE	0.004358619	CHMP6/CHMP2B/RAB11A/CHMP7/CHMP1A/CHMP5/ZW10/KNTC1/ZWILCH/KIF18A/CHMP4C/CENPE
GOMF_ANTIGEN_BINDING	GOMF_ANTIGEN_BINDING	0.004357885	HLA-DPB1/IGLV1-40/MAML1/IGHV3-23/KLRC1/KLRD1/IGHV4-4/IGHV1-3/IGHV4-59/IGLC6/NCR3LG1/TRAV12-3/HLA-H/TAPBP/HLA-DPA1/IGHV4-61/JCHAIN/IGHV3-30/HLA-E/TRDC/HLA-DRA/HLA-B/KLRC2/HLA-A/TRAV23DV6/IGLL5/HLA-DQA1/IGLV2-11/IGLV3-1/IGHA1/HLA-DRB5/DHCR24/HLA-F/HLA-C/IGHD/IGLV3-21/IGHV3-72/TAP2/IGHV3-64D/HLA-DRB1/IGHV3-33/SLC7A5/HLA-DQB1/IGHV3-48/IGHV3-66/IGLV2-14/IGKV1-16/LAG3/IGHA2/IGHV1-58/IGLV3-27/IGHM/ABO/IGHV3-49/TAP1/IGKV1D-12/IGKV5-2/IGHV3-13/IGLL1

GOBP_ANTERIOR_POSTERIOR_PATTERN_SPECIFICATION	GOBP_ANTERIOR_POSTERIOR_PATTERN_SPECIFICATION	0.004362727	CER1/SFRP1/NEUROD1/MEOX2/ALDH1A2/BARX1/DLL3/SIX2/SFRP2/NOG/GPC3/ZBTB16/HOXD10/CDON/WT1/GLI3/FOXF1/HOXA4/CRB2/NODAL/TBX18/HEYL/TDRD10/HOXA2/SHH/SMO/OSR1/PBX1/DKK1/PBX3/MEOX1/WNT2B/FOXC2/HOXD4/TCF15/SOX17
GOBP_MEIOSIS_I_CELL_CYCLE_PROCESS	GOBP_MEIOSIS_I_CELL_CYCLE_PROCESS	0.004384093	ING2/MSH6/PSMC3IP/EHMT2/FANCM/SLX4/RAD54B/EME1/MUS81/BRCA2/RAD21/SGO1/FBXO5/SIRT7/NDC80/RMI1/AURKA/RAD51AP1/CDC25C/DMC1/CKS2/PTTG1/EME2/CENPX/CCNB2/NDC1/RAD51/FANCD2/ESPL1/MAPK15/PLK1/TOP2A/RAD54L/CDC25A/CCNE2/SYCE2/BRIP1/REC8/TEX19

GOBP_NUCLEOSIDE_TRIPHOSPHATE_METABOLIC_PROCESS	GOBP_NUCLEOSIDE_TRIPHOSPHATE_METABOLIC_PROCESS	0.00438234	OGDH/GALK1/NDUFB5/NDUFC1/EIF6/NDUFA1/DHTKD1/MFN1/APP/PGAM4/ENPP3/SLC2A6/NDUFB4/ATP5MG/RHOA/NDUFS8/NME1/TIGAR/NME3/PRKAG1/NDUFB2/ATP5MF/DGUOK/NDUFA8/ATP6V1A/PSEN1/NUDT5/ATP5F1C/NDUFA6/CMPK2/NDUFB10/ATP5PD/NDUFS5/ENTPD3/ITPA/ENO2/NDUFB3/NDUFA12/GNAI3/NDUFA5/FOXK2/RPTOR/ATP6V1B2/FOXK1/GPD1/NDUFA9/NDUFB6/ZBTB7A/BAD/HSPA8/NDUFA11/ENTPD7/PARG/NDUFS6/NUDT15/GTPBP1/NDUFA10/NDUFS2/FIGNL1/ATP5F1EP2/NME6/HKDC1/SPHK2/NDUFA2/ADPGK/NDUFS1/NDUFS3/SDHD/CLPX/NDUFB7/IMPDH2/SMPDL3A/ATP5PO/NDUFAB1/ENTPD4/HK3/MTOR/ADK/UCK2/ATP5MC1/MLST8/ATP5PB/NDUFA13/NMNAT1/LDHA/PARP1/NUDT2/UQCC3/RAN/AK1/ATP5I[illegible]
GOBP_REGULATION_OF_PLATELET_AGGREGATION	GOBP_REGULATION_OF_PLATELET_AGGREGATION	0.004411189	CELA2A/CTSG/FGG/ADAMTS18/MMRN1/PRKG1/SERPINE2/IL6/PDPN/BLK/IL6ST
GOBP_MITOTIC_G2_M_TRANSITION_CHECKPOINT	GOBP_MITOTIC_G2_M_TRANSITION_CHECKPOINT	0.004425028	BRCC3/BRCA1/TOPBP1/BARD1/DONSON/CCNG1/TAOK2/RINT1/FZR1/RBBP8/CDC14B/NBN/CDK1/CHEK1/BLM/TICRR/CLSPN/DTL/PLK1/ORC1

GOBP_G_PROTEIN_COUPLED_RECEPTOR_SIGNALING_PATHWAY_INVOLV	GOBP_G_PROTEIN_COUPLED_RECEPTOR_SIGNALING_PATHWAY_INVOLVED_IN_HEART_PROC	0.004428118	PLN/CHGA/ATP2B4/CAV1/EDNRA/RGS2
GOMF_LIPASE_ACTIVITY	GOMF_LIPASE_ACTIVITY	0.004423991	PLA2G1B/PNLIP/PNLIPRP1/CEL/PNLIPRP2/PLCD4/CES1/PLA2G5/CLC/PLA2G2C/PLA2G12B/PLA2G4C/EDNRA/PLCL1
GOCC_NEURON_PROJECTION_MEMBRANE	GOCC_NEURON_PROJECTION_MEMBRANE	0.004472951	KCNB1/ITGA8/MAPT/HPCA/LAMP5/SGCE/MPP2/EPB41L3/THY1/GABBR1/RIPOR2/GPER1/GABRE
GOBP_NEGATIVE_REGULATION_OF_TRANSMEMBRANE_TRANSPORT	GOBP_NEGATIVE_REGULATION_OF_TRANSMEMBRANE_TRANSPORT	0.004505163	CASQ2/ATP1A2/ACTN2/PLN/GRP/RGS4/KCNE4/KCNH2/THBS1/PID1/CAV1/GEM/RGS2/PKD2/PRKCB/OSR1/REM1/SLN/MIR326/RRAD/CLIC2/TWIST1

GOBP_EMBRYONIC_HEART_TUBE_DEVELOPMENT	GOBP_EMBRYONIC_HEART_TUBE_DEVELOPMENT	0.004515153	HAND1/HAND2/NKX2-5/ASB2/PLXNA4/EDNRA/NODAL/PKD2/SHH/SMO/FOXC2/PIFO/SOX17/RYR2/MEF2C/VANGL2/ACVR1/NDRG4/APLNR/MICAL2/PITX2/ENG/DLL1/NPHP3/SOX18/TEAD2/NOTCH2/ARL13B/CITED2/TGFBR2/TBX2/EDN1
GOBP_POSITIVE_REGULATION_OF_CANONICAL_WNT_SIGNALING_PATHWAY	GOBP_POSITIVE_REGULATION_OF_CANONICAL_WNT_SIGNALING_PATHWAY	0.004581339	SFRP1/FGF10/MIR145/RSPO1/SFRP2/GPC3/RSPO3/FGF2/SFRP4/DAAM2/RECK/RSPO2/CAV1/DACT1/LRRK2/WNT10B/ZEB2/ADGRA2/ILK/DKK2/EDA/WLS
GOMF_TRANSFERASE_ACTIVITY_TRANSFERRING_NITROGENOUS_GROUPS	GOMF_TRANSFERASE_ACTIVITY_TRANSFERRING_NITROGENOUS_GROUPS	0.004622821	GOT2/BCAT2/GFPT1/GAPDH/GOT1/PSAT1/BCAT1/GPT2/GPT

GOCC_RECEPTOR_COMPLEX	GOCC_RECEPTOR_COMPLEX	0.00464763	PLP1/GRIK3/CHRNA3/ADCYAP1R1/EPHA7/AMHR2/GRIN2A/GFRA1/HTR2A/GHR/GRIK5/HTR2B/GFRA3/EPHA3/ROR2/FLNA/DDR2/ITGA7/P2RX2/TF/GABBR2/CACNG4/PLXNA4/GFRA2/PTH1R/TRPC1/NLGN1/LEPR/CD36/ITGA8/ITGA9/CUBN/ROS1/CHRNB4/ITGBL1/VLDLR/FGFR1/NPR1/ITGA1/NRP2/TNFRSF11B/ITLN1/ITGA5/GPBAR1/IL6/NR1H4/EMILIN1/PDGFRA/ADRB2/CRLF1/TSPAN32/DLG4/CPT1C/BMPR1B/MTTP/RAMP2/TGFBR3/TEK/LY96/GABBR1/GABRR1/ITGB3/IL13RA2/CHRNB2/TRBV23-1/FLT3/TRAV35/LIFR/APBB1IP/MUSK/ROR1/ITGA11/IL6ST/KIT/ACVR1/PDGFRB/LRP1/GABRE/AXL/TLR7/TRIL/NRP1/RAMP3/GPR20/MERTK/PRLR/TIE1/CSF2/OLR1/FGFR2/CNTFR/RAMP1/PLXDC1/PLA2R1/GPR63/GPR37
GOBP_MESENCHYMAL_CELL_MIGRATION	GOBP_MESENCHYMAL_CELL_MIGRATION	0.004652299	SOX10/HAND2/HTR2B/CDH2/SEMA3E/RADIL/EDNRA/NRP2/SHH/SMO/FN1/ISL1/SEMA6A/SEMA6D/ANXA6/TWIST1/SEMA6C/SEMA3G/SEMA5B/SEMA3D/ACVR1/SEMA3C/EDNRB/NRP1

GOBP_NEGATIVE_REGULATION_OF_CELL_DEVELOPMENT	GOBP_NEGATIVE_REGULATION_OF_CELL_DEVELOPMENT	0.004676846	SYT4/SOX10/EPHA7/F2/DLL3/IGF1/NOG/FBLN1/PTN/PRTG/FRZB/DAB1/DAAM2/SEMA3E/NTN1/NFATC4/NKX6-1/FGF13/S1PR3/NODAL/FBN1/IL6/SPART/EFNB3/DPYSL5/ID4/ATOH1/SEMA6A/CCL11/PTHLH/SEMA6D/PAEP/THY1/NPR2/SEMA6C/SEMA3G/MAP2/SEMA5B/SEMA3D/SEMA3C/EDNRB/NRP1/NPPC
GOBP_ONE_CARBON_METABOLIC_PROCESS	GOBP_ONE_CARBON_METABOLIC_PROCESS	0.004719048	MAT2B/MTHFD2L/ALDH1L1/MTHFR/FPGS/CA13/DHFRP1/SFXN1/SHMT1/MTHFD2/MTHFD1L/DHFR/MTHFD1/SHMT2/TYMS/CA1/CA2/AHCYL2/CA9

GOBP_FIBROBLAST_GROWTH_FACTOR_RECEPTOR_SIGNALING_PATHWAY	GOBP_FIBROBLAST_GROWTH_FACTOR_RECEPTOR_SIGNALING_PATHWAY	0.004721411	NGFR/FGF10/TRIM71/FLRT2/FGF7/NOG/SMOC2/FGF2/THBS1/HHIP/FGFR1/FLRT1/KLB/CCN2/FGF1/MIR149/KL/FGF12/SULF1/SHISA2
GOMF_HORMONE_ACTIVITY	GOMF_HORMONE_ACTIVITY	0.004736114	GCG/UCN3/SPX/CARTPT/GHRH/VIP/NPPB/PENK/ADIPOQ/ADCYAP1/SCT/RETNLB/IGF1/GRP/FNDC5/TTR/NPY/IGF2/THPO/GIP/AGT/FBN1/MLN/GUCA2A/CGA/RETN/PTHLH/INSL6/STC1/KL/INHBA/INHA

GOBP_VIRAL_GENE_EXPRESSION	GOBP_VIRAL_GENE_EXPRESSION	0.004742903	DENR/CCNT1/EIF2D/EIF3B/TARDBP/PSMC3/EIF3D/ATG5/MAN2A1/PARP9/USF1/MON1B/GSK3A/SMARCB1/MAN1B1/MGAT5/PARP16/DHX9/MOGS/EIF3A/EIF3G/ST6GALNAC4/CANX/TARBP2/SP1/FUT8/RRP1B/CTDP1/TRIM21/TRIM27/TRIM11/MGAT2/CCL5/PRMT1/PTBP1/HDAC1/TFAP4/TRIM14/PARP10/MGAT4B/TRIM31/FURIN/SMARCA4/CCL3/ST6GALNAC2/POU2F3
GOBP_MITOCHONDRIAL_DNA_REPLICATION	GOBP_MITOCHONDRIAL_DNA_REPLICATION	0.004754298	POLG/RRM2B/DNAJA3/STOML2/TWNK/RRM1/DNA2/ENDOG
GOBP_PHYSIOLOGICAL_CARDIAC_MUSCLE_HYPERTROPHY	GOBP_PHYSIOLOGICAL_CARDIAC_MUSCLE_HYPERTROPHY	0.004776353	AKAP6/IGF1/PI16/RGS4/AGT/RGS2/SORBS2
GOBP_MITOTIC_G2_DNA_DAMAGE_CHECKPOINT_SIGNALING	GOBP_MITOTIC_G2_DNA_DAMAGE_CHECKPOINT_SIGNALING	0.004789132	BRCC3/BRCA1/TOPBP1/DONSON/CCNG1/TAOK2/RINT1/FZR1/CDC14B/NBN/CDK1/CHEK1/BLM/TICRR/CLSPN/DTL/PLK1

GOBP_ADENYLATE_CYCLASE_ACTIVATING_ADRENERGIC_RECEPTOR_SI	GOBP_ADENYLATE_CYCLASE_ACTIVATING_ADRENERGIC_RECEPTOR_SIGNALING_PATHWAY	0.004816561	PLN/ADRA1D/CHGA/ADRA1B/ATP2B4/ADRB2
GOMF_RNA_METHYLTRANSFERASE_ACTIVITY	GOMF_RNA_METHYLTRANSFERASE_ACTIVITY	0.004826875	BCDIN3D/HENMT1/METTL8/CMTR1/TRMT13/METTL5/METTL14/METTL16/PCIF1/LCMT2/TRMT61A/TRMT10C/TRMT2B/TRMT5/NSUN6/DIMT1/NSUN3/METTL3/TARBP1/ALKBH8/TYW3/BUD23/THUMPD3/THUMPD2/TFB2M/TRMO/TRMT61B/FTSJ1/FTSJ3/MEPCE/METTL15/NSUN4/TRMT11/METTL2A/METTL2B/TRMT10A/ZCCHC4/FDXACB1/TRMT2A/NSUN2/NSUN5/FBL/MRM1/CMTR2/WDR4/TGS1/MRM3/TFB1M/TRMT44/TRMT1
GOBP_HIGH_DENSITY_LIPOPROTEIN_PARTICLE_REM	GOBP_HIGH_DENSITY_LIPOPROTEIN_PARTICLE_REMODELING	0.004856639	APOA2/APOA1/APOC3
GOBP_REGULATION_OF_THE_FORCE_OF_HEART_CONTRACTION	GOBP_REGULATION_OF_THE_FORCE_OF_HEART_CONTRACTION	0.004866473	ATP1A2/PLN/MYL3/CHGA/ATP2B4/CAV1/SLC8A1/RYR2/EDN2/MYL4
GOBP_NEGATIVE_REGULATION_OF_PHAGOCYTOSIS	GOBP_NEGATIVE_REGULATION_OF_PHAGOCYTOSIS	0.004866473	PRTN3/ADIPOQ/PIP4P2/SYT11

GOCC_TELOMERASE_HOLOENZYME_COMPLEX	GOCC_TELOMERASE_HOLOENZYME_COMPLEX	0.00490149	TEP1/SMG6/NOP10/SMG7/SMG5/ACD/SNRPB/NAT10/DKC1/HNRNPU/HNRNPC/PTGES3/GAR1/WRAP53/NHP2/TERT
GOBP_INOSITOL_LIPID_MEDIATED_SIGNALING	GOBP_INOSITOL_LIPID_MEDIATED_SIGNALING	0.004912648	RELN/MYOC/PLCD4/NPR3/F2/HTR2A/IGF1/HTR2B/ROR2/FGF2/DCN/SEMA3E/TNFAIP8L3/AGT/CSF3/SELP/HGF/FGFR1/ANGPT1/TGFB2/SERPINE2/PLCL1/PDGFRA/PTPN13/FN1/PREX2/PDGFD/FBXL2/CRNN/TEK/PDGFC/TWIST1/FLT3/GPER1/ROR1/KIT/PDGFRB

GOBP_DEFENSE_RESPONSE_TO_BACTERIUM	GOBP_DEFENSE_RESPONSE_TO_BACTERIUM	0.004909952	PLA2G1B/ELANE/MPO/REG3G/AZU1/DEFA4/HP/RNASE3/BPI/FGA/CTSG/LYPD8/F2/RNASE2/SSC5D/TF/COLEC12/GBP6/DEFA6/CXCL6/PRKD1/CD36/CHGA/CAMP/S100A8/SELP/C10orf99/IL6/IGHV1-69D/S100A9/NR1H4/SERPINE1/EMILIN1/DEFB126/PGLYRP1/SYT11/IGHV1-69-2/IGHG4/LYG2/WFDC3/IGHV6-1/RARRES2/FGB/WFDC10B/IGHV7-4-1
GOCC_IMMUNOLOGICAL_SYNAPSE	GOCC_IMMUNOLOGICAL_SYNAPSE	0.004942996	BCL10/PDCD6IP/LGALS3/CORO1A/ARHGDIA/ALCAM/IL4I1/HLA-DRA/GZMA/SOCS6/STOML2/PRKCQ/SCRIB/HLA-DRB1/PTPRJ/ICAM1/LCK/EZR/GZMB

GOBP_SKELETAL_SYSTEM_MORPHOGENESIS	GOBP_SKELETAL_SYSTEM_MORPHOGENESIS	0.004944539	CER1/COL2A1/SFRP1/MGP/PAPPA2/GHR/SIX2/NOG/NKX3-2/GREM1/SFRP4/HOXD10/FREM1/GLI3/ZEB1/RAB23/TGFB3/HOXA4/HHIP/SOX5/NODAL/FGFR1/MMP2/TMEM119/COMP/COL3A1/HOXA2/PDGFRA/OSR1/MMP16/PRRX1/WNT10B/FOXC2/CCN2/HOXD4/TCF15/HAS2/BMPR1B/TBX15/STC1/MEF2C/LTBP3/ANXA6/TWIST1/HOXA5/HOXD9/COL1A1/NPPC/FOXN3/FGFR2/HOXA3/SOX11/BMP6/HOXD8/FUZ/PKD1
GOMF_TELOMERIC_DNA_BINDING	GOMF_TELOMERIC_DNA_BINDING	0.0049887	XRCC6/TP53BP1/RPA2/SMG7/NABP2/TERF2/SMG1/SMG5/XRCC5/ACD/RPA1/STN1/NCL/HNRNPD/PIF1/KDM1A/UPF1/HNRNPA2B1/WRN/TELO2/HNRNPA1/BLM/TERT

GOMF_PRENYLTRANSFERASE_ACTIVITY	GOMF_PRENYLTRANSFERASE_ACTIVITY	0.005010744	FNTA/PGGT1B/UBIAD1/NUS1/RABGGTB/FDPS/PDSS2/RABGGTA/FDFT1/PDSS1/DHDDS/COX10/COQ2
GOCC_PROTEASOME_ACCESSORY_COMPLEX	GOCC_PROTEASOME_ACCESSORY_COMPLEX	0.005022018	PSMD1/PSMC6/PSMD4/PSME3/PSMD5/PSMD6/PSMC5/PSMD13/PSMD7/PSMC3/PSMC2/PSMC4/PSMD2/PSMD9/PSMD8/PSMC1/PSMD12/PSMD14/ADRM1/PSME1/PSME2/PSMD3
GOBP_VASCULAR_ENDOTHELIAL_GROWTH_FACTOR_SIGNALING_PATHWAY	GOBP_VASCULAR_ENDOTHELIAL_GROWTH_FACTOR_SIGNALING_PATHWAY	0.005030705	SMOC2/DCN/PRKD1/ADAMTS3/NRP2/MIR342/PDGFRA/CCBE1/SEMA6A/ADGRA2/PTP4A3/HSPB1/VEGFC/ROBO1/FLT3/PDGFRB/GAB1/NRP1

GOCC_MEDIATOR_COMPLEX	GOCC_MEDIATOR_COMPLEX	0.005072006	MED20/MED6/CDK9/MED4/MED31/MED28/MED22/MED10/CDK4/MED30/MED12/MED8/MED11/MED27/CDK19/BCLAF1/MED13/MED14/THRAP3/CCNC/MED25/MED23/MED16/MED15/MED9/PPARGC1B/MED26/MED1/MED18/CDK6/MED24
GOBP_LEUKOCYTE_CHEMOTAXIS	GOBP_LEUKOCYTE_CHEMOTAXIS	0.005082154	PLA2G1B/AZU1/CCL16/TAFA4/THBS4/CTSG/SLIT2/CCL26/PTN/GREM1/JAM3/CXCL6/CXCL12/F7/CHGA/CCL21/ITGA9/CH25H/THBS1/CCL19/S100A8/CX3CR1/CNR2/C10orf99/CYP7B1/TGFB2/ITGA1/MMP2/IL6/S100A9/SERPINE1/CCL2/FOLR2/GPR183/CCN3/CCL11/S1PR1/DUSP1/BST1/CXCR2/CD300H/RARRES2/CAMK1D/SCG2/CXCR1/S100A12/RIPOR2/VEGFC/MPP1/CXCR4/CCR7/IL34/KIT/FFAR2/EDNRB/TNFSF18/EDN2/FPR2

GOBP_TRIGLYCERIDE_METABOLIC_PROCESS	GOBP_TRIGLYCERIDE_METABOLIC_PROCESS	0.005101182	APOA2/PNLIPRP2/APOA1/APOC3/APOH/APOB/RGN/THRSP/TNXB/GPIHBP1/CAV1/APOA4/LPL/MTTP/PNPLA1/GPLD1/MOGAT1
GOBP_REGULATION_OF_STRIATED_MUSCLE_CONTRACTION	GOBP_REGULATION_OF_STRIATED_MUSCLE_CONTRACTION	0.005111701	CASQ2/ATP1A2/PLN/CASQ1/NKX2-5/CTNNA3/ANK2/DMD/MYL3/CACNA1C/HRC/CHGA/ADRA1B/DMPK/CAV1/FGF13/RGS2/SLC8A1/CCN2/RYR2/STC1/CLIC2/PDE5A
GOMF_STEROID_BINDING	GOMF_STEROID_BINDING	0.005134965	APOA2/APOA1/APOC3/ATP1A2/APOD/PGR/AR/SOAT2/SERPINA6/CAV1/SHBG/ESR1/ESRRG/NR1H4/APOA4/GPR183/HSD11B1/ABCA1/KL/PTCH1/NR3C1/ANXA6/CYP3A4/SYP/GPER1

GOBP_MITOCHONDRIAL_RNA_PROCESSING	GOBP_MITOCHONDRIAL_RNA_PROCESSING	0.005209064	FASTKD3/TRMT10C/TRMT5/PRORP/PNPT1/TRNT1/FASTKD2/TRIT1/MTO1/FASTKD5/TRMT61B/HSD17B10/FASTK/FASTKD1/PUS1/SUPV3L1/RPUSD4/TBRG4/ELAC2
GOBP_PROTEIN_EXPORT_FROM_NUCLEUS	GOBP_PROTEIN_EXPORT_FROM_NUCLEUS	0.005234821	CSE1L/RAPGEF3/EMD/NXT1/RBM22/DESI1/DUSP16/CTDSPL2/XPO5/NUP214/TPR/SMURF1/TCF7L2/XPO4/NUTF2/RANBP3/PARK7/XPO1/IFI27/TXN/FRAT1/CALR/IL1B/XPO6/BARD1/YWHAE/ANP32B/RAN/XPO7/CCHCR1/HSPA9/CDK5/RANGAP1/SIRT7/UHMK1/SIRT6/CHP1/SFN

GOBP_NEUTROPHIL_MIGRATION	GOBP_NEUTROPHIL_MIGRATION	0.005235712	PLA2G1B/PRTN3/CCL16/THBS4/SLIT2/CCL26/JAM3/CXCL6/CCL21/ITGA9/GP2/CCL19/S100A8/IL1R1/TGFB2/ITGA1/S100A9/CCL2/CCL11/BST1/CXCR2/CD300H/CAMK1D/CXCR1/S100A12/RIPOR2/CD99L2/MPP1/CCR7
GOBP_CELL_FATE_SPECIFICATION	GOBP_CELL_FATE_SPECIFICATION	0.0052636	NKX2-2/FEV/ASCL1/PTCH2/AR/SIX2/SFRP2/FGF2/HOXD10/CDON/GLI3/NODAL/TBX18/SHH/SMO/DHH/DKK1/ISL1/FZD7/ATOH1/TENM4/TBX15/SOX17/TBX10/PTCH1/TBX5

GOBP_MULTI_MULTICELLULAR_ORGANISM_PROCESS	GOBP_MULTI_MULTICELLULAR_ORGANISM_PROCESS	0.005261207	TAC1/TACR1/ADCYAP1/PGR/AR/SLC38A3/PTGFR/FBLN1/TAC3/PTN/CNR1/GIP/AMBP/AGT/RECK/TGFB3/P2RX1/LDOC1/PLA2G4C/NDP/TRO/RGS2/NODAL/ENDOU/SCGB1A1/SERPINE2/FOSB/ITGA5/MMP2/ESR1/CORIN/AVPR1A/IGFBP7/IGFBP5/OXTR/EPYC/GJA1/COL16A1/DKKL1/SYDE1/PTHLH/THBD/STC1/PAPPA/ITGB3/CYP1A1/STOX2/STC2/TIMP1/OXT
GOCC_NUCLEAR_UBIQUITIN_LIGASE_COMPLEX	GOCC_NUCLEAR_UBIQUITIN_LIGASE_COMPLEX	0.005280986	ANAPC11/ANAPC7/PCGF2/ANAPC16/PCGF3/CUL7/CDC27/CDC26/PCGF1/ANAPC4/PCGF6/ANAPC1/CDC23/ANKRD39/ANAPC15/ANAPC5/ASB6/BRCC3/BRCA1/PCGF5/ANAPC2/BCOR/BARD1/BRCA2/CBX4/FZR1/CBX2/UBE2S/CBX8/BUB1B/CDC20/RAD51

GOBP_OLIGOSACCHARIDE_LIPID_INTERMEDIATE_BIOSYNTHETIC_PROCESS	GOBP_OLIGOSACCHARIDE_LIPID_INTERMEDIATE_BIOSYNTHETIC_PROCESS	0.00530032	SRD5A3/ALG2/ALG3/ALG8/RFT1/NUS1/DPAGT1/ALG10/ALG6/DOLPP1/DHDDS/ALG1/MPDU1/ALG10B
GOBP_POSTSYNAPSE_ORGANIZATION	GOBP_POSTSYNAPSE_ORGANIZATION	0.005299805	RELN/FRRS1L/INA/CHRDL1/EPHA7/NRXN2/GAP43/GLRB/CDH2/ZDHHC15/PAK3/NLGN4X/NLGN1/PTPRD/NPTX1/CNTNAP1/NLGN4Y/NRP2/KIF1A/NLGN3/LRRK2/LRRC4/CTNND2/TMEM108/DLG4/NEFL/PRNP/COLQ/ACTN1/NRCAM/NLGN2/FCGR2B/MUSK/ARC/NEFH/NRP1/LRRC4B
GOBP_REGULATION_OF_CARTILAGE_DEVELOPMENT	GOBP_REGULATION_OF_CARTILAGE_DEVELOPMENT	0.005310723	SIX2/GDF6/NOG/EFEMP1/NKX3-2/ZBTB16/FRZB/GREM1/GLI3/WNT9A/SOX5/CCN1/CTSK/SNAI2/CCN4/CCN2/PTHLH/MAF/BMPR1B/LTBP3

GOBP_EMBRYONIC_DIGESTIVE_TRACT_MORPHOGENESIS	GOBP_EMBRYONIC_DIGESTIVE_TRACT_MORPHOGENESIS	0.005346578	RBPMS2/FGF10/SIX2/GLI3/FOXF1/SHH/PDGFRA/TCF21
GOBP_COPII_COATED_VESICLE_BUDDING	GOBP_COPII_COATED_VESICLE_BUDDING	0.005354363	TRAPPC8/TRAPPC2/TRAPPC1/PPP6C/SEC24B/RAB1A/SAR1A/TMED10/SAR1B/TFG/CSNK1D/KLHL12/VAPB/TRAPPC12/SCAP/TMED2/MIA3/CUL3/TRAPPC4/TRAPPC11/TRAPPC2L/PDCD6/SEC24D/SEC24A/INSIG1/SEC31B/PREB/SEC13/SEC24C/SEC23B/SEC16A/MAPK15

GOBP_RESPONSE_TO_KETONE	GOBP_RESPONSE_TO_KETONE	0.00537209	UCN3/NKX2-2/SOX10/SFRP1/TACR1/ADCY5/SLIT2/AR/UGT3A2/SLIT3/ADCY2/PTGFR/CYP1B1/PRKAA2/ADCY1/F7/CCL21/FBXO32/TGFB3/THBS1/CCL19/SERPINF1/CAV1/FIBIN/TGFB2/KLF9/HPCA/FOSB/MIR342/AVPR1A/IGFBP7/A2M/OXTR
GOBP_ENDONUCLEOLYTIC_CLEAVAGE_INVOLVED_IN_RRNA_PROCESSING	GOBP_ENDONUCLEOLYTIC_CLEAVAGE_INVOLVED_IN_RRNA_PROCESSING	0.005387704	ABT1/BMS1/UTP20/RCL1/TSR1/NOP9/TBL3/RRS1/BOP1/NOP14
GOBP_REGULATION_OF_DEFENSE_RESPONSE_TO_VIRUS_BY_HOST	GOBP_REGULATION_OF_DEFENSE_RESPONSE_TO_VIRUS_BY_HOST	0.005400928	TOMM70/PARP9/HSP90AA1/APOBEC3G/DHX9/DTX3L/IL1B/PYCARD/TARBP2/ZDHHC11/PTPN22/IL23A/IL12RB1/TRAF3IP2/MMP12/STAT1/MICB/CGAS/ZC3H12A/AIM2

GOBP_RESPONSE_TO_INTERFERON_GAMMA	GOBP_RESPONSE_TO_INTERFERON_GAMMA	0.005409448	CCL15/VAMP3/NMI/TRIM21/ASS1/PDE12/EPRS1/CDC42EP4/TLR3/IL12RB1/CYP27B1/IRF8/ACTG1/GCH1/CCL5/MED1/RAB20/PARP14/CCL3L3/CXCL16/RAB43/SLC26A6/SOCS1/IFITM1/STAT1/CD74/CDC37/CX3CL1/CCL3/BST2/PPARG/GAPDH/TP53/LGALS9/CASP1/FLNB/GBP3/GBP1/NOS2/FASLG/CCL23/CIITA/NLRC5/IRF1/JAK2/IFNG/GBP5/GBP4
GOBP_CELLULAR_OXIDANT_DETOXIFICATION	GOBP_CELLULAR_OXIDANT_DETOXIFICATION	0.005441498	ALB/MPO/HP/FBLN5/CD36/S100A9/APOA4/HBB/SOD3/GPX3/TPO/GPX7/PTGS1/CLIC2/GPX8/H19/NXN/LTC4S/HBA2

GOBP_NEGATIVE_REGULATION_OF_ION_TRANSMEMBRANE_TRANSPORT	GOBP_NEGATIVE_REGULATION_OF_ION_TRANSMEMBRANE_TRANSPORT	0.005441498	CASQ2/ATP1A2/ACTN2/PLN/GRP/RGS4/KCNE4/KCNH2/CAV1/GEM/RGS2/PKD2/OSR1/REM1/SLN/RRAD/CLIC2/TWIST1/HAMP/KCNAB1
GOMF_EXTRACELLULAR_LIGAND_GATED_ION_CHANNEL_ACTIVITY	GOMF_EXTRACELLULAR_LIGAND_GATED_ION_CHANNEL_ACTIVITY	0.005449986	GRIK3/CHRNA3/GRIK1/GRIN2A/GRIK5/GLRB/P2RX2/CHRNB4/P2RX1/SLC1A7
GOMF_FOUR_WAY_JUNCTION_DNA_BINDING	GOMF_FOUR_WAY_JUNCTION_DNA_BINDING	0.005463321	XRCC2/MSH6/HMGB3/FANCM/MEN1/WRN/HMGB2/XRCC3/MSH2/GEN1/BLM
GOBP_EPITHELIAL_CELL_APOPTOTIC_PROCESS	GOBP_EPITHELIAL_CELL_APOPTOTIC_PROCESS	0.005461361	PLA2G1B/HAND2/NEUROD1/FGA/NKX2-5/FGG/SFRP4/THBS1/EDNRA/ANGPT1/IL6/ESR1/SERPINE1/CCL2/ATOH1/IL11/RAMP2/RYR2/SCG2/FGB/TEK/H19/CD248/GPER1/LEF1/ECSCR/NDNF/GSN

GOBP_PYRIMIDINE_DEOXYRIBONUCLEOTIDE_METABOLIC_PROCESS	GOBP_PYRIMIDINE_DEOXYRIBONUCLEOTIDE_METABOLIC_PROCESS	0.005491132	UPP1/MBD4/NEIL1/SMUG1/OGG1/NT5M/NEIL2/TDG/DPYD/CMPK2/DCTD/NT5C/NTHL1/UNG/DTYMK/DUT/DCTPP1/SHMT1/TYMP/TYMS
GOBP_REGULATION_OF_CARDIAC_MUSCLE_CELL_MEMBRANE_REPOLARIZATION	GOBP_REGULATION_OF_CARDIAC_MUSCLE_CELL_MEMBRANE_REPOLARIZATION	0.005493663	ANK2/KCNA5/KCNH6/FLNA/SCN4B/KCNE4/KCNH2/CACNA2D1
GOBP_NUCLEOSIDE_TRIPHOSPHATE_BIOSYNTHETIC_PROCESS	GOBP_NUCLEOSIDE_TRIPHOSPHATE_BIOSYNTHETIC_PROCESS	0.00553894	ATP5MC2/NDUFB8/DMAC2L/UCKL1/NDUFC2/NDUFB5/NDUFC1/NDUFA1/NDUFB4/ATP5MG/NDUFS8/NME1/NME3/NDUFB2/ATP5MF/DGUOK/NDUFA8/ATP6V1A/ATP5F1C/NDUFA6/CMPK2/NDUFB10/ATP5PD/NDUFS5/NDUFB3/NDUFA12/NDUFA5/NDUFA9/NDUFB6/NDUFA11/NDUFS6/NDUFA10/NDUFS2/ATP5F1EP2/NME6/SPHK2/NDUFA2/NDUFS1/NDUFS3/SDHD/NDUFB7/IMPDH2/ATP5PO/NDUFAB1/ADK/UCK2/ATP5MC1/ATP5PB/NDUFA13/PARP1/NUDT2/UQCC3/AK1/IMPDH1/SDHB/CAD/CTPS1/ATP5MC3/ATP5F1A/VCP/ANTKMT/SDHA/DTYMK/ATP5F1B/STOML2/CMPK1/ATP5ME/NDUFV1/AK7/NDUFS7/ATP5F1D/ENO1/TYMS/ALDOA

GOBP_PROTEIN_LOCALIZATION_TO_CHROMATIN	GOBP_PROTEIN_LOCALIZATION_TO_CHROMATIN	0.005549582	MCM8/H4C13/SETD2/WAPL/MACROH2A1/VCPIP1/RB1/H4C9/PIH1D1/H2BC11/CHMP7/H4C4/H4C3/RUVBL2/LRWD1/H4C2/MCM9/H4C1/RAD21/VRK1/VCP/SIRT6/CENPA/MSH2/EZH2/CDCA5/PLK1/ESCO2/H1-5
GOBP_REGULATION_OF_DNA_STRAND_ELONGATION	GOBP_REGULATION_OF_DNA_STRAND_ELONGATION	0.005578982	ACTR5/ACTL6A/UCHL5/INO80D/RUVBL1/INO80E/INO80/RUVBL2/MCRS1/NFRKB/INO80C
GOMF_OXIDOREDUCTASE_ACTIVITY_ACTING_ON_THE_CH_NH_GROUP_OF_DONORS	GOMF_OXIDOREDUCTASE_ACTIVITY_ACTING_ON_THE_CH_NH_GROUP_OF_DONORS	0.005589667	MTHFD2L/ALDH1L1/PYCR3/IL4I1/MTHFR/BLVRB/DHFRP1/MTHFD2/MTHFD1L/AIFM2/DHFR/PYCR1/MTHFD1/PRODH

GOBP_CELL_FATE_COMMITMENT	GOBP_CELL_FATE_COMMITMENT	0.005599859	NKX2-2/SFRP1/NEUROD1/FEV/NKX2-5/FGF10/ASCL1/PTCH2/GAP43/AR/SIX2/SFRP2/TNXB/EBF2/FGF2/GAS1/HOXD10/CDON/WT1/GLI3/TGFB1I1/WNT9A/EDNRA/SOX5/FGF13/NODAL/TBX18/ONECUT1/IL6/ZNF521/HOXA2/SHH/TAL1/SMO/DHH/PRRX1/DKK1/ISL1/FZD7/GDF7/WNT2B/WNT10B/FOXC2/ATOH1/TENM4/TBX15/SOX17/PDPN/TBX10/MEF2C
GOCC_PROTEASOME_CORE_COMPLEX	GOCC_PROTEASOME_CORE_COMPLEX	0.005657283	PSMA7/PSMB5/PSMB4/PSMF1/PSMB1/PSMA3/PSMB6/PSMB3/PSMB7/PSMA6/PSMB2/PSMA4/PSMA5/PSMB9/PSMB8/PSMB10
GOBP_NITRIC_OXIDE_MEDIATED_SIGNAL_TRANSDUCTION	GOBP_NITRIC_OXIDE_MEDIATED_SIGNAL_TRANSDUCTION	0.005661513	NEUROD1/GUCY1A1/AGT/CD36/ATP2B4/THBS1/GUCY1B1/RASD1/GUCY1A2/FPR1/PDE5A/NDNF

GOBP_REGULATION_OF_INSULIN_LIKE_GROWTH_FACTOR_RECEPTOR_SIGNALING_PATHW	GOBP_REGULATION_OF_INSULIN_LIKE_GROWTH_FACTOR_RECEPTOR_SIGNALING_PATHWAY	0.005660389	GHRH/IGF1/AR/CILP/IGFBP1/IGFBP6/IGFBP4/IGFBP5
GOBP_REGULATION_OF_HEART_RATE_BY_CARDIAC_CONDUCTION	GOBP_REGULATION_OF_HEART_RATE_BY_CARDIAC_CONDUCTION	0.005657548	CTNNA3/ANK2/KCNA5/KCNH6/CACNA1C/SCN4B/CACNB2/KCNE4/KCNH2/CAV1/CACNA2D1/ISL1
GOBP_NEUTRAL_LIPID_CATABOLIC_PROCESS	GOBP_NEUTRAL_LIPID_CATABOLIC_PROCESS	0.005657548	APOA2/PNLIPRP2/APOA1/APOC3/APOB/GPIHBP1/APOA4/LPL/PNPLA1/GPLD1
GOBP_POSITIVE_REGULATION_OF_ENDOCYTOSIS	GOBP_POSITIVE_REGULATION_OF_ENDOCYTOSIS	0.005678048	VTN/GPC3/CLIP3/TF/GREM1/SFRP4/NLGN1/CALY/CD36/CCL21/CCL19/ANGPT1/SERPINE1/DAB2/RAB31/SGIP1/NTF3/LRRK2
GOBP_REGULATION_OF_CHONDROCYTE_DIFFERENTIATION	GOBP_REGULATION_OF_CHONDROCYTE_DIFFERENTIATION	0.005701704	SIX2/GDF6/EFEMP1/NKX3-2/ZBTB16/GREM1/GLI3/WNT9A/SOX5/SNAI2/CCN4/CCN2/PTHLH/MAF/BMPR1B/LTBP3/TRPS1

GOMF_POSTSYNAPTIC_NEUROTRANSMITTER_RECEPTOR_ACTIVITY	GOMF_POSTSYNAPTIC_NEUROTRANSMITTER_RECEPTOR_ACTIVITY	0.00571516	CHRM2/GRIK3/CHRNA3/GRIK1/GRIK5/GLRB/CHRNB4/CHRM4/GABBR1/GABRR1/CHRNB2/GABRE/GRID1/CHRM3
GOMF_ACTIN_BINDING	GOMF_ACTIN_BINDING	0.005717237	CNN1/MYH11/SYNPO2/LDB3/ACTN2/FLNC/LMOD1/MYLK/KCNMA1/TAGLN/CTNNA3/KLHL4/SORBS1/TPM2/PDLIM3/TMOD1/TNS1/DMD/NEXN/CFL2/MYL3/CALD1/FERMT2/CAP2/PKNOX2/MAP1B/FLNA/SVIL/SMTN/MYOM1/MAP1A/DAAM2/DIXDC1/ADD2/WASF3/CORO6/CACNB2/TPM1/MAPT/PIP/PALLD/LMOD3/PDLIM4/HPCA/SYNE1/PRKN/MYH10/FAM107A/FHOD3/ANTXR1/LRRK2/PARVA/SYNPO/GAS7/PDLIM7/EPB41L3/KLHL5/PLEKHH2/TMSB15A/ACTN1/VASH2/MTSS1/DST/WASF1/ANXA6/CORO2B/PHACTR1/MYRIP/DSTN/CXCR4/ENAH/NCALD/SPIRE1/SHROOM4/GSN/MYOZ1/RDX/PFN2/CNN3/RAI14/MYL4/MICAL2/SNTA1/TMOD2/KLHL3/FHL3/SAMD14/SLC6A4/WIPF3/TMSB4Y/ESPNL/TLN1/EVL/LIMCH1

GOBP_NEUROPEPTIDE_SIGNALING_PATHWAY	GOBP_NEUROPEPTIDE_SIGNALING_PATHWAY	0.005729239	CARTPT/TAC1/NPPB/PENK/SCG5/ADCYAP1/NPFFR2/GRPR/CPE/NXPH3/GRP/GLRB/NPY/TAC3/NMUR1/SORCS2/TENM1/CYSLTR1/PCSK1N/SSTR2
GOBP_MICROTUBULE_ORGANIZING_CENTER_LOCALIZATION	GOBP_MICROTUBULE_ORGANIZING_CENTER_LOCALIZATION	0.005747362	CEP83/INTS13/GPSM2/NDE1/NUBP1/FHOD1/ASPM/AURKA/DLGAP5/MAD2L1/MISP/SYNE2/EZR
GOBP_REGULATION_OF_CELLULAR_RESPONSE_TO_VASCULAR_ENDOTHELIAL_GROWTH_FACTOR_STIMULUS	GOBP_REGULATION_OF_CELLULAR_RESPONSE_TO_VASCULAR_ENDOTHELIAL_GROWTH_FACTOR_STIMULUS	0.005737205	SMOC2/DCN/ATP2B4/ADAMTS3/MIR342/CCBE1/SEMA6A/ADGRA2/PTP4A3/ROBO1
GOBP_REGULATION_OF_DIGESTIVE_SYSTEM_PROCESS	GOBP_REGULATION_OF_DIGESTIVE_SYSTEM_PROCESS	0.005751059	APOA2/APOA1/NEUROD1/TACR1/FGF10/SCT/PTGER3/APOA4/OXTR

GOBP_NEGATIVE_REGULATION_OF_PROTEOLYSIS	GOBP_NEGATIVE_REGULATION_OF_PROTEOLYSIS	0.005746083	AHSG/CSN2/VTN/NLRP7/ITIH2/SERPIND1/F2/CRYAB/SERPINA10/SFRP2/GPC3/PI16/CST4/NGF/PI15/GAS1/TFPI2/MAP1A/SERPINA6/ITIH3/SERPINF2/AMBP/AGT/ITIH5/RECK/THBS1/SERPINF1/CRB2/HGF/SERPINA3/C4A/SERPINE2/C4B/TIMP4/SERPINA5/WFDC1/SERPINE1/SHH/SPOCK1/PROS1/CSTA/TIMP2/A2M/LRRK2/CST2/DPEP1/PCSK1N/R3HDML/PAPLN/WFDC3/CST5/COL6A3/PRNP/A2ML1/WFDC10B/TIMP3/SERPING1/TFPI/ANOS1/MAGEA3
GOMF_CYCLASE_ACTIVITY	GOMF_CYCLASE_ACTIVITY	0.005831204	ADCY5/ADCY2/ADCY1/GUCY1A1/GUCY1B1/NPR1/GUCY1A2/BST1/NPR2
GOMF_ASPARTIC_TYPE_PEPTIDASE_ACTIVITY	GOMF_ASPARTIC_TYPE_PEPTIDASE_ACTIVITY	0.005880034	CASP3/CTSE/BACE2/CASP7/PGC/PGA3/ASTL/PGA5/PGA4

GOBP_REGULATION_OF_COLLAGEN_METABOLIC_PROCESS	GOBP_REGULATION_OF_COLLAGEN_METABOLIC_PROCESS	0.005878996	MIR145/F2/DDR2/MFAP4/SERPINF2/TGFB3/LARP6/IL6/EMILIN1/CCN2/VIM/MIR149/FAP/INHBA/PDGFRB/NPPC/ENG/CYGB/F2R
GOBP_EMBRYONIC_APPENDAGE_MORPHOGENESIS	GOBP_EMBRYONIC_APPENDAGE_MORPHOGENESIS	0.005878679	HAND2/ALDH1A2/SFRP2/NOG/GPC3/CACNA1C/ZBTB16/GREM1/HOXD10/GLI3/RECK/RSPO2/AFF3/SALL4/MAP3K20/TGFB2/SHH/OSR1/PBX1/PRRX1/DKK1/CRABP2/PTCH1/TWIST1/TBX5/DYNC2H1/HOXD9/LEF1

GOBP_PROTON_TRANSMEMBRANE_TRANSPORT	GOBP_PROTON_TRANSMEMBRANE_TRANSPORT	0.005911915	UQCRFS1/ATP5F1EP2/SLC15A3/SPHK2/CLCN5/SLC9B2/TMCO3/COX5B/SLC11A2/ATP5PO/UQCR10/CHP2/TACO1/SLC25A3/COX4I1/SLC25A5/ATP6V0B/ATP5MC1/ATP5PB/COX6A1/SLC9A3R1/ATP1A1/SLC17A5/SLC9A4/COX15/TESC/COX5A/ATP5MC3/ATP5F1A/UQCRFS1P1/UQCRH/COX8A/ANTKMT/ATP5F1B/CLCN3/ATP5ME/PHB2/CLCN4/TCIRG1/SLC9A7/CHP1/PPIF/NDUFS7/ATP5F1D/UQCRC1/SLC22A18/CYC1/SLC9A2/MFSD3/SLC25A22/SLC9A3/ATP1B1/SLC9A1/ATP4B/ATP12A/SLC4A11/ATP4A
GOBP_PHOSPHATIDYLETHANOLAMINE_METABOLIC_PROCESS	GOBP_PHOSPHATIDYLETHANOLAMINE_METABOLIC_PROCESS	0.005937468	PCYT2/SELENOI/ETNK1/MFSD2A/ALOX15/PLAAT4/PLAAT2/PLA2G3/PLA2G2A/PLA2G4E
GOBP_LEUKOCYTE_TETHERING_OR_ROLLING	GOBP_LEUKOCYTE_TETHERING_OR_ROLLING	0.005968072	ELANE/JAM2/CXCL12/ADD2/CCL21/CX3CR1/SELP

GOBP_REGULATION_OF_REPRODUCTIVE_PROCESS	GOBP_REGULATION_OF_REPRODUCTIVE_PROCESS	0.006006717	VIP/TACR2/SFRP1/TACR1/MAEL/BNC1/RGN/IGF1/AR/SERPINA10/ZFPM2/CNR1/TEX11/CACNA1H/WT1/PDE3A/IHO1/SERPINF1/NODAL/ESR1/SHH/GDF9/RETN/OXTR/GJA1/SYDE1/PTGDS/DUSP1/PDE5A/PAEP/NPR2/INHBA/ITGB3/P2RY1/SULF1/KIT/TIMP1/OXT/EDNRB/NPPC
GOBP_RIBOSOMAL_LARGE_SUBUNIT_ASSEMBLY	GOBP_RIBOSOMAL_LARGE_SUBUNIT_ASSEMBLY	0.006079267	RPL5/NOP53/RPL11/RPL38/RPL24/RPL23A/FASTKD2/MRPL20/BRIX1/RPL6/RPF2/MDN1/DDX28/RPLP0/RPLP0P6/DHX30/TRAF7/NLE1/RRS1/MRTO4/BOP1/PPAN
GOMF_LIPOPROTEIN_PARTICLE_RECEPTOR_BINDING	GOMF_LIPOPROTEIN_PARTICLE_RECEPTOR_BINDING	0.006083285	APOA2/APOA1/RELN/APOC3/APOB/APBB1/DAB2/DKK1/CLU/SYT1/LRP1

GOMF_METALLOENDOPEPTIDASE_ACTIVITY	GOMF_METALLOENDOPEPTIDASE_ACTIVITY	0.00608059	ADAM33/PAPPA2/ADAMTS8/ADAMTS18/ADAMTS16/ADAMTSL3/ADAM23/MMP8/PRSS2/ADAMTS1/TLL1/MMP3/TRABD2B/CLCA1/ADAMTS3/MMP24/MMP2/ADAMTSL1/MMP7/MMP19/MMP16/ADAMTS10/MMP23B/PAPLN/PAPPA/ADAMTS9/ADAMTS5/TMPRSS6
GOBP_NEGATIVE_REGULATION_OF_RESPONSE_TO_BIOTIC_STIMULUS	GOBP_NEGATIVE_REGULATION_OF_RESPONSE_TO_BIOTIC_STIMULUS	0.006129822	NLRX1/MIR26B/AHR/NR1H2/CRK/METTL3/NLRC3/BANF1/PCBP2/CEACAM1/NPLOC4/TRIB1/FOXP3/UFD1/RIOK3/MUL1/TRAFD1/OAS1/TYRO3/MICA/ADAR/DUSP10/LYAR/ATG5/GPATCH3/ZDHHC18/YTHDF2/KLRC1/KLRD1/RNF26/DHX58/NR1H3/SLAMF8/RPS19/NFKBIL1/CNOT7/TARBP2/CACTIN/NMI/PARP1/HLA-E/TRIM21/GFER/CARD16/IL4I1/HLA-B/SIGIRR/HLA-A/PARP14/USP18/OAS3/MMP12/TKFC/DRD2/HLA-F/MICB/PPARG/PRDX2/LGALS9/C1QBP/KIR2DL4/TTLL12/NLRC5

GOBP_CATECHOL_CONTAINING_COMPOUND_METABOLIC_PROCESS	GOBP_CATECHOL_CONTAINING_COMPOUND_METABOLIC_PROCESS	0.006150648	HAND2/GRIN2A/INSM1/PAH/NPY/RNF180/EDNRA/NPR1/TGFB2/PDE1B/PRKN/MOXD1/HDC/KL/CHRNB2
GOMF_LIGAND_GATED_ION_CHANNEL_ACTIVITY	GOMF_LIGAND_GATED_ION_CHANNEL_ACTIVITY	0.006203016	GRIK3/CHRNA3/GRIK1/GRIN2A/ABCC9/RYR3/GRIK5/KCNH6/GLRB/P2RX2/KCNJ8/KCNH2/CHRNB4/P2RX1/SLC1A7/PKD2/CNGA1/ITPR1/RYR2/ANXA6/MCOLN3/GABRR1/CHRNB2
GOBP_NEGATIVE_REGULATION_OF_FAT_CELL_DIFFERENTIATION	GOBP_NEGATIVE_REGULATION_OF_FAT_CELL_DIFFERENTIATION	0.006232919	MIR483/ADIPOQ/FERMT2/ZFPM2/RUNX1T1/TGFB1I1/WWTR1/IL6/MIR27B/WNT10B/ID4/CCN4

GOBP_ANTIGEN_PROCESSING_AND_PRESENTATION_OF_EXOGENOUS_PEPTIDE_ANTIGEN	GOBP_ANTIGEN_PROCESSING_AND_PRESENTATION_OF_EXOGENOUS_PEPTIDE_ANTIGEN	0.00624708	HLA-DPA1/HLA-DMB/HLA-E/HLA-DOB/HLA-DRA/HLA-A/HLA-DQA2/MFSD6/HLA-DQA1/CTSE/HLA-DRB5/CD74/HLA-F/TAP2/HLA-DRB1/HLA-DOA/HLA-DQB1/DNM2/HLA-DQB2/HLA-DMA/TAP1
GOBP_DOPAMINE_RECEPTOR_SIGNALING_PATHWAY	GOBP_DOPAMINE_RECEPTOR_SIGNALING_PATHWAY	0.006280566	NSG2/ADCY5/GNAO1/RGS4/FLNA/CALY/GNAL/PALM/LRRK2/PTGER1/GNA14
GOMF_PROTEOGLYCAN_BINDING	GOMF_PROTEOGLYCAN_BINDING	0.006280566	AZU1/COL2A1/HPSE2/SLIT2/TNC/THBS1/COMP/FN1/CTSK/LPL/CFH/NID1/GPNMB/LRP1/COL5A1/FBLN7
GOBP_ENDOTHELIAL_CELL_CHEMOTAXIS	GOBP_ENDOTHELIAL_CELL_CHEMOTAXIS	0.006274881	SMOC2/FGF2/PRKD1/THBS1/FGFR1/CCN3/FGF1/MIR149/HSPB1/EGR3/NRP1/MET/SEMA5A

GOBP_POSITIVE_REGULATION_OF_COAGULATION	GOBP_POSITIVE_REGULATION_OF_COAGULATION	0.006274881	APOH/VTN/F2/F7/SERPINF2/CD36/THBS1/SERPINE1/EMILIN1/THBD
GOBP_SOMITE_DEVELOPMENT	GOBP_SOMITE_DEVELOPMENT	0.006287412	SFRP1/MEOX2/ALDH1A2/DLL3/SFRP2/NOG/FRZB/FOXF1/CRB2/TBX18/SHH/SMO/DKK1/MEOX1/PPP2R3A/FOXC2/TCF15
GOBP_REGULATION_OF_EPITHELIAL_CELL_MIGRATION	GOBP_REGULATION_OF_EPITHELIAL_CELL_MIGRATION	0.006291987	MIR483/APOH/TACR1/FGF10/MEOX2/SLIT2/FGF7/BMPER/PTN/SMOC2/FGF2/DCN/PRKD1/AGT/AKT3/ATP2B4/THBS1/SERPINF1/ATOH8/FGFR1/ANGPT1/TGFB2/RHOJ/NRP2/MCC/MIR342/CCBE1/MIR27B/FOXC2/FGF1/ADGRA2/MIR149/HAS2/SRPX2/RHOB/HSPB1/STC1/TEK/MEF2C/MIR222/SPARC/MIR10B/ADAMTS9/PTPRM/GPLD1/ITGB3/MMRN2/VEGFC/ADGRB1/MIR196A1/STARD13/PLPP3/NRP1/PFN2/RRAS

GOCC_DENDRITE_MEMBRANE	GOCC_DENDRITE_MEMBRANE	0.006300719	KCNB1/ITGA8/HPCA/LAMP5/SGCE/MPP2/THY1/GPER1/GABRE
GOBP_POSITIVE_REGULATION_OF_CARBOHYDRATE_METABOLIC_PROCESS	GOBP_POSITIVE_REGULATION_OF_CARBOHYDRATE_METABOLIC_PROCESS	0.006310338	GCG/ADCYAP1R1/SORBS1/HTR2A/RGN/IGF1/PRKAA2/PTH1R/IGF2/ZBTB20/HAS2/PPP1R3G/NNMT/GPLD1/P2RY1/GPER1
GOMF_PHOSPHOTRANSFERASE_ACTIVITY_FOR_OTHER_SUBSTITUTED_PHOSPHATE_GROUPS	GOMF_PHOSPHOTRANSFERASE_ACTIVITY_FOR_OTHER_SUBSTITUTED_PHOSPHATE_GROUPS	0.006329457	PIGN/PTDSS2/AASDHPPT/SGMS1/CHPT1/PIGF/DPAGT1/CEPT1/PIGG/PTDSS1/CRLS1/SELENOI/PIGO/CDS1
GOBP_POSITIVE_REGULATION_OF_STEM_CELL_DIFFERENTIATION	GOBP_POSITIVE_REGULATION_OF_STEM_CELL_DIFFERENTIATION	0.006333968	NKX2-5/RBM24/NAP1L2/PTN/FGF2/SOX5/TGFB2/TCF15/SOX17/LTBP3/TBX5
GOCC_GOLGI_LUMEN	GOCC_GOLGI_LUMEN	0.006341167	DEFA4/OGN/KERA/VTN/OMD/F2/PRELP/GPC3/NGF/DEFA6/DCN/MUC21/F7/LUM/CSPG4/SOD3/PROS1/CGA/MMP16/GPC6/F8/SDC2/MUC15/VCAN/FMOD/MUC2/F10/BGN

GOBP_BRANCHING_INVOLVED_IN_SALIVARY_GLAND_MORPHOGENESIS	GOBP_BRANCHING_INVOLVED_IN_SALIVARY_GLAND_MORPHOGENESIS	0.006370233	FGF10/FGF7/LAMA1/HGF/SHH/SNAI2/SEMA3C/NRP1/FGFR2/NTN4
GOBP_PYRIMIDINE_NUCLEOSIDE_MONOPHOSPHATE_METABOLIC_PROCESS	GOBP_PYRIMIDINE_NUCLEOSIDE_MONOPHOSPHATE_METABOLIC_PROCESS	0.006428672	NT5M/DHODH/DPYD/UMPS/UCK2/DCTD/NT5C/CAD/CDA/DCK/DUT/CMPK1/SHMT1/TYMP/TYMS
GOMF_ATP_DEPENDENT_ACTIVITY_ACTING_ON_RNA	GOMF_ATP_DEPENDENT_ACTIVITY_ACTING_ON_RNA	0.006433023	DDX51/DHX33/DDX20/MTREX/DHX38/DHX58/DDX41/DHX9/AQR/DDX56/EIF4A3/SNRNP200/FANCM/DDX28/DDX21/DDX23/DHX37/PIF1/RAD54B/DHX34/G3BP1/DDX60/UPF1/SUPV3L1/YTHDC2/DDX49/DDX11/DDX31/DHX30/DDX1/DHX15/DDX12P/MOV10/DDX54/HELZ2/DDX39A/BRIP1

GOMF_PROTEIN_SELF_ASSOCIATION	GOMF_PROTEIN_SELF_ASSOCIATION	0.006462169	CTSC/TEC/BCL10/PTK2B/SSNA1/AXIN1/HSF1/GPSM2/MYD88/ABCD3/SMTNL1/PLEC/FHOD1/SLC9A3R1/EML2/SGTA/CCL5/MDH2/SVIP/NKX3-1/S100A7A/KCTD9/DMTN/ADAM8/ZFYVE27/SLC2A1/PPARG/CCDC88C/TP53/SPTBN5/PCSK9
GOBP_GLIAL_CELL_MIGRATION	GOBP_GLIAL_CELL_MIGRATION	0.006478545	AZU1/RELN/ATP1B2/DAB1/NDN/GLI3/NTN1/CSPG4/CX3CR1/TGFB2/COL3A1/CCL2/GPR183/BMERB1

GOBP_NEGATIVE_REGULATION_OF_NEURON_PROJECTION_DEVELOPMENT	GOBP_NEGATIVE_REGULATION_OF_NEURON_PROJECTION_DEVELOPMENT	0.006537224	STMN2/EPHA7/RGMA/FAT3/SLIT2/DPYSL3/FLNA/DAB1/NLGN1/SEMA3E/NTN1/FGF13/LPAR1/NR2F1/SPOCK1/DAB2/DKK1/SPART/EFNB3/LRRK2/DENND5A/SEMA6A/VIM/SEMA6D/PMP22/THY1/SEMA6C/SEMA3G/MAP2/SEMA5B/SEMA3D/SEMA3C/NRP1/CRMP1
GOBP_MULTIVESICULAR_BODY_SORTING_PATHWAY	GOBP_MULTIVESICULAR_BODY_SORTING_PATHWAY	0.006550526	MITD1/SYTL4/RUBCN/STAM2/SNF8/STAM/RAB27A/CHMP2A/TMEM50B/SORT1/VPS25/TMEM50A/VPS36/VPS37A/VPS37D/VPS4A/UBXN6/UBAP1/MVB12A/VPS37C/CHMP6/VTA1/VPS28/CHMP2B/LEPROTL1/PDCD6IP/RILP/CHMP7/CHMP1A/VPS4B/HGS/CHMP5/PTPN23/VCP/VPS37B/CHMP4C/RAB27B/EXPH5

GOBP_POSITIVE_REGULATION_OF_DOUBLE_STRAND_BREAK_REPAIR_VIA_NONHOMOLOGOUS_END_JOINING	GOBP_POSITIVE_REGULATION_OF_DOUBLE_STRAND_BREAK_REPAIR_VIA_NONHOMOLOGOUS_END_JOINING	0.006599756	SHLD2/SHLD3/MAD2L2/PNKP/RIF1/PARP9/SMCHD1/DTX3L/PRKDC/KMT5C/WRAP53/PARP3
GOBP_CELL_COMMUNICATION_BY_ELECTRICAL_COUPLING_INVOLVED_IN_CARDIAC_CONDUCTION	GOBP_CELL_COMMUNICATION_BY_ELECTRICAL_COUPLING_INVOLVED_IN_CARDIAC_CONDUCTION	0.00659377	ATP1A2/ATP1B2/CACNA1C/HRC/GJC1/CAV1/SLC8A1/GJA1/RYR2/IRX3/TBX5
GOMF_NUCLEOBASE_CONTAINING_COMPOUND_TRANSMEMBRANE_TRANSPORTER_ACTIVITY	GOMF_NUCLEOBASE_CONTAINING_COMPOUND_TRANSMEMBRANE_TRANSPORTER_ACTIVITY	0.006604749	SLC35B3/SLC25A23/ABCD1/SLC25A17/SLC35A1/LRRC8A/SLC35D1/SLC25A5/SLC35B1/SLC35A2/SLC17A9/SLC25A33/SLC35D2/SLC25A24/SLC25A19/SLC35A4/SLC35C1/SLC29A2/SLC28A3/SLC35A3/SLC22A1/SLC19A1/SLC35D3/SLC28A2
GOBP_NEGATIVE_REGULATION_OF_ION_TRANSMEMBRANE_TRANSPORTER_ACTIVITY	GOBP_NEGATIVE_REGULATION_OF_ION_TRANSMEMBRANE_TRANSPORTER_ACTIVITY	0.00668353	CASQ2/ATP1A2/ACTN2/PLN/GRP/KCNE4/CAV1/GEM/PKD2/OSR1/REM1/SLN/RRAD/CLIC2

GOCC_EXTERNAL_SIDE_OF_PLASMA_MEMBRANE	GOCC_EXTERNAL_SIDE_OF_PLASMA_MEMBRANE	0.006688781	LCT/ASGR2/FGA/SLC2A4/GFRA1/F2/FGG/GHR/GFRA3/PRND/ATP1B2/ITGA7/TF/GFRA2/GPIHBP1/ENOX1/CXCL12/LEPR/CD36/ITGA8/COLEC11/ITGA9/CUBN/P2RX1/THBS1/GP2/MS4A2/IL1R1/CX3CR1/NCAM1/SELP/FCN1/SERPINE2/ITGA1/ABCB1/ITGA5/IGHV1-69D/SERPINA5/PDGFRA/CD302/FOLR2/CRLF1/MCAM/ANTXR1/CD1C/CTSK/IGHV1-69-2/CA4/SLC22A11/SCARA5/IGHG4/S1PR1/BCAM/ENPEP/THBD/PRNP/SCUBE1/ASGR1/RAET1G/ABCA1/HEG1/IGHV6-1/CXCR2/FCER2/FGB/IGHV7-4-1/TGFBR3/THY1/NRCAM/CXCR1/FCGR2B/F10/ITGB3/SELE/CLEC10A/IL13RA2/CHRNB2/CXCR4/LIFR/CD248/CCR7/CD34/ITGA11/CD1E/ACKR3/IL7R/IL6ST/KIT/CLEC14A/C[illegible]
GOBP_NEGATIVE_REGULATION_OF_EPITHELIAL_CELL_MIGRATION	GOBP_NEGATIVE_REGULATION_OF_EPITHELIAL_CELL_MIGRATION	0.006700961	MIR483/APOH/MEOX2/SLIT2/FGF2/DCN/ATP2B4/THBS1/SERPINF1/MCC/MIR149/STC1/MEF2C/ADAMTS9/PTPRM/MMRN2/ADGRB1/MIR196A1/STARD13/PFN2/ANGPT2/TBXA2R/HDAC5/EVL

GOBP_MONOVALENT_INORGANIC_CATION_HOMEOSTASIS	GOBP_MONOVALENT_INORGANIC_CATION_HOMEOSTASIS	0.006697645	SPX/NPPB/ATP1A2/AGTR1/SCN7A/TACR1/KCNMA1/KCNA5/ATP1B2/AGT/KCNH2/PDK4/EDNRA/NPR1/SLC8A1/CORIN/AVPR1A/CLIC4/RHCG/CCKBR/SLC9A9/UPK3A/LRRK2/RHBG/AVPR2/SLC26A3
GOBP_POSITIVE_REGULATION_OF_SYNAPSE_ASSEMBLY	GOBP_POSITIVE_REGULATION_OF_SYNAPSE_ASSEMBLY	0.006712223	FLRT2/ADGRB3/NLGN1/PTPRD/LRRN1/CLSTN2/SLITRK6/OXTR/NLGN3/SRPX2/SYNDIG1/NLGN2/THBS2/ADGRB1/AMIGO2/OXT/LRRC4B/AMIGO1/GHRL/CLSTN3/NTRK2

GOBP_INTRINSIC_APOPTOTIC_SIGNALING_PATHWAY_IN_RESPONSE_TO_DNA_DAMAGE_BY_P53_CLASS_MEDIATOR	GOBP_INTRINSIC_APOPTOTIC_SIGNALING_PATHWAY_IN_RESPONSE_TO_DNA_DAMAGE_BY_P53_CLASS_MEDIATOR	0.006715751	ZNF385A/SNW1/SHISA5/CDKN1A/EP300/TAF9/SIRT1/TOPORS/RPS27L/RPL26/BAG6/BCL3/USP28/ATAD5/CHEK2/ING2/HIPK1/HNRNPK/PML/PYCARD/KDM1A/BRCA2/TRIAP1/MSH2/CD74/DDIT4/TP53/AEN/TP73/MUC1/ELL3
GOBP_REGULATION_OF_MITOTIC_CELL_CYCLE_SPINDLE_ASSEMBLY_CHECKPOINT	GOBP_REGULATION_OF_MITOTIC_CELL_CYCLE_SPINDLE_ASSEMBLY_CHECKPOINT	0.006723206	XRCC3/AURKB/NDC80/GEN1/INCENP/CCNB1/MAD2L1/BIRC5/CDCA8/CDT1
GOBP_ACTIVATION_OF_PHOSPHOLIPASE_C_ACTIVITY	GOBP_ACTIVATION_OF_PHOSPHOLIPASE_C_ACTIVITY	0.006789137	ADCYAP1R1/HTR2A/HTR2B/AGT/EDNRA/LPAR1/NMUR1/AVPR1A/SELE/RASGRP4
GOBP_POSITIVE_REGULATION_OF_STRIATED_MUSCLE_CELL_DIFFERENTIATION	GOBP_POSITIVE_REGULATION_OF_STRIATED_MUSCLE_CELL_DIFFERENTIATION	0.006779683	SMYD1/MYOCD/AKAP6/IGF1/RBM24/LMOD3/HOPX/SHH/MEF2C/MAMSTR

GOBP_POSITIVE_REGULATION_OF_PEPTIDYL_TYROSINE_PHOSPHORYLATION	GOBP_POSITIVE_REGULATION_OF_PEPTIDYL_TYROSINE_PHOSPHORYLATION	0.006784742	RELN/ADIPOQ/VTN/THBS4/FGF10/GFRA1/HTR2A/IGF1/FGF7/GHR/EPGN/CNTN1/IL24/GREM1/ALKAL1/IGF2/AGT/CD36/TSLP/CSF3/CSPG4/HGF/ANGPT1/ITGA5/IL6/CTF1/CRLF1/ISL1/NTF3/DOCK3/DLG4/NEURL1/IL11/PRNP/NOX4/ITGB3/FLT3/IL6ST/IL34/KIT/ACVR1/PLPP3/PECAM1/NRP1/TNFSF18/HPX/CSF2

GOMF_MODIFICATION_DEPENDENT_PROTEIN_BINDING	GOMF_MODIFICATION_DEPENDENT_PROTEIN_BINDING	0.006801501	SQSTM1/BRD9/SPRTN/FAAP20/DPF2/OPTN/BABAM2/ZMYND8/PHF13/CHD8/TAF1/KDM7A/ZBTB1/ABRAXAS2/GLYR1/PWWP2A/WDR81/PSMD4/PWP1/USP15/BPTF/ING1/ATAD2B/YEATS2/IKBKG/PHF8/VAV1/ING3/KDM8/BRD4/ZRANB3/UBXN1/THAP7/MTF2/ING5/FMR1/HDAC6/TRIM24/SHARPIN/CHD1/NPLOC4/PHF19/DNAJB2/YEATS4/PHIP/TDRD3/DAXX/UFD1/RNF168/UIMC1/RNF169/SUZ12/DNAJC2/TP53BP1/CBX3/BAG6/ZZZ3/BRD7/RBBP5/RAD23B/PSME4/UBQLN1/HDGFL2/BAZ2A/PTPN6/FAN1/ZFAND2B/ING2/AGAP3/UBL7/MSH6/PRPF8/BRCC3/IDE/ZZEF1/PLCG2/BRD2/PML/LRWD1/L3MBTL2/UBQLN4/ATRIP/RAD18/WDR5/AGL/TNIP2/IKBKE/RAD23A/CBX4/SPIN4/VCP/CARM1/KDM4A/CBX2/ANKRD13B/PARP10/CXXC1/SMA[illegible]

GOBP_SPECIFICATION_OF_SYMMETRY	GOBP_SPECIFICATION_OF_SYMMETRY	0.00682937	HAND1/CER1/HAND2/NKX2-5/FGF10/ALDH1A2/ASB2/GREM2/NKX3-2/GREM1/DAAM2/BICC1/FOXF1/NODAL/PKD2/SHH/SMO/CC2D2A/SOX17/MEF2C/DYNC2H1/VANGL2/ACVR1/NDRG4/APLNR/MICAL2/PITX2/DNAH5/ENG/DLL1/NPHP3/SOX18
GOCC_U2_TYPE_CATALYTIC_STEP_2_SPLICEOSOME	GOCC_U2_TYPE_CATALYTIC_STEP_2_SPLICEOSOME	0.006855395	SNRPE/SYF2/BUD31/CWC22/SNRPD2/SNW1/SNRPD3/DHX8/CDC5L/RBM22/BCAS2/SNRPG/CRNKL1/PPIL1/SNRPF/SNRPA1/CDC40/SNRPB/XAB2/EFTUD2/PRPF19/PRPF8/SRRM2/SNRNP40/AQR/SNRPD1/PLRG1
GOCC_PLATELET_DENSE_GRANULE	GOCC_PLATELET_DENSE_GRANULE	0.006862567	APOH/ITIH3/SELP/SELENOP/CLEC3B/RARRES2/ITPR1/TIMP3

GOBP_OLIGODENDROCYTE_DEVELOPMENT	GOBP_OLIGODENDROCYTE_DEVELOPMENT	0.006897385	NKX2-2/SOX10/PLP1/ASCL1/CNTN1/WASF3/CNTNAP1/LPAR1/MAL/ASPA/SHH/ID4/TENM4/CLU/PRDM8
GOBP_DIENCEPHALON_DEVELOPMENT	GOBP_DIENCEPHALON_DEVELOPMENT	0.006950365	GHRH/FGF10/PCSK1/ALDH1A2/PTCHD1/NOG/FGF2/SEMA3E/GLI1/NRP2/SHH/SMO/ISL1
GOMF_ENDOPEPTIDASE_REGULATOR_ACTIVITY	GOMF_ENDOPEPTIDASE_REGULATOR_ACTIVITY	0.007008075	AHSG/CSN2/NLRP7/ITIH2/SERPIND1/SERPINA10/SFRP2/CST4/NGF/TFPI2/SERPINA6/ITIH3/SERPINF2/AMBP/AGT/ITIH5/RECK/SERPINF1/CRB2/SERPINA3/C4A/SERPINE2/C4B/TIMP4/SERPINA5/WFDC1/SERPINE1/SPOCK1/PROS1/CSTA/TIMP2/A2M/CST2/DPEP1/PCSK1N/PAPLN/WFDC3/CST5/COL6A3/PRNP/A2ML1/WFDC10B/TIMP3/SERPING1/TFPI/ANOS1

GOBP_TRANSCRIPTION_PREINITIATION_COMPLEX_ASSEMBLY	GOBP_TRANSCRIPTION_PREINITIATION_COMPLEX_ASSEMBLY	0.00704165	TAF6/MED4/MED31/CAND1/BDP1/MED28/TAF1/MED22/PSMC6/TBP/MED10/TAF13/TAF4/GTF2B/GTF2A1/HMGB1/TAF9/MED30/UBTF/TAF12/TAF2/MED8/DR1/MED11/MED27/POLR1E/MED14/TAF1C/BAZ2A/TAF6L/MED25/SMARCB1/MED23/PIH1D1/MED16/RRN3/MED15/MED9/THRA/BRF2/BRF1/MED26/TAF5/MED1/MED18/GTF2A2/SMARCA4/TP53/MED24
GOBP_CARDIAC_SEPTUM_MORPHOGENESIS	GOBP_CARDIAC_SEPTUM_MORPHOGENESIS	0.007083021	HAND1/NKX2-5/SLIT2/NOG/SLIT3/ZFPM2/CCN1/TGFB2/NRP2/HEYL/SMO/ISL1/PARVA/TGFBR3/TBX5/ROBO1/ACVR1/SEMA3C/NRP1/APLNR/BMP5/FGFR2/FZD1/SOX11

GOBP_MESENCHYMAL_CELL_PROLIFERATION	GOBP_MESENCHYMAL_CELL_PROLIFERATION	0.0071259	HAND2/FGF7/SIX2/GPC3/ZEB1/CHRD/FOXF1/SHH/SMO/OSR1/PRRX1/DCHS1
GOBP_CYTOPLASMIC_PATTERN_RECOGNITION_RECEPTOR_SIGNALING_PATHWAY	GOBP_CYTOPLASMIC_PATTERN_RECOGNITION_RECEPTOR_SIGNALING_PATHWAY	0.007116885	SLC15A2/USP15/XIAP/BIRC2/NLRX1/CLPB/PUM2/PUM1/NPLOC4/UFD1/RIOK3/IFIH1/OTULIN/RELA/IRF7/MAP2K6/GPATCH3/ALPK1/SLC15A3/DHX58/IRF3/ANKRD17/DDX60/PTPN22/RIPK2/TIFA/OAS3/TKFC/PHB2/OASL/TRIM25/BIRC3/C1QBP/INAVA/TRIM15

GOCC_NUCLEAR_PERIPHERY	GOCC_NUCLEAR_PERIPHERY	0.007123848	SMARCC1/PIAS4/HAT1/ACTL6A/SMARCC2/NONO/PHF5A/STAG1/HLCS/POLA1/DCAF7/SMARCB1/PSMA6/CFL1/SFPQ/RUVBL1/CHMP1A/PRPF40A/LMNA/SRPK1/AKAP8/HNRNPU/CENPW/PML/RUVBL2/PRKCD/CASK/MEN1/NUP93/DAG1/HNRNPA2B1/ARFGEF1/PAXIP1/EBNA1BP2/SRRM1/RAD21/NUP205/GMCL1/PSPC1/SMC1A/HNRNPM/SUV39H1/CAD/ENC1/SMARCD2/MAEA/TELO2/SMARCE1/CXXC1/PHB2/SMARCA4/ANXA2/CENPF/PCNA/KIF4A/DGKQ/BLM/TP53/NSMF/GFI1B/PRKCZ/LMNB2/LMNTD2/LMNB1/MYB/KRT8/UHRF1

GOBP_BIOMINERALIZATION	GOBP_BIOMINERALIZATION	0.007136081	AHSG/CER1/OMD/MGP/IGF1/GPC3/ROR2/DDR2/PTN/GREM1/PTH1R/ASPN/GPM6B/RSPO2/TGFB3/CCN1/SLC8A1/TMEM119/COMP/OSR1/CLEC3B/ADRB2/NELL1/WNT10B/SLC24A3/PTHLH/S1PR1/LOX/BMPR1B/KL/MEF2C/LTBP3/FAM20A/COL1A2/TWIST1/WLS/COL1A1/ACVR1/SRGN/FBLN7/FGFR2/AMTN/FAM20C/BMP6/KLF10
GOBP_POSITIVE_REGULATION_OF_VASCULAR_PERMEABILITY	GOBP_POSITIVE_REGULATION_OF_VASCULAR_PERMEABILITY	0.007149533	TACR2/TACR1/PDE3A/PDE2A/ANGPT1/PTP4A3/CXCR2
GOBP_TRICARBOXYLIC_ACID_CYCLE	GOBP_TRICARBOXYLIC_ACID_CYCLE	0.007292039	SUCLG1/SDHAF2/OGDH/IDH3G/DHTKD1/SUCLA2/SUCLG2/ACO1/IREB2/MRPS36/MDH1/SDHD/DLST/IDH3B/PDHA1/SDHB/CS/MDH2/ACO2/SDHA/IDH3A/FH/IDH2/IDH1/DLAT

GOBP_NEGATIVE_REGULATION_OF_MUSCLE_CELL_DIFFERENTIATION	GOBP_NEGATIVE_REGULATION_OF_MUSCLE_CELL_DIFFERENTIATION	0.007303659	MYOCD/NKX2-5/RBPMS2/PI16/RGS4/IGF2/PRDM6/PLPP7/RGS2/TMEM119/SHH/DKK1/FZD7/CCN3
GOBP_CYTOSKELETON_DEPENDENT_CYTOKINESIS	GOBP_CYTOSKELETON_DEPENDENT_CYTOKINESIS	0.007302457	JTB/SPAST/IST1/ACTR2/ACTR3/CUL7/USP8/EXOC2/RASA1/SEPTIN8/RHOA/VPS4A/POLDIP2/SEPTIN1/RAB35/EXOC1/STAMBP/ARF1/SEPTIN9/TMEM250/RHOC/CHMP6/ALKBH4/CHMP2B/NUP62/PDCD6IP/RTKN/CFL1/CHMP7/CHMP1A/CKAP2/VPS4B/CHMP5/ANK3/SPTBN1/PLEC/SPIRE2/KIF20A/STMN1/AURKB/KIF20B/KIF23/CENPA/EXOC6/BIN3/CNTROB/TTC19/CIT/ANLN/INCENP/KIF4A/RACGAP1/CHMP4C/ESPL1/ECT2/PLK1/BIRC5/CEP55/CDCA8/TRIM36

GOBP_RIBONUCLEOSIDE_MONOPHOSPHATE_METABOLIC_PROCESS	GOBP_RIBONUCLEOSIDE_MONOPHOSPHATE_METABOLIC_PROCESS	0.007359756	UCKL1/HPRT1/NT5C2/GDA/RFK/DHODH/ADSS2/DPYD/AMPD2/UMPS/MAGI3/GMPS/TJP2/GUK1/APRT/IMPDH2/ADK/UCK2/CASK/NT5C/GART/NUDT2/PNP/ATIC/ADSS1/AK1/IMPDH1/AK2/PFAS/XDH/CAD/PRPS2/CDA/DCK/CMPK1/PPAT/PAICS/ADA
GOBP_NADPH_REGENERATION	GOBP_NADPH_REGENERATION	0.007407475	RBKS/TIGAR/RPTOR/RPE/PGLS/SHPK/TALDO1/MTOR/MLST8/ALDH1L1/TKT/PRPS2/PGAM1/PGD/TP53
GOBP_POSITIVE_REGULATION_OF_CELL_GROWTH	GOBP_POSITIVE_REGULATION_OF_CELL_GROWTH	0.00745157	SYT4/SFRP1/AKAP6/F2/IGF1/SFRP2/MAP1B/NGF/IGFBP1/CXCL12/NTN1/PRSS2/MAPT/RND2/LPAR3/S100A8/TGFB2/L1CAM/S100A9/PRKN/AVPR1A/FN1

GOBP_CYTOPLASMIC_TRANSLATIONAL_INITIATION	GOBP_CYTOPLASMIC_TRANSLATIONAL_INITIATION	0.007491622	DHX29/EIF2B3/MCTS1/EIF4A1/METTL3/EIF3M/EIF3K/EIF3J/MIF4GD/EIF2S3/DENR/EIF2D/RPL13A/EIF3B/EIF3D/EIF3H/YTHDF2/EIF3E/EIF3I/NCBP1/EIF4G1/EIF3A/EIF3G/EIF4B/MTOR/EIF4H/EIF3CL/EIF2S3B/SLBP/EIF3C
GOBP_REGULATION_OF_TELOMERE_CAPPING	GOBP_REGULATION_OF_TELOMERE_CAPPING	0.007517111	RAD50/MAPKAPK5/SMG6/PNKP/MAP3K4/NABP2/TERF2/RTEL1/MAP2K7/XRCC1/USP7/MAPK3/NEK2/HNRNPD/AURKB/NBN/PRKCQ/TNKS/MAPK15
GOBP_RNA_5_END_PROCESSING	GOBP_RNA_5_END_PROCESSING	0.007514108	RPP14/TRMT10C/RPP38/PIWIL2/PRORP/PNPT1/POP5/HSD17B10/POP4/ABT1/UTP20/POP7/NOP9/RPP25/THG1L/TBL3/RPP25L/POP1/NOP14

GOBP_BLOOD_VESSEL_ENDOTHELIAL_CELL_MIGRATION	GOBP_BLOOD_VESSEL_ENDOTHELIAL_CELL_MIGRATION	0.007524072	APOA1/MIR483/MEOX2/SLIT2/CYP1B1/FGF2/GREM1/PRKD1/AKT3/ATP2B4/THBS1/FGFR1/ANGPT1/RHOJ/MIR342/MIR27B/FOXC2/MIR149/SRPX2/HSPB1/MEF2C/EGR3/MIR10B/GPLD1/MMRN2/VEGFC/ROBO1/MIR196A1/STARD13/CLEC14A/NRP1
GOCC_ORGANELLE_MEMBRANE_CONTACT_SITE	GOCC_ORGANELLE_MEMBRANE_CONTACT_SITE	0.007533243	VPS13A/STARD3/ATG5/SARAF/CHCHD6/SERAC1/APOO/MICOS13/GRAMD2A/MBOAT7/TMEM41B/CANX/TMX2/IMMT/GRAMD1C/ATG16L1/BCAP31/ATG2A/RMDN3/CHCHD3/PDZD8/C2CD2L/TOMM40/GRAMD1B
GOBP_REGULATION_OF_CAMP_DEPENDENT_PROTEIN_KINASE_ACTIVITY	GOBP_REGULATION_OF_CAMP_DEPENDENT_PROTEIN_KINASE_ACTIVITY	0.00756384	ADIPOQ/NPFFR2/ATP2B4/PRKAR2B/PKIA/PKIG/ADRB2

GOBP_NADH_DEHYDROGENASE_COMPLEX_ASSEMBLY	GOBP_NADH_DEHYDROGENASE_COMPLEX_ASSEMBLY	0.007564027	NDUFB8/NDUFC2/OXA1L/NDUFB5/NDUFC1/NDUFA1/NDUFB4/DMAC1/NDUFS8/NDUFB2/NDUFA8/ACAD9/NDUFA6/NDUFB10/NDUFS5/NDUFB3/NDUFA12/NDUFA5/NDUFAF5/NDUFAF2/NDUFAF6/TMEM186/NDUFA9/NDUFB6/NDUFA11/NDUFAF1/NDUFA10/NDUFS2/NDUFA2/NDUFS1/NDUFS3/NDUFB7/BCS1L/NDUFAB1/NDUFA13/TIMM21/ECSIT/FOXRED1/NDUFS7
GOCC_EXCITATORY_SYNAPSE	GOCC_EXCITATORY_SYNAPSE	0.00758318	IGSF11/NLGN4X/NLGN1/SLC17A7/NLGN4Y/PALLD/SYT11/BSN/NLGN3/LRRC4/DLG4/SRPX2/SYNDIG1/NLGN2/SYT1/SYP
GOBP_CELLULAR_RESPONSE_TO_CAMP	GOBP_CELLULAR_RESPONSE_TO_CAMP	0.007587273	AQP8/PENK/ADIPOQ/AKAP6/CYP1B1/WT1/PDE2A/SLC8A1/PKD2/IGFBP5/WNT10B/SLC26A3/STC1

GOBP_CELLULAR_RESPONSE_TO_CALCIUM_ION	GOBP_CELLULAR_RESPONSE_TO_CALCIUM_ION	0.007613744	SYT4/KCNB1/RYR3/PRKAA2/NLGN1/ADCY1/RASGRP2/HPCA/FOSB/PKD2/CLIC4/SYT11/ACER1/DPEP1/CPNE8/MEF2C/ITPKB/GPLD1/SYT1/RASA4B/SYT5
GOBP_FIBROBLAST_MIGRATION	GOBP_FIBROBLAST_MIGRATION	0.007677062	MIR145/ACTA2/HAS1/TNS1/DDR2/PAK3/FGF2/AKAP12/THBS1/SLC8A1/CCN3/ILK/ITGB3/CD248
GOBP_REGULATION_OF_STEROL_TRANSPORT	GOBP_REGULATION_OF_STEROL_TRANSPORT	0.007759235	APOA2/APOA1/APOC3/ADIPOQ/ABCA8/CES1/CAV1/APOA4/SHH/MIR27B/ABCA1/PTCH1/PLTP/ENPP7/LRP1/MIR613
GOMF_OXIDOREDUCTASE_ACTIVITY_ACTING_ON_THE_CH_NH_GROUP_OF_DONORS_NAD_OR_NADP_AS_ACCEPTOR	GOMF_OXIDOREDUCTASE_ACTIVITY_ACTING_ON_THE_CH_NH_GROUP_OF_DONORS_NAD_OR_NADP_AS_ACCEPTOR	0.007807842	QDPR/MTHFD2L/ALDH1L1/PYCR3/MTHFR/BLVRB/DHFRP1/MTHFD2/MTHFD1L/DHFR/PYCR1/MTHFD1

GOBP_NEPHRON_MORPHOGENESIS	GOBP_NEPHRON_MORPHOGENESIS	0.007810661	TMEM59L/SIX2/NOG/ADAMTS16/GPC3/FGF2/GREM1/WT1/GLI3/GREB1L/AGT/PKD2/SHH/SMO/OSR1/PBX1/WNT2B/TCF21/DCHS1/FGF1/ILK/PTCH1/IRX3
GOBP_NEGATIVE_REGULATION_OF_INTRACELLULAR_TRANSPORT	GOBP_NEGATIVE_REGULATION_OF_INTRACELLULAR_TRANSPORT	0.007800186	ADIPOQ/PLN/CRYAB/APOD/MAP1B/CD36/RAB23/ANGPT1/PKIA/PKIG/MIR27B/LRRK2
GOBP_REGULATION_OF_RESPIRATORY_GASEOUS_EXCHANGE	GOBP_REGULATION_OF_RESPIRATORY_GASEOUS_EXCHANGE	0.007859405	ATP1A2/GRPR/GRP/PBX3/NLGN3/TSHZ3/NLGN2
GOBP_ALDITOL_METABOLIC_PROCESS	GOBP_ALDITOL_METABOLIC_PROCESS	0.007905643	GPD2/SHPK/MOGAT2/PCK2/TPI1/COQ3/TKFC/COQ2/SORD/PCK1/GOT1/PLA2G4A
GOBP_PROTEIN_LOCALIZATION_TO_SYNAPSE	GOBP_PROTEIN_LOCALIZATION_TO_SYNAPSE	0.007908272	RELN/NRXN2/GRIN2A/ZDHHC15/MAP1A/NLGN1/NPTX1/MAPT/DLG2/SNAP25/GPC6/BSN/KIF5A/DLG4/HSPB1/NLGN2

GOBP_ENDOSOME_ORGANIZATION	GOBP_ENDOSOME_ORGANIZATION	0.007922371	TMEM9/PHETA2/ARFGEF2/LAMTOR1/SQSTM1/EXOC8/STAM2/PI4K2A/SNF8/STAM/VPS11/ALS2/RAB27A/CHMP2A/RAB22A/SNX10/TMEM127/AQP11/ATP6AP1/WASH3P/ATP6V1D/VPS25/ATP6V1H/VPS33B/ATP6V1F/VPS18/IST1/USP8/VPS36/VPS37A/VPS37D/STX6/VPS4A/ATP6V1A/UBAP1/MVB12A/VPS37C/CHMP6/VTA1/PLEKHF2/VPS28/CHMP2B/RNF26/PDCD6IP/RILP/RAB11A/CHMP7/CHMP1A/VPS4B/HGS/ATP6V0B/CHMP5/HOOK2/ALS2CL/HOOK1/ANXA8/PLEKHJ1/CLCN3/VPS37B/PHETA1/CHMP4C/PI4K2B/ANXA8L1/FASLG

GOMF_RIBONUCLEASE_ACTIVITY	GOMF_RIBONUCLEASE_ACTIVITY	0.007927656	PLD6/DIS3L2/RNASET2/SLFN13/CNOT8/EXOSC8/USB1/PARN/CNOT6L/CPSF3/DIS3L/DIS3/RIDA/PAN2/NUDT16L1/KHNYN/PPP1R8/ISG20L2/XRN1/TSEN34/AZGP1/TSEN2/POP4/PIWIL4/RCL1/CNOT7/EXOSC10/INTS11/SND1/EXOSC2/EXOSC7/EXOSC5/POP7/PDE12/RPP25/CNOT6/AGO2/EXOSC9/YBEY/EXOSC4/NUDT12/CNOT1/TOE1/ELAC2/DCPS/MRPL44/ERI2/NOB1/ISG20/HELZ2/POLRMT/ZC3H12A/ERI1/POP1/EXO1/FEN1/ENDOG/PIWIL1/RNASE7/ERN2
GOBP_GLUTAMATE_SECRETION	GOBP_GLUTAMATE_SECRETION	0.007960815	SYT4/AVPR1A/GJA1/APBA1/GABBR1/NTRK2/SNCA/NTSR1/KMO/P2RX7/STXBP1/ADORA1
GOBP_CRISTAE_FORMATION	GOBP_CRISTAE_FORMATION	0.007991839	SAMM50/CHCHD6/APOO/MICOS13/AFG3L2/IMMT/UQCC3/CHCHD3/DNAJC11/CHCHD10/LETM1

GOBP_NEGATIVE_REGULATION_OF_SUPRAMOLECULAR_FIBER_ORGANIZATION	GOBP_NEGATIVE_REGULATION_OF_SUPRAMOLECULAR_FIBER_ORGANIZATION	0.008003442	STMN2/MYOC/LMOD1/CRYAB/SLIT2/TMOD1/TUBB4A/MAP1B/CLIP3/SVIL/NAV3/PHLDB2/ADD2/KANK4/CGNL1/FGF13/LMOD3/HDGFL3/PRKN/EMILIN1/FHOD3/DLC1/BMERB1/ARHGAP28/MIR149/S1PR1/PLEKHH2/TMSB15A/CLU/CORO2B/MAP2/MYADM/GSN/RDX/PFN2
GOBP_POSITIVE_REGULATION_OF_CARDIOCYTE_DIFFERENTIATION	GOBP_POSITIVE_REGULATION_OF_CARDIOCYTE_DIFFERENTIATION	0.008036953	MYOCD/NKX2-5/MIR145/TGFB2/MEF2C/TBX5/GPER1
GOBP_CELL_MIGRATION_INVOLVED_IN_HEART_DEVELOPMENT	GOBP_CELL_MIGRATION_INVOLVED_IN_HEART_DEVELOPMENT	0.008075618	HAND2/BVES/EDNRA/SNAI2/DCHS1/TWIST1/TBX5/PDGFRB/SEMA3C/NDRG4/PITX2/ENG
GOBP_TRABECULA_FORMATION	GOBP_TRABECULA_FORMATION	0.008090881	SFRP1/NKX2-5/GREM1/ADAMTS1/RBP4/MMP2/WNT10B/TGFBR3/TEK/COL1A1

GOBP_REGULATION_OF_CELL_SUBSTRATE_JUNCTION_ORGANIZATION	GOBP_REGULATION_OF_CELL_SUBSTRATE_JUNCTION_ORGANIZATION	0.008152107	SFRP1/MYOC/APOD/FERMT2/EPHA3/PHLDB2/GREM1/GPM6B/THBS1/FAM107A/DLC1/COL16A1/TEK/THY1
GOBP_TRANSCRIPTION_ELONGATION_BY_RNA_POLYMERASE_II_PROMOTER	GOBP_TRANSCRIPTION_ELONGATION_BY_RNA_POLYMERASE_II_PROMOTER	0.008155284	ELL/ERCC3/BRD4/ELOB/CDK13/ERCC6/NELFE/MED30/CTNNB1/RECQL5/MED8/RNF168/MED11/ELOA/MED27/CDK12/SETD2/SUPT16H/CCNT1/TCEA1/CTR9/CCNK/TCERG1/VHL/LEO1/SUPT5H/INTS12/MED14/MED25/WDR43/MED23/NCBP1/MED16/INTS13/EAF1/INTS7/AXIN1/INTS14/INTS9/HNRNPU/INTS8/MED15/INTS11/MED9/INTS1/ELOF1/INTS4/NELFCD/CTDP1/NELFB/GTF2F1/INTS5/ADRM1/MED26/MED1/SIRT6/MED18/NELFA/INTS2/EZH2/PWWP2B/INTS10/MED24/ELL3

GOMF_ISOPRENOID_BINDING	GOMF_ISOPRENOID_BINDING	0.008275867	ALDH1A2/UGT2B17/ADH7/RBP7/UGT1A7/RBP4/RLBP1/SERPINA5/CRABP2/PTGDS/LRAT/RBP1/ADH4/RBP5/CYP2W1/LCN12/RBP2/UGT2B15
GOBP_POSITIVE_REGULATION_OF_BLOOD_CIRCULATION	GOBP_POSITIVE_REGULATION_OF_BLOOD_CIRCULATION	0.008317136	ATP1A2/NKX2-5/RGS4/ADRA1D/HRC/TPM1/CHGA/ADRA1B/RGS2/TGFB2/AVPR1A/CCN2/RYR2
GOBP_STEM_CELL_DEVELOPMENT	GOBP_STEM_CELL_DEVELOPMENT	0.008349123	SOX10/HAND2/ALDH1A2/HTR2B/CDH2/FGF2/SEMA3E/RADIL/EDNRA/NRP2/SHH/SMO/FN1/ISL1/SNAI2/FOXC2/SEMA6A/SEMA6D/ANXA6/TWIST1/SEMA6C/SEMA3G/SEMA5B/SEMA3D/ACVR1/SEMA3C/EDNRB/NRP1
GOBP_CORONARY_VASCULATURE_DEVELOPMENT	GOBP_CORONARY_VASCULATURE_DEVELOPMENT	0.008375171	HAND2/MYOCD/MIR145/GPC3/SGCD/FGF2/PRICKLE1/TGFBR3/TBX5/DYNC2H1/GPER1/PDGFRB/NRP1/APLNR

GOBP_SENSORY_PERCEPTION_OF_SMELL	GOBP_SENSORY_PERCEPTION_OF_SMELL	0.008458712	GJB4/OMP/OR2I1P/SLC6A3/OR8K5
GOMF_3_5_DNA_HELICASE_ACTIVITY	GOMF_3_5_DNA_HELICASE_ACTIVITY	0.008487244	DHX9/FANCM/WRN/ASCC3/MCM6/BLM/MCM5/RECQL4/MCM2
GOBP_NEGATIVE_REGULATION_OF_SMOOTHENED_SIGNALING_PATHWAY	GOBP_NEGATIVE_REGULATION_OF_SMOOTHENED_SIGNALING_PATHWAY	0.008494906	PTCH2/GPC3/GLI3/GLI1/HHIP/SERPINE2/GPR161/PTCH1/GLIS2/PRKACB
GOBP_REGULATION_OF_VIRAL_INDUCED_CYTOPLASMIC_PATTERN_RECOGNITION_RECEPTOR_SIGNALING_PATHWAY	GOBP_REGULATION_OF_VIRAL_INDUCED_CYTOPLASMIC_PATTERN_RECOGNITION_RECEPTOR_SIGNALING_PATHWAY	0.008506104	NOP53/USP15/BIRC2/NLRX1/PUM2/PUM1/NPLOC4/UFD1/RIOK3/GPATCH3/DHX58/ANKRD17/DDX60/TKFC/OASL/BIRC3/C1QBP/TRIM15
GOBP_DNA_DAMAGE_RESPONSE_SIGNAL_TRANSDUCTION_BY_P53_CLASS_MEDIATOR_RESULTING_IN_CELL_CYCLE_ARREST	GOBP_DNA_DAMAGE_RESPONSE_SIGNAL_TRANSDUCTION_BY_P53_CLASS_MEDIATOR_RESULTING_IN_CELL_CYCLE_ARREST	0.008511622	RPL26/CRADD/CHEK2/PML/TRIAP1/PIDD1/CASP2/GTSE1/PLK2/TP53/MDM2/MUC1
GOBP_POSITIVE_REGULATION_OF_SMOOTH_MUSCLE_CONTRACTION	GOBP_POSITIVE_REGULATION_OF_SMOOTH_MUSCLE_CONTRACTION	0.008574035	SPX/TACR2/TACR1/MYOCD/OXTR/PROK2/GPER1/KIT/OXT/EDN2/CHRM3/TBXA2R/GHRL/F2R

GOBP_CARDIOBLAST_DIFFERENTIATION	GOBP_CARDIOBLAST_DIFFERENTIATION	0.008569277	MYOCD/NKX2-5/GREM1/PRICKLE1/TGFB2/ISL1/TBX5
GOBP_POSITIVE_REGULATION_OF_CELLULAR_CARBOHYDRATE_METABOLIC_PROCESS	GOBP_POSITIVE_REGULATION_OF_CELLULAR_CARBOHYDRATE_METABOLIC_PROCESS	0.008574955	GCG/ADCYAP1R1/SORBS1/RGN/IGF1/PTH1R/IGF2/PPP1R3G/NNMT/GPLD1/P2RY1/GPER1
GOBP_AORTA_DEVELOPMENT	GOBP_AORTA_DEVELOPMENT	0.008574955	MYLK/NGFR/MIR145/FKBP10/PRICKLE1/EDNRA/PDE2A/TGFB2/PKD2/COL3A1/EFEMP2/LOX/ADAMTS9/ROBO1/PDGFRB/LRP1/APLNR
GOBP_EPITHELIAL_CELL_DIFFERENTIATION_INVOLVED_IN_KIDNEY_DEVELOPMENT	GOBP_EPITHELIAL_CELL_DIFFERENTIATION_INVOLVED_IN_KIDNEY_DEVELOPMENT	0.008592192	ADIPOQ/ACTA2/SIX2/GREM1/WT1/EDNRA/WWTR1/KLF15/SMO/OSR1/FOXC2/MTSS1/MEF2C/CD34/EDNRB
GOBP_RNA_CAPPING	GOBP_RNA_CAPPING	0.008617844	SNRPD2/SNRPD3/SNRPG/RAMAC/NCBP3/RNGTT/SNRPF/SNRPB/ABT1/NCBP1/UTP20/CMTR2/SNRPD1/NOP9/TGS1/TBL3/NOP14

GOCC_EUCHROMATIN	GOCC_EUCHROMATIN	0.008624076	ICE2/HSF1/RUVBL2/EXOSC10/H1-2/SP1/EXOSC5/RRP1B/KLF4/TRIM28/H1-3/EXOSC4/CBX2/H2AZ1/TCF3/NSMF/TRNP1/JAK2/UHRF1
GOBP_OLEFINIC_COMPOUND_METABOLIC_PROCESS	GOBP_OLEFINIC_COMPOUND_METABOLIC_PROCESS	0.008625067	PNLIP/AFP/CEL/ADH1B/PTGIS/ALDH1A2/ELOVL2/TTR/CYP1B1/CACNA1H/RDH8/CYP11A1/ADH1A/ADH7/PLA2G4C/RBP4/DAB2/HSD17B6/CYP2U1/DKKL1/PTGDS/DKK3/PTGS1/LRAT/ALDH1A3/CYP3A4/CYP1A1/ELOVL5/ADH4/FADS2

GOBP_CELLULAR_RESPONSE_TO_INORGANIC_SUBSTANCE	GOBP_CELLULAR_RESPONSE_TO_INORGANIC_SUBSTANCE	0.00864924	SYT4/KCNA1/CER1/FABP4/KCNB1/KCNK3/SLC13A5/RYR3/PRKAA2/TF/CACNA1H/NLGN1/NPTX1/ADCY1/DLG2/NFATC4/SERPINF1/GUCY1B1/MMP3/RASGRP2/HPCA/FOSB/PKD2/PRKN/SHH/MIR342/CLIC4/SYT11/ACER1/LRRK2/DPEP1/DLG4/CPNE8/PRNP/MEF2C/ITPKB/GPLD1/SYT1/SLC30A10/CYP1A1/RASA4B
GOBP_NCRNA_TRANSCRIPTION	GOBP_NCRNA_TRANSCRIPTION	0.008660518	GTF3C3/CDK7/MACROH2A1/GTF3C4/POLR2L/MEPCE/ZC3H4/TCOF1/POLR1E/GTF3C2/SMARCB1/PIH1D1/ICE2/GTF3C6/NCL/MTOR/NOL11/POLR1B/IPPK/MARS1/BRF1/DDX11/GTF3C5/SIRT7/SNAPC4/SMARCA4/CC2D1A/NPM3/TP53/ELL3

GOCC_CYTOSOLIC_SMALL_RIBOSOMAL_SUBUNIT	GOCC_CYTOSOLIC_SMALL_RIBOSOMAL_SUBUNIT	0.008674509	RPS27A/RPS29/EIF2A/RPS20/DHX29/RPS26/RPS25/RPS15A/RPS13/RPS24/RPS21/RPS11/UBA52/RPS8/RPS16/RPS4X/RPS12/RPS10/FAU/RPS27L/RPS14/RPS9/RPS18/RPS3/RPS5/RPS23/MRPS11/RPS3A/RPS7/RPS28/RACK1/RPS19/RPS6/RPS17/RPS15/RPSA/RPS2

GOBP_SIGNAL_TRANSDUCTION_BY_P53_CLASS_MEDIATOR	GOBP_SIGNAL_TRANSDUCTION_BY_P53_CLASS_MEDIATOR	0.008751373	CRADD/BATF/COPS3/MAP2K6/ATR/ARMC10/NDRG1/CDK5RAP3/HINT1/ZNHIT1/BCL3/RFFL/KAT5/SETD9/USP10/MYO6/USP28/RPS7/ATAD5/CHEK2/ING2/HIPK1/RRN3/KMT5A/USP7/BAX/MYC/HNRNPK/MTOR/PML/STK11/PYCARD/KDM1A/SESN2/AKT1/PERP/CASP6/PPM1D/PAXIP1/BRCA2/TRIAP1/URB2/RRM2B/PIDD1/RPS15/YJU2/MSX1/CASP2/AURKB/TFAP4/NBN/AURKA/MSH2/GTSE1/PRMT5/RRS1/CD74/NPM1/MYBBP1A/PLK2/FOXM1/CHEK1/BOP1/PMAIP1/DDIT4/E2F1/TP53/EIF5A/MDM2/AEN/E2F7/TP73/MUC1/ACER2/E2F2/ELL3
GOBP_REGULATION_OF_NCRNA_TRANSCRIPTION	GOBP_REGULATION_OF_NCRNA_TRANSCRIPTION	0.008807197	MACROH2A1/MEPCE/ZC3H4/SMARCB1/PIH1D1/NCL/MTOR/NOL11/IPPK/MARS1/DDX11/SIRT7/SMARCA4/CC2D1A
GOMF_EXODEOXYRIBONUCLEASE_ACTIVITY	GOMF_EXODEOXYRIBONUCLEASE_ACTIVITY	0.008807197	MGME1/RAD9A/APEX2/APTX/DCLRE1B/POLE/ISG20/DCLRE1A/EXO1/FEN1/POLD1

GOBP_RESPONSE_TO_CORTICOSTEROID	GOBP_RESPONSE_TO_CORTICOSTEROID	0.008795756	APOA2/UCN3/BCHE/ADIPOQ/PCSK1/CDO1/SLIT2/GHR/SLIT3/CYP1B1/CPN1/FBXO32/SERPINF1/FIBIN/SCGB1A1/KLF9/FOSB/IL6/FAM107A/AVPR1A/IGFBP7/A2M/ISL1/CCN2/SSTR2/PTGDS/NEFL/STC1/PAPPA/NR3C1/FLT3/GPER1/COL1A1/ACVR1
GOBP_POSITIVE_REGULATION_OF_EXOSOMAL_SECRETION	GOBP_POSITIVE_REGULATION_OF_EXOSOMAL_SECRETION	0.008843346	PDCD6IP/VPS4B/HGS/ATP13A2/SDC1/IFNG/SMPD3
GOBP_REGULATION_OF_INTEGRIN_MEDIATED_SIGNALING_PATHWAY	GOBP_REGULATION_OF_INTEGRIN_MEDIATED_SIGNALING_PATHWAY	0.008859077	LIMS2/FLNA/LAMA2/PRKD1/LAMA1/BST1/NID1/LAMC1/TIMP1
GOBP_BODY_FLUID_SECRETION	GOBP_BODY_FLUID_SECRETION	0.008865076	CSN2/CEL/VIP/NPPB/TACR1/FGF10/CDO1/NPR3/SCT/CAV1/NPR1

GOBP_POSITIVE_REGULATION_OF_SMALL_GTPASE_MEDIATED_SIGNAL_TRANSDUCTION	GOBP_POSITIVE_REGULATION_OF_SMALL_GTPASE_MEDIATED_SIGNAL_TRANSDUCTION	0.008878084	APOA1/RELN/ADCYAP1R1/FGF10/IGF1/NGF/CDON/LPAR4/GPR17/LPAR1/COL3A1/ITPKB/RASGRP4/ROBO1/PDGFRB/GPR20/F2RL2/F2R/F2RL3/GPR65/GPR174/NOTCH2
GOBP_RIG_I_SIGNALING_PATHWAY	GOBP_RIG_I_SIGNALING_PATHWAY	0.008910193	USP15/BIRC2/NLRX1/CLPB/PUM2/PUM1/NPLOC4/UFD1/GPATCH3/DHX58/ANKRD17/DDX60/OAS3/PHB2/OASL/TRIM25/BIRC3/C1QBP/TRIM15
GOBP_ACTIVATION_OF_NF_KAPPAB_INDUCING_KINASE_ACTIVITY	GOBP_ACTIVATION_OF_NF_KAPPAB_INDUCING_KINASE_ACTIVITY	0.00890146	TRAF2/TLR3/TRAF4/CARD14/IRAK1/TNFRSF10B/CARD10/TNFRSF10A
GOBP_ESTABLISHMENT_OF_PROTEIN_LOCALIZATION_TO_TELOMERE	GOBP_ESTABLISHMENT_OF_PROTEIN_LOCALIZATION_TO_TELOMERE	0.00890146	CCT6A/NABP2/ACD/DKC1/TCP1/CCT8/CCT2/CCT3/BRCA2/CCT4/CCT7/CCT5/WRAP53/TERT

GOBP_TERPENOID_METABOLIC_PROCESS	GOBP_TERPENOID_METABOLIC_PROCESS	0.008913209	PNLIP/CEL/ADH1B/ALDH1A2/TTR/CYP1B1/RDH8/ADH1A/ADH7/UGT1A7/RBP4/RLBP1/HSD17B6/CRABP2/RARRES2/LRAT/ALDH1A3/CYP3A4/CYP1A1/RBP1/ADH4/LRP1
GOBP_REGULATION_OF_PROTEIN_EXPORT_FROM_NUCLEUS	GOBP_REGULATION_OF_PROTEIN_EXPORT_FROM_NUCLEUS	0.008948781	TPR/TCF7L2/XPO4/PARK7/XPO1/IFI27/TXN/FRAT1/IL1B/BARD1/YWHAE/ANP32B/CDK5/RANGAP1/SIRT7/UHMK1/SIRT6/SFN
GOCC_MYOFILAMENT	GOCC_MYOFILAMENT	0.00895806	LMOD1/TPM2/TMOD1/MYOM1/ACTA1/TPM1/LMOD3

GOBP_REGULATION_OF_LIPID_LOCALIZATION	GOBP_REGULATION_OF_LIPID_LOCALIZATION	0.008973328	APOA2/APOA1/APOC3/AGTR1/ADIPOQ/CIDEA/ABCA8/CES1/APOB/AGT/CD36/THBS1/CAV1/FABP3/IL6/PRKN/APOA4/SHH/DAB2/MIR27B/GDF9/RETN/ABCA1/LPL/LRAT/MIR10B/PTCH1/ITGB3/ACACB/PLIN2/PLTP/ENPP7/LRP1/MIR613/OXT
GOBP_REGULATION_OF_SPINDLE_ASSEMBLY	GOBP_REGULATION_OF_SPINDLE_ASSEMBLY	0.008985227	CHMP6/EML3/CHMP2B/PDCD6IP/CHMP7/CHMP1A/VPS4B/RNF4/GPSM2/HNRNPU/CHMP5/SASS6/CCSAP/SPAG5/CHMP4C/MAPK15/PLK1/KIF15/STIL
GOCC_CORNIFIED_ENVELOPE	GOCC_CORNIFIED_ENVELOPE	0.008997779	FLG/DSG3/DSC2/PI3/SPRR2D/EVPL/CAPN1/DSG2/ANXA2/KRT17/PKP3/JUP/KLK6/PKP2/CST6/SERPINB5/KRT16/SPRR2E/KLK7/SPRR2B/SPRR2G

GOMF_LIGASE_ACTIVITY_FORMING_CARBON_NITROGEN_BONDS	GOMF_LIGASE_ACTIVITY_FORMING_CARBON_NITROGEN_BONDS	0.009050707	PPCS/GSS/NADSYN1/GATB/GART/ADSS1/TTLL4/ASS1/PFAS/CAD/CTPS1/FPGS/MTHFD1L/TTLL6/NAPRT/MTHFD1/PAICS/TTLL12/CARNS1/CPS1/QRSL1
GOMF_NEUROTRANSMITTER_RECEPTOR_ACTIVITY_INVOLVED_IN_REGULATION_OF_POSTSYNAPTIC_MEMBRANE_POTENTIAL	GOMF_NEUROTRANSMITTER_RECEPTOR_ACTIVITY_INVOLVED_IN_REGULATION_OF_POSTSYNAPTIC_MEMBRANE_POTENTIAL	0.009055589	GRIK3/CHRNA3/GRIK1/GRIK5/GLRB/CHRNB4/GABBR1/GABRR1/CHRNB2/GABRE/GRID1
GOBP_NEURAL_PRECURSOR_CELL_PROLIFERATION	GOBP_NEURAL_PRECURSOR_CELL_PROLIFERATION	0.009054821	KCNA1/SOX10/TRIM71/ASCL1/INSM1/LIMS2/CDH2/PTN/FGF2/EML1/IGF2BP1/DIXDC1/CDON/GLI3/GLI1/HHIP/CX3CR1/SMARCD3/SOX5/FGF13/SHH/SMO/LRRK2/ID4/ILK/PTPRZ1/SLC16A2/VEGFC
GOBP_RELAXATION_OF_CARDIAC_MUSCLE	GOBP_RELAXATION_OF_CARDIAC_MUSCLE	0.009083898	ATP1A2/PLN/HRC/CHGA/RGS2/SLC8A1/PDE5A/GSN/GSTM2

GOBP_CELLULAR_METAL_ION_HOMEOSTASIS	GOBP_CELLULAR_METAL_ION_HOMEOSTASIS	0.009105929	ELANE/CASQ2/ATP1A2/PLN/KCNMA1/CASQ1/JPH2/SCGN/ANK2/AKAP6/F2/SLC8A2/HTR2A/RGN/DMD/RYR3/HTR2B/PRND/FLNA/ATP1B2/CACNA1C/FGF2/TRPC4/TF/PTH1R/TRPC1/CXCL12/CYBRD1/HRC/SLC22A17/PRKD1/AGT/CCL21/SYPL2/SV2A/P2RX1/ATP2B4/CCL19/MICU3/DMPK/CAV1/S100A8/EDNRA/TMEM178A/SLC30A2/SLC8A1/PKD2/BDKRB1/S100A9/PRKCB/CDH23/STEAP4/SLC24A3/SCARA5/CCL11/PRNP/ITPR1/RYR2/STC1/CLIC2/THY1/JPH3/ANXA6/MCOLN3/ITGB3/SLC30A10/P2RY1/GPER1/CCR7/TMPRSS6/SLC46A1/STC2
GOCC_PLATELET_ALPHA_GRANULE_MEMBRANE	GOCC_PLATELET_ALPHA_GRANULE_MEMBRANE	0.009115724	CD36/SELP/PCDH7/SPARC/ITGB3/PECAM1/CD109/SNCA

GOBP_SNRNA_METABOLIC_PROCESS	GOBP_SNRNA_METABOLIC_PROCESS	0.009156426	CDK7/USB1/MEPCE/MTREX/INTS12/ZCCHC8/TUT1/INTS13/INTS7/ICE2/INTS14/INTS9/DKC1/INTS8/EXOSC10/INTS11/INTS1/EXOSC2/EXOSC7/INTS4/EXOSC5/INTS5/EXOSC9/EXOSC4/TOE1/SNAPC4/INTS2/NHP2/CC2D1A/INTS10/ELL3
GOBP_REGULATION_OF_B_CELL_APOPTOTIC_PROCESS	GOBP_REGULATION_OF_B_CELL_APOPTOTIC_PROCESS	0.009187607	AURKB/CD74/ORMDL3/NOC2L/PDCD1/ADA/LYN/IL10
GOMF_LIGAND_GATED_CATION_CHANNEL_ACTIVITY	GOMF_LIGAND_GATED_CATION_CHANNEL_ACTIVITY	0.009202783	GRIK3/CHRNA3/GRIK1/GRIN2A/ABCC9/RYR3/GRIK5/KCNH6/P2RX2/KCNJ8/KCNH2/CHRNB4/P2RX1/PKD2/CNGA1/ITPR1/RYR2/MCOLN3/CHRNB2
GOBP_REGULATION_OF_GASTRULATION	GOBP_REGULATION_OF_GASTRULATION	0.009331478	PHLDB2/CRB2/NODAL/OSR1/TENM4/PHLDB1
GOBP_MULTICELLULAR_ORGANISM_AGING	GOBP_MULTICELLULAR_ORGANISM_AGING	0.009379739	CRYAB/IGFBP1/EDNRA/COMP/AVPR1A/INHBA

GOBP_OLIGODENDROCYTE_DIFFERENTIATION	GOBP_OLIGODENDROCYTE_DIFFERENTIATION	0.009381659	NKX2-2/SOX10/PLP1/VTN/ASCL1/CNTN1/PTN/TRPC4/DAAM2/GLI3/IL33/WASF3/CNTNAP1/GPR17/NKX6-1/LPAR1/MAL/ASPA/TSPAN2/SHH/DUSP15/ID4/TENM4/PTPRZ1/CLU/PRDM8/CXCR4/IL34
GOCC_PERICENTRIC_HETEROCHROMATIN	GOCC_PERICENTRIC_HETEROCHROMATIN	0.009464605	LRWD1/KDM4C/KMT5C/SIRT6/KDM4A/CENPA/EZH2/INCENP/DNMT1/NCAPD3/HELLS/ESCO2
GOBP_REGULATION_OF_CYCLASE_ACTIVITY	GOBP_REGULATION_OF_CYCLASE_ACTIVITY	0.009463202	ADCYAP1/NPFFR2/NPR3/CAP2/CACNA1C/GABBR2/VIPR2/NCS1/EDNRA/RGS2/HPCA/GUCA2A/PALM/ADRB2/GLP1R/AVPR2/GABBR1/ADORA3

GOCC_INTRINSIC_COMPONENT_OF_MITOCHONDRIAL_MEMBRANE	GOCC_INTRINSIC_COMPONENT_OF_MITOCHONDRIAL_MEMBRANE	0.009502255	PPOX/TIMM17B/OXA1L/C12orf73/MFN1/TIMM23/PISD/TIMM23B/TMEM223/ETFDH/TMEM177/TIMM17A/MUL1/PET100/ABCB10/TOMM70/MFN2/TMEM11/BID/SAMM50/TMEM70/CHCHD6/MTX1/MTX2/APOO/CPT1A/MICOS13/MCUB/MPC1/AFG3L2/MICU1/SLC25A3/SCO1/IMMT/RHOT2/SLC8B1/UQCC3/SFXN4/HSPA9/CHCHD3/SLC25A19/GHITM/L2HGDH/COQ2/SFXN2/DNAJC11/TOMM40/SFXN1/COX18/CHCHD10/CCDC51/BAK1/MCU/SCO2

GOBP_ORGANIC_ACID_CATABOLIC_PROCESS	GOBP_ORGANIC_ACID_CATABOLIC_PROCESS	0.009503139	ACOX3/PCCB/ACAD8/SESN2/AKT1/ABCD3/ALDH1L1/ABHD3/PCK2/MPST/GSTZ1/CARNMT1/KYAT3/DCXR/ACADVL/IL4I1/HADH/FABP1/PEX7/ACAA1/CYP26B1/TYSND1/IVD/SLC27A4/AKR1A1/HIBCH/PON1/LPIN2/DECR1/PPARD/CPT2/ILVBL/GOT2/MCAT/BCAT2/ADTRP/PGD/CYP4F12/ACAT2/MFSD2A/SORD/SHMT1/ECI1/MCCC2/FAAH/TST/CYP4F11/GCAT/DDAH1/ALDH4A1/PCK1/ECHS1/ABHD2/NUDT8/GOT1/SLC27A2/SHMT2/ACADS/NOS2/LDHD/GPT2/PRODH/CARNS1/GAD1/GPT/IDO1/GLYATL2
GOBP_SKELETAL_MUSCLE_CELL_DIFFERENTIATION	GOBP_SKELETAL_MUSCLE_CELL_DIFFERENTIATION	0.009528645	SMYD1/MYOCD/ANKRD1/KLHL41/ASB2/RBM24/HLF/CDON/HEYL/SHH
GOBP_REGULATION_OF_NEURAL_PRECURSOR_CELL_PROLIFERATION	GOBP_REGULATION_OF_NEURAL_PRECURSOR_CELL_PROLIFERATION	0.009572905	SOX10/TRIM71/ASCL1/INSM1/LIMS2/CDH2/PTN/FGF2/CDON/GLI3/GLI1/CX3CR1/SMARCD3/SHH/SMO/LRRK2/ID4/ILK/PTPRZ1/SLC16A2/VEGFC

GOMF_TRNA_METHYLTRANSFERASE_ACTIVITY	GOMF_TRNA_METHYLTRANSFERASE_ACTIVITY	0.009625564	LCMT2/TRMT61A/TRMT10C/TRMT2B/TRMT5/NSUN6/NSUN3/TARBP1/ALKBH8/TYW3/THUMPD3/THUMPD2/TRMO/TRMT61B/FTSJ1/TRMT11/METTL2A/METTL2B/TRMT10A/TRMT2A/NSUN2/WDR4/TRMT44/TRMT1
GOBP_REGULATION_OF_ACTIN_FILAMENT_BASED_MOVEMENT	GOBP_REGULATION_OF_ACTIN_FILAMENT_BASED_MOVEMENT	0.009631067	ATP1A2/PLN/CTNNA3/ANK2/ACTA2/CACNA1C/CAV1/FGF13/PDPN/RYR2/STC1
GOMF_CARBOHYDRATE_KINASE_ACTIVITY	GOMF_CARBOHYDRATE_KINASE_ACTIVITY	0.009652052	PFKFB4/XYLB/GALK2/HKDC1/HK3/PFKL/POMK/KHK/PFKP/PFKFB2/HK2
GOBP_HISTONE_H2A_UBIQUITINATION	GOBP_HISTONE_H2A_UBIQUITINATION	0.009657573	BABAM2/TRIP12/UBE2A/UBR2/PCGF2/PCGF3/PCGF1/UBR5/TRIM37/PCGF6/RNF168/DDB1/OTUB1/KDM2B/BRCC3/BRCA1/DTX3L/PCGF5/BCOR/BARD1/BRCA2/DDB2/OTUB2/RAD51

GOMF_THIOLESTER_HYDROLASE_ACTIVITY	GOMF_THIOLESTER_HYDROLASE_ACTIVITY	0.009665357	ACOT11/ABCD1/ABHD10/ABHD17A/PLA2G6/ABCD3/BAAT/PNKD/HIBCH/LYPLA2/LYPLA1/ACOT7/ABHD12B/FASN/THEM5/ABHD17C/HAGHL
GOBP_EMBRYONIC_ORGAN_DEVELOPMENT	GOBP_EMBRYONIC_ORGAN_DEVELOPMENT	0.009669051	HAND1/COL2A1/HAND2/NEUROD1/NKX2-5/RBPMS2/FGF10/ALDH1A2/SCT/ASB2/SIX2/NOG/EFEMP1/RSPO3/NKX3-2/ZFPM2/FRZB/PLXNA4/HOXD10/IGF2/GLI3/ZEB1/MFAP5/NTN1/ITGA8/FOXF1/TGFB3/HOXA4/GLI1/SOBP/EDNRA/CRB2/NODAL/CCN1/TBX18/TGFB2/FBN1/RBP4/PKD2/CDH23/HOXA2/SHH/TAL1/ZNF568/SMO/PDGFRA/OSR1/PBX1/MMP16/PRRX1/SLITRK6/FOXF2/TPO/A2M/PBX3/SOX15/TCF21/FOXC2/ATOH1/PIFO/HOXD4/CC2D2A/TBX15/SOX17/USH1G/RARRES2/RYR2/VASH2/MEF2C/ALDH1A3/PDGFC/PTCH1/MFAP2/TWIST1/HOXA5/CTHRC1/HOXD9/TEAD1/VANGL2/LEF1/KIT/ACVR1

GOBP_RESPONSE_TO_ANTIBIOTIC	GOBP_RESPONSE_TO_ANTIBIOTIC	0.00970105	CPT1A/CASP8/SRD5A1/GJB2/EHMT2/HYAL3/HSPA5/SOD1/KDM1A/GFER/CASP3/EZH2/PLA2G4F/TP53/ENDOG/HYAL1/MDM2/CIITA/CRIP1/SLC9A1/PLA2G4A/JAK2
GOBP_SUBSTRATE_DEPENDENT_CELL_MIGRATION	GOBP_SUBSTRATE_DEPENDENT_CELL_MIGRATION	0.009719657	SLIT2/STON1/NTN1/CSPG4/FN1/SNAI2/VEGFC/ROBO1/ITGA11/NRP1/P2RY12
GOBP_REGULATION_OF_VASCULAR_ENDOTHELIAL_GROWTH_FACTOR_RECEPTOR_SIGNALING_PATHWAY	GOBP_REGULATION_OF_VASCULAR_ENDOTHELIAL_GROWTH_FACTOR_RECEPTOR_SIGNALING_PATHWAY	0.009723616	VTN/FGF10/ITGA5/PRKCB/EMILIN1/MIR10B/ITGB3/MMRN2/VEGFC/NRP1/FZD4/TMEM204/NEDD4
GOBP_NEGATIVE_REGULATION_OF_TELOMERE_MAINTENANCE_VIA_TELOMERASE	GOBP_NEGATIVE_REGULATION_OF_TELOMERE_MAINTENANCE_VIA_TELOMERASE	0.009795043	TENT4B/TERF2/XRN1/ACD/STN1/SRC/NAT10/HNRNPU/HNRNPC/EXOSC10/PIF1/PINX1/HNRNPA1
GOBP_NEGATIVE_REGULATION_OF_OSTEOBLAST_DIFFERENTIATION	GOBP_NEGATIVE_REGULATION_OF_OSTEOBLAST_DIFFERENTIATION	0.009847423	SFRP1/HAND2/NOG/TWIST2/TNN/GREM1/GDF10/CHRD/HOXA2/IGFBP5/PTCH1/TWIST1/VEGFC

GOBP_NEPHRON_EPITHELIUM_DEVELOPMENT	GOBP_NEPHRON_EPITHELIUM_DEVELOPMENT	0.009849068	ADIPOQ/TMEM59L/SIX2/NOG/ADAMTS16/GPC3/FGF2/GREM1/WT1/GLI3/GREB1L/AGT/EDNRA/WWTR1/HEYL/PKD2/KLF15/SHH/SMO/OSR1/PBX1/WNT2B/TCF21/FOXC2/DCHS1/FGF1/ILK/MTSS1/MEF2C/PTCH1/IRX3/CD34/PECAM1/EDNRB
GOBP_POSITIVE_REGULATION_OF_ADAPTIVE_IMMUNE_RESPONSE	GOBP_POSITIVE_REGULATION_OF_ADAPTIVE_IMMUNE_RESPONSE	0.009903722	ATAD5/CYRIB/AZGP1/TBX21/DENND1B/IL18R1/HLA-H/IL1B/PYCARD/TRAF2/TFRC/PAXIP1/HLA-E/IL23A/RIPK2/NECTIN2/HLA-DRA/HLA-B/IL12RB1/HMCES/HLA-A/TYK2/KMT5C/FZD5/IL27RA/MSH2/HSPD1/IL18/PRKCQ/HLA-F/PVR/TAP2/HLA-DRB1/FADD/NSD2/ADA/PRKCZ/CD55/PLA2G4A/JAK2/TNFSF13/CD274

GOCC_MYOSIN_II_COMPLEX	GOCC_MYOSIN_II_COMPLEX	0.009914417	MYH11/MYL9/MYL3/MYH10
GOMF_OUTWARD_RECTIFIER_POTASSIUM_CHANNEL_ACTIVITY	GOMF_OUTWARD_RECTIFIER_POTASSIUM_CHANNEL_ACTIVITY	0.009968208	KCNB1/KCNK3/KCNA5/KCNK2/KCNT2/PKD2/KCND1/KCND2
GOBP_REGULATION_OF_EXTRINSIC_APOPTOTIC_SIGNALING_PATHWAY_VIA_DEATH_DOMAIN_RECEPTORS	GOBP_REGULATION_OF_EXTRINSIC_APOPTOTIC_SIGNALING_PATHWAY_VIA_DEATH_DOMAIN_RECEPTORS	0.010016008	SFRP1/FGA/FGG/SFRP2/THBS1/HGF/MAL/SERPINE1/BMPR1B/FGB/MIR222/TIMP3
GOBP_NEGATIVE_REGULATION_OF_VASCULAR_ASSOCIATED_SMOOTH_MUSCLE_CELL_PROLIFERATION	GOBP_NEGATIVE_REGULATION_OF_VASCULAR_ASSOCIATED_SMOOTH_MUSCLE_CELL_PROLIFERATION	0.010098104	CNN1/ADIPOQ/MYOCD/TPM1/PRKG1/TGFB3/EFEMP2/MEF2C/TAFA5/GPER1

GOBP_NUCLEUS_ORGANIZATION	GOBP_NUCLEUS_ORGANIZATION	0.010109946	EMD/SELENOF/USP36/NUP155/AGFG1/PHF8/NUP107/MFSD14A/ANKLE2/KDM3A/AKAP8L/SPAST/BANF1/NUP133/LPIN1/SUMO1/DCTN1/SMARCA5/LEMD2/SIRT1/NUP153/UBE2I/VPS4A/TMF1/TSSK6/NUP98/NSFL1C/NEK6/TPR/UBXN2B/PYGO2/H3-3B/NUP54/TARDBP/CHMP6/AHCTF1/MACROH2A1/SEH1L/KAT5/SF1/H3-3A/LEMD3/NUP35/TMEM170A/CHMP2B/ZPR1/BAZ2A/ACTR6/PDCD6IP/CHMP7/CHMP1A/RPS19/RRN3/VPS4B/LMNA/SRPK1/PML/CHMP5/NUP93/POLR1B/PLEC/CTDNEP1/NECTIN2/SERBP1/NUP205/HMGB2/SUV39H1/DDX11/VRK1/WRAP53/CDK1/NOLC1/CHEK1/GOLM1/NDC1/CHMP4C/REEP4/PLK1/CEP55/LMNB2/RCC1/IRAG2/LMNB1
GOMF_DNA_DAMAGE_SENSOR_ACTIVITY	GOMF_DNA_DAMAGE_SENSOR_ACTIVITY	0.01016212	RAD50/RAD51D/MSH3/RAD23B/PMS2/PMS1/XRCC2/XPC/MSH6/XRCC3/SIRT6/MSH2/DMC1/RAD51

GOMF_PEPTIDE_LYSINE_N_ACETYLTRANSFERASE_ACTIVITY	GOMF_PEPTIDE_LYSINE_N_ACETYLTRANSFERASE_ACTIVITY	0.010157775	TAF1/CLOCK/KAT6B/EP300/GTF2B/ATAT1/KAT6A/TAF9/USP22/NAA60/HAT1/GTF3C4/KAT5/ESCO1/NAA50/SRCAP/MCM3AP/BRCA2/NAA40/KAT2A/MED24/ESCO2
GOCC_SODIUM_CHANNEL_COMPLEX	GOCC_SODIUM_CHANNEL_COMPLEX	0.010178833	SCN7A/GRIK3/GRIK5/CACNA1H/SCN4B/SCN3A
GOBP_MITOCHONDRIAL_GENOME_MAINTENANCE	GOBP_MITOCHONDRIAL_GENOME_MAINTENANCE	0.010189168	PIF1/SESN2/PARP1/SLC25A33/POLG/RRM2B/DNAJA3/STOML2/STOX1/TWNK/RRM1/LONP1/TP53/TYMP/DNA2/ENDOG
GOBP_MIRNA_MEDIATED_GENE_SILENCING_BY_INHIBITION_OF_TRANSLATION	GOBP_MIRNA_MEDIATED_GENE_SILENCING_BY_INHIBITION_OF_TRANSLATION	0.010243299	MIR483/TRIM71/MIR145/MIR520C/MIR27B/MIR222/MIR10B/MIR148B
GOCC_OUTER_MITOCHONDRIAL_MEMBRANE_PROTEIN_COMPLEX	GOCC_OUTER_MITOCHONDRIAL_MEMBRANE_PROTEIN_COMPLEX	0.010312667	TOMM70/SAMM50/CHCHD6/TOMM22/MTX1/MTX2/APOO/MICOS13/TOMM5/IMMT/HSPA9/CHCHD3/DNAJC11/TOMM40

GOMF_CAMP_BINDING	GOMF_CAMP_BINDING	0.010329677	POPDC2/BVES/PRKAR2B/PDE2A/POPDC3
GOMF_ACTIVE_ION_TRANSMEMBRANE_TRANSPORTER_ACTIVITY	GOMF_ACTIVE_ION_TRANSMEMBRANE_TRANSPORTER_ACTIVITY	0.010344842	SLC5A6/SLC9B2/SLC24A1/TMCO3/ATP2A3/SLC25A11/SLC1A1/COX5B/SLC11A2/SLC37A4/UQCR10/SLC2A13/SLC25A3/COX4I1/SLC25A5/SLC12A7/ATP2C1/ATP6V0B/SLC35B1/ATP2A2/SLC26A1/SLC12A9/COX6A1/SLC8B1/SLC4A2/SLC6A8/SLC3A2/SLC6A9/ATP1A1/SLC34A2/SLC16A3/SLC17A5/SLC9A4/SLC4A8/COX15/COX5A/ATP7B/ABCC3/UQCRFS1P1/UQCRH/SLC12A2/SLC5A1/COX8A/SLC26A6/ATP8A1/ATP5F1B/CLCN3/ATP13A1/CLCN4/TCIRG1/SLC9A7/SLC13A3/MFSD2A/CHP1/SLC10A5/ATP2A1/SLC39A14/SLC28A3/SLC45A4/UQCRC1/ATP13A2/SLC22A18/CYC1/SLC9A2/SLC5A2/ATP2C2/MFSD3/KCNJ11/SLC45A3/SLC25A22/SLC22A1/SLC9A3/SLC4A4/SLC19A1/LETM1/ATP1B1/SLC37A1/SLC9A1/ATP4B/ATP12A [illegible]
GOBP_NEURON_MATURATION	GOBP_NEURON_MATURATION	0.01042278	MYOC/FEV/KCNB1/ADGRB3/LGI4/EDNRA/C1QL1/LRRK2/NRCAM/EDNRB/C3/KCNIP2/GLDN/NTN4/BCL11A

GOMF_FRIZZLED_BINDING	GOMF_FRIZZLED_BINDING	0.010483927	SFRP1/MYOC/RSPO3/WNT9A/NDP/FZD7/WNT2B/WNT10B/DLG4/CTHRC1/FZD1/WNT6/WNT16/WNT5B
GOBP_NEURONAL_ACTION_POTENTIAL	GOBP_NEURONAL_ACTION_POTENTIAL	0.010545536	KCNA1/SCN7A/CACNA1H/CHRNB4/P2RX1/SCN3A/KCND2/FGF12/GPER1/SCN9A
GOBP_VASCULOGENESIS	GOBP_VASCULOGENESIS	0.010553615	MYOCD/NKX2-5/TMEM100/ZFPM2/WT1/FOXF1/GJC1/CAV1/SHH/SMO/HAS2/HEG1/SOX17/RAMP2/QKI/TGFBR3/TBX5/CD34/ACKR3/PDGFRB/RASIP1/NRP1/FZD4/APLNR/RRAS/TIE1/ENG/NTRK2/SOX18/ADM/TEAD2
GOCC_INTRINSIC_COMPONENT_OF_POSTSYNAPTIC_DENSITY_MEMBRANE	GOCC_INTRINSIC_COMPONENT_OF_POSTSYNAPTIC_DENSITY_MEMBRANE	0.010566094	EPHA7/GRIN2A/GRIK5/PLPPR4/CACNG4/ADCY1/RGS7BP/CLSTN2/EFNB3/LRRC4/DLG4/LRRC4C/NRCAM/LRRC4B/GRID1/CNIH2/CLSTN3
GOBP_REGULATION_OF_LEUKOCYTE_TETHERING_OR_	GOBP_REGULATION_OF_LEUKOCYTE_TETHERING_OR_ROLLING	0.010596423	ELANE/CXCL12/CCL21/SELP

GOBP_NEUROMUSCULAR_SYNAPTIC_TRANSMISSION	GOBP_NEUROMUSCULAR_SYNAPTIC_TRANSMISSION	0.010643699	CHRNA3/DTNA/P2RX2/NLGN1/CHRNB4/EGR3/CHRNB2/ADARB1
GOBP_RESPONSE_TO_INTERLEUKIN_6	GOBP_RESPONSE_TO_INTERLEUKIN_6	0.010659363	PTGIS/FGG/C1QTNF4/PID1/CAMP/IL6/MIR149/ST3GAL6/IL6ST/HAMP
GOBP_EMBRYONIC_HEART_TUBE_MORPHOGENESIS	GOBP_EMBRYONIC_HEART_TUBE_MORPHOGENESIS	0.010661123	HAND1/HAND2/NKX2-5/ASB2/NODAL/PKD2/SHH/SMO/SOX17/RYR2/MEF2C/VANGL2/ACVR1/NDRG4/APLNR/MICAL2/ENG/DLL1/NPHP3/SOX18/TEAD2/NOTCH2/ARL13B/CITED2/TGFBR2/TBX2

GOMF_DOUBLE_STRANDED_RNA_BINDING	GOMF_DOUBLE_STRANDED_RNA_BINDING	0.010672094	HSP90AB1/IFIH1/RC3H2/LRRFIP1/OAS1/NLRP6/ADAR/DHX33/ILF2/OAS2/ELAVL1/DHX58/DHX9/DGCR8/EIF4B/HNRNPU/DDX21/TARBP2/STRBP/EIF4H/TFRC/MTDH/APTX/DDX60/SUPV3L1/SLC3A2/TLR3/AGO2/DUS2/OAS3/DHX30/ILF3/YRDC/DDX1/DHX15/MRPL44/HSPD1/TUBA1B/OASL/TUBB4B
GOBP_TRIGLYCERIDE_CATABOLIC_PROCESS	GOBP_TRIGLYCERIDE_CATABOLIC_PROCESS	0.010686325	PNLIPRP2/APOA1/APOC3/APOB/GPIHBP1/APOA4/LPL/PNPLA1/GPLD1
GOBP_B_CELL_APOPTOTIC_PROCESS	GOBP_B_CELL_APOPTOTIC_PROCESS	0.010724795	BCL10/TNFRSF21/BAX/TRAF3IP2/AURKB/CD74/ORMDL3/NOC2L/PDCD1/BAK1/ADA/LYN/IL10

GOBP_DIGESTIVE_TRACT_MORPHOGENESIS	GOBP_DIGESTIVE_TRACT_MORPHOGENESIS	0.010737863	SOX10/SFRP1/RBPMS2/FGF10/SIX2/SFRP2/GLI3/FOXF1/GLI1/NODAL/DACT1/SHH/PDGFRA/TCF21/SOX17
GOBP_REGULATION_OF_ACTOMYOSIN_STRUCTURE_ORGANIZATION	GOBP_REGULATION_OF_ACTOMYOSIN_STRUCTURE_ORGANIZATION	0.010780328	APOA1/SFRP1/MYOC/TACR1/FERMT2/PHLDB2/SERPINF2/TPM1/TGFB3/PPM1E/CGNL1/LPAR1/DLC1/CCN2/ARHGAP28/MIR149/S1PR1
GOBP_MICROVILLUS_ORGANIZATION	GOBP_MICROVILLUS_ORGANIZATION	0.010794664	STK26/SLC9A3R1/MINK1/USH1C/ATP8B1/TNIK/PLS1/MYO1A/KLF5/CDHR2/EZR
GOBP_SOMATIC_DIVERSIFICATION_OF_IMMUNOGLOBULINS_INVOLVED_IN_IMMUNE_RESPONSE	GOBP_SOMATIC_DIVERSIFICATION_OF_IMMUNOGLOBULINS_INVOLVED_IN_IMMUNE_RESPONSE	0.010810955	RNF168/SHLD2/SHLD3/TP53BP1/MAD2L2/RIF1/BATF/ATAD5/TBX21/MSH6/TFRC/PAXIP1/HMCES/UNG/KMT5C/IL27RA/NBN/MSH2/HSPD1/PARP3/EXO1/NSD2/TNFSF13/IL10

GOBP_TELENCEPHALON_DEVELOPMENT	GOBP_TELENCEPHALON_DEVELOPMENT	0.010817788	KCNA1/RELN/ATP1A2/NEUROD1/ASCL1/FOXP2/SCT/SLIT2/CDH2/SLIT3/NPY/ATP1B2/IGF2BP1/DCLK2/DAB1/PLXNA4/DIXDC1/CDON/CXCL12/GLI3/ATP2B4/FAT4/FGF13/LPAR1/FEZ1/SLC8A1/COL3A1/SHH/AVPR1A/SMO/OXTR/LRRK2/ID4/ATOH1/BMERB1/TMEM108/LHX6/NEFL/AVPR2/INHBA/ALDH1A3/TACC1/PHACTR1/ROBO1/CXCR4
GOBP_SUBSTRATE_ADHESION_DEPENDENT_CELL_SPREADING	GOBP_SUBSTRATE_ADHESION_DEPENDENT_CELL_SPREADING	0.010861269	APOA1/MYOC/FGA/BVES/FGG/FERMT2/LIMS2/FBLN1/FLNA/ITGA8/RADIL/DAB2/FN1/FZD7/ANTXR1/PARVA/ILK/HAS2/PDPN/FGB/TEK/LAMC1/ITGB3/MYADM/AXL/NRP1/FZD4/MERTK

GOMF_CALCIUM_ION_TRANSMEMBRANE_TRANSPORTER_ACTIVITY	GOMF_CALCIUM_ION_TRANSMEMBRANE_TRANSPORTER_ACTIVITY	0.010908907	GPM6A/GRIN2A/SLC8A2/RYR3/CACNA1C/TRPC4/CACNG4/CNR1/TRPC1/CACNA1H/CACNB2/NCS1/ATP2B4/TRPM6/SLC8A1/PKD2/CACNA2D3/CACNA2D1/CACNA1A/SLC24A3/ITPR1/RYR2/ANXA6/MCOLN3
GOBP_REGULATION_OF_CELL_MORPHOGENESIS	GOBP_REGULATION_OF_CELL_MORPHOGENESIS	0.010945059	SYT4/APOA1/RELN/MYOC/FGA/BVES/F2/FGG/FERMT2/LIMS2/FBLN1/FLNA/PAK3/ITGA7/PLXNA4/ZNF135/PTPRD/SEMA3E/WASF3/TPM1/CDC42EP3/RND2/LPAR1/RHOJ/PRKN/PALMD/MYH10/DAB2/FN1/PALM/CCL2/DLC1/PARVA/CCL11/EPB41L3/DLG4/PLEKHO1/FGD1/ILK/HAS2/RHOB/PDPN/FGB/SPARC/SYT1/ITGB3/P2RY1/CXCR4/RHOQ/ARC/MYADM/KIT/ARHGAP15/WTIP/NRP1/RDX/FZD4

GOCC_EUKARYOTIC_TRANSLATION_INITIATION_FACTOR_3_COMPLEX	GOCC_EUKARYOTIC_TRANSLATION_INITIATION_FACTOR_3_COMPLEX	0.010979964	EIF3M/EIF3K/EIF3J/EIF3B/EIF3D/EIF3H/EIF3E/EIF3I/COPS5/EIF3A/EIF3G/EIF3CL/EIF3C
GOBP_ORGANELLE_DISASSEMBLY	GOBP_ORGANELLE_DISASSEMBLY	0.011088342	SPAST/NEDD9/HAX1/PLK3/HDAC6/ATG3/SLC25A46/ATG13/FBXL4/UBA5/CSNK2A2/TIGAR/RETREG2/HUWE1/ATG4B/AMBRA1/MUL1/GBF1/CISD2/FBXO7/ATG7/SNX7/UFM1/DENR/EIF2D/PNPLA2/MFN2/GOLGA2/ATG5/PARK7/STX5/VPS13C/KAT5/ASCC2/ATG9A/GSK3A/SPATA33/UFL1/RRP7A/ZFAND1/SLC25A5/SREBF2/PLIN3/SPATA18/ATP5IF1/ASCC3/GFM2/MRRF/ATG2A/ARFIP2/VDAC1/WDR45B/DDRGK1/MTIF2/VRK1/VCP/MARK2/LRBA/GABARAP/FZD5/TEX264/ATG9B/HTT/AURKA/PHB2/CDC37/KIF9/CTTN/CDK1/ATP13A2/TP53/ATG4D/TSPO/HK2/SREBF1
GOBP_POSITIVE_REGULATION_OF_NEURAL_PRECURSOR_CELL_PROLIFERATION	GOBP_POSITIVE_REGULATION_OF_NEURAL_PRECURSOR_CELL_PROLIFERATION	0.011112196	SOX10/ASCL1/INSM1/FGF2/CDON/GLI3/GLI1/CX3CR1/SMARCD3/SHH/SMO/ID4

GOBP_REGULATION_OF_CHOLESTEROL_EFFLUX	GOBP_REGULATION_OF_CHOLESTEROL_EFFLUX	0.011141037	APOA1/ADIPOQ/ABCA8/CES1/CAV1/SHH/MIR27B/ABCA1/PTCH1/PLTP/LRP1/MIR613
GOBP_PROTEIN_INSERTION_INTO_MEMBRANE	GOBP_PROTEIN_INSERTION_INTO_MEMBRANE	0.011240208	BAG6/MAIP1/UBL4A/TOMM70/EMC9/TIMM22/TIMM8B/SAMM50/HSP90AA1/TOMM22/MTX1/MTX2/GET4/HSPA4/BCS1L/EMC1/TIMM29/BAX/EMC8/CCDC47/TOMM5/GET3/SEC61A1/RTP4/NDUFA13/EGFR/SGTA/TIMM13/TOMM40/COX18/EMC6/BBC3/GZMB/GRIN3B
GOBP_PYRIMIDINE_NUCLEOTIDE_CATABOLIC_PROCESS	GOBP_PYRIMIDINE_NUCLEOTIDE_CATABOLIC_PROCESS	0.011400752	ENTPD5/MBD4/NEIL1/SMUG1/OGG1/NT5M/NEIL2/TDG/DPYD/ENTPD7/ENTPD4/NT5C/NTHL1/UNG/DUT/DCTPP1/TYMP
GOMF_CXCR_CHEMOKINE_RECEPTOR_BINDING	GOMF_CXCR_CHEMOKINE_RECEPTOR_BINDING	0.011400752	YARS1/CX3CL1/CXCL2/TFF2/CXCL11/PPBP/CXCL1/CXCL9/CXCL10/CXCL3

GOBP_MIDBRAIN_DEVELOPMENT	GOBP_MIDBRAIN_DEVELOPMENT	0.011397887	PLP1/SFRP1/INA/CMA1/SFRP2/FGF2/SYPL2/CKB/SHH/DKK1/GDF7/GNB4/CXCR2
GOBP_PROTEIN_LOCALIZATION_TO_NUCLEOLUS	GOBP_PROTEIN_LOCALIZATION_TO_NUCLEOLUS	0.011500385	NMD3/NOL8/UTP25/RPF2/RRP7A/POLR1A/MCRS1/RAN/BYSL/WRN/PINX1/RRS1/NPM1/TERT
GOBP_RESPONSE_TO_GROWTH_HORMONE	GOBP_RESPONSE_TO_GROWTH_HORMONE	0.011496741	GDF15/LYN/JAK2/CPS1/CSH1
GOBP_SENSORY_PERCEPTION	GOBP_SENSORY_PERCEPTION	0.011490939	SPX/KCNA1/VIP/TAC1/TAFA4/PENK/COL2A1/KERA/TACR1/OBP2B/GRIN2A/TMEM100/HTR2A/GLRB/EFEMP1/CST4/NPY/COL11A2/P2RX2/REEP2/HMCN1/CNR1/NDN/TUB/BEST2/CXCL12/NCAM2/IL33/GIP/RDH8/CACNB2/CD36/CA6/PIP/PRPH2/GJC1/SOBP/LUM/HOXD1/NDP/CNR2/CRB2/SERPINE2/RBP4/MMP24/BDKRB1/GNAL/RLBP1/CDH23/MIR342/TMEM150C/CCL2/SLITRK6/PIEZO2/FFAR4/CCN3/UCHL1/WNT10B/EPYC/CST2/THRB/SNAI2/CNGA1/CLDN19/TGFBI/USH1G/KCND2/LRAT/TIMP3/NLGN2/PROK2/CRYBA2/RIPOR2/CHRNB2/SEMA5B/VN1R1/P2RY1/CXCR4/ROR1/LEF1/KIT/COL1A1/BEST1/OXT/EDNRB/SCN3B/FZD4/BACE1

GOBP_POSITIVE_REGULATION_OF_INTERFERON_GAMMA_PRODUCTION	GOBP_POSITIVE_REGULATION_OF_INTERFERON_GAMMA_PRODUCTION	0.011552057	EBI3/CRTAM/BCL3/HLA-DPB1/CD226/CYRIB/CD244/IL18R1/HLA-DPA1/IL1B/PYCARD/PTPN22/IL23A/RIPK2/TRIM27/BTN3A2/TLR3/IL12RB1/HLA-A/IRF8/TYK2/HRAS/FZD5/IL27RA/ZFPM1/HSPD1/IL18/F2RL1/SCRIB/FADD/SLC7A5/LGALS9/BTN3A1/JAK2
GOMF_STEROL_TRANSFER_ACTIVITY	GOMF_STEROL_TRANSFER_ACTIVITY	0.011576831	APOA2/APOA1/APOB/APOA4/ABCA1/MTTP/PLTP
GOMF_RNA_POLYMERASE_II_COMPLEX_BINDING	GOMF_RNA_POLYMERASE_II_COMPLEX_BINDING	0.011642293	RPRD2/RTF1/PCIF1/ELP2/PCF11/ZNF326/SCAF8/RPRD1B/AGO1/GTF2B/RPRD1A/CDC73/BRD4/RECQL5/SMYD2/SCAF1/SCAF4/CTR9/LEO1/WDR43/DHX9/UVSSA/HNRNPU/ELOF1/ERCC5/AGO2/CCAR2

GOBP_POSITIVE_REGULATION_OF_BONE_MINERALIZATION	GOBP_POSITIVE_REGULATION_OF_BONE_MINERALIZATION	0.011654243	PTN/GPM6B/TGFB3/CCN1/SLC8A1/TMEM119/OSR1/ADRB2/NELL1/WNT10B/BMPR1B/KL/MEF2C/ACVR1/FAM20C/BMP6
GOBP_NATURAL_KILLER_CELL_MEDIATED_IMMUNITY	GOBP_NATURAL_KILLER_CELL_MEDIATED_IMMUNITY	0.011696301	KLRC1/KLRD1/UNC13D/CD226/PRDX1/PTPN6/CORO1A/ULBP2/HLA-E/GFER/NECTIN2/AP1G1/HLA-B/KLRC2/HLA-A/SLAMF7/SH2D1B/IL18/HLA-F/PVR/NKG7/TUBB4B/RNF19B/LGALS9/ULBP3/KIR2DL4/LAG3/GZMB
GOBP_IRON_SULFUR_CLUSTER_ASSEMBLY	GOBP_IRON_SULFUR_CLUSTER_ASSEMBLY	0.011768978	BOLA1/CIAO1/GLRX5/IBA57/FXN/CIAO2A/GLRX3/BOLA2B/MMS19/NDUFAB1/NUBP1/BOLA3/CIAPIN1/CIAO2B/NUBP2/CIAO3/NDOR1/XDH/HSPA9
GOBP_REGULATION_OF_COLLATERAL_SPROUTING	GOBP_REGULATION_OF_COLLATERAL_SPROUTING	0.011789226	EPHA7/NGF/RND2/LPAR3/FGF13/SPART/CRABP2

GOBP_MULTIVESICULAR_BODY_ORGANIZATION	GOBP_MULTIVESICULAR_BODY_ORGANIZATION	0.011961155	STAM2/SNF8/STAM/RAB27A/CHMP2A/VPS25/IST1/VPS36/VPS37A/VPS37D/VPS4A/UBAP1/MVB12A/VPS37C/CHMP6/VTA1/VPS28/CHMP2B/PDCD6IP/RAB11A/CHMP7/CHMP1A/VPS4B/HGS/CHMP5/VPS37B/CHMP4C
GOBP_MONOCARBOXYLIC_ACID_CATABOLIC_PROCESS	GOBP_MONOCARBOXYLIC_ACID_CATABOLIC_PROCESS	0.011955443	PEX5/GCDH/ABCD1/HSD17B10/SLC25A17/CYP4F2/SCP2/XYLB/CPT1A/ACOX1/PEX2/HSD17B4/HACL1/ACOX3/PCCB/SESN2/AKT1/ABCD3/ABHD3/PCK2/DCXR/ACADVL/HADH/FABP1/PEX7/ACAA1/CYP26B1/TYSND1/IVD/SLC27A4/AKR1A1/LPIN2/DECR1/PPARD/CPT2/ILVBL/MCAT/ADTRP/PGD/CYP4F12/ACAT2/MFSD2A/SORD/ECI1/FAAH/CYP4F11/PCK1/ECHS1/ABHD2/NUDT8/SLC27A2/ACADS/LDHD/GLYATL2

GOBP_BASEMENT_MEMBRANE_ORGANIZATION	GOBP_BASEMENT_MEMBRANE_ORGANIZATION	0.012062141	CMA1/FLRT2/PHLDB2/HMCN1/LAMA2/LAMA1/CAV1/COL3A1/RAMP2/NID1/PHLDB1/LAMC1/NTNG2/PXDN/NTN4/CAV2/NID2
GOBP_ORGANIC_HYDROXY_COMPOUND_METABOLIC_PROCESS	GOBP_ORGANIC_HYDROXY_COMPOUND_METABOLIC_PROCESS	0.012055452	PNLIP/APOA2/CEL/APOA1/LCT/HAND2/TPH1/ADCYAP1R1/ADH1B/CES1/GRIN2A/ALDH1A2/CYP24A1/SULT2A1/NUDT10/APOB/IGF1/INSM1/PAH/TTR/NPY/CYP1B1/PRKAA2/FGF2/FAXDC2/PTH1R/TRPC1/CACNA1H/SOAT2/DIO1/RDH8/SULT1C4/RNF180/LEPR/CYP11A1/CUBN/ADH1A/CH25H/ADH7/VLDLR/EDNRA/CYP7B1/NPR1/TGFB2/RBP4/PDE1B/PRKN/NR1H4/APOA4/DAB2/CGA/NUDT11/HSD17B6/TPO/DIO2/CTSK/ACER1/MALRD1/MOXD1/ZEB2/CYP39A1/HDC/SNAI2/ALDH1B1/DKKL1/FGF1/DKK3/ABCA1/CYP27A1/KL/LRAT/ALDH1A3/ITPKB/SLC16A2/CYP3A4/CPQ/CYP1A1/CHRNB2/SLC16A10/P2RY1/PLTP/MOGAT1/ADH4/TYRP1/GPER1
GOBP_NEGATIVE_REGULATION_OF_DOUBLE_STRAND_BREAK_REPAIR_VIA_HOMOLOGOUS_RECOMBINATION	GOBP_NEGATIVE_REGULATION_OF_DOUBLE_STRAND_BREAK_REPAIR_VIA_HOMOLOGOUS_RECOMBINATION	0.012082899	RECQL5/SHLD2/SHLD3/TP53BP1/MAD2L2/RIF1/SMCHD1/FANCB/UBQLN4/HELB/PARPBP/CGAS/POLQ/RMI2

GOBP_ASSOCIATIVE_LEARNING	GOBP_ASSOCIATIVE_LEARNING	0.012076351	RELN/ATP1A2/TACR1/GRIN2A/NPTX2/NOG/MAP1A/LRRN4/AGT/PDE1B/SNAP25/BRSK1/PDE8B
GOBP_PLATELET_DERIVED_GROWTH_FACTOR_RECEPTOR_SIGNALING_PATHWAY	GOBP_PLATELET_DERIVED_GROWTH_FACTOR_RECEPTOR_SIGNALING_PATHWAY	0.012094045	ADIPOQ/MYOCD/APOD/STON1/PDGFRL/NR4A3/F7/CSPG4/PDGFRA/PDGFD/PTGIR/LOX/PDGFC/ITGB3/PDGFRB/LRP1/NDRG4/NRP1/TXNIP
GOBP_REGULATION_OF_MYOTUBE_DIFFERENTIATION	GOBP_REGULATION_OF_MYOTUBE_DIFFERENTIATION	0.012107544	SMYD1/MYOCD/NKX2-5/RBM24/PLPP7/DMPK/LMOD3/TMEM119/CCN3

GOBP_CARDIAC_SEPTUM_DEVELOPMENT	GOBP_CARDIAC_SEPTUM_DEVELOPMENT	0.012141356	HAND1/NKX2-5/ANK2/SLIT2/NOG/SLIT3/ZFPM2/SALL4/PDE2A/CCN1/TGFB2/NRP2/HEYL/SMO/ISL1/PARVA/HEG1/TGFBR3/TBX5/ROBO1/ACVR1/SEMA3C/NRP1/APLNR/BMP5/FGFR2/FZD1/SOX11/GJA5/ENG/NPHP3/ADAMTS6/TGFBR1/NOTCH2/GATA3
GOMF_OXIDOREDUCTASE_ACTIVITY_ACTING_ON_THE_CH_CH_GROUP_OF_DONORS_NAD_OR_NADP_AS_ACCEPTOR	GOMF_OXIDOREDUCTASE_ACTIVITY_ACTING_ON_THE_CH_CH_GROUP_OF_DONORS_NAD_OR_NADP_AS_ACCEPTOR	0.012164279	AKR1C2/TM7SF2/SRD5A3/DPYD/MECR/ZADH2/DHDH/SRD5A1/BLVRB/TECR/DECR1/DHCR7/CYP2S1/DHCR24/PTGES2/FASN
GOBP_PURINE_NUCLEOSIDE_MONOPHOSPHATE_METABOLIC_PROCESS	GOBP_PURINE_NUCLEOSIDE_MONOPHOSPHATE_METABOLIC_PROCESS	0.012236851	TJP2/GUK1/APRT/IMPDH2/ADK/CASK/NT5C/GART/NUDT2/PNP/ATIC/ADSS1/AK1/IMPDH1/AK2/PFAS/XDH/PRPS2/DCK/PPAT/PAICS/ADA

GOBP_NEGATIVE_REGULATION_OF_STRIATED_MUSCLE_CELL_DIFFERENTIATION	GOBP_NEGATIVE_REGULATION_OF_STRIATED_MUSCLE_CELL_DIFFERENTIATION	0.012374282	MYOCD/NKX2-5/PI16/RGS4/PLPP7/RGS2/TMEM119/DKK1/FZD7/CCN3/MIR222
GOMF_CYTOKINE_RECEPTOR_BINDING	GOMF_CYTOKINE_RECEPTOR_BINDING	0.012368673	KITLG/PIDD1/CCL5/TYK2/TNFSF10/TRAF4/CCL3L3/CASP3/CXCL16/OSM/IL27RA/CD2AP/TGFBR3L/DAB2IP/IL18/STAT1/YARS1/CX3CL1/CCL3/CXCL2/FADD/TRAP1/VEGFD/IL7/BAMBI/TFF2/LRG1/CXCL11/S100A14/PPBP/FASLG/CCL28/CCL23/CXCL1/DEFB1/JAK2/IFNG/TNFSF13/IL36RN/CXCL9/CXCL10/CXCL3/AMH/IFNE/CD70/DEFB4A/TNFSF9/LEFTY1/IL10/CSH1
GOBP_POSITIVE_REGULATION_OF_EXTRACELLULAR_MATRIX_ORGANIZATION	GOBP_POSITIVE_REGULATION_OF_EXTRACELLULAR_MATRIX_ORGANIZATION	0.012450993	TNXB/DDR2/PHLDB2/AGT/IL6/EMILIN1/EFEMP2/PDPN/PHLDB1
GOMF_NEUROPEPTIDE_RECEPTOR_BINDING	GOMF_NEUROPEPTIDE_RECEPTOR_BINDING	0.012450993	UCN3/GHRH/TAC1/ADCYAP1/GNAO1/NPY

GOCC_SPECIFIC_GRANULE	GOCC_SPECIFIC_GRANULE	0.012440875	ELANE/MS4A3/DEFA4/HP/BPI/CEACAM3/MMP8/PTX3/CD36/CHRNB4/P2RX1/CAMP/QPCT/ORM2/TIMP2/SNAP25/PGLYRP1/RETN/STOM/BST1/MCEMP1/SLC2A3/ATP8B4
GOBP_PRESYNAPSE_ORGANIZATION	GOBP_PRESYNAPSE_ORGANIZATION	0.012482965	NLGN4X/NLGN1/PTPRD/NLGN4Y/DKK1/GPC6/SLITRK6/BSN/NLGN3/NLGN2/PCDH17/LRRC4B/FZD1/NTNG2/CLSTN3/SNCA/LRP4/C5AR1/MDGA1
GOBP_TRABECULA_MORPHOGENESIS	GOBP_TRABECULA_MORPHOGENESIS	0.01251232	SFRP1/NKX2-5/NOG/GREM1/ADAMTS1/TGFB2/RBP4/MMP2/WNT10B/S1PR1/HEG1/TGFBR3/TEK/COL1A1/BMP5/ENG/THBS3
GOBP_INSULIN_LIKE_GROWTH_FACTOR_RECEPTOR_SIGNALING_PATHWAY	GOBP_INSULIN_LIKE_GROWTH_FACTOR_RECEPTOR_SIGNALING_PATHWAY	0.01254593	GHRH/IGF1/GHR/AR/CILP/IGFBP1/IGFBP6/IGFBP4/IGFBP5
GOBP_NEGATIVE_REGULATION_OF_	GOBP_NEGATIVE_REGULATION_OF_LIPASE_A	0.01262384	APOA2/APOC3/RGS2

GOBP_POSITIVE_REGULATION_OF_LIPID_CATABOLIC_PROCESS	GOBP_POSITIVE_REGULATION_OF_LIPID_CATABOLIC_PROCESS	0.012663665	APOA2/APOA1/SCT/APOA4/TWIST1/ENPP7
GOBP_POSITIVE_REGULATION_OF_ORGAN_GROWTH	GOBP_POSITIVE_REGULATION_OF_ORGAN_GROWTH	0.012680168	AKAP6/IGF1/ZFPM2/FGF2/IGF2/WT1/GLI1/SMO/TGFBR3/MEF2C/MIR222/ACACB/TBX5/HAMP/FGFR2
GOBP_RENAL_TUBULE_DEVELOPMENT	GOBP_RENAL_TUBULE_DEVELOPMENT	0.01291156	TMEM59L/SIX2/NOG/ADAMTS16/GPC3/FGF2/GREM1/WT1/GLI3/GREB1L/AGT/WWTR1/HEYL/PKD2/SHH/SMO/OSR1/PBX1/WNT2B/TCF21/DCHS1/FGF1/ILK/MTSS1/MEF2C/PTCH1/IRX3
GOBP_RIBOSOMAL_SMALL_SUBUNIT_ASSEMBLY	GOBP_RIBOSOMAL_SMALL_SUBUNIT_ASSEMBLY	0.012920834	MRPS7/RPS27L/RPS14/ERAL1/RPS5/MRPS11/XRCC5/RPS28/ABT1/RPS19/RRP7A/PRKDC/RPS15/RPSA/PWP2
GOMF_CADHERIN_BINDING_INVOLVED_IN_CELL_CELL_ADHESION	GOMF_CADHERIN_BINDING_INVOLVED_IN_CELL_CELL_ADHESION	0.013059024	PDLIM1/BAIAP2L1/EPCAM/PPP1CA/ANXA2/PAK4/PKP3/KRT18/TRIM29/CDC42EP1

GOBP_PROGRAMMED_NECROTIC_CELL_DEATH	GOBP_PROGRAMMED_NECROTIC_CELL_DEATH	0.013103155	CASP8/PELI1/MLKL/ALKBH7/MAP3K5/ADPRS/BAX/IRF3/IPMK/TRAF2/CASP6/FAS/PARP1/AIFM1/TLR3/PPIF/ITPK1/FADD/TP53/BIRC3/PGAM5/FASLG
GOBP_NEGATIVE_REGULATION_OF_HORMONE_SECRETION	GOBP_NEGATIVE_REGULATION_OF_HORMONE_SECRETION	0.013248384	CARTPT/TACR2/SFRP1/ADIPOQ/KCNB1/CHGA/PRKN/FFAR4/CCN3/GJA1/IL11/KLF7/INHBA/INHA
GOBP_RRNA_MODIFICATION	GOBP_RRNA_MODIFICATION	0.01329235	DIMT1/NSUN3/TRMT112/NOP10/BUD23/TFB2M/TRMT61B/TSR3/RPUSD3/FTSJ3/NAF1/METTL15/NSUN4/ZCCHC4/FDXACB1/NAT10/DKC1/NSUN5/FBL/MRM1/NSUN5P1/MRM3/RPUSD2/TFB1M/RPUSD4/GAR1/NHP2/RPUSD1

GOBP_LYMPHOCYTE_ACTIVATION_INVOLVED_IN_IMMUNE_RESPONSE	GOBP_LYMPHOCYTE_ACTIVATION_INVOLVED_IN_IMMUNE_RESPONSE	0.013325675	TBX21/PTK2B/CD244/MSH6/IL18R1/LGALS3/RPS6/CORO1A/PLCG2/MTOR/SHB/CDH17/TFRC/PAXIP1/HLA-DMB/IL23A/RIPK2/AP1G1/HLA-DRA/IL12RB1/KLRC2/CD46/HMCES/IRF8/UNG/KMT5C/IL27RA/NBN/ZFPM1/MSH2/HSPD1/ZBTB7B/IL18/F2RL1/CD74/HLA-F/NKG7/LFNG/ZC3H12A/HLA-DRB1/PARP3/ICAM1/TP53/EXO1/NSD2/PCK1/LGALS9/ADA/PRKCZ/POU2AF1/ZNF683/IFNG/MYB/TNFSF13/IFNE/IL10
GOBP_POSITIVE_REGULATION_OF_RECEPTOR_MEDIATED_ENDOCYTOSIS	GOBP_POSITIVE_REGULATION_OF_RECEPTOR_MEDIATED_ENDOCYTOSIS	0.013576166	VTN/TF/GREM1/SFRP4/CCL21/CCL19/ANGPT1/SERPINE1/DAB2/SGIP1/NTF3/CLU/SELE

GOBP_POSITIVE_REGULATION_OF_IMMUNE_EFFECTOR_PROCESS	GOBP_POSITIVE_REGULATION_OF_IMMUNE_EFFECTOR_PROCESS	0.013593247	KLRD1/UNC13D/CD226/SPHK2/ATAD5/PTPN6/CYRIB/AZGP1/PTAFR/DHX58/BCL10/TBX21/GAB2/DENND1B/CD244/IL18R1/RPS19/HLA-H/PLCG2/IL1B/DDX21/SHB/ANKRD17/PYCARD/TRAF2/TFRC/MYD88/DDX60/VAMP3/PTPN22/SYK/PAXIP1/GPRC5B/HLA-DMB/HLA-E/IL23A/RIPK2/NECTIN2/AP1G1/HLA-DRA/TLR3/HLA-B/IL12RB1/KLRC2/CD46/HMCES/HLA-A/KMT5C/SH2D1B/DDX1/FZD5/TICAM1/MSH2/HSPD1/IL18/STXBP2/F2RL1/PHB2/CD177/CD74/HLA-F/PVR/EPHB2/TNFRSF14/ADORA2B/TAP2/HLA-DRB1/PTPRJ/FADD/GPI/NSD2/SLC7A5/PCK1/LGALS9/KIR2DL4/INAVA/LYN/PRKCZ/NOS2/LAG3/KLK5/CD55/PGC/IFNG/MYB[illegible]
GOBP_ROOF_OF_MOUTH_DEVELOPMENT	GOBP_ROOF_OF_MOUTH_DEVELOPMENT	0.013608262	COL2A1/HAND2/MEOX2/BNC2/COL11A2/GLI3/TGFB3/TGFB2/SHH/PDGFRA/OSR1/PRRX1/FOXF2/TCF21/SNAI2/LRRC32/TGFBR3/CLDN5/INHBA/TWIST1/MSC/LEF1

GOBP_CELL_JUNCTION_MAINTENANCE	GOBP_CELL_JUNCTION_MAINTENANCE	0.013676815	PRTN3/FERMT2/ADGRB3/NLGN1/CNTNAP1/C1QL1/BSN/KIRREL1/DLG4/MTSS1/NLGN2
GOBP_FATTY_ACID_CATABOLIC_PROCESS	GOBP_FATTY_ACID_CATABOLIC_PROCESS	0.013719837	ETFDH/ETFA/ABCD4/LPIN3/EHHADH/NUDT19/PEX13/HADHA/PEX5/GCDH/ABCD1/HSD17B10/SLC25A17/SCP2/CPT1A/ACOX1/PEX2/HSD17B4/HACL1/ACOX3/PCCB/SESN2/AKT1/ABCD3/ABHD3/PCK2/ACADVL/HADH/FABP1/PEX7/ACAA1/TYSND1/IVD/SLC27A4/LPIN2/DECR1/PPARD/CPT2/ILVBL/MCAT/ADTRP/ACAT2/MFSD2A/ECI1/FAAH/PCK1/ECHS1/ABHD2/NUDT8/SLC27A2/ACADS/GLYATL2
GOMF_RNA_POLYMERASE_ACTIVITY	GOMF_RNA_POLYMERASE_ACTIVITY	0.013733148	CRCP/TRNT1/POLR2C/TENT2/POLR1C/POLR2L/POLR3H/POLR2E/POLR1A/POLR3B/POLR3A/POLR2B/POLR1B/POLR2A/POLR3K/PRIM1/POLRMT/TERT/POLR3G

GOBP_MIDDLE_EAR_MORPHOGENESIS	GOBP_MIDDLE_EAR_MORPHOGENESIS	0.013792911	SIX2/NOG/NKX3-2/EDNRA/HOXA2/OSR1/PRRX1
GOBP_ANIMAL_ORGAN_FORMATION	GOBP_ANIMAL_ORGAN_FORMATION	0.013784256	HAND2/FGF10/AR/NOG/NKX3-2/FGF2/WT1/GLI3/SMARCD3/SHH/DKK1/ISL1/WNT2B/FGF1/SOX17/MEF2C/TBX5/ROBO1/SULF1
GOBP_CELLULAR_RESPONSE_TO_PEPTIDE	GOBP_CELLULAR_RESPONSE_TO_PEPTIDE	0.013785557	PLA2G1B/AHSG/IRS4/AGTR1/ACTN2/ADIPOQ/NGFR/SLC2A4/TRARG1/MIR145/GLP2R/SORBS1/GRIN2A/IGF1/GHR/MAP1B/CYP1B1/DDR2/IGFBP1/NR4A3/IGF2/AGT/CD36/CYP11A1/PDK4/LDOC1/PID1/NKX6-1/CAV1/EDNRA/FBN1/KLF15/CACNA2D1/PRKCB/NR1H4/CACNA1A/RAB31/ADRB2/GLP1R/TNS2/GJA1/FOXC2/VIM/SESN3/PRNP/LPL/RARRES2/KL/GPR173/INHBA/FCGR2B/GPLD1/ITGB3/SLC30A10/SOGA1

GOBP_BRANCHED_CHAIN_AMINO_ACID_METABOLIC_PROCESS	GOBP_BRANCHED_CHAIN_AMINO_ACID_METABOLIC_PROCESS	0.013811019	DBT/BCKDHB/AUH/HIBADH/SLC25A44/SDSL/DLD/HSD17B10/MCCC1/ACADSB/HMGCL/PCCB/ACAD8/IVD/HIBCH/ILVBL/BCAT2/MCCC2/ECHS1/BCAT1
GOBP_ANTIGEN_PROCESSING_AND_PRESENTATION_OF_EXOGENOUS_ANTIGEN	GOBP_ANTIGEN_PROCESSING_AND_PRESENTATION_OF_EXOGENOUS_ANTIGEN	0.013833783	HLA-DMB/HLA-E/AP3B1/HLA-DOB/HLA-DRA/HLA-A/HLA-DQA2/MFSD6/HLA-DQA1/PSME1/CTSE/HLA-DRB5/CD74/HLA-F/TAP2/HLA-DRB1/HLA-DOA/HLA-DQB1/DNM2/HLA-DQB2/HLA-DMA/TAP1

GOBP_RESPONSE_TO_MOLECULE_OF_BACTERIAL_ORIGIN	GOBP_RESPONSE_TO_MOLECULE_OF_BACTERIAL_ORIGIN	0.013827332	ELANE/MPO/DEFA4/PENK/BPI/NLRP7/CTSG/FGF10/PCSK1/ANKRD1/APOB/SSC5D/PTGFR/IL24/DEFA6/CNR1/CXCL6/TRIM6/THPO/KCNJ8/FMO1/CD36/AKAP12/LDOC1/CAMP/PLSCR4/CSF3/S100A8/CX3CR1/CNR2/SELP/SCGB1A1/C4B/BDKRB1/IL6/S100A9/TIMP4/NR1H4/SERPINE1/MIR342/CCL2/DIO2/VIM/PTGIR/PTGER1/THBD/ABCA1/MEF2C/LILRA2/LY96/LY86/FCGR2B/SELE/CYP1A1
GOBP_REGULATION_OF_PROTEIN_KINASE_C_SIGNAL	GOBP_REGULATION_OF_PROTEIN_KINASE_C_SIGNALING	0.013875764	SEZ6L/SEZ6/DGKG/AKAP12
GOCC_PROTON_TRANSPORTING_ATP_SYNTHASE_COMPLEX	GOCC_PROTON_TRANSPORTING_ATP_SYNTHASE_COMPLEX	0.013888164	ATP5MC2/DMAC2L/ATP5MG/ATP5MF/ATP5F1C/ATP5PD/ATP5F1EP2/ATP5PO/ATP5MC1/ATP5PB/ATP5MC3/ATP5F1A/ATP5F1B/ATP5ME/ATP5F1D

GOBP_REGULATION_OF_MITOCHONDRIAL_TRANSLATION	GOBP_REGULATION_OF_MITOCHONDRIAL_TRANSLATION	0.013945326	METTL8/NGRN/MALSU1/ALKBH1/CDK5RAP1/FASTKD3/TRMT10C/UQCC2/NSUN3/FASTKD2/RMND1/MTG2/RCC1L/RPUSD3/TRUB2/TACO1/MTG1/MPV17L2/LRPPRC/RPUSD4/MRPS27/C1QBP/SHMT2
GOMF_POTASSIUM_CHANNEL_REGULATOR_ACTIVITY	GOMF_POTASSIUM_CHANNEL_REGULATOR_ACTIVITY	0.013957287	DPP6/KCNMB1/ABCC9/KCNIP1/FLNA/KCNE4/CAV1/ADRB2/KCNS1/KCNAB1/LRRC26/AMIGO1/KCNIP2/KCNS3
GOMF_PHOSPHORUS_OXYGEN_LYASE_ACTIVITY	GOMF_PHOSPHORUS_OXYGEN_LYASE_ACTIVITY	0.013994264	ADCY5/ADCY2/ADCY1/GUCY1A1/GUCY1B1/NPR1/GUCY1A2/BST1/NPR2/MOCS1

GOBP_TUMOR_NECROSIS_FACTOR_SUPERFAMILY_CYTOKINE_PRODUCTION	GOBP_TUMOR_NECROSIS_FACTOR_SUPERFAMILY_CYTOKINE_PRODUCTION	0.013996027	AZU1/ADIPOQ/BPI/CIDEA/IGF1/C1QTNF4/MMP8/IL33/CD36/NFATC4/AKAP12/THBS1/CCL19/CX3CR1/ANGPT1/IL6/ZBTB20/NR1H4/ORM2/SYT11/MIR27B/ISL1/LILRA4/LRRK2/MIR149/LPL/HSPB1/GPNMB/LILRA2/LY96/CLU/TWIST1
GOCC_ANAPHASE_PROMOTING_COMPLEX	GOCC_ANAPHASE_PROMOTING_COMPLEX	0.014030606	ANAPC11/ANAPC7/ANAPC16/CUL7/CDC27/CDC26/ANAPC4/ANAPC1/CDC23/ANAPC15/ANAPC5/ANAPC2/FZR1/UBE2S/BUB1B/CDC20
GOBP_NEGATIVE_REGULATION_OF_BLOOD_CIRCULAT	GOBP_NEGATIVE_REGULATION_OF_BLOOD_CIRCULATION	0.014025723	SPX/ATP1A2/PLN
GOBP_RESPONSE_TO_VITAMIN	GOBP_RESPONSE_TO_VITAMIN	0.014105838	BCHE/PENK/SFRP1/ASCL1/ALDH1A2/CYP24A1/MAP1B/F7/MN1/KANK2/FOLR2/SNAI2/STC1/KL/LRAT/CYP1A1/COL1A1/STC2/HAMP

GOBP_RECEPTOR_MEDIATED_ENDOCYTOSIS	GOBP_RECEPTOR_MEDIATED_ENDOCYTOSIS	0.01413551	APOC3/ADIPOQ/VTN/RSPO1/HTR2B/TF/GREM1/CACNG4/SFRP4/LYVE1/CALY/CD36/CCL21/CUBN/CCL19/VLDLR/CAV1/ANGPT1/HPCA/SERPINE1/DAB2/SNAP25/RAB31/DKK1/ADRB2/SGIP1/MIR27B/NTF3/NLGN3/PDLIM7/TMEM108/DLG4/ASGR1/RAMP2/USH1G/CXCR2/CXCR1/WASF1/CLU/FCGR2B/ITGB3/SELE/LDLRAD3/ARC/ACKR3/LRP1/RAMP3/CLTCL1/C3/DNM1/RAMP1/PLA2R1/NEDD4/MRC1

GOBP_GOLGI_ORGANIZATION	GOBP_GOLGI_ORGANIZATION	0.014127145	CORO7/LMAN1/RAB2A/TRIP11/PLK3/COG3/CSNK1D/CUL7/BAG5/USP6NL/NPLOC4/GORASP1/SEC23IP/VPS13B/TRAPPC12/CSNK1A1/COG6/TMED2/HUWE1/COG5/NSFL1C/COG8/GBF1/STK25/TRAPPC11/COG1/TMED4/UBXN2B/USO1/SEC22B/MYO18A/ARL1/COG4/GOLGA2/STX5/GOLGB1/VCPIP1/GORASP2/GOLGA2P5/ARHGAP21/TMED5/TMED7/COG2/VTI1A/VPS51/TMED9/MAP2K1/MAPK3/YWHAZ/GOLPH3L/SURF4/COG7/ARFGEF1/MAP2K2/ZW10/RAB1B/TMED3/LMAN2/RAB8A/FHDC1/HOOK1/VRK1/STX18/RAB43/HTT/SEC16A/TMED1/PRMT5/ATP8B1/CDK1/CIT/GAK/DNM2/PI4K2B/CAMSAP3/SPTBN5

GOBP_MEMBRANE_FISSION	GOBP_MEMBRANE_FISSION	0.014167127	EXOC4/EXOC3/EXOC8/STAM2/SNF8/STAM/EXOC7/CHMP2A/VPS25/SPAST/MIEF2/SLC25A46/VPS36/EXOC2/VPS37A/VPS37D/VPS4A/UBAP1/EXOC1/MVB12A/VPS37C/CHMP6/VPS28/CHMP2B/CHMP7/CHMP1A/VPS4B/HGS/CHMP5/EXOC6/VPS37B/CHMP4C
GOBP_ESTABLISHMENT_OF_SISTER_CHROMATID_COHESION	GOBP_ESTABLISHMENT_OF_SISTER_CHROMATID_COHESION	0.014195428	MAU2/STAG2/SMC3/WAPL/RB1/STAG1/NAA50/RAD21/SMC1A/DDX11/DDX12P/CDCA5
GOBP_GRANULOCYTE_CHEMOTAXIS	GOBP_GRANULOCYTE_CHEMOTAXIS	0.014198087	PLA2G1B/CCL16/THBS4/SLIT2/CCL26/JAM3/CXCL6/CCL21/ITGA9/THBS1/CCL19/S100A8/TGFB2/ITGA1/S100A9/CCL2/CCL11/BST1/CXCR2/CD300H/RARRES2/CAMK1D/SCG2/CXCR1/S100A12/RIPOR2/MPP1/CCR7/IL34

GOBP_MONOUBIQUITINATED_PROTEIN_DEUBIQUITINATION	GOBP_MONOUBIQUITINATED_PROTEIN_DEUBIQUITINATION	0.014209049	TAF6/ENY2/ATXN7/USP47/USP15/TAF4/MYSM1/TAF9/TAF12/TAF2/TADA3/TRRAP/TAF5L/USP22/TADA1/BAP1/TADA2B/TAF6L/ATXN7L3/USP7/KAT2A/SF3B5/TAF5/SF3B3/USP1
GOBP_REPRODUCTIVE_BEHAVIOR	GOBP_REPRODUCTIVE_BEHAVIOR	0.014235044	VIP/TAC1/TACR1/CNR1/P2RX1/SERPINE2/AVPR1A/OXTR/THRB/NR3C1/P2RY1/OXT/EDNRB
GOBP_CELLULAR_RESPONSE_TO_UV	GOBP_CELLULAR_RESPONSE_TO_UV	0.014231244	H2AW/USP28/XPC/POLA1/CUL4A/POLH/PTPRK/BAX/INO80/MYC/HYAL3/RUVBL2/PRKCD/STK11/KDM1A/PPID/PARP1/EIF2S1/CARD16/TRIAP1/MAPK13/AURKB/POLD3/NPM1/TMEM161A/CHEK1/DDB2/PCNA/TP53/NOC2L/BAK1/HYAL1/POLD1/CDC25A/CRIP1/PBK

GOMF_PHOSPHATIDYLCHOLINE_BINDING	GOMF_PHOSPHATIDYLCHOLINE_BINDING	0.014344613	APOA2/APOA1/SERPINA5/APOA4/ABCA1/PCYT1B/PLTP
GOBP_PROTEIN_LOCALIZATION_TO_CELL_JUNCTION	GOBP_PROTEIN_LOCALIZATION_TO_CELL_JUNCTION	0.014443114	RELN/NRXN2/GRIN2A/FERMT2/ZDHHC15/JAM3/MAP1A/NLGN1/NPTX1/MAPT/DLG2/CGNL1/SNAP25/GPC6/BSN/KIF5A/DLG4/HEG1/HSPB1/NLGN2
GOBP_ACYLGLYCEROL_HOMEOSTASIS	GOBP_ACYLGLYCEROL_HOMEOSTASIS	0.014493226	APOA1/APOC3/GPIHBP1/GIP/PLA2G12B/NR1H4/APOA4/LPL
GOBP_REGULATION_OF_HOMOTYPIC_CELL_CELL_ADHESION	GOBP_REGULATION_OF_HOMOTYPIC_CELL_CELL_ADHESION	0.014475132	CELA2A/CTSG/FGG/ADAMTS18/MMRN1/PRKG1/SERPINE2/IL6/PDPN/BLK/IL6ST/RDX

GOBP_MEIOTIC_CHROMOSOME_SEGREGATION	GOBP_MEIOTIC_CHROMOSOME_SEGREGATION	0.014478017	EHMT2/FANCM/SLX4/NUF2/EME1/MUS81/NCAPH2/RAD21/SMC4/TTK/SMC1A/SGO1/SIRT7/BUB1/RMI1/DMC1/SMC2/PTTG1/EME2/CENPX/BUB1B/NDC1/NCAPH/FANCD2/ESPL1/MAPK15/PLK1/TOP2A/CCNE2/SYCE2/BRIP1/REC8
GOBP_POSITIVE_REGULATION_OF_REPRODUCTIVE_PROCESS	GOBP_POSITIVE_REGULATION_OF_REPRODUCTIVE_PROCESS	0.014497499	VIP/TACR2/TACR1/BNC1/AR/ZFPM2/CACNA1H/WT1/PDE3A/SHH/RETN/OXTR/SYDE1/PDE5A/INHBA/P2RY1/OXT/EDNRB

GOBP_REGULATION_OF_PEPTIDYL_TYROSINE_PHOSPHORYLATION	GOBP_REGULATION_OF_PEPTIDYL_TYROSINE_PHOSPHORYLATION	0.014470702	RELN/SFRP1/ADIPOQ/VTN/THBS4/FGF10/EPHA7/GFRA1/HTR2A/IGF1/FGF7/GHR/EPGN/SFRP2/CNTN1/IL24/GREM1/ALKAL1/IGF2/AGT/CD36/TSLP/CSF3/CAV1/CSPG4/HGF/ANGPT1/ITGA5/IL6/CTF1/CRLF1/ISL1/NTF3/DOCK3/PDGFD/DLG4/NEURL1/IL11/PRNP/BST1/NOX4/THY1/PDGFC/ITGB3/FLT3/IL6ST/IL34/KIT/ACVR1/PLPP3/PECAM1/NRP1/TNFSF18/HPX/CSF2
GOBP_NEUROINFLAMMATORY_RESPONSE	GOBP_NEUROINFLAMMATORY_RESPONSE	0.014515479	AZU1/IGF1/MMP8/ADCY1/IL33/MAPT/CX3CR1/MMP3/IL6/SMO/SYT11/LRRK2/CD200
GOBP_RNA_SURVEILLANCE	GOBP_RNA_SURVEILLANCE	0.014552489	ZCCHC7/DXO/EXOSC8/ZC3H4/XRN1/EXOSC10/EXOSC2/EXOSC7/EXOSC5/SUPV3L1/EXOSC9/EXOSC4
GOBP_ATRIAL_CARDIAC_MUSCLE_TISSUE_DEVELOPMENT	GOBP_ATRIAL_CARDIAC_MUSCLE_TISSUE_DEVELOPMENT	0.014583083	NKX2-5/BVES/NOG/EDNRA/TBX18/ISL1/TBX5

GOBP_POSITIVE_REGULATION_OF_B_CELL_ACTIVATION	GOBP_POSITIVE_REGULATION_OF_B_CELL_ACTIVATION	0.014618512	IGHV4-61/TFRC/SYK/PAXIP1/IGHV3-30/TRDC/HMCES/TNIP2/KMT5C/IGLL5/IGHA1/TICAM1/MSH2/CD74/EPHB2/IGHD/CD27/IGHV3-72/IGHV3-64D/IGHV3-33/NSD2/IGHV3-48/IGHV3-66/IL7/ADA/CD320/CD38/IGHA2/IGHV1-58/IGHM/IGHV3-49/TNFSF13/IGHV3-13/IL10/IGLL1
GOBP_QUINONE_METABOLIC_PROCESS	GOBP_QUINONE_METABOLIC_PROCESS	0.014686365	NDUFA9/UBIAD1/PPTC7/CYP4F2/NQO2/CBR4/COQ8B/PDSS2/AKR7A2/PDSS1/COQ9/COQ4/AKR1A1/COQ3/NQO1/COQ2/CYP4F12/AIFM2/CYP4F11/AKR1B10/FDXR
GOBP_NEGATIVE_REGULATION_OF_MYELOID_LEUKOCYTE_DIFFERENTIATION	GOBP_NEGATIVE_REGULATION_OF_MYELOID_LEUKOCYTE_DIFFERENTIATION	0.014655051	CARTPT/SFRP1/ADIPOQ/LRRC17/TMEM178A/FBN1/TNFRSF11B/INHBA/INHA

GOBP_TISSUE_REGENERATION	GOBP_TISSUE_REGENERATION	0.014679452	FGF10/SGCA/APOD/IGF1/GAP43/PTN/IGFBP1/MYMX/HOPX/FZD7/CCN3/SOX15/WNT10B
GOBP_HETEROTYPIC_CELL_CELL_ADHESION	GOBP_HETEROTYPIC_CELL_CELL_ADHESION	0.014693581	APOA1/ADIPOQ/FGA/CTNNA3/FGG/ITGA7/JAM3/ITGA5/PARVA/CD200/FGB/THY1/NRCAM/ITGB3/MYADM
GOBP_LONG_CHAIN_FATTY_ACID_TRANSPORT	GOBP_LONG_CHAIN_FATTY_ACID_TRANSPORT	0.014693581	PLA2G1B/SPX/FABP4/PLA2G5/PLA2G2C/CD36/PLA2G12B/THBS1/SLC27A6/FABP3

GOCC_RNAI_EFFECTOR_COMPLEX	GOCC_RNAI_EFFECTOR_COMPLEX	0.014646285	CARMN/MIR145/MIR320C2/MEG3/MIR938/DNM3OS/MIR604/MIR342/MIR27B/MIR326/MIR320D1/MIR149/MIR23B/MIR222/MIR32/MIR10B/MIR148B/MIR648/MIR196A1/MIR576/MIR624/MIR1183/MIR339/MIR1296/MIR181B2/MIR331/MIR454/MIR29B2CHG/MIR320B2/MIR320E/MIR221/MIR194-1
GOBP_HISTONE_MRNA_METABOLIC_PROCESS	GOBP_HISTONE_MRNA_METABOLIC_PROCESS	0.014707358	TENT4B/MTPAP/TENT2/CPSF2/ZNF473/CPSF3/TUT7/XRN1/TUT1/NCBP1/EXOSC10/UPF1/EXOSC4/SLBP/ERI1

GOBP_REGULATION_OF_LIPID_TRANSPORT	GOBP_REGULATION_OF_LIPID_TRANSPORT	0.014716797	APOA2/APOA1/APOC3/AGTR1/ADIPOQ/ABCA8/CES1/AGT/THBS1/CAV1/FABP3/PRKN/APOA4/SHH/DAB2/MIR27B/GDF9/RETN/ABCA1/LRAT/PTCH1/ITGB3/PLTP/ENPP7/LRP1/MIR613/OXT
GOMF_STRUCTURAL_CONSTITUENT_OF_CYTOSKELETON	GOMF_STRUCTURAL_CONSTITUENT_OF_CYTOSKELETON	0.01484332	SYNM/TUBA3C/DES/INA/ANK2/DMD/KRT5/TUBB4A/ACTA1/ADD2/TPM1/TUBA1A/TUBB6/KRT15/PRPH/SORBS2/VIM/EPB41L3/NEFL
GOBP_ATRIAL_CARDIAC_MUSCLE_CELL_TO_AV_NODE_CELL_SIGNALING	GOBP_ATRIAL_CARDIAC_MUSCLE_CELL_TO_AV_NODE_CELL_SIGNALING	0.014919036	ANK2/KCNA5/FLNA/CACNA1C/CACNB2/GJC1/GJA1/RYR2/SCN3B/GJA5/KCNJ5

GOBP_PYRIMIDINE_NUCLEOTIDE_METABOLIC_PROCESS	GOBP_PYRIMIDINE_NUCLEOTIDE_METABOLIC_PROCESS	0.014923036	ENTPD5/UCK1/MBD4/NEIL1/SMUG1/UCKL1/OGG1/ENPP3/NME1/NME3/NT5M/NEIL2/DHODH/TDG/DPYD/CMPK2/UMPS/ENTPD7/NME6/ENTPD4/UCK2/DCTD/NT5C/NTHL1/UNG/CAD/CTPS1/DTYMK/CDA/DCK/DUT/DCTPP1/CMPK1/SHMT1/TYMP/TYMS
GOBP_POSITIVE_REGULATION_OF_NATURAL_KILLER_CELL_ACTIVATION	GOBP_POSITIVE_REGULATION_OF_NATURAL_KILLER_CELL_ACTIVATION	0.015012171	IL15RA/HLA-E/IL23A/AP1G1/KLRC2/TYK2/TICAM1/IL18/HLA-F/JAK2
GOBP_NEGATIVE_REGULATION_OF_TYPE_I_INTERFERON_MEDIATED_SIGNALING_PATHWAY	GOBP_NEGATIVE_REGULATION_OF_TYPE_I_INTERFERON_MEDIATED_SIGNALING_PATHWAY	0.015017909	MUL1/OAS1/ADAR/YTHDF2/CNOT7/CACTIN/USP18/OAS3/MMP12/TTLL12/NLRC5
GOMF_POLYSACCHARIDE_BINDING	GOMF_POLYSACCHARIDE_BINDING	0.015001029	PRG4/VTN/PPP1R3C/PTX3/ENDOU

GOMF_SECONDARY_ACTIVE_TRANSMEMBRANE_TRANSPORTER_ACTIVITY	GOMF_SECONDARY_ACTIVE_TRANSMEMBRANE_TRANSPORTER_ACTIVITY	0.015093742	SLC35D1/SLC2A12/SLC10A7/SLC15A3/CLCN5/SLC5A6/SLC9B2/SLC24A1/TMCO3/SLC25A11/SLC1A1/SLC11A2/SLC37A4/SLC2A13/SLC22A5/SLC25A3/SLC25A5/SLC12A7/SLC35B1/CDH17/SLC26A1/SLC12A9/SLC8B1/SLC4A2/SLC6A8/SLC35D2/SLC25A15/SLC3A2/SLC6A9/SLC1A5/SLC34A2/SLC16A3/SLC17A5/SLC9A4/SLC4A8/SLC12A2/SLC5A1/SLC26A6/SLC16A5/CLCN3/SLC35E4/SLC35C1/CLCN4/SLC9A7/SLC13A3/MFSD2A/CHP1/SLC16A8/SLC6A19/SLC16A1/SLC10A5/SLC25A10/SLC39A14/SLC28A3/SLC45A4/SLC22A18/SLC9A2/SLC5A2/MFSD3/SLC7A5/SLC45A3/SLC25A22/SLC22A1/MFSD12/SLC9A3/SLC4A4/SLC19A1/LETM1/SLC35D3/SLC37A1/MCU/SLC9A1/SLC4A11/SLCO4A1/SLC7A11/SLC6A14/SLC26A9/[illegible]
GOBP_RESPONSE_TO_ISOQUINOLINE_ALKALOID	GOBP_RESPONSE_TO_ISOQUINOLINE_ALKALOID	0.015138856	PENK/TACR1/PCSK1/GHR/RGS4/CNR1/FOSB

GOBP_REGULATION_OF_INTRINSIC_APOPTOTIC_SIGNALING_PATHWAY	GOBP_REGULATION_OF_INTRINSIC_APOPTOTIC_SIGNALING_PATHWAY	0.01514359	PARK7/RPS3/BID/PIAS4/ERP29/BAD/BCL2L1/SGMS1/UBQLN1/NONO/CREB3/FIGNL1/TMBIM6/RPS7/ATAD5/NDUFS3/SELENOS/PLAUR/ING2/SFPQ/RACK1/PTPMT1/SYVN1/PIK3CB/RRN3/BAX/MYC/HNRNPK/SRC/DNAJA1/SOD1/PYCARD/KDM1A/AKT1/NDUFA13/PPIA/PARP1/BCAP31/SLC9A3R1/TRIAP1/RRM2B/TXNDC12/MSX1/DDIAS/CCAR2/IVNS1ABP/HDAC1/PDX1/NKX3-1/P4HB/CD74/TMEM161A/CREB3L1/HYOU1/PPIF/VDAC2/PMAIP1/VNN1/TRAP1/TP53/ENO1/NOC2L/EIF5A/HELLS/MDM2/RTKN2/BBC3/LCK/IL20RA/MUC1/ELL3

GOBP_RESPIRATORY_SYSTEM_DEVELOPMENT	GOBP_RESPIRATORY_SYSTEM_DEVELOPMENT	0.015176415	MYOCD/FGF10/ASCL1/ALDH1A2/PGR/FGF7/NOG/GPC3/ZFPM2/FGF2/WT1/GLI3/FOXF1/RSPO2/TGFB3/LAMA1/GLI1/HHIP/NODAL/CRISPLD2/RBP4/COL3A1/HOPX/SHH/PDGFRA/CCBE1/HSD11B1/IGFBP5/WNT2B/TCF21/THRB/CCN2/FGF1/LOX/HEG1/PDPN/LTBP3/EDA/ALDH1A3/TBX5/HOXA5/MSC/LEF1/PDGFRB/SLC23A1/GLI2
GOBP_SYNAPSE_MATURATION	GOBP_SYNAPSE_MATURATION	0.015196043	SEZ6L/RELN/CHRDL1/SEZ6/NLGN1/NFATC4/CX3CR1/PALM/NEURL1/NEFL/NFIA/ARHGEF15/IGSF21

GOCC_PML_BODY	GOCC_PML_BODY	0.01520753	SENP2/SUMO1/SIRT1/NR2C1/SRSF2/MAX/RPAIN/DAXX/UBE2I/TOPORS/RNF111/TDG/ZNF451/SPTBN4/SUMO2/RNF6/TCF7L2/RPA2/CALCOCO2/SMC5/ATR/PARK7/PIAS4/RB1/ELF4/EIF3E/TOP3A/CHEK2/HIPK1/RPA1/HIRA/USP7/RNF4/TOPBP1/HSF1/MTOR/PML/UBN1/LRCH4/TRIM27/PATL1/IKBKE/TRIM16/RFWD3/NBN/ISG20/RGS14/BLM/MKNK2/TP53/RAD51/TERT/RDM1/CIITA
GOBP_CELLULAR_RESPONSE_TO_TOXIC_SUBSTANCE	GOBP_CELLULAR_RESPONSE_TO_TOXIC_SUBSTANCE	0.015271037	ALB/MPO/HP/FBLN5/CD36/S100A9/PRKN/APOA4/HBB/SOD3/GPX3/TPO/GPX7/PTGS1/CLIC2/GPX8/H19/NXN/ADH4/LTC4S/HBA2
GOBP_POSITIVE_REGULATION_OF_SMOOTHENED_SIGNALING_PATHWAY	GOBP_POSITIVE_REGULATION_OF_SMOOTHENED_SIGNALING_PATHWAY	0.015298623	SFRP1/GPC3/GLI1/EVC/SHH/SMO/DHH/PRRX1/RAB34/SCUBE1/DYNC2H1/CIBAR1/DCDC2/ARMC9

GOBP_CARBOHYDRATE_HOMEOSTASIS	GOBP_CARBOHYDRATE_HOMEOSTASIS	0.015298977	GCG/CARTPT/ADIPOQ/NEUROD1/KCNB1/NGFR/SLC2A4/CMA1/ADCY5/PTPRN/GRIK5/PRKAA2/MIR320C2/CNR1/NPTX1/LEPR/PDK4/RFX6/SERPINF1/RBP4/KLF15/IL6/ZBTB20/NR1H4/IGFBP5/CCN4/MIR320D1/GPR27/SESN3/KLF7/NOX4/PPP1R3G/PTCH1/GPLD1
GOBP_INTRINSIC_APOPTOTIC_SIGNALING_PATHWAY_IN_RESPONSE_TO_ENDOPLASMIC_RETICULUM_STRESS	GOBP_INTRINSIC_APOPTOTIC_SIGNALING_PATHWAY_IN_RESPONSE_TO_ENDOPLASMIC_RETICULUM_STRESS	0.015329196	PARK7/ERP29/BCL2L1/CREB3/TMBIM6/SELENOS/ATP2A3/SYVN1/MAP3K5/BAX/PML/TRAF2/APAF1/AIFM1/TXNDC12/CASP4/PDX1/DAB2IP/CREB3L1/HYOU1/ATP2A1/PMAIP1/ERO1A/TNFRSF10B/TP53/BAK1/BBC3/ERN2/CHAC1

GOMF_ALDO_KETO_REDUCTASE_NADP_ACTIVITY	GOMF_ALDO_KETO_REDUCTASE_NADP_ACTIVITY	0.015358176	AKR7A2/RDH12/RDH13/AKR1A1/DHRS4/SPR/DHRS13/AKR1B10/MIOX/AKR7L/ALDH3A1/AKR7A3/AKR1B15
GOBP_RESPONSE_TO_IONIZING_RADIATION	GOBP_RESPONSE_TO_IONIZING_RADIATION	0.015355563	FBXO4/ATR/NABP2/RAD9A/MTA1/RAD9B/NHEJ1/KAT5/BCL2L1/SPIDR/XRCC5/XRCC2/FIGNL1/USP28/GRB2/CHEK2/EEF1D/APOBEC1/BRCC3/BRCA1/INTS7/POLB/BAX/INO80/MYC/TOPBP1/NET1/HSF1/HSPA5/PML/STK11/RAD54B/MEN1/KDM1A/BARD1/PARP1/PAXIP1/KARS1/WRN/BRCA2/BRAT1/PRKDC/HRAS/CASP3/EYA3/RFWD3/ITGB6/MSH2/RAD51AP1/CYBA/BLM/TP53/RAD51/TICRR/DNM2/FANCD2/ECT2/BAK1/H2AX/MDM2/CCND1/AEN/RAD54L/CXCL10
GOCC_MRNA_CLEAVAGE_FACTOR_COMPLEX	GOCC_MRNA_CLEAVAGE_FACTOR_COMPLEX	0.015415054	PCF11/SCAF8/CSTF1/CLP1/WDR33/SYMPK/CPSF7/CPSF6/SSU72/CPSF2/CPSF3/CPSF4/FIP1L1/NUDT21/ZC3H3/CSTF2/CPSF1

GOMF_PROTEIN_DEMETHYLASE_ACTIVITY	GOMF_PROTEIN_DEMETHYLASE_ACTIVITY	0.015445527	RIOX1/KDM7A/JMJD6/KDM6B/PHF8/KDM3A/KDM8/RIOX2/KDM3B/RSBN1/KDM4B/KDM5C/KDM2B/KDM1A/KDM4C/KDM1B/FBXL19/KDM4A/HR
GOBP_NEURON_PROJECTION_EXTENSION	GOBP_NEURON_PROJECTION_EXTENSION	0.015610486	SYT4/SLIT2/SLIT3/MAP1B/TNN/PLXNA4/NDN/CXCL12/SEMA3E/NTN1/MAPT/NKX6-1/EDNRA/L1CAM/NRP2/PRKN/SH3GL2/FLRT1/FN1/NLGN3/SEMA6A/TMEM108/SEMA6D/ISLR2/NRCAM/WASF1/SEMA6C/SYT1/SEMA3G/DCLK1/MAP2/SEMA5B/SEMA3D/CXCR4
GOMF_LYSINE_ACETYLATED_HISTONE_BINDING	GOMF_LYSINE_ACETYLATED_HISTONE_BINDING	0.015631723	BRD9/DPF2/ZMYND8/TAF1/ATAD2B/BRD4/THAP7/TRIM24/YEATS4/PHIP/ZZZ3/BRD7/PSME4/BAZ2A/ZZEF1/BRD2/CARM1/SMARCA4

GOBP_POST_GOLGI_VESICLE_MEDIATED_TRANSPORT	GOBP_POST_GOLGI_VESICLE_MEDIATED_TRANSPORT	0.015651538	GGA2/LAMP1/STEAP2/VTI1B/SCAMP1/MON2/AP4M1/AP3S2/SORT1/BBS2/SORL1/CORO7/DOP1B/KIF13A/AP2A1/LLGL1/CCDC93/EXOC2/VPS52/GOLGA4/GBF1/ANKFY1/SCAMP3/GGA3/EXOC1/RAB10/VPS13A/KIF16B/VPS13C/DOP1A/RAB14/PKDCC/AP3D1/GOLGA7/GGA1/VTI1A/RACK1/VPS54/ATP2C1/CCDC22/ANK3/SCAMP2/GOLPH3L/VAMP3/SPTBN1/AP1G1/NSF/AP1AR/EXOC6/CSK/SEC16A/LYPLA1/AP1G2/ARFRP1/GAK/DNM2/AMN/KRT18
GOMF_ORGANOPHOSPHATE_ESTER_TRANSMEMBRANE_TRANSPORTER_ACTIVITY	GOMF_ORGANOPHOSPHATE_ESTER_TRANSMEMBRANE_TRANSPORTER_ACTIVITY	0.015719347	SLC35B3/SLC25A23/ABCD1/SLC25A17/LRRC8A/SLC37A4/SLC25A5/SLC35B1/SLC17A9/SLC25A33/SLC25A24/SLC25A19/SLC44A4/MFSD2A/SLC19A1/SLC37A1
GOBP_NEGATIVE_REGULATION_OF_CHONDROCYTE_DIFFERENTIATION	GOBP_NEGATIVE_REGULATION_OF_CHONDROCYTE_DIFFERENTIATION	0.015732528	EFEMP1/NKX3-2/GREM1/GLI3/WNT9A/SNAI2/CCN4/PTHLH/LTBP3

GOBP_REGULATION_OF_CELLULAR_EXTRAVASATION	GOBP_REGULATION_OF_CELLULAR_EXTRAVASATION	0.015723656	ELANE/JAM3/LYVE1/CXCL12/CCL21/IL1R1/SELP/PDGFD/BST1/THY1/RIPOR2/SELE/CD99L2
GOBP_POSITIVE_REGULATION_OF_TRANSCRIPTION_ELONGATION_BY_RNA_POLYMERASE_II	GOBP_POSITIVE_REGULATION_OF_TRANSCRIPTION_ELONGATION_BY_RNA_POLYMERASE_II	0.01573462	CDC73/ELL/BRD4/CDK13/MED30/CTNNB1/MED8/MED11/MED27/CDK12/SUPT16H/CCNT1/CCNK/TCERG1/LEO1/MED14/MED25/MED23/NCBP1/MED16/MED15/MED9/GTF2F1/MED26/MED1/MED18/PWWP2B/MED24/ELL3
GOBP_CELLULAR_RESPONSE_TO_INTERLEUKIN_1	GOBP_CELLULAR_RESPONSE_TO_INTERLEUKIN_1	0.015709562	CCL16/SFRP1/NLRP7/ANKRD1/PTGIS/FGG/CCL26/CCL21/AKAP12/CAMP/CCL19/IL1R1/MMP2/IL6/CCL2/CCL11/HAS2/FGB

GOMF_CORE_PROMOTER_SEQUENCE_SPECIFIC_DNA_BINDING	GOMF_CORE_PROMOTER_SEQUENCE_SPECIFIC_DNA_BINDING	0.015770483	TBPL1/TAF1/TBP/AGO1/TAF4/GTF2B/GTF2A1/REST/UBTF/DR1/ZBTB17/RELA/H3-3B/DRAP1/PARK7/MACROH2A1/H3-3A/TAF1C/BAZ2A/SMARCB1/PIH1D1/RRN3/MYC/RUVBL2/AGO2/MMP12/HDAC1/EZH2/STAT1/NPM1/SMARCA4/H2AZ1/TP53
GOBP_SYNAPTIC_VESICLE_LOCALIZATION	GOBP_SYNAPTIC_VESICLE_LOCALIZATION	0.015790876	SLC2A4/CDH2/NLGN1/LIN7A/PRKN/KIF5A/LRRK2/BRSK1/SYNDIG1/NLGN2/MAP2/PCDH17/AP3B2/DNM1/BRSK2/CNIH2/SNCA
GOBP_PROTEIN_ADP_RIBOSYLATION	GOBP_PROTEIN_ADP_RIBOSYLATION	0.015807683	BANF1/SIRT5/ADNP/SIRT1/ARF4/PNKP/SPINDOC/PUM3/PARP9/PARP4/PARP16/XRCC1/PARP12/PARP1/PARP14/SIRT6/PARP10/TNKS/PARP3/ART3/IFNG/WARS1

GOBP_DEVELOPMENT_OF_PRIMARY_SEXUAL_CHARACTERISTICS	GOBP_DEVELOPMENT_OF_PRIMARY_SEXUAL_CHARACTERISTICS	0.01581783	AFP/SFRP1/ADCYAP1R1/AMHR2/GFRA1/SLIT2/PGR/PTPRN/AR/SFRP2/SLIT3/CYP1B1/ZFPM2/TEX11/WT1/PTX3/ADAMTS1/ZFP42/TGFB2/MMP2/ESR1/MMP19/PDGFRA/OSR1/DHH/CGA/A2M/GDF9/RETN/SOX15/WNT2B/TCF21
GOBP_FORMATION_OF_CYTOPLASMIC_TRANSLATION_INITIATION_COMPLEX	GOBP_FORMATION_OF_CYTOPLASMIC_TRANSLATION_INITIATION_COMPLEX	0.015840287	EIF3M/EIF3K/EIF3J/EIF3B/EIF3D/EIF3H/EIF3E/EIF3I/EIF3A/EIF3G/EIF3CL/EIF3C
GOBP_MEIOSIS_II_CELL_CYCLE_PROCESS	GOBP_MEIOSIS_II_CELL_CYCLE_PROCESS	0.015865441	MASTL/STAG2/SMC3/PPP2R1A/STAG1/SGO2/RAD21/SMC1A/SGO1/BUB1/BUB1B/REC8

GOBP_REGULATORY_T_CELL_DIFFERENTIATION	GOBP_REGULATORY_T_CELL_DIFFERENTIATION	0.015859111	FOXP3/AMBRA1/MDK/SOX12/DUSP10/PLA2G2D/KAT5/BTN2A2/KLHL25/IL4I1/HLA-DRA/KAT2A/CD46/SOCS1/HLA-DRB1/LGALS9/FANCD2/CTLA4/LAG3/FANCA/IRF1/IFNG
GOBP_REGULATION_OF_MACROPHAGE_DIFFERENTIATION	GOBP_REGULATION_OF_MACROPHAGE_DIFFERENTIATION	0.015888386	ADIPOQ/MIR145/ROR2/INHBA/INHA/IL34
GOBP_POSITIVE_REGULATION_OF_PATHWAY_RESTRICTED_SMAD_PROTEIN_PHOSPHORYLATION	GOBP_POSITIVE_REGULATION_OF_PATHWAY_RESTRICTED_SMAD_PROTEIN_PHOSPHORYLATION	0.015956881	BMP3/GDF6/GDF10/TGFB3/LEFTY2/NODAL/TGFB2/RBPMS/DAB2/GDF9/GDF7/INHBA/INHA/ACVR1/BMP5/BMP6/ENG/INHBB/TGFBR1/TWSG1

GOBP_REGULATION_OF_EXTENT_OF_CELL_GROWTH	GOBP_REGULATION_OF_EXTENT_OF_CELL_GROWTH	0.015994181	EPHA7/MAP1B/NGF/PLXNA4/CXCL12/SEMA3E/NTN1/MAPT/RND2/LPAR3/NKX6-1/FGF13/L1CAM/FN1/SPART/CRABP2/SEMA6A/SEMA6D/ISLR2/NRCAM/SEMA6C/SEMA3G/MAP2/SEMA5B/SEMA3D/SEMA3C/NRP1
GOBP_ACTIVIN_RECEPTOR_SIGNALING_PATHWAY	GOBP_ACTIVIN_RECEPTOR_SIGNALING_PATHWAY	0.016146636	CER1/FGF10/AMHR2/GDF6/NODAL/DACT1/SHH/GDF7/IGSF1/INHBA/ACVR1
GOMF_O_METHYLTRANSFERASE_ACTIVITY	GOMF_O_METHYLTRANSFERASE_ACTIVITY	0.016175651	FTSJ1/FTSJ3/MEPCE/ICMT/ARMT1/MRM1/CMTR2/MRM3/COMTD1/TRMT44/COQ3
GOMF_CYCLIC_NUCLEOTIDE_BINDING	GOMF_CYCLIC_NUCLEOTIDE_BINDING	0.016176419	POPDC2/BVES/PRKG1/PRKAR2B/PDE2A/POPDC3/CNGA1/PDE5A

GOMF_ELECTRON_TRANSFER_ACTIVITY	GOMF_ELECTRON_TRANSFER_ACTIVITY	0.016367168	NDUFB8/NDUFC2/ETFB/DHRS3/NDUFB5/NDUFC1/NDUFA1/FDX1/COX7B/NDUFB4/COX6B1/NDUFS8/UQCR11/NDUFB2/ETFDH/ETFA/NDUFA8/P4HA2/COX7A2L/CYB5A/NDUFA6/NDUFB10/ALDH2/NDUFS5/NDUFB3/NDUFA12/NDUFA5/NDUFA9/NDUFB6/NDUFS6/UQCRFS1/NDUFA10/NCF1/NDUFS2/QDPR/NDUFA2/NDUFS1/NDUFS3/SDHD/SRD5A1/NDUFB7/IDH3B/COX5B/NQO2/UQCR10/ME2/COX4I1/COX6A1/CYCS/CIAPIN1/AKR7A2/SDHB/NDOR1/RDH16/COX15/COX5A/UQCRFS1P1/UQCRH/COX8A/SDHA/ASPH/NDUFV1/GPX2/CYBA/NDUFS7/AIFM2/UQCRC1/CYC1/ALDH4A1/GSR/GFUS/AKR7A3/IDO1
GOBP_CELLULAR_GLUCOSE_HOMEOSTASIS	GOBP_CELLULAR_GLUCOSE_HOMEOSTASIS	0.016380924	GCG/CARTPT/NEUROD1/KCNB1/NGFR/CMA1/ADCY5/PTPRN/GRIK5/PRKAA2/MIR320C2/NPTX1/RFX6/SERPINF1/KLF15/ZBTB20/NR1H4/MIR320D1/GPR27/KLF7/NOX4
GOBP_REGULATION_OF_ANION_TRANSMEMBRANE_TRANSPORT	GOBP_REGULATION_OF_ANION_TRANSMEMBRANE_TRANSPORT	0.016458779	ATP1A2/RGS4/AGT/RGS2/ABCB1/PRNP

GOBP_FORMATION_OF_PRIMARY_GERM_LAYER	GOBP_FORMATION_OF_PRIMARY_GERM_LAYER	0.016468248	HAND1/VTN/MIR145/SIX2/SFRP2/NOG/ITGA7/MMP8/COL8A1/ITGA8/FOXF1/ATOH8/CRB2/NODAL/ITGA5/MMP2/FN1/DKK1/FZD7/GJA1/FOXC2/DUSP1/SOX17/INHBA/ITGB3/WLS/LEF1/ACVR1/COL5A1/COL7A1/COL12A1/COL5A2/FGFR2/COL4A2
GOBP_TYPE_B_PANCREATIC_CELL_DIFFERENTIATION	GOBP_TYPE_B_PANCREATIC_CELL_DIFFERENTIATION	0.016485389	NKX2-2/INSM1/CDH2/RFX6/NKX6-1/ONECUT1/SMO

GOCC_ENDOPEPTIDASE_COMPLEX	GOCC_ENDOPEPTIDASE_COMPLEX	0.016511652	PSMD10/PSMA1/UBE3A/USP14/PSMA7/PSME3/HTRA2/PSMB5/GPAA1/PSMD5/PSMD6/PSMB4/TXNL1/PSMC5/PSMF1/PSMB1/CASP9/PSMA3/PIGU/PSMD13/CRADD/PSMB6/RAD23B/PSMD7/PSMC3/PSMC2/PSMC4/PSMD2/PSME4/PSMD9/PIGS/PSMB3/UBQLN1/CAPNS1/PSMD8/UCHL5/PSMC1/PSMB7/PSMA6/PLAUR/PSMD12/PSMB2/PSMD14/ECPAS/CAPN2/PSMA4/UBE3C/ADRM1/RAD23A/PIDD1/PSMA5/VCP/CASP2/PSME1/PSMB9/PSME2/PSMB8/PSMD3/PSMB10
GOBP_ENDOSOME_TRANSPORT_VIA_MULTIVESICULAR_BODY_SORTING_PATHWAY	GOBP_ENDOSOME_TRANSPORT_VIA_MULTIVESICULAR_BODY_SORTING_PATHWAY	0.016584433	STAM2/SNF8/STAM/CHMP2A/TMEM50B/SORT1/VPS25/TMEM50A/VPS36/VPS37A/VPS37D/VPS4A/UBXN6/UBAP1/MVB12A/VPS37C/CHMP6/VTA1/VPS28/CHMP2B/LEPROTL1/RILP/CHMP7/CHMP1A/VPS4B/HGS/CHMP5/PTPN23/VCP/VPS37B/CHMP4C

GOMF_PROTON_TRANSMEMBRANE_TRANSPORTER_ACTIVITY	GOMF_PROTON_TRANSMEMBRANE_TRANSPORTER_ACTIVITY	0.016583949	UQCRFS1/ATP5F1EP2/SLC15A3/CLCN5/SLC9B2/TMCO3/COX5B/SLC11A2/ATP5PO/UQCR10/SLC2A13/SLC25A3/COX4I1/ATP6V0B/ATP5MC1/ATP5PB/COX6A1/SLC16A3/SLC17A5/SLC9A4/COX15/COX5A/ATP5MC3/ATP5F1A/UQCRFS1P1/UQCRH/COX8A/ATP5F1B/CLCN3/ATP5ME/CLCN4/TCIRG1/SLC9A7/CHP1/ATP5F1D/SLC45A4/UQCRC1/SLC22A18/CYC1/SLC9A2/MFSD3/SLC45A3/SLC25A22/SLC9A3/LETM1/SLC9A1/ATP4B/ATP12A/SLC4A11/ATP4A
GOBP_HISTONE_H2A_MONOUBIQUITINATION	GOBP_HISTONE_H2A_MONOUBIQUITINATION	0.016626229	PCGF2/PCGF3/PCGF1/TRIM37/PCGF6/RNF168/DDB1/KDM2B/BRCC3/BRCA1/PCGF5/BCOR/BARD1/BRCA2/DDB2/RAD51
GOMF_PROTON_TRANSPORTING_ATP_SYNTHASE_ACTIVITY_ROTATIONAL_MECHANISM	GOMF_PROTON_TRANSPORTING_ATP_SYNTHASE_ACTIVITY_ROTATIONAL_MECHANISM	0.016612968	ATP5MG/ATP5MF/ATP6V1A/ATP5F1C/ATP5PD/ATP5F1EP2/ATP5PO/ATP5PB/ATP5F1A/ATP5F1B/ATP5ME/ATP5F1D

GOBP_RESPONSE_TO_ESTROGEN	GOBP_RESPONSE_TO_ESTROGEN	0.016652702	APOA2/SFRP1/AR/GLI3/F7/TGFB3/CAV1/TNFRSF11B/MMP2/ESR1/DHH
GOBP_NEUTRAL_LIPID_METABOLIC_PROCESS	GOBP_NEUTRAL_LIPID_METABOLIC_PROCESS	0.016650347	APOA2/PNLIPRP2/APOA1/APOC3/APOH/APOB/RGN/THRSP/TNXB/GPIHBP1/DGKG/CAV1
GOBP_APPENDAGE_DEVELOPMENT	GOBP_APPENDAGE_DEVELOPMENT	0.016634866	COL2A1/HAND2/FGF10/MEOX2/ALDH1A2/SFRP2/NOG/FERMT2/GPC3/CACNA1C/ZBTB16/GREM1/HOXD10/GLI3/RECK/RSPO2/AFF3/SALL4/MAP3K20/TGFB2/COMP/COL3A1/SHH/OSR1/PBX1/PRRX1/DKK1/CRABP2/PTCH1/TWIST1/TBX5/DYNC2H1/RSPO4/HOXD9/LEF1/SEMA3C/FGFR2/CIBAR1/SP8/SOX11/PITX2

GOMF_SNRNA_BINDING	GOMF_SNRNA_BINDING	0.016687372	GEMIN5/CCNT1/PRPF31/LSM3/LSM2/SNRPA1/MEPCE/SNRNP70/LSM7/SART3/EFTUD2/TUT1/PRPF8/HNRNPU/DDX21/PRPF4/SF3B3/SNRPA/TOE1/LSM4/ISG20/EIF5A
GOCC_ACTIN_CYTOSKELETON	GOCC_ACTIN_CYTOSKELETON	0.016688546	ACTG2/MYH11/SYNPO2/LDB3/ACTN2/PGM5/HSPB7/LMOD1/MYLK/NPFFR2/MYL9/SORBS1/FILIP1/CRYAB/TPM2/PDLIM3/ACTA2/TMOD1/DMD/NEXN/CFL2/MYL3/CALD1/FERMT2/DPYSL3/CDH2/EPHA3/PKNOX2/FLNA/DDR2/SVIL/SMTN/CNR1/JAM3/SMTNL2/ACTA1/NTN1/ADD2/AMPH/TPM1/CDC42EP3/ACTC1/CGNL1/LPP/PALLD/LMOD3/PDLIM4/PRKCB/MYH10/CORIN/FAM107A/CLIC4/CTTNBP2/DLC1/PARVA/SORBS2/SYNPO/GAS7/PDLIM7/ILK/USH1G/PLEKHH2/CLDN5/TEK/ACTN1/MTSS1/WASF1/CORO2B/MYRIP/DSTN/SH3PXD2B/RHOQ/ROR1/ARC/MYADM/SHROOM4/GSN/MYOZ1/AXL/DENND2A/RDX

GOBP_REGULATION_OF_FIBROBLAST_GROWTH_FACTOR_RECEPTOR_SIGNALING_PATHWAY	GOBP_REGULATION_OF_FIBROBLAST_GROWTH_FACTOR_RECEPTOR_SIGNALING_PATHWAY	0.016717477	NGFR/NOG/SMOC2/FGF2/THBS1/HHIP/MIR149/SULF1/SHISA2/FAM20C/FUZ/SPRY1
GOCC_HISTONE_DEACETYLASE_COMPLEX	GOCC_HISTONE_DEACETYLASE_COMPLEX	0.016730961	CHD3/TBL1XR1/OGT/NCOR1/ING1/SAP30/MBD2/SAP18/KDM3A/ING3/CSNK2A1/RBBP7/HDAC6/SATB2/TRRAP/SAP130/TRERF1/EP400/KDM3B/BRD8/HINT1/MTA1/HDAC3/ZNHIT1/KAT5/HDAC2/HDAC10/TAF6L/ACTR6/ING2/SUDS3/MBD3/RUVBL1/RUVBL2/SRCAP/ANP32E/RBBP4/SINHCAF/BRMS1/GATAD2A/HDAC1/RCOR1/MTA2/RCOR2/HR

GOCC_ENDONUCLEASE_COMPLEX	GOCC_ENDONUCLEASE_COMPLEX	0.016859274	RPP14/TRMT10C/AGO1/RPP38/PRORP/CLP1/ERN1/POP5/TSN/HSD17B10/LAS1L/TSEN34/TSEN2/POP4/DHX9/DGCR8/TARBP2/EME1/MUS81/POP7/RPP25/AGO2/TSEN54/EME2/RPP25L/POP1
GOBP_RESPIRATORY_CHAIN_COMPLEX_IV_ASSEMBLY	GOBP_RESPIRATORY_CHAIN_COMPLEX_IV_ASSEMBLY	0.016937858	SURF1/FASTKD3/COA1/COX16/COA8/COX19/C12orf73/SLC25A46/TMEM223/COA6/SMIM20/PET100/COA4/BCS1L/TACO1/SCO1/TIMM21/COX15/COX10/COX18/SCO2
GOBP_MATING_BEHAVIOR	GOBP_MATING_BEHAVIOR	0.016924316	VIP/TAC1/TACR1/CNR1/P2RX1/SERPINE2/AVPR1A/OXTR/THRB/P2RY1/OXT/EDNRB
GOBP_REGULATION_OF_SUBSTRATE_ADHESION_DEPENDENT_CELL_SPREADING	GOBP_REGULATION_OF_SUBSTRATE_ADHESION_DEPENDENT_CELL_SPREADING	0.016944938	APOA1/MYOC/FGA/FGG/FERMT2/LIMS2/FBLN1/FLNA/DAB2/ILK/HAS2/PDPN/FGB/ITGB3/MYADM/NRP1

GOCC_PEPTIDASE_COMPLEX	GOCC_PEPTIDASE_COMPLEX	0.016965967	PSMA1/UBE3A/USP14/PSMA7/PSME3/HTRA2/SEC11A/PSMB5/TAF9/GPAA1/PSMD5/SPCS1/TAF12/PSMD6/PSMB4/TXNL1/PSMC5/PSMF1/TADA3/SPCS3/PSMB1/CASP9/TRRAP/SPCS2/PSMA3/TAF5L/USP22/TADA1/PIGU/TADA2B/PSMD13/CRADD/PSMB6/RAD23B/PSMD7/PSMC3/PSMC2/PSMC4/PSMD2/PSME4/PSMD9/PIGS/PSMB3/UBQLN1/CAPNS1/PSMD8/UCHL5/PSMC1/TAF6L/PSMB7/ATXN7L3/PSMA6/PLAUR/AFG3L2/PSMD12/PSMB2/PSMD14/ECPAS/CAPN2/PSMA4/UBE3C/F3/ADRM1/KAT2A/RAD23A/PIDD1/SF3B5/SF3B3/PSMA5/VCP/CASP2/PSME1/PSMB9/PSME2/PSMB8/PSMD3/PSMB10
GOBP_ENDOPLASMIC_RETICULUM_TO_CYTOSOL_TRANSPORT	GOBP_ENDOPLASMIC_RETICULUM_TO_CYTOSOL_TRANSPORT	0.017037761	RHBDD1/ERLEC1/EDEM2/HERPUD1/SEC61B/DERL2/NPLOC4/UFD1/DERL1/FAF2/UBE2J1/UBAC2/OS9/HM13/SELENOS/AUP1/SYVN1/YOD1/TMEM129/DERL3/BCAP31/HSP90B1/VCP/SVIP/EDEM1

GOBP_NEGATIVE_REGULATION_OF_ENDOPLASMIC_RETICULUM_UNFOLDED_PROTEIN_RESPONSE	GOBP_NEGATIVE_REGULATION_OF_ENDOPLASMIC_RETICULUM_UNFOLDED_PROTEIN_RESPONSE	0.017058309	ATF6B/PPP1R15B/BFAR/RACK1/UFL1/HSPA5/DDRGK1/ABCA7
GOBP_MATURATION_OF_LSU_RRNA_FROM_TRICISTRONIC_RRNA_TRANSCRIPT_SSU_RRNA_5_8S_RRNA_LSU_RRNA	GOBP_MATURATION_OF_LSU_RRNA_FROM_TRICISTRONIC_RRNA_TRANSCRIPT_SSU_RRNA_5_8S_RRNA_LSU_RRNA	0.017058309	GTPBP4/NOL9/NIFK/DDX18/ZNHIT3/RPF2/WDR12/FTSJ3/RPL35/RPL7/RPL7L1/PES1/URB1/BOP1
GOBP_NEGATIVE_REGULATION_OF_DNA_REPAIR	GOBP_NEGATIVE_REGULATION_OF_DNA_REPAIR	0.017043527	MAGEF1/ERCC6/OGG1/UBR5/RECQL5/RNF169/SHLD2/SHLD3/TP53BP1/MAD2L2/RIF1/CYREN/TERF2/RPS3/TFIP11/NUDT16L1/OTUB1/SMCHD1/HSF1/FANCB/UBQLN4/HELB/PARPBP/CGAS/OTUB2/POLQ/AUNIP/RMI2
GOBP_REGULATION_OF_CELL_COMMUNICATION_BY_ELECTRICAL_COUP	GOBP_REGULATION_OF_CELL_COMMUNICATION_BY_ELECTRICAL_COUPLING	0.017020559	CASQ2/HRC/CAV1/SLC8A1/IRX3/TBX5
GOBP_LEUKOCYTE_ADHESION_TO_VASCULAR_ENDOTHELIAL_CELL	GOBP_LEUKOCYTE_ADHESION_TO_VASCULAR_ENDOTHELIAL_CELL	0.017054132	ELANE/JAM2/CXCL12/ADD2/CCL21/CX3CR1/SELP/IL6/SELL/MIR222/SELE

GOBP_MORPHOGENESIS_OF_AN_EPITHELIAL_FOLD	GOBP_MORPHOGENESIS_OF_AN_EPITHELIAL_FOLD	0.017072599	FGF10/AR/NOG/NODAL/SHH/GDF7/WNT2B/SULF1/BMP5/FGFR2/HOXD13/TP63
GOCC_PROTEASOME_REGULATORY_PARTICLE	GOCC_PROTEASOME_REGULATORY_PARTICLE	0.017100284	PSMD1/PSMC6/PSMD4/PSMD6/PSMC5/PSMD13/PSMD7/PSMC3/PSMC2/PSMC4/PSMD2/PSMD9/PSMD8/PSMC1/PSMD12/PSMD14/ADRM1/PSMD3
GOMF_CARBON_CARBON_LYASE_ACTIVITY	GOMF_CARBON_CARBON_LYASE_ACTIVITY	0.017101545	CLYBL/AMD1/HACL1/HMGCL/ME2/ARMT1/PCK2/AZIN1/MVD/ODC1/PDXDC1/SGPL1/PCK1/GOT1/PAICS/SHMT2/ALDOA/GAD1/CYP17A1
GOBP_POSITIVE_REGULATION_OF_FOCAL_ADHESION_ASSEMBLY	GOBP_POSITIVE_REGULATION_OF_FOCAL_ADHESION_ASSEMBLY	0.017179312	SFRP1/MYOC/FERMT2/COL16A1/TEK/THY1/NRP1
GOBP_STEROID_CATABOLIC_PROCESS	GOBP_STEROID_CATABOLIC_PROCESS	0.017223187	CYP24A1/HSD17B6/HSD11B1/HSD17B14/CYP39A1/CYP27A1/CYP3A4
GOCC_STRIATED_MUSCLE_THIN_FILAMENT	GOCC_STRIATED_MUSCLE_THIN_FILAMENT	0.017223187	LMOD1/TPM2/TMOD1/ACTA1/TPM1/LMOD3

GOBP_KIDNEY_MORPHOGENESIS	GOBP_KIDNEY_MORPHOGENESIS	0.017244768	FGF10/TMEM59L/SIX2/NOG/ADAMTS16/GPC3/FGF2/GREM1/WT1/GLI3/GREB1L/AGT/WWTR1/PKD2/SHH/SMO/OSR1/PBX1/WNT2B/TCF21/LRRK2/DCHS1/FGF1/ILK/PTCH1/IRX3
GOBP_U2_TYPE_PRESPLICEOSOME_ASSEMBLY	GOBP_U2_TYPE_PRESPLICEOSOME_ASSEMBLY	0.017307281	SF3B4/SNIP1/SF3B6/SNRPE/SNRPD2/SNRPD3/DDX46/SNRPG/HTATSF1/SF3B1/SF3A1/SNRPF/SNRPA1/PHF5A/SF3A3/SNRPB/BUD13/SF3B2/SNRPD1/SF3A2/SF3B5/SF3B3
GOBP_REGULATION_OF_CD8_POSITIVE_ALPHA_BETA_T_CELL_ACTIVATION	GOBP_REGULATION_OF_CD8_POSITIVE_ALPHA_BETA_T_CELL_ACTIVATION	0.017387677	PTPN22/HLA-E/HLA-A/CBFB/SOCS1/SH3RF1/ZBTB7B/IRF1/CD274
GOBP_ADHESION_OF_SYMBIONT_TO_HOST	GOBP_ADHESION_OF_SYMBIONT_TO_HOST	0.017385649	NECTIN2/HSPD1/NECTIN1/ICAM1/SFTPD/BPIFA1
GOBP_NEGATIVE_REGULATION_OF_CALCIUM_ION_TRANSMEMBRANE_TRANSPORT	GOBP_NEGATIVE_REGULATION_OF_CALCIUM_ION_TRANSMEMBRANE_TRANSPORT	0.017423758	CASQ2/ATP1A2/PLN/GEM/PKD2/REM1/SLN/RRAD/CLIC2

GOBP_TRANSFORMING_GROWTH_FACTOR_BETA_RECEPTOR_SIGNALING_PATHWAY	GOBP_TRANSFORMING_GROWTH_FACTOR_BETA_RECEPTOR_SIGNALING_PATHWAY	0.017496635	APOA1/MYOCD/CIDEA/AMHR2/FERMT2/ASPN/TGFB1I1/GDF10/ZEB1/ITGA8/TGFB3/THBS1/CAV1/LTBP1/NODAL/TGFB2/FBN1/ONECUT1/SMAD9/COL3A1/MIR342/EMILIN1/DAB2/GDF9/CDKN1C/ZEB2/DKK3/LOX/LRRC32/FMOD/TGFBR3/CLDN5/LTBP3/HSPA1A/COL1A2/LDLRAD4/LTBP2/TET1/VASN/ACVR1/NREP/HTRA1/SPRED3/CD109/SOX11/LTBP4/ENG/CAV2/CDH5/ADAMTSL2/SPRY1/HTRA3/USP9Y/TGFBR1/TWSG1
GOBP_REGULATION_OF_URINE_VOLUME	GOBP_REGULATION_OF_URINE_VOLUME	0.017520636	NPPB/NPR3/NPR1/AVPR2/HAS2/OXT/EDNRB
GOBP_POSITIVE_REGULATION_OF_CHOLESTEROL_EFFLUX	GOBP_POSITIVE_REGULATION_OF_CHOLESTEROL_EFFLUX	0.017571978	APOA1/ADIPOQ/ABCA8/CES1/CAV1/ABCA1/PTCH1/PLTP/LRP1
GOBP_DNA_DOUBLE_STRAND_BREAK_PROCESSING	GOBP_DNA_DOUBLE_STRAND_BREAK_PROCESSING	0.017636988	RAD50/UBE2N/RNF138/SMARCAD1/SLX4/HELB/RBBP8/NBN/BLM/DNA2/BRIP1

GOCC_TRANSLATION_PREINITIATION_COMPLEX	GOCC_TRANSLATION_PREINITIATION_COMPLEX	0.017636988	EIF3M/EIF3K/EIF3J/EIF1/EIF3B/EIF3D/EIF3H/EIF3E/EIF3I/EIF3A/EIF3G/EIF2S1/EIF3CL/EIF3C
GOBP_CELLULAR_RESPONSE_TO_PROSTAGLANDIN_ST	GOBP_CELLULAR_RESPONSE_TO_PROSTAGLANDIN_STIMULUS	0.017618622	SFRP1/APOB/PTGFR/PRKAA2
GOBP_BRANCHED_CHAIN_AMINO_ACID_CATABOLIC_PROCESS	GOBP_BRANCHED_CHAIN_AMINO_ACID_CATABOLIC_PROCESS	0.01767507	BCKDK/ACAT1/DBT/BCKDHB/AUH/HIBADH/SLC25A44/DLD/HSD17B10/MCCC1/ACADSB/HMGCL/ACAD8/IVD/HIBCH/BCAT2/MCCC2/ECHS1
GOBP_ACTIN_FILAMENT_BASED_TRANSPORT	GOBP_ACTIN_FILAMENT_BASED_TRANSPORT	0.01767507	WASL/MYO6/MYO1G/FNBP1L/MYO1E/MYO19/MYO1A/SYNE2/MYO5B/MYO5C
GOMF_NADP_BINDING	GOMF_NADP_BINDING	0.01766128	MTHFR/NDOR1/GRHPR/DUS2/DECR1/HSD17B1/DHCR7/DHFRP1/IDH1/PGD/DUOX1/SPR/GAPDH/DHFR/HMGCR/GSR/NOS2/GMDS

GOBP_PROTEIN_DEMETHYLATION	GOBP_PROTEIN_DEMETHYLATION	0.017723938	RIOX1/KDM7A/JMJD6/KDM6B/HSF4/PHF8/KDM3A/KDM8/RIOX2/PPME1/KDM3B/KDM4B/ALKBH4/KDM5C/KDM2B/KDM1A/KDM4C/KDM1B/FBXL19/KDM4A/HR
GOBP_RESPONSE_TO_AXON_INJURY	GOBP_RESPONSE_TO_AXON_INJURY	0.017737627	KCNB1/PCSK1/RGMA/APOD/GAP43/KCNK2/DPYSL3/MAP1B/FLNA/PTN/FGF2/TNC/GIP/MMP2/APOA4/ISL1/NEFL/THY1/MIR222
GOBP_MUCUS_SECRETION	GOBP_MUCUS_SECRETION	0.017774031	NLRP6/ATG5/VAMP8/SCN1B/VAMP3/TRAF3IP2/CYBA/ANO1/ADA/ALOX12B/AGR2
GOBP_POSITIVE_REGULATION_OF_BIOMINERALIZATION	GOBP_POSITIVE_REGULATION_OF_BIOMINERALIZATION	0.017775994	PTN/GPM6B/TGFB3/CCN1/SLC8A1/TMEM119/OSR1/ADRB2/NELL1/WNT10B/BMPR1B/KL/MEF2C/ACVR1/FBLN7/AMTN/FAM20C/BMP6

GOBP_LIPID_HOMEOSTASIS	GOBP_LIPID_HOMEOSTASIS	0.017753773	APOA2/APOA1/APOC3/FABP4/CES1/APOB/PRKAA2/SOAT2/GPIHBP1/GIP/PLA2G12B/CAV1/CYP7B1/FABP3/ZBTB20/NR1H4/APOA4/MIR27B/MALRD1/CYP39A1/ACOX2/ABCA1/LPL/MTTP/PNPLA1
GOCC_TRANSCRIPTION_FACTOR_TFIID_COMPLEX	GOCC_TRANSCRIPTION_FACTOR_TFIID_COMPLEX	0.017815459	TAF1/GTF2E1/TBP/TAF13/TAF4/GTF2B/GTF2A1/ERCC3/TAF9/TAF12/TAF2/TAF5L/ERCC2/GTF2H2/TCEA1/TAF6L/TAF4B/GTF2H3/GTF2F1/TAF5/GTF2E2/GTF2A2
GOBP_CELL_FATE_DETERMINATION	GOBP_CELL_FATE_DETERMINATION	0.017859352	NKX2-2/ASCL1/PTCH2/TNXB/EBF2/HOXA2/PRRX1/ISL1/ATOH1/SOX17/MEF2C/PTCH1
GOBP_REGULATION_OF_PROTEIN_LOCALIZATION_TO_CELL_SURFACE	GOBP_REGULATION_OF_PROTEIN_LOCALIZATION_TO_CELL_SURFACE	0.017859352	ACTN2/TMEM35A/RIC3/MAP1A/GPM6B

GOBP_REGULATION_OF_ANION_TRANSPORT	GOBP_REGULATION_OF_ANION_TRANSPORT	0.017898512	SYT4/ATP1A2/CES1/SLC38A3/RGS4/SFRP4/AGT/SV2A/RGS2/ABCB1/AVPR1A/PRNP/STC1/GABBR1/GABRE/OXT/CLTRN/PLA2R1/PRKG2/SNCA/PDZK1/ACE2/NTSR1/TNFSF11
GOBP_CELLULAR_MODIFIED_AMINO_ACID_METABOLIC_PROCESS	GOBP_CELLULAR_MODIFIED_AMINO_ACID_METABOLIC_PROCESS	0.017924199	DUOX2/FOLR1/SOD1/MTHFD2L/ALDH1L1/GART/GSTZ1/ATIC/CKM/ASS1/SLC6A8/DIO3/CKMT1B/MTHFR/GCH1/GSTK1/MED1/PTDSS1/GGT6/CPT2/FPGS/CKMT1A/GOT2/MMACHC/MBOAT2/SERINC2/DHFRP1/CHDH/IDH1/GSTM4/DUOX1/MFSD2A/SHMT1/MTHFD2/MTHFD1L/DUOXA2/MBOAT1/VNN1/ABHD12B/GSTO2/DHFR/ALDH4A1/CHAC2/MTHFD1/SLC19A1/ETHE1/SHMT2/GSR/TYMS/LPCAT4/PLA2G2F/PRODH/OPLAH/SLC7A11/CHAC1/PLA2G3

GOMF_CATION_TRANSMEMBRANE_TRANSPORTER_ACTIVITY	GOMF_CATION_TRANSMEMBRANE_TRANSPORTER_ACTIVITY	0.017927147	KCNA1/SLC18A3/ATP1A2/SCN7A/GRIK3/CHRNA3/KCNB1/KCNMA1/KCNK3/GRIK1/GPM6A/KCNMB1/GRIN2A/KCNA5/SLC13A5/SLC8A2/ABCC9/RYR3/GRIK5/KCNK2/KCNH6/SLC38A3/KCNT2/KCNIP1/CACNA1C/P2RX2/TRPC4/SLC18A2/SLC7A2/CACNG4/TMEM63C/CNR1/TRPC1/CACNA1H/SCN4B/ATP6V1G2/NIPAL4/KCNJ8/CACNB2/KCNE4/SLC17A7/KCNH2/COX7A1/CHRNB4/SLC39A2/NCS1/P2RX1/ATP2B4/TRPM6/COX7B2/TUSC3/SLC7A10/SCN3A/SLC30A2/SLC45A1/SLC1A7/SLC8A1/PKD2/CACNA2D3/CACNA2D1/CACNA1A/KCNG1/SNAP25/RHCG/PIEZO2/SLC9A9/SLC10A4/KCNQ3/SLC17A4/KCND1/SLC24A3/RHBG/SLC29A4/CNGA1/CALHM5/SLC5A9
GOBP_REGULATION_OF_RHO_PROTEIN_SIGNAL_TRANSDUCTION	GOBP_REGULATION_OF_RHO_PROTEIN_SIGNAL_TRANSDUCTION	0.018075476	APOA1/APOC3/MYOC/ARHGEF25/LPAR4/GPR17/KANK2/LPAR1/COL3A1/DLC1/ABCA1/HEG1/RIPOR2/RASGRF2/ROBO1/STARD13/PDGFRB/RASIP1/NRP1/GPR20/CAVIN4/F2RL2/KCTD10/MET/F2R/F2RL3/GPR65/STARD8/GPR174

GOBP_REGULATION_OF_ALPHA_BETA_T_CELL_ACTIVATION	GOBP_REGULATION_OF_ALPHA_BETA_T_CELL_ACTIVATION	0.018105192	PTPN22/SYK/PNP/HLA-E/IL23A/AP3B1/RIPK2/KLHL25/HLA-DRA/IL12RB1/HLA-A/CBFB/TYK2/LGALS9B/SOCS1/SH3RF1/ZBTB7B/IL18/PRKCQ/TNFRSF14/ZC3H12A/HLA-DRB1/LGALS9/ADA/PRKCZ/CD55/LGALS9C/ZNF683/IRF1/JAK2/IFNG/MYB/CD274
GOBP_IMP_METABOLIC_PROCESS	GOBP_IMP_METABOLIC_PROCESS	0.018152009	HPRT1/NT5C2/ADSS2/AMPD2/APRT/NT5C/GART/PNP/ATIC/ADSS1/PFAS/XDH/PPAT/PAICS
GOBP_REGULATION_OF_EXTRINSIC_APOPTOTIC_SIGNALING_PATHWAY	GOBP_REGULATION_OF_EXTRINSIC_APOPTOTIC_SIGNALING_PATHWAY	0.018189757	COL2A1/SFRP1/FGA/FGF10/IGF1/FGG/SRPX/AR/SFRP2/AGT/THBS1/CAV1/HGF/FGFR1/MAL/SERPINE1/SNAI2/BMPR1B/SCG2/FGB/HSPA1A/MIR222/TNFSF12/TIMP3/INHBA

GOBP_NUCLEAR_TRANSCRIBED_MRNA_CATABOLIC_PROCESS_EXONUCLEOLYTIC	GOBP_NUCLEAR_TRANSCRIBED_MRNA_CATABOLIC_PROCESS_EXONUCLEOLYTIC	0.018249856	EXOSC6/POLR2G/EXOSC3/DCP2/SKIV2L/DIS3L2/CNOT8/EXOSC8/DIS3/CNOT7/EXOSC2/EXOSC7/EXOSC5/CNOT6/EXOSC9/EXOSC4/DCPS
GOBP_PHOSPHATIDYLCHOLINE_METABOLIC_PROCESS	GOBP_PHOSPHATIDYLCHOLINE_METABOLIC_PROCESS	0.018239426	PLA2G1B/APOA2/PNLIPRP2/APOA1/PLA2G5/PLA2G2C/PLA2G4C/FABP3/APOA4/PLAAT1
GOCC_IMMUNOGLOBULIN_COMPLEX_CIRCULATING	GOCC_IMMUNOGLOBULIN_COMPLEX_CIRCULATING	0.01824331	IGHV4-61/JCHAIN/IGHV3-30/TRDC/IGLL5/IGHA1/IGHD/IGHV3-72/IGHV3-64D/IGHV3-33/IGHV3-48/IGHV3-66/PIGR/IGHA2/IGHV1-58/IGHM/IGHV3-49/IGHV3-13/IGLL1

GOBP_ALPHA_AMINO_ACID_METABOLIC_PROCESS	GOBP_ALPHA_AMINO_ACID_METABOLIC_PROCESS	0.018238489	QDPR/ACADSB/OTC/ASL/ADI1/DLST/SMS/PLOD3/MRI1/ICMT/HMGCL/ENOPH1/ACAD8/MTHFD2L/AGMAT/BAAT/GART/MPST/GSTZ1/KYAT3/ASS1/PYCR3/IL4I1/AZIN1/ASPG/ODC1/MTHFR/PFAS/MTAP/CAD/THAP4/IVD/PCBD1/CTPS1/HIBCH/FPGS/ILVBL/GOT2/BCAT2/DHFRP1/GFPT1/SHMT1/ALDH18A1/MSRA/MCCC2/TST/PPAT/GCAT/DHFR/DDAH1/ALDH4A1/PYCR1/MTHFD1/GOT1/SHMT2/PSAT1/NOS2/BCAT1/GPT2/PRODH/SLC7A11/GAD1/CTH/GPT/CPS1/IDO1
GOBP_LEUKOTRIENE_BIOSYNTHETIC_PROCESS	GOBP_LEUKOTRIENE_BIOSYNTHETIC_PROCESS	0.018321477	PLA2G1B/PLA2G5/GGT5/GGTA1
GOBP_REGULATION_OF_AMINO_ACID_TRANSMEMBRAN	GOBP_REGULATION_OF_AMINO_ACID_TRANSMEMBRANE_TRANSPORT	0.018569764	ATP1A2/RGS4/AGT/RGS2
GOBP_PYRIMIDINE_CONTAINING_COMPOUND_BIOSYNTHETIC_PROCESS	GOBP_PYRIMIDINE_CONTAINING_COMPOUND_BIOSYNTHETIC_PROCESS	0.018609205	MTOR/UCK2/TK2/DCTD/CAD/CTPS1/DTYMK/CDA/DCK/DUT/CMPK1/TPK1/SHMT1/TYMS/CPS1

GOBP_NEGATIVE_REGULATION_OF_NERVOUS_SYSTEM_DEVELOPMENT	GOBP_NEGATIVE_REGULATION_OF_NERVOUS_SYSTEM_DEVELOPMENT	0.018691805	SYT4/SOX10/EPHA7/F2/DLL3/NOG/PTN/PRTG/DAB1/DAAM2/SEMA3E/NTN1/NFATC4/NKX6-1/FGF13/IL6/DKK1/SPART/EFNB3/DPYSL5/ID4/ATOH1/SEMA6A/CCL11/SEMA6D/THY1/SEMA6C/SEMA3G/MAP2/SEMA5B/SEMA3D/ELAPOR2/SEMA3C/NRP1
GOBP_PURINE_DEOXYRIBONUCLEOTIDE_METABOLIC_PROCESS	GOBP_PURINE_DEOXYRIBONUCLEOTIDE_METABOLIC_PROCESS	0.018728328	NUDT16/NT5C2/GDA/DGUOK/NUDT15/GUK1/ADK/NT5C/PNP/XDH/NUDT18/DCK/ADA
GOBP_RETINOIC_ACID_METABOLIC_PROCESS	GOBP_RETINOIC_ACID_METABOLIC_PROCESS	0.018725402	ADH1B/ALDH1A2/ADH1A/ADH7/UGT1A7/CRABP2/ALDH1A3/CYP3A4/CYP1A1/RBP1/CYP2W1

GOBP_RESPONSE_TO_PURINE_CONTAINING_COMPOUND	GOBP_RESPONSE_TO_PURINE_CONTAINING_COMPOUND	0.018712034	AQP8/CASQ2/PENK/ADIPOQ/CDO1/AKAP6/ABCC9/RYR3/CYP1B1/P2RX2/WT1/PDE3A/P2RX1/PDE2A/FOSB/SLC8A1/PKD2/MMP19/IGFBP5/WNT10B/THBD/SLC26A3/RYR2/STC1
GOBP_NEGATIVE_REGULATION_OF_PRODUCTION_OF_MOLECULAR_MEDIATOR_OF_IMMUNE_RESPONSE	GOBP_NEGATIVE_REGULATION_OF_PRODUCTION_OF_MOLECULAR_MEDIATOR_OF_IMMUNE_RESPONSE	0.018885124	APOA2/APOA1/IL33/TGFB3/ANGPT1/TGFB2/FCGR2B/TWIST1/IL13RA2/AXL/CR1
GOBP_NEGATIVE_REGULATION_OF_COLD_INDUCED_THERMOGENESIS	GOBP_NEGATIVE_REGULATION_OF_COLD_INDUCED_THERMOGENESIS	0.018974144	ADIPOQ/CIDEA/NPR3/NOVA1/ZNF423/PLCL1/LAMA4/WNT10B/FOXC2
GOBP_REGULATION_OF_CD4_POSITIVE_ALPHA_BETA_T_CELL_ACTIVATION	GOBP_REGULATION_OF_CD4_POSITIVE_ALPHA_BETA_T_CELL_ACTIVATION	0.018980617	IL23A/RIPK2/KLHL25/HLA-DRA/IL12RB1/CBFB/LGALS9B/SOCS1/SH3RF1/ZBTB7B/IL18/PRKCQ/ZC3H12A/HLA-DRB1/LGALS9/PRKCZ/CD55/LGALS9C/IFNG/MYB/CD274

GOBP_EXOCRINE_SYSTEM_DEVELOPMENT	GOBP_EXOCRINE_SYSTEM_DEVELOPMENT	0.019065807	SOX10/FGF10/FGF7/IGF2/TGFB3/LAMA1/HGF/TGFB2/SHH/SNAI2/EDA/WLS/SEMA3C/NRP1/FGFR2
GOCC_KINESIN_COMPLEX	GOCC_KINESIN_COMPLEX	0.019167309	KIF20B/KIF23/KIF14/KIF9/KIFC1/KLC2/KIF18A/KIF18B/KIF22/KIFC2/KIF13B/KIF2C/KIF15/KIF11/KIF26A
GOCC_CYTOPLASMIC_EXOSOME_RNASE_COMPLEX	GOCC_CYTOPLASMIC_EXOSOME_RNASE_COMPLEX	0.019205476	EXOSC8/DIS3L/DIS3/GTPBP1/EXOSC10/EXOSC2/EXOSC7/EXOSC5/SUPV3L1/EXOSC9/EXOSC4
GOBP_ADAPTIVE_THERMOGENESIS	GOBP_ADAPTIVE_THERMOGENESIS	0.01935317	ADIPOQ/FABP4/CIDEA/ADCYAP1/NPR3/SCT/NOVA1/EBF2/LEPR/CD36/CAV1/ZNF423/PLCL1/ESRRG/SCTR/ADRB2/FFAR4/LAMA4/DIO2/OXTR/WNT10B/SLN/GJA1/FOXC2/PDGFC/GADD45G/MFAP2/CXCR4/ADAMTS5

GOBP_REGULATION_OF_PLATELET_DERIVED_GROWTH_FACTOR_RECEPTOR_SIGNALING_P	GOBP_REGULATION_OF_PLATELET_DERIVED_GROWTH_FACTOR_RECEPTOR_SIGNALING_PATHWAY	0.019441028	ADIPOQ/MYOCD/APOD/F7/PTGIR/LOX/LRP1/NDRG4
GOBP_NEUTROPHIL_CHEMOTAXIS	GOBP_NEUTROPHIL_CHEMOTAXIS	0.019468219	PLA2G1B/CCL16/THBS4/SLIT2/CCL26/JAM3/CXCL6/CCL21/ITGA9/CCL19/S100A8/TGFB2/ITGA1/S100A9/CCL2/CCL11/BST1/CXCR2/CD300H/CAMK1D/CXCR1/S100A12/RIPOR2/MPP1/CCR7
GOBP_RESPONSE_TO_ANGIOTENSIN	GOBP_RESPONSE_TO_ANGIOTENSIN	0.019543264	AGTR1/ACTN2/MIR145/DDR2/AGT/CAV1/COL3A1/KL/INHBA/SLC30A10
GOBP_RESPONSE_TO_STEROL_DEPLETION	GOBP_RESPONSE_TO_STEROL_DEPLETION	0.019581185	SPRING1/EIF2A/PIP4P1/INSIG2/NPC1L1/ARHGEF10L/AMFR/SCAP/TMED2/ERLIN2/LPCAT3/INSIG1/SREBF2/ZBTB7B/ERLIN1/LYN/SREBF1

GOBP_REGULATION_OF_ADENYLATE_CYCLASE_ACTIVITY	GOBP_REGULATION_OF_ADENYLATE_CYCLASE_ACTIVITY	0.019634935	ADCYAP1/NPFFR2/NPR3/CAP2/CACNA1C/GABBR2/VIPR2/EDNRA/HPCA/PALM/ADRB2/GLP1R/AVPR2/GABBR1/ADORA3
GOBP_NUCLEOSIDE_METABOLIC_PROCESS	GOBP_NUCLEOSIDE_METABOLIC_PROCESS	0.019701826	APOBEC3F/APOBEC3B/UCKL1/HPRT1/NT5C2/GDA/APOBEC3D/DGUOK/DPYD/NUDT1/AHCY/DNPH1/ERH/APOBEC3G/APOBEC3H/APOBEC1/APRT/ICMT/ADK/TK2/PNP/ATIC/NT5C3A/OARD1/MTAP/PGM2/XDH/DTYMK/CDA/ACP3/MACROD2/MACROD1/TYMP/ADA/TK1

GOCC_CONDENSED_NUCLEAR_CHROMOSOME	GOCC_CONDENSED_NUCLEAR_CHROMOSOME	0.019743921	BRCA1/PSMC3IP/CHMP1A/TOPBP1/NEK2/NCAPH2/BRCA2/RAD21/SMC4/LRPPRC/SMC1A/SUV39H1/RRS1/DMC1/SMC2/INCENP/NCAPG2/CHEK1/NOL6/BLM/RAD51/NCAPH/H2AX/PLK1/RSPH1/RCC1/NCAPG/SYCE2/REC8
GOCC_H4_HISTONE_ACETYLTRANSFERASE_COMPLEX	GOCC_H4_HISTONE_ACETYLTRANSFERASE_COMPLEX	0.019737024	MSL3/ATF2/VPS72/KAT8/MORF4L2/EP400P1/KANSL1/DMAP1/MSL2/OGT/YEATS2/KANSL3/ING3/KANSL2/YEATS4/TRRAP/MLLT1/EP400/BRD8/MRGBP/ACTL6A/KAT5/RUVBL1/RUVBL2/MCRS1/WDR5/HCFC1/MSL1/ACTBL2
GOBP_PITUITARY_GLAND_DEVELOPMENT	GOBP_PITUITARY_GLAND_DEVELOPMENT	0.019932434	GHRH/FGF10/PCSK1/ALDH1A2/NOG/FGF2/GLI1/ISL1

GOBP_REGULATION_OF_ENDOCRINE_PROCESS	GOBP_REGULATION_OF_ENDOCRINE_PROCESS	0.019930247	AGTR1/TACR2/AGT/DAB2/GDF9/RETN/GJA1/INHBA/INHA/BMP6/GHRL/F2R/INHBB/C1QTNF1
GOBP_EXIT_FROM_MITOSIS	GOBP_EXIT_FROM_MITOSIS	0.020015148	RPL24/SPAST/PPP1R9B/PPP2R2D/MAD2L1BP/EPS8/CDC23/CHMP7/CTDP1/ZW10/KNTC1/SIRT7/CDC14B/PHB2/UBE2S/ANLN/CDCA5
GOBP_MEMBRANE_REPOLARIZATION_DURING_ACTION_POTENTIAL	GOBP_MEMBRANE_REPOLARIZATION_DURING_ACTION_POTENTIAL	0.020033135	KCNA5/KCNH6/FLNA/KCNJ8/KCNE4/KCNH2/CAV1/CACNA2D1
GOCC_PRESYNAPTIC_ACTIVE_ZONE_MEMBRANE	GOCC_PRESYNAPTIC_ACTIVE_ZONE_MEMBRANE	0.020033135	GPM6A/CDH2/CNTNAP1/P2RX1/ATP2B4/SYT11/APBA1/P2RY1/STX1B/NTNG2/STX2
GOBP_REGULATION_OF_DNA_DAMAGE_CHECKPOINT	GOBP_REGULATION_OF_DNA_DAMAGE_CHECKPOINT	0.020088484	RPA2/FBXO4/PPP1R10/CUL4A/CHEK2/BRCC3/BRCA1/BARD1/BRCA2/CCAR2/RFWD3/TELO2/TTI2/RAD51/WDR76/PBK

GOBP_POSITIVE_REGULATION_OF_LEUKOCYTE_DEGRANULATION	GOBP_POSITIVE_REGULATION_OF_LEUKOCYTE_DEGRANULATION	0.02009781	VAMP8/SPHK2/PTAFR/GAB2/SYK/AP1G1/KLRC2/F2RL1/CD177/HLA-F/ADORA2B/PLA2G3
GOMF_UBIQUITIN_PROTEIN_TRANSFERASE_REGULATOR_ACTIVITY	GOMF_UBIQUITIN_PROTEIN_TRANSFERASE_REGULATOR_ACTIVITY	0.02009781	RPL5/BTRC/RPS20/RPL11/UBE2L3/TRIB1/GLMN/RPS7/AUP1/RPS15/FBXO5/FZR1/CBX8/CDC20/TRIB2
GOBP_SPINAL_CORD_MOTOR_NEURON_DIFFERENTIATION	GOBP_SPINAL_CORD_MOTOR_NEURON_DIFFERENTIATION	0.020086133	NKX2-2/HOXD10/GLI3/SHH/ISL1/PTCH1/DYNC2H1
GOBP_CHONDROCYTE_PROLIFERATION	GOBP_CHONDROCYTE_PROLIFERATION	0.020086133	SIX2/DDR2/COMP/MMP16/CCN3/CCN2/BMPR1B/STC1/LEF1/PRKG2/PBXIP1
GOBP_POSITIVE_REGULATION_OF_MITOTIC_SISTER_CHROMATID_SEPARATION	GOBP_POSITIVE_REGULATION_OF_MITOTIC_SISTER_CHROMATID_SEPARATION	0.02013667	ANAPC11/ANAPC7/CDC27/CUL3/MAD2L1BP/CDC23/RB1/ANAPC5/AURKB/INCENP/DLGAP5/ESPL1/BIRC5/CDCA8/CDT1

GOBP_RRNA_TRANSCRIPTION	GOBP_RRNA_TRANSCRIPTION	0.020152364	ERCC2/GTF3C1/GTF3C3/MACROH2A1/GTF3C4/TCOF1/POLR1E/GTF3C2/SMARCB1/PIH1D1/GTF3C6/NCL/MTOR/NOL11/POLR1B/IPPK/MARS1/BRF1/DDX11/GTF3C5/SIRT7/SMARCA4/NPM3/TP53
GOBP_NEGATIVE_REGULATION_OF_MONONUCLEAR_CELL_MIGRATION	GOBP_NEGATIVE_REGULATION_OF_MONONUCLEAR_CELL_MIGRATION	0.020192974	APOD/SLIT2/GREM1/CCL2/CCN3/CD200/DUSP1/RIPOR2/BMP5
GOBP_NEGATIVE_REGULATION_OF_CYTOKINE_PRODUCTION_INVOLVED	GOBP_NEGATIVE_REGULATION_OF_CYTOKINE_PRODUCTION_INVOLVED_IN_IMMUNE_RESPO	0.020337767	APOA2/APOA1/TGFB3/ANGPT1/TGFB2
GOBP_NEGATIVE_REGULATION_OF_EPITHELIAL_CELL_APOPTOTIC_PROCESS	GOBP_NEGATIVE_REGULATION_OF_EPITHELIAL_CELL_APOPTOTIC_PROCESS	0.020346387	HAND2/NEUROD1/FGA/NKX2-5/FGG/ANGPT1/SERPINE1/ATOH1/IL11/RAMP2/SCG2/FGB/TEK

GOBP_POSITIVE_REGULATION_OF_DNA_REPLICATION	GOBP_POSITIVE_REGULATION_OF_DNA_REPLICATION	0.020350909	DBF4B/LPIN1/SMARCA5/MAP2K4/SSBP1/WIZ/CIZ1/CDK2/ATF1/FAF1/KCTD13/ATAD5/UCN/STN1/DHX9/INO80/EGFR/DBF4/STOML2/CDK1/PCNA/DNA2/ENDOG/CDC7/E2F8/E2F7/CDT1
GOBP_MORPHOGENESIS_OF_A_POLARIZED_EPITHELIUM	GOBP_MORPHOGENESIS_OF_A_POLARIZED_EPITHELIUM	0.020321612	SFRP1/SFRP2/PRICKLE2/GPC3/ROR2/RSPO3/PRICKLE1/FOXF1/LAMA1/DACT1/DAB2/ANKRD6/DKK1/GPC6/FOXF2/FZD7/BRSK1/TCF15/CTHRC1/ROR1/VANGL2/JHY/FZD1/FUZ/PLEKHA4/NPHP1/BRSK2
GOBP_ETHANOL_METABOLIC_PROCESS	GOBP_ETHANOL_METABOLIC_PROCESS	0.020437573	ADH1B/SULT2A1/SULT1C4/ADH1A/ADH7/ALDH1B1/ADH4
GOMF_HORMONE_RECEPTOR_BINDING	GOMF_HORMONE_RECEPTOR_BINDING	0.020429818	UCN3/GHRH/VIP/NPPB/FABP4/ADCYAP1/GNAO1

GOBP_MULTICELLULAR_ORGANISMAL_RESPONSE_TO_STRESS	GOBP_MULTICELLULAR_ORGANISMAL_RESPONSE_TO_STRESS	0.020430437	TAC1/PENK/RELN/ATP1A2/TACR1/ADCYAP1R1/THBS4/GRPR/GRP/P2RX2/THBS1
GOBP_RESPONSE_TO_VITAMIN_D	GOBP_RESPONSE_TO_VITAMIN_D	0.020493215	PENK/SFRP1/CYP24A1/MN1/KANK2/SNAI2/STC1/KL/STC2
GOCC_CORE_MEDIATOR_COMPLEX	GOCC_CORE_MEDIATOR_COMPLEX	0.02071137	MED20/MED6/MED4/MED31/MED28/MED22/MED10/MED30/MED8/MED11/MED27/MED14/MED25/MED23/MED16/MED15/MED9/MED26/MED1/MED18/MED24
GOCC_MICROVILLUS_MEMBRANE	GOCC_MICROVILLUS_MEMBRANE	0.020676791	SLC7A5/AMN/SYTL1/PTPRH/CDHR2/EZR/PROM2/S100P/CEACAM20/CA9
GOBP_CELL_DIFFERENTIATION_IN_SPINAL_CORD	GOBP_CELL_DIFFERENTIATION_IN_SPINAL_CORD	0.020691742	NKX2-2/ASCL1/HOXD10/GLI3/SHH/ISL1/GDF7/PTCH1/DYNC2H1/GLI2
GOBP_WHITE_FAT_CELL_DIFFERENTIATION	GOBP_WHITE_FAT_CELL_DIFFERENTIATION	0.020706495	FABP4/FGF10/PDGFRA/FFAR4/SNAI2
GOBP_MESENCHYMAL_TO_EPITHELIAL_TRANSITION	GOBP_MESENCHYMAL_TO_EPITHELIAL_TRANSITION	0.020761373	SIX2/GREM1/WT1/SMO/FZD7/TCF15

GOBP_BIOLOGICAL_PROCESS_INVOLVED_IN_INTRASPECIES_INTERACTION_BETWEEN_ORGANISMS	GOBP_BIOLOGICAL_PROCESS_INVOLVED_IN_INTRASPECIES_INTERACTION_BETWEEN_ORGANISMS	0.020806161	NRXN3/NRXN2/GRPR/PTCHD1/GRP/NLGN4X/NLGN4Y/CX3CR1/AVPR1A/OXTR/NLGN3/DLG4/NLGN2/CHRNB2/OXT/GRID1
GOBP_DETECTION_OF_MECHANICAL_STIMULUS_INVOLVED_IN_SENSORY_PERCEPTION	GOBP_DETECTION_OF_MECHANICAL_STIMULUS_INVOLVED_IN_SENSORY_PERCEPTION	0.020826501	KCNA1/HTR2A/CXCL12/SERPINE2/PIEZO2/CXCR4/KIT/BACE1/FYN/PJVK/PDZD7
GOMF_CHOLESTEROL_BINDING	GOMF_CHOLESTEROL_BINDING	0.020856356	APOA2/APOA1/APOC3/APOD/SOAT2/CAV1/APOA4/ABCA1/PTCH1/ANXA6/SYP
GOBP_MAINTENANCE_OF_PROTEIN_LOCALIZATION_IN_ORGANELLE	GOBP_MAINTENANCE_OF_PROTEIN_LOCALIZATION_IN_ORGANELLE	0.020850081	TOPORS/CIZ1/ANKRD13C/PARK7/KDELR1/KDELR2/OS9/INSIG1/TXN/TAPT1/ARL2BP/HNRNPU/HSPA5/PML/AKT1/BARD1/KDELR3/RER1/CDK5/RANGAP1/CHCHD10/TSPO/HK2/PDIA2
GOBP_POSITIVE_REGULATION_OF_FILOPODIUM_ASSEMBLY	GOBP_POSITIVE_REGULATION_OF_FILOPODIUM_ASSEMBLY	0.020876255	GPM6A/DPYSL3/NLGN1/CCL21/TGFB3/TENM1/PALM/NEURL1/DOCK11/RIPOR2/CCR7/RHOQ/NRP1

GOBP_POSITIVE_REGULATION_OF_TELOMERE_CAPPING	GOBP_POSITIVE_REGULATION_OF_TELOMERE_CAPPING	0.021024942	PNKP/MAP3K4/NABP2/RTEL1/MAP2K7/MAPK3/NEK2/HNRNPD/AURKB/PRKCQ/TNKS/MAPK15
GOMF_UBIQUITIN_BINDING	GOMF_UBIQUITIN_BINDING	0.021014552	UBE2A/CUEDC2/ILRUN/RNFT1/BIRC2/IKBKG/UBXN10/TOLLIP/N4BP1/UBXN1/MARK4/HDAC6/SHARPIN/VPS36/NPLOC4/UCHL3/DNAJB2/UBR5/AMFR/N4BP2/UBE2N/NSFL1C/UBAP1/RNF168/SMAD3/FBXO7/GGA3/FAF1/UBXN2B/FAF2/UBXN8/MVB12A/USP33/RAD23B/DDI2/PLAA/USP5/ASCC2/VPS28/RBCK1/OTUB1/SMARCAD1/NUP62/GGA1/ZFAND2B/AUP1/HGS/CKS1B/TOM1L1/ADRM1/RAD23A/UBXN11/BUB3/CKS2/UBE2L6/OTUB2/RNF19B/MDM2/TOP2A
GOBP_GLOMERULAR_MESANGIUM_DEVELOPMENT	GOBP_GLOMERULAR_MESANGIUM_DEVELOPMENT	0.021178684	ACTA2/WT1/PDGFD/FOXC2/ITGB3/CD34/PDGFRB
GOMF_COPPER_ION_BINDING	GOMF_COPPER_ION_BINDING	0.021219067	ALB/AOC3/PRND/APOA4/SOD3/F8/MOXD1/SNCG/LOX/PRNP/S100A12

GOBP_REGULATION_OF_TRANSLATIONAL_FIDELITY	GOBP_REGULATION_OF_TRANSLATIONAL_FIDELITY	0.021328838	AARS2/GATC/LARS1/EIF2S3/DNAJC2/RPS9/AARS1/RPS5/DTD2/IARS1/TARS2/YRDC/EIF2S3B/IARS2/VARS1/LARS2
GOBP_TRNA_WOBBLE_BASE_MODIFICATION	GOBP_TRNA_WOBBLE_BASE_MODIFICATION	0.021328838	NSUN3/ALKBH8/ELP1/MTO1/ELP5/URM1/ELP6/CTU1/TRMU/ELP3/CTU2/ADAT2/GTPBP3
GOBP_MRNA_MODIFICATION	GOBP_MRNA_MODIFICATION	0.021343167	WTAP/METTL14/METTL16/PCIF1/CBLL1/PUS3/TRMT61A/TRMT10C/METTL3/PUS7L/TRMT6/VIRMA/DNAJB11/TRMT61B/TRUB2/APOBEC1/NSUN2/DKC1/TRUB1/PUS1/CMTR2/PUS7/RPUSD2/RPUSD4/HNRNPAB
GOBP_REGULATION_OF_RIG_I_SIGNALING_PATHWAY	GOBP_REGULATION_OF_RIG_I_SIGNALING_PATHWAY	0.021411188	NOP53/USP15/BIRC2/NLRX1/PUM2/PUM1/NPLOC4/UFD1/GPATCH3/DHX58/ANKRD17/DDX60/OASL/BIRC3/C1QBP/TRIM15

GOBP_POSITIVE_REGULATION_OF_MEIOTIC_CELL_CYCLE	GOBP_POSITIVE_REGULATION_OF_MEIOTIC_CELL_CYCLE	0.021411188	CDC25B/OVOL1/YTHDC2/MSX1/RAD51AP1/CDC25C/LFNG/MSX2/MAPK15/CDC25A
GOCC_RNA_POLYMERASE_III_COMPLEX	GOCC_RNA_POLYMERASE_III_COMPLEX	0.021525385	CRCP/POLR3D/POLR2H/POLR1C/POLR2L/POLR3E/POLR3H/POLR2E/POLR3B/POLR3A/POLR3K/POLR3G
GOBP_ADRENERGIC_RECEPTOR_SIGNALING_PATHWAY	GOBP_ADRENERGIC_RECEPTOR_SIGNALING_PATHWAY	0.021506378	PLN/ADRA1D/CHGA/ADRA1B/ATP2B4/RGS2/ADRB2
GOBP_ENDOCRINE_PANCREAS_DEVELOPMENT	GOBP_ENDOCRINE_PANCREAS_DEVELOPMENT	0.021529835	NKX2-2/NEUROD1/INSM1/CDH2/GIP/RFX6/NKX6-1/ONECUT1/IL6/SMO

GOBP_NEGATIVE_REGULATION_OF_CYTOSKELETON_ORGANIZATION	GOBP_NEGATIVE_REGULATION_OF_CYTOSKELETON_ORGANIZATION	0.021546488	STMN2/MYOC/LMOD1/SLIT2/TMOD1/TUBB4A/MAP1B/CLIP3/SVIL/NAV3/PHLDB2/ADD2/KANK4/CGNL1/FGF13/LMOD3/HDGFL3/PRKN/IQCJ-SCHIP1/FHOD3/DLC1/BMERB1/ARHGAP28/MIR149/S1PR1/PLEKHH2/TMSB15A/CORO2B/MAP2/MYADM/GSN/RDX/PFN2
GOBP_NEGATIVE_REGULATION_OF_CELL_MIGRATION_INVOLVED_IN_SPROUTING_ANGIOGENESIS	GOBP_NEGATIVE_REGULATION_OF_CELL_MIGRATION_INVOLVED_IN_SPROUTING_ANGIOGENESIS	0.021677064	MIR483/MEOX2/THBS1/MIR149/MMRN2/MIR196A1/STARD13/TBXA2R/HDAC5
GOBP_REGULATION_OF_RRNA_PROCESSING	GOBP_REGULATION_OF_RRNA_PROCESSING	0.021764185	USP36/NUDT16/DIMT1/RIOK1/TRMT112/RIOK2/BUD23/SHQ1/HEATR1/YTHDF2/WDR75/WDR43/UTP15/SIRT7

GOCC_FLEMMING_BODY	GOCC_FLEMMING_BODY	0.021799253	EXOC2/VPS4A/CHAMP1/EXOC1/RALA/BIRC6/USP3/NUP62/CNTRL/PDCD6IP/VPS4B/RAN/ZFYVE19/ARF6/KIF23/EXOC6/KIF14/RACGAP1/CHMP4C/AGAP2/CEP55
GOBP_FOLIC_ACID_METABOLIC_PROCESS	GOBP_FOLIC_ACID_METABOLIC_PROCESS	0.021829795	FOLR1/MTHFD2L/FPGS/DHFRP1/SHMT1/MTHFD2/DHFR/SLC19A1/SHMT2
GOBP_RESPONSE_TO_AMPHETAMINE	GOBP_RESPONSE_TO_AMPHETAMINE	0.021865164	GRIN2A/RGS4/SLC18A2/EDNRA/CNR2/RGS2/PDE1B/OXTR
GOBP_NUCLEOLAR_LARGE_RRNA_TRANSCRIPTION_BY_RNA_POLYMERASE_I	GOBP_NUCLEOLAR_LARGE_RRNA_TRANSCRIPTION_BY_RNA_POLYMERASE_I	0.021884251	MACROH2A1/TCOF1/POLR1E/SMARCB1/PIH1D1/NCL/MTOR/NOL11/IPPK/MARS1/DDX11/SIRT7/SMARCA4

GOBP_CENTRAL_NERVOUS_SYSTEM_NEURON_DEVELOPMENT	GOBP_CENTRAL_NERVOUS_SYSTEM_NEURON_DEVELOPMENT	0.021896837	ASCL1/SLIT2/NPY/DCLK2/PLXNA4/SEMA3E/MAPT/NRP2/CDH11/LHX6/DCLK1/CHRNB2/MAP2/ADARB1/GLI2/NDNF/NRP1/SCN1B/FGFR2/NOVA2/DISC1/NTRK2
GOMF_ENDORIBONUCLEASE_ACTIVITY_PRODUCING_5_PHOSPHOMONOESTERS	GOMF_ENDORIBONUCLEASE_ACTIVITY_PRODUCING_5_PHOSPHOMONOESTERS	0.022125105	DROSHA/ELAC1/DICER1/RPP40/RNASEH1/APEX1/RPP14/ENDOV/RPP38/PRORP/NUDT16/POP5/DBR1/PLD6/NUDT16L1/POP4/POP7/RPP25/AGO2/NUDT12/ELAC2/MRPL44/POP1/EXO1/FEN1
GOCC_INHIBITORY_SYNAPSE	GOCC_INHIBITORY_SYNAPSE	0.022140937	NLGN4X/NLGN1/NLGN4Y/CEP112/SYT11/NLGN3/IQSEC3/NLGN2/IGSF21

GOMF_CYTOSKELETAL_MOTOR_ACTIVITY	GOMF_CYTOSKELETAL_MOTOR_ACTIVITY	0.02232037	KIF20B/KIF23/MYO1E/KIF14/KIF9/KIFC1/KIF18A/MYO19/KIF18B/KIF4A/KIF24/KIF22/KIFC2/KIF13B/MYO1A/KIF2C/CENPE/MYH14/MYO5B/KIF15/KIF11/MYO5C/DNAH6/KIF26A/MYO3B
GOBP_POSITIVE_REGULATION_OF_FAT_CELL_DIFFERENTIATION	GOBP_POSITIVE_REGULATION_OF_FAT_CELL_DIFFERENTIATION	0.022437182	SFRP1/TPH1/HTR2A/LMO3/SFRP2/FNDC5/MEDAG/ZBTB16/FRZB/FFAR4/SNAI2/DKKL1/LPL/RARRES2
GOBP_VASCULAR_TRANSPORT	GOBP_VASCULAR_TRANSPORT	0.022568966	ATP1A2/SLC2A4/SLC8A2/ABCC9/SLC38A3/ATP1B2/SLC7A2/KCNJ8/LEPR/CD36/ATP2B4/ABCB1/AVPR1A/SLC16A7/SLC24A3/SLC29A4/ABCC2/SLC2A3/SLC16A2

GOBP_REGULATION_OF_LYASE_ACTIVITY	GOBP_REGULATION_OF_LYASE_ACTIVITY	0.022615348	ADCYAP1/NPFFR2/NPR3/CAP2/CACNA1C/GABBR2/VIPR2/NCS1/EDNRA/HPCA/GUCA2A/PALM/ADRB2/GLP1R/AVPR2/GABBR1/ADORA3
GOBP_SODIUM_ION_IMPORT_ACROSS_PLASMA_MEMBRANE	GOBP_SODIUM_ION_IMPORT_ACROSS_PLASMA_MEMBRANE	0.022636357	SCNN1B/HCN2/SLC9A4/TRPM4/SLC12A2/SLC5A1/SLC9A7/SLC9A2/SLC5A2/SLC9A3/SLC9A1/SCNN1G
GOCC_SARCOPLASMIC_RETICULUM_MEMBRANE	GOCC_SARCOPLASMIC_RETICULUM_MEMBRANE	0.022711586	CASQ2/PLN/CASQ1/JPH2/AKAP6/KLHL41/RYR3/DMPK/SLN/RYR2/JPH3/SRL

GOBP_ATP_BIOSYNTHETIC_PROCESS	GOBP_ATP_BIOSYNTHETIC_PROCESS	0.022706304	ATP5MC2/NDUFB8/DMAC2L/NDUFC2/NDUFB5/NDUFC1/NDUFA1/NDUFB4/ATP5MG/NDUFS8/NDUFB2/ATP5MF/DGUOK/NDUFA8/ATP6V1A/ATP5F1C/NDUFA6/NDUFB10/ATP5PD/NDUFS5/NDUFB3/NDUFA12/NDUFA5/NDUFA9/NDUFB6/NDUFA11/NDUFS6/NDUFA10/NDUFS2/ATP5F1EP2/SPHK2/NDUFA2/NDUFS1/NDUFS3/SDHD/NDUFB7/ATP5PO/NDUFAB1/ATP5MC1/ATP5PB/NDUFA13/PARP1/NUDT2/UQCC3/SDHB/ATP5MC3/ATP5F1A/VCP/ANTKMT/SDHA/ATP5F1B/STOML2/ATP5ME/NDUFV1/NDUFS7/ATP5F1D/ENO1/ALDOA
GOBP_CYTOLYSIS	GOBP_CYTOLYSIS	0.022777472	GZMA/MICB/NKG7/LYZ/GZMH/C8G/PRF1/GSDMB/GZMB/PLA2G2A/C6
GOBP_CLATHRIN_COAT_ASSEMBLY	GOBP_CLATHRIN_COAT_ASSEMBLY	0.022878968	NSG2/CALY/DAB2/SGIP1
GOBP_POSITIVE_REGULATION_OF_EPITHELIAL_CELL_APOPTOTIC_PROCESS	GOBP_POSITIVE_REGULATION_OF_EPITHELIAL_CELL_APOPTOTIC_PROCESS	0.022854246	PLA2G1B/SFRP4/THBS1/IL6/CCL2/H19/CD248/GPER1/ECSCR/GSN

GOMF_STEROL_BINDING	GOMF_STEROL_BINDING	0.022867116	APOA2/APOA1/APOC3/APOD/SOAT2/CAV1/APOA4/GPR183/ABCA1/PTCH1/ANXA6/SYP
GOBP_NEGATIVE_REGULATION_OF_EPITHELIAL_TO_MESENCHYMAL_TRANSITION	GOBP_NEGATIVE_REGULATION_OF_EPITHELIAL_TO_MESENCHYMAL_TRANSITION	0.023144765	SFRP1/DACT3/SFRP2/NOG/MARK1/TGFB2/ZNF750/MIR149/LDLRAD4/TBX5/VASN/SPRED3/BMP5/FUZ
GOMF_ANTIPORTER_ACTIVITY	GOMF_ANTIPORTER_ACTIVITY	0.023185915	CLCN5/SLC9B2/SLC24A1/TMCO3/SLC25A11/SLC37A4/SLC25A5/SLC35B1/SLC26A1/SLC8B1/SLC4A2/SLC35D2/SLC25A15/SLC3A2/SLC9A4/SLC4A8/SLC26A6/CLCN3/SLC35E4/SLC35C1/CLCN4/SLC9A7/CHP1/SLC25A10/SLC22A18/SLC9A2/SLC7A5/SLC9A3/SLC4A4/SLC19A1/LETM1/SLC35D3/SLC37A1/SLC9A1/SLC4A11/SLC7A11/SLC26A9

GOBP_REGULATION_OF_PEPTIDYL_SERINE_PHOSPHORYLATION	GOBP_REGULATION_OF_PEPTIDYL_SERINE_PHOSPHORYLATION	0.023218899	GCG/DMD/SFRP2/NGF/TRIM6/HRC/PRKD1/ATP2B4/CSF3/CAV1/HGF/ANGPT1/TENM1/IL6/DKK1/NTF3
GOMF_GDP_DISSOCIATION_INHIBITOR_ACTIVITY	GOMF_GDP_DISSOCIATION_INHIBITOR_ACTIVITY	0.023309553	GDI2/CHML/GPSM2/SESN2/ARHGDIA/RANBP1/RGS14/ARHGDIG
GOBP_CELLULAR_RESPONSE_TO_STEROL_DEPLETION	GOBP_CELLULAR_RESPONSE_TO_STEROL_DEPLETION	0.023309553	SPRING1/EIF2A/INSIG2/NPC1L1/ARHGEF10L/AMFR/SCAP/TMED2/ERLIN2/LPCAT3/INSIG1/SREBF2/ZBTB7B/ERLIN1/SREBF1
GOBP_DNA_LIGATION	GOBP_DNA_LIGATION	0.023309553	HMGB1/MGMT/XRCC6/PNKP/TFIP11/NHEJ1/XRCC1/APTX/PARP3/RAD51/LIG1/TOP2A
GOBP_SOMITOGENESIS	GOBP_SOMITOGENESIS	0.023367143	SFRP1/MEOX2/ALDH1A2/DLL3/SFRP2/FOXF1/CRB2/TBX18/DKK1/MEOX1/FOXC2/TCF15

GOBP_ENDOCARDIAL_CUSHION_FORMATION	GOBP_ENDOCARDIAL_CUSHION_FORMATION	0.023557538	TMEM100/NOG/TGFB3/TGFB2/HEYL/SNAI2/DCHS1/ROBO1/ACVR1/APLNR/BMP5/ENG/SNAI1
GOBP_REGULATION_OF_MEMBRANE_LIPID_DISTRIBUTION	GOBP_REGULATION_OF_MEMBRANE_LIPID_DISTRIBUTION	0.023586046	CLPTM1L/RFT1/ATG9A/TMEM41B/ATP9B/PRKCD/TRIAP1/XKR9/SLC66A2/ATP11A/GBA2/ATP8A1/ATG9B/ABCA2/PRELID1/ATP8B1/MFSD2A/TLCD2/TMEM30B/ABCB4/KCNN4/ABCA7/ATP10B/FASLG/ABCA12
GOBP_MYOTUBE_CELL_DEVELOPMENT	GOBP_MYOTUBE_CELL_DEVELOPMENT	0.023609742	IGF1/KLHL41/DNER/P2RX2/ACTA1/CNTNAP1/LMOD3/SMO/WNT10B
GOBP_MITOCHONDRIAL_CYTOCHROME_C_OXIDASE_ASSEMBLY	GOBP_MITOCHONDRIAL_CYTOCHROME_C_OXIDASE_ASSEMBLY	0.023726533	COX17/SMIM4/SURF1/FASTKD3/COA1/COX16/COA8/COX19/C12orf73/TMEM223/SMIM20/PET100/COA4/BCS1L/TACO1/SCO1/TIMM21/COX18/SCO2

GOMF_LIPOPOLYSACCHARIDE_BINDING	GOMF_LIPOPOLYSACCHARIDE_BINDING	0.023728983	RNASE3/BPI/F2/RNASE2/CAMP/SELP/LY96/ADGRB1/TRIL
GOBP_SEROTONIN_TRANSPORT	GOBP_SEROTONIN_TRANSPORT	0.023750522	SLC18A2/CNR1/GPM6B/P2RX1/SLC29A4/SLC18A1/ITGB3/SLC6A4/SNCA/SLC22A3
GOBP_MESODERM_DEVELOPMENT	GOBP_MESODERM_DEVELOPMENT	0.023747529	HAND1/CER1/MIR145/DLL3/SIX2/SFRP2/NOG/RPS6KA6/ZFPM2/ITGA8/FOXF1/BMX/CRB2/NODAL/SHH/SMO/OSR1/DKK1/GJA1/FOXC2/TCF15/INHBA/IRX3/ITGB3/WLS/LEF1/ACVR1

GOMF_UBIQUITIN_LIKE_PROTEIN_BINDING	GOMF_UBIQUITIN_LIKE_PROTEIN_BINDING	0.023746267	UBE2A/CUEDC2/CASP8AP2/ILRUN/RNFT1/BIRC2/DCUN1D3/IKBKG/UBXN10/TOLLIP/N4BP1/UBXN1/MARK4/HDAC6/SHARPIN/VPS36/NPLOC4/UCHL3/DNAJB2/UBR5/AMFR/N4BP2/RNF111/UBE2N/NSFL1C/UBAP1/RNF168/SMAD3/TDG/FBXO7/GGA3/HERC2/FAF1/UBXN2B/FAF2/DCUN1D5/UBXN8/MVB12A/USP33/RAD23B/DDI2/PLAA/USP5/PELP1/ASCC2/VPS28/RBCK1/OTUB1/SMARCAD1/NUP62/GGA1/ZFAND2B/AUP1/HGS/RNF4/PML/CKS1B/SIMC1/SERBP1/TOM1L1/ADRM1/RAD23A/CBX4/UBXN11/BUB3/CKS2/UBE2L6/OTUB2/RNF19B/MDM2/TOP2A
GOBP_STEROL_METABOLIC_PROCESS	GOBP_STEROL_METABOLIC_PROCESS	0.023841337	APOA2/APOA1/CES1/SULT2A1/APOB/CYP1B1/PRKAA2/FAXDC2/SOAT2/LEPR/CYP11A1/CUBN/CH25H/VLDLR/CYP7B1/NR1H4/APOA4/CYP39A1/FGF1/ABCA1/CYP27A1

GOBP_CELL_REDOX_HOMEOSTASIS	GOBP_CELL_REDOX_HOMEOSTASIS	0.024017648	GPX1/TXNRD2/BOLA1/GLRX5/KRIT1/ERP44/SELENOT/DLD/SLC2A10/PRDX3/GLRX3/BOLA2B/PRDX1/SELENOS/TXNRD1/TXN/PRDX5/TXN2/BOLA3/GIT1/NQO1/PRDX2/ERO1A/GSR/NOS2
GOBP_MICROVILLUS_ASSEMBLY	GOBP_MICROVILLUS_ASSEMBLY	0.024055583	STK26/SLC9A3R1/MINK1/ATP8B1/TNIK/MYO1A/KLF5/EZR
GOBP_BROWN_FAT_CELL_DIFFERENTIATION	GOBP_BROWN_FAT_CELL_DIFFERENTIATION	0.0240627	ADIPOQ/FABP4/SLC2A4/FNDC5/EBF2/RGS2/FABP3/ADRB2/FFAR4/DIO2
GOBP_WATER_SOLUBLE_VITAMIN_METABOLIC_PROCESS	GOBP_WATER_SOLUBLE_VITAMIN_METABOLIC_PROCESS	0.024158659	PNPO/AKR1A1/SLC25A19/PDXP/FPGS/MMACHC/PDXK/DHFRP1/ACP3/TPK1/SHMT1/MTHFD2/SLC2A1/SLC52A2/SLC52A3/VNN1/GSTO2/DHFR/AMN/SLC19A1/CD320/SHMT2/PSAT1

GOBP_NUCLEOLUS_ORGANIZATION	GOBP_NUCLEOLUS_ORGANIZATION	0.024302817	USP36/PHF8/SMARCA5/SIRT1/MACROH2A1/BAZ2A/ACTR6/RPS19/RRN3/POLR1B/SUV39H1/DDX11/NOLC1
GOCC_TRANSPORT_VESICLE	GOCC_TRANSPORT_VESICLE	0.02430295	SYT4/DEFA4/SPX/PENK/SLC18A3/SYNGR4/SLC2A4/PCSK1/SCGN/GRIN2A/CPE/IGF1/PTPRN/STON1/DPYSL3/MAP6/SCG3/NGF/SLC18A2/GALNT15/DEFA6/SV2B/PCSK2/ATP6V1G2/NPTX1/IL33/AMPH/SLC17A7/CHGA/SYPL2/SV2A/SSPN/COPZ2/CPA3/CRISPLD2/PRRT1/CTTNBP2/SNAP25/LAMP5/SYT11/BSN/NTF3/LRRK2/BRSK1/CA4/DLG4/TRIM9/RAB3B/RAB3IL1/ITPR1/SEPTIN4/SYNDIG1/SLC18A1/APBA1/RAB3C/GABBR1/BGN/SYT1/MYRIP
GOBP_POSITIVE_REGULATION_OF_CELL_MORPHOGENESIS_INVOLVED_IN_DIFFERENTIATION	GOBP_POSITIVE_REGULATION_OF_CELL_MORPHOGENESIS_INVOLVED_IN_DIFFERENTIATION	0.024403568	APOA1/RELN/MYOC/FGA/FGG/FERMT2/LIMS2/FLNA/PAK3/PTPRD/DAB2/ILK/HAS2/FGB/ITGB3/MYADM/NRP1

GOMF_TRANSMEMBRANE_TRANSPORTER_BINDING	GOMF_TRANSMEMBRANE_TRANSPORTER_BINDING	0.024402623	ACTN2/KCNB1/FHL1/ANK2/AKAP6/ABCC9/KCNIP1/FLNA/SCN4B/HRC/KCNE4/CAV1/FGF13/STAC/BAG2/SLC8A1/PKD2/LRRK2/PRNP/ACTN1/FGF12/KCNAB1/SCN3B/SCN1B/SNTA1/LRRC26/KCNIP2/PKD1/CDH5/PDE4B/FYN/RIMS3
GOBP_NEGATIVE_REGULATION_OF_LEUKOCYTE_MIGRATION	GOBP_NEGATIVE_REGULATION_OF_LEUKOCYTE_MIGRATION	0.024424539	APOD/SLIT2/GREM1/CXCL12/IL33/CCL21/EMILIN1/CCL2/CCN3/CD200/DUSP1/RIPOR2
GOBP_CELLULAR_RESPONSE_TO_OXYGEN_RADICAL	GOBP_CELLULAR_RESPONSE_TO_OXYGEN_RADICAL	0.0244612	MPO/FBLN5/CD36/APOA4/SOD3
GOBP_FIBROBLAST_PROLIFERATION	GOBP_FIBROBLAST_PROLIFERATION	0.024509417	PLA2G1B/SFRP1/NGFR/FGF10/IGF1/DDR2/AGT/CAV1/ESR1/COL3A1/PDGFRA/FN1/PDGFD/PDGFC/ITGB3/CD248/PDGFRB

GOBP_VESICLE_TARGETING	GOBP_VESICLE_TARGETING	0.024894449	TBC1D23/TRAPPC8/EXOC4/TRAPPC2/EXOC3/TRAPPC1/PPP6C/EXOC8/SNAP23/TRAPPC13/WDR11/MAP4K2/EXOC7/SAR1A/TMED10/TRIP11/SAR1B/TFG/CSNK1D/KLHL12/TRAPPC12/EXOC2/TRAPPC9/TMED2/C17orf75/CUL3/PSEN1/GBF1/TRAPPC4/TRAPPC11/EXOC1/ARFGAP2/ARFGAP3/STARD3/TRAPPC2L/PDCD6/TRAPPC10/YKT6/FAM91A1/TMED9/PREB/AP1AR/EXOC6/SEC16A/SCRIB/AP1M2/MAPK15/IRAG2
GOBP_REGULATION_OF_AMPA_RECEPTOR_ACTIVITY	GOBP_REGULATION_OF_AMPA_RECEPTOR_ACTIVITY	0.024952989	RELN/CACNG4/NLGN1/PRRT1/ADRB2/NLGN3/MEF2C/NLGN2/ARC
GOBP_STEM_CELL_DIVISION	GOBP_STEM_CELL_DIVISION	0.024961041	MIR145/SFRP2/NAP1L2/ZBTB16/TGFB2/WWTR1/FZD7/SOX17/KIT/LBH/HOXB4

GOCC_U12_TYPE_SPLICEOSOMAL_COMPLEX	GOCC_U12_TYPE_SPLICEOSOMAL_COMPLEX	0.024977479	ZMAT5/SF3B4/SNRPGP15/SF3B6/SNRPE/SNRNP35/SNRPD2/SNRPD3/PDCD7/SNRPG/SF3B1/SNRPF/SNRNP48/PHF5A/LSM7/SNRPB/SF3B2/SNRPD1/YBX1/SNRNP25/SF3B5/SF3B3/DHX15
GOBP_REGULATION_OF_PROTEIN_SERINE_THREONINE_KINASE_ACTIVITY	GOBP_REGULATION_OF_PROTEIN_SERINE_THREONINE_KINASE_ACTIVITY	0.025125628	PLA2G1B/ELANE/SFRP1/ADIPOQ/MYOCD/NPFFR2/SLC8A2/HTR2A/GHR/EPGN/SFRP2/FERMT2/HTR2B/TNXB/ROR2/FGF2/IGF2/AGT/ATP2B4/THBS1/CCL19/CAV1/PRKAR2B/FZD10/RGS2/FGFR1/TENM1/SLC8A1/PKD2/PKIA/DAB2/DIRAS3/PKIG/DKK1/ADRB2/NTF3/CDKN1C/UCHL1/LRRK2/CCNJL/PDGFD/FGF1/CAB39L/DUSP1/BMPR1B/HEG1/NOX4/PDE5A/THY1/MEF2C/FAM20A/TCL1A/S100A12/PDGFC/ROBO1/FLT3/HERC5/IL34/KIT/PDGFRB/DIRAS1/RASIP1

GOBP_ATP_SYNTHESIS_COUPLED_ELECTRON_TRANSPORT	GOBP_ATP_SYNTHESIS_COUPLED_ELECTRON_TRANSPORT	0.025175725	NDUFB5/DNAJC15/NDUFC1/NDUFA1/COX7B/CHCHD2/NDUFB4/UQCRB/COX6B1/NDUFS8/UQCR11/NDUFB2/COA6/DGUOK/COX7C/NDUFA8/COX7A2L/NDUFA6/NDUFB10/DLD/NDUFS5/COX7A2P2/NDUFB3/NDUFA12/NDUFA5/UQCRQ/PARK7/BID/NDUFA9/NDUFB6/NDUFAF1/NDUFS6/UQCRFS1/AFG1L/NDUFA10/NDUFS2/NDUFA2/NDUFS1/COX7A2/NDUFS3/SDHD/NDUFB7/COX5B/NDUFAB1/UQCR10/COX4I1/COX6A1/UQCC3/CYCS/SDHB/COQ9/COX15/COX5A/UQCRC2/UQCRFS1P1/UQCRH/GHITM/COX8A/SDHA/NDUFV1/CDK1/NDUFS7/UQCRC1/UQCRHL/CYC1/MIR210/CCNB1

GOBP_NEGATIVE_REGULATION_OF_DNA_METABOLIC_PROCESS	GOBP_NEGATIVE_REGULATION_OF_DNA_METABOLIC_PROCESS	0.025274259	MSH3/RECQL5/DFFA/TENT4B/RNF169/SHLD2/SHLD3/DNAJC2/TP53BP1/MAD2L2/RIF1/WAPL/ATR/CYREN/H1-10/TERF2/RPS3/TFIP11/NUDT16L1/RTEL1/XRCC5/OTUB1/SMCHD1/XRN1/ACD/GMNN/MSH6/STN1/XRCC1/SRC/HSF1/NAT10/HNRNPU/HNRNPC/SLX4/EXOSC10/PIF1/MEN1/CDAN1/H1-2/FANCB/PARP1/UBQLN4/DONSON/BRCA2/PINX1/ZSCAN4/HELB/PDS5A/TRIM28/H1-3/GZMA/FBXO5/NBN/MSH2/TIPIN/HNRNPA1/TNKS/NIBAN2/PARPBP/CGAS/TIMELESS/CHEK1/OTUB2/PARP3/BLM/POLQ/TP53/AUNIP/DFFB/RMI2/MCIDAS/H1-4/CDT1/H1-5
GOBP_CELLULAR_RESPONSE_TO_NUTRIENT	GOBP_CELLULAR_RESPONSE_TO_NUTRIENT	0.025339316	PENK/SFRP1/CYP24A1/MN1/KANK2/NR1H4/FOLR2/SNAI2/LPL

GOMF_RNA_POLYMERASE_BINDING	GOMF_RNA_POLYMERASE_BINDING	0.025326059	RPRD2/RTF1/PCIF1/ELP2/PCF11/ZNF326/SCAF8/RPRD1B/AGO1/GTF2B/RPRD1A/CDC73/MAF1/BRD4/CCNT2/RPAP2/RECQL5/SMYD2/PPIB/SCAF1/PABPN1/CCNT1/SCAF4/CTR9/TCERG1/LEO1/WDR43/DHX9/BRCA1/UVSSA/RRN3/HNRNPU/PHRF1/SPTY2D1/ELOF1/ERCC5/ANP32B/YTHDC2/AGO2/PKN2/CCAR2
GOBP_REGULATION_OF_LYMPHOCYTE_APOPTOTIC_PROCESS	GOBP_REGULATION_OF_LYMPHOCYTE_APOPTOTIC_PROCESS	0.025501448	PRKCQ/CD74/ADAM8/PRELID1/ORMDL3/CD27/FADD/TP53/LGALS9/NOC2L/PDCD1/ADA/LYN/CD274/IL10/IDO1
GOBP_REGULATION_OF_CARBOHYDRATE_BIOSYNTHETIC_PROCESS	GOBP_REGULATION_OF_CARBOHYDRATE_BIOSYNTHETIC_PROCESS	0.025530504	GCG/ADIPOQ/ADCYAP1R1/SORBS1/PPP1R3C/IGF1/PTH1R/IGF2/LEPR/HAS2/PPP1R3G/NR3C1/NNMT/SOGA1/P2RY1/GPER1

GOBP_POSITIVE_REGULATION_OF_ALPHA_BETA_T_CELL_PROLIFERATION	GOBP_POSITIVE_REGULATION_OF_ALPHA_BETA_T_CELL_PROLIFERATION	0.025577836	PTPN22/SYK/HLA-E/IL23A/RIPK2/HLA-A/TYK2/IL18/PRKCQ/LGALS9/CD55/JAK2
GOMF_ENDONUCLEASE_ACTIVITY_ACTIVE_WITH_EITHER_RIBO_OR_DEOXYRIBONUCLEIC_ACIDS_AND_PRODUCING_5_PHOSPHOMONOESTERS	GOMF_ENDONUCLEASE_ACTIVITY_ACTIVE_WITH_EITHER_RIBO_OR_DEOXYRIBONUCLEIC_ACIDS_AND_PRODUCING_5_PHOSPHOMONOESTERS	0.025598817	DBR1/PLD6/DNASE1L1/NUDT16L1/FAN1/POP4/POP7/RPP25/AGO2/NUDT12/ELAC2/ZGRF1/MRPL44/GEN1/DNASE1L2/POP1/EXO1/FEN1/DNA2
GOCC_SYNAPTIC_VESICLE_MEMBRANE	GOCC_SYNAPTIC_VESICLE_MEMBRANE	0.025636603	SYT4/SYNGR4/SLC18A2/SV2B/ATP6V1G2/AMPH/SLC17A7/SYPL2/SV2A/PRRT1/LAMP5/BSN/LRRK2/RAB3B/SYNDIG1/SLC18A1/RAB3C/SYT1/SYP/SYT5/SYNGR1
GOBP_GENITALIA_DEVELOPMENT	GOBP_GENITALIA_DEVELOPMENT	0.025746849	FGF10/AR/ROR2/WT1/GREB1L/RBP4/ESR1/SHH/PDGFRA/FOXF2/AXL/MERTK/BMP5/BMP6/PKD1/HOXD13/TP63
GOBP_HYALURONAN_METABOLIC_PROCESS	GOBP_HYALURONAN_METABOLIC_PROCESS	0.025735012	ITIH2/HAS1/FGF2/LYVE1/ITIH3/ITIH5

GOBP_MITOCHONDRIAL_OUTER_MEMBRANE_PERMEABILIZATION_INVOLVED_IN_PROGRAMMED_CELL_DEATH	GOBP_MITOCHONDRIAL_OUTER_MEMBRANE_PERMEABILIZATION_INVOLVED_IN_PROGRAMMED_CELL_DEATH	0.025751957	BID/SLC35F6/GSK3A/RTL10/BAX/SLC25A5/RHOT2/ATP5IF1/ZNF205/HIP1R/PPIF/CHCHD10/VDAC2/BAK1/TMEM102/BBC3/GZMB
GOMF_ENDODEOXYRIBONUCLEASE_ACTIVITY	GOMF_ENDODEOXYRIBONUCLEASE_ACTIVITY	0.025851022	FAN1/DNASE2/XRCC1/MUS81/ERCC5/XRCC3/ZGRF1/RBBP8/GEN1/DNASE1L2/EXO1/DFFB/FEN1/DNA2/ENDOG
GOMF_SIGNAL_SEQUENCE_BINDING	GOMF_SIGNAL_SEQUENCE_BINDING	0.02590043	BRAP/TOMM40L/RANBP6/TNPO1/NUP153/NUP98/NUP214/IPO5/TOMM70/PEX5/TIMM22/KDELR1/KPNB1/KDELR2/SRP68/TNPO2/POM121C/NUP58/SEC61A1/KDELR3/POM121/AP2B1/PEX7/KPNA2/NOLC1/POM121B
GOBP_VENTRICULAR_SEPTUM_MORPHOGENESIS	GOBP_VENTRICULAR_SEPTUM_MORPHOGENESIS	0.025965632	NKX2-5/SLIT2/NOG/SLIT3/ZFPM2/TGFB2/HEYL/TGFBR3/ROBO1/ACVR1/APLNR/FGFR2/FZD1/SOX11

GOBP_POSITIVE_REGULATION_OF_SMOOTH_MUSCLE_CELL_PROLIFERATION	GOBP_POSITIVE_REGULATION_OF_SMOOTH_MUSCLE_CELL_PROLIFERATION	0.026008563	ELANE/RBPMS2/IGF1/DDR2/FGF2/NR4A3/SERPINF2/AGT/ADAMTS1/THBS1/MMP2/IL6/MIR27B/IGFBP5/GJA1/PDGFD/CCN4/S1PR1
GOBP_POSITIVE_REGULATION_OF_CELL_ADHESION	GOBP_POSITIVE_REGULATION_OF_CELL_ADHESION	0.026	ELANE/AZU1/APOA1/SFRP1/MYOC/VTN/FGA/CTSG/CCDC80/IGF1/FGG/ABI3BP/SFRP2/FERMT2/LIMS2/NPY/FLNA/ZBTB16/DUSP26/VIT/NR4A3/IGF2/COL26A1/LAMA2/CXCL12/GLI3/CHRD/COL8A1/SERPINF2/PRSS2/MMRN1/TPM1/CD36/CCL21/FOXF1/LAMA1/CCL19/CAV1/ECM2/SMARCD3/SELP/NODAL/CCN1/ANGPT1/TGFB2/ITGA5/IL6/SHH/EMILIN1/DAB2/FN1/CCL2/EFEMP2/MIR27B/EFNB3/FBLN2/WNT10B/FOXC2/COL16A1/ILK/HAS2/PDPN/FGB/NID1/TEK/THY1/EGR3/LAMC1/ITPKB/ITGB3/SELE/LGALS1/APBB1IP/CCR7/IL7R/MYADM/IL6ST/LEF1/PLPP3/GLI2/NDNF/NRP1/CR1/TNFSF18/RRAS/EDIL3/EGFLAM

GOBP_EAR_DEVELOPMENT	GOBP_EAR_DEVELOPMENT	0.026041667	COL2A1/NEUROD1/KCNK3/FGF10/SIX2/NOG/KCNK2/NKX3-2/BMPER/PLPPR4/FGF2/FRZB/GLI3/ZEB1/NTN1/ITGA8/TGFB3/SOBP/EDNRA/LIN7A/TBX18/TGFB2/HPCA/CYTL1/CDH23/HOXA2/SHH/OSR1/PRRX1/SLITRK6/DCHS1/ATOH1/TCF15/MAF/USH1G/ALDH1A3/MCOLN3/TWIST1/CTHRC1/ROR1/PDGFRB
GOBP_PROTEIN_MATURATION_BY_IRON_SULFUR_CLUSTER_TRANSFER	GOBP_PROTEIN_MATURATION_BY_IRON_SULFUR_CLUSTER_TRANSFER	0.026133545	MAGEF1/CIAO1/GLRX5/CIAO2A/GLRX3/BOLA2B/MMS19/LTO1/BOLA3/CIAO2B/CISD3
GOMF_E_BOX_BINDING	GOMF_E_BOX_BINDING	0.026155403	HAND2/NEUROD1/ASCL1/ZEB1/ATOH8/TAL1/TCF21/SNAI2/TCF15/TCF4/TWIST1/CIART/PER1/SNAI1/MITF

GOBP_PROTEIN_K63_LINKED_DEUBIQUITINATION	GOBP_PROTEIN_K63_LINKED_DEUBIQUITINATION	0.026197221	BABAM2/NOP53/OTUD1/ABRAXAS2/BABAM1/DESI2/OTUD4/USP8/SPATA2/UIMC1/STAMBP/USP33/BRCC3/BRCA1/YOD1/PSMD14/USP7/BARD1/STAMBPL1/VCP/OTUB2/SHMT2
GOCC_MICROBODY_LUMEN	GOCC_MICROBODY_LUMEN	0.026335721	SCP2/ACOX1/HSD17B4/HACL1/HMGCL/IDE/PRDX5/ACOX3/ABCD3/BAAT/FABP1/PEX7/ACAA1/GRHPR/TYSND1/GSTK1/NUDT12/DHRS4/EPHX2/IDH1/NOS2
GOBP_RESPONSE_TO_ESTRADIOL	GOBP_RESPONSE_TO_ESTRADIOL	0.026339214	PENK/SFRP1/TACR1/ADCYAP1R1/FGF10/ALDH1A2/CRYAB/APOB/PTGFR/MAP1B/CYP1B1/F7/AGT/MMP2/ESR1/DHH/OXTR/CCN2/SSTR2/PTCH1/GPER1/COL1A1/PDGFRB/OXT/RAMP3/TXNIP

GOBP_STEROID_METABOLIC_PROCESS	GOBP_STEROID_METABOLIC_PROCESS	0.026342452	AFP/APOA2/APOA1/CES1/CYP24A1/SULT2A1/APOB/CYP1B1/PRKAA2/UGT2B17/FAXDC2/CACNA1H/SOAT2/SERPINA6/RDH8/LEPR/CYP11A1/CUBN/CH25H/VLDLR/CYP7B1/UGT1A7/ESR1/NR1H4/APOA4/SHH/PDGFRA/DAB2/DHH/PBX1/IGFBP7/CGA/HSD17B6/HSD11B1/HSD17B14/MALRD1/CYP39A1/SNAI2/PDE8B/DKKL1/FGF1/DKK3/ABCA1/CHST10/CYP27A1

GOBP_CELLULAR_RESPONSE_TO_INTERFERON_GAMMA	GOBP_CELLULAR_RESPONSE_TO_INTERFERON_GAMMA	0.026484582	CCL15/VAMP3/NMI/ASS1/PDE12/EPRS1/CDC42EP4/TLR3/IL12RB1/IRF8/ACTG1/CCL5/MED1/RAB20/PARP14/CCL3L3/RAB43/SLC26A6/SOCS1/STAT1/CDC37/CX3CL1/CCL3/PPARG/GAPDH/TP53/LGALS9/CASP1/FLNB/GBP3/GBP1/NOS2/FASLG/CCL23/NLRC5/IRF1/JAK2/IFNG/GBP5/GBP4

GOBP_DETECTION_OF_STIMULUS	GOBP_DETECTION_OF_STIMULUS	0.026483051	KCNA1/TAC1/CASQ2/TACR1/NGFR/KCNMB1/NPFFR2/HTR2A/SRPX/CDH2/CST4/SSC5D/P2RX2/CXCL12/ELOVL4/FOXF1/TGFB3/CA6/PIP/PRPH2/NKX6-1/SERPINE2/MMP24/PKD2/C4B/SMO/PGLYRP1/PIEZO2/FFAR4/CST2/FAP/RYR2/LY96/SYT1/GNGT1/SEMA5B/CXCR4/KIT/BEST1
GOMF_RNA_POLYMERASE_II_CORE_PROMOTER_SEQUENCE_SPECIFIC_DNA_BINDING	GOMF_RNA_POLYMERASE_II_CORE_PROMOTER_SEQUENCE_SPECIFIC_DNA_BINDING	0.026524766	TBPL1/TAF1/TBP/GTF2B/GTF2A1/REST/RELA/H3-3B/MACROH2A1/H3-3A/RUVBL2/HDAC1/EZH2/STAT1/H2AZ1
GOCC_SMN_SM_PROTEIN_COMPLEX	GOCC_SMN_SM_PROTEIN_COMPLEX	0.026524766	SNRPGP15/SNRPE/GEMIN8/GEMIN6/GEMIN2/SNRPD2/SNRPD3/SMN1/SNRPG/STRAP/GEMIN7/GEMIN5/SNRPF/DDX20/SNRPB/SNRPD1/GEMIN4

GOMF_ACYLGLYCEROL_O_ACYLTRANSFERASE_ACTIVITY	GOMF_ACYLGLYCEROL_O_ACYLTRANSFERASE_ACTIVITY	0.026631594	PNPLA2/LCLAT1/LPCAT3/MBOAT7/GPAT3/AGPAT3/MOGAT2/GPAT4/PNPLA4/CRLS1/MBOAT2/DGAT1/LPCAT1/MBOAT1/AGPAT2/AGPAT5/LPCAT4
GOBP_SECRETION_BY_TISSUE	GOBP_SECRETION_BY_TISSUE	0.026631728	CEL/TACR1/FGF10/NPR3/SCT
GOBP_HISTONE_DEUBIQUITINATION	GOBP_HISTONE_DEUBIQUITINATION	0.026771012	TAF6/ENY2/ATXN7/BABAM2/USP21/USP15/BABAM1/TAF4/USP36/MYSM1/TAF9/TAF12/TAF2/TADA3/TRRAP/TAF5L/USP22/TADA1/UIMC1/BAP1/TADA2B/USP3/EPOP/TAF6L/SART3/ATXN7L3/BRCC3/BRCA1/USP7/BARD1/KAT2A/SF3B5/TAF5/SF3B3

GOCC_TRANS_GOLGI_NETWORK_MEMBRANE	GOCC_TRANS_GOLGI_NETWORK_MEMBRANE	0.026770075	CLTC/APP/RAB6A/KIF13A/USP6NL/LLGL1/RABEPK/COG6/VPS52/AFTPH/STX6/COG5/COG8/SCAMP3/COG1/AP4E1/LGR5/ARL1/COG4/HLA-DPB1/FUT9/CLTA/COG2/TMEM165/BAIAP3/M6PR/ARFIP1/CLBA1/VTI1A/SCAMP4/VPS51/VPS53/VPS54/HLA-DPA1/SCAMP2/GOLPH3L/VAMP3/COG7/RGP1/AP1B1/AP1G1/HLA-DRA/RAB8A/ARFIP2/HLA-DQA2/ATP7B/PICK1/HLA-DQA1/RAB43/HLA-DRB5/CD74/AP4B1/ARFRP1/HLA-DRB1/LGR6/AP1S1/STX10/AP1S3/ATP2C2/HLA-DQB1/HLA-DQB2/AP1M2
GOBP_SPLEEN_DEVELOPMENT	GOBP_SPLEEN_DEVELOPMENT	0.026874429	NKX2-5/FGF10/BARX1/NKX3-2/ONECUT1/PBX1/TCF21

GOCC_POLYSOME	GOCC_POLYSOME	0.026866225	AGO1/RPL11/VBP1/IMPACT/RPL38/PSMA1/RPS21/NUFIP2/RPL24/PNPT1/FMR1/RPS4X/HDLBP/DRG1/RPL10A/RPL32/DIS3L2/RPL6/RPS3/EIF3H/RPS23/RPL30/MSI2/RPL18/LARP4B/BTF3/FXR2/RPL7A/PSMA6/RPS28/DHX9/EIF4G1/RPS6/CALR/MCRS1/EIF2S1/EEF2/RPL19/GCN1/AGO2/RPL36/LARP1/RPL41/RPL18A/RPL8
GOBP_POSITIVE_REGULATION_OF_CD4_POSITIVE_ALPHA_BETA_T_CELL_DIFFERENTIATION	GOBP_POSITIVE_REGULATION_OF_CD4_POSITIVE_ALPHA_BETA_T_CELL_DIFFERENTIATION	0.026919892	SHB/IL23A/RIPK2/KLHL25/HLA-DRA/IL12RB1/SOCS1/ZBTB7B/IL18/HLA-DRB1/LGALS9/PRKCZ/IFNG/MYB

GOBP_NEGATIVE_REGULATION_OF_INFLAMMATORY_RESPONSE	GOBP_NEGATIVE_REGULATION_OF_INFLAMMATORY_RESPONSE	0.026910657	ELANE/APOA1/ADIPOQ/MIR145/PTGIS/IGF1/NPY/FOXF1/GPR17/SERPINF1/CNR2/HGF/WFDC1/NR1H4/KRT1/PGLYRP1/SYT11/ISL1/FFAR4/CCN3/CD200/MIR149/TEK/MIR222/FCGR2B/IL20RB/GPER1/HAMP/FPR2/RORA/NLRP3/MIR3909/GHRL/CDH5/FYN/CST7/PROC/ALOX5/SOCS5/CALCRL/GATA3
GOBP_POSITIVE_REGULATION_OF_VIRAL_PROCESS	GOBP_POSITIVE_REGULATION_OF_VIRAL_PROCESS	0.027045333	NOTCH1/DHX9/SRPK1/CNOT7/TARBP2/PABPC1/PPID/PPIA/TRIM21/PDE12/TRIM11/PKN2/RAD23A/CCL5/LARP1/P4HB/CD74/FURIN/KPNA2/HACD3/VPS37B/HLA-DRB1/TMPRSS2/LGALS9/TMPRSS4/TOP2A

GOBP_REGULATION_OF_ESTABLISHMENT_OF_PROTEIN_LOCALIZATION	GOBP_REGULATION_OF_ESTABLISHMENT_OF_PROTEIN_LOCALIZATION	0.027054108	PLA2G1B/GCG/SYT4/UCN3/CARTPT/CELA2A/SFRP1/ADIPOQ/NEUROD1/NNAT/KCNB1/FGA/PCSK1/ANKRD1/ADCY5/APOD/KCNA5/IGF1/FGG/FLNA/CNR1/MYOM1/GLI3/GIP/PRKD1/CD36/CHGA/RAB23/TGFB3/MAPT/APBB1/RFX6/NKX6-1/ANGPT1/TGFB2/TENM1/HPCA/RBP4/IL6/PRKN/PKIA/MYH10/NR1H4/SHH/SMO/PKIG/SNAP25/ISL1/CCN3/LRRK2/CD200/STOM/GPR27/PTP4A3/PRNP/KLF7/FGB/NLGN2/BLK/CORO2B/GPLD1/MYRIP/WLS/VEGFC
GOBP_CARDIAC_MUSCLE_CELL_MEMBRANE_REPOLARIZATION	GOBP_CARDIAC_MUSCLE_CELL_MEMBRANE_REPOLARIZATION	0.027145888	ANK2/KCNA5/KCNH6/FLNA/SCN4B/KCNJ8/KCNE4/KCNH2/CACNA2D1
GOMF_NON_MEMBRANE_SPANNING_PROTEIN_TYROSINE_KINASE_ACTIVITY	GOMF_NON_MEMBRANE_SPANNING_PROTEIN_TYROSINE_KINASE_ACTIVITY	0.027206304	PTK2/STK16/WEE1/PKDCC/TEC/PTK2B/SRC/PRKCD/SYK/RIPK2/PTK6/TYK2/CSK/TNK2/MELK/STYK1/FRK/LYN/TNK1/LCK/SRMS/JAK2

GOBP_REGULATION_OF_CELLULAR_KETONE_METABOLIC_PROCESS	GOBP_REGULATION_OF_CELLULAR_KETONE_METABOLIC_PROCESS	0.027233115	APOA1/APOC3/ADIPOQ/CES1/RGN/CNR1/NR4A3/GIP/FMO1/PDK4/ATP2B4/CAV1/FABP3/NR1H4/APOA4/AVPR1A/DAB2/MALRD1/DKKL1/DKK3/FMO2/TWIST1/ACACB/ELOVL5
GOBP_NUCLEOBASE_CONTAINING_SMALL_MOLECULE_BIOSYNTHETIC_P	GOBP_NUCLEOBASE_CONTAINING_SMALL_MOLECULE_BIOSYNTHETIC_PROCESS	0.02757205	APRT/ADK/TK2/PNP/MTAP/PGM2/DTYMK/ADA
GOBP_CELLULAR_RESPONSE_TO_ALDEHYDE	GOBP_CELLULAR_RESPONSE_TO_ALDEHYDE	0.0275595	SCNN1B/RDH12/AIFM1/ALDH1A1/AKR1A1/AKR1B10/SCNN1G/IGF1R

GOBP_NATURAL_KILLER_CELL_ACTIVATION	GOBP_NATURAL_KILLER_CELL_ACTIVATION	0.02754294	ULBP1/HECTD1/TUSC2/BAG6/TYRO3/MICA/ELF4/KLRC1/KLRD1/UNC13D/PRDX1/CASP8/CD244/IL18R1/CORO1A/ULBP2/PTPN22/IL15RA/HLA-E/IL23A/DCAF15/AP1G1/KLRC2/TYK2/SLAMF7/IL2RB/TICAM1/RHBDD3/IL21R/IL18/HLA-F/NKG7/ULBP3/ZNF683/JAK2/IFNE
GOBP_NEGATIVE_REGULATION_OF_LIPID_METABOLIC_PROCESS	GOBP_NEGATIVE_REGULATION_OF_LIPID_METABOLIC_PROCESS	0.027593819	APOA2/APOC3/CIDEA/APOD/CNR1/FMO1/HCAR1/MALRD1/SNAI2/PDE8B/DKKL1/DKK3/FMO2/GPLD1/ACACB/GPER1/CCDC3/PDE3B/BMP5

GOBP_BONE_MORPHOGENESIS	GOBP_BONE_MORPHOGENESIS	0.027746948	CER1/COL2A1/PAPPA2/GHR/SFRP4/FREM1/GLI3/RAB23/TGFB3/TMEM119/COMP/COL3A1/MMP16/HAS2/BMPR1B/STC1/MEF2C/LTBP3/ANXA6/TWIST1/COL1A1/NPPC/FOXN3/FGFR2/BMP6
GOBP_SEGMENTATION	GOBP_SEGMENTATION	0.027839644	SFRP1/MEOX2/ALDH1A2/DLL3/SFRP2/WT1/FOXF1/CRB2/TBX18/NRP2/TDRD10/HOXA2/SHH/OSR1/DKK1/MEOX1/FOXC2/TCF15/IRX3/LEF1/SEMA3C/NRP1
GOBP_POSITIVE_REGULATION_OF_VIRAL_LIFE_CYCLE	GOBP_POSITIVE_REGULATION_OF_VIRAL_LIFE_CYCLE	0.027913509	TRIM21/TRIM11/P4HB/CD74/FURIN/KPNA2/VPS37B/HLA-DRB1/TMPRSS2/LGALS9/TMPRSS4

GOBP_CELL_CELL_JUNCTION_ORGANIZATION	GOBP_CELL_CELL_JUNCTION_ORGANIZATION	0.028008299	PRTN3/CDH19/ANK2/FERMT2/LIMS2/CDH2/SVEP1/FLNA/CLDN6/MPDZ/CLDN11/JAM3/NLGN4X/AGT/CNTNAP1/TGFB3/GJC1/CAV1/TGFB2/GPBAR1/CDH11/HOPX/NR1H4/KIRREL1/CLDN9/GJA1/SNAI2/DCHS1/CTNND2/EPB41L3/CLDN19/HEG1/RAMP2/CDH6/CLDN5/MTSS1/NLGN2/IRX3/TBX5/PKP1/MYADM/MPP7/PECAM1/RDX/APLNR/GJA4/BMP6/NPHP1/GJA5/SNAI1/TLN1/F2R/CDH5/DSG1/NUMBL/CLDN10/PKHD1/ACE2/VCL/TGFBR1/CSF1R
GOCC_TIM23_MITOCHONDRIAL_IMPORT_INNER_MEMBRANE_TRANSLOCASE_COMPLEX	GOCC_TIM23_MITOCHONDRIAL_IMPORT_INNER_MEMBRANE_TRANSLOCASE_COMPLEX	0.028051551	TIMM10/PAM16/TIMM17B/DNAJC15/TIMM23/TIMM23B/TIMM17A/TIMM50/GRPEL2/TIMM21/HSPA9/GRPEL1/TIMM44
GOBP_COENZYME_A_METABOLIC_PROCESS	GOBP_COENZYME_A_METABOLIC_PROCESS	0.028749453	DCAKD/PPCDC/NUDT19/COASY/PPCS/PANK4/PANK1/PANK3/ACOT7/ACLY/MCCC2/HMGCR/NUDT8

GOBP_VIRAL_TRANSLATION	GOBP_VIRAL_TRANSLATION	0.028749453	EIF3F/SSB/EIF2AK4/SHFL/ATG12/MCTS1/PCBP2/DENR/EIF2D/EIF3B/EIF3D/ATG5/DHX9/EIF3A/EIF3G/PTBP1
GOBP_REGULATION_OF_MYOBLAST_FUSION	GOBP_REGULATION_OF_MYOBLAST_FUSION	0.028749453	GDF15/CXCL9/CXCL10/SCGB3A1
GOBP_REGULATION_OF_HISTONE_H3_K9_METHYLATION	GOBP_REGULATION_OF_HISTONE_H3_K9_METHYLATION	0.028947333	EHMT1/SMARCB1/PIH1D1/BRCA1/LMNA/EHMT2/KDM1A/KDM4C/KDM4A/DNMT1/MYB/H1-5
GOBP_RIBOSOMAL_SUBUNIT_EXPORT_FROM_NUCLEUS	GOBP_RIBOSOMAL_SUBUNIT_EXPORT_FROM_NUCLEUS	0.029010556	LSG1/NMD3/RIOK2/EIF6/LTV1/XPO1/ABCE1/MDN1/RAN/NOP9/NUP88/RPS15/RRS1/NPM1
GOBP_NERVE_DEVELOPMENT	GOBP_NERVE_DEVELOPMENT	0.02900232	NKX2-2/NGFR/NGF/PLXNA4/NPTX1/GLI3/EDNRA/LPAR1/SERPINE2/NRP2/SLITRK6/ISL1/NTF3/ILK/CHRNB2/SULF1/ACKR3/ADARB1/NRP1

GOBP_RHO_PROTEIN_SIGNAL_TRANSDUCTION	GOBP_RHO_PROTEIN_SIGNAL_TRANSDUCTION	0.029032258	APOA1/APOC3/AGTR1/MYOC/NGFR/ARHGEF25/NTN1/LPAR4/CDC42EP3/GPR17/KANK2/LPAR1/RHOJ/COL3A1/DLC1/ABCA1/HEG1/RHOB/PDPN/COL1A2/RIPOR2/RASGRF2/ROBO1/STARD13/PDGFRB/RASIP1/NRP1/ARHGAP29/GPR20/CAVIN4
GOBP_POSITIVE_REGULATION_OF_SYNCYTIUM_FORMATION_BY_PLASM	GOBP_POSITIVE_REGULATION_OF_SYNCYTIUM_FORMATION_BY_PLASMA_MEMBRANE_FUSIO	0.029058651	ADAM9/GDF15/CXCL9/SCGB3A1
GOBP_ANTIGEN_PROCESSING_AND_PRESENTATION_OF_ENDOGENOUS_ANTIGEN	GOBP_ANTIGEN_PROCESSING_AND_PRESENTATION_OF_ENDOGENOUS_ANTIGEN	0.029120363	AZGP1/IDE/HLA-H/TAPBP/ERAP1/HLA-E/HLA-DRA/HLA-B/HLA-A/CD74/HLA-F/HLA-C/ERAP2/TAP2/HLA-DRB1/TAP1
GOBP_MRNA_CLEAVAGE	GOBP_MRNA_CLEAVAGE	0.029170847	POLR1H/ERN1/CPSF7/CPSF6/CPSF2/CSTF3/CPSF3/CPSF4/TUT1/NCBP1/FIP1L1/NUDT21/CSTF2/CPSF1

GOMF_N_ACETYLTRANSFERASE_ACTIVITY	GOMF_N_ACETYLTRANSFERASE_ACTIVITY	0.029177239	TAF1/NAGS/SAT1/CLOCK/KAT6B/EP300/GTF2B/ATAT1/KAT6A/NAA30/TAF9/USP22/NAA60/NAA20/NAA15/NAA80/HAT1/GTF3C4/KAT5/ESCO1/NAT9/NAA25/NAA50/NAT10/NAA10/SRCAP/MCM3AP/BRCA2/NAA40/KAT2A/NAT1/SMARCE1/NAT2/GNPNAT1/MED24/ESCO2

GOBP_NEGATIVE_REGULATION_OF_CELL_PROJECTION_ORGANIZATION	GOBP_NEGATIVE_REGULATION_OF_CELL_PROJECTION_ORGANIZATION	0.029227557	STMN2/EPHA7/RGMA/FAT3/SLIT2/DPYSL3/FLNA/DAB1/NLGN1/SEMA3E/NTN1/CCL21/NFATC4/FGF13/LPAR1/NR2F1/SPOCK1/DAB2/DKK1/SPART/EFNB3/LRRK2/DPYSL5/DENND5A/SEMA6A/VIM/SEMA6D/PRNP/PMP22/THY1/SEMA6C/SEMA3G/MAP2/SEMA5B/SEMA3D/MIR196A1/SEMA3C/ARHGAP24/NRP1/CRMP1/PFN2

GOBP_DNA_TEMPLATED_TRANSCRIPTION_INITIATION	GOBP_DNA_TEMPLATED_TRANSCRIPTION_INITIATION	0.0293814	TBPL1/BDP1/SETX/E2F3/CCNH/MED28/TAF1/GTF2E1/MED22/PSMC6/PWWP2A/TBP/MED10/CDK4/TAF13/TAF4/GTF2B/GTF2A1/ERCC3/ELOB/CRCP/ERCC6/HMGB1/TAF9/MED30/UBTF/ELOC/SMARCA5/TAF12/MED12/TAF2/MED8/DR1/MED11/ELOA/ZNF451/MED27/BCLAF1/GTF3C1/GTF2H2/MED13/TFB2M/CDK7/GTF3C4/POLR1E/MED14/TAF1C/POLR2D/BAZ2A/TAF6L/TFAM/MED25/SMARCB1/MED23/PIH1D1/TAF4B/MED16/POLR3H/RRN3/MED15/MED9/THRA/HNF1A/PPM1D/PAXIP1/BRF2/GTF2F1/BRF1/FOSL1/MED26/TFB1M/TAF5/MED1/GTF3C5/MED18/GTF2E2/GTF2A2/MAZ/SMARCA4/CTNNBIP1/POLRMT/HNF1B/TP53/MED24/E2F2
GOBP_DETECTION_OF_ABIOTIC_STIMULUS	GOBP_DETECTION_OF_ABIOTIC_STIMULUS	0.029445074	KCNA1/TAC1/TACR1/NGFR/NPFFR2/HTR2A/CDH2/CXCL12/ELOVL4/PRPH2/SERPINE2/MMP24/PKD2/PIEZO2/GNGT1/SEMA5B/CXCR4/KIT/BEST1/BACE1/LXN/PDE6B

GOBP_L_ALPHA_AMINO_ACID_TRANSMEMBRANE_TRANSPORT	GOBP_L_ALPHA_AMINO_ACID_TRANSMEMBRANE_TRANSPORT	0.02953228	ATP1A2/SLC38A3/RGS4/SLC7A2/AGT/SLC17A7/SLC7A10/SLC38A4/RGS2/SLC1A7/SLC7A9
GOMF_DEMETHYLASE_ACTIVITY	GOMF_DEMETHYLASE_ACTIVITY	0.029517776	KDM6A/ALKBH1/RIOX1/KDM7A/JMJD6/KDM6B/PHF8/KDM3A/KDM8/RIOX2/ALKBH5/KDM3B/RSBN1/KDM4B/ALKBH4/KDM5C/KDM2B/KDM1A/KDM4C/KDM1B/FBXL19/KDM4A/MMACHC/ALKBH2/CYP51A1/HR
GOBP_REGULATION_OF_DOUBLE_STRAND_BREAK_REPAIR_VIA_NONHOMOLOGOUS_END_JOINING	GOBP_REGULATION_OF_DOUBLE_STRAND_BREAK_REPAIR_VIA_NONHOMOLOGOUS_END_JOINING	0.02959721	SHLD2/SHLD3/WAS/MAD2L2/PNKP/RIF1/CYREN/TFIP11/DEK/PARP9/NUDT16L1/SMCHD1/DTX3L/HSF1/PRKDC/KMT5C/SIRT7/WRAP53/PARP3/AUNIP/NSD2
GOBP_REGULATION_OF_RESPONSE_TO_TUMOR_CELL	GOBP_REGULATION_OF_RESPONSE_TO_TUMOR_CELL	0.02965921	AHR/DAPK1/HMGB1/CEACAM1/CRTAM/CD226/NECTIN2/IL4I1/HSPD1/PVR/HLA-DRB1/CD274

GOBP_GLYCEROLIPID_CATABOLIC_PROCESS	GOBP_GLYCEROLIPID_CATABOLIC_PROCESS	0.029761905	APOA2/PNLIPRP2/APOA1/APOC3/APOB/PLA2G5/GPIHBP1/PLA2G4C/APOA4/LPL/PNPLA1/GPLD1
GOBP_VESICLE_TARGETING_ROUGH_ER_TO_CIS_GOLGI	GOBP_VESICLE_TARGETING_ROUGH_ER_TO_CIS_GOLGI	0.029866288	TRAPPC8/TRAPPC2/TRAPPC1/PPP6C/SAR1A/TMED10/SAR1B/TFG/CSNK1D/KLHL12/TRAPPC12/TMED2/CUL3/TRAPPC4/TRAPPC11/TRAPPC2L/PDCD6/PREB/SEC16A/MAPK15
GOBP_LATE_ENDOSOME_TO_VACUOLE_TRANSPORT_VIA_MULTIVESICULAR_BODY_SORTING_PATHWAY	GOBP_LATE_ENDOSOME_TO_VACUOLE_TRANSPORT_VIA_MULTIVESICULAR_BODY_SORTING_PATHWAY	0.029939826	STAM2/SNF8/STAM/TMEM50B/VPS25/TMEM50A/VPS36/VPS37A/VPS37D/UBAP1/MVB12A/VPS37C/CHMP6/VTA1/VPS28/LEPROTL1/CHMP7/HGS/CHMP5/PTPN23/VPS37B/CHMP4C
GOBP_REGULATION_OF_OXIDOREDUCTASE_ACTIVITY	GOBP_REGULATION_OF_OXIDOREDUCTASE_ACTIVITY	0.029966704	HP/AGTR1/NPR3/RGN/AOC3/HTR2B/CNR1/AGT/ATP2B4/CAV1/CNR2/PRKN/MIR27B/LRRK2

GOBP_PALLIUM_DEVELOPMENT	GOBP_PALLIUM_DEVELOPMENT	0.030139935	KCNA1/RELN/NEUROD1/ASCL1/FOXP2/SCT/SLIT2/CDH2/NPY/IGF2BP1/DCLK2/DAB1/DIXDC1/CDON/GLI3/ATP2B4/FAT4/FGF13/FEZ1/COL3A1/SMO/ID4/ATOH1/BMERB1/TMEM108/LHX6/NEFL
GOBP_REGULATION_OF_TISSUE_REMODELING	GOBP_REGULATION_OF_TISSUE_REMODELING	0.030197445	CARTPT/SFRP1/HAND2/THBS4/DDR2/TF/GREM1/AGT/LEPR/PDK4/TNFRSF11B/TMEM119/IL6/MIR27B/S1PR1/GPNMB/LTBP3/ITGB3

GOBP_REGULATION_OF_SMALL_MOLECULE_METABOLIC_PROCESS	GOBP_REGULATION_OF_SMALL_MOLECULE_METABOLIC_PROCESS	0.030272452	GCG/APOA1/APOC3/ADIPOQ/ADCYAP1R1/SORBS1/CES1/HTR2A/APOB/RGN/IGF1/PRKAA2/CNR1/PTH1R/NR4A3/IGF2/GIP/FMO1/LEPR/PDK4/NCS1/ATP2B4/PID1/CAV1/FABP3/HPCA/GUCA2A/IGFBP4/PRKN/ZBTB20/NR1H4/APOA4/AVPR1A/DAB2/MALRD1/SNAI2/DKKL1/FGF1/DKK3/FMO2/PPP1R3G/NR3C1/TWIST1/NNMT/GPLD1/SOGA1/ACACB/H19/P2RY1/ELOVL5/GPER1/KIT
GOBP_POSITIVE_REGULATION_OF_PEPTIDYL_SERINE_PHOSPHORYLATION	GOBP_POSITIVE_REGULATION_OF_PEPTIDYL_SERINE_PHOSPHORYLATION	0.030337079	GCG/SFRP2/NGF/TRIM6/PRKD1/ATP2B4/CSF3/CAV1/ANGPT1/TENM1/IL6/NTF3/IL11/TCL1A
GOBP_RESPONSE_TO_ALKALOID	GOBP_RESPONSE_TO_ALKALOID	0.030337079	BCHE/CASQ2/PENK/TACR1/PCSK1/HTR2A/GHR/RYR3/RGS4/CACNG4/CNR1/FOSB/SLC8A1

GOCC_KERATIN_FILAMENT	GOCC_KERATIN_FILAMENT	0.030357559	KRT17/KRT80/KRT6B/EPPK1/KRT18/KRT7/FAM83H/KRT87P/KRT8/KRT81/KRTAP3-2
GOBP_POSITIVE_REGULATION_OF_TRANSMEMBRANE_RECEPTOR_PROTEIN_SERINE_THREONINE_KINASE_SIGNALING_PATHWAY	GOBP_POSITIVE_REGULATION_OF_TRANSMEMBRANE_RECEPTOR_PROTEIN_SERINE_THREONINE_KINASE_SIGNALING_PATHWAY	0.030501089	BMP3/MYOCD/GDF6/GPC3/TGFB1I1/GDF10/ITGA8/TGFB3/LEFTY2/THBS1/ZNF423/CRB2/NODAL/CCN1/TGFB2/RBPMS/DAB2/GDF9/GDF7/CDKN1C/ZEB2/ILK/TGFBR3/INHBA/INHA/SCUBE3/SULF1/ELAPOR2/ACVR1
GOBP_EMBRYONIC_PATTERN_SPECIFICATION	GOBP_EMBRYONIC_PATTERN_SPECIFICATION	0.030552291	CHRDL1/FGF10/MEOX2/ZBTB16/WT1/NODAL/MEIS1/NRP2/TDRD10/SHH/MEOX1/MEIS3P1/PTCH1/MEIS3/CXXC4/NRP1/FGFR2/HOXD8

GOBP_RESPONSE_TO_ORGANOPHOSPHORUS	GOBP_RESPONSE_TO_ORGANOPHOSPHORUS	0.030567686	AQP8/PENK/ADIPOQ/CDO1/AKAP6/ABCC9/RYR3/CYP1B1/P2RX2/WT1/PDE3A/P2RX1/LPAR1/PDE2A/FOSB/SLC8A1/PKD2/MMP19/IGFBP5/WNT10B/THBD/SLC26A3/STC1
GOBP_NEGATIVE_REGULATION_OF_CILIUM_ASSEMBLY	GOBP_NEGATIVE_REGULATION_OF_CILIUM_ASSEMBLY	0.030709067	CEP97/CCP110/EVI5L/MPHOSPH9/TESK1/CDK10/TCHP/TBC1D7/GDI2/LIMK2/TBC1D30/DNM2/KIF24
GOMF_DNA_BINDING_BENDING	GOMF_DNA_BINDING_BENDING	0.030709067	TFAM/HMGB3/FOXL1/HMGB2/FOXD1/HMGA1/TOP2A/FOXC1/FOXD4/FOXI1
GOCC_CARBOXY_TERMINAL_DOMAIN_PROTEIN_KINASE_COMPLEX	GOCC_CARBOXY_TERMINAL_DOMAIN_PROTEIN_KINASE_COMPLEX	0.030709067	CDK9/GTF2H1/CCNH/MNAT1/ERCC3/CDK13/CCNT2/ERCC2/CDK12/GTF2H2/CCNT1/GTF2H2C/CDK7/CCNK/MMS19/GTF2H3

GOBP_VESICLE_COATING	GOBP_VESICLE_COATING	0.030682192	TRAPPC8/TRAPPC2/AP3S1/TRAPPC1/PPP6C/TRAPPC13/SAR1A/AP3S2/TMED10/SAR1B/TFG/CSNK1D/KLHL12/TRAPPC12/TRAPPC9/TMED2/CUL3/GBF1/TRAPPC4/TRAPPC11/AP3M2/ARFGAP2/ARFGAP3/TRAPPC2L/PDCD6/TRAPPC10/AP3D1/TMED9/PREB/SEC16A/MAPK15
GOBP_REGULATION_OF_LIPID_BIOSYNTHETIC_PROCESS	GOBP_REGULATION_OF_LIPID_BIOSYNTHETIC_PROCESS	0.030752916	APOA1/APOC3/SORBS1/CES1/HTR2A/APOB/RGN/THRSP/HTR2B/GIP/PDK4/CCN1/FABP3/ZBTB20/NR1H4/APOA4/AVPR1A/DAB2/DHH/IGFBP7/CGA/MALRD1/SNAI2/PDE8B/DKKL1/FGF1/DKK3/NR3C1/GPLD1/ENPP7/ELOVL5/GPER1/CCDC3

GOBP_REGULATION_OF_INNATE_IMMUNE_RESPONSE	GOBP_REGULATION_OF_INNATE_IMMUNE_RESPONSE	0.030823471	APPL2/XRCC6/TRAFD1/IRF7/OAS1/TYRO3/MICA/TOMM70/CRTAM/ADAR/DUSP10/IKBKB/LYAR/ATG5/PARP9/ZDHHC18/HSP90AA1/YTHDF2/XRCC5/NONO/NCF1/KLRC1/KLRD1/CD226/IFI35/PTPN6/DHX58/NR1H3/CASP8/SFPQ/SLAMF8/DHX9/RPS19/SRC/POLR3B/PLCG2/ERAP1/IRF3/CNOT7/PYCARD/CACTIN/CASP6/PTPN22/NMI/SYK/PARP1/HLA-E/TRIM21/GFER/NECTIN2/RBM14/AP1G1/HLA-B/KLRC2/HLA-A/PRKDC/PSPC1/IKBKE/TRIM41/HMGB2/CCL5/MED1/PARP14/USP18/OAS3/SH2D1B/MMP12/SOCS1/TKFC/DRD2/CDC37/HLA-F/PVR/ADAM8/CGAS/PPARG/FADD/BIRC3/LGALS9/KIR2DL4/TTLL12/LYN/LAG3/POLR3G/NLRC5/IRF1/LRP8/GBP5/AIM2
GOBP_REGULATION_OF_FATTY_ACID_METABOLIC_PROCESS	GOBP_REGULATION_OF_FATTY_ACID_METABOLIC_PROCESS	0.030888337	APOA1/APOC3/ADIPOQ/RGN/CNR1/NR4A3/GIP/FMO1/PDK4/CAV1/FABP3/APOA4/AVPR1A/FMO2/TWIST1/ACACB/ELOVL5

GOBP_ENERGY_RESERVE_METABOLIC_PROCESS	GOBP_ENERGY_RESERVE_METABOLIC_PROCESS	0.030963303	PYGM/PPP1R1A/SORBS1/PPP1R3C/IGF1/IGF2/LEPR/GFPT2/PID1/RUBCNL/PHKG1/KL/PPP1R3G/GABARAPL1/IL6ST/GYG1/PYGL/IRS1/EPM2AIP1/ADGRF5/GNMT
GOMF_ORGANIC_HYDROXY_COMPOUND_TRANSMEMBRANE_TRANSPORTER_ACTIVITY	GOMF_ORGANIC_HYDROXY_COMPOUND_TRANSMEMBRANE_TRANSPORTER_ACTIVITY	0.030982583	SLC16A3/ABCC3/SLC5A1/MFSD10/SLC16A8/SLC16A1/SLC10A5/ASIC3/SLC22A1/STRA6/SLC10A2/SLCO1B3/SLC6A3/AQP2

GOBP_RNA_3_END_PROCESSING	GOBP_RNA_3_END_PROCESSING	0.031011454	RPS21/ERI3/ZC3H14/RPRD1A/CDC73/CLP1/PNPT1/PAPOLA/WDR33/TRNT1/APP/VIRMA/SYMPK/CPSF7/CPSF6/TENT4B/MTPAP/SSU72/TENT2/EXOSC8/CPSF2/ZNF473/USB1/PARN/CSTF3/HSD17B10/CPSF3/LEO1/TUT7/TENT4A/CPSF4/INTS12/POLR2D/TUT1/NCBP1/INTS7/FIP1L1/INTS14/HSF1/INTS9/DKC1/INTS8/FBL/EXOSC10/INTS1/EXOSC2/EXOSC7/PABPC1/BARD1/EXOSC5/NUDT21/SUPV3L1/INTS5/ZC3H3/EXOSC9/CSTF2/POLR3K/EXOSC4/SNRPA/TOE1/ELAC2/CPSF1/INTS2/ERI2/GRSF1/SLBP/ERI1
GOBP_EMBRYONIC_SKELETAL_SYSTEM_DEVELOPMENT	GOBP_EMBRYONIC_SKELETAL_SYSTEM_DEVELOPMENT	0.031055061	COL2A1/HAND2/SIX2/NOG/NKX3-2/HOXD10/GLI3/ZEB1/WNT9A/TGFB3/HOXA4/EDNRA/HOXD1/NODAL/RBP4/HOXA2/SHH/PDGFRA/OSR1/PBX1/MMP16/PRRX1/FOXC2/HOXD4/TBX15/TWIST1/HOXA5/HOXD9/SULF1/COL1A1

GOBP_HYDROGEN_PEROXIDE_METABOLIC_PROCESS	GOBP_HYDROGEN_PEROXIDE_METABOLIC_PROCESS	0.031100478	MPO/HP/MMP3/APOA4/HBB/GPX3/TPO
GOBP_POSITIVE_REGULATION_OF_STEM_CELL_PROLIFERATION	GOBP_POSITIVE_REGULATION_OF_STEM_CELL_PROLIFERATION	0.031261863	FGF10/FERMT2/FGF2/MIR320C2/THPO/PBX1/PRRX1/WNT10B/ID4/GJA1/MIR320D1/LTBP3/VEGFC/HOXA3/SOX11/CCNE1/TP63/MIR320B2/MIR320E/HMGA2
GOBP_NEGATIVE_REGULATION_OF_EPITHELIAL_CELL_DIFFERENTIATION	GOBP_NEGATIVE_REGULATION_OF_EPITHELIAL_CELL_DIFFERENTIATION	0.03125	REG3G/FGF10/SIX2/FRZB/ZEB1/CAV1/S1PR3/NODAL/SMO/OSR1

GOBP_EPIGENETIC_REGULATION_OF_GENE_EXPRESSION	GOBP_EPIGENETIC_REGULATION_OF_GENE_EXPRESSION	0.031293378	CDK2/SUZ12/TDG/CBX3/TPR/H3C12/SMARCD1/RIF1/CTCF/SETDB1/GLMN/H3-3B/RBM15B/CTR9/SMYD5/MTA1/H3C10/HAT1/MACROH2A1/RB1/H3-3A/GSK3A/PRMT7/SMCHD1/SPHK2/BAHD1/BAZ2A/H3C1/APOBEC1/MBD3/BRCA1/PCGF5/LMNA/AXIN1/HNRNPU/EXOSC10/KMT2D/SPTY2D1/KDM1A/POLE3/MCRS1/PPM1D/ATF7IP2/TRIM27/KDM1B/CTBP1/WDR5/TRIM28/SUV39H1/RBM15/H3C2/SIRT6/HDAC1/MSL1/H3C7/EZH2/CENPV/CHEK1/ZFP57/DOT1L/H3C6/DNMT1/SAMD1/H3C8/HELLS/LMNB2/H3C11/H3C3/LMNB1/KCNQ1
GOBP_PROTEOGLYCAN_METABOLIC_PROCESS	GOBP_PROTEOGLYCAN_METABOLIC_PROCESS	0.031322076	COL2A1/IGF1/DCN/HS3ST6/DSEL/CYTL1/HS6ST3/BMPR1B/CHST9/CHST10/CHSY3/CHST15/CHST7/BGN/HS3ST2/SULF1/CHST13/DSE/NDNF/HS3ST5/EGFLAM

GOBP_REGULATION_OF_HEMATOPOIETIC_PROGENITOR_CELL_DIFFERENTIATION	GOBP_REGULATION_OF_HEMATOPOIETIC_PROGENITOR_CELL_DIFFERENTIATION	0.031372296	ZNHIT1/KAT5/YTHDF2/SETD1A/NOTCH1/NUDT21/PUS7/PRKDC/KITLG/HSPA9/OSM/MIXL1/CDK6/FOXC1/MYB/HES5
GOBP_PORE_COMPLEX_ASSEMBLY	GOBP_PORE_COMPLEX_ASSEMBLY	0.03141783	NUP107/AFDN/NUP153/NUP98/TPR/AHCTF1/BAD/TMEM170A/NUP93/CCT8/CCT3/NUP205/GSDMD/ADAM10/PLEKHA7/NDC1
GOCC_SITE_OF_POLARIZED_GROWTH	GOCC_SITE_OF_POLARIZED_GROWTH	0.031578947	STMN2/NGFR/GPM6A/PCDH9/BOC/GAP43/DPYSL3/MAP1B/FLNA/IGF2BP1/CNR1/MAPT/APBB1/PALLD/FGF13/L1CAM/FEZ1/SNAP25/LAMP5/LRRK2/TSHZ3/NEFL/THY1/PTCH1/MAP2/PCDHGB1/FRY/CRMP1/MAP3K12/PTBP2

GOBP_POSITIVE_REGULATION_OF_INSULIN_SECRETION	GOBP_POSITIVE_REGULATION_OF_INSULIN_SECRETION	0.03172738	GCG/UCN3/NNAT/GIP/RFX6/NKX6-1/RBP4/NR1H4/ISL1/GPR27/NLGN2/BLK/GPLD1/MYRIP/GPER1
GOBP_POSITIVE_REGULATION_OF_DEFENSE_RESPONSE_TO_VIRUS_BY_HOST	GOBP_POSITIVE_REGULATION_OF_DEFENSE_RESPONSE_TO_VIRUS_BY_HOST	0.031924767	TOMM70/PARP9/HSP90AA1/APOBEC3G/DTX3L/PYCARD/ZDHHC11/PTPN22/IL23A/IL12RB1/TRAF3IP2/STAT1/CGAS/ZC3H12A/AIM2
GOBP_POSITIVE_REGULATION_OF_GLUCOSE_METABO	GOBP_POSITIVE_REGULATION_OF_GLUCOSE_METABOLIC_PROCESS	0.032096356	GCG/SORBS1/RGN/IGF1/IGF2
GOBP_NEGATIVE_REGULATION_OF_DNA_TEMPLATED_DNA_REPLICATION	GOBP_NEGATIVE_REGULATION_OF_DNA_TEMPLATED_DNA_REPLICATION	0.032367077	GMNN/DONSON/BRCA2/FBXO5/TIPIN/TIMELESS/BLM/CDT1
GOBP_ZYMOGEN_ACTIVATION	GOBP_ZYMOGEN_ACTIVATION	0.032412965	HP/APOH/FGA/GRIN2A/FGG/SERPINF2/THBS1/SERPINE2/SERPINE1/CLEC3B/C1R/THBD/FGB
GOBP_REGULATION_OF_KERATINOCYTE_PROLIFERATION	GOBP_REGULATION_OF_KERATINOCYTE_PROLIFERATION	0.032490975	REG3G/FGF10/FGF7/ZEB1/PRKD1/TGM1/KLF9/SLURP1/SNAI2/HAS2/CRNN/PTCH1

GOBP_RESPONSE_TO_PROGESTERONE	GOBP_RESPONSE_TO_PROGESTERONE	0.032622334	NKX2-2/SOX10/TACR1/CYP1B1/TGFB3/THBS1/CAV1/TGFB2/FOSB/OXTR
GOBP_MIDBODY_ABSCISSION	GOBP_MIDBODY_ABSCISSION	0.03265703	CHMP6/CHMP2B/PDCD6IP/CHMP7/CHMP1A/VPS4B/CHMP5/KIF20A/CHMP4C/CEP55
GOBP_OSTEOBLAST_PROLIFERATION	GOBP_OSTEOBLAST_PROLIFERATION	0.032663317	SFRP1/NPR3/TNN/GREM1/LRRC17/MN1/CCN1/TMEM119/NELL1/ITGB3/CTHRC1
GOBP_RETINA_DEVELOPMENT_IN_CAMERA_TYPE_EYE	GOBP_RETINA_DEVELOPMENT_IN_CAMERA_TYPE_EYE	0.032679739	NEUROD1/GPM6A/FAT3/CYP1B1/TUB/CDON/SLC17A7/SDK2/VSTM4/PRPH2/ATP2B4/LAMA1/SERPINF1/NDP/SMARCD3/CRB2/TGFB2/RHOJ/HPCA/RBP4/CLIC4/PDGFRA/THRB/CELF4/BMPR1B/THY1/PTPRM/PDGFRB/ARHGEF15/NRP1/FZD4/MERTK/PDE6B/NPHP1/SDK1/OBSL1/FJX1/NTRK2/VAX2/DLL1

GOBP_DOPAMINE_SECRETION	GOBP_DOPAMINE_SECRETION	0.0327654	SYT4/HTR2A/CNR1/CXCL12/PRKN/SYT11/SNCG/GABBR1/SYT1/CHRNB2/SYT5
GOBP_REGULATION_OF_ACTIVIN_RECEPTOR_SIGNALING_PATHWAY	GOBP_REGULATION_OF_ACTIVIN_RECEPTOR_SIGNALING_PATHWAY	0.032808399	CER1/FGF10/NODAL/DACT1/SHH/IGSF1
GOBP_REGULATION_OF_NEUROBLAST_PROLIFERATION	GOBP_REGULATION_OF_NEUROBLAST_PROLIFERATION	0.032828283	SOX10/PTN/FGF2/GLI3/CX3CR1/SMARCD3/SHH/SMO/LRRK2/ID4
GOBP_POSITIVE_REGULATION_OF_GROWTH	GOBP_POSITIVE_REGULATION_OF_GROWTH	0.032854209	SYT4/GHRH/SFRP1/AKAP6/F2/IGF1/GHR/SFRP2/MAP1B/NGF/ZFPM2/FGF2/IGFBP1/IGF2/CXCL12/WT1/NTN1/PRSS2/MAPT/RND2/GLI1/LPAR3/S100A8/TGFB2/L1CAM/S100A9/PRKN/HOPX/AVPR1A/SMO/FN1/SOX15/CRABP2/FOXS1/ISLR2/TGFBR3/MEF2C/MIR222/SYT1/ACACB/TBX5
GOBP_PLASMINOGEN_ACTIVATION	GOBP_PLASMINOGEN_ACTIVATION	0.03298153	APOH/FGA/FGG/SERPINF2/THBS1/SERPINE2/SERPINE1/CLEC3B/FGB

GOBP_RESPONSE_TO_TESTOSTERONE	GOBP_RESPONSE_TO_TESTOSTERONE	0.033184536	BAD/NCF1/XRN1/SRD5A1/HSF1/CALR/NASP/MTAP/NKX3-1/NQO1/HOXB13/GPI/HOXA10/TSPO/HOXA13/HOXA9/HOXA11/IGF1R/CA9
GOBP_AUTONOMIC_NERVOUS_SYSTEM_DEVELOPMENT	GOBP_AUTONOMIC_NERVOUS_SYSTEM_DEVELOPMENT	0.033292232	SOX10/HAND2/ASCL1/INSM1/GFRA3/PLXNA4/EDNRA/NRP2/FN1/ACKR3/ADARB1/EDNRB/NRP1/ISX/SOX11/HLX/TP63
GOBP_POSITIVE_REGULATION_OF_MUSCLE_CONTRACTION	GOBP_POSITIVE_REGULATION_OF_MUSCLE_CONTRACTION	0.033292232	SPX/TACR2/TACR1/MYOCD/CHGA/RGS2/OXTR/CCN2/PROK2/GPER1/KIT/OXT/EDN2/CHRM3/TBXA2R/GHRL/F2R
GOBP_CELL_ADHESION_MEDIATED_BY_INTEGRIN	GOBP_CELL_ADHESION_MEDIATED_BY_INTEGRIN	0.033278806	VTN/SFRP2/CYP1B1/ITGA7/JAM3/MMRN1/ITGA8/CCL21/ITGA9/ITGBL1/TGFB2/ITGA1/FBN1/ITGA5/SERPINE1/EMILIN1/CCN3/SNAI2/FOXC2/COL16A1

GOCC_SECRETORY_GRANULE_MEMBRANE	GOCC_SECRETORY_GRANULE_MEMBRANE	0.033299697	SYCN/MS4A3/SYT4/AZU1/CCDC136/CPE/NFASC/CEACAM3/RAB9B/SCG3/FRMPD3/CD36/RND2/CHRNB4/P2RX1/GP2/CAV1/SELP/CLCA1/SERPINA5/SNAP25/RAB31/KIF1A/MOXD1/PCDH7/CA4/GLIPR1/STOM/SELL/BST1/CXCR2/ITPR1/FPR1/MCEMP1/CXCR1/SPARC/SLC2A3/ATP8B4/SYT1/ITGB3/FCAR/PKP1/SYT5/PECAM1/SYNGR1/CADPS/CR1/SPACA6/FPR2/CD109/OLR1
GOMF_K63_LINKED_POLYUBIQUITIN_MODIFICATION_DEPENDENT_PROTEIN_BINDING	GOMF_K63_LINKED_POLYUBIQUITIN_MODIFICATION_DEPENDENT_PROTEIN_BINDING	0.033416664	SQSTM1/SPRTN/FAAP20/OPTN/ZBTB1/WDR81/IKBKG/ZRANB3/NPLOC4/RNF168/UIMC1/RNF169/PRPF8/ATRIP/TNIP2/IKBKE/PARP10
GOBP_NUCLEOTIDE_SALVAGE	GOBP_NUCLEOTIDE_SALVAGE	0.033333712	UPP1/AMPD1/UCK1/UCKL1/HPRT1/DGUOK/AMPD2/APRT/ADK/UCK2/FCSK/CDA/DCK/NAPRT/ADA

GOBP_RENAL_WATER_HOMEOSTASIS	GOBP_RENAL_WATER_HOMEOSTASIS	0.03339001	ADCY6/CYP4F12/MYO5B/AQP2
GOBP_TRNA_5_END_PROCESSING	GOBP_TRNA_5_END_PROCESSING	0.03339001	SSB/RPP40/RPP14/TRMT10C/RPP38/PRORP/POP5/HSD17B10/POP4/POP7/RPP25/THG1L/RPP25L/POP1
GOBP_TRANSLATIONAL_INITIATION	GOBP_TRANSLATIONAL_INITIATION	0.033416812	EIF3J/TMED2/MIF4GD/EIF1/EIF2AK3/EIF2S3/TPR/PPP1R15B/BZW1/EIF2AK1/CDC123/DENR/EIF2D/RPL13A/EIF2B5/EIF1AX/EIF3B/EIF4E2/EIF3D/RPS3/RPS5/EIF2B2/DHX33/EIF3H/MTFMT/YTHDF2/RPS3A/ABCE1/EIF3E/DNAJC3/POLR2D/EIF3I/LTO1/NCBP1/KHDRBS1/EIF4G1/COPS5/EIF3A/EIF3G/EIF4B/MTOR/EIF1AD/EIF4H/RPS17/EIF2S1/EIF4E/KLHL25/AGO2/CCL5/LARP1/MTIF2/EIF3CL/UHMK1/DDX1/EIF4EBP1/EIF2S3B/PPP1CA/GLE1/SLBP/NPM1/BZW2/RPS6KB2/EIF3C

GOMF_PEPTIDE_N_ACETYLTRANSFERASE_ACTIVITY	GOMF_PEPTIDE_N_ACETYLTRANSFERASE_ACTIVITY	0.033406613	CLOCK/KAT6B/EP300/GTF2B/ATAT1/KAT6A/NAA30/TAF9/USP22/NAA60/NAA20/NAA15/NAA80/HAT1/GTF3C4/KAT5/ESCO1/NAA25/NAA50/NAA10/SRCAP/MCM3AP/BRCA2/NAA40/KAT2A/MED24/ESCO2
GOBP_CELLULAR_RESPONSE_TO_PEPTIDE_HORMONE_STIMULUS	GOBP_CELLULAR_RESPONSE_TO_PEPTIDE_HORMONE_STIMULUS	0.03343465	PLA2G1B/AHSG/IRS4/AGTR1/ACTN2/ADIPOQ/SLC2A4/TRARG1/MIR145/GLP2R/SORBS1/GHR/MAP1B/CYP1B1/DDR2/IGFBP1/NR4A3/IGF2/AGT/CYP11A1/PDK4/PID1/NKX6-1/CAV1/EDNRA/FBN1/PRKCB/NR1H4/RAB31/GLP1R/TNS2/FOXC2/SESN3/LPL/RARRES2/KL/GPR173/INHBA/GPLD1/ITGB3/SLC30A10/SOGA1

GOBP_POSITIVE_REGULATION_OF_COLLAGEN_METABOLIC_PROCESS	GOBP_POSITIVE_REGULATION_OF_COLLAGEN_METABOLIC_PROCESS	0.033462033	F2/DDR2/SERPINF2/TGFB3/LARP6/CCN2/VIM/MIR149/INHBA/PDGFRB/ENG/F2R/RGCC/SCX
GOBP_REGULATION_OF_NATURAL_KILLER_CELL_ACTIVATION	GOBP_REGULATION_OF_NATURAL_KILLER_CELL_ACTIVATION	0.033551212	PTPN22/IL15RA/HLA-E/IL23A/DCAF15/AP1G1/KLRC2/TYK2/TICAM1/RHBDD3/IL18/HLA-F/ZNF683/JAK2
GOBP_NEGATIVE_REGULATION_OF_CALCIUM_ION_TRANSPORT	GOBP_NEGATIVE_REGULATION_OF_CALCIUM_ION_TRANSPORT	0.033613445	CASQ2/ATP1A2/PLN/GEM/PKD2/REM1/SLN/RRAD/STC1/CLIC2
GOMF_STEROL_TRANSPORTER_ACTIVITY	GOMF_STEROL_TRANSPORTER_ACTIVITY	0.03375	APOA2/APOA1/APOB/APOA4/ABCA1/MTTP/PLTP
GOBP_GLYCOSYL_COMPOUND_BIOSYNTHETIC_PROCESS	GOBP_GLYCOSYL_COMPOUND_BIOSYNTHETIC_PROCESS	0.03379183	APRT/ADK/TK2/PNP/MTAP/PGM2/DTYMK/ADA

GOBP_PROTEIN_EXIT_FROM_ENDOPLASMIC_RETICULUM	GOBP_PROTEIN_EXIT_FROM_ENDOPLASMIC_RETICULUM	0.033775944	RHBDD1/TM9SF4/ERLEC1/EDEM2/SAR1A/HERPUD1/SEC61B/TMEM30A/SORL1/DERL2/LMAN1/SAR1B/NPLOC4/UFD1/DERL1/FAF2/UBE2J1/UBAC2/OS9/HM13/SELENOS/INSIG1/AUP1/SYVN1/TMED9/PREB/YOD1/TMEM129/DERL3/SURF4/BCAP31/HSP90B1/SEC13/VCP/SVIP/SEC16A/EDEM1/TMEM30B/SLC35D3
GOBP_REGULATION_OF_INTRINSIC_APOPTOTIC_SIGNALING_PATHWAY_BY_P53_CLASS_MEDIATOR	GOBP_REGULATION_OF_INTRINSIC_APOPTOTIC_SIGNALING_PATHWAY_BY_P53_CLASS_MEDIATOR	0.033850654	RPS7/ATAD5/ING2/RRN3/MYC/HNRNPK/KDM1A/TRIAP1/RRM2B/MSX1/CD74/TP53/EIF5A/MDM2/MUC1/ELL3
GOBP_DETERMINATION_OF_ADULT_LIFESPAN	GOBP_DETERMINATION_OF_ADULT_LIFESPAN	0.033858642	MSH6/IDE/RAD54B/SIRT6/TFCP2L1/MSH2/CGAS/TP53/RAD54L
GOMF_QUATERNARY_AMMONIUM_GROUP_BINDING	GOMF_QUATERNARY_AMMONIUM_GROUP_BINDING	0.033973315	APOA2/APOA1/SERPINA5/APOA4/ABCA1/PCYT1B/CHRNB2/PLTP/OGDHL

GOBP_NECROTIC_CELL_DEATH	GOBP_NECROTIC_CELL_DEATH	0.033992583	RBCK1/ATG9A/UCN/CASP8/PELI1/HEBP2/MLKL/ALKBH7/MAP3K5/ADPRS/BAX/IRF3/IPMK/TRAF2/CASP6/FAS/PARP1/AIFM1/TLR3/ATG9B/PPIF/ITPK1/FADD/TP53/BIRC3/PGAM5/OLFM4/TSPO/FASLG
GOBP_ANATOMICAL_STRUCTURE_HOMEOSTASIS	GOBP_ANATOMICAL_STRUCTURE_HOMEOSTASIS	0.034308779	ALB/CARTPT/COL2A1/NEUROD1/PLN/SCGN/SLC8A2/DMD/CFL2/CST4/ATP1B2/TF/JAM2/PTH1R/TUB/JAM3/MAP1A/LAMA2/VSTM4/CDHR1/PDK4/CUBN/PIP/AKT3/P2RX1/CAV1/CRB2/SERPINA3/ANGPT1/RBP4/CYTL1/WWTR1/TNFRSF11B/TMEM119/COMP/IL6/PRKN/CDH23/SH3GL2/COL3A1/SMO/KRT1/ADRB2/CTSK/GJA1/CCN2/S1PR1/LOX/USH1G/HSPB1/NOX4/CLDN5/LTBP3/LAMC1/LDB2/MUC2/NANOS1/ITGB3

GOBP_PERK_MEDIATED_UNFOLDED_PROTEIN_RESPONSE	GOBP_PERK_MEDIATED_UNFOLDED_PROTEIN_RESPONSE	0.03433171	TMED2/QRICH1/EIF2AK3/PPP1R15B/HSPA5/EIF2S1/TMEM33/ABCA7/AGR2
GOBP_REGULATION_OF_LEUKOCYTE_MIGRATION	GOBP_REGULATION_OF_LEUKOCYTE_MIGRATION	0.034437283	ELANE/TACR1/THBS4/APOD/SLIT2/PTN/GREM1/JAM2/JAM3/LYVE1/CXCL12/F7/IL33/CCL21/THBS1/CCL19/IL1R1/CX3CR1/SELP/C10orf99/BDKRB1/IL6/SERPINE1/EMILIN1/CCL2/CCN3/CD200/PDGFD/DUSP1/BST1/CXCR2/RARRES2/CAMK1D/THY1/RIPOR2/ITGB3/SELE/VEGFC/CD99L2/MPP1/CCR7/IL34/TNFSF18/EDN2/FPR2/BMP5/XG/P2RY12

GOBP_REGULATION_OF_ADAPTIVE_IMMUNE_RESPONSE	GOBP_REGULATION_OF_ADAPTIVE_IMMUNE_RESPONSE	0.034671157	KLRC1/KLRD1/CD226/ATAD5/PTPN6/CYRIB/AZGP1/TNFRSF1B/TBX21/DENND1B/IL18R1/HLA-H/ADCY7/IL1B/PYCARD/TRAF2/TFRC/C4BPB/PAXIP1/HLA-E/IL23A/RIPK2/NECTIN2/IL4I1/TRIM27/HLA-DRA/HLA-B/IL12RB1/CD46/HMCES/HLA-A/TRPM4/TYK2/KMT5C/FZD5/IL27RA/MSH2/HSPD1/ZBTB7B/IL18/FOXJ1/PRKCQ/HLA-F/PVR/EPHB2/TNFRSF14/TAP2/ZC3H12A/HLA-DRB1/FADD/PARP3/NSD2/ALOX15/ADA/PRKCZ/CD55/PLA2G4A/IRF1/JAK2/TNFSF13/CD274/IL10
GOBP_MUCOPOLYSACCHARIDE_METABOLIC_PROCESS	GOBP_MUCOPOLYSACCHARIDE_METABOLIC_PROCESS	0.034753363	ITIH2/HAS1/FGF2/DCN/LYVE1/ITIH3/ITIH5/DSEL/EDNRA/ANGPT1/HAS2/CHST9/CHSY3/CHST15/CHST7/ST3GAL6/BGN/CHST13/DSE/NDNF/EDNRB/EGFLAM

GOCC_BASOLATERAL_PLASMA_MEMBRANE	GOCC_BASOLATERAL_PLASMA_MEMBRANE	0.034780233	FOLR1/CXADR/CDH17/CASK/ANK3/TGFA/TFRC/SLC26A1/DAG1/EGFR/LIN7C/STK39/SLC4A2/CLDN7/EPB41/SLC3A2/ERBB3/EPCAM/SLC6A9/ATP1A1/SLC16A3/SLC9A4/ITGA3/ATP7B/ABCC3/SLC12A2/ADRA2A/SLC26A6/BSG/SLC7A1/SLC13A3/SCRIB/ANXA2/SLC16A8/SLC29A2/SLC16A1/SLC2A1/SLC39A14/ATP2C2/SLC7A5/ST14/MAP7/SLC22A1/MYO1A/SLC4A4/EPPK1/ANK1/SLC19A1/DLG3/CD38/LDLR/ATP1B1/EZR/VSIG1/SLC9A1/ATP12A/SLC4A11/CALHM3/CNNM4/PROM2/KCNQ1/CDH16/SLCO1B3/CA9/CLDN8/AQP2
GOMF_SIALIC_ACID_BINDING	GOMF_SIALIC_ACID_BINDING	0.034867503	ADIPOQ/SIGLEC6/SELP/FCN1/SELE
GOBP_VASCULAR_ENDOTHELIAL_GROWTH_FACTOR_RECEPTOR_SIGNALING_PATHWAY	GOBP_VASCULAR_ENDOTHELIAL_GROWTH_FACTOR_RECEPTOR_SIGNALING_PATHWAY	0.034939759	VTN/FGF10/PRKD1/ITGA5/PRKCB/EMILIN1/FOXC2/HSPB1/MIR10B/ITGB3/MMRN2/VEGFC/SULF1/CLEC14A/AXL/NRP1/FZD4/TMEM204

GOBP_REGULATION_OF_FILOPODIUM_ASSEMBLY	GOBP_REGULATION_OF_FILOPODIUM_ASSEMBLY	0.034981906	GPM6A/GAP43/DPYSL3/DAAM2/NLGN1/CCL21/TGFB3/TENM1/PALM/NEURL1/DOCK11/RIPOR2/CCR7/RHOQ/NRP1
GOCC_CELL_SUBSTRATE_JUNCTION	GOCC_CELL_SUBSTRATE_JUNCTION	0.034965035	CNN1/SYNPO2/ACTN2/PGM5/FLNC/FHL1/SORBS1/FLRT2/TNS1/DMD/NEXN/SMPX/NFASC/FERMT2/LIMS2/CDH2/FLNA/DDR2/SVIL/TNC/CSRP1/PHLDB2/HMCN1/DIXDC1/TGFB1I1/LAYN/ACTC1/ITGA8/AKAP12/ITGBL1/CAV1/CSPG4/LPP/PALLD/ITGA1/L1CAM/ITGA5/EFS/FLRT1/FAM107A/DAB2/MCAM/CSRP2/DLC1/TNS2/PARVA/GJA1/SORBS2/VIM/PDLIM7/TSPAN9/ILK/FAP/RHOB/HSPB1/NOX4/TEK/ACTN1/THY1/HSPA1A/DST/WASF1/MRC2/SCARF2/ANXA6/CORO2B/ITGB3/CD99L2/ADGRB1/ENAH/APBB1IP/ITGA11/NME2/PDGFRB/LRP1/SHROOM4/GSN/ARHGAP24/NRP1/RDX/FBLN7/RRAS/CNN3/ARHGAP31
GOBP_P_BODY_ASSEMBLY	GOBP_P_BODY_ASSEMBLY	0.035008336	NOCT/DYNC1H1/EDC3/LSM3/CNOT6L/PAN2/CNOT7/CNOT6/PATL1/CNOT1/LIMD1/LSM4

GOBP_SLEEP	GOBP_SLEEP	0.035040431	GHRH/GRIN2A/HTR2A/OXTR/PTGDS/CHRNB2/PTGDR/OXT/CSF2/GHRL
GOBP_REGULATION_OF_FATTY_ACID_BIOSYNTHETIC_PROCESS	GOBP_REGULATION_OF_FATTY_ACID_BIOSYNTHETIC_PROCESS	0.035131744	APOA1/APOC3/RGN/GIP/PDK4/APOA4/AVPR1A
GOCC_CELL_LEADING_EDGE	GOCC_CELL_LEADING_EDGE	0.035140562	ACTG2/STMN2/KCNB1/MYLK/PSD/CTNNA3/ACTA2/ARHGEF26/KLHL41/STON1/FERMT2/DPYSL3/CDH2/IGF2BP1/PHLDB2/ACTA1/LAYN/AMPH/WASF3/PDE9A/TPM1/ACTC1/ITGA8/MAPT/APBB1/BMX/CSPG4/PALLD/RASGRP2/PDLIM4/HPCA/ITGA5/PKD2/MYH10/MCC/FAM107A/PTPN13/LAMP5/SGCE/ANTXR1/DLC1/MPP2/PARVA/SORBS2/VIM/PDLIM7/EPB41L3/PLEKHO1/RAB34/FGD1/ILK/FAP/PLEKHH2/PDPN/ACTN1/THY1/MTSS1/DST/WASF1/S100B/LDB2/PTPRM/GABBR1/RIPOR2/ITGB3/CXCR4/ENAH/APBB1IP/GPER1/MYADM/NME2/GABRE
GOBP_REGULATION_OF_CELL_MORPHOGENESIS_INVOLVED_IN_DIFFERENTIATION	GOBP_REGULATION_OF_CELL_MORPHOGENESIS_INVOLVED_IN_DIFFERENTIATION	0.035164835	APOA1/RELN/MYOC/FGA/FGG/FERMT2/LIMS2/FBLN1/FLNA/PAK3/PTPRD/DAB2/ILK/HAS2/PDPN/FGB

GOBP_RESPONSE_TO_IMMOBILIZAT	GOBP_RESPONSE_TO_IMMOBILIZATION_STRE	0.035422343	UCN3/TPH1/SLC8A1
GOBP_RESPONSE_TO_OXYGEN_RADICAL	GOBP_RESPONSE_TO_OXYGEN_RADICAL	0.035386632	MPO/FBLN5/CD36/APOA4/SOD3
GOBP_CELLULAR_RESPONSE_TO_VITAMIN	GOBP_CELLULAR_RESPONSE_TO_VITAMIN	0.035433071	PENK/SFRP1/CYP24A1/MN1/KANK2/FOLR2/SNAI2
GOBP_POSITIVE_REGULATION_OF_MITOCHONDRIAL_TRANSLATION	GOBP_POSITIVE_REGULATION_OF_MITOCHONDRIAL_TRANSLATION	0.035460668	METTL8/NGRN/CDK5RAP1/FASTKD3/TRMT10C/UQCC2/FASTKD2/RMND1/RCC1L/RPUSD3/TRUB2/MPV17L2/RPUSD4/MRPS27/C1QBP
GOMF_NADPH_BINDING	GOMF_NADPH_BINDING	0.035460668	QDPR/SRD5A1/CBR4/NDOR1/GRHPR/DUS2/DECR1/DHFRP1/DHFR/HMGCR
GOBP_HISTONE_EXCHANGE	GOBP_HISTONE_EXCHANGE	0.035460668	ZNHIT1/ACTL6A/KAT5/ACTR6/RUVBL1/NASP/RUVBL2/SPTY2D1/SRCAP/ANP32E
GOCC_STEREOCILIUM_BUNDLE	GOCC_STEREOCILIUM_BUNDLE	0.035476864	SLC9A3R1/ADCY6/USH1C/ATP8B1/TPRN/FSCN2/PLS1/MYO3B/IDO1/CALB1

GOBP_POSITIVE_REGULATION_OF_MITOCHONDRION_ORGANIZATION	GOBP_POSITIVE_REGULATION_OF_MITOCHONDRION_ORGANIZATION	0.035459907	MIEF2/FAM162A/SSBP1/MMP9/ZDHHC6/AMBRA1/MUL1/PLD6/FBXO7/RALBP1/PARK7/BID/RALA/BAD/MARCHF5/GSK3A/PLAUR/MIEF1/PLA2G6/BAX/SLC25A5/PYCARD/ATP5IF1/ZNF205/VDAC1/TNFSF10/HTT/AURKA/STOML2/HIP1R/PPIF/CHCHD10/PMAIP1/TP53/PGAM5/ENDOG/BIK/BAK1/BBC3/MCU/GZMB
GOMF_GENERAL_TRANSCRIPTION_INITIATION_FACTOR_ACTIVITY	GOMF_GENERAL_TRANSCRIPTION_INITIATION_FACTOR_ACTIVITY	0.035496988	TBPL1/CCNH/TAF1/GTF2E1/TBP/TAF13/TAF4/GTF2B/GTF2A1/TAF9/UBTF/TAF12/TAF2/DR1/GTF3C1/GTF2H2/GTF3C3/DRAP1/GTF3C4/TAF1C/GTF3C2/TAF6L/TAF4B/RRN3/GTF3C6/GTF2H3/GTF2F1/BRF1/TAF5/GTF3C5/SNAPC4/GTF2E2/GTF2A2

GOBP_PROTEIN_IMPORT_INTO_MITOCHONDRIAL_MATRIX	GOBP_PROTEIN_IMPORT_INTO_MITOCHONDRIAL_MATRIX	0.035524704	TOMM40L/PAM16/TIMM17B/DNAJC15/TIMM23/TIMM23B/TIMM17A/TIMM50/TOMM70/GRPEL2/TIMM21/GRPEL1/TOMM40/TIMM44
GOBP_REGULATION_OF_POSITIVE_CHEMOTAXIS	GOBP_REGULATION_OF_POSITIVE_CHEMOTAXIS	0.035573123	AZU1/FGF10/CXCL12/F7/NTF3/S1PR1/SCG2/VEGFC
GOBP_TYPE_B_PANCREATIC_CELL_DEVELOPMENT	GOBP_TYPE_B_PANCREATIC_CELL_DEVELOPMENT	0.035573123	NKX2-2/INSM1/CDH2/NKX6-1/SMO
GOBP_POSITIVE_REGULATION_OF_ORGANELLE_ASSEMBLY	GOBP_POSITIVE_REGULATION_OF_ORGANELLE_ASSEMBLY	0.035585965	MARK4/DYNC1H1/ATMIN/G3BP2/CCP110/CROCC/RHOA/PIP4K2C/CEP135/MAPK9/SNX7/SEPTIN9/CNOT6L/PAN2/NUP62/PDCD6IP/HAP1/CEP295/MNS1/PPP1R35/VPS4B/TAPT1/SRC/GPSM2/G3BP1/CNOT6/SASS6/ATG2A/STX18/ENTR1/ARHGEF5/CNOT1/SPAG5/HTT/WRAP73/PLK4/ELAPOR1/MAPK15/SDC1/STIL

GOBP_OUTFLOW_TRACT_SEPTUM_MORPHOGENESIS	GOBP_OUTFLOW_TRACT_SEPTUM_MORPHOGENESIS	0.035620053	NKX2-5/ZFPM2/TGFB2/NRP2/ISL1/PARVA/ROBO1/ACVR1/SEMA3C/NRP1/FGFR2/ENG
GOBP_RESPONSE_TO_ACTIVITY	GOBP_RESPONSE_TO_ACTIVITY	0.035714286	GCG/ADIPOQ/FNDC5/PRKAA2/ZEB1/AGT/MMP2/IL6/TNS2/RYR2/KL/ITGB3/CXCR4/OXT/COL4A2/ANGPT2/BMP6
GOBP_POSITIVE_REGULATION_OF_CD4_POSITIVE_ALPHA_BETA_T_CELL_ACTIVATION	GOBP_POSITIVE_REGULATION_OF_CD4_POSITIVE_ALPHA_BETA_T_CELL_ACTIVATION	0.035720771	SHB/IL23A/RIPK2/KLHL25/HLA-DRA/IL12RB1/SOCS1/ZBTB7B/IL18/PRKCQ/HLA-DRB1/LGALS9/PRKCZ/CD55/IFNG/MYB
GOBP_REGULATION_OF_MITOCHONDRIAL_OUTER_MEMBRANE_PERMEABILIZATION_INVOLVED_IN_APOPTOTIC_SIGNALING_PATHWAY	GOBP_REGULATION_OF_MITOCHONDRIAL_OUTER_MEMBRANE_PERMEABILIZATION_INVOLVED_IN_APOPTOTIC_SIGNALING_PATHWAY	0.035869197	SLC35F6/GSK3A/SLC25A5/ATP5IF1/ZNF205/HIP1R/CHCHD10/BAK1/TMEM102/BBC3/GZMB

GOBP_DENDRITE_DEVELOPMENT	GOBP_DENDRITE_DEVELOPMENT	0.03600823	RELN/CHRNA3/FAT3/SEZ6/ADGRB3/ZDHHC15/MAP1B/MAP6/PAK3/PTN/IGF2BP1/DAB1/MAP1A/NLGN1/PTPRD/NTN1/PRKG1/DGKG/NFATC4/VLDLR/MARK1/LPAR1/FLRT1/PREX2/KIF1A/SDC2/LRRK2/DPYSL5/CTNND2/DLG4/NEURL1/KLF7/CAMK1D/MEF2C/NLGN2/PHACTR1/DCLK1/CHRNB2/MAP2/ARC/NRP1/LZTS1/BMP5/DCDC2/NEDD4/GPR37/GRIP1/DISC1/GHRL/SDK1/OBSL1/BCL11A/FYN/LRP4/ASAP1
GOBP_PROSTATE_GLAND_MORPHOGENESIS	GOBP_PROSTATE_GLAND_MORPHOGENESIS	0.036036036	SFRP1/FGF10/AR/NOG/TNC/CYP7B1/MMP2/ESR1/SHH/ID4
GOMF_OXIDOREDUCTASE_ACTIVITY_ACTING_ON_PEROXIDE_AS_ACCEPTOR	GOMF_OXIDOREDUCTASE_ACTIVITY_ACTING_ON_PEROXIDE_AS_ACCEPTOR	0.036101083	MPO/HBB/GPX3/TPO/GPX7/SESN3/PTGS1/CLIC2/GPX8/LTC4S/HBA2/GSTM2/CYGB/PXDN

GOMF_LYSOPHOSPHOLIPID_ACYLTRANSFERASE_ACTIVITY	GOMF_LYSOPHOSPHOLIPID_ACYLTRANSFERASE_ACTIVITY	0.036185726	LCLAT1/LPCAT3/MBOAT7/GPAT3/AGPAT3/GPAT4/CRLS1/MBOAT2/LPCAT1/MBOAT1/AGPAT2/AGPAT5/LPCAT4
GOBP_REGULATION_OF_RAC_PROTEIN_SIGNAL_TRANSDUCTION	GOBP_REGULATION_OF_RAC_PROTEIN_SIGNAL_TRANSDUCTION	0.036185726	KRAS/PIK3CB/ARF6/SSX2IP/GABARAP/ARHGAP44/SH3BP1/DNM2/RASGRF1
GOBP_MONOCARBOXYLIC_ACID_BIOSYNTHETIC_PROCESS	GOBP_MONOCARBOXYLIC_ACID_BIOSYNTHETIC_PROCESS	0.036344756	PLA2G1B/APOA1/PLP1/APOC3/PTGIS/CES1/ALDH1A2/RGN/ELOVL2/PRKAA2/GIP/ELOVL4/PDK4/HACD4/HACD1/CYP7B1/GAMT/NR1H4/APOA4/AVPR1A/FADS6/MALRD1/CRABP2/CYP39A1/PTGDS/LPL/PTGS1/QKI/CYP27A1/ALDH1A3/CYP3A4/ACACB/CYP1A1/RBP1/ELOVL5/LTC4S/FADS2/EDN2/ACMSD/THNSL2/GSTM2/LIPC

GOBP_REGULATION_OF_NEUTROPHIL_CHEMOTAXIS	GOBP_REGULATION_OF_NEUTROPHIL_CHEMOTAXIS	0.036432161	THBS4/SLIT2/JAM3/CCL21/CCL19/BST1/CXCR2/CAMK1D/RIPOR2/MPP1/CCR7
GOMF_ENDONUCLEASE_ACTIVITY	GOMF_ENDONUCLEASE_ACTIVITY	0.036417887	TSN/PMS2/RPS3/CPSF3/LAS1L/DIS3/RIDA/NUDT16L1/KHNYN/PPP1R8/TSEN34/TSEN2/FAN1/POP4/DNASE2/XRCC1/PIWIL4/APEX2/RCL1/INTS11/SND1/G3BP1/EME1/MUS81/POP7/ERCC5/NTHL1/REXO4/RPP25/AGO2/YBEY/NUDT12/XRCC3/ELAC2/ZGRF1/RBBP8/MRPL44/GEN1/NOB1/EME2/ZC3H12A/DNASE1L2/POP1/EXO1/DFFB/FEN1/DNA2/ENDOG/PIWIL1/RNASE7/ERN2
GOBP_REGULATION_OF_ORGANIC_ACID_TRANSPORT	GOBP_REGULATION_OF_ORGANIC_ACID_TRANSPORT	0.036513545	SYT4/ATP1A2/SLC38A3/RGS4/AGT/SV2A/THBS1/RGS2/FABP3/AVPR1A

GOBP_NEGATIVE_REGULATION_OF_PROTEIN_MATURATION	GOBP_NEGATIVE_REGULATION_OF_PROTEIN_MATURATION	0.036697248	NLRP7/GAS1/SERPINF2/THBS1/SERPINE2/SERPINE1/LRRK2/PRNP/MAGEA3/CARD18/PLAT/SIRT4/CST7
GOBP_DIOL_METABOLIC_PROCESS	GOBP_DIOL_METABOLIC_PROCESS	0.036689778	GCH1/PCBD1/SPTLC2/ABCA2/DHFRP1/SPR/DHFR/SPTSSB/PLPP2/SGPP2/ACER2/DEGS2
GOBP_T_CELL_MEDIATED_IMMUNITY	GOBP_T_CELL_MEDIATED_IMMUNITY	0.036819833	KLRC1/KLRD1/CTSC/CYRIB/AZGP1/TNFRSF1B/TBX21/BTN3A3/MYO1G/DENND1B/IL18R1/HLA-H/IL1B/TRAF2/HLA-E/IL23A/NECTIN2/IL4I1/BTN3A2/HLA-DRA/HLA-B/IL12RB1/CD46/HLA-A/TRPM4/CD8A/FZD5/JAG1/HSPD1/IL18/HLA-F/PVR/TAP2/HLA-DRB1/FADD/ICAM1/PRKCZ/SCART1/CD55/PRF1/CD70

GOBP_LATE_ENDOSOME_TO_VACUOLE_TRANSPORT	GOBP_LATE_ENDOSOME_TO_VACUOLE_TRANSPORT	0.036847655	STAM2/BECN1/SNF8/STAM/CHMP2A/TMEM50B/VPS25/TMEM50A/VPS36/VPS37A/VPS37D/VPS4A/UBAP1/MVB12A/VPS37C/CHMP6/VTA1/VPS28/CHMP2B/LEPROTL1/CHMP7/CHMP1A/VPS4B/HGS/CHMP5/PTPN23/VPS37B/CHMP4C
GOBP_POSITIVE_REGULATION_OF_NEUROBLAST_PROLIFERATION	GOBP_POSITIVE_REGULATION_OF_NEUROBLAST_PROLIFERATION	0.036939314	SOX10/FGF2/GLI3/CX3CR1/SMARCD3/SHH/SMO/ID4
GOBP_MITOTIC_RECOMBINATION	GOBP_MITOTIC_RECOMBINATION	0.037245559	TEP1/RAD50/RAD51D/MSH3/ERCC2/SMC5/TERF2/XRCC1/RAD54B/BRCA2/ZSCAN4/XRCC3/MSH2/GEN1/DMC1/RAD51
GOBP_MITOTIC_CYTOKINETIC_PROCESS	GOBP_MITOTIC_CYTOKINETIC_PROCESS	0.037248121	RHOA/VPS4A/ARF1/CHMP6/CHMP2B/NUP62/PDCD6IP/CHMP7/CHMP1A/VPS4B/CHMP5/KIF20A/KIF20B/CNTROB/CHMP4C/CEP55

GOBP_CELL_PROLIFERATION_INVOLVED_IN_KIDNEY_DEVELOPMENT	GOBP_CELL_PROLIFERATION_INVOLVED_IN_KIDNEY_DEVELOPMENT	0.037234043	GPC3/WT1/SHH/OSR1/PDGFD/PTCH1/ITGB3/PDGFRB
GOBP_RESPONSE_TO_PROSTAGLANDIN_E	GOBP_RESPONSE_TO_PROSTAGLANDIN_E	0.037234043	SFRP1/PRKAA2/CCL21/CCL19/A2M/TGFBR3/CCR7/OXT
GOBP_REGULATION_OF_TRANSCRIPTION_REGULATORY_REGION_DNA_B	GOBP_REGULATION_OF_TRANSCRIPTION_REGULATORY_REGION_DNA_BINDING	0.037259615	HAND1/HAND2/NEUROD1/IGF1/TRIM6
GOBP_CELLULAR_RESPONSE_TO_KETONE	GOBP_CELLULAR_RESPONSE_TO_KETONE	0.037280702	SOX10/SFRP1/ADCY5/AR/UGT3A2/ADCY2/PTGFR/CYP1B1/PRKAA2/ADCY1/FBXO32/SERPINF1/KLF9/MIR342
GOBP_APOPTOTIC_PROCESS_INVOLVED_IN_DEVELOPMENT	GOBP_APOPTOTIC_PROCESS_INVOLVED_IN_DEVELOPMENT	0.037453184	HAND2/NKX2-5/CRYAB/SLIT2/SLIT3/CCN1/TGFB2/FOXC2

GOBP_ATP_METABOLIC_PROCESS	GOBP_ATP_METABOLIC_PROCESS	0.03764297	OGDH/GALK1/NDUFB5/NDUFC1/EIF6/NDUFA1/DHTKD1/APP/PGAM4/ENPP3/SLC2A6/NDUFB4/ATP5MG/NDUFS8/TIGAR/PRKAG1/NDUFB2/ATP5MF/DGUOK/NDUFA8/ATP6V1A/PSEN1/NUDT5/ATP5F1C/NDUFA6/NDUFB10/ATP5PD/NDUFS5/ENO2/NDUFB3/NDUFA12/NDUFA5/FOXK2/RPTOR/ATP6V1B2/FOXK1/GPD1/NDUFA9/NDUFB6/ZBTB7A/BAD/HSPA8/NDUFA11/PARG/NDUFS6/NDUFA10/NDUFS2/FIGNL1/ATP5F1EP2/HKDC1/SPHK2/NDUFA2/ADPGK/NDUFS1/NDUFS3/SDHD/CLPX/NDUFB7/ATP5PO/NDUFAB1/HK3/MTOR/ATP5MC1/MLST8/ATP5PB/NDUFA13/NMNAT1/LDHA/PARP1/NUDT2/UQCC3/AK1/ATP5IF1/SDHB/AK2/OLA1/PGK1/ATP5MC3/GIT1/ATP5F1A/VCP/PFKL/NADK/TPI1/ANTKMT/SDHA/FBP1/SIRT6/ATP5F1B/STOML2/PGAM1[illegible]
GOBP_NUCLEAR_MEMBRANE_REASSEMBLY	GOBP_NUCLEAR_MEMBRANE_REASSEMBLY	0.037713723	ANKLE2/SPAST/BANF1/UBE2I/VPS4A/NSFL1C/UBXN2B/CHMP6/CHMP2B/CHMP7/CHMP1A/VPS4B/CHMP5/CHMP4C/REEP4/RCC1
GOBP_PHASIC_SMOOTH_MUSCLE_CONTRACTION	GOBP_PHASIC_SMOOTH_MUSCLE_CONTRACTION	0.037685061	TACR2/HTR2B/P2RX2/PTGER3/SSTR2/KIT/EDNRB/EDN2/GHRL

GOBP_CENTRAL_NERVOUS_SYSTEM_NEURON_DIFFERENTIATION	GOBP_CENTRAL_NERVOUS_SYSTEM_NEURON_DIFFERENTIATION	0.037715517	NKX2-2/ASCL1/SLIT2/NPY/DCLK2/PLXNA4/HOXD10/GLI3/SEMA3E/MAPT/NKX6-1/NRP2/CDH11/SHH/SPOCK1/SMO/ISL1/GDF7/ID4/LHX6/BMPR1B/INHBA/PTCH1/DYNC2H1/DCLK1/CHRNB2/MAP2/CHD5/ADARB1/GLI2/NDNF/NRP1/SCN1B/FGFR2/RORA/NOVA2
GOBP_SENSORY_PERCEPTION_OF_BITTER_TASTE	GOBP_SENSORY_PERCEPTION_OF_BITTER_TASTE	0.037868163	CST4/REEP2/CA6/PIP/CST2
GOBP_POSITIVE_REGULATION_OF_ENDOTHELIAL_CELL_MIGRATION	GOBP_POSITIVE_REGULATION_OF_ENDOTHELIAL_CELL_MIGRATION	0.038043478	SMOC2/FGF2/PRKD1/AGT/AKT3/THBS1/ATOH8/FGFR1/ANGPT1/RHOJ/NRP2/MIR342/CCBE1/MIR27B/FOXC2/FGF1/ADGRA2/SRPX2/RHOB/HSPB1/TEK/SPARC/MIR10B/GPLD1/ITGB3/VEGFC/PLPP3/NRP1/RRAS

GOBP_LIMBIC_SYSTEM_DEVELOPMENT	GOBP_LIMBIC_SYSTEM_DEVELOPMENT	0.038202247	KCNA1/RELN/ATP1A2/NEUROD1/SCT/DCLK2/DAB1/GLI3/SEMA3E/ATP2B4/FGF13/NRP2/FEZ1/SMO/ID4/TMEM108/NEFL
GOCC_INTEGRAL_COMPONENT_OF_SYNAPTIC_VESICLE_MEMBRANE	GOCC_INTEGRAL_COMPONENT_OF_SYNAPTIC_VESICLE_MEMBRANE	0.038359788	SYT4/ATP6V1G2/SLC17A7/SV2A/SYT1/SYP/SYNGR1
GOBP_COLLATERAL_SPROUTING	GOBP_COLLATERAL_SPROUTING	0.038359788	EPHA7/NGF/RND2/LPAR3/FGF13/SPART/CRABP2
GOBP_MODULATION_OF_PROCESS_OF_ANOTHER_ORGANISM	GOBP_MODULATION_OF_PROCESS_OF_ANOTHER_ORGANISM	0.038359788	REG3G/DEFA4/REG1B/DEFA6/S100A9
GOBP_NCRNA_CATABOLIC_PROCESS	GOBP_NCRNA_CATABOLIC_PROCESS	0.038312677	ZCCHC7/TENT4B/DIS3L2/SLFN13/TENT2/EXOSC8/PARN/DIS3L/DIS3/ZC3H4/NUDT16L1/MTREX/XRN1/ZCCHC8/MIAT/DEDD2/NSUN2/EXOSC10/SND1/EXOSC2/EXOSC7/EXOSC5/GAS5/DDX49/EXOSC9/EXOSC4/ELAC2/ZC3H12A/POP1/ERN2

GOBP_RESPONSE_TO_OXYGEN_LEVELS	GOBP_RESPONSE_TO_OXYGEN_LEVELS	0.038306452	UCN3/PENK/SFRP1/ADIPOQ/KCNMA1/SLC2A4/KCNK3/MYOCD/KCNMB1/MIR145/PTGIS/CRYAB/KCNA5/KCNK2/DDR2/P2RX2/CXCL12/GUCY1A1/F7/HIF3A/TGFB3/THBS1/GUCY1B1/CAV1/EDNRA/LPAR1/CLCA1/TGFB2/SLC8A1/MMP2/SOD3/PLOD2/OXTR/GUCY1A2/CCN2/KCND2/PDPN/ITPR1/RYR2/STC1/TGFBR3/TWIST1/VEGFC/CYP1A1/CHRNB2/GNGT1/VASN/CXCR4/CD34/MB/COL1A1/PDGFRB/STC2/WTIP/NDNF
GOBP_REGULATION_OF_CALCIUM_ION_IMPORT	GOBP_REGULATION_OF_CALCIUM_ION_IMPORT	0.038610039	GCG/PLN/CXCL12/PKD2/CCL2/SLN/STC1
GOBP_REGULATION_OF_TRIGLYCERIDE_METABOLIC_	GOBP_REGULATION_OF_TRIGLYCERIDE_METABOLIC_PROCESS	0.038701623	APOA1/APOC3/RGN/THRSP/APOA4
GOBP_MEMBRANE_LIPID_CATABOLIC_PROCESS	GOBP_MEMBRANE_LIPID_CATABOLIC_PROCESS	0.038701623	CEL/PNLIPRP2/LCT/CYP1B1

GOBP_VESICLE_TARGETING_TO_FROM_OR_WITHIN_GOLGI	GOBP_VESICLE_TARGETING_TO_FROM_OR_WITHIN_GOLGI	0.038826981	TRAPPC8/TRAPPC2/TRAPPC1/PPP6C/SAR1A/TMED10/SAR1B/TFG/CSNK1D/KLHL12/TRAPPC12/TMED2/CUL3/GBF1/TRAPPC4/TRAPPC11/ARFGAP2/ARFGAP3/TRAPPC2L/PDCD6/TMED9/PREB/AP1AR/SEC16A/MAPK15
GOBP_SEX_DIFFERENTIATION	GOBP_SEX_DIFFERENTIATION	0.038894575	AFP/SFRP1/ADCYAP1R1/FGF10/AMHR2/GFRA1/SLIT2/PGR/PTPRN/AR/SFRP2/SLIT3/ROR2/CYP1B1/ZFPM2/TEX11/WT1/CDKL2/PTX3/GREB1L/ADAMTS1/ZFP42/TGFB2/RBP4/MMP2/ESR1/MMP19/SHH/PDGFRA/OSR1/DHH/PBX1/CGA/FOXF2/A2M/GDF9/RETN/SOX15/WNT2B/TCF21

GOCC_SAGA_COMPLEX	GOCC_SAGA_COMPLEX	0.038934869	TAF9/TAF12/TADA3/TRRAP/TAF5L/USP22/TADA1/TADA2B/TAF6L/ATXN7L3/KAT2A/SF3B5/SF3B3
GOBP_NEURON_PROJECTION_EXTENSION_INVOLVED_IN_NEURON_PROJECTION_GUIDANCE	GOBP_NEURON_PROJECTION_EXTENSION_INVOLVED_IN_NEURON_PROJECTION_GUIDANCE	0.038944724	SLIT2/SLIT3/PLXNA4/CXCL12/SEMA3E/NRP2/SEMA6A/SEMA6D/SEMA6C/SEMA3G/SEMA5B/SEMA3D/SEMA3C/NRP1
GOBP_FEEDING_BEHAVIOR	GOBP_FEEDING_BEHAVIOR	0.038990826	GCG/CARTPT/HAND2/TACR1/NEGR1/NPY/CNR1/NR4A3/AGT/LEPR/SGIP1/RETN/UCHL1/OXTR
GOBP_REGULATION_OF_TRANSCRIPTION_OF_NUCLEOLAR_LARGE_RRNA_BY_RNA_POLYMERASE_I	GOBP_REGULATION_OF_TRANSCRIPTION_OF_NUCLEOLAR_LARGE_RRNA_BY_RNA_POLYMERASE_I	0.039080136	MACROH2A1/SMARCB1/PIH1D1/NCL/MTOR/NOL11/IPPK/MARS1/DDX11/SIRT7/SMARCA4
GOBP_REGULATION_OF_SYNCYTIUM_FORMATION_BY_PLASMA_MEMBRAN	GOBP_REGULATION_OF_SYNCYTIUM_FORMATION_BY_PLASMA_MEMBRANE_FUSION	0.039095094	ADAM9/GDF15/CXCL9/CXCL10/SCGB3A1
GOBP_NEGATIVE_REGULATION_OF_CELL_JUNCTION_ASSEMBLY	GOBP_NEGATIVE_REGULATION_OF_CELL_JUNCTION_ASSEMBLY	0.039190898	EPHA7/APOD/PHLDB2/THBS1/FAM107A/DKK1/DLC1

GOMF_SODIUM_CHANNEL_ACTIVITY	GOMF_SODIUM_CHANNEL_ACTIVITY	0.039190898	SCN7A/GRIK3/GRIK1/GRIK5/CACNA1H/SCN4B/SCN3A/PKD2
GOBP_NEGATIVE_REGULATION_OF_PROTEIN_IMPORT_INTO_NUCLEUS	GOBP_NEGATIVE_REGULATION_OF_PROTEIN_IMPORT_INTO_NUCLEUS	0.039130435	APOD/CD36/RAB23/ANGPT1/PKIA/PKIG
GOMF_LIPASE_INHIBITOR_ACTIVITY	GOMF_LIPASE_INHIBITOR_ACTIVITY	0.039130435	APOA2/APOC3/SCGB1A1/PINLYP
GOBP_TAIL_ANCHORED_MEMBRANE_PROTEIN_INSERTION_INTO_ER_MEMBRANE	GOBP_TAIL_ANCHORED_MEMBRANE_PROTEIN_INSERTION_INTO_ER_MEMBRANE	0.039107945	EMC10/EMC2/EMC3/EMC4/MMGT1/EMC7/BAG6/UBL4A/EMC9/GET4/EMC1/EMC8/GET3/SGTA/EMC6
GOMF_DISULFIDE_OXIDOREDUCTASE_ACTIVITY	GOMF_DISULFIDE_OXIDOREDUCTASE_ACTIVITY	0.039058447	GLRX3/PDIA3/CHCHD4/TXNDC17/TXNRD1/PDIA6/QSOX1/TXN/PDIA4/QSOX2/TMX2/TXN2/TMX1/GFER/TXNDC5/PGK1/TXNDC12/P4HB/ERO1A/GSTO2/GSR/SCO2/PDIA2
GOBP_HISTONE_H3_K4_TRIMETHYLATION	GOBP_HISTONE_H3_K4_TRIMETHYLATION	0.039197271	BEND3/WDR61/WDR82/RTF1/OGT/SETD1B/ZNF335/NFYA/NFYC/CTR9/SETD1A/TET2/TET3/KMT2D/H1-2/H1-3/H1-4

GOCC_PROTEIN_KINASE_COMPLEX	GOCC_PROTEIN_KINASE_COMPLEX	0.039171814	ERN1/DAPK1/CSNK2A1/DBF4B/PHKB/ERCC3/SMCR8/CDK13/CDKN2D/ATG13/CSNK2A2/CCNT2/PRKAG1/ACVR1B/CDK2/ERCC2/XRCC6/CDK12/GTF2H2/BCCIP/CCNQ/CCNT1/GTF2H2C/PHKG2/CDK7/CCND3/IKBKB/CCNK/CDK5R1/LAS1L/XRCC5/CCNC/MMS19/CDK16/CCNJ/CNPPD1/MAP3K5/ACVR1C/CCNI2/STK11/PYCARD/CKS1B/SESN2/GTF2H3/CCNG1/CHUK/DBF4/PRKY/PRKDC/IKBKE/CDK5/PRKCI/ATG101/PRKX/PRKAR1B/PARD6A/CKS2/CDK1/CDK6/CCNB2/PCNA/CCNB1/CCNP/CCNA2/CCND1/CCNF/CCNE2/CCNO/TRIM40/IGF1R
GOBP_CELL_AGGREGATION	GOBP_CELL_AGGREGATION	0.039270687	COL2A1/MPZ/MGP/SOX5/CCN2/BMPR1B
GOBP_NODAL_SIGNALING_PATHWAY	GOBP_NODAL_SIGNALING_PATHWAY	0.039270687	CER1/NODAL/DACT1/SHH

GOBP_PHAGOCYTOSIS	GOBP_PHAGOCYTOSIS	0.039354188	ELANE/PRTN3/AZU1/APOA2/AHSG/APOA1/TAFA4/ADIPOQ/SRPX/PLA2G5/COLEC12/TUB/PTX3/LEPR/CD36/COLEC11/THBS1/PIP4P2/C4A/FCN1/C4B/IGHV1-69D/CD302/CCL2/RAB31/SYT11/HAVCR1/IGHV1-69-2/IGHG4/RAB34/TIMD4/ABCA1/IGHV6-1/CAMK1D/IGHV7-4-1/FCGR2B/ITGB3/ELMO1/ADGRB1/LRP1/RAB39A/PECAM1/GSN/AXL/IGHV3-43/MERTK/FPR2/IGLC7/C3/CD93/PEAR1/IGHE/IGHV2-70D/MET/MSR1/TLR2/MFGE8/PLPP4/SLC11A1/FYN/SPON2/CEACAM4/IGHV3-64
GOBP_AXIS_SPECIFICATION	GOBP_AXIS_SPECIFICATION	0.039414414	CER1/SFRP1/CHRDL1/FGF10/SIX2/GPC3/WT1/NODAL/TDRD10/SHH/SMO/STC1/PTCH1/WLS/CXXC4
GOBP_NEGATIVE_REGULATION_OF_ACTIN_FILAMENT_BUNDLE_ASSEMBLY	GOBP_NEGATIVE_REGULATION_OF_ACTIN_FILAMENT_BUNDLE_ASSEMBLY	0.039490446	MYOC/PHLDB2/CGNL1/PRKN/DLC1/ARHGAP28/MIR149/S1PR1/CORO2B
GOBP_POSITIVE_REGULATION_OF_NERVOUS_SYSTEM_PROCESS	GOBP_POSITIVE_REGULATION_OF_NERVOUS_SYSTEM_PROCESS	0.039490446	CARTPT/SOX10/WASF3/CHRNB4/HGF/TENM4/NLGN2

GOBP_BEHAVIORAL_DEFENSE_RESPONSE	GOBP_BEHAVIORAL_DEFENSE_RESPONSE	0.039490446	PENK/ATP1A2/GRPR/GRP/PDE8B/MEF2C
GOMF_TUMOR_NECROSIS_FACTOR_RECEPTOR_BINDING	GOMF_TUMOR_NECROSIS_FACTOR_RECEPTOR_BINDING	0.039539383	TNFSF10/TRAF4/STAT1/FADD/TRAP1/FASLG/TNFSF13/CD70/TNFSF9
GOBP_VENTRICULAR_CARDIAC_MUSCLE_CELL_MEMBRANE_REPOLARIZATION	GOBP_VENTRICULAR_CARDIAC_MUSCLE_CELL_MEMBRANE_REPOLARIZATION	0.03968254	ANK2/KCNH6/SCN4B/KCNJ8/KCNE4/KCNH2/CACNA2D1/SCN1B/SNTA1/GJA5/KCNJ5
GOBP_POSITIVE_REGULATION_OF_EXCITATORY_POSTSYNAPTIC_POTENTIAL	GOBP_POSITIVE_REGULATION_OF_EXCITATORY_POSTSYNAPTIC_POTENTIAL	0.03968254	RELN/GRIN2A/IGSF11/RGS4/NLGN1/ADRB2/NLGN3/DLG4/NLGN2
GOBP_EXPORT_ACROSS_PLASMA_MEMBRANE	GOBP_EXPORT_ACROSS_PLASMA_MEMBRANE	0.039759036	ATP1A2/KCNB1/KCNA5/SLC8A2/KCNT2/ATP1B2/KCNE4/KCNH2/ABCB1/SLC8A1/GJA1/MIR326/SLC29A4/ABCA1
GOBP_MACROPHAGE_MIGRATION	GOBP_MACROPHAGE_MIGRATION	0.039759036	AZU1/TAFA4/ROR2/THBS1/CX3CR1/MMP2/EMILIN1/CCL2/CD200/RARRES2/IL34/EDNRB/TNFSF18/EDN2/P2RY12

GOBP_POSITIVE_REGULATION_OF_DNA_BINDING	GOBP_POSITIVE_REGULATION_OF_DNA_BINDING	0.039759036	HAND2/NEUROD1/MYOCD/IGF1/NGF/MMP8/TRIM6/PRKN/ISL1
GOMF_CARGO_RECEPTOR_ACTIVITY	GOMF_CARGO_RECEPTOR_ACTIVITY	0.039727582	ASGR2/PRG4/VTN/SSC5D/COLEC12/LYVE1/CD36/SCARA3/CUBN/VLDLR/ENDOU/SSC4D/DAB2/FOLR2/SCARA5/ASGR1/ABCA1/HHIPL1/SCARF2/ACKR3/LRP1/LRP12/PGBD1/OLR1/ACKR2
GOCC_SPLICEOSOMAL_TRI_SNRNP_COMPLEX	GOCC_SPLICEOSOMAL_TRI_SNRNP_COMPLEX	0.039829802	SNU13/SNRPD2/PRPF18/SNRPD3/LSM5/LSM6/RNU5A-1/USP39/SNRPG/PRPF6/RBM42/CD2BP2/PRPF31/LSM3/SNRPF/LSM2/PPIH/RNU5B-1/RNU5F-1/SART1/LSM7/SNRPB/EFTUD2/TXNL4B/PRPF8/SNRNP40/SNRNP200/PRPF4/DDX23/SNRPD1/TXNL4A/SNRPA/LSM4

GOMF_MICROTUBULE_MOTOR_ACTIVITY	GOMF_MICROTUBULE_MOTOR_ACTIVITY	0.039860715	KIF20B/KIF23/KIF14/KIF9/KIFC1/KIF18A/KIF18B/KIF4A/KIF24/KIF22/KIFC2/KIF13B/KIF2C/CENPE/KIF15/KIF11/DNAH6/KIF26A
GOBP_NEGATIVE_REGULATION_OF_PROTEIN_MODIFICATION_BY_SMALL_PROTEIN_CONJUGATION_OR_REMOVAL	GOBP_NEGATIVE_REGULATION_OF_PROTEIN_MODIFICATION_BY_SMALL_PROTEIN_CONJUGATION_OR_REMOVAL	0.039876845	PER2/SPOPL/UBXN1/MINAR1/SENP2/BAG5/UBR5/SPRY2/CTNNB1/PSEN1/RELA/MAD2L2/HMG20A/GLMN/PLAA/ATG5/PARK7/RPS3/LIMK1/VPS28/OTUB1/RPS7/UFL1/DTX3L/DNAJA1/AKT1/PRMT3/PPIA/TRIM21/PINX1/RPS15/CDK5/U2AF2/FBXO5/SIRT7/BUB3/IVNS1ABP/PARP10/PRR7/HMG20B/SH3RF2/CHP1/OTUB2/BUB1B/CDC20/PRKCG/MAD2L1/TSPO

GOBP_POSITIVE_REGULATION_OF_PRODUCTION_OF_MOLECULAR_MEDIATOR_OF_IMMUNE_RESPONSE	GOBP_POSITIVE_REGULATION_OF_PRODUCTION_OF_MOLECULAR_MEDIATOR_OF_IMMUNE_RESPONSE	0.040044255	CD226/SPHK2/ATAD5/BCL10/TBX21/DENND1B/CD244/IL18R1/PLCG2/IL1B/DDX21/PYCARD/TRAF2/TFRC/MYD88/VAMP3/PTPN22/SYK/PAXIP1/GPRC5B/HLA-E/RIPK2/TLR3/HMCES/HLA-A/KMT5C/DDX1/FZD5/TICAM1/MSH2/IL18/F2RL1/PHB2/CD74/HLA-F/EPHB2/TNFRSF14/GPI/NSD2/SLC7A5/KIR2DL4/INAVA/PRKCZ/KLK5/CD55/PGC/TNFSF13/KLK7/IL10
GOMF_SERINE_TYPE_ENDOPEPTIDASE_INHIBITOR_ACTIVITY	GOMF_SERINE_TYPE_ENDOPEPTIDASE_INHIBITOR_ACTIVITY	0.040137615	ITIH2/SERPIND1/SERPINA10/TFPI2/SERPINA6/ITIH3/SERPINF2/AMBP/AGT/ITIH5/RECK/SERPINF1/SERPINA3/SERPINE2/SERPINA5/WFDC1/SERPINE1/SPOCK1/A2M/PCSK1N/PAPLN/WFDC3/COL6A3/A2ML1/WFDC10B/SERPING1/TFPI/ANOS1

GOBP_REGULATION_OF_BINDING	GOBP_REGULATION_OF_BINDING	0.040160643	GCG/HAND1/HAND2/ADIPOQ/NEUROD1/VTN/PLN/MYOCD/RGMA/IGF1/NOG/GREM2/NGF/SPON1/EBF2/MMP8/TRIM6/CDON/ADD2/DZIP1/NFATC4/LDOC1/CSF3/CAV1/SMARCD3/ANGPT1/DACT1/PLCL1/BAG2/LARP6/PRKN/HOPX/SMO/DAB2/DKK1/ADRB2/MIR27B/ISL1/LRRK2/IFIT1/SLN/ARHGAP28/LOX/TGFBR3/CLIC2/CLDN5/BLK/TWIST1/RIPOR2/MAP2/CTHRC1/P2RY1
GOBP_POSITIVE_REGULATION_OF_STEROL_TRANSPORT	GOBP_POSITIVE_REGULATION_OF_STEROL_TRANSPORT	0.040201005	APOA1/ADIPOQ/ABCA8/CES1/CAV1/ABCA1/PTCH1/PLTP/ENPP7/LRP1
GOBP_DETECTION_OF_MECHANICAL_STIMULUS	GOBP_DETECTION_OF_MECHANICAL_STIMULUS	0.04035309	KCNA1/HTR2A/CDH2/CXCL12/SERPINE2/PKD2/PIEZO2/CXCR4/KIT/BACE1/PKD1/FYN/PJVK/PDZD7

GOBP_POSITIVE_REGULATION_OF_TELOMERASE_ACTIVITY	GOBP_POSITIVE_REGULATION_OF_TELOMERASE_ACTIVITY	0.040364683	HSP90AB1/CTNNB1/PNKP/MAP3K4/PARN/HSP90AA1/MAP2K7/XRCC5/NAF1/ACD/MYC/DKC1/MAPK3/TCP1/PTGES3/NEK2/CCT2/KLF4/CCT4/AURKB/WRAP53/PRKCQ/TNKS/GRHL2/MAPK15
GOMF_PEPTIDE_BINDING	GOMF_PEPTIDE_BINDING	0.040363269	BCHE/APOA1/ADCYAP1R1/NGFR/CMA1/NPFFR2/GLP2R/GRPR/NPR3/GRIN2A/CRYAB/GHR/CCKAR/PTH1R/VIPR2/NLGN1/LEPR/CD36/APBB1/NMUR1/NPR1/TGFB2/CLSTN2/BDKRB1/CACNA1A/AVPR1A/SCTR/ADRB2/TRHDE/CCKBR/GLP1R/CD1C/SLC7A9/OXTR/GPRASP2/SSTR2/AVPR2/ENPEP/PRNP/RAMP2/NPR2/INHBA/APBA1/CLU/FCGR2B/LDLRAD3/CD1E/ACVR1/LRP1/NPY6R/EDNRB/FZD4/BACE1/RAMP3/PRLR/FPR2/DLGAP3/GSTM2/RAMP1/KCNIP2/GPR37/PTH2R
GOBP_STARTLE_RESPONSE	GOBP_STARTLE_RESPONSE	0.040446304	KCNA1/PENK/GRIN2A/GLRB/PRKN/SLITRK6

GOBP_CORONARY_VASCULATURE_MORPHOGENESIS	GOBP_CORONARY_VASCULATURE_MORPHOGENESIS	0.040446304	HAND2/SGCD/FGF2/TGFBR3/TBX5/PDGFRB/NRP1
GOBP_NEGATIVE_REGULATION_OF_CELL_SUBSTRATE_JUNCTION_ORGA	GOBP_NEGATIVE_REGULATION_OF_CELL_SUBSTRATE_JUNCTION_ORGANIZATION	0.040446304	APOD/PHLDB2/THBS1/FAM107A/DLC1
GOBP_RESPONSE_TO_REACTIVE_OXYGEN_SPECIES	GOBP_RESPONSE_TO_REACTIVE_OXYGEN_SPECIES	0.040456432	MPO/HP/TACR1/CRYAB/APOD/KCNA5/PTPRN/CYP1B1/DDR2/NR4A3/FBLN5/TPM1/CD36/MAPT/HGF/SCGB1A1/SLC8A1/PKD2/MMP2/IL6/APOA4/HBB/PDGFRA/SOD3/LRRK2/PDGFD/SESN3/RHOB
GOMF_PROTEASOME_BINDING	GOMF_PROTEASOME_BINDING	0.040637322	USP14/PSMF1/BAG6/PSMG1/RAD23B/PSME4/UCHL5/PSMD14/ZFAND1/ECPAS/ADRM1/ID1/RAD23A

GOMF_TRANSMEMBRANE_RECEPTOR_PROTEIN_KINASE_ACTIVITY	GOMF_TRANSMEMBRANE_RECEPTOR_PROTEIN_KINASE_ACTIVITY	0.040770102	EPHA7/AMHR2/EFEMP1/EPHA3/ROR2/DDR2/PDGFRL/ROS1/LTBP1/FGFR1/NRP2/PDGFRA/EFNB3/BMPR1B/TGFBR3/TEK/FLT3/MUSK/ROR1/KIT/ACVR1/PDGFRB/AXL/NRP1/MERTK/TIE1/FGFR2/LTBP4/ENG/MET/NTRK2
GOMF_TRANSLATION_REPRESSOR_ACTIVITY	GOMF_TRANSLATION_REPRESSOR_ACTIVITY	0.040770102	MIR483/TRIM71/MIR145/MIR520C/SAMD4A/MIR342/MIR27B/MIR326/MIR149/CELF4/MIR222/MIR32/MIR10B/MIR148B/NANOS1/ZNF540/MIR196A1/MIR613/MIR644A/MIR339/MIR3909
GOBP_CARDIAC_MUSCLE_CELL_PROLIFERATION	GOBP_CARDIAC_MUSCLE_CELL_PROLIFERATION	0.040914561	NKX2-5/NOG/KCNK2/ZFPM2/FGF2/GLI1/TGFB2/RBP4/FOXC2/TENM4/TGFBR3/MEF2C/MIR222/TBX5/NDRG4/FGFR2

GOBP_POSITIVE_REGULATION_OF_ESTABLISHMENT_OF_PROTEIN_LOCALIZATION_TO_MITOCHONDRION	GOBP_POSITIVE_REGULATION_OF_ESTABLISHMENT_OF_PROTEIN_LOCALIZATION_TO_MITOCHONDRION	0.041105299	VPS11/ARIH2/PRKAA1/UBE2L3/USP36/MAPK8/HTRA2/NMT1/SAE1/ATG13/CSNK2A2/HUWE1/UBE2D3/BAP1/RAC2/UBE2J2/GSK3A/HPS4/SREBF2/MICALL2/FZD5/BBC3/GZMB
GOBP_SOMATIC_STEM_CELL_POPULATION_MAINTENANCE	GOBP_SOMATIC_STEM_CELL_POPULATION_MAINTENANCE	0.041079812	SFRP1/FGF10/MIR145/SIX2/NOG/SALL4/NODAL/SMO
GOBP_SENSORY_PERCEPTION_OF_LIGHT_STIMULUS	GOBP_SENSORY_PERCEPTION_OF_LIGHT_STIMULUS	0.041081081	COL2A1/KERA/GLRB/EFEMP1/HMCN1/RDH8/CACNB2/PRPH2/GJC1/LUM/NDP/CRB2/RBP4/RLBP1/CDH23/SLITRK6/CNGA1/CLDN19/TGFBI/USH1G/LRAT/TIMP3/CRYBA2/CHRNB2/SEMA5B/COL1A1/BEST1/CRYBB1/PDE6B/CLIC5

GOBP_TEMPERATURE_HOMEOSTASIS	GOBP_TEMPERATURE_HOMEOSTASIS	0.04109589	ADIPOQ/FABP4/CIDEA/ADCYAP1/NPR3/HTR2A/NOVA1/EBF2/PTGER3/CNR1/LEPR/CD36/CAV1/ZNF423/PLCL1/ESRRG/ADRB2/FFAR4/LAMA4/DIO2/OXTR/WNT10B/SLN/GJA1/FOXC2/PDGFC/GADD45G/MFAP2/CXCR4/ADAMTS5/OXT/EDNRB/PRLR/EDN2
GOBP_NEGATIVE_REGULATION_OF_CARDIAC_MUSCLE_TISSUE_GROWTH	GOBP_NEGATIVE_REGULATION_OF_CARDIAC_MUSCLE_TISSUE_GROWTH	0.041184041	NOG/KCNK2/PI16/RGS4/RGS2/RBP4
GOBP_RESPONSE_TO_COPPER_ION	GOBP_RESPONSE_TO_COPPER_ION	0.041309803	HSF1/SOD1/TFRC/MT1A/MT1F/MT1M/ATP7B/MT1DP/CDK1/SORD/ATP5F1D/MT1E/MT1H/MT1G/AQP2

GOBP_RNA_PHOSPHODIESTER_BOND_HYDROLYSIS_ENDONUCLEOLYTIC	GOBP_RNA_PHOSPHODIESTER_BOND_HYDROLYSIS_ENDONUCLEOLYTIC	0.041389204	RPS21/PIWIL2/PRORP/NUDT16/NOL9/ERN1/ANG/POP5/SMG6/ZC3H12D/DBR1/PLD6/RNASET2/SLFN13/KRI1/CPSF3/RIDA/KHNYN/TSEN34/TSEN2/POP4/ABT1/BMS1/UTP20/RCL1/INTS11/SND1/POP7/TSR1/NOP9/RPP25/AGO2/YBEY/NUDT12/ELAC2/TBL3/RRS1/NOB1/ZC3H12A/BOP1/POP1/EXO1/FEN1/ENDOG/PIWIL1/NOP14/ERN2
GOBP_AXON_EXTENSION	GOBP_AXON_EXTENSION	0.041394336	SLIT2/SLIT3/MAP1B/PLXNA4/NDN/CXCL12/SEMA3E/NTN1/MAPT/NKX6-1/EDNRA/L1CAM/NRP2/FN1/NLGN3/SEMA6A/SEMA6D/ISLR2/NRCAM/SEMA6C/SEMA3G/DCLK1/MAP2/SEMA5B/SEMA3D/SEMA3C/NRP1/EDN2

GOMF_NUCLEAR_VITAMIN_D_RECEPTOR_BINDING	GOMF_NUCLEAR_VITAMIN_D_RECEPTOR_BINDING	0.041477661	SNW1/RXRA/MED30/MED12/MED13/TOB2/MED14/THRAP3/MED16/MED1/MED24
GOBP_REGULATION_OF_CYSTEINE_TYPE_ENDOPEPTIDASE_ACTIVITY_INVOLVED_IN_APOPTOTIC_SIGNALING_PATHWAY	GOBP_REGULATION_OF_CYSTEINE_TYPE_ENDOPEPTIDASE_ACTIVITY_INVOLVED_IN_APOPTOTIC_SIGNALING_PATHWAY	0.041477661	MMP9/SMAD3/PARK7/PIH1D1/PLAUR/BAX/FAS/CAAP1/NLE1/TFAP4/PRR7/LGALS9/JAK2
GOCC_INTRINSIC_COMPONENT_OF_PEROXISOMAL_MEMBRANE	GOCC_INTRINSIC_COMPONENT_OF_PEROXISOMAL_MEMBRANE	0.041477661	FIS1/PEX12/PEX11A/PEX26/PEX16/PEX3/PEX13/PEX11G/ABCD1/SLC25A17/PEX11B/PEX2/FAR1/SLC27A2
GOBP_RECEPTOR_INTERNALIZATION	GOBP_RECEPTOR_INTERNALIZATION	0.041484716	RSPO1/HTR2B/GREM1/CACNG4/SFRP4/CALY/CD36/CAV1/ANGPT1/HPCA/SNAP25/RAB31/DKK1/NTF3/DLG4/RAMP2/CXCR2/CXCR1/ITGB3/SELE/ARC/ACKR3/LRP1/RAMP3/DNM1/RAMP1/NEDD4

GOBP_PROTEIN_N_LINKED_GLYCOSYLATION	GOBP_PROTEIN_N_LINKED_GLYCOSYLATION	0.04151252	GAL3ST1/B4GALT1/ALG3/MAGT1/NUDT14/UGGT1/ALG8/UBE2J1/RFT1/FUT9/NUS1/RPN1/TMEM165/MGAT3/DPAGT1/MGAT5/MOGS/PGM3/STT3B/ALG10/DERL3/ALG6/FUT8/STT3A/DOLPP1/DHDDS/MGAT2/PMM2/DDOST/ALG1/GFPT1/MGAT4B/MLEC/MPDU1/ALG10B/FUT3
GOCC_NEURON_PROJECTION_CYTOPLASM	GOCC_NEURON_PROJECTION_CYTOPLASM	0.041573034	GRIK3/MAP1A/CALY/MAPT/DLG2/HPCA/KIF1A/KIF5A/UCHL1/LRRK2/TMEM108/DLG4/NEFL/HSPB1/DST/WASF1/MAP2/ARC/AP3B2

GOBP_MORPHOGENESIS_OF_EMBRYONIC_EPITHELIUM	GOBP_MORPHOGENESIS_OF_EMBRYONIC_EPITHELIUM	0.041755889	HAND1/SFRP1/FGF10/TRIM71/RGMA/ALDH1A2/AR/SFRP2/NOG/GREM1/TGFB1I1/PRICKLE1/SALL4/NODAL/TBX18/TGFB2/SHH/OSR1/DLC1/GDF7/WNT2B/CC2D2A/ALDH1A3/PTCH1/TWIST1/IRX3/VEGFC/CTHRC1/SULF1/VANGL2/PRKACB/BMP5/FGFR2/FZD1/SOX11/FUZ

GOCC_RESPIRASOME	GOCC_RESPIRASOME	0.041820529	NDUFB5/NDUFC1/NDUFA1/COX7B/NDUFB4/UQCRB/COX6B1/NDUFS8/UQCR11/NDUFB2/COA6/COX7C/NDUFA8/HIGD1A/COX7A2L/NDUFA6/NDUFB10/C2orf69/NDUFS5/COX7A2P2/NDUFB3/NDUFA12/NDUFA5/UQCRQ/NDUFA9/NDUFB6/NDUFA11/NDUFAF1/NDUFS6/UQCRFS1/NDUFA10/NDUFS2/HIGD2A/NDUFA2/NDUFS1/COX7A2/NDUFS3/SDHD/NDUFB7/COX5B/BCS1L/NDUFAB1/UQCR10/COX4I1/COX6A1/NDUFA13/CYCS/SDHB/COX15/COX5A/UQCRC2/UQCRFS1P1/UQCRH/COX8A/SDHA/RAB5IF/NDUFV1/FOXRED1/TTC19/NDUFS7/C15orf48/UQCRC1/UQCRHL/CYC1/COX6B2
GOMF_C_ACYLTRANSFERASE_ACTIVITY	GOMF_C_ACYLTRANSFERASE_ACTIVITY	0.041957166	HADHA/SCP2/SPTLC1/ACSM1/ACAA1/SPTLC2/ACAT2/GCAT/SPTSSB/ACSM3
GOBP_REGULATION_OF_HETEROTYPIC_CELL_CELL_A	GOBP_REGULATION_OF_HETEROTYPIC_CELL_CELL_ADHESION	0.041939712	APOA1/ADIPOQ/FGA/FGG
GOMF_PROTEIN_LIPID_COMPLEX_BINDING	GOMF_PROTEIN_LIPID_COMPLEX_BINDING	0.041939712	APOA2/APOA1/COLEC12/GPIHBP1/CD36/MAPT/THBS1/VLDLR

GOCC_SAGA_TYPE_COMPLEX	GOCC_SAGA_TYPE_COMPLEX	0.041963314	KAT14/TAF6/ENY2/ATXN7/TAF4/YEATS2/TAF9/TAF12/TAF2/TADA3/TRRAP/TAF5L/USP22/TADA1/DR1/TADA2B/ZZZ3/TAF6L/ATXN7L3/POLE3/WDR5/KAT2A/SF3B5/TAF5/SF3B3
GOMF_ICOSANOID_RECEPTOR_ACTIVITY	GOMF_ICOSANOID_RECEPTOR_ACTIVITY	0.042028986	PTGFR/PTGER3/CYSLTR1/PTGIR/PTGER1/PTGDR/TBXA2R
GOBP_MITOCHONDRION_LOCALIZATION	GOBP_MITOCHONDRION_LOCALIZATION	0.042067308	ALB/MAP1B/MAPT/MARK1/FEZ1/UCHL1/LRRK2/NEFL/WASF1
GOBP_CELLULAR_COMPONENT_MAINTENANCE	GOBP_CELLULAR_COMPONENT_MAINTENANCE	0.042253521	PRTN3/MYOCD/FERMT2/ADGRB3/NLGN1/CNTNAP1/C1QL1/BSN/KIRREL1/DLG4/PRNP/MTSS1/NLGN2/FCGR2B
GOBP_NEGATIVE_REGULATION_OF_BLOOD_VESSEL_ENDOTHELIAL_CELL_MIGRATION	GOBP_NEGATIVE_REGULATION_OF_BLOOD_VESSEL_ENDOTHELIAL_CELL_MIGRATION	0.042288557	MIR483/MEOX2/FGF2/ATP2B4/THBS1/MIR149/MEF2C/MMRN2/MIR196A1/STARD13/ANGPT2/TBXA2R/HDAC5

GOBP_POSITIVE_REGULATION_OF_LEUKOCYTE_ADHESION_TO_VASCUL	GOBP_POSITIVE_REGULATION_OF_LEUKOCYTE_ADHESION_TO_VASCULAR_ENDOTHELIAL_C	0.042328042	ELANE/SELP/IL6
GOBP_RETROGRADE_VESICLE_MEDIATED_TRANSPORT_GOLGI_TO_ENDOPLASMIC_RETICULUM	GOBP_RETROGRADE_VESICLE_MEDIATED_TRANSPORT_GOLGI_TO_ENDOPLASMIC_RETICULUM	0.042441567	COPB2/COG3/RAB6A/ARF4/COPA/ERGIC1/ARCN1/KIF1C/ARF3/GBF1/BET1L/SEC22B/ARF5/COG4/KDELR1/COPG2/TMEM115/KDELR2/SCYL1/COPZ1/BNIP1/ATP9B/TAPBP/GOLPH3L/COG7/KDELR3/RER1/ZW10/COPE/RINT1/LMAN2/PICK1/STX18/HTT/NBAS/RAB6D
GOCC_TRANSCRIPTION_ELONGATION_FACTOR_COMPLEX	GOCC_TRANSCRIPTION_ELONGATION_FACTOR_COMPLEX	0.042441567	EAF2/CDC73/ELL/ELOB/CDK13/ERCC6/NELFE/ELOC/CCNT2/MLLT1/ELOA/CDK12/SUPT16H/CCNT1/CTR9/CCNK/LEO1/SUPT5H/PEX2/EAF1/ICE2/ELOF1/NELFCD/NELFB/SSRP1/NELFA/TTF2/ELL3

GOBP_REGULATION_OF_PROTEIN_DEPOLYMERIZATION	GOBP_REGULATION_OF_PROTEIN_DEPOLYMERIZATION	0.042410714	STMN2/ACTN2/LMOD1/TMOD1/CFL2/MAP1B/SVIL/NAV3/MAP1A/ADD2/FGF13/LMOD3/HDGFL3/SLN/BMERB1/PLEKHH2
GOCC_VOLTAGE_GATED_CALCIUM_CHANNEL_COMPLEX	GOCC_VOLTAGE_GATED_CALCIUM_CHANNEL_COMPLEX	0.042471042	CACNA1C/CACNG4/CACNA1H/HSPA2/CACNB2/CACNA2D3/CACNA2D1/CACNA1A
GOBP_RESPONSE_TO_INTERLEUKIN_1	GOBP_RESPONSE_TO_INTERLEUKIN_1	0.042529989	CCL16/SFRP1/NLRP7/PCSK1/ANKRD1/PTGIS/FGG/GHR/CCL26/CCL21/AKAP12/CAMP/CCL19/IL1R1/MMP2/IL6/CCL2/CCL11/HAS2/FGB

GOBP_REGULATION_OF_PROTEIN_BINDING	GOBP_REGULATION_OF_PROTEIN_BINDING	0.042663892	GCG/ADIPOQ/VTN/RGMA/NOG/GREM2/SPON1/CDON/ADD2/DZIP1/NFATC4/LDOC1/CSF3/CAV1/SMARCD3/ANGPT1/DACT1/PLCL1/BAG2/PRKN/HOPX/DAB2/DKK1/ADRB2/MIR27B/LRRK2/IFIT1/LOX/TGFBR3/CLDN5/BLK/MAP2/CTHRC1
GOMF_WIDE_PORE_CHANNEL_ACTIVITY	GOMF_WIDE_PORE_CHANNEL_ACTIVITY	0.042789046	GJB1/GJD3/GJC3/VDAC1/GSDMD/VDAC3/TOMM40/VDAC2/GJB4/GJA3/PRF1/GJB3/GJB5
GOBP_ENDOSOME_TO_LYSOSOME_TRANSPORT_VIA_MULTIVESICULAR_BODY_SORTING_PATHWAY	GOBP_ENDOSOME_TO_LYSOSOME_TRANSPORT_VIA_MULTIVESICULAR_BODY_SORTING_PATHWAY	0.042782465	VPS4A/UBXN6/MVB12A/CHMP6/CHMP2B/CHMP7/CHMP1A/VPS4B/CHMP5/VCP/CHMP4C
GOBP_PROTEIN_QUALITY_CONTROL_FOR_MISFOLDED_OR_INCOMPLETELY_SYNTHESIZED_PROTEINS	GOBP_PROTEIN_QUALITY_CONTROL_FOR_MISFOLDED_OR_INCOMPLETELY_SYNTHESIZED_PROTEINS	0.042887613	AKIRIN2/LONP2/UBE2W/UGGT2/YME1L1/OMA1/HDAC6/UFD1/CUL3/BAG6/DERL1/UGGT1/STUB1/SDF2L1/AUP1/NGLY1/VCP/RNF126/CLPP/LONP1

GOBP_MUSCLE_CELL_APOPTOTIC_PROCESS	GOBP_MUSCLE_CELL_APOPTOTIC_PROCESS	0.042889391	HAND2/APOH/MYOCD/NKX2-5/MIR145/IGF1/SFRP2/AGT/DMPK
GOBP_RESPONSE_TO_CAMP	GOBP_RESPONSE_TO_CAMP	0.042889391	AQP8/PENK/ADIPOQ/CDO1/AKAP6/CYP1B1/WT1/PDE2A/FOSB/SLC8A1/PKD2/MMP19/IGFBP5/WNT10B/THBD/SLC26A3/STC1
GOBP_TRANSCRIPTION_INITIATION_AT_RNA_POLYMERASE_II_PROMOTER	GOBP_TRANSCRIPTION_INITIATION_AT_RNA_POLYMERASE_II_PROMOTER	0.043033694	E2F3/CCNH/MED28/TAF1/GTF2E1/MED22/PSMC6/TBP/MED10/CDK4/TAF13/TAF4/GTF2B/GTF2A1/ERCC3/ELOB/ERCC6/HMGB1/TAF9/MED30/ELOC/TAF12/MED12/TAF2/MED8/DR1/MED11/ELOA/ZNF451/MED27/GTF2H2/MED13/CDK7/MED14/POLR2D/TAF6L/MED25/MED23/TAF4B/MED16/MED15/MED9/THRA/HNF1A/PPM1D/PAXIP1/GTF2F1/MED26/TAF5/MED1/MED18/GTF2E2/GTF2A2/MAZ/CTNNBIP1/HNF1B/TP53/MED24/E2F2
GOBP_POSITIVE_REGULATION_OF_RECEPTOR_SIGNA	GOBP_POSITIVE_REGULATION_OF_RECEPTOR_SIGNALING_PATHWAY	0.043113715	JAK2/HES5/IL10/CSH1

GOMF_HISTONE_BINDING	GOMF_HISTONE_BINDING	0.043118485	ING3/KAT6A/IPO9/MYSM1/KDM8/BRD4/RBBP7/ASF1A/THAP7/MTF2/ING5/FMR1/BAZ1B/TRIM24/SBNO1/CHD1/SMARCA5/SIRT1/PHF19/YEATS4/TSPYL1/PHIP/WDTC1/TDRD3/DAXX/MLLT1/RNF168/UIMC1/SUZ12/MLLT6/PHF6/DNAJC2/TP53BP1/CBX3/HINFP/ZZZ3/BRD7/PYGO2/RBBP5/SCML1/DEK/ZNHIT1/SMARCC1/HAT1/PARP9/USP3/PSME4/KAT5/SMARCC2/PRMT7/HDGFL2/SPHK2/BAZ2A/SART3/PIH1D1/SFMBT1/ING2/MSH6/NAP1L4/SETSIP/GRWD1/DTX3L/HIRA/ZZEF1/INO80/NASP/BRD2/LRWD1/L3MBTL2/ANP32A/SET/CKS1B/KMT2D/SPTY2D1/SRCAP/ANP32B/ANP32E/MCM3AP/RBBP4/KDM1B/WDR5/SAMD13/HJURP/NCAPD2/CBX4/SPIN4/SSRP1/KMT5C/VRK1/PTMA/CARM1/KDM4A/MPND/CBX2/WRAP53/CXXC1/STAT1/[illegible]
GOMF_PEPTIDOGLYCAN_BINDING	GOMF_PEPTIDOGLYCAN_BINDING	0.043235704	REG3G/REG1B/PGLYRP1
GOMF_HYALURONIC_ACID_BINDING	GOMF_HYALURONIC_ACID_BINDING	0.043235704	ITIH2/SUSD5/CHODL/LYVE1/LAYN
GOBP_ACTIVATION_OF_TRANSMEMBRANE_RECEPTOR_PROTEIN_TYROSINE_KINASE_ACTIVITY	GOBP_ACTIVATION_OF_TRANSMEMBRANE_RECEPTOR_PROTEIN_TYROSINE_KINASE_ACTIVITY	0.043235704	CHRNA3/EPGN/GREM1/ANGPT1/ADRB2/PDGFC/PRLR/EREG
GOBP_REGULATION_OF_INTRINSIC_APOPTOTIC_SIGNALING_PATHWAY_IN_RESPONSE_T	GOBP_REGULATION_OF_INTRINSIC_APOPTOTIC_SIGNALING_PATHWAY_IN_RESPONSE_TO_DNA_DAMAGE_BY_P53_	0.043225652	ATAD5/ING2/HNRNPK/KDM1A/TRIAP1/CD74/MUC1/ELL3

GOBP_RESPONSE_TO_NUTRIENT	GOBP_RESPONSE_TO_NUTRIENT	0.043196544	BCHE/PENK/SFRP1/ADIPOQ/ASCL1/ALDH1A2/CYP24A1/SFRP2/MAP1B/CYP1B1/CNR1/F7/MN1/KANK2/TNFRSF11B/SLC8A1/NR1H4/A2M/FOLR2/SNAI2/LPL/STC1/KL/LRAT
GOBP_STRIATED_MUSCLE_CELL_APOPTOTIC_PROCESS	GOBP_STRIATED_MUSCLE_CELL_APOPTOTIC_PROCESS	0.043425814	HAND2/MYOCD/NKX2-5/MIR145/SFRP2/AGT
GOCC_SYNAPTIC_CLEFT	GOCC_SYNAPTIC_CLEFT	0.043478261	ADGRB3/NLGN1/NPTX1/LAMA2/C1QL1/COLQ/EGFLAM
GOBP_CELLULAR_GLUCAN_METABOLIC_PROCESS	GOBP_CELLULAR_GLUCAN_METABOLIC_PROCESS	0.043478261	PYGM/PPP1R1A/SORBS1/PPP1R3C/IGF1/IGF2/LEPR/RUBCNL/PHKG1/PPP1R3G/GABARAPL1/IL6ST/GYG1/PYGL/IRS1/EPM2AIP1/GNMT/PPP1R3F/NHLRC1
GOBP_REGULATION_OF_GLUCAN_BIOSYNTHETIC_PRO	GOBP_REGULATION_OF_GLUCAN_BIOSYNTHETIC_PROCESS	0.04353562	SORBS1/PPP1R3C/IGF1/IGF2

GOBP_ISOPRENOID_METABOLIC_PROCESS	GOBP_ISOPRENOID_METABOLIC_PROCESS	0.043572985	PNLIP/CEL/ADH1B/ALDH1A2/TTR/CYP1B1/RDH8/ADH1A/ADH7/UGT1A7/RBP4/RLBP1/HSD17B6/CRABP2/RARRES2/LRAT/ALDH1A3/CYP3A4/CYP1A1/RBP1/ADH4/LRP1
GOBP_POSITIVE_REGULATION_OF_TUMOR_NECROSIS_FACTOR_SUPERFAMILY_CYTOKINE_PRODUCTION	GOBP_POSITIVE_REGULATION_OF_TUMOR_NECROSIS_FACTOR_SUPERFAMILY_CYTOKINE_PRODUCTION	0.043572985	AZU1/C1QTNF4/MMP8/IL33/CD36/NFATC4/AKAP12/THBS1/CCL19/IL6/ZBTB20/ORM2/MIR27B/ISL1/LRRK2/LPL/HSPB1/LILRA2/LY96/CLU/TWIST1
GOBP_REGULATION_OF_OSTEOBLAST_PROLIFERATION	GOBP_REGULATION_OF_OSTEOBLAST_PROLIFERATION	0.043758044	SFRP1/NPR3/TNN/GREM1/MN1/CCN1/TMEM119/NELL1/ITGB3/CTHRC1
GOCC_INTRINSIC_COMPONENT_OF_SYNAPTIC_VESICLE_MEMBRANE	GOCC_INTRINSIC_COMPONENT_OF_SYNAPTIC_VESICLE_MEMBRANE	0.043969849	SYT4/ATP6V1G2/SLC17A7/SV2A/RAB3B/RAB3C/SYT1/SYP/SYNGR1
GOBP_SERINE_FAMILY_AMINO_ACID_METABOLIC_PROCESS	GOBP_SERINE_FAMILY_AMINO_ACID_METABOLIC_PROCESS	0.043938318	BAAT/GART/MPST/DHFRP1/SHMT1/TST/DHFR/MTHFD1/SHMT2/PSAT1/SLC7A11/CTH

GOBP_POSITIVE_REGULATION_OF_PROTEIN_LOCALIZATION	GOBP_POSITIVE_REGULATION_OF_PROTEIN_LOCALIZATION	0.043956044	PLA2G1B/GCG/UCN3/TMEM35A/NNAT/KCNB1/FGA/NGFR/PCSK1/ANKRD1/SORBS1/F2/IGF1/FGG/RIC3/FERMT2/EPHA3/CLIP3/FLNA/PRKAA2/MYOM1/MAP1A/GLI3/GIP/PRKD1/TGFB3/DZIP1/MAPT/ATP2B4/APBB1/RFX6/NKX6-1/STAC/TGFB2/TENM1/HPCA/RBP4/WWTR1/PRKN/MYH10/NR1H4/SHH/SMO/SYT11/ISL1/STOM/GPR27/DLG4/PTP4A3/PRNP/FGB/GNAI1/NLGN2/BLK/CORO2B/GPLD1/GLIS2/MYRIP/WLS/VEGFC

GOBP_CELLULAR_ION_HOMEOSTASIS	GOBP_CELLULAR_ION_HOMEOSTASIS	0.044	ELANE/CASQ2/ATP1A2/PLN/KCNMA1/CASQ1/JPH2/SCGN/ANK2/AKAP6/F2/SLC8A2/HTR2A/RGN/DMD/RYR3/HTR2B/PRND/FLNA/ATP1B2/CACNA1C/FGF2/TRPC4/TF/PTH1R/TRPC1/CXCL12/CYBRD1/HRC/SLC22A17/PRKD1/AGT/CCL21/SYPL2/SV2A/P2RX1/ATP2B4/CCL19/MICU3/DMPK/CAV1/S100A8/EDNRA/TMEM178A/SLC30A2/CKB/SLC8A1/PKD2/BDKRB1/S100A9/PRKCB/CDH23/AVPR1A/CLIC4/STEAP4/RHCG/SLC9A9/LRRK2/SLC24A3/SCARA5/CCL11/PRNP/SLC26A3/ITPR1/RYR2/STC1/SLC34A3/CLIC2/THY1/JPH3/ANXA6/MCOLN3/ITGB3/SLC30A10/P2RY1/GPER1/CCR7/TMPRSS6/SLC46A1/STC2/RAB39A/HAMP
GOBP_REGULATION_OF_UBIQUITIN_PROTEIN_LIGASE_ACTIVITY	GOBP_REGULATION_OF_UBIQUITIN_PROTEIN_LIGASE_ACTIVITY	0.044091682	RPS15/FBXO5/FZR1/CDC14B/UBE2S/CDC20/MAD2L1/PLK1

GOBP_ENDOTHELIUM_DEVELOPMENT	GOBP_ENDOTHELIUM_DEVELOPMENT	0.044133477	TMEM100/BARX1/ARHGEF26/ZEB1/EDNRA/ATOH8/FSTL1/PDE2A/S1PR3/CLIC4/APOLD1/FOXC2/FGF1/S1PR1/SCUBE1/HEG1/SOX17/RHOB/PDPN/STC1/CLDN5/COL15A1/CXCR4/CD34/MYADM/STARD13/ACVR1/PECAM1/EDNRB/NRP1/RDX/TIE1/GJA4/FZD1/BMP6/GJA5/PPP1R12A/MET/CDH5/DLL1/SOX18/PROC/GSTM3/VCL
GOBP_REGULATION_OF_SMOOTH_MUSCLE_CELL_DIFFERENTIATION	GOBP_REGULATION_OF_SMOOTH_MUSCLE_CELL_DIFFERENTIATION	0.044191919	MYOCD/RBPMS2/MIR145/ZEB1/PRDM6/SHH/EFEMP2/GPER1/KIT/FGFR2/EREG/ENG
GOBP_HEART_TRABECULA_MORPHOGENESIS	GOBP_HEART_TRABECULA_MORPHOGENESIS	0.044247788	NKX2-5/NOG/ADAMTS1/TGFB2/RBP4/S1PR1/HEG1/TGFBR3/TEK

GOBP_NUCLEAR_TRANSCRIBED_MRNA_CATABOLIC_PROCESS_NONSENSE_MEDIATED_DECAY	GOBP_NUCLEAR_TRANSCRIBED_MRNA_CATABOLIC_PROCESS_NONSENSE_MEDIATED_DECAY	0.04425589	ETF1/PNRC2/DCP2/MAGOHB/SMG8/SKIV2L/SECISBP2/SMG6/MAGOH/GSPT1/SMG7/PARN/SMG1/SMG5/EIF3E/NCBP1/APOBEC1/SMG9/EIF4A3/EXOSC10/DHX34/PABPC1/UPF1/CASC3/RNPS1/PYM1/NBAS/HELZ2/HNRNPAB
GOBP_ANATOMICAL_STRUCTURE_MATURATION	GOBP_ANATOMICAL_STRUCTURE_MATURATION	0.044238683	SOX10/MYOC/FEV/KCNB1/ASCL1/BNC1/ALDH1A2/IGF1/PGR/FGG/ADGRB3/ZBTB16/GREM1/PTH1R/LGI4/PDE3A/CCL21/RECK/CCL19/NKX6-1/EDNRA/CX3CR1/C1QL1/TGFB2/MMP2/TDRD10/RHEX/CABYR/LRRK2/WNT10B/DCHS1/LHX6/S1PR1/SLC26A3/PAEP/NRCAM/NPR2

GOBP_POSITIVE_REGULATION_OF_CELL_CYCLE_G2_M_PHASE_TRANSITION	GOBP_POSITIVE_REGULATION_OF_CELL_CYCLE_G2_M_PHASE_TRANSITION	0.04431518	RRM2B/FBXO5/STOX1/CDC25C/NPM1/CDK1/RRM1/RCC2/CCNB1/DTL/CDC7/CCND1/CDC25A
GOBP_POSITIVE_REGULATION_OF_CELL_KILLING	GOBP_POSITIVE_REGULATION_OF_CELL_KILLING	0.044339954	KLRD1/CD226/CYRIB/AZGP1/HLA-H/SYK/HLA-E/IL23A/NECTIN2/AP1G1/HLA-DRA/HLA-B/IL12RB1/KLRC2/HLA-A/F2RL1/HLA-F/PVR/TAP2/HLA-DRB1/FADD/NOS2/LAG3/PRF1/IFNG
GOBP_AXONAL_FASCICULATION	GOBP_AXONAL_FASCICULATION	0.044414536	EPHA3/CRTAC1/CNR1/NDN/NCAM2/CNTN4/NRCAM/NRP1/AMIGO1/SEMA5A/RTN4/EPHA4
GOBP_GLYCOSYL_COMPOUND_CATABOLIC_PROCESS	GOBP_GLYCOSYL_COMPOUND_CATABOLIC_PROCESS	0.044664554	APOBEC3F/APOBEC3B/FUCA1/NAGA/GLA/GDA/APOBEC3D/FUCA2/DPYD/NUDT1/AHCY/ABHD10/APOBEC3G/APOBEC3H/APOBEC1/PNP/MTAP/XDH/GBA2/CDA/ADA

GOBP_ACTIN_CYTOSKELETON_REORGANIZATION	GOBP_ACTIN_CYTOSKELETON_REORGANIZATION	0.044711014	FGF10/NRK/FGF7/ASB2/THSD7B/FLNA/HMCN1/SEMA3E/CSF3/PDLIM4/EFS/THSD7A/PDGFRA/NTF3/ANTXR1/PARVA/SYDE1/S1PR1/CLDN19/TEK/PHACTR1/KIT/GAB1/GSN/NRP1
GOBP_ORGANIC_ACID_TRANSPORT	GOBP_ORGANIC_ACID_TRANSPORT	0.044715447	PLA2G1B/SYT4/SPX/ATP1A2/FABP4/SLC13A5/PLA2G5/SLC38A3/RGS4/TRPC4/PLA2G2C/SLC7A2/AGT/SLC17A7/CD36/SV2A/PLA2G12B/THBS1/SLC27A6/SLC7A10/SLC38A4/RGS2/RBP7/FABP3/SLC1A7/ABCB1/AVPR1A/SLC16A7/FOLR2/SLC10A4/SLC7A9/CRABP2/GJA1/SLC29A4/SLC26A3/PDPN/ABCC2/APBA1/GABBR1/SLC10A6/SLC16A2/SLC16A10/PLIN2/SLC5A12/RBP1/RBP5/SLC46A1/SLC23A1/OXT/SLC51B/PRAF2/CLTRN/ABCG2/SLC47A1/SLC26A2/PLA2R1/AQP9/SLC16A11/SLC36A4/SLC51A/NTRK2/RBP2/SNCA/ABCD2/SLC11A1

GOBP_MITOCHONDRIAL_OUTER_MEMBRANE_PERMEABILIZATION	GOBP_MITOCHONDRIAL_OUTER_MEMBRANE_PERMEABILIZATION	0.044808383	BID/SLC35F6/GSK3A/RTL10/BAX/SLC25A5/RHOT2/ATP5IF1/ZNF205/HIP1R/CHCHD10/VDAC2/BAK1/TMEM102/BBC3/GZMB
GOBP_MAMMARY_GLAND_EPITHELIAL_CELL_DIFFERENTIATION	GOBP_MAMMARY_GLAND_EPITHELIAL_CELL_DIFFERENTIATION	0.044880785	FGF2/FOXF1/SMO/PTCH1/HOXA5/PRLR/LBH
GOCC_PSEUDOPODIUM	GOCC_PSEUDOPODIUM	0.044880785	LDB3/ACTN2/KLHL41
GOMF_FIBROBLAST_GROWTH_FACTOR_RECEPTOR_BINDING	GOMF_FIBROBLAST_GROWTH_FACTOR_RECEPTOR_BINDING	0.044880785	FGF10/FLRT2/FGF7/FGF2/KLB/FGF1/KL
GOMF_MYOSIN_BINDING	GOMF_MYOSIN_BINDING	0.044982699	MYOC/MYL9/DMD/MYL3/CALD1/ACTA1/ACTC1/LARP6/RAB6B/KIRREL1/RAB3B/RAB3C/MYRIP/CXCR4/SHROOM4/GSN/RAB39B/AXL/MYL4

GOBP_MULTICELLULAR_ORGANISMAL_HOMEOSTASIS	GOBP_MULTICELLULAR_ORGANISMAL_HOMEOSTASIS	0.04509018	ALB/CARTPT/COL2A1/ADIPOQ/NEUROD1/FABP4/CIDEA/ADCYAP1/NPR3/HTR2A/DMD/NOVA1/CST4/ATP1B2/EBF2/PRKAA2/PTGER3/TF/JAM2/CNR1/PTH1R/NDN/TUB/JAM3/NR4A3/LAMA2/FMO1/LEPR/CD36/VSTM4/CDHR1/PDK4/CUBN/PIP/AKT3/CAV1/ZNF423/CRB2/SERPINA3/ANGPT1/RBP4/CYTL1/PLCL1/WWTR1/TNFRSF11B/TMEM119/COMP/IL6/ESRRG/CDH23/SH3GL2/COL3A1/SMO/KRT1/ADRB2/SGIP1/FFAR4/LAMA4/DIO2/TNS2/CTSK/ACER1/OXTR/WNT10B/SLN/GJA1/FOXC2/CCN2/S1PR1/FMO2/HAS2/USH1G/HSPB1/NOX4/CLDN5/LTBP3/LAMC1/LDB2/MUC2/PDGFC/GADD45G/MFAP2/NANOS1/IRX3/ITGB3/ACACB
GOBP_FATTY_ACID_BIOSYNTHETIC_PROCESS	GOBP_FATTY_ACID_BIOSYNTHETIC_PROCESS	0.045258621	PLA2G1B/APOA1/PLP1/APOC3/PTGIS/RGN/ELOVL2/PRKAA2/GIP/ELOVL4/PDK4/HACD4/HACD1/APOA4/AVPR1A/FADS6/PTGDS/LPL/PTGS1/QKI/CYP3A4/ACACB/CYP1A1/ELOVL5/LTC4S/FADS2/EDN2/THNSL2/GSTM2/LIPC

GOBP_RESPIRATORY_SYSTEM_PROCESS	GOBP_RESPIRATORY_SYSTEM_PROCESS	0.045454545	ATP1A2/NDN/CCBE1/PBX3/NLGN3/TSHZ3/TCF15/NLGN2/HOXA5
GOBP_POSITIVE_REGULATION_OF_GLUCOSE_IMPORT	GOBP_POSITIVE_REGULATION_OF_GLUCOSE_IMPORT	0.045454545	ADIPOQ/SORBS1/IGF1/GPC3/ITLN1/KLF15
GOBP_REGULATION_OF_LEUKOCYTE_ADHESION_TO_VASCULAR_ENDOTHELIAL_CELL	GOBP_REGULATION_OF_LEUKOCYTE_ADHESION_TO_VASCULAR_ENDOTHELIAL_CELL	0.045454545	ELANE/CXCL12/CCL21/SELP/IL6/MIR222/SELE
GOBP_RESPONSE_TO_ENDOPLASMIC_RETICULUM_STRESS	GOBP_RESPONSE_TO_ENDOPLASMIC_RETICULUM_STRESS	0.045585405	BAG6/PPP1R15B/USP19/UBE4A/RHBDD2/UFM1/FAF1/DERL1/FAF2/UBXN8/EIF2B5/SERP1/UGGT1/UBE2J2/CDK5RAP3/PARK7/UBE2J1/ERP29/PDIA3/UBAC2/GORASP2/UBXN4/BCL2L1/UBQLN1/STUB1/GET4/CREB3/TMBIM6/OS9/DNAJC3/BFAR/LPCAT3/HM13/SELENOS/NR1H3/SDF2L1/MAN1B1/ATP2A3/ANKZF1/DNAJB12/PDIA6/RACK1/PARP16/AUP1/EIF4G1/UFL1/SYVN1/MAP3K5/YOD1/COPS5/PLA2G6/STT3B/PDIA4/BAX/CALR/CCDC47/TMEM129/HSPA5/PML/ECPAS/ATP2A2/CANX/DERL3/TRAF2/NGLY1/SESN2/MANF/EIF2S1/TMX1/TBL2/BCAP31/HSP90B1/APAF1/AIFM1/SGTA/ANKS4B/TMUB1/DDRGK1/EDEM3/TXNDC12/CASP4/TMEM259/VCP/SVIP/PDX1/P4HB/DAB2IP/SEC16A/ERLIN1/EDEM1/TMEM33/CREB3L1/HYOU1/[illegible]

GOBP_REGULATION_OF_PROTEIN_POLYMERIZATION	GOBP_REGULATION_OF_PROTEIN_POLYMERIZATION	0.045643154	STMN2/LMOD1/SLIT2/TMOD1/CCL26/TUBB4A/MAP1B/CLIP3/PAK3/SVIL/NAV3/DAAM2/ELN/ADD2/CDC42EP3/CCL21/MAPT/KANK4/CSF3/LMOD3/TENM1/FHOD3/ANKRD53/KIRREL1/CCL11/ARHGAP28/TMSB15A/HSPA1A/CYRIA/MAP2/CCR7/MYADM/GSN/RDX/ARHGAP40/PFN2/TMOD2/TMSB4Y/EVL/MET/TBK1/KANK3/CDH5/MAPRE3/SNCA
GOBP_RESPONSE_TO_AUDITORY_STIMULUS	GOBP_RESPONSE_TO_AUDITORY_STIMULUS	0.045760431	ATP1A2/TACR1/NRXN2/PTN/SLITRK6
GOBP_POSITIVE_CHEMOTAXIS	GOBP_POSITIVE_CHEMOTAXIS	0.045783133	AZU1/CCL16/FGF10/FGF7/FGF2/CXCL12/F7/HGF/ANGPT1/NTF3/S1PR1/SCG2/GPNMB/VEGFC

GOBP_POSITIVE_REGULATION_OF_LEUKOCYTE_MEDIATED_IMMUNITY	GOBP_POSITIVE_REGULATION_OF_LEUKOCYTE_MEDIATED_IMMUNITY	0.045793434	KLRD1/CD226/ATAD5/CYRIB/AZGP1/PTAFR/TBX21/DENND1B/IL18R1/HLA-H/PLCG2/IL1B/DDX21/TRAF2/TFRC/PAXIP1/HLA-E/IL23A/NECTIN2/AP1G1/HLA-DRA/HLA-B/IL12RB1/KLRC2/HMCES/HLA-A/KMT5C/SH2D1B/DDX1/FZD5/TICAM1/MSH2/HSPD1/IL18/F2RL1/CD177/HLA-F/PVR/TAP2/HLA-DRB1/FADD/NSD2/KIR2DL4/PRKCZ/NOS2/LAG3/CD55/TNFSF13
GOBP_MITOCHONDRIAL_TRANSCRIPTION	GOBP_MITOCHONDRIAL_TRANSCRIPTION	0.046054507	TFB2M/THAP11/TFAM/SLC25A33/PPARGC1B/TFB1M/MRPL12/TWNK/POLRMT/CHCHD10

GOMF_S_ADENOSYLMETHIONINE_DEPENDENT_METHYLTRANSFERASE_ACTIVITY	GOMF_S_ADENOSYLMETHIONINE_DEPENDENT_METHYLTRANSFERASE_ACTIVITY	0.046137106	LCMT1/NSD3/ASH2L/EEF2KMT/PEMT/SUV39H2/SMYD2/BUD23/CAMKMT/THUMPD3/THUMPD2/SETD2/EEF1AKMT4/TFB2M/TRMO/SETDB1/RBBP5/TRMT61B/SMYD5/FTSJ1/EHMT1/FTSJ3/MEPCE/PRMT7/SETD9/KMT2B/METTL15/SETD1A/NSUN4/TRMT11/METTL2A/METTL2B/METTL21A/TRMT10A/ZCCHC4/FDXACB1/ICMT/KMT5A/NSD1/TRMT2A/NSUN2/EHMT2/SETD6/NSUN5/ARMT1/PRDM16/FBL/MRM1/KMT2D/CMTR2/PRMT3/WDR4/CARNMT1/WDR5/MRM3/TFB1M/SUV39H1/NTMT1/PRMT1/KMT5C/COMTD1/ANTKMT/COQ3/CARM1/TRMT1/CXXC1/EZH2/PRMT5/MECOM/TPMT/DOT1L/CSKMT/NSD2/DNMT1/TTLL12
GOBP_POSITIVE_REGULATION_OF_EPITHELIAL_CELL_MIGRATION	GOBP_POSITIVE_REGULATION_OF_EPITHELIAL_CELL_MIGRATION	0.046267087	TACR1/FGF10/FGF7/SMOC2/FGF2/PRKD1/AGT/AKT3/THBS1/ATOH8/FGFR1/ANGPT1/TGFB2/RHOJ/NRP2/MIR342/CCBE1/MIR27B/FOXC2/FGF1/ADGRA2/HAS2/SRPX2/RHOB/HSPB1/TEK/MIR222/SPARC/MIR10B/GPLD1/ITGB3/VEGFC

GOBP_MALE_GENITALIA_DEVELOPMENT	GOBP_MALE_GENITALIA_DEVELOPMENT	0.046321526	FGF10/AR/ROR2/WT1/GREB1L/SHH/PDGFRA
GOBP_PURINE_NUCLEOSIDE_METABOLIC_PROCESS	GOBP_PURINE_NUCLEOSIDE_METABOLIC_PROCESS	0.046440685	HPRT1/NT5C2/GDA/DGUOK/NUDT1/AHCY/APRT/ICMT/ADK/PNP/OARD1/MTAP/PGM2/XDH/ACP3/MACROD2/MACROD1/ADA
GOBP_MORPHOGENESIS_OF_AN_EPITHELIAL_BUD	GOBP_MORPHOGENESIS_OF_AN_EPITHELIAL_BUD	0.046376812	FGF10/AR/NOG/SHH/WNT2B/SULF1/FGFR2/TP63
GOBP_REGULATION_OF_CYTOSOLIC_CALCIUM_ION_CONCENTRATION	GOBP_REGULATION_OF_CYTOSOLIC_CALCIUM_ION_CONCENTRATION	0.04642409	PLN/SCGN/F2/SLC8A2/TRPC4/TRPC1/HRC/P2RX1/ATP2B4/CAV1/TMEM178A/SLC8A1/CDH23/ITPR1/RYR2/P2RY1/GPER1
GOBP_NEURON_PROJECTION_REGENERATION	GOBP_NEURON_PROJECTION_REGENERATION	0.046428571	RGMA/APOD/GAP43/MAP1B/FLNA/PTN/TNC/HGF/MMP2/APOA4/PRRX1/ISL1/NEFL/THY1/MIR222
GOBP_MESODERM_MORPHOGENESIS	GOBP_MESODERM_MORPHOGENESIS	0.046457607	HAND1/MIR145/SIX2/SFRP2/NOG/ITGA8/FOXF1/CRB2/NODAL/DKK1/GJA1/FOXC2/INHBA/ITGB3/WLS/LEF1/ACVR1

GOBP_COPPER_ION_TRANSPORT	GOBP_COPPER_ION_TRANSPORT	0.046849318	SLC11A2/SLC31A2/ATP7B/SLC31A1/FKBP4/HEPHL1/CP/STEAP3
GOBP_POSITIVE_REGULATION_OF_RESPONSE_TO_EXTERNAL_STIMULUS	GOBP_POSITIVE_REGULATION_OF_RESPONSE_TO_EXTERNAL_STIMULUS	0.047047047	AZU1/AGTR1/APOH/FABP4/VTN/THBS4/FGF10/F2/SLIT2/PLA2G5/CCL26/NPY/FLNA/PAK3/PTN/SMOC2/FGF2/PTGER3/CNR1/MMP8/TRIM6/CXCL12/F7/IL33/SERPINF2/PRKD1/AGT/COLEC11/CCL21/TSLP/THBS1/CCL19/S100A8/CX3CR1/CREB3L3/LPAR1/PDE2A/FGFR1/FCN1/IL6/S100A9/NR1H4/SERPINE1/EMILIN1/NTF3/LRRK2/PDGFD/CCN4/S1PR1/THBD/RAET1G/LPL/CXCR2/RARRES2/HSPB1/CAMK1D/SCG2/MIR222/LILRA2/LY96/LY86/S100A12/RIPOR2/MMRN2/VEGFC/CLNK/LGALS1/CXCR4/CCR7/IL6ST/IL34/PDGFRB/FFAR2/RAET1E/TLR7/NRP1/MNDA/TNFSF18/EDN2/FPR2/HPX/P2RY12/C3/CD28/CADM1/BMP6/EREG/NCR3/NLRP3/PLAT/GHRL/MET/TTBK1/CCL7/SNCA/TLR2/SEMA5A/FYN/IL1RL1/VEGFB/C3AR1[illegible]
GOBP_REGULATION_OF_SKELETAL_MUSCLE_TISSUE_DEVELOPMENT	GOBP_REGULATION_OF_SKELETAL_MUSCLE_TISSUE_DEVELOPMENT	0.047169811	IGF2/CDON/LMOD3/SHH/WNT10B/MEF2C/TWIST1/ACVR1

GOBP_NUCLEOBASE_CONTAINING_SMALL_MOLECULE_CATABOLIC_PROCESS	GOBP_NUCLEOBASE_CONTAINING_SMALL_MOLECULE_CATABOLIC_PROCESS	0.047226419	APOBEC3F/APOBEC3B/GDA/APOBEC3D/DPYD/NUDT1/AHCY/ENTPD7/GSK3A/APOBEC3G/APOBEC3H/APOBEC1/ENTPD4/PNP/MTAP/XDH/CDA/ADA
GOBP_NEGATIVE_REGULATION_OF_DNA_REPLICATION	GOBP_NEGATIVE_REGULATION_OF_DNA_REPLICATION	0.047226419	SMARCAL1/LIG3/S100A11/RAD17/TTF1/GTPBP4/ZRANB3/WAPL/ATR/GMNN/CDAN1/DONSON/BRCA2/PDS5A/FBXO5/TIPIN/TIMELESS/BLM/TP53/MCIDAS/CDT1

GOBP_NEGATIVE_REGULATION_OF_CELL_ACTIVATION	GOBP_NEGATIVE_REGULATION_OF_CELL_ACTIVATION	0.04742268	FGL1/SFRP1/BPI/CTSG/F2/FGG/ADAMTS18/CNR1/GLI3/PRKG1/FOXF1/CNR2/FGL2/SCGB1A1/SERPINE2/SHH/EMILIN1/PDGFRA/PGLYRP1/SYT11/TSPAN32/CD200/THBD/PRNP/LRRC32/GPNMB/PDE5A/INHBA/INHA/BLK/FCGR2B/RIPOR2/IL13RA2/CLNK/IL20RB/GPER1/TNFRSF13B/LGALS7B/AXL/CR1/MNDA/MERTK/TNFSF18/SOX11/DUSP3/CYGB/VSIG4/ILDR2/HLX/VTCN1/CST7/ADGRF5/TNFSF4/XCL1/TWSG1/MILR1/SOCS5/C1QTNF1
GOBP_PROTEIN_LOCALIZATION_TO_PHAGOPHORE_ASSEMBLY_SITE	GOBP_PROTEIN_LOCALIZATION_TO_PHAGOPHORE_ASSEMBLY_SITE	0.047506852	WIPI2/PACS2/ATG13/ATG4B/MFN2/ATG9A/ATG16L1/ARFIP2/WDR45B/LRBA/ATG9B/ATG4D
GOBP_PEPTIDE_CROSS_LINKING	GOBP_PEPTIDE_CROSS_LINKING	0.047619048	DCN/IVL/F13A1/TGM1/THBS1/SPRR3/COL3A1/FN1/CSTA/KRT1/BGN/NDNF/EGFLAM/SPRR1A

GOMF_CHEMOREPELLENT_ACTIVITY	GOMF_CHEMOREPELLENT_ACTIVITY	0.047619048	APOA1/EPHA7/FLRT2/SEMA3E/SEMA6A/SEMA6D/SEMA6C/SEMA3G/SEMA5B/SEMA3D/SEMA3C
GOBP_NEURAL_NUCLEUS_DEVELOPMENT	GOBP_NEURAL_NUCLEUS_DEVELOPMENT	0.047619048	PLP1/INA/ASCL1/FOXP2/FGF2/SYPL2/CKB/GNB4/ALDH1A3/CHRNB2
GOBP_UBIQUITIN_DEPENDENT_PROTEIN_CATABOLIC_PROCESS_VIA_THE_MULTIVESICULAR_BODY_SORTING_PATHWAY	GOBP_UBIQUITIN_DEPENDENT_PROTEIN_CATABOLIC_PROCESS_VIA_THE_MULTIVESICULAR_BODY_SORTING_PATHWAY	0.04780155	HDAC6/VPS36/VPS37A/VPS37D/VPS4A/UBAP1/MVB12A/VPS37C/PLAA/CHMP6/VPS28/CHMP2B/CHMP7/CHMP1A/VPS4B/HGS/CHMP5/PTPN23/TOM1L1/RNF126/VPS37B/CHMP4C
GOBP_NEGATIVE_REGULATION_OF_RECEPTOR_SIGNALING_PATHWAY_V	GOBP_NEGATIVE_REGULATION_OF_RECEPTOR_SIGNALING_PATHWAY_VIA_JAK_STAT	0.04787234	NEUROD1/DAB1/PTPRD/CAV1
GOBP_REGULATION_OF_KIDNEY_DEVELOPMENT	GOBP_REGULATION_OF_KIDNEY_DEVELOPMENT	0.048040455	ADIPOQ/SIX2/NOG/GREM1/WT1/AGT/SMO/OSR1/WNT2B
GOBP_PROTEIN_TARGETING_TO_PEROXISOME	GOBP_PROTEIN_TARGETING_TO_PEROXISOME	0.048031784	PEX12/LONP2/PEX6/PEX26/PEX16/PEX3/PEX13/PEX5/PEX2/HACL1/PEX1/PEX14/PEX7/PEX10

GOBP_PIGMENT_BIOSYNTHETIC_PROCESS	GOBP_PIGMENT_BIOSYNTHETIC_PROCESS	0.047995783	SLC11A2/SLC25A39/SLC25A38/ATP5IF1/SLC6A9/COX15/COX10/TMEM14DP/CPOX/ALAS1/GIPC1/CDH3/HMBS/MFSD12/TSPO/OCA2/SLC7A11
GOMF_UBIQUITIN_LIKE_PROTEIN_PEPTIDASE_ACTIVITY	GOMF_UBIQUITIN_LIKE_PROTEIN_PEPTIDASE_ACTIVITY	0.047980306	ATG4A/USP15/USP31/SENP8/USP34/USP40/USP36/USP14/COPS4/DESI2/USP42/MYSM1/USP39/OTUD4/DESI1/SENP2/USP8/UCHL3/UFD1/ZUP1/ATG4B/OTUD3/USP22/SENP1/OTULIN/BAP1/USP45/USP19/STAMBP/SENP3/USP33/PSMD7/HINT1/USP5/JOSD1/EIF3H/VCPIP1/USP3/USP37/PAN2/OTUB1/USP10/UCHL5/USP28/OTUD6B/USP48/PRPF8/BRCC3/YOD1/COPS5/PSMD14/USP7/USP24/USP46/USP38/STAMBPL1/USP18/USP1/USP54/ZC3H12A/OTUB2/USP43
GOBP_CELLULAR_RESPONSE_TO_COPPER_ION	GOBP_CELLULAR_RESPONSE_TO_COPPER_ION	0.04813191	HSF1/MT1A/MT1F/MT1M/MT1DP/MT1E/MT1H/MT1G/AQP2

GOCC_CLATHRIN_COATED_ENDOCYTIC_VESICLE_MEMBRANE	GOCC_CLATHRIN_COATED_ENDOCYTIC_VESICLE_MEMBRANE	0.048178613	CHRM2/SLC18A3/APOB/EPGN/ROR2/TF/SH3GL2/ADRB2/SGIP1/AVPR2/SYT1/TYRP1/IL7R/CD207/FZD4
GOBP_STRIATED_MUSCLE_CELL_PROLIFERATION	GOBP_STRIATED_MUSCLE_CELL_PROLIFERATION	0.048165138	NKX2-5/NOG/KCNK2/ZFPM2/FGF2/GLI1/HGF/ANGPT1/TGFB2/RBP4/SHH/FOXC2/TENM4/TGFBR3/MEF2C/MIR222/TBX5
GOBP_AMINE_METABOLIC_PROCESS	GOBP_AMINE_METABOLIC_PROCESS	0.048192771	HAND2/GRIN2A/INSM1/AOC3/PAH/INMT/NPY/RNF180/ATP2B4/EDNRA/NPR1/TGFB2/PDE1B/PRKN/NR1H4/MOXD1/HDC/SLC29A4/KL/NNMT/CYP1A1/CHRNB2
GOBP_INTERMEMBRANE_LIPID_TRANSFER	GOBP_INTERMEMBRANE_LIPID_TRANSFER	0.048250905	APOA2/APOA1/APOB/TNFAIP8L3/APOA4/ABCA1/MTTP
GOMF_LIPID_TRANSFER_ACTIVITY	GOMF_LIPID_TRANSFER_ACTIVITY	0.048250905	APOA2/APOA1/APOB/TNFAIP8L3/APOA4/ABCA1/MTTP

GOBP_EPITHELIAL_TO_MESENCHYMAL_TRANSITION_INVOLVED_IN_ENDOCARDIAL_CUSHION_FORMATION	GOBP_EPITHELIAL_TO_MESENCHYMAL_TRANSITION_INVOLVED_IN_ENDOCARDIAL_CUSHION_FORMATION	0.048452221	TMEM100/NOG/TGFB3/TGFB2/HEYL/SNAI2/ACVR1/ENG/SNAI1/TGFBR1
GOBP_LOW_DENSITY_LIPOPROTEIN_PARTICLE_CLEARANCE	GOBP_LOW_DENSITY_LIPOPROTEIN_PARTICLE_CLEARANCE	0.048492792	APOC3/ADIPOQ/APOB/SOAT2/CD36/NR1H4/MIR27B
GOBP_POSITIVE_REGULATION_OF_BINDING	GOBP_POSITIVE_REGULATION_OF_BINDING	0.048677249	GCG/HAND2/NEUROD1/VTN/MYOCD/IGF1/NGF/SPON1/EBF2/MMP8/TRIM6/ADD2/CSF3/CAV1/DACT1/PLCL1/LARP6/PRKN/ISL1/LRRK2/CLIC2/CLDN5/BLK/TWIST1/RIPOR2/CTHRC1
GOCC_SCHAFFER_COLLATERAL_CA1_SYNAPSE	GOCC_SCHAFFER_COLLATERAL_CA1_SYNAPSE	0.048809524	INA/EPHA7/PTPRD/ADCY1/AKAP12/CDH11/BSN/LRRC4/NEFL/LRRC4C/ITPR1/APBA1/GABBR1/SYP/NEFH/PFN2/TAMALIN/NTNG2/PLAT/GHRL

GOBP_ERYTHROCYTE_HOMEOSTASIS	GOBP_ERYTHROCYTE_HOMEOSTASIS	0.048796253	DNASE2/SLC11A2/UFL1/RPS19/RPS6/SLC25A38/SLC25A5/MFHAS1/ZNF16/RPS17/SCNN1B/THRA/ATP5IF1/PRKDC/PTBP3/HMGB2/MED1/HSPA9/PRMT1/SLC25A40/CASP3/TSPO2/ETV2/MAEA/ZFPM1/P4HTM/STAT1/DMTN/RCOR1/ALAS1/CDK6/SFXN1/NCAPG2/GPI/HOXB6/LYN/TRIM10/JAK2/MYB/KCNQ1
GOBP_NEGATIVE_REGULATION_OF_CELL_MATRIX_ADHESION	GOBP_NEGATIVE_REGULATION_OF_CELL_MATRIX_ADHESION	0.048994975	MYOC/APOD/PHLDB2/SEMA3E/THBS1/SERPINE1/FAM107A/DLC1

GOBP_RNA_POLYMERASE_II_PREINITIATION_COMPLEX_ASSEMBLY	GOBP_RNA_POLYMERASE_II_PREINITIATION_COMPLEX_ASSEMBLY	0.049144622	TAF6/MED4/MED31/CAND1/MED28/TAF1/MED22/PSMC6/TBP/MED10/TAF13/TAF4/GTF2B/GTF2A1/HMGB1/TAF9/MED30/TAF12/TAF2/MED8/DR1/MED11/MED27/MED14/TAF6L/MED25/MED23/MED16/MED15/MED9/THRA/MED26/TAF5/MED1/MED18/GTF2A2/TP53/MED24
GOBP_NEUROTRANSMITTER_METABOLIC_PROCESS	GOBP_NEUROTRANSMITTER_METABOLIC_PROCESS	0.049202128	BCHE/PRIMA1/PAH/MOXD1/COLQ
GOBP_PRESYNAPTIC_ENDOCYTOSIS	GOBP_PRESYNAPTIC_ENDOCYTOSIS	0.049353702	SLC2A4/STON1/NLGN4X/NLGN1/AMPH/SLC17A7/NLGN4Y/SH3GL2/NLGN3/LRRK2/SNCG/NLGN2/SYT1/SYP/AP3B2/SYT5

GOCC_ENDOCYTIC_VESICLE	GOCC_ENDOCYTIC_VESICLE	0.049395161	ELANE/MPO/HP/APOA1/CHRM2/SLC18A3/APOB/EPGN/PLA2G5/RAB9B/ROR2/TF/COLEC12/CACNG4/CD36/RAB23/CUBN/CSF3/CAV1/PIP4P2/LPAR1/SH3GL2/AVPR1A/HBB/SMO/RAB31/PGLYRP1/ADRB2/SGIP1/SYT11/FFAR4/NLGN3/VIM/DLG4/GPR161/SCGB3A2/EHD2/RAB34/AVPR2/ABCA1/MTSS1/SPARC/PTCH1/SYT1/WLS/TYRP1/IL7R/LRP1/HBA2/RAB39A/GSN/CD207/TLR7/FZD4/CLTCL1/ATP6V0D2/HPX
GOBP_POSITIVE_REGULATION_OF_MEMBRANE_PERMEABILITY	GOBP_POSITIVE_REGULATION_OF_MEMBRANE_PERMEABILITY	0.049432017	SLC35F6/GSK3A/HEBP2/RTL10/BAX/SLC25A5/RHOT2/ATP5IF1/ZNF205/HIP1R/PPIF/CHCHD10/VDAC2/TP53/BAK1/TMEM102/BBC3/SLC9A1/GZMB

GOBP_NEGATIVE_REGULATION_OF_CYTOKINE_PRODUCTION	GOBP_NEGATIVE_REGULATION_OF_CYTOKINE_PRODUCTION	0.049445005	ELANE/APOA2/APOA1/ADIPOQ/BPI/NLRP7/CIDEA/MIR145/F2/APOD/MIR520C/IGF1/SSC5D/NAV3/MMP8/IL33/TGFB3/THBS1/CX3CR1/HGF/ANGPT1/TGFB2/SCGB1A1/IL6/NR1H4/FN1/PGLYRP1/SYT11/MIR27B/FFAR4/LILRA4/CD200/MIR149/PRNP/LRRC32/GPNMB/MIR222/INHBA/INHA/FCGR2B/TWIST1/IL20RB/CCR7/CD34/LEF1/LGALS7B/AXL/SRGN/CR1
GOBP_CD8_POSITIVE_ALPHA_BETA_T_CELL_ACTIVATION	GOBP_CD8_POSITIVE_ALPHA_BETA_T_CELL_ACTIVATION	0.049557464	PTPN22/HLA-E/HLA-A/CBFB/SOCS1/SH3RF1/ZBTB7B/IRF1/CD274
GOCC_INTEGRIN_COMPLEX	GOCC_INTEGRIN_COMPLEX	0.049681529	PLP1/ITGA7/ITGA8/ITGA9/ITGBL1/ITGA1/ITGA5/EMILIN1/TSPAN32/ITGB3/ITGA11
GOBP_CHEMOKINE_PRODUCTION	GOBP_CHEMOKINE_PRODUCTION	0.049778761	ELANE/AZU1/ADIPOQ/ADCYAP1/APOD/ACKR1/CXCL6/IL33/TSLP/IL6/NR1H4

GOBP_VASCULAR_ASSOCIATED_SMOOTH_MUSCLE_CONTRACTION	GOBP_VASCULAR_ASSOCIATED_SMOOTH_MUSCLE_CONTRACTION	0.049803408	HTR2A/ACTA2/AGT/P2RX1/EDNRA/SLC8A1/COMP
GOBP_POSITIVE_REGULATION_OF_AMINO_ACID_TRANSPORT	GOBP_POSITIVE_REGULATION_OF_AMINO_ACID_TRANSPORT	0.049865229	SYT4/SLC38A3/AGT/AVPR1A/GABBR1/CLTRN/ACE2/NTSR1/KMO/P2RX7/STXBP1
